# Supplementary material for: Prevalence and correlates of SARS-CoV-2 seropositivity among people who inject drugs in the San Diego-Tijuana border region
Source: PLoS One. 2021 Nov 22;16(11):e0260286. doi: 10.1371/journal.pone.0260286 (PMC8608290; doi:10.1371/journal.pone.0260286)
Supplement: S1 File — (DOCX) [file pone.0260286.s001.DOCX]

| UCSD Human Research Protections Program **RESEARCH PLAN** |
| --- |
| 1. **PROJECT TITLE** |
| Ethno-epidemiology of HCV, HIV and Overdose associated with Drug Markets and Drug Tourism |
| **2. PRINCIPAL INVESTIGATOR** |
| Steffanie Strathdee, Ph.D., Professor and Harold Simon Chair, Associate Dean of Global Health Sciences, Division of Infectious Disease and Global Public Health, UCSD Department of Medicine |
| **3. FACILITIES** |
| The UCSD investigators and staff for this project have offices on the main UCSD campus. This will include the UCSD Center for AIDS Research (CFAR) laboratory where HIV+ and HCV+ samples will be sequenced and where SARS-CoV-2 RNA-PCR will be conducted. This will also include Dr. Jack Gilbert’s lab that will test fecal matter for microbiome testing in our substudy. The UCLA investigators and staff for this project have offices on the main UCLA campus.  The field operations for the study will be carried out in part on the streets and collaborating community health clinics in Tijuana and San Diego. Eligibility assessment and recruitment will be conducted in the community where the target population is at in Tijuana and San Diego. Genalyte Inc. will manage SARS-CoV-2/COVID-19 antibody testing serology for our sub-studies. The US Centers for Disease and Control (CDC) laboratory will manage sequencing for our SARS-CoV-2/COVID-19 samples. |
| **4. ESTIMATED DURATION OF THE STUDY** |
| Main study: 5 years. (2020-2025)  Sub-study #1: 2 years. (2020-2022)  Sub-study #2: 2 years (2021-2023) |
| **5. LAY LANGUAGE SUMMARY OR SYNOPSIS (no more than one paragraph)** |
| **Main Study**:The goal of the present study (La Frontera) is to describe the characteristics of new cases of Human Immunodeficiency Virus (HIV) and Hepatitis C Virus (HCV) infection and drug overdose (OD) among people who inject drugs (PWID) related to drug markets and drug tourism between San Diego, (CA, US) and Tijuana (BC, Mexico).  **Sub-study #1 (COVID Prevalence and Network features):** The goal of the sub-study is to determine the prevalence and correlates of SARS-CoV2 infection among PWID in La Frontera and the extent to which PWID who test positive are shedding SARS-CoV2.  **Sub-study #2 (Barriers to COVID19 testing and Vaccination):** The goal of this sub-study is to increase access and uptake of COVID-19 testing and vaccination among people who inject drugs, a population highly vulnerable to COVID-19. We will study barriers and predictors of COVID-19 testing and vaccination through La Frontera to examine behavioral, serological and health outcomes. |
| **6.** **SPECIFIC AIMS** |
| The research proposed herein is a mixed quantitative and qualitative study to explore trends in incidence of HIV, HCV, and overdose (OD) associated with binational drug markets and drug tourism (DT) between San Diego (SD) and Tijuana (TJ), Mexico. The proposed study aims to describe the following:  AIM 1. To prospectively characterize cross-border DT between SD and TJ and its association with retail drug market characteristics.  H1.1. Over time, we will observe a significant increase in DT among SD people who inject drugs (PWID), mediated by perceptions in increased availability and decreased price of drugs in TJ (i.e., heroin, fentanyl, meth and cocaine).  H1.2. PWID in TJ will be more likely to report injecting powder drug formulations (i.e. heroin and/or fentanyl) and prescription drugs relative to non-DTs (NDTs) who remain in SD.  AIM 2. To characterize retail drug market trends in TJ versus SD by conducting epidemiologic and ethnographic surveillance, triangulating quantitative and qualitative findings and DEA administrative data.  H2.1. A higher proportion of PWID in TJ will initially report fentanyl use relative to SD; this proportion will subsequently equalize in both cities.  H2.2. Heroin, fentanyl and meth will increase in availability and decrease in price in TJ vs. SD; over time, availability will synchronize in both cities but price will remain lower in TJ.  AIM 3. To study real-time HCV and HIV cross-border transmission among PWID with molecular epidemiology.  H3.1. DT will be significantly associated with new cross-border HCV and HIV transmission clusters.  H3.2. Incident HIV and HCV will show greater geographical dispersion over time by phylogeographic analysis.  H3.3. Fentanyl injection (alone & with meth) will be independently associated with HCV and HIV incidence.  AIM 4. To characterize OD incidence and its associations with drug retail markets and DT.  H4.1. Higher OD incidence will occur among DTs vs. NDTs.  H4.2. In both cities, OD incidence will be highest among co-injectors of meth and powder fentanyl compared to PWID who inject other drugs.  AIM 5. To conduct dynamic modeling of future HIV, HCV and OD incidence among PWID in SD in the context of structural determinants such as changing drug retail markets and DT to inform prevention interventions.  **Sub-Study #1 (COVID19 Prevalence and Network Features)**:  Substance users may be at higher risk of acquiring and transmitting SARSCoV-2 due to their living conditions (e.g., crowded living spaces, homelessness, incarceration) and drug-seeking behaviors (e.g. time spent on the street or in shooting galleries) as well as specific types of substance may damage cilia and lung tissue, which could predispose to SARSCoV-2 and complications (e.g., secondary bacterial infections). This sub-study is nested within La Frontera; UCSD IRB # 191390 and will address the proposed aims:  Aim 1. To determine the prevalence and correlates of subclinical and symptomatic SARSCoV-2 infection among PWID within the SD/TJ border region.  Aim 2. To determine the prevalence and correlates of SARSCoV-2 shedding in nasal secretions and fecal specimens among PWID.  Aim 3. To determine network features associated with cross-border transmission of subclinical SARSCoV-2 infection among PWID.  Aim 4: To use molecular epidemiology to determine the global migration of SARSCoV-2 between PWID in San Diego and Tijuana and factors associated with local and global dispersal.  **Sub-Study #2 (Barriers to COVID19 testing and Vaccination):**  Preliminary data from our first sub-study shows that one third of PWID i have been infected with SARS-CoV-2, of whom two thirds had not been previously tested, and only 3% had been vaccinated against COVID-19. We will amend our study protocol to assess barriers to COVID-19 testing and vaccination.. In California, the California Department of Public Health is planning to offer rapid COVID testing and vaccination at syringe service programs (SSPs), and in Tijuana, the Mexican Border Health Commission hopes to soon offer COVID-19 vaccine to marginalized communities. We hope to evaluate these efforts. Our aims are:  **Aim 1**. To prospectively evaluate the prevalence, predictors and barriers to COVID-19 testing among PWID.  H1.1. PWID who endorse COVID misinformation and conspiracy theories will be less likely to a) seek COVID-19 testing and b) wear facemasks, controlling for race/ethnicity, sex, age and vaccination status.  H1.2. PWID who know someone who died of COVID will have greater uptake of COVID-19 testing.  **Aim 2**. To prospectively assess incidence, predictors and barriers to COVID-19 vaccination among PWID.  H2.1. Incidence of vaccine uptake will be lower among PWID who encounter structural impediments such as: a) lack of transportation, b) homelessness, c) low health literacy, d) lacking health insurance and e) being undocumented as compared to their counterparts, controlling for race/ethnicity, sex and age.  H2.2. PWID with evidence of SARSCoV-2 infection will subsequently show greater uptake of COVID-19 vaccination based on longitudinal serology patterns directed against SARSCoV-2 N and S proteins.  H2.3. PWID who are homeless will have lower uptake of COVID vaccination vs. those who are not homeless.  **Aim 3**. To compare COVID testing and vaccination rates before and after the CDPH introduces a) COVID-19 rapid testing and b) COVID-19 vaccination at OnPoint, which represents a natural experiment.  H3.1 We will observe significant increases in uptake of a) COVID tests and b) vaccination after these services are co-located at OnPoint compared to earlier periods.  **Aim 4**. To prospectively assess the impact of COVID-19 testing and vaccination on PWIDs’ behaviors.  H4.1. PWID who receive COVID-19 vaccine will report greater mobility, including cross-border travel to Mexico. |
| **7. BACKGROUND AND SIGNIFICANCE** |
| The US is facing an opioid crisis like never before, disproportionately driven by fentanyl use. Opioid ODs doubled from 2013-17 coinciding with the emergence of fentanyl,^[1, 2]^ accompanied by increased incidence of HCV and sporadic HIV outbreaks.^[3, 4]^ Mexico, which is the most important source of illicit drugs in the US,^[5]^ now accounts for >90% of the heroin supply and recent US federal legislation aims to reduce Mexican-sourced drugs. Interdiction, however, may inadvertently increase incidence of HIV, HCV & OD especially if cartels increasingly shift to low volume, higher potency fentanyl formularies to avoid detection and if US drug users along the border travel to Mexico to buy, use and themselves increase micro-trafficking of drugs (i.e., drug tourism [DT]). In 2014, one in six PWID from SD travelled to TJ to inject drugs in the last months.^[6, 7]^ One reason is that in 2009, Mexico partially decriminalized drug possession in stark contrast to US drug policy. In 2015, Mexico relaxed opiate prescription laws which may facilitate black market diversion, as occurred in the US.^[8]^ We hypothesize that these divergent national drug policies will increase availability and decrease drug prices in Mexico, fueling increased DT (Aim 1). Our project, La Frontera, is uniquely positioned to study DT and retail drug markets in both SD and TJ and unintended negative health consequences such as increased penetration of fentanyl. Since TJ sits on a major transit route whereby heroin, fentanyl, cocaine and meth are trafficked to the US, we will study how retail drug markets in TJ influence those in SD (Aim 2), given that drug trends observed in SD may serve as a sentinel for other US cities.^[9]^  SD PWID who are drug tourists (DTs) to TJ are at high risk of HCV, HIV and OD since TJ’s thriving drug market abuts a zona roja where sex work is quasi-legal. Although HIV prevalence among PWID is relatively low in TJ (5%), HIV incidence in female PWIDs has risen to 2.3/100 person years -the highest among PWID in North America. We recently reported that the direction of HIV transmission has shifted from Mexico to the US for the first time^[10]^. Mexican cartels recently introduced fentanyl powder into TJ and US drug scenes where black tar heroin had predominated^[11]^. Its short half-life^[12]^ leads PWID to inject more frequently which may increase needle sharing^[13]^. In contrast to black tar that requires heating, ‘cold’ preparation of powder opiates may increase HCV and HIV transmission (see 3Dii)^[14, 15]^. La Frontera will test these hypotheses for the first time (Aim 3). Since fentanyl is more potent than heroin and is now being be sold as a powder or in combination with other drugs in TJ to unsuspecting DTs from SD, we expect higher OD rates among DTs vs. non-DTs (NDTs) (Aim 4). We will integrate quantitative and qualitative surveillance of retail drug markets in TJ and SD, administrative DEA data, longitudinal data from epidemiologic surveys and HIV and HCV sequencing data to inform dynamic models that forecast future drug trends, their health impacts and interventions to reduce HIV incidence (Aim 5), which is a high NIH priority^[16]^.  **Sub-Study #1 (COVID19 Prevalence and Network Features):**  Recent studies suggest that people with subclinical SARSCoV-2 infections, including asymptomatic infection, may be important carriers that transmit the virus to others.^44.45^ Available estimates of the proportion of asymptomatically infected persons with SARSCoV-2 range from 12.5-50%^46,47^. SARSCoV-2 viral load is heaviest in nasal secretions^21^, suggesting that sharing of paraphernalia used to snort drugs (e.g., straws, dollar bills) might serve as fomites that transmit the virus. SARSCoV-2 can also be shed in feces^48,49^, suggesting that people living in areas with poor sanitation may be more likely to acquire and transmit the virus.  The US-Mexico border region is particularly vulnerable to infectious diseases due to population mixing for work, leisure, sex work and substance use. Mexico’s permissive stance on substance use and sex work has fueled drug tourism from San Diego (SD) to Tijuana (TJ). Both cities have elevated prevalence of HIV, HCV, especially among PWID. Mexico’s health system is severely constrained and cannot support contact tracing; the federal response to COVID-19 has been slow. Moreover, many PWID live in the Tijuana River Canal and are homeless. Over half of female PWID in this region rely on sex work to support their drug addiction. Undocumented people living in SD are also at heightened risk of SARSCoV-2 since they lack health care and paid sick leave, pressuring them to continue working when ill. These factors increase the vulnerability of the US-Mexico border region to ongoing spread of SARSCoV-2. In 2009, Tijuana was a hotspot for the H1N1 epidemic, where our field team conducted surveillance among PWID and the homeless^23^, suggesting this population is at increased risk of infection. Whether underlying conditions (i.e. microbiome environment) are also increasing the risk of infection in these population need to be further investigated.  Available data suggests direct binding of SARSCoV-2 to bacterial surfaces creates a bacterial-viral complex that influences virus pathogenesis and virulence^40^. This is particularly true of influenza and Streptococcus pneumoniae^41^. The microbiome of the human skin and mucosal linings of the nasal passages are teeming with microbes that influence human health. Substance use increases the risk of acquiring a variety of microbial pathogens, possibly due to the effects of the drug on pathogenic bacterial growth^42^. For example, smoking tobacco promotes pneumococcal colonization and nasal enrichment of Fusobacterium, Gemella, and Neisseria.^43^ An important unexplored hypothesis is that drug users might be at increased risk of SARSCoV-2 due to increased growth of pathogenic microbes and interaction with SARSCoV-2 within the respiratory system.  To date, there has been almost no SARSCoV-2 testing among substance users such as PWID. Data on the prevalence of asymptomatic infection is sparse, but may range from 12.5-50%^47,48^. A better understanding of both the prevalence and factors associated with subclinical infection and SARSCoV-2 shedding is critically needed, especially in the Mexico-US border region, where border closures have been enacted to curtail the spread of SARSCoV-2. The economic cost of the border shutdown is $1.85 billion per day;^46^ although cross-border mobility has decreased significantly, drug tourism is continuing Although social distancing recommendations have been implemented widely to stop SARSCoV-2 transmission, PWID are unlikely to be able to adhere to these recommendations due to their unstable, often crowded living conditions, and their need to travel to engage in sex work or buy drugs to cope with withdrawal symptoms. In a previous study of SD PWID, 54% and 30% used non-injection and injection drugs in TJ, respectively^50^. These data suggest that PWID likely represent a ‘sentinel’ population that can be monitored to inform COVID-19 mitigation planning, including the utility of border closures. Moreover, little data exists on social network characteristics associated with more efficient propagation of SARSCoV-2 community spread. It is possible that there are distinct social network structures (i.e. particularly dense cross-border social networks) and subgroups (i.e., PWID who smoke/vape, sex workers) that may fuel community spread. SARSCoV-2 community spread, like almost all biological networks, is expected to demonstrate a property of preferential attachment, whereby new nodes (i.e. infected individuals) are more likely to join the network by connecting to an infected individual who already has many connections (i.e., infected contacts). A better understanding of the characteristics of these social network “hubs” could provide critical guidance to inform public health policy.  **Sub-Study #2 (Barriers to COVID19 testing and Vaccination)**:  More than one year into the pandemic, SARS-CoV-2 is becoming endemic.[17] In the US, herd immunity against this virus is increasingly being considered unachievable due to vaccine hesitancy and new circulating escape variants.[18] The implications are enormous: control over COVID-19 will only become possible with vaccine equity, which requires an infrastructure to offer COVID-19 testing, vaccination, boosters and health care to the most vulnerable communities. Understanding and overcoming barriers to COVID-19 testing and vaccination and developing interventions to improve their uptake is a critical public health priority. As the most populous US state, California (CA) has done an admirable job of scaling up COVID-19 vaccination. However, like the rest of the country,[19] vaccine coverage is lower among under-represented minorities, especially Blacks and Latinx.[20, 21] In SD County, the 8th largest US city, 31% of residents are Latinx and 6% are Black. Vaccine hesitancy is significantly greater among Blacks and Latinx due to historical, cultural and political factors.[22, 23] Of ~25,000-28,000 PWID in SD County, ~60% are Latinx and 10% are Black.  SSPs are undergoing expansion in CA and elsewhere. 185 SSPs operating in the US have potential as venues to reach PWID at high risk of COVID-19.[24] The CDC and CDPH publicly endorse SSPs,[25, 26] and SD County’s Supervisors recently embraced SSPs in 01/21.[27] In 2020, CDPH provided funding to 37 SSPs across the state, including our lead community partner, the SD Harm Reduction Coalition’s SSP, OnPoint. The CDPH are beginning to offer rapid COVID-19 and confirmatory PCR tests at SSPs across CA and also plan to implement COVID-19 vaccination at SSPs. However, medical mistrust and vaccine hesitancy are anticipated to be persistent barriers, which our study intends address. |
| **8. PROGRESS REPORT** |
| Due to safety concerns during the COVID-19 epidemic, the start of the study was delayed until the end of October, 2020. As of May 14^th^, 2021, we have enrolled 314 participants meeting our eligibility criteria (116 DTs and 198 NDTs). A quarter of recruited DTs are women, many who reported buying and selling drugs and engaging in sex work. |
| **9. RESEARCH DESIGN AND METHODS** |
| Design of Main Study: La Frontera is a prospective quantitative and qualitative epidemiological surveillance study of retail drug markets in both SD and TJ that will collect data on n=600 PWID: 200 SD DTs, 200 SD NDTs & 200 TJ NDTs.  Based on our experience with prior studies, we anticipate that 95% of the sample will be Hispanic/Latino and 5% will be American, of whom 2% are likely to be White and 3% Hispanic and 6% will be women. In addition, participants will range in age from 18 to 71 years (mean approx. 35); mean level of educational attainment will be 7 years; 5% will be illiterate; and 60% will have children. Pregnancy status will be assessed via self-report. No prisoners or inmates will be recruited into the study.  We will recruit 600 participants all by September 1, 2021 (see more information about recruitment process in item 11). Participants are required to be PWID who injected within the last month, since PWID are more sensitive to drug market changes and have higher risk of HIV, HCV and OD, optimizing statistical power. Proof of injection drug use will be through inspection of track marks. We require PWID to be aged ≥18 or older (minors are not permitted to cross the US border without parental accompaniment or notarized letter). We will recruit 3 groups of 200 PWID (N=600 total): 200 PWID who injected drugs in TJ ≤6 mo ago but live in SD (DTs) will be recruited and interviewed in TJ (at baseline) to ensure that they are truly border-crossers. NDTs are PWID who live in SD county (N=200) or TJ (N=200) but have never used illicit drugs across the border. Based on our previous experience, ~5% of DTs will become NDTs over time and vice versa, captured through our prospective surveys. In sensitivity analyses, we will conduct analyses with a more liberal DT definition.  Screener: Recruitment will take place in areas in which injection drug users hang out in Tijuana and San Diego. Based on our experience, injection drug use venues include bars, hotels, street corners, riverbanks, canals, canyons and shooting galleries. PWID will be approached in the field by a trained outreach worker who will ascertain the participant‘s willingness to participate in the study through a number of screening questions, asked in a conversational manner prior request of *verbal consent* to ask the screening questions. Our promoters are very familiar with these venues, as they have worked in these communities for several years and some are former injection drug users. PWID who are potentially appropriate for inclusion and willing to complete the eligibility process will be referred to the storefront office, where they will be screened and receive a detailed description of the study through the informed consent procedure. People who provide verbal consent to participate and complete the screening questionnaire will receive $5 dollars for their participation regardless of whether they are recruited into the study.  Monetary reimbursements will be provided to potential participants at the screening visits and for enrolled participants at each visit as this has been the most successful way to recruit PWID in other studies in Tijuana and San Diego. The screening visit will consist of an interviewer-administered pre-interview screener (~5 min in length) for which participants will be compensated $5. Each of the 600 eligible study participants will receive $20 each time they complete a semi-annually administered questionnaire and $5 after completion of the locator form.  Interview:  After providing written informed consent in a confidential setting (eg., interview room at storefront study site), subjects will participate in baseline interview for ~45 minutes which will either be conducted in Tijuana or in San Diego at participating county clinics. Since surveys will be administered in either English or Spanish, the Spanish version will be back-translated into English by Dr. Vera and programmed into CAPI by our data manager so interviewers can collect data on their laptops. Interview answers will be marked by the participant’s code number only and no other identifiers.  Questions will ask for the following:   1. Socio-demographics: sex at birth, gender identity, date, place of birth 2. Mobility: intra-urban travel within SD, cross border travel to TJ and elsewhere in Mexico in 6 mo 3. Substance Use: history, practices, and social-environmental influences regarding substance use in SD, TJ, and other cities 4. Retail drug market characteristics: perceived availability, potency, purity, and price of drugs, formulations (e.g., powder vs. tar, crystal, color) and perceived changes and drug preferences in last 6 mo in SD and TJ 5. HIV & HCV risk behavior: receptive and distributive syringe sharing, injection and non-injection paraphernalia (e.g. back & front loading syringes, splitting drugs in a syringe, buying and using pre-filled syringes, frequency of injection and syringe sharing, syringe cleaning, needing help injecting, rushed or public injection, injection drug use and needle sharing in jail/prison and sex provided in exchange for drugs 6. OD history: if and when they ever passed out after using drugs/couldn’t wake up/lips turn blue. 7. OD risks: injecting alone, recent incarceration or drug treatment, period of opioid cessation, co-administration of drugs/alcohol, knowingly or unknowingly injecting fentanyl, use of naloxone, suicidality and mental health diagnoses. 8. HIV and HCV Serostatus and treatment: testing history, dates of first positive results, use of ART or HCV treatment 9. Sexual Behaviors: In SD, TJ, and other foreign cities, include # and frequency of unprotected vaginal and anal sex with regular, casual and client female, male and transgender partners in the past 6 months, sex work, # of sex partners who are PWID and condom use.   HIV/HCV Testing: Each subject will undergo HIV and HCV rapid tests at baseline and semi-annually, until mid-year in Year 4. Results are available within 30 min. Rapid HIV/HCV testing will be done using Determine®, Miriad HIV/HCV Ab InTec Rapid Anti-HCV Test and Orasure. which have an overall Specificity (Sp) of 99.7% and Sensitivity (Se) of 99.8%. Sensitivity is defined as the number of positive specimens detected by the rapid assay divided by the total number of positive specimens as per the CDC testing algorithm. Specificity is defined as the number of negative specimens identified by the rapid assay divided by the total number of negative specimens as per the CDC algorithm.    A review of the literature shows that compared to MedMira HIV/HCV Combo and Chembio HCV rapid tests, OraSure had the highest sensitivity at 92.7% (95% confidence interval [CI] = 88.8%-96.5%) followed closely by Chembio's 3 blood tests at 92.1% (95% CI = 87.7%-96.4%), 91.5% (95% CI = 87.2%-95.7%), and 92.3% (95% CI = 88.4%-96.2%). The sensitivities of MedMira HIV/HCV tends to be the lowest, at 79.1% (95% CI = 72.6%-85.5%). Specificity for the OraSure was 99.8% (95% CI = 99.4%-100%); specificity for the Chembio blood tests was 99.2% (95% CI = 98.6%-99.9%), 99.4% (95% CI = 98.8%-99.9%), and 99.3% (95% CI = 98.8%-99.9%); and specificity for the MedMira was 100% and 100%. False-negative results were associated with HIV and hepatitis B core antibody serostatus.    Similarly, in a 2012 CDC study assessing the Se and Sp of HCV rapid tests the MedMira assay yielded the largest proportion of false-negative results (ranging from 8% to 19%) as compared to the Chembio (0.4–7%) and OraSure (0–6%) assays.  All these tests are approved for testing in Mexico following FDA standards. All specimens are re-tested with a second rapid test. Study participants will be informed that a non-reactive test results means that no antibodies to HIV or HCV have been detected by the tests; a positive antibody test implies current or past infection. We will repeat with a second rapid test from a manufacturer different from the initial test. In the event of a negative HCV rapid test at baseline, HCV testing will be done again at 1 week follow up to detect any false negative cases at baseline.  After the interview and HIV/HCV testing, post-test counseling for HIV/HCV testing will be performed as per guidelines by the U.S. Centers for Disease Control and Prevention. Patients testing HIV or HCV -positive will be referred to a medical doctor on-site at the municipal health clinic for follow-up. Subjects experiencing duress will be referred to local psychiatric and/or counseling services, which are also on-site. In Tijuana, we will also provide clinical referrals to the Tijuana General Hospital or municipal specialty clinic (CAPASITS) when medical attention is warranted. Counseling services and monitoring and treatment for HIV and HCV are provided by the municipal health clinic free of charge, regardless of their health insurance status. In San Diego, we will provide referrals to San Diego County Public Health Department for follow-up. Subjects who continue to report active drug use are offered individual counseling and referral for treatment of their drug use when they agree. To be able to document whether a participant referred to community clinics or public health centers received medical care, we would like to ask study participants to review and sign a HIPPA Authorization Form to be able to access information on a participant’s: a) HIV testing, anti-retroviral treatment, hospitalization, pre-exposure prophylaxis (PrEP); b) HCV testing, access to treatment, hospitalization c) SARSCoV-2 testing, vaccination, treatment, hospitalization; and d) treatment for drug overdose or addiction treatment, and e) death  Locator form: Since this is a prospective study requiring follow-up interviews, interviewers will collect from each study participant information on how to locate each participant. This information will include personally identifying signs or marks on body (such as tattoos, scars, birth marks), aliases, home and work address (if allowed to locate at either or both) and phone numbers, contact information for friends or relatives that can provide whereabouts, etc. Based on our previous studies, these locator forms have been a key component of cohort maintenance rate > 90%. The information from the locator forms so that s/he can be located at follow-up visits will be stored in locked file cabinets in locked offices at the research site, and will be destroyed once follow-up is completed. Locator forms with identifying data will be stored separately from the interview data. Study participants will receive $5 US dollars for each completed locator form.  Retention: Follow-up visits will occur in either SD or TJ and include quarterly locator check-in where contact data is updated (i.e. cell phone, regular sleeping sites, income-generating venues, shooting galleries). Semi-annual follow-up interview mirror the baseline interview with recall periods covering prior 6 m and last drug injections. Each semi-annual interview will also include HIV/HCV and drug test.  Ethnographic component: Midway through year 1, we will randomly sample 75 PWID from each of the 3 groups, (25 DT from SD, 25 NDTs from SD, and 25 NDTs in TJ) aiming for 30% females and 75% fentanyl injectors, using Bourgois’s adapted Strategically Targeted Information-Rich Respondents Protocol (STI-RRP)^[28-33]^. In this protocol, the ethnographer initiates friendly conversations with people on the street and their social interlocutors to develop a rapport with individuals who may be relevant to the research. When individuals are receptive and relevant, the ethnographer explains the topic, obtains verbal consent, and conducts open-ended conversations and observations focused on: 1) basic demographics; 2) income-generating strategies; 3) drug use patterns and preferences; 4) friendship networks; 5) relationships to institutions: criminal justice, education, social/clinical services; 6) attitudes around race and ethnicity, gender, sexuality, violence; 7) relationships to family; 8) general health status, etc. Conversations are documented in written field notes avoiding personal identification information and obscuring precise details that risk endangering anonymity. Some conversations of particular impact or importance may be audiotaped or videotaped with participants' consent. Dr. Bourgois will train other ethnographers in these participant-observation anthropological methods. Participants in the ethnographic component will be given a separate informed consent form that fully explains in detail the process (see attached ethnographic informed consent for more detailed information). Each participant in the ethnographic component will receive $40 dollars for their participation, which is approx. 1-1.5 hrs.  Since the ethnographic data collection is seeking to understand the larger context of the risk environment in order to increase the generalizability, validity, and consistency of the quantitative and modeling arms of this research project, ethnographers will be surveying a wider range of individuals who may themselves have never used drugs but are knowledgeable about the risks faced by injectors. Examples of these non-users will include: service providers, family members of injectors, peers of injectors, etc. These interviewees will be verbally consented (see item 12 for a justification to waiver documented informed consent). They will not be administered the formal interviews or be subject to formal six month follow-up contacts. Many, if not most of them, will not be people who inject drugs.  Study activities will take place on the following schedule.   \| Month \| 0 \| 3 \| 6 \| 9 \| 12 \| 15 \| 18 \| 21 \| 24 \| ... \| 60 \| 63 \| 66 \| \| --- \| --- \| --- \| --- \| --- \| --- \| --- \| --- \| --- \| --- \| --- \| --- \| --- \| --- \| \| Interview \| x \|  \| x \|  \| X \|  \| x \|  \| X \| … \| X \|  \| x \| \| Ethnography \| x \| x \| x \| x \| X \| x \| x \| x \| X \|  \| X \| x \| x \| \| HIV & HCV  tests \| x \|  \| x \|  \| X \|  \| x \|  \| X \| … \| X \|  \| x \| \| Locator check- in \| x \| x \| x \| x \| X \| x \| x \| x \| X \| … \| X \| x \| x \|   **Sub-Study #1 (COVID19 Prevalence and Network Features)**: *(Additional Measures)*  Network Survey. We will add a supplemental survey within one week of the baseline visit. Each participant will be administered a survey on COVID-19 symptoms (e.g., fever, dry cough, shortness of breath, muscle aches, loss of smell and taste), potential exposures, and a social network survey that asks them to list social network members who they have had frequent contact (e.g., meals, meetings, drug use, social gatherings, sex, sex work), in the past 14 days. It will assess the demographics of each social network member, the type of contact (physical, non-physical [i.e. in person conversation], or sexual), the total duration of each contact (less than 5 min, 5–15 min, 15 min to 1 h, 1–4 h, and 4 h or more), the average frequency of contact with each person in the past 2 weeks (daily or almost daily, about once or twice a week, just once)^36^, and their relationship to each social network member (co-worker, family member, romantic partner). The survey will also collect details on the location of the contacts; both the name and the category of the location (home, shooting gallery, party, sex work venue). These locations will allow us to assess potential hot-spots where transmission occurs, and construct a multi-level network that contains both people and events.  Specimen Collection: We will collect three nasal secretion samples per participant using dual tipped sterile polyester swabs, by asking participants to rotate the swab in their most clogged nasal passage. One of these will be used for Fluxergy SARSCoV-2 testing (N=600). For those testing SARSCoV-2 RNA+, we will subject a second nasal swab to genetic sequencing, and the third for microbiome analysis (as described below. Specimens from PWID testing SARSCoV-2 negative will be used as controls in microbiome analyses. For microbiome analysis, we will test the fecal smears of those participants who tested SARSCoV-2 positive. In addition, we will about 3cc of whole blood for SARSCoV-2 antibody testing.  SARSCoV-2 testing. SARSCoV2 testing will have the same procedures as a similar study of PWID that was recently approved by the HRPP (protocol # 201153). All study participants will be asked to provide a self-collected nasal swab sample for SARSCoV2 RNA testing (RT-PCR) which detects an active COVID19 infection. Following CDC recommendations and 2019-nCoV Real-Time RT-PCR Diagnostic Panel targeting the virus nucleocapsid (N) gene^37^, Samples will be transported to UCSD’s Center for AIDS Research lab where lab staff will use a pooling approach based on the Fluxergy system® to detect SARSCoV2 RNA. All pools testing positive will be deconvoluted to identify the positive specimen. Dr. Chaillon’s laboratory adapted and validated the Taqman protocol to quantify SARS-CoV-2 in various samples (e.g. saliva, stool, nasal swabs). SARS-CoV-2 antibody testing serology will be conducted at Genalyte labs.  Specimens testing positive for SARSCoV-2 RNA will undergo RNA extraction and stored for whole genome sequencing. The CDC in Atlanta, GA has offered to sequence our SARS-CoV- 2 positive samples at no cost, provided we can send them the extracted RNA. We will ship the extracted RNA to the CDC in Atlanta in batches stripped of all identifiers. CDC will never be given any identifying information. CDC will deposit the sequences into the Global Initiative on Sharing All Influenza Data (GISAID), which is an open access database.  Microbiome Testing. For the microbiome samples, nasal and fecal specimens will be collected in a tube containing 95% ethanol to preserve both bacterial DNA and viral RNA. DNA will be used to construct metagenomic libraries from 1 ng using the NexteraXT kit, which will be barcoded and multiplex sequenced to generate ~100 Billion bp per sample on the NovaSeq platform. Reads will be assembled de novo using IDBA-UD, annotated using RAST, and binned by taxon using tetranucleotide frequency, cross samples coverage, and %GC. Genome bins will be further assembled and curated to produce draft assemblies of microbial genomes from which specific biomarkers will be mined.  Data Analysis: For each outcome, contingency tables (2x2) will be generated for specific subgroups (e.g. PWID who inject in TJ vs not, injected fentanyl vs. not, injected prescription drugs vs. not) using proportions for categorical variables or means, medians and inter-quartile ranges for continuous variables. Hypotheses testing will primarily use generalized linear mixed models (GLMM) which address attrition, missing data and variable timing of visits. For correlations over time, we assume an autoregressive covariance structure. We will also consider potential covariates (e.g., gender, ethnicity, language, age, income).  For Aim 1, to test H1.1a: Over time, we expect significant increases in DT among SD PWID and H1.1b: The relationship between time and DT will be mediated by increased availability and decreased price of drugs in TJ. For H1.1a, we will use a Generalized Poisson Regression Model, with # of times SD PWID injected drugs in TJ in the last 6 mo as the outcome and time as the main fixed effect. For H1.1b, since our outcome is a count (Y=# times a SD PWID injected drugs in TJ within the previous 6 mo), we will use equations provided by Geldof et al (2018)^38^ to calculate conditional indirect effects to evaluate our mediation model: 1) The path between Time (X) and Price (M) will be modeled using linear regression, allowing us to derive the prediction equation for the Expected (Price)=a0+a1(Time). 2) The path between Price (M) and the # of times SD PWID injected drugs in TJ (Y) will be modeled by log-linear regression with a Poisson link, allowing us to derive the prediction equation for the Expected (#times injected drugs)=exp (β0+β1(Price)+C1’ (Time)). 3) The conditional indirect effects for various levels of Time (i.e., visit 1, visit 2, etc…) will be computed by multiplying the first partial derivative of the prediction equation for Price with respect to Time (=a1) with the first partial derivative of the prediction equation for # of times a SD PWID injected drugs in TJ with respect to Price (= β1*exp (β0+β1 (Price) + C1’ (Time))). This product will be calculated for various levels of Time by substituting Price with its expected value obtained by using the prediction equation between Time and Price. Significance of indirect effects for each level of Time will be tested by bootstrapping. Potential covariates will also be assessed and if needed will be included in the prediction equations by being set to appropriate levels (e.g., mean).^39^  H1.2 posits that PWID who inject in TJ will be more likely to report a) injecting powder formulations (i.e. heroin & fentanyl) and b) prescription drugs compared to NDTs from SD. Based on estimates from 3D.ii, we will use a Logistic Regression Mixed Model with injecting powder drug formulations/injecting prescription drugs (yes vs no) as the outcome and injected in TJ past 6 mo (yes vs no), time, and the interaction between the two as main fixed effects. The interaction will only be tested to ensure the integrity of the model; however, if it is significant the simple main effects (i.e., effect of time stratified by injection location and/or effect of injection location stratified by time) will be calculated from the full model and corresponding significance tests will be conducted.  Aim 2. H2.1 posits that time will moderate associations between location (TJ vs. SD) and the outcome (PWID self-reporting use of fentanyl ). Specifically, we hypothesize that at baseline we will see a significantly higher % of PWID in TJ reporting fentanyl use compared to SD, but this difference will diminish over time. We will use a logistic regression mixed model, with fentanyl use as the outcome (positive vs. negative), location, time, and location*time interaction as fixed effects, and subject as a random effect. We expect the interaction to be significant, in which case, we will assess the simple main effects of time on the outcome stratified by location, as well as the simple main effects of location on the outcome, stratified by time point.  H2.2 posits that time will moderate the association between location and the a) availability and b) price of heroin, fentanyl, and meth, respectively. That is, at baseline we will see a significant difference between the two locations with regards to the availability and price, but over time this will diminish with respect to availability and possibly price. We will use Logistic Regression Mixed Models with a) availability (increased vs. decreased/stayed the same) during the previous 6 mo; and b) price (decreased vs. increased/stayed the same) during the previous 6 mo reported at each time point as the outcome, and the same fixed and random effects and time*location interaction assessed as in H2.1.  **Sub-Study #1 Data Analysis**:  **Aim 1. To determine the prevalence and correlates of asymptomatic and symptomatic SARSCoV-2 infection among PWID within the San Diego/Tijuana border region.**  **Statistical Power:**  To test H1.1, power calculations were done using PASS, based on 2-sided tests with α=0.05, adjusted for covariates to yield R^2^=0.05, based on a logistic regression with *symptomatic SARSCoV-2 infection (y/n)* as the outcome and *smoking tobacco, marijuana, methamphetamine or crack at least once per month in the past 6 months (Group1: smoker vs. Group 2: non-smoker)* as the predictor variable. We used N=600, distributed 80% in Group 1 and 20% in Group 2 based on estimates from the ECIV and STAHR studies). If the proportion of SARSCoV-2 infection in Group 2 is 20%, 40%, or 60%, we have ≥80% power to detect a significant difference if the corresponding proportion of SARSCoV-2 infection in Group 1 is ≥33% (OR_Group1/Group2_=1.98), 55% (OR_Group1/Group2_=1.80), or 74% (OR_Group1/Group2_=1.86), respectively. For instance, if the % with asymptomatic SARSCoV-2 infection in Group 2 is 40%, we have ≥80% power to detect a significant difference between the two groups if the corresponding proportion in Group 1 is ≥55% (corresponding OR=1.80) which is the smallest effect size we can detect.  **Aim 1.** Data Generated: Survey data at baseline and supplemental survey data will be merged along with SARSCoV2 testing data.  Statistical Analysis: First, we will calculate descriptive statistics (i.e., frequencies and percentages) and prevalence estimates of symptomatic and asymptomatic SARSCoV-2 along with 95%CIs overall as well as stratified by site and gender. For hypotheses testing, our primary analytic approach will be using GLMM, drawing from data from the baseline and 1 week study visits. A random intercept for subjects will be used to account for the variability in visit dates. In all analyses, study site will be used as a covariate. Age, gender, homelessness status and a history of pre-existing conditions are other potential covariates. To ensure integrity of the models, all possible interactions between independent variables included in the models will be assessed and either ruled out (if not significant) or, if significant, evaluated by calculating simple main effects stratified by various levels of the variables involved in interactions. Additionally, all multivariable models will be tested for multi-collinearity by using diagnostics such as largest condition index and variance inflation factors.  To evaluate H1.1, H1.2and H1.3, we will be using Logistic Regression Mixed Effects Models with *subclinical SARSCoV-2 infection* (H1.1) and *prevalent SARSCoV-2 infection,* respectively, as the outcome. *Smoking frequency in the past 6 months (at least once per month vs. less often/none)* (H1.1), *type of drug used past 6 months (fentanyl vs. heroin)* (H1.2) and *frequency of vaping cannabis or e-cigarettes (at least once per week vs. less often/none)* (H1.3), respectively, will be the primary main predictor. We will also conduct exploratory analyses to determine if sharing of paraphernalia used to snort drugs (e.g., straws, dollar bills) is associated with testing SARSCoV2 RNA+.  **Aim 2: To determine the prevalence and correlates of SARSCoV-2 shedding in nasal and fecal specimens among PWID.**  Data Generated. Participants will be divided into those who have tested SARSCoV-2 RNA+ vs. RNA-negative. Of these, we will randomly select 200 RNA+ and 200 RNA-negative participants who will serve as controls. To quantify *S. pneumoniae* abundance (H2.1), DNA will be isolated from nasal swabs and applied to quantitative PCR for primers specific to this species; primer selection will also be improved using data on genome-assemblies of specific strains from metagenomic analysis. To quantify the proportions of different bacterial species, present in fecal (H2.2) material, microbial DNA will be extracted using standard protocols. DNA will be used to construct metagenomic libraries from 1 ng using the NexteraXT kit, which will be barcoded, and multiplex sequenced to generate ~5 Billion bp per sample on the NovaSeq platform. These data will be used to identify significant associations between the proportion of bacterial species and those testing SARSCoV-2 RNA+.  **Statistical Analysis**: To test H2.1 and H2.2 we will use Logistic Regression Mixed Effects Models with *SARSCoV-2 RNA (+ vs. -)* shedding in nasal (H2.1) and fecal specimens (H2.2), respectively, as the outcome and the quantity of *Streptococcus pneumoniae* (#cells/volume) (H2.1) and number of specific bacterial species (H2.2), respectively, as the main predictor. To determine microbial species (genotypes) present in each fecal sample, Novaseq reads will be assembled de novo using IDBA-UD, annotated using RAST, and binned by taxon using tetranucleotide frequency, cross samples coverage, and %GC. Genome bins will be further assembled and curated to produce draft assemblies of microbial genomes (strain-level genotypes). These microbial genotypes will be associated with the presence of SARSCoV-2 RNA by regression analysis and generalized linear modeling.  **Expected Results:** Using regression analysis and GLM, we will determine associations between SARSCoV-2 and specific microbes present in fecal samples, and with *S. pneumoniae* in nasal secretions. These data will suggest possible mechanisms by which SARSCoV-2 selectively alters infectivity and pathogenicity in specific patients. It is possible that we may not detect a strong association with SARSCoV-2 and *S. pneumoniae* in nasal secretions, as the relative abundance of specific microbes in the nasal passages of SARSCoV-2 patients is currently unknown. If this is the case, following completion of analysis of fecal samples, we will rescreen the nasal swab specimens for the top pathogenic microbes that we find associated with SARSCoV-2 in feces and conduct analyses accordingly.  **Aim 3: To determine network features associated with cross border transmission of asymptomatic SARSCoV-2 infection among PWID.**  **Data Generated**: Survey data at baseline and from the supplemental social network survey will be merged along with SARSCoV2 testing data.  **Statistical Analyses**: All hypotheses will be evaluated based on N=600 using data from the baseline and 2 week study visits. Similar to Aim 1, our primary analytic approach will be using GLMM. To evaluate H3.1-H3.3, we will be using Logistic Regression Mixed Effects Models with *asymptomatic SARSCoV-2 infection* (H3.1 and H3.2) and prevalent (H3.2), respectively, as the outcome. *The number of people who vape in the social network (H3.1), the number of DTs in the social network (H3.2), and having attended TJ events of high intensity past 6 months (yes/no)* respectively, will be the main predictors.  **AIM 4: To use molecular epidemiology to determine the global migration of SARSCoV-2 in and out of SD and TJ and factors associated with local and global dispersal.**  **Data Generated:** For each participant who tests SARSCoV-2 RNA+, we will isolate, reverse transcribe and PCR amplify using 30 sets of overlapping primers^40^. After amplification and purification, we will sequence each amplicon using Illumina MiSeq sequencing platform^41^. Each amplicon-sequence will be combined through alignment of overlapping reads to obtain near full-length SARS-SARSCoV-2 genome. This procedure and its application has been described for coronaviruses^42^ and the GISAID^43^ has already gathered over 700 genomes sampled between Dec 2019 and March 2020. While CoV-2 does not evolve as rapidly as HIV, it does show genetic differences, mostly non-synonymous, in close local spread that can be used to evaluate migration.  Analysis: First, we will combine sequence data with publicly available sequences data available on the NCBI and GISAID platforms with known date and sampling location[34, 35]. For example, as of March 28, 2020, >2,000 high quality FL genomes from >50 countries were available on GISAID including 564 from the US across 11 States (34 from California). Next, using these informed datasets, we will develop Bayesian phylogeographic models using the asymmetric discrete trait diffusion model and a generalized time reversible + gamma (GTR+Γ) substitution model[36-39] implemented in the BEAST 1.10.5 software[40]. Estimates of the posterior probability of expected number of migration events between all pairs of locations (Markov jumps) will be computed through stochastic mapping techniques[41, 42]. We will specify a normal distribution as prior on the mean clock rate with mean 0.0015 s/s/y and standard deviation such that the 95% credible interval (CI) ranges from 0.0013 s/s/y to 0.0018 s/s/y based on our preliminary analyses (unpublished). Of note, given high sequence homology, our group and others have shown that timing of all events should be considered based on their intervals and not point values[43]. Our group has also recently validated a new measure of significance that has a lower false-positive rate by incorporating information on the relative abundances of samples from each location in the data set, which was coined as the adjusted Bayes factor (BF_adj_)[44]. This will allow us to take into account the sampling heterogeneity in our inferences. Simultaneously, with reconstructing the geographical migration history, the spread of CoV-2 dispersal across social network groups (i.e. high vs low contact rate) within SD will also be estimated. Finally, we will use a GLM extension of the discrete trait model implemented in BEAST 1.10.5[45] to investigate the potential contribution of epidemiological variables to the dispersal rates across locations (i.e. SARSCoV-2 incidence, population density, human migration, air traffic, etc).Using these models, we will explore the chain of dispersal within the SD/TJ border region and between TJ, SD and the rest of the US. We will identify the timing (with uncertainties mentioned above) and origin of viral introduction within the SD/TJ area. Given the expected density of the social network of new COVID-19 cases, we hypothesize that we will identify a limited number of viral introduction events within the SD/TJ border area. Using the GLM implemented into the discrete diffusion models, we also expect to identify epidemiological factors associated with local dynamics (i.e. number of contacts in social networks) and global dynamics (i.e. incidence in sampled location, population density and human mobility).  **Sub-Study #2 (Barriers to COVID19 testing and Vaccination):** *(Additional Measures)*  Based on in-depth interviews in 04/21, we will add items to assess misinformation and conspiracy theories about COVID-19 (e.g., “it was created by the Chinese government”) and vaccines [22, 46] (e.g., they “include a tracking device”, or “alter human DNA”). We will also ask if mandated reporting of COVID+ tests discourages PWID from seeking testing, which may particularly concern those who are undocumented.  We will obtain information on COVID testing and vaccination in three ways: 1) *Self-report from La Frontera follow-up interviews*. 2) *Record linkage with the SDHHS COVID-19 database* based on electronic health records (EHRs) for participants who provide a release of medical information.. Record linkage will confirm if and where COVID testing was done elsewhere, if COVID-19 vaccine was administered, type, dates and # of doses, as well as hospitalizations and deaths. 3) *Biological validation* of vaccination will also be done based on COVID-19 antibody testing of La Frontera participants through Genalyte providing independent validation that does not rely on self-report. Presence of antibodies to SARS-CoV2 “S” protein is indicative of vaccination, as opposed to natural infection.  **Sub Study # 2 Data Analysis Plan**  For each outcome, cross-tabulations will be generated using proportions for categorical variables and means, medians and inter-quartile ranges for continuous variables. For hypotheses testing, our primary approach will be generalized linear mixed models with random slopes and intercepts for each subject to account for unmeasured differences between subjects, variable timing of visits, and correlations induced by multiple measurements per subject. For hypotheses involving incidence, we will use Cox regression with time dependent predictors considering potential fixed covariates (e.g., gender, ethnicity/race, age, education). We will also disaggregate and analyze results by sex/gender; race/ethnicity; age, income, country of birth, sexual orientation and consider intersectionality where power allows.  **H1.1** posits that PWID who are more likely to endorse misinformation and conspiracy theories will be less likely to a) seek COVID-19 tests and b) wear facemasks. Endorsing misinformation and believing in conspiracy theories will be assessed based on responses to a number of related statements from the literature [22, 46] and new measures based on our preliminary data. These will be scored on a 4-point scale from “Definitely not true” to “Definitely true”. Binary variables indicating belief in a particular “conspiracy theory” (True/Definitely True vs. False/Definitely False) will be constructed for each statement. Next, the relationship between each binary variable and outcome will be assessed. The distribution as well as the internal consistency of items will be evaluated and, depending on the results, an index score obtained either by averaging the responses to the original statements or by summing up the “True/Definitely True” responses, will be created and used as the main predictor. We will use Logistic Regression Mixed Models with a) *sought COVID-19 testing past 6 months (yes vs. no)* and b) *reported wearing a mask in public past 6 months (yes vs. no)* as the outcome and *endorsement of conspiracy theories index, time (baseline vs. visit 2)*, and *interaction between the two* as main fixed effects. *COVID-19 vaccination status* and *known or perceived exposure to COVID-19 in the past 6 months* will also be included in the model as covariates, along with aforementioned covariates. All interactions among covariates and between covariates and main effects will be tested. If any are found to be significant but not confounding, the corresponding simple main effects will be evaluated from the full model. In case of significant and confounding interactions (i.e., interactions driven by the direction of the main effect) stratified analyses will be undertaken. However, we do not hypothesize nor expect confounding interactions.  **H1.2** will be evaluated as above except that knowing someone who died of COVID will be the main predictor.  **H2.1** posits that the incidence of vaccine uptake (e.g., determined by longitudinal serology that reflects S antibodies) will be lower among those who a) lack transportation b) experience homelessness, c) have low health literacy, d) lack health insurance or e) are undocumented vs. their counterparts. We will use Cox regression with *time to vaccination as the outcome*, and variables of interest as binary time varying predictors. Time at risk will be length of time from baseline to the 1st vaccine. Those not vaccinated during follow-up will be right censored. Time dependent predictors will indicate whether one was part of the exposure risk set prior to the event (e.g., lacked transportation prior to vaccine uptake or prior to the end of the follow-up period, whichever comes first). Those found to have been vaccinated will be excluded from incidence analyses.  **H2.2** posits that PWID with evidence of SARSCoV-2 infection (based on longitudinal serology) will have greater uptake of COVID-19 vaccination compared to uninfected PWID. H2.2 will be evaluated in a similar manner as H2.1, except that *evidence of SARSCoV-2 infection (yes vs. no)* will be the primary predictor.  **Aim 4.** To compare COVID testing and vaccination rates before vs. after the CDPH introduces these services at OnPoint, for the period before introduction of testing at OnPoint, we will use La Frontera survey data from 3/1/21-8/31/21 (when rapid COVID19 testing will begin at OnPoint). For the period after the introduction of testing at OnPoint, we will use data collected between 9/1/21-3/1/22.  **H4.1** posits that the rate of a) tests and b) vaccination will be significantly higher after co-locating these services at OnPoint, vs. corresponding rates during preceding periods. We will conduct segmented regression analysis to estimate month-to-month changes in COVID-19 tests within each period and changes between the two periods. Survey data will be divided into 12 one-month segments, with six corresponding to pre- and six corresponding to post-OnPoint testing implementation. Then, a mixed effects logistic regression model with a random intercept and an unstructured covariance matrix (to control for within subject correlations) will be used to estimate participants’ probability of getting tested during each of the 12 months in question. Next, the probabilities from the aforementioned model will be aggregated to determine the overall monthly testing rate. Last, aggregated estimates will be used in the segmented regression to estimate month-to-month changes in COVID-19 testing within each period, and changes between the two periods. These models will be tested for autocorrelation by using the Durbin-Watson test,[49] and if detected, auto-regressive terms will be added.  **Aim 5**. Among vaccinated PWID, we will monitor post-vaccination behaviors and breakthrough SARS-CoV-2 infections..  **H5.1** posits that those who get vaccinated will take more trips outside of SD County as opposed to those who have not been vaccinated. We will use a negative binomial mixed regression with # of trips outside SD County by the end of the study as the outcome. Vaccination status (complete, partial, none), # days elapsed since their first shot (with 0 being used for those who were not vaccinated) and the interaction between the two will be main fixed effects. A random effect for subject will be included in the model. Group, gender, age, income, homelessness and whether they have immediate family living outside of SD County or in Mexico will be considered as potential covariates or effect modifiers.  **3.6.13. Power:** Power was estimated using PASS,^®^ 2-sided tests with α=0.05 adjusted for covariates to yield R^2^=0.06, conservatively assuming that at least 70% complete La Frontera visit 2 (N=280). Since calculations were based on single time points, longitudinal methods will increase power. Incidence was based on N=230, assuming that by Fall/2021,18% may have already been vaccinated (excluded from incidence calculations).  **H1.1a** posits that PWID who endorse misinformation or conspiracy theories will be less likely to have COVID-19 tests. Power was based on a logistic regression with *COVID-19 test in the past 6 months (y/n)* as the outcome and *endorses conspiracy theories (Group 1=yes vs. Group 2=no)* as the predictor, with N=280 and 30% distributed in Group 1 and 70% in Group 2. If the % that had a COVID-19 test in Group 2 is 25%, 30% or 35% (Fig. 2), we have ≥80% power to detect significant differences between the two groups if the corresponding proportion in Group 1 is 10% (OR=0.34), 14% (OR=0.38), or 18% (OR=0.41), respectively. If the % endorsing conspiracy theories in Group 2 is 25%, we have ≥80% power to detect a significant difference between the two groups if the corresponding proportion in Group 1 is 10% (corresponding OR Group 1/Group 2=0.34). Estimates were based on a recent study which found that belief in conspiracy theories among Whites was 10-26% and 29-34% among Latinx [22] and data from La Frontera (32% of SD PWIDs reporting COVID-19 testing).  **H1.3** posits that SSP attenders will be more likely to report COVID-19 tests. Power was based on a logistic regression with *COVID-19 test in the past 6 months (y/n)* as the outcome and *attended SSP in the past 6 months (Group 1=yes vs. Group 2=no)* as the predictor, with N=280 and 40% distributed in Group 1 and 60% in Group 2. If the % who had COVID-19 tests in Group 2 is 10%, 15% or 20%, we have ≥80% power to detect a significant difference between the two groups if the corresponding proportion in Group 1 is 23% (OR=2.7), 30% (OR=2.4), or 36% (OR=2.2), respectively. Power was based on La Frontera where 43% of SD PWID attended SSPs in the past 6 months, of whom 53% had a COVID-19 test vs. 15% of non-attenders.  **H2.1b** posits that COVID-19 vaccination incidence rate (IR) will be lower among homeless PWID vs. those who are not. Power was based on a 2-sided Cox regression considering homelessness as a binary predictor. Assuming that the 6-month vaccination IR Group 2 is 10%, 20% or 30%, a Cox regression of the ln (IRR) on the group variable with sd=7.4 will have ≥80% power to detect a ln IRR= -0.083, -0.051, and -0.069, respectively. Thus the smallest corresponding IRR (Group 1/Group 2) we can detect with ≥80% power would be 0.92, 0.95, and 0.93, respectively (i.e., ~1-2% difference). Power was based on La Frontera estimates, where 0% of homeless PWID were vaccinated vs. 6.4% of PWID who were not homeless. |
| **10. HUMAN SUBJECTS** |
| **Characteristics:**  We will recruit 3 groups of 200 PWID (N=600 total): 200 PWID who injected drugs in TJ ≤6 months ago but live in SD (DTs) will be recruited and interviewed in TJ to ensure that they are truly border-crossers. Non-DTs (NDTs) are PWID who live in SD county but have never injected illicit drugs in Mexico and will be interviewed in SD (N=200) and PWID who live in TJ who have never injected across the border (N=200). The characteristics of our proposed study population are by design injection drug users (PWIDs) 18 years or older. By the nature of our studies sites, ethnic background are expected to be Hispanic/Latino. We will make every effort to recruit an equal number of female and male subjects, although past study surveys have indicated that males form the vast majority (about 94%) of injecting drug users in Tijuana and San Diego. In addition, participants must be able to speak English or Spanish. Participants must be people who injected within the last month because active injectors are more sensitive to drug market changes and have higher risk of HIV, HCV and OD, optimizing statistical power. Injection drug use will be verified by inspecting track marks.  *Inclusion Criteria:*   1. *Be > 18years old;* 2. *People who injected drugs within the last month;* 3. *Proof of injection via track marks on arms, legs, other body parts;* 4. *Group A: Have injected in Mexico within the past year; live in SD at least part-time and intend to live in the SD/TJ border region for 2 more years* 5. *Group B: People who inject drugs who live in TJ but have not been in the US in the past two years and intend to live in Tijuana for 2 more years;* 6. *Group C: PWID who live in SD but have not been to Mexico in the past 2 years and intend to live in SD County for 2 more years.*   *Exclusion Criteria:*   1. *Be < 18 years old;* 2. *People who do not inject drugs;* 3. *People who injected drugs longer than one month ago;* 4. *No evidence of track marks on arms, legs or other body parts;* 5. *Group A: Have injected in Tijuana longer than the past year but live in SD; Have injected in Tijuana > longer than the past year and do not live in SD even part time; intend to live in the SD/TJ border region for less than 2 years;* 6. *Group B: PWIDs who live in Tijuana and have been to the US less than 2 years ago; PWIDs who do not live in Tijuana and have never used illicit drugs in SD; intend to live in Tijuana for less than 2 years* 7. *Group C: PWIDs who live in SD but have used illegal drugs in Tijuana within the past year; PWIDs who do not live in SD and have used illegal drugs in Tijuana; PWIDs who do not live in SD and have never used illegal drugs in Tijuana. PWIDs who intend to live in SD for less than 2 years.*   **Ethnographic Component:**  We will sample 75 PWID from each of the 3 groups enrolled in La Frontera, (25 DT from SD, 25 NDTs from SD, and 25 NDTs in TJ) aiming for 30% females and 75% fentanyl injectors. In addition to the 75 PWID, we will look for background social structural contextualization and so will interview kin, peers and service providers because of their knowledge about PWID in Tijuana/San Diego. Approximately 20% to 30% of our sample will consist of these participants at any given stage of the research. We anticipate that by the end of the five years we will have consented approximately 75 to 100 of these peripheral participants.  Inclusion Criteria:   1. Be > 18years old; 2. Any gender; 3. Any ethnic backgrounds (likely Hispanic/Latino) 4. Health status should not affect inclusion 5. Has a relation to drug markets (kin, peer, service provider, etc.) 6. Must reside in San Diego or Tijuana   Exclusion Criteria:   1. Be < 18 years old 2. Has no relation to drug marketsDoes not reside in San Diego or Tijuana |
| **11. RECRUITMENT AND PROCEDURES PREPARATORY TO RESEARCH** |
| *Recruitment will take place in areas in which injection drug users hang out in Tijuana and San Diego. Based on our experience, injection drug use venues include bars, hotels, street corners, riverbanks, canals, canyons and shooting galleries. PWID will be approached in the field by a trained outreach worker who will ascertain the participant‘s willingness to participate in the study through a number of screening questions, asked in a conversational manner prior request of verbal consent to ask the screening questions. Our outreach workers are very familiar with these venues, as they have worked in these communities for several years and some are former injection drug users. PWID who are potentially appropriate for inclusion and willing to complete the eligibility process will be referred to the storefront office, where they will be screened and receive a detailed description of the study through the informed consent procedure.*  Potential recruits will complete a pre-interview screener, which will help us determine eligibility, language proficiency, and willingness to participate in the intervention, in addition to minimizing respondent burden. Questions include age, date of last injection, city of residence, history of border crossing, foreign cities where they have injected drugs and date last injected there. We will add ‘red herring’ questions to prevent people from guessing eligibility criteria. While not part of the eligibility criteria, we will inquire about the relationship between recruiters and their recruits, their network size and its composition (e.g., % female, % from US and TJ, ethnicity). Pre-interview screenings will typically occur in our storefront offices or outreach vans in locations where PWIDs frequent. Screened PWID will each receive $5 USD regardless of whether or not they are enrolled.  *The research staff who will be responsible for obtaining voluntary informed consent will assess whether the potential participant has understood the study and consent form by asking key questions (e.g., “How much time will this take you?”; “What are the possible benefits for you?”). Because it is possible that some of our participants may be cognitively impaired, or they may not initially understand the consent process, we will test all potential participants for their comprehension of critical points in the consent form. Errors will be corrected and these potential participants will then be asked if they need further clarification. If they are confused due to inebriation or suffering from withdrawal symptoms, they will be rescheduled for the next day. If, after further attempts to clarify any misunderstandings, we determine that they may not fully comprehend the critical aspects of the study, they will not be enrolled. If a potential participant decides she or he does not wish to participate, his or her decision will be honored regardless of how well they comprehend the study information.* |
| **12. INFORMED CONSENT** |
| During the screener, initial verbal informed consent will be done carefully out of earshot from eavesdroppers (often when possible in greater privacy and safety of parked vehicle, or in most neutral and safe public space available).  We previously obtained a waiver of documented written consent for the screener portion of the study. The only record linking the subject and the research would be the consent document and the principal risk would be potential harm resulting from a breach of confidentiality. Each subject will be asked whether the subject wants documentation linking the subject with the research and the subject’s will to govern.  Within a 48-hour period, PWIDs deemed eligible based on the screener will be invited back to the study clinic where they will undergo informed consent procedures, which involves providing written consent. PWID who are too high to provide informed consent will be rescheduled. Recruitment of subjects and informed consent procedures (both for main study and ethnographic component) follow procedures used in our U.S. based studies. Fully informed consent will be obtained before any data are gathered. Written consent will be obtained for the quantitative component of the study including the SARS-CoV-2 substudy. The consent form was written in simple language at a level that can be understood by the potential participants. Details of study participation, including the need for follow-up contact information, will be described in the consent form and explained verbally. Consent forms will be read aloud by the study staff and the potential participant can follow the reading with his/her copy. If potential participants are not able to read, they can listen to the text being read by the study staff and ask all the necessary questions. To assess the level of comprehension of the potential participant we will implement a “Decisional Capacity Assessment” tool and ask participants to mention in their own words two potential risks of participating in the study and to explain what they need to do if they want to withdraw participation from the study.  The research staff who will be responsible for obtaining informed consent will assess whether the potential participant has understood the study and consent form by asking key questions (e.g., “How much time will this take you?”; “What are the possible benefits for you?”). Because it is possible that some of our participants may be cognitively impaired, or they may not initially understand the consent process, we will test all potential participants for their comprehension of critical points in the consent form. Errors will be corrected and these potential participants will then be asked if they need further clarification. If, after further attempts to clarify any misunderstandings, we determine that they may not fully comprehend the critical aspects of the study, they will not be enrolled. The field coordinator will also be available to answer any questions raised by a given participant. If a potential participant decides she or he does not wish to participate, his or her decision will be honored regardless of how well they comprehend the study information. A copy of the consent form without any identifiers will be offered to all participants. The consent form includes a description of the study, the telephone numbers of the PIs in Mexico and the U.S. where participants can call or leave a message 24 hours a day if they have any questions or concerns.  **Ethnographic Component:**  All La Frontera study participants will be considered for inclusion in the ethnographic component as described in detailed in the informed consent form. For this reason we have decided to combine both original informed consents into one. We will ask study participants already recruited (n=31) to read and if in agreement, sign the revised informed consent. Ethnographers will initiate contact with recruited PWID for involvement in the ethnographic component through friendly conversation in public venues or through screened referral from main study interviewers who staff the follow-up quarterly locator check-ins. If the study participant is interested and willing, the ethnographer will then reiterate the description of the ethnographic component, its objectives, the possible risks and expected benefits that were addressed in the initial informed consent process..  For non-PWID participants recruited because they are knowledgeable of drug users and service, and who do not possess drug paraphernalia), we will request for a waiver of documented consent. This portion of the study is minimal risk (the main one being loss of confidentiality). Non-PWID are not subject to the requirements of the six-month follow-up interview over the course of the project. Additionally, for qualitative data, the benefits to protecting confidentiality provided through a signed consent form are outweighed by the risks to confidentiality that a signature on a hard copy paper form represents. The hard copy signature becomes the most direct significant identifying marker. Furthermore, among street-based populations with substance use disorders, especially in Mexico, requesting a signature can be interpreted as a sign of distrust. We have also found that the verbal process of consent can be more meaningful and comprehensible to homeless participants in street settings (some of whom have limited or no literacy skills) when the consent is conducted in a conversational oral mode rather than through reading and signing a written form.  For the ethnographic component we would like to know about participants’ lives, their needs and the life of their friends and companions beyond what is asked about in a formal questionnaire. This would involve possibly meeting them more frequently on a semi-regular basis so that ethnographers could learn more about participants’ lives by having longer conversations with them. Perhaps on occasion, when convenient for study participants, one or more members of the research team might be able to accompany them in their daily activities, going with them to meet their friends and associates, health clinics, and/or possibly meet members of their family or people they are currently living with now, or just go visit where they sleep and spend most of their time on any given day. Ethnographers may be interested in meeting friends or employers study participants have worked with in the past or where they are currently working. They would be interested in knowing how study participants obtain food and money and drugs to survive, or more simply they might learn interesting things by just walking around the city with study participants to places they think ethnographers should know about to learn more about the reality of people in Tijuana who use drugs. On those days or interview sessions, the ethnographers would be interested in possibly recording some of their conversations with study participants or maybe conduct a more formal life history with them.  Study participants who participate in the ethnographic component will receive $40 for their involvement. Study participants will also be informed that participation in the ethnographic component is voluntary and their decision to be part in it or not in no way affects their involvement in the other components of the study.  All members of the ethnographic and quantitative research team will have CITI Biomedical Research Ethics training and HIPAA certification training.  For the named individuals in the network survey incorporated in the sub-study questionnaire, we requested and obtained a waiver of consent to obtain name and locations (geospatial locations such as home, shooting gallery, party, sex work venue) for research purposes. The following conditions still apply:  1. The research is minimal risk. We will protect the participants’ confidentiality and protect their PII.  2. The waiver will not adversely affect the rights and welfare of the subjects. Obtaining the names and locations will be protected.  3. The research could not practicably be carried out without the waiver. In order to construct the network, we need to obtain name and location. |
| **13. ALTERNATIVES TO STUDY PARTICIPATION** |
| The subject population has traditionally been medically underserved. Counseling services and monitoring and treatment for HIV and HCV are provided by the municipal health clinic in Tijuana free of charge, regardless of their health insurance status. |
| **14. POTENTIAL RISKS** |
| The major potential risk to subjects is a confidentiality breach about their SARSCoV-2 status, their risky behaviors, institutional relationships or health. This could cause them psychological distress, discrimination, and possible victimization. It could also include violation of privacy, or possible discomfort/distress when recounting unpleasant past experiences, which may include physical, emotional or sexual trauma, victimization, or arrest. Further mentioned in item 15 are the procedures in place to ensure there is no access to private data beyond the research team.  Other potential risks identified include:  **Physical Risk:**  When drawing blood via venipuncture could cause hematoma at the site of the needle prick; we will make every effort to hire qualified staff who have experience drawing blood on people who have hard draws. To date, we have not had any incidents of hematomas during blood drawing procedures.  **Disclosure of HIV test results:**  Participants testing positive for HIV/HCV may experience distress when learning about their health condition and its moral, psychological, social and ethical implications. US and Mexican Law upholds the right to privacy and confidentiality of HIV/HCV study participants. All project participants with a HIV positive result will be referred for follow up at San Diego Public Health Clinics, Community Clinics in San Diego and in Tijuana at CAPASITS Tijuana, the local specialty clinic set up to provide free access to care and treatment for HIV and other STIs in Tijuana. Being tested for HIV can make a person feel nervous or anxious about the test results. A positive test indicates that the person is infected with HIV, but no one knows for certain when, if ever, one will get AIDS or a related condition. Receiving positive results may make a person very upset. If other people learn about someone’s positive test result, there might be a risk that one could be treated unfairly or badly, and even have trouble obtaining employment. To the extent permitted by law, the researchers will keep a person’s test results confidential and will not release them to anyone without the person’s written permission. If a person test positive, California law requires health care providers and clinical laboratories to report the HIV test results with the person’s personal identifying information to the local health department.  **Psychological Risks:**  Some of the survey questions related to drug use, cross-border activities, transporting drugs, mental state, stress level and living conditions may be stressful and psychologically difficult for subjects. We will make every attempt to provide these participants with immediate counseling to assist with any medical and/or psychological concerns in the consenting process and throughout the data collection process. They can refuse to answer any questions if they are uncomfortable. Our study staff are highly experienced counselors who have worked in the HIV/AIDS and mental health fields for many years. We are therefore confident that we will be able to resolve immediate crises experienced by any of these participants.  **Legal Risk:**  There is also legal risk if information on illegal behavior (i.e., drug use, drug dealing activities) were to become public knowledge. For this reason our study team members will inform the police departments in San Diego and Tijuana of the study. We will also provide each participant with a study card and 1-800 number in case they are detained by police, so that our staff can advocate on their behalf if needed. The data will also be protected in the United States by a Federal Certificate of Confidentiality which can be used in US courts to refuse to disclose information that may identify you in any US federal, state, or local civil, criminal, administrative, legislative, or other proceedings. A court subpoena can override the federal certificate of confidentiality in exceptional circumstances primarily related to organized crime and capital crime cases, but historically it has proved effective for routine misdemeanor and felony charges.  **Disclosure of SARSCoV-2 test results:**  Participants testing positive for SARSCoV-2 may experience distress when learning about their health condition and its moral, psychological, social and ethical implications. US and Mexican Law upholds the right to privacy and confidentiality of all study participants. We will make sure that all study participants fully understand the difference between a SARSCoV-2 Ab and PCR positive test result. A positive SARSCoV-2 Ab test result means that the person has been exposed but a SARSC0V-2 PCR test is needed to rule out current active infection. All project participants with a confirmatory SARSCoV-2 PCR positive test result will be referred for follow up at San Diego Public Health Clinics, Community Clinics or assigned hospitals in San Diego and in Tijuana Hospital General de Tijuana or IMSS 20 regional Hospital, the two designated hospitals set up to provide free access to care and treatment for SARSCoV-2 in Tijuana. Being tested for SARSCoV-2 can make a person feel nervous or anxious about the test results. A positive PCR test indicates that the person is infected with SARSCoV-2, but no one knows for certain when or where or whether the person will develop a symptomatic or asymptomatic, mild or serious infection. Receiving positive results may make a person very upset. If other people learn about someone’s positive test result, there might be a risk that one could be treated unfairly or badly, and even have trouble obtaining employment or lodging. To the extent permitted by law, the researchers will keep a person’s test results confidential and will not release them to anyone without the person’s written permission. If a person tests positive, California and Mexican law require health care providers and clinical laboratories to report the SARSCoV-2 test results with the person’s personal identifying information to the local epidemiology department.  **Risk of SARSCoV-2 community transmission on study premises:**  There is a probability that community transmission could take place on the study premises while screening study participants. We will make every attempt to implement control measures: potential participants will be given a face protection device upon entering the premises to be worn at all times while on the study premises and will be requested to wash their hands with soap and water or with hand sanitizer; next, participants will be screened in the open air in the area designated for this purpose with plenty of air flow; only two participants will be seen at a given time by study staff: one completing the symptom screening questionnaire while the second one is undergoing biological sample collection and testing; both stations will be placed at least 15 feet apart to guarantee social distancing at all times. Study staff will wear face protection, N95 masks, nitrile gloves at all times and any other protective equipment deemed necessary. We will also setup clear, plastic screens to provide a physical barrier between study participant and study staff while completing the supplemental survey. Upon completion of all study measures, study participants will be given an extra facial protection and useful information about SARSCoV-2 preventive measures and led to the facility’s exit area. We have developed a detailed protocol based on CDC Guidelines for SARSCoV-2 screening in community settings (See Appendix 1). |
| **15. RISK MANAGEMENT PROCEDURES AND ADEQUACY OF RESOURCES** |
| **Confidentiality of participation:**  Every possible procedure to prevent breach of confidentiality will be taken to ensure there is no access to private data beyond the research team. Audio interviews will be collected carefully out of earshot from eavesdroppers (often when possible in the greater privacy and safety of the ethnographer's parked vehicle or in the most neutral and safe public space available). Fieldnotes and audio data will be securely stored in encrypted machines in locked offices. Strathdee, Bourgois and his ethnographers in over two decades have never experienced a negative incident with respect to loss of privacy, confidentiality, victimization or psychological distress. Once NIH funding has been obtained, ***we will have a*** Federal Certificate of Confidentiality that will allow us to protect participant information from outside requests and subpoenas in the U.S. To date, these certificates have also been respected in Mexico.  Blood specimens will also be obtained by venipuncture and will be stored on site at the study clinic before it is shipped on a weekly basis to the UCSD CFAR lab. Following specimen collection, trained interviewers who have extensive experience working with PWIDs will administer the questionnaires. Instruments will be piloted in order to ensure that questions are culturally sensitive to the situational factors faced by PWIDs in Tijuana and San Diego and that the interview will not exceed one hour in order to reduce respondent burden.  All study staff will be trained not to press participants to answer questions that seem to be distressing to them, and interviews will be terminated if the participant is overly distressed, fatigued, or frustrated by the effort. In the event that clinically significant depression symptoms, suicidality, or other psychiatric conditions are noted, the staff will be instructed to report any incidents or concerns to the field coordinator who will arrange for appropriate referral to the on-site psychologist. In addition, the PI, all co-investigators and all field study staff in San Diego and Tijuana will have completed Human Subjects Training and CITI Certification Program before data collection begins.  **Participant Withdrawal from Study:**  At the time of screening, ethnographers will carefully minimize calling undue attention to participants and clearly advising them that they may ask the ethnographers to leave/discontinue the conversation/interview at any time. Participants' wishes and concerns will always be immediately respected. Similarly, participants are reminded they have an option not to participate and that there are no consequences for not participating.  **Communication with Local Authorities**  A signed letter from the Secretaria de Salud in Tijuana will be sent to local police authorities in Tijuana outlining the study objectives and duration. A similar letter signed by the principal investigator will be sent to San Diego police department. Heightening awareness and ensuring support from local police authorities will not only increase safety for participants and study personnel, but will also reassure study participants that their involvement in the study will in no way put them in any danger or lead to harassment by the police.  **SARSCoV-2 screening and sample collection:**  Nasal swabs and stool samples will also be obtained by qualified staff and will be stored on site at the study clinic before it is shipped on a weekly basis to the public health lab in San Diego. Following specimen collection, trained interviewers who have extensive experience working with PWIDs will administer the supplemental questionnaire. Instruments will be piloted in order to ensure that questions are culturally sensitive to the situational factors faced by PWIDs in Tijuana and San Diego and that the interview will not exceed one hour in order to reduce respondent burden.  We will implement SARSCoV-2 transmission control measures following CDC Guidelines for healthcare settings adapted to our study site: potential participants will be given a face protection device upon entering the premises to be worn at all times while on the study premises and will be requested to wash their hands with soap and water or with hand sanitizer; next, participants will be screened in the open air in the area designated for this purpose with plenty of air flow; only two participants will be seen at a given time by study staff: one completing the symptom screening questionnaire while the second one is undergoing biological sample collection and testing; both stations will be placed at least 15 feet apart to guarantee social distancing at all times. Study staff will wear face protection, N95 masks, nitrile gloves at all times and any other protective equipment deemed necessary. We will also setup clear, plastic screens to provide a physical barrier between study participant and study staff while completing the supplemental survey. Upon completion of all study measures, study participants will be given an extra facial protection and useful information about SARSCoV-2 preventive measures and led to the facility’s exit area. We have developed a detailed protocol based on CDC Guidelines for SARSCoV-2 screening in community settings (See Appendix 1) |
| **16. PRIVACY AND CONFIDENTIALITY CONSIDERATIONS INCLUDING DATA ACCESS AND MANAGEMENT** |
| To guard confidentiality, only the participant’s code number will appear on questionnaires and specimens. At the research sites in Tijuana and San Diego, the key to participants’ code numbers will be encrypted in a computer file, which will be locked in the site co-investigator’s office. Only the Project Coordinator, Field Coordinator or Dr. Vera will be able to unencrypt the computer file. Questionnaire data gathered through interviews will be shipped to San Diego on a weekly basis. The only materials that will be stored at the research site will be contact information, including locator sheets, so that participants can be followed throughout the study period. This information will be stored in locked file cabinets in the co-investigator’s locked office. Questionnaire data will be entered into computer files and digitally transferred on a regular basis to UCSD for permanent storage. At UCSD, computer records will be protected by standard measures that limit access to the data to research project personnel. All computer files will be identifiable only by the participant’s code number. Participants’ names will never be used. At UCSD, the single name to ID relational file will be kept in an encrypted form, which only Dr. Strathdee and Dr. Vera will have access to. Other security considerations at UCSD include: Physical security: Computer files are kept in locked rooms accessible only to authorized personnel. Any paper records that reflect electronic data are kept in locked file cabinets. Rotating Backup Systems: Full volume tape backups are done weekly and any changed data are backed up daily. Other mechanisms include: security workshops; regular meetings of the Data Management Committee, and written security policy procedures. No individual participant data will be identified in any report or publication distributed beyond the research team. Audio recordings will be uploaded to a secure, encrypted computer off-site for secure transcription and storage by the research team.  Field notes and locator forms, which are necessary for participant follow-up tracking, will be stored in cabinets at the study offices in San Diego and Tijuana, respectively. The cabinets will be locked with coded locks, and the codes will be made available only to authorized staff. Additionally, the codes will be changed periodically. Pre-baseline screening, baseline and follow-up interviews will be conducted electronically using the QDS Audio Computer Administered Self Interview (ACASI) module on “field” laptops. The password for this database will only be provided to the database manager (Ms. Sajina Shakya) and the principal statistician (Ms. Abramovitz). Additionally, only the computers used by Ms. Shakya and the Ms. Abramovitz will be equipped with the QDS Warehouse Manager module, which makes possible data extraction. In cases when computer problems arise, which we expect to be rare, pre-baseline screening information, interviews, or lab results may be collected using pencil and paper forms. The forms will be initially stored at the corresponding study offices in separate locked cabinets and entered electronically as soon as possible. Then, on a weekly basis, these forms will be transported to the UCSD data center (which is located at the HNRC offices in Hillcrest) and immediately stored in locked cabinets. The data collected at the study offices electronically (i.e., pre-baseline screening, survey data, and laboratory data) will be transferred weekly by the database manager(s) from the “field” laptops onto the database manager’s computer. The aforementioned data transfer will take place remotely, using TeamViewer which has an infrastructure that “is ISO 27001 certified and completely HIPAA and SOC2 compliant” (https://www.teamviewer.com/en/products/teamviewer/).  Medical record information will be de-identified of any personally identifiable information of the study participant. Records will instead be marked with the participant’s code number which will be unique to this study. Data will be stored on encrypted electronic folders and password protected. Data will only be accessible to the PI and the study coordinator.  At the study offices, at the end of each workday, the data from all the “field” laptops will be backed up by the study coordinator onto an external hard drive. Then, all the laptops and the external hard drive will be locked up in separate cabinets. At the UCSD data center, where all the data will be collected, stored and processed by Ms. Shakya, only she and Ms. Abramovitz will be able to access the data using their UCSD active directory credentials and additional passwords, as needed. Regarding the physical security of the computers and cabinets located at the data center, the corresponding suite is located inside a building that has video surveillance and which during off hours is locked and can be accessed only by authorized persons via a key padded lock. Furthermore, the entry door to the suite is kept locked during all hours and only the staff working in the suite and the building manager have keys to the suite. Furthermore, the center itself is protected by motion sensing security system when no one is on site. UCSD security responds to the alarm within minutes.  Communication between UCLA and UCSD personnel will occur orally in private settings, and/or handed over physically on encrypted flash keys and/or sent through encrypted e-mail and/or uploaded to HIPPA compliant servers maintained in locked offices. Ethnographic fieldnotes, transcriptions, audio, and visual data that may have private identifying information including self-reported health information will be securely stored in encrypted machines in locked offices at the Tijuana and San Diego study site respectively. Again, the codes to the locked cabinets will only be available through authorized staff and the codes will be changed periodically. The department of psychiatry IT office in UCLA will maintain the encrypted Zotero server of ethnographic field notes, transcriptions, audio, and visual data. |
| **17. POTENTIAL BENEFITS** |
| Benefits to the subject include free confidential testing and counseling for HIV/HCV, as well as facilitated referrals for medical/social care, in a population that has traditionally been medically underserved. The testing of participants to determine HIV/HCV serostatus at baseline and six-month follow up for the follow-up period will potentially lead to early identification and early treatment of HIV/HCV. The use of rapid HIV/HCV testing can also maximize risk reduction benefits, since respondents receive their test results within 20 minutes. In addition, voluntary HIV/HCV testing and counseling (VCT) has been shown to generate reductions in unprotected sex among PWID.  Through this project, we have also strived to continue developing research capacity among our Mexican and US collaborators. For example, our team has conducted research in TJ since 2004 which has significantly influenced Mexican HIV prevention. This study is led by Dr Steffanie Strathdee an epidemiologist with >20 years’ experience and >600 publications on HIV prevention research, especially PWID. In TJ, she has led a PWID cohort, Proyecto El Cuete which is supported through a MERIT Award through 2020 and was Co-I on the STAHR studies in SD that ended in 2014.  Sub-Study #1 (COVID19 Prevalence and Network Features) Potential Benefits:  Benefits to the subject include SARS-CoV-2 testing, again in a population that has traditionally been medically underserved. The testing of participants to determine SARS CoV-2 serostatus at supplemental visit will potentially lead to early identification and early treatment of SARS CoV-2. In addition, voluntary SARS CoV-2 testing and counseling has been shown to generate reductions in disease transmission in the community.  Sub-Study #2 Potential Benefits:  Benefits to the subject include SARS-CoV-2 antibody testing, linkage to OnPoint SSP services, and referrals for COVID-19 vaccination. Vaccinations will generate reductions in COVID-19 disease transmission in San Diego and Tijuana, which will improve general public health. |
| **18. RISK/BENEFIT RATIO** |
| The direct benefits that subjects can derive from participating in the study are: a) obtaining results of testing for HIV,HCV, and SARS-CoV-2; b) obtaining information on disease transmission, treatment, and protection from infection; c) information about drug treatment programs in their local community; d) respectful companionship and advocacy for emergency and supportive services by ethnographic, epidemiologic survey administrators and outreach personnel. Potential significant risks associated with the research are breach of confidentiality about study participation or personal information collected in the screener, interview, and testing. Since steps are taken by the researchers to mitigate the probability of the occurrence of these risks, we feel that the potential benefits outweigh the potential risks identified. |
| **19. EXPENSE TO PARTICIPANT** |
| None other than time taken and, depending on where they live and work, transportation to the clinic. |
| **20. COMPENSATION FOR PARTICIPATION** |
| **Screening**: During screening, participants will be compensated a total of $5 US dollars to complete the screening regardless of their eligibility to participate in the study.  **Quantitative Questionnaire Reimbursement**: Each of the subjects recruited will receive $20 for their baseline interview and each time they complete a semi-annually administered interview. There will be 8 interviews in total, for a maximum compensation of $160.  **Locator Form Incentives**: We have found that attrition is reduced if we conduct ‘locator check-ins’ with each participant mid-way between their scheduled visits for a normal reimbursement. Each 200 NDTs from SD will receive $5 for updating their locator form. There will be 8 locator check-ins in total, for a maximum compensation of $40.  **Ethnographic Sub-cohort Incentives:** We will compensate for the 75 PWID in the sub-cohort of the main study $40 per session twice a year for intensive participant observation and in-depth interviews at locations where they buy and inject drugs to surveil the retail drug markets and surrounding circumstances. There will be 8 interviews in total for a maximum compensation of $320.  **SARS-CoV-2 Sub-Study #1 (COVID19 Prevalence and Network Features)**: Study participants are scheduled to receive a total of $20 dollars upon completion of biological sample collection and supplemental survey.  Participants who are recruited because of their knowledge of drug users, services, etc. and are not injection drug users will not be provided compensation. This is because they are not subject to the requirements of the six-month follow-up interview over the course of the project and they are also not subject to the testing of drug paraphernalia.  The total possible compensation if the study participant took the screener, attended every semi-annual interview, HCV/HIV tests, drug tests, and locator check-in will be $545 over the course of a 5 year study. We view the above amounts as reasonable and not excessive compensation to study participants for their time and transportation costs. |
| **21. PRIVILEGES/CERTIFICATIONS/LICENSES AND RESEARCH TEAM RESPONSIBILITIES** |
| Steffanie A. Strathdee, Ph.D.— Principal Investigator for the project. Dr. Strathdee began her appointment in January of 2004 at UCSD as Professor and is currently Harold Simon Chair, Associate Dean of Global Health Sciences. As an HIV epidemiologist with 15 years of experience, a central focus of Dr. Strathdee’s new research program will be studies of HIV/AIDS among high risk populations in Mexico. To this end, she designed this collaborative investigation of injection drug users in Tijuana and San Diego. She will be taking the lead role manuscript writing.  Thomas L. Patterson, Ph.D.— Co-investigator for the project. His study of female sex workers in both cities has been previously approved by the IRB and provides an important infrastructure and logistical base for the proposed study. He contributed to the study design of this project and will play a key role in its coordination.  Annick Bórquez – Co-investigator for the project. A Mexican-born infectious disease modeler who received her Masters and PhD training in epidemiology at the Department of Infectious Disease Epidemiology at Imperial College London. She is an Assistant Professor in the Department of Medicine (Division of Infectious Diseases and Global Health. Dr Borquez will oversee the modeling of the overdose and HIV outcomes in Aim 5. As a native Spanish speaker, she will also co-supervise the Data Center and the integration of data from Aims 1-4 into modeling scenarios in Aim 5.  Antoine Chaillon, MD, PhD – Co-investigator for the project. He is an Assistant Professor in the Department of Medicine (Division of Infectious Diseases and Global Health) at UCSD. He has also developed expertise in spatial genetics with the joint use of genetic and spatial information to explore viral phylodynamics. These new tools can help understanding the past demographic history of microorganisms and the factors (e.g. environmental, epidemiological) that may have influenced it. In this project, he will be responsible for the following aspects of Aim 3: 1) developing phylogeographic models of viral dispersal, 2) processing and analyzing next generation sequencing data, 3) molecular evolutionary analyses, and 4) HIV transmission network analysis. He work closely with Dr. Strathdee and Mehta to develop and utilize the latest techniques to maximize the accuracy of phylogenetic relationships of sampled viral sequence data. He will also play a role in applying data to the proposed modeling work.  Natasha K Martin, ScD – Co-investigator for the project. She is an Associate Professor in the Department of Medicine (Division of Infectious Diseases and Global Health) at UCSD. She is an infectious disease economic modeler who develops dynamic transmission models to evaluate the impact and cost-effectiveness of public health interventions. She has worked for 18 years developing mathematical models of disease progression and transmission in both communicable and non-communicable diseases. Given her substantial expertise, Dr. Martin is uniquely poised to conduct the modeling work predicting the HIV and HCV epidemics among PWID in Tijuana and San Diego, working closely with Dr. Borquez on the overdose models.  Sanjay R. Mehta, MD – Co-investigator for the project. He is an Assistant Professor in the Department of Medicine (Division of Infectious Diseases and Global Health) at UCSD. Trained in internal medicine and infectious diseases, his research interests are in the molecular epidemiology of HIV, and has worked with Drs. Strathdee and Patterson to study HIV transmission dynamics on the Mexico-US border by combining viral genetic, geographic and socio-demographic information to model HIV transmission patterns. Dr. Mehta is an internationally recognized researcher in this field and will work closely with Dr. Chaillon to interpret phylo-geographic analyses related to cross-border HIV and HCV transmission patterns.  Philippe Bourgois, PhD – Co-investigator for the project. He is a Spanish speaking medical anthropologist who directs the Center for Social Medicine at UCLA. He has a two decade long history of collaboration with Dr Strathdee using multi-methods (ethnography/epidemiology) to document rapidly changing HIV risk-environments among street-based, out-of-treatment injection drug users. He has dedicated his career to operationalizing the application of social science theories to urgent public health problems to develop upstream structural HIV prevention and treatment interventions. Dr. Bourgois will oversee the ethnographic field work and analysis in both San Diego and Tijuana, and will work closely with Dr. Strathdee to triangulate qualitative and quantitative findings to inform future interventions and the proposed modeling.  Gudelia Rangel – Sub-contract Principal investigator for the project. She is the Baja California State Coordinator of the Comision Fronteriza (US Mexico Border Health Comission), a position to which she was appointed by the Secretary of Health of Baja California. She received her PhD. in health sciences with a concentration in epidemiology from the Instituto Nacional de Salud Pública in Cuernavaca, Morelos, which is the only accredited public health doctoral program outside of the U.S. In light of her track record, she is eminently qualified to be the PI of the proposed sub-contract and will oversee field operations including budgetary oversight in Tijuana. On the present study, Dr. Rangel participate in regular investigators’ meetings, data analysis and interpretation, the writing of articles based on study findings.  Tetyana Vasyleva, PhD – Consultant for the project. Dr. Vasyleva is an HIV molecular epidemiologist based at the University of Oxford who specializes in modeling dispersion of HIV associated with population mobility. She will serve as a consultant advising on phylodynamic and phylogeographic analyses.  Alicia Harvey-Vera, MPH, PhD – Project Manager for the project. Dr Vera is fully bilingual and bicultural with an MPH and PhD from the Universidad Autonoma de Baja California. She has directed our research projects on both sides of the San Diego/Tijuana border for 15 years and has a SENTRI pass to facilitate border crossing. She has expertise in both qualitative and quantitative data collection with marginalized populations. She will supervise the team’s field work in ethnographic and quantitative survey data collection. She will be responsible for hiring, supervising and the training of interviewers and outreach workers together with Drs. Strathdee and Bourgois; piloting and revising the study instruments; creating protocols for maintaining all study-related documents; overseeing subject screening, interviewing, follow-up and daily data transfer; preparing and maintaining human subjects protocols in the U.S. and Mexico and liaising with co-investigators, field staff and policy makers.  Daniela Abramovitz, M.Sc – Senior statistician for the project. Ms. Abramovitz is a biostatistician who completed all the course requirements for a PhD in biostatistics. She has >15 years’ experience in data management and analysis and joined our UCSD research team in 2005. She has overseen analyses for all of our San Diego and Tijuana studies, including those that involved cross-border data collection (i.e., Sexo Seguro). She is familiar with relational databases and with data maintained in SAS and SPSS, as well as software for spatial analyses (ArcView), and she is very experienced with RDS methodology, including its assumptions and weighting using RDSSat software. She has co-authored a paper on RDS methods applied to our PWID cohort in Tijuana. Abramovitz is also well versed in statistical techniques for prospective cohort studies, including random effects models, Cox regression, Poisson regression, hierarchical modeling techniques and mediation analyses. She will oversee Ms. Shakya’s data management and programming of the baseline and follow-up surveys in QDS. Ms. Abramowitz will generate follow-up reports and conduct all statistical analysis for progress reports, abstracts and manuscripts.  Sajina Shakya, MS. – Quantitative Data Manager for the project. Ms. Shakya is a data manager who has worked for our binational research team for 5 years. She will be responsible for programming the baseline and follow-up surveys in QDS to allow the interviews to be conducted using CAPI. She will also monitor the transfer of data collected at the Tijuana and Ciudad Juarez sites and will concatenate data from each timepoint into the study’s central database. She will develop a system for tracking participants’ visits and keeping complete records, organizing and filing data, and storing consent forms. She will also generate summary data reports and conduct basic statistical analyses under the supervision of Ms. Abramovitz.  **Sub-Study #1 (COVID19 Prevalence and Network Features) Additional Faculty**:  **Britt Skaathun, PhD, MPH** is an expert in social network analysis and infectious disease transmission.  **Jack Gilbert, PhD** is an expert in microbial ecology and microbiome analysis techniques and will participate with no salary requested.  **Sarah Allard, PhD** is also an expert in microbial ecology and will carry out microbial and SARSCoV-2 sample collection and analysis.  Sub-Study #2 Additional Faculty:  **Davey Smith, MD** is a Professor of Medicine at the University of California, San Diego (UCSD), as well as an Infectious Disease physician and HIV researcher. He is currently the co-director and PI of the San Diego Center for AIDS Research (CFAR) and Director of the Antiviral Research Center (AVRC) Lab, which has a CLIA that covers rapid antigen testing for this project. He has a broad background in clinical HIV medicine, molecular virology, substance use, and bioinformatics, with specific training and expertise in key research areas for this application. He will provide expertise in issues related to COVID-19 diagnostics and vaccines and will facilitate the clinical care for any subjects diagnosed with COVID-19. He will serve as a member of the Steering Committee, will co-chair the Human Subjects Unit, and facilitate communications across RAD and RADxUP consortia. |
| **22. BIBLIOGRAPHY** |
| 1. Goodman-Meza, D., et al., *Where Is the Opioid Use Epidemic in Mexico? A Cautionary Tale for Policymakers South of the US-Mexico Border.* Am J Public Health, 2018: p. e1-e10.  2. National Institute of Drug Abuse (NIDA). *Overdose Death Rates*. 2018 10/01/2018]; Available from: <https://www.drugabuse.gov/related-topics/trends-statistics/overdose-death-rates>.  3. Conrad, C., et al., *Community Outbreak of HIV Infection Linked to Injection Drug Use of Oxymorphone--Indiana, 2015.* MMWR Morb Mortal Wkly Rep, 2015. **64**(16): p. 443-4.  4. Strathdee, S.A. and C. Beyrer, *HIV Outbreak in Indiana.* N Engl J Med, 2015. **373**(14): p. 1380-1.  5. Drug Enforcement Administration (DEA), *2017 National Drug Threat Assessment*. Washington DC Department of Justice. p. Report # DEA-DCT-DIR-040-17.  6. Wagner, K.D., et al., *The social and environmental context of cross-border drug use in Mexico: findings from a mixed methods study of young injection drug users living in San Diego, CA.* J Ethn Subst Abuse, 2012. **11**(4): p. 362-78.  7. Horyniak, D., et al., *Cross-border injection drug use and HIV and hepatitis C virus seropositivity among people who inject drugs in San Diego, California.* Int J Drug Policy, 2017. **47**: p. 9-17.  8. Macy, B., *Dopesick: Dealers, Doctors and the Drug Company that Addicted America*. 2018: Head of Zeus Ltd.  9. Bucardo, J., et al., *Historical trends in the production and consumption of illicit drugs in Mexico: implications for the prevention of blood borne infections.* Drug Alcohol Depend, 2005. **79**(3): p. 281-93.  10. Mehta, S.R., et al., *HIV transmission networks in the San Diego–Tijuana border region.* EBioMedicine, 2015. **2**(10): p. 1456-1463.  11. Mars, S.G., et al., *The textures of heroin: User perspectives on “black tar” and powder heroin in two US cities.* Journal of Psychoactive Drugs, 2016. **48**(4): p. 270-278.  12. Poklis, A., *Fentanyl: a review for clinical and analytical toxicologists.* Journal of Toxicology: Clinical Toxicology, 1995. **33**(5): p. 439-447.  13. Talu, A., et al., *HIV infection and risk behaviour of primary fentanyl and amphetamine injectors in Tallinn, Estonia: implications for intervention.* Int J Drug Policy, 2010. **21**(1): p. 56-63.  14. Ciccarone, D. and P. Bourgois, *Explaining the geographical variation of HIV among injection drug users in the United States.* Subst Use Misuse, 2003. **38**(14): p. 2049-63.  15. Roth, A.M., et al., *Cold Preparation of Heroin in a Black Tar Market.* Substance use & misuse, 2017. **52**(9): p. 1242-1246.  16. Office of AIDS Research (OAR). *NIH HIV/AIDS Research Priorities and Guidelines for Determining AIDS Funding*. 2018 [cited 2018 12/13/18]; Available from: <https://grants.nih.gov/grants/guide/notice-files/NOT-OD-15-137.html>.  17. Lavine, J.S., O.N. Bjornstad, and R. Antia, *Immunological characteristics govern the transition of COVID-19 to endemicity.* Science, 2021. **371**(6530): p. 741-745.  18. Aschwanden, C., *Five reasons why COVID herd immunity is probably impossible*, in *Nature*. 2021, Nature. p. 520-522.  19. Whiteman, A., et al., *Demographic and Social Factors Associated with COVID-19 Vaccination Initiation Among Adults Aged ≥65 Years — United States, December 14, 2020–April 10, 2021.* MMWR Morb Mortal Wkly Rep, 2021.  20. California For All. *Vaccination Progress Data*. 2021 [cited 2021 May 5]; Available from: <https://covid19.ca.gov/vaccination-progress-data/#progress-by-group>.  21. Branch, C.o.S.D.-H.a.H.S.A.-P.H.S.-E.a.I.S. *County of San Diego - COVID-19 Vaccination*. 2021 [cited 2021 May 5]; Available from: <https://sdcounty.maps.arcgis.com/apps/dashboards/c0f4b16356b840478dfdd50d1630ff2a>.  22. Romer, D. and K.H. Jamieson, *Conspiracy theories as barriers to controlling the spread of COVID-19 in the U.S.* Soc Sci Med, 2020. **263**: p. 113356.  23. Romer, D. and K.H. Jamieson, *Patterns of Media Use, Strength of Belief in COVID-19 Conspiracy Theories, and the Prevention of COVID-19 From March to July 2020 in the United States: Survey Study.* J Med Internet Res, 2021. **23**(4): p. e25215.  24. NASTAD. *COVID-19 Vaccine: Guidance for Syringe Sharing Services Programs, Health Departments, and People Who Use Drugs*. 2021 [cited 2021 May 12]; Available from: <https://www.nastad.org/resource/covid-19-vaccine-guidance-syringe-services-programs-health-departments-and-people-who-use-0>.  25. AIDS, C.D.o.P.H.-C.f.I.D.O.o. *Guidelines for Syringe Exchange Programs Funded by the California Department of Public Health, Office of AIDS*. 2021 [cited 2021 May 5]; Available from: <https://www.cdph.ca.gov/Programs/CID/DOA/CDPH%20Document%20Library/Guidelines%20for%20SEPs_ADA.pdf>.  26. Health, C.D.o.P. *Laying a Foundation for Getting to Zero: California's Integrated HIV Surveillence, Prevention, and Care Plan*. 2016 [cited 2021 May 5]; Available from: <https://www.cdph.ca.gov/Programs/CID/DOA/CDPH%20Document%20Library/IP_2016_Final_ADA.pdf>.  27. Warmth, G. *Supervisors approve needle exchange for drug users*. The San Diego Tribune, 2021.  28. Lopez, A.M., et al., *Interdisciplinary mixed methods research with structurally vulnerable populations: case studies of injection drug users in San Francisco.* Int J Drug Policy, 2013. **24**(2): p. 101-9.  29. Bourgois, P., et al., *Reinterpreting ethnic patterns among white and African American men who inject heroin: a social science of medicine approach.* PLoS Med, 2006. **3**(10): p. e452.  30. Bourgois, P., B. Prince, and A. Moss, *The Everyday Violence of Hepatitis C Among Young Women Who Inject Drugs in San Francisco.* Hum Organ, 2004. **63**(3): p. 253-264.  31. Hahn, J.A., et al., *Hepatitis C virus seroconversion among young injection drug users: relationships and risks.* J Infect Dis, 2002. **186**(11): p. 1558-64.  32. Branas, C.C., et al., *Citywide cluster randomized trial to restore blighted vacant land and its effects on violence, crime, and fear.* Proc Natl Acad Sci U S A, 2018. **115**(12): p. 2946-2951.  33. Messac, L., et al., *The good-enough science-and-politics of anthropological collaboration with evidence-based clinical research: Four ethnographic case studies.* Soc Sci Med, 2013. **99**: p. 176-86.  34. Shu, Y. and J. McCauley, *GISAID: Global initiative on sharing all influenza data - from vision to reality.* Euro surveillance : bulletin Europeen sur les maladies transmissibles = European communicable disease bulletin, 2017. **22**(13): p. 30494.  35. Elbe, S. and G. Buckland-Merrett, *Data, disease and diplomacy: GISAID's innovative contribution to global health.* Global Challenges, 2017. **1**(1): p. 33-46.  36. Lemey, P., et al., *Bayesian phylogeography finds its roots.* PLoS Computational Biology, 2009. **5**(9).  37. Edwards, C.J., et al., *Ancient hybridization and an Irish origin for the modern polar bear matriline.* Current biology: CB, 2011. **21**(15): p. 1251-1258.  38. Perez, A.B., et al., *Increasing importance of European lineages in seeding the hepatitis C virus subtype 1a epidemic in Spain.* Euro Surveill, 2019. **24**(9).  39. Faria, N.R., et al., *Phylogeographical footprint of colonial history in the global dispersal of human immunodeficiency virus type 2 group A.* The Journal of general virology, 2012. **93**(Pt 4): p. 889-899.  40. Suchard, M.A., et al., *Bayesian phylogenetic and phylodynamic data integration using BEAST 1.10.* Virus Evol, 2018. **4**(1): p. vey016.  41. Minin, V.N. and M.A. Suchard, *Counting labeled transitions in continuous-time Markov models of evolution.* Journal of mathematical biology, 2008. **56**(3): p. 391-412.  42. Minin, V.N., E.W. Bloomquist, and M.A. Suchard, *Smooth skyride through a rough skyline: Bayesian coalescent-based inference of population dynamics.* Molecular Biology and Evolution, 2008. **25**(7): p. 1459-1471.  43. Rambaut, A. *Phylogenetic analysis of nCoV-2019 genomes*. 2020 [cited 2020 March 16]; Available from: <http://virological.org/t/phylodynamic-analysis-176-genomes-6-mar-2020/356>.  44. Chaillon, A., et al., *HIV persists throughout deep tissues with repopulation from multiple anatomical sources* Journal of Clinical Investigation, 2020: p. In Press.  45. Lemey, P., et al., *Unifying viral genetics and human transportation data to predict the global transmission dynamics of human influenza H3N2.* PLoS Pathog, 2014. **10**(2): p. e1003932.  46. Prevention, C.f.D.C.a. *Myths and Facts about COVID-19 Vaccines*. 2021 [cited 2021 May 5]; Available from: <https://www.cdc.gov/coronavirus/2019-ncov/vaccines/facts.html>.  47. Prince-Guerra, J.L., et al., *Evaluation of Abbott BinaxNOW Rapid Antigen Test for SARS-CoV-2 Infection at Two Community-Based Testing Sites - Pima County, Arizona, November 3-17, 2020.* MMWR Morb Mortal Wkly Rep, 2021. **70**(3): p. 100-105.  48. Centers for Disease Control and Prevention, *Erratum.* MMWR Morb Mortal Wkly Rep, 2021. **70**(144).  49. UCLA Institute for Digital Research & Eductation Statistical Consulting. *How can I compute Durbin-Watson statistic and 1st order autocorrelation in time series data? SAS FAQ*. 2021 [cited 2021 May 14]; Available from: <https://stats.idre.ucla.edu/sas/faq/how-can-i-compute-durbin-watson-statistic-and-1st-order-autocorrelation-in-time-series-data/>. |
| **23. FUNDING SUPPORT FOR THIS STUDY** |
| This main study is funded by the National Institutes of Health (NIH) – National Institute of Drug Abuse (NIDA) (R01 DA049644-01A1). The grant’s project period is from 04/01/2020 until 01/31/2025. The fiscal contact person for this work is Mr. William Gentz [wgentz@health.ucsd.edu](mailto:wgentz@health.ucsd.edu).  Substudy #1 is funded by an administrative supplement from the same grant (R01 DA049644-01A1S1).  Substudy #2 is anticipated to be funded by a competitive revision to La Frontera recently submitted as of 05/25/2021. The proposed start date for the project is in 09/01/2021. |
| **24. BIOLOGICAL MATERIALS TRANSFER AGREEMENT** |
| Transfer protocols will be worked out between field staff in Tijuana, field staff in San Diego, and the UCSD CFAR laboratory, Genalyte Laboratory, and CDC Laboratory to ensure the safe storage and timely testing of biosamples. |
| **25. INVESTIGATIONAL DRUG FACT SHEET AND IND/IDE HOLDER** |
| Not applicable. |
| **26. IMPACT ON NURSING STAFF** |
| Not applicable. |
| **27. CONFLICT OF INTEREST** |
| The researchers involved in the study do not have any conflict of interest. |
| **28. SUPPLEMENTAL INSTRUCTIONS FOR CANCER-RELATED STUDIES** |
| Not applicable. |
| **29. OTHER APPROVALS/REGULATED MATERIALS** |
| Not applicable. |
| **30. PROCEDURES FOR SURROGATE CONSENT AND/OR DECISIONAL CAPACITY ASSESSMENT** |
| The research staff who will be responsible for obtaining informed consent will assess whether the potential participant has understood the study and consent form by asking key questions (e.g., “How much time will this take you?”; “What are the possible benefits for you?”). Because it is possible that some of our participants may be cognitively impaired, or they may not initially understand the consent process, we will test all potential participants for their comprehension of critical points in the consent form. Errors will be corrected and these potential participants will then be asked if they need further clarification. If, after further attempts to clarify any misunderstandings, we determine that they may not fully comprehend the critical aspects of the study, they will not be enrolled. If a potential participant decides she or he does not wish to participate, his or her decision will be honored regardless of how well they comprehend the study information. |
