# Supplementary material for: Prevalence and correlates of SARS-CoV-2 seropositivity among people who inject drugs in the San Diego-Tijuana border region
Source: PLoS One. 2021 Nov 22;16(11):e0260286. doi: 10.1371/journal.pone.0260286 (PMC8608290; doi:10.1371/journal.pone.0260286)
Supplement: S2 File — (PDF) [file pone.0260286.s002.pdf]

TABLE OF CONTENTS

BASELINE QUESTIONNAIRE – ENGLISH VERSION 2

BASELINE QUESTIONNAIRE – SPANISH VERSION 345

SUPPLEMENTAL QUESTIONNAIRE – ENGLISH VERSION 717

SUPPLEMENTAL QUESTIONNAIRE – SPANISH VERSION 1074

La Frontera Project

Baseline Survey – English version

Disclaimer: This survey has been formatted for programming into QDS and is not intended to be administered on pencil and paper

PI: Steffanie Strathdee
10-18-2021

LaFrontera.Quant1.20210616 (English)

READ: La Frontera
Quantitative Survey 1 (Baseline)
English

VERSION = 20210616

Q1.	Interviewer: Please enter interview date.	__ __ / __ __ / __ __ __ __	mm / dd / yyyy

If Q1 is greater than TODAY and TODAY is not equal to "missing" then READ: "That date occurs in the future. Please check the entry." and skip to Q1.

Q2.	Interviewer Initials:		__ __ __ __
Q3.	Visit Number (Choose one)	1	Baseline (Visit 1)
Q4.	City of the Interview<br>**Interviewer, it is crucial that you enter the interview location correctly!**<br>(Choose one)	1	San Diego
		2	Tijuana

If Q4 is equal to 1 then skip to Q5

If not, then skip to instruction before Q7.

Q5.	Place of Interview (Choose one)	1	San Diego 1
		2	San Diego 2
		3	San Diego 3
		4	Rehabilitation Center
		5	Jail
		6	Other

If Q5 is not equal to 6, then skip to instruction before Q7.

Q6. Specify other place of Interview

____

If Q4 is equal to 2 then skip to Q7

If not, then skip to Q9.

Q7. Place of Interview (Choose one)

1 Tijuana 1 Zona Norte - El Mapa

2 Tijuana 2 Coahuila Office

3 Tijuana 3

4 Rehabilitation Center

5 Jail

6 Other

If Q7 is not equal to 6, then skip to Q9.

Q8. Specify other place of Interview

____

Q9. Interviewer, how will this interview be conducted? (Choose one)

1 In Person, Inside

2 In Person, Outside

3 Video call (e.g., zoom, skype)

4 Audio Call (e.g., phone, zoom)

5 Other

8 Refuse to Answer

If Q9 is not equal to 5, then skip to Q11.

Q10. Please, specify Other.

____

Q11. Participant ID Number (e.g., LF001): _____

ID1 = SubString(Q11, 0, 1)

ID2 = SubString(Q11, 1, 1)

ID3 = SubString(Q11, 2, 1)

ID4 = SubString(Q11, 3, 1)

ID5 = SubString(Q11, 4, 1)

IDSTUDY = Concatenated(ID1, ID2)

If IDSTUDY is not equal to LF then READ: "Check ID." and skip to Q11.

If ID3 is not equal to 0 and ID3 is not equal to 1 and ID3 is not equal to 2 and ID3 is not equal to 3 and ID3 is not equal to 4 and ID3 is not equal to 5 and ID3 is not equal to 6 and ID3 is not equal to 7 and ID3 is not equal to 8 and ID3 is not equal to 9 or ID4 is not equal to 0 and ID4 is not equal to 1 and ID4 is not equal to 2 and ID4 is not equal to 3 and ID4 is not equal to 4 and ID4 is not equal to 5 and ID4 is not equal to 6 and ID4 is not equal to 7 and ID4 is not equal to 8 and ID4 is not equal to 9 or ID5 is not equal to 0 and ID5 is not equal to 1 and ID5 is not equal to 2 and ID5 is not equal to 3 and ID5 is not equal to 4 and ID5 is not equal to 5 and ID5 is not equal to 6 and ID5 is not equal to 7 and ID5 is not equal to 8 and ID5 is not equal to 9 then READ: "Check ID." and skip to Q11.

Q12. Please enter the SCREENER ID of the Subject (e.g., LF0001).
NOTE TO THE INTERVIEWER: Please do not confuse the screener number with the Subject ID number!

______

SCRID1 = SubString(Q12, 0, 1)

SCRID2 = SubString(Q12, 1, 1)

SCRID3 = SubString(Q12, 2, 1)

SCRID4 = SubString(Q12, 3, 1)

SCRID5 = SubString(Q12, 4, 1)

SCRID6 = SubString(Q12, 5, 1)

IDSCREEN = Concatenated(SCRID1, SCRID2)

If IDSCREEN is not equal to LF then READ: "Check Screen ID." and skip to Q12.

If SCRID3 is not equal to 0 and SCRID3 is not equal to 1 and SCRID3 is not equal to 2 and SCRID3 is not equal to 3 and SCRID3 is not equal to 4 and SCRID3 is not equal to 5 and SCRID3 is not equal to 6 and SCRID3 is not equal to 7 and SCRID3 is not equal to 8 and SCRID3 is not equal to 9 or SCRID4 is not equal to 0 and SCRID4 is not equal to 1 and SCRID4 is not equal to 2 and SCRID4 is not equal to 3 and SCRID4 is not equal to 4 and SCRID4 is not equal to 5 and SCRID4 is not equal to 6 and SCRID4 is not equal to 7 and SCRID4 is not equal to 8 and SCRID4 is not equal to 9 or SCRID5 is not equal to 0 and SCRID5 is not equal to 1 and SCRID5 is not equal to 2 and SCRID5 is not equal to 3 and SCRID5 is not equal to 4 and SCRID5 is not equal to 5 and SCRID5 is not equal to 6 and SCRID5 is not equal to 7 and SCRID5 is not equal to 8 and SCRID5 is not equal to 9 or SCRID6 is not equal to 0 and SCRID6 is not equal to 1 and SCRID6 is not equal to 2 and SCRID6 is not equal to 3 and SCRID6 is not equal to 4 and SCRID6 is not equal to 5 and SCRID6 is not equal to 6 and SCRID6 is not equal to 7 and SCRID6 is not equal to 8 and SCRID6 is not equal to 9 then READ: "Check Screen ID." and skip to Q11.

Q13. What languages do you speak, even if not very well?
Interviewer: Read out the options and check all that apply (Check all that apply)

__ Spanish

__ English

__ Indigenous language

__ Other

__ Refuse to Answer

If Q13D is not equal to 1, then skip to instruction before Q15.

Q14. Please specify other language. ______________________________

If Q13B is not equal to 1, then skip to instruction before Q17.

Q15. How well do you **speak** English? (Choose one)

0	Fluently/Native speaker
1	Very well
2	OK
3	Not very well
8	Refuse to Answer

Q16. How well do you **Read/Write** English? (Choose one)

0	Perfectly
1	Very well
2	OK
3	Not very well
4	Not at all
8	Refuse to Answer

If Q13A is not equal to 1, then skip to instruction before A1.

Q17. How well do you **speak** Spanish? (Choose one)

0	Fluently/Native speaker
1	Very well
2	OK
3	Not very well
8	Refuse to Answer

Q18. How well do you **Read/Write** Spanish? (Choose one)

0	Perfectly
1	Very well
2	OK
3	Not very well
4	Not at all
8	Refuse to Answer

READ: INTERVIEW, BEFORE STARTING, PLEASE READ THE FOLLOWING TEXT ALOUD TO THE RESPONDENT:
This interview is part of a research study about HIV/HCV and overdose among people who inject drugs in San Diego and Tijuana. The questions we will be asking are related to some basic information about you, your drug use history, experiences in San Diego and Tijuana, sexual behaviors, utilization of drug treatment and other services, and your attitudes towards some of these issues. I know some of the questions we will ask may be a little uncomfortable for you to answer, but the interview is confidential, and we will not give any of your personal information to anyone outside of this project. Nothing that you tell us can be traced back to you as an individual. It is important that you know that your participation in this interview is entirely voluntary. You do not need to answer a question if you do not want to, and you may stop the interview at any point. Do you have any questions before we begin?

SCREENING

READ: INTERVIEWER READ TO THE RESPONDENT: First, I am going to ask you some basic information about yourself.

A1. What is your age, in years? __ __ __ Years Old

998 Refuse to Answer

A2. What is your date of birth? __ __ / __ __ / __ __ __ __ mm / dd / yyyy

2098 Refuse to Answer (Year)

If Age(A2, TODAY) is not equal to A1 and A1 is not equal to 998 and A2 is not equal to 2098 then READ: "The age and date of birth you have entered do not agree. Please check the responses." and skip to A1.

If A1 is less than 18 or A1 is equal to 998, then skip to end of questionnaire.

A3. When was the last time you injected drugs?
Interviewer: code "TODAY" as 0 days ago __ __ __ __ MONTHS

__ __ __ __ DAYS

9998 Refuse to Answer (Days)

If A3 is greater than 30 or A3 is equal to 9998, then skip to end of questionnaire.

A4. Have you previously been enrolled in any research study such as El Cuete, Mujer Segura, Mujer Mas Segura?

1 Yes

0 No

8 Refuse to Answer

If A4 is not equal to 1, then skip to instruction before A6.

A5. Which study did you participate in? (Check all that apply)

__ El Cuete

__ Mujer Saludable Siempre

__ Proyecto EPICO

__ Other

__ Refuse to Answer

If A5D is not equal to 1, then skip to A7.

A6. Please specify Which other project you participated in

____

A7. In what country do you live in? (Choose one)

1 United States

2 Mexico

3 Other

8 Refuse to Answer

If A7 is equal to 3 or A7 is equal to 8, then skip to end of questionnaire.

If A7 is equal to 1 then skip to A8

If not, then skip to instruction before A12.

A8. What region of San Diego County do you live in? (Choose one)

1 City of San Diego

2 East County

3 North County Coastal

4 North County Inland

5 South Bay

6 Other San Diego region

7 I do not live in San Diego County

8 Refuse to Answer

If A8 is not equal to 6, then skip to instruction before A10.

A9. Please specify what region you live in.

____

If A8 is equal to 7 or A8 is equal to 8, then skip to end of questionnaire.

A10. What City/Unincorporated areas do you live in? (Choose one)

01	Carlsbad
02	Chula Vista
03	Coronado
04	Del Mar
05	El Cajon
06	Encinitas
07	Escondido
08	Imperial Beach
09	La Mesa
10	Lemon Grove
11	National City
12	Oceanside
13	Poway
14	San Diego
15	San Marcos
16	Santee
17	Solana Beach
18	Vista
19	Other
98	Refuse to Answer

If A10 is not equal to 19, then skip to instruction before A12.

A11. Please specify other City/Unincorporated areas you live in

____

If A7 is equal to 2 then skip to A12

If not, then skip to instruction before A14.

A12. What city do you live in? (Choose one)

1	Tijuana
2	Mexicali
3	Ensenada
4	Rosarito
5	Other
8	Refuse to Answer

If A12 is not equal to 5, then skip to instruction before A14.

A13. Please specify other city you live in

____

If A12 is not equal to 1, then skip to end of questionnaire.

If A7 is equal to 1 then skip to instruction before A14

If not, then skip to instruction before A19.

If Q4 is equal to 1 then skip to A14

If not, then skip to A16.

A14. Have you ever been to Mexico?

1	Yes
0	No
8	Refuse to Answer

If A14 is not equal to 1, then skip to instruction before A19.

A15. When was the last time you were in Mexico? __ __ / __ __ / __ __ __ __ mm / dd / yyyy

2098 Refuse to Answer (Year)

If A15 is less than A2 and A15 is not equal to 2098 then READ: "You have indicated that the participant visited Mexico before their date of birth. Please check the response." and skip to A15.

A16. Have you ever used any drugs in Mexico?

1 Yes

0 No

8 Refuse to Answer

If A16 is not equal to 1, then skip to instruction before A19.

A17. Have you ever injected drugs in Mexico?

1 Yes

0 No

8 Refuse to Answer

If A17 is not equal to 1, then skip to instruction before A19.

A18. When was the last time you injected drugs in Mexico?

__ __ / __ __ / __ __ __ __ mm / dd / yyyy

2098 Refuse to Answer (Year)

If A18 is less than A2 and A18 is not equal to 2098 then READ: "You have indicated that the participant injected drugs before their date of birth. Please check the response." and skip to A18.

If Q4 is equal to 2 and A7 is equal to 2 then skip to A19

If not, then skip to instruction before A24.

A19. Have you ever been to the United States?

1 Yes

0 No

8 Refuse to Answer

If A19 is not equal to 1, then skip to instruction before A24.

A20. When was the last time you were in the US? __ __ / __ __ / __ __ __ __ mm / dd / yyyy

2098 Refuse to Answer (Year)

If A20 is less than A2 and A20 is not equal to 2098 then READ: "You have indicated that the participant visited Mexico before their date of birth. Please check the response." and skip to A20.

A21. Have you ever used any drugs in the US?

1	Yes
0	No
8	Refuse to Answer

If A21 is not equal to 1, then skip to instruction before A24.

A22. Have you ever injected drugs in the US?

1	Yes
0	No
8	Refuse to Answer

If A22 is not equal to 1, then skip to instruction before A24.

A23. When was the last time you injected drugs in the US?

__ __ / __ __ / __ __ __ __	mm / dd / yyyy
2098	Refuse to Answer (Year)

If A23 is less than A2 and A23 is not equal to 2098 then READ: "You have indicated that the participant injected drugs in the US before they used drug in the US. Please check the responses." and skip to A23.

If A7 is equal to 1 or A12 is equal to 1 and A19 is equal to 1 then skip to A24

If not, then skip to instruction before B1.

A24. Are you a US citizen or US legal resident?

1	Yes
0	No
8	Refuse to Answer

LCOUNTRY = If(A7 is equal to 1, the US, If(A7 is equal to 2, Mexico,))

LCITY = If(A8 is equal to 1 or A8 is equal to 2 or A8 is equal to 3 or A8 is equal to 4 or A8 is equal to 5 or A8 is equal to 6, San Diego County, If(A12 is equal to 1, Tijuana,))

DT_SD = If((A8 is equal to 1 or A8 is equal to 2 or A8 is equal to 3 or A8 is equal to 4 or A8 is equal to 5 or A8 is equal to 6) and A16 is equal to 1 and A17 is equal to 1 and A18 is not equal to 2098 and TODAY - A18 is less than 366, 1, 0)

NDT_SD = If(Q4 is equal to 1 and (A8 is equal to 1 or A8 is equal to 2 or A8 is equal to 3 or A8 is equal to 4 or A8 is equal to 5 or A8 is equal to 6) and (A14 is equal to 0 or A14 is equal to 1 and TODAY - A15 is greater than 730), 1, 0)

NDT_TJ = If(Q4 is equal to 2 and A12 is equal to 1 and (A19 is equal to 0 or A19 is equal to 1 and TODAY - A20 is greater than 730), 1, 0)

GROUP = If(DT_SD is equal to 1, 1, If(NDT_SD is equal to 1, 2, If(NDT_TJ is equal to 1, 3, 0)))

If GROUP is equal to 0, then skip to end of questionnaire.

DEMOGRAPHIC DATA

READ: INTERVIEWER READ TO THE RESPONDENT: Now, I am going to ask you some questions about your life and where you are you from.

If A7 is equal to 1 then skip to B1

If not, then skip to instruction before B3.

B1.	What is your height?	__ __	inch
		98	Refuse to Answer
B2.	What is your weight?	__ __ __	lb
		998	Refuse to Answer

If A7 is equal to 2 then skip to B3

If not, then skip to B5.

B3.	What is your height?	__ __ __	cm
		998	Refuse to Answer
B4.	What is your weight?	__ __ __	kg
		998	Refuse to Answer
B5.	In what country were you born? (Choose one)	1	United States
		2	Mexico
		3	Other
		7	Don't Know
		8	Refuse to Answer

If B5 is equal to 2, then skip to B8.

If B5 is equal to 1, then skip to B11.

If B5 is equal to 7 or B5 is equal to 8, then skip to B15.

B6. Please specify the other country in which you were born.

____

B7. Please tell me the state where you were born.

____

If 1 is equal to 1, then skip to B15.

B8. In which state were you born?
Interviewer: Please select the button that corresponds to the first letter of the state of birthplace.
(Choose one)

1	A - M
2	N - Z
8	Refuse to Answer

If B8 is not equal to 1, then skip to instruction before B10.

B9. **Interviewer: Please select the correct state of the participant's birthplace.** (Choose one)

01	Aguascalientes (Ags)
02	Baja California (BC)
03	Baja California Sur (BCS)
04	Chihuahua (Chih)
05	Colima (Col)
06	Campeche (Camp)
07	Coahuila (Coah)
08	Chiapas (Chis)
09	Distrito Federal (DF)
10	Durango (Dgo)
11	Guerrero (Gro)
12	Guanajuato (Gto)
13	Hidalgo (Hgo)
14	Jalisco (Jal)
15	Michoacán (Mich)
16	Morelos (Mor)
17	México (Mex or Edomex)
98	Refuse to Answer

If B8 is not equal to 2, then skip to instruction before B11.

B10. **Interviewer: Please select the correct state of the participant's birthplace.** (Choose one)

01	Nayarit (Nay)
02	Nuevo León (NL)
03	Oaxaca (Oax)
04	Puebla (Pue)
05	Quintana Roo (Q Roo)
06	Querétaro (Qro.)
07	Sinaloa (Sin)
08	San Luis Potosí (SLP)
09	Sonora (Son)
10	Tabasco (Tab)
11	Tlaxcala (Tlax)
12	Tamaulipas (Tamps)
13	Veracruz (Ver)
14	Yucatán (Yuc)
15	Zacatecas (Zac)
98	Refuse to Answer

If 1 is equal to 1, then skip to B15.

B11. In which state were you born?
Interviewer: Please select the button that corresponds to the first letter of the state of birthplace.
(Choose one)

1	A - I
2	K - R
3	S - Z
8	Refuse to Answer

If B11 is not equal to 1, then skip to instruction before B13.

B12. **Interviewer: Please select the correct state of the participant's birthplace.** (Choose one)

01	Alabama
02	Alaska
03	Arizona
04	Arkansas
05	California
06	Colorado
07	Connecticut
08	Delaware
09	Florida
10	Georgia
11	Hawaii
12	Idaho
13	Illinois
14	Indiana
15	Iowa
98	Refuse to Answer

If B11 is not equal to 2, then skip to instruction before B14.

B13. **Interviewer: Please select the correct state of the participant's birthplace.** (Choose one)

01	Kansas
02	Kentucky
03	Louisiana
04	Maine
05	Maryland
06	Massachusetts
07	Michigan
08	Minnesota
09	Mississippi
10	Missouri
11	Montana
12	Nebraska
13	Nevada
14	New Hampshire
15	New Jersey
16	New Mexico
17	New York
18	North Carolina
19	North Dakota
20	Ohio
21	Oklahoma
22	Oregon
23	Pennsylvania

24 Rhode Island

98 Refuse to Answer

If B11 is not equal to 3, then skip to B15.

B14. **Interviewer: Please select the correct state of the participant's birthplace.** (Choose one)

01 South Carolina

02 South Dakota

03 Tennessee

04 Texas

05 Utah

06 Vermont

07 Virginia

08 Washington

09 Washington, D.C.

10 West Virginia

11 Wisconsin

12 Wyoming

98 Refuse to Answer

B15. Please tell me the city where you were born.

____

B16. What kinds of identification do you own? (Check all that apply)

__ US birth certificate

__ Mexico birth certificate

__ Passport

__ Sentri pass

__ Global Entri pass

__ Ready Lane pass

__ US Tourist visa

__ US Work visa

__ Credencial de elector (INE) Mexican ID

__ US Drivers license

__ Other border crossing document

__ Other

__ None of the above

__ Refuse to Answer

If B16M is equal to 1 and B16A + B16B + B16C + B16D + B16E + B16F + B16G + B16H + B16I + B16J + B16K + B16L is greater than 0 then READ: "You can only choose the response "None of the above" by itself. Please check responses." and skip to B16.

If B16L is not equal to 1, then skip to instruction before B18.

B17. Please, specify other documents

____

If B16C is not equal to 1, then skip to instruction before B20.

B18. What types of passports do you own?
Interviewer: any passport is OK, even if not legal.
(Choose one)

1	Mexican
2	US
3	Both
4	I dont own a passport
5	Other
8	Refuse to Answer

If B18 is not equal to 5, then skip to instruction before B20.

B19. Please, specify other passport

____

If B5 is not equal to 1 and (B18 is equal to 2 or B18 is equal to 3) then skip to B20

If not, then skip to B21.

B20. When did you become a US citizen?
Please enter the date of naturalization __ __ / __ __ / __ __ __ __ mm / dd / yyyy

2098 Refuse to Answer (Year)

If B20 is less than A2 and B20 is not equal to "skipped" and B20 is not equal to 2098 then READ: "Participant has responded with a length of time greater than his or her age. Please check the responses." and skip to B20.

B21. Do you consider yourself to be Hispanic/Latino/Mexican?

1	Yes
0	No
8	Refuse to Answer

B22. What racial group or groups do you consider yourself to be?
Interviewer: Read out the options and check all that apply (Check all that apply)

__ White

__ Black

__ Asian

__ Native Hawaiian or other Pacific Islander

__ Indigenous/ American Indian/ Alaskan Native

__ Mixed race

__ Other

__ Refuse to Answer

If B22G is not equal to 1, then skip to B24.

B23. Please specify other racial group

— —

B24. **Interviewer, if necessary, ask: What do you consider your gender to be?**
(Choose one)

1 Man

2 Woman

3 Transgender woman or transfeminine

4 Transgender man or transmasculine

5 Nonbinary

6 Other

8 Refuse to Answer

If B24 is not equal to 6, then skip to B26.

B25. Please specify other gender you consider yours to be

— —

B26. What sex was listed on your birth certificate? (Choose one)

1	Male
2	Female
3	Intersex
8	Refuse to Answer

B27. What is your marital status? (Choose one)

0	Single/ never married
1	Married/common law
2	Divorced
3	Separated
4	Widowed
8	Refuse to Answer

B28. What is the highest level of schooling you completed? (Choose one)

00	None, no formal education
01	Incomplete Primary School
02	Complete Primary School
03	Incomplete Secondary School
04	Complete Secondary School
05	Incomplete Preparatory
06	Complete Preparatory
07	Technical/Trade
08	University/College
09	Other
98	Refuse to Answer

If B28 is not equal to 9, then skip to instruction before B30.

B29. Please specify other level of schooling completed.

____

If B28 is equal to 0 or B28 is equal to 98, then skip to instruction before B31.

B30. Then in total, how many years of education have you completed?
Interviewer: If participant says Do not know, ask them for their best guess or estimate

__ __	Years
98	Refuse to Answer

If B30 is greater than A1 and B30 is not equal to 98 then READ: "The participant has responded that their years of education are greater than their age." and skip to B30.

B31. In the past 6 months, tell me if you have lived in or slept in any of the following places. **Interviewer: Read out list and check all that apply** (Check all that apply)

__ Parent's house or apartment

__ In your own house or apartment

__ Your spouse's/sexual partners house or apartment

__ Family house or apartment

__ Friend's house or apartment

__ Migrant worker's camp

__ Asylum seekers shelter

__ Shelter, welfare residence

__ Work place

__ Rented room (hotel, motel or other rooming house)

__ Car, bus, truck or other vehicle

__ Abandoned Building

__ Deportee shelter/camp

__ Correctional Institution (jail, prison, detention center)

__ Drug treatment center

__ On the Streets, beach, canal

__ Shooting gallery

__ Medical care facility (i.e., hospital, hospice, or nursing home)

__ Other

__ Refuse to Answer

If B31S is not equal to 1, then skip to instruction before B33.

B32. Please specify the other place where you slept or lived in the past 6 months.

____

If B31A + B31B + B31C + B31D + B31E + B31F + B31G + B31H + B31I + B31J + B31K + B31L + B31M + B31N + B31O + B31P + B31Q + B31R + B31S is equal to 1 or B31 is equal to 98, then skip to B35.

B33. In the past 6 months, which place did you sleep in <u>most of the time?</u> (Choose one)

01	Parent's house or apartment
02	In your own house or apartment
03	Your spouse's/sexual partners house or apartment
04	Family's house or apartment
05	Friend's house or apartment
06	Migrant worker camp
07	Asylum seekers shelter
08	Shelter, welfare residence
09	Work place
10	Rented room (hotel, motel or other rooming house)
11	Car, bus, truck or other vehicle
12	Abandoned Building
13	Deportee shelter/camp
14	Correctional Institution (jail, prison, detention center)
15	Drug treatment center
16	On the Streets, beach, canal
17	Shooting gallery
18	Medical care facility (i.e., hospital, hospice, or nursing home)
19	Other
98	Refuse to Answer

If B33 is equal to 1 and B31A is not equal to 1 or B33 is equal to 2 and B31B is not equal to 1 or B33 is equal to 3 and B31C is not equal to 1 or B33 is equal to 4 and B31D is not equal to 1 or B33 is equal to 5 and B31E is not equal to 1 or B33 is equal to 6 and B31F is not equal to 1 or B33 is equal to 7 and B31G is not equal to 1 or B33 is equal to 8 and B31H is not equal to 1 or B33 is equal to 9 and B31I is not equal to 1 or B33 is equal to 10 and B31J is not equal to 1 or B33 is equal to 11 and B31K is not equal to 1 or B33 is equal to 12 and B31L is not equal to 1 or B33 is equal to 13 and B31M is not equal to 1 or B33 is equal to 14 and B31N is not equal to 1 or B33 is equal to 15 and B31O is not equal to 1 or B33 is equal to 16 and B31P is not equal to 1 or B33 is equal to 17 and B31Q is not equal to 1 or B33 is equal to 18 and B31R is not equal to 1 or B33 is equal to 19 and B31S is not equal to 1 then READ: "Participant could only chooce the most often place out of places named before." and skip to B33.

If B33 is not equal to 19, then skip to B35.

B34. Please specify the place where you slept most often in the past 6 months.

____

B35. Has your housing situation been affected by the COVID/coronavirus pandemic that began in March 2020? (Choose one)

1 Yes

2 No

3 Unsure

8 Refuse to Answer

If B35 is not equal to 1, then skip to B37.

B36. Is your housing situation better or worse since COVID began? (Choose one)

1 Worse

2 Better

8 Refuse to Answer

9 Not Applicable

B37. Approximately, how many people (including yourself) currently live in your household or, if homeless, the place where you stay most often in the last 6 months?
Interviewer: If the participant lives by itself, enter 1.

___ people

998 Refuse to Answer

B38. In the past 6 months, on a typical day, how many hours did you spend on the streets? This includes looking for or dealing drugs, other forms of obtaining money, using drugs, and sleeping on the street.

__ __	hours per day
98	Refuse to Answer

If B16A is equal to 1 or B16J is equal to 1 or B18 is equal to 2 or B18 is equal to 3 or A24 is equal to 1 then skip to B39

If not, then skip to instruction before B41.

B39. Have you ever been a member of the US military?

1	Yes
0	No
8	Refuse to Answer

If B39 is not equal to 1, then skip to instruction before B41.

B40. When did you leave the military?

__ __	YEARS
__ __	MONTHS
98	Refuse to Answer (Years)

If B40 is greater than A1 and B40 is not equal to 98 then READ: "Participant has responded with a length of time greater than his or her age. Please check the responses." and skip to B40.

B41. In the past six months, what were all your sources of income?
Interviewer, please say: Remember your answers are confidential. Read out list and check all that apply. (Check all that apply)

__ A legal job with pay

__ Informal work/odd jobs

__ Military (Army, Marine, Air force)

__ Running a shooting gallery

__ Selling drugs

__ Prostitution /Sex work

__ Sex work (pimp, manager)

__ Other illegal sources of income

__ Family/friends

__ Other

__ None

__ Refuse to Answer

If B41K is equal to 1 and B41A + B41B + B41C + B41D + B41E + B41F + B41G + B41H + B41I + B41J is greater than 0 then READ: "You can only choose the response "none" by itself. Please check responses." and skip to B41.

If B41H is not equal to 1, then skip to instruction before B43.

B42. Please specify other illegal sources of income

____

If B41J is not equal to 1, then skip to instruction before B44.

B43. Please specify other source of income.

____

If B41A + B41B + B41C + B41D + B41E + B41F + B41G + B41H + B41I + B41J + B41K is equal to 1 or B41 is equal to 98, then skip to B47.

B44. In the past year, what was your main source of income? (Choose one)

01	A legal job with pay
02	Informal work/odd jobs
03	Military (Army, Marine, Air force)
04	Selling drugs
05	Running a shooting gallery
06	Prostitution /Sex work
07	Sex work (pimp, manager)
08	Other illegal sources of income
09	Family/friends
10	Other
11	None
98	Refuse to Answer

If B44 is equal to 1 and B41A is not equal to 1 or B44 is equal to 2 and B41B is not equal to 1 or B44 is equal to 3 and B41C is not equal to 1 or B44 is equal to 4 and B41E is not equal to 1 or B44 is equal to 5 and B41D is not equal to 1 or B44 is equal to 6 and B41F is not equal to 1 or B44 is equal to 7 and B41G is not equal to 1 or B44 is equal to 8 and B41H is not equal to 1 or B44 is equal to 9 and B41I is not equal to 1 or B44 is equal to 10 and B41J is not equal to 1 or B44 is equal to 11 and B41K is not equal to 1 then READ: "Participant could only chooce the main source of income out of sources named before." and skip to B44.

If B44 is not equal to 8, then skip to instruction before B46.

B45. Please specify other illegal source of income

____

If B44 is not equal to 10, then skip to B47.

B46. Please specify other source of income

____

B47. Please, select the type of currency you get your income. (Choose one)

1	Pesos
2	Dollars
8	Refuse to Answer

If B47 is not equal to 1, then skip to instruction before B49.

B48. What was your average monthly income over the **past year**, before taxes, including legal and illegal income in pesos? (Choose one)

0	No income
1	Less than 1000 pesos
2	1000-1499 pesos
3	1500-1999 pesos
4	2000-2499 pesos
5	2500-2999 pesos
6	3000-3499 pesos
7	More than 3500 pesos
8	Refuse to Answer

If B47 is not equal to 2, then skip to B50.

B49. What was your average monthly income over the **past year**, before taxes, including legal and illegal income in US Dollars? (Choose one)

00	No income
01	Less than $500 dollars
02	$500-$999 dollars
03	$1000-$1499 dollars
04	$1500-$1999 dollars
05	$2000-$2499 dollars
06	$2500-$2999 dollars
07	$3000-$3499 dollars
08	More than $3500 dollars
98	Refuse to Answer

B50. Has your income level been affected by the COVID/coronavirus pandemic that began in March 2020? (Choose one)

1	Yes
2	No
3	Unsure
8	Refuse to Answer

If B50 is not equal to 1, then skip to instruction before B52.

B51. Is your income level better or worse since COVID began? (Choose one)

1	Worse
2	Better
8	Refuse to Answer
9	Not Applicable

READ: INTERVIEWER READ TO THE RESPONDENT: I am going to ask you about the location of where you live, work, and inject. These questions are meant to help plan the location of health services. This information will not be linked to any of your personal information, like your name.
INTERVIEWER: HAVE THE SAN DIEGO THOMAS BOOK OR T MAPS FOR TIJUANA READY AND AVAILABLE TO SHOW PARTICIPANT AND TOGETHER POINT TO THE NEIGHBORHOOD

B52. What is the name of the neighborhood where you currently live?

—

B53. What is the name of the neighborhood where you **Most Often** work/ earn money?

—

B54. In the **past 6 months**, in which neighborhood did you buy or obtain drugs **Most Often**?

—

B55. In the **past 6 months**, in which neighborhood did you inject drugs the **Most Often**?

—

READ: INTERVIEWER READ TO THE RESPONDENT: Now I am going to ask you about some of the places you may have lived.

If A7 is equal to 2 then skip to B56

If not, then skip to instruction before B60.

B56. Have you lived in Tijuana your whole life?

1	Yes
0	No
8	Refuse to Answer

If B56 is equal to 1, then skip to instruction before B60.

B57. How long have you lived in Tijuana?

__ __	YEARS
__ __	MONTHS
__ __	WEEKS
98	Refuse to Answer (Years)

If B57 is greater than A1 and B57 is not equal to 98 then READ: "Participant has responded with a length of time greater than his or her age. Please check the responses." and skip to B57.

B58. What was the main reason that you come to Tijuana? (Choose one)

0	Looking for work/better life in Tijuana
1	Family moved here
2	Job relocation/transfer
3	To be close to relative
4	I was deported to Tijuana
5	Other
8	Refuse to Answer

If B58 is not equal to 5, then skip to instruction before B60.

B59. Please specify the reason why you came to Tijuana.

____

If A7 is equal to 1 then skip to B60

If not, then skip to instruction before B64.

B60. Have you lived in San Diego County your whole life?

1	Yes
0	No
8	Refuse to Answer

If B60 is equal to 1, then skip to instruction before B64.

B61. How long have you lived in San Diego County?

__ __	YEARS
__ __	MONTHS
__ __	WEEKS
98	Refuse to Answer (Years)

If B61 is greater than A1 and B61 is not equal to 98 then READ: "Participant has responded with a length of time greater than his or her age. Please check the responses." and skip to B61.

B62. What was the main reason that you moved to San Diego County? (Choose one)

0 Looking for work/better life in San Diego

1 Family moved here

2 Job relocation/transfer

3 To be close to relative

4 Other

8 Refuse to Answer

If B62 is not equal to 4, then skip to instruction before B64.

B63. Please specify the reason why you moved to San Diego County.

____

DRUG USE

READ: INTERVIEWER READ TO THE RESPONDENT: Now I will ask you some questions about your alcohol and drug use. Some of these questions will ask you for details that can be difficult to remember. Please do your best to give an answer even if it is your best guess. Recall that we will not share your answers with the police or anyone else outside of this study.

B64. How often do you have a drink containing alcohol? (Choose one)

0	Never
1	Monthly or less
2	2-4 times a month
3	2-3 times a week
4	4 or more times a week
8	Refuse to Answer

If B64 is equal to 0 or B64 is equal to 8, then skip to B68.

B65. How many standard drinks do you have on a typical day when you are drinking? (Choose one)

1	1 to 2
2	3 to 4
3	5 to 6
4	7 to 9
5	10 or more
8	Refuse to Answer

If B26 is equal to 2 or B26 is equal to 3 then skip to B66

If not, then skip to instruction before B67.

B66. How often do you have six or more standard drinks on one occasion? (Choose one)

0 Never
1 Less than monthly
2 Monthly
3 Weekly
4 Daily or almost daily
8 Refuse to Answer

If B26 is equal to 1 then skip to B67

If not, then skip to B68.

B67. How often do you have eight or more standard drinks on one occasion? (Choose one)

0 Never
1 Less than monthly
2 Monthly
3 Weekly
4 Daily or almost daily
8 Refuse to Answer

B68. Have you ever smoked cigarettes?

1 Yes
0 No
8 Refuse to Answer

If B68 is not equal to 1, then skip to B73.

B69. Do you smoke cigarettes now, even a puff?

1 Yes
0 No
8 Refuse to Answer

If B69 is equal to 0 then skip to B70

If not, then skip to B71.

B70. About how long has it been since you have smoked cigarettes?
Interviewer: Enter 0 if the response is Less than one year.

__ __	YEARS
98	Refuse to Answer (Years)

If B70 is greater than A1 and B70 is not equal to 98 then READ: "That response exceeds the participant's current age. Please check the response." and skip to B70.

B71. How old were you when you first started smoking cigarettes?

__ __	Years Old
98	Refuse to Answer

If B71 is greater than A1 and B71 is not equal to 98 then READ: "That response exceeds the participant's current age. Please check the response." and skip to B71.

If B69 is equal to 1 then skip to B72

If not, then skip to B73.

B72. How many cigarettes per day do you smoke?
Interviewer: There are 20 cigarettes in a pack

__ __ __	cigarettes per day
998	Refuse to Answer

B73. How old were you the first time you used an illegal drug?

__ __	years old
98	Refuse to Answer

If B73 is greater than A1 then READ: "That response exceeds the participant's current age. Please check the response." and skip to B73.

B74. What drug did you use the first time you used an illegal drug? (Check all that apply)

__ Black tar/Heroin by itself

__ Powder heroin by itself

__ Cocaine (powder) by itself

__ Heroin and cocaine together (speedball)

__ Methamphetamine/crystal

__ Crack by itself

__ China White by itself

__ OxyContin, without a prescription

__ Other prescription opioids w/o prescription (Vicodin, Darvon, Percocet)

__ Tranquilizers (Rivotril, Diazepam, Valium, Ativan or Restoril)

__ Barbiturates without a prescription (Amytal, Nembutal, and Seconal)

__ Fentanyl

__ Another drug or combination that was not already mentioned

__ Refuse to Answer

If B74M is not equal to 1, then skip to B76.

B75. Please specify another drug or combination:

____

B76. How old were you when you first injected drugs? __ __ years old

98 Refuse to Answer

If B76 is less than B73 and B73 is not equal to 98 or B76 is greater than A1 and B76 is not equal to 98 then READ: "The response is either smaller than the age when the participant used drugs for the first time or exceeds the participant's age. Please check the response." and skip to B76.

B77. What drug or drug combination did you inject the first time you injected? (Check all that apply)

__ Black tar/Heroin by itself

__ Powder heroin by itself

__ Cocaine (powder) by itself

__ Heroin and cocaine together (speedball)

__ Methamphetamine/crystal

__ Crack by itself

__ China White by itself

__ OxyContin, without a prescription

__ Other prescription opioids w/o prescription (Vicodin, Darvon, Percocet)

__ Tranquilizers (Rivotril, Diazepam, Valium, Ativan or Restoril)

__ Barbiturates without a prescription (Amytal, Nembutal, and Seconal)

__ Fentanyl

__ Another drug or combination that was not already mentioned

__ Refuse to Answer

If B77M is not equal to 1, then skip to B79.

B78. Please specify other drug or combination:

____

B79. Who did you inject with the first time you injected? (Check all that apply)

__ No one (Alone)

__ Friend

__ Spouse

__ Partner

__ Brother

__ Sister

__ Cousin

__ Aunt

__ Uncle

__ Sexual partner other than spouse/partner

__ Acquaintance

__ Stranger

__ Other

__ Refuse to Answer

If B79A is equal to 1 and B79B + B79C + B79D + B79E + B79F + B79G + B79H + B79I + B79J + B79K + B79L + B79M is greater than 0 then READ: "You can only choose the response "alone" by itself. Please check responses." and skip to B79.

If B79M is not equal to 1, then skip to instruction before B81.

B80. Please specify other person:

____

READ: INTERVIEWER READ TO THE RESPONDENT: Now, I am going to ask you about different drugs you have used in [LCOUNTRY], your home country. Pease tell me if you have ever used any of these drugs in the last 6 months. If you were in prison/jail in the past 6 months please think of the time when you've been outside prison/jail to answer these questions.

B81. Have you ever smoked or vaped marijuana or hashish by itself in [LCOUNTRY]?

1	Yes
0	No
8	Refuse to Answer

If B81 is not equal to 1, then skip to B86.

B82. How old were you when you first smoked or vaped marijuana or hashish by itself?

__ __	Years Old
98	Refuse to Answer

If B82 is less than B73 or B82 is greater than A1 and B82 is not equal to 98 then READ: "The response is either smaller than the age when the participant used drugs for the first time or exceeds the participant's age. Please check the response." and skip to B82.

B83. During the last 6 months, on average how often have you smoked marijuana or hashish by itself in [LCOUNTRY]? (Choose one)

00	Never
01	One time per month or less
02	2 to 3 days per month
03	One time per week
04	2 to 3 days per week
05	4 to 6 days per week
06	One time per day
07	2-3 times per day
08	4 times or more per day
98	Refuse to Answer

B84. Have you ever vaped marijuana or hashish by itself?

1	Yes
0	No
8	Refuse to Answer

If B84 is not equal to 1, then skip to B86.

B85. During the last 6 months, on average how often have you vaped marijuana or hashish by itself in [LCOUNTRY]? (Choose one)

00	Never
01	One time per month or less
02	2 to 3 days per month
03	One time per week
04	2 to 3 days per week
05	4 to 6 days per week
06	One time per day
07	2-3 times per day
08	4 times or more per day
98	Refuse to Answer

B86. Have you ever used heroin?

1	Yes
0	No
8	Refuse to Answer

If B86 is not equal to 1, then skip to B100.

B87. Have you ever smoked, snorted, or inhaled heroin?

1	Yes
0	No
8	Refuse to Answer

If B87 is not equal to 1, then skip to B90.

B88. How old were you when you first smoked, snorted, or inhaled heroin?

__ __	Years old
98	Refuse to Answer

If B88 is less than B73 or B88 is greater than A1 and B88 is not equal to 98 then READ: "The response is either smaller than the age when the participant used drugs for the first time or exceeds the participant's age. Please check the response." and skip to B88.

B89. During the last 6 months, on average how often have you smoked, snorted, or inhaled heroin in [LCOUNTRY]? (Choose one)

00	Never
01	One time per month or less
02	2 to 3 days per month
03	One time per week
04	2 to 3 days per week
05	4 to 6 days per week
06	One time per day
07	2-3 times per day
08	4 times or more per day
98	Refuse to Answer

B90. Have you ever injected heroin by itself?

1	Yes
0	No
8	Refuse to Answer

If B90 is not equal to 1, then skip to instruction before B93.

B91. How old were you when you first injected heroin by itself?

__ __	Years Old
98	Refuse to Answer

If B91 is less than B76 or B91 is greater than A1 and B91 is not equal to 98 then READ: "The response is either smaller than the age when the participant inject drugs for the first time or exceeds the participant's age. Please check the response." and skip to B91.

B92. During the last 6 months, on average how often have you injected heroin by itself in [LCOUNTRY]? (Choose one)

00	Never
01	One time per month or less
02	2 to 3 days per month
03	One time per week
04	2 to 3 days per week
05	4 to 6 days per week
06	One time per day
07	2-3 times per day
08	4 times or more per day
98	Refuse to Answer

If (B89 is equal to 0 or B89 is equal to 98 or B89 is equal to "skipped") and (B92 is equal to 0 or B92 is equal to 98 or B92 is equal to "skipped"), then skip to B100.

B93. In the last 6 months, when you used heroin in [LCOUNTRY] what colors did it come in? **Interviewer: Read out the options and check all that apply**
(Check all that apply)

__	Black tar
__	Brown powder
__	White powder
__	Yellow powder
__	Orange powder
__	Other
__	Refuse to Answer

If B93F is not equal to 1, then skip to instruction before B95.

B94. Please, specify the color of the heroin you used.

____

If B92 is equal to 0 or B92 is equal to 98 or B92 is equal to "skipped", then skip to B100.

B95. In the last 6 months, when you injected heroin in [LCOUNTRY], what color was it **most ofte**n? (Choose one)

1 Black tar

2 Brown powder

3 White powder

4 Yellow powder

5 Orange powder

6 Other

8 Refuse to Answer

If B95 is not equal to 6, then skip to instruction before B97.

B96. Please, specify the color of the heroin you used.

____

If B95 is not equal to 4, then skip to instruction before B98.

B97. During the last 6 months when you injected heroin in [LCOUNTRY], on average how often was it ***yellow***? (Choose one)

1 Never

2 Less than half the time

3 About half of the time

4 More than half the time

5 Always

8 Refuse to Answer

If B95 is not equal to 5, then skip to instruction before B99.

B98. During the last 6 months when you injected heroin in [LCOUNTRY], on average how often was it ***orange***? (Choose one)

1 Never
2 Less than half the time
3 About half of the time
4 More than half the time
5 Always
8 Refuse to Answer

If B95 is not equal to 6, then skip to B100.

B99. During the last 6 months when you injected heroin in [LCOUNTRY], on average how often was it [Response to B96]? (Choose one)

1 Never
2 Less than half the time
3 About half of the time
4 More than half the time
5 Always
8 Refuse to Answer

B100. Have you ever used cocaine or crack?

1 Yes
0 No
8 Refuse to Answer

If B100 is not equal to 1, then skip to B111.

B101. Have you ever smoked, snorted, or inhaled crack or powder cocaine?

1 Yes
0 No
8 Refuse to Answer

If B101 is not equal to 1, then skip to B104.

B102. How old were you when you <u>first</u> smoked, snorted, or inhaled crack or powder cocaine?

__ __	Years Old
98	Refuse to Answer

If B102 is less than B73 or B102 is greater than A1 and B102 is not equal to 98 then READ: "The response is either smaller than the age when the participant used drugs for the first time or exceeds the participant's age. Please check the response." and skip to B102.

B103. During the last 6 months, on average how often have you smoked, snorted, or inhaled crack or powder cocaine in [LCOUNTRY]? (Choose one)

00	Never
01	One time per month or less
02	2 to 3 days per month
03	One time per week
04	2 to 3 days per week
05	4 to 6 days per week
06	One time per day
07	2-3 times per day
08	4 times or more per day
98	Refuse to Answer

B104. Have you ever injected cocaine by itself?

1	Yes
0	No
8	Refuse to Answer

If B104 is not equal to 1, then skip to instruction before B107.

B105. How old were you when you <u>first</u> injected cocaine by itself?

__ __	Years Old
98	Refuse to Answer

If B105 is less than B76 or B105 is greater than A1 and B105 is not equal to 98 then READ: "The response is either smaller than the age when the participant inject drugs for the first time or exceeds the participant's age. Please check the response." and skip to B105.

B106. During the last 6 months, on average how often have you injected cocaine by itself in [LCOUNTRY]? (Choose one)

00	Never
01	One time per month or less
02	2 to 3 days per month
03	One time per week
04	2 to 3 days per week
05	4 to 6 days per week
06	One time per day
07	2-3 times per day
08	4 times or more per day
98	Refuse to Answer

If B86 is not equal to 1, then skip to B111.

B107. Have you ever injected heroin/black tar and cocaine together (speedball)?

1	Yes
0	No
8	Refuse to Answer

If B107 is not equal to 1, then skip to B111.

B108. How old were you when you <u>first</u> injected heroin and cocaine together (speedball)?

__ __	Years Old
98	Refuse to Answer

If B108 is less than B76 or B108 is greater than A1 and B108 is not equal to 98 then READ: "The response is either smaller than the age when the participant inject drugs for the first time or exceeds the participant's age. Please check the response." and skip to B108.

B109. During the last 6 months, on average how often have you injected heroin and cocaine together (speedball) in [LCOUNTRY]? (Choose one)

00	Never
01	One time per month or less
02	2 to 3 days per month
03	One time per week
04	2 to 3 days per week
05	4 to 6 days per week
06	One time per day
07	2-3 times per day
08	4 times or more per day
98	Refuse to Answer

If B109 is equal to 0 or B109 is equal to 98, then skip to B111.

B110. Over the last 6 months, how many times a day on average did you inject heroin and cocaine together (speedball)?
Interviewer: If the participant says Do not Know ask them for their best guess or estimate

__ __	times a day
97	Don't Know
98	Refuse to Answer

B111. Have you ever used methamphetamine/crystal?

1	Yes
0	No
8	Refuse to Answer

If B111 is not equal to 1, then skip to B128.

B112. Have you ever smoked, snorted, inhaled, or vaped meth/crystal by itself?

1	Yes
0	No
8	Refuse to Answer

If B112 is not equal to 1, then skip to B115.

B113. How old were you when you first smoked, snorted, inhaled, or vaped meth/crystal?

__ __	Years Old
98	Refuse to Answer

If B113 is less than B73 or B113 is greater than A1 and B113 is not equal to 98 then READ: "The response is either smaller than the age when the participant used drugs for the first time or exceeds the participant's age. Please check the response." and skip to B113.

B114. During the last 6 months, on average how often have you smoked, snorted, inhaled, or vaped meth/crystal in [LCOUNTRY]? (Choose one)

00	Never
01	One time per month or less
02	2 to 3 days per month
03	One time per week
04	2 to 3 days per week
05	4 to 6 days per week
06	One time per day
07	2-3 times per day
08	4 times or more per day
98	Refuse to Answer

B115. Have you ever injected meth/crystal by itself?

1	Yes
0	No
8	Refuse to Answer

If B115 is not equal to 1, then skip to instruction before B122.

B116. How old were you when you first injected meth/crystal by itself?

__ __	Years Old
98	Refuse to Answer

If B116 is less than B76 or B116 is greater than A1 and B116 is not equal to 98 then READ: "The response is either smaller than the age when the participant inject drugs for the first time or exceeds the participant's age. Please check the response." and skip to B116.

B117. During the last 6 months, on average how often have you injected meth/crystal by itself in [LCOUNTRY]? (Choose one)

00	Never
01	One time per month or less
02	2 to 3 days per month
03	One time per week
04	2 to 3 days per week
05	4 to 6 days per week
06	One time per day
07	2-3 times per day
08	4 times or more per day
98	Refuse to Answer

If B117 is equal to 0 or B117 is equal to 98, then skip to instruction before B122.

B118. In the last 6 months when you injected methamphetamine (by itself or in combination with other drugs) in [LCOUNTRY], what colors was it? (Check all that apply)

__	White
__	Pink
__	Green
__	Black
__	Yellow
__	Clear (ice)
__	Other
__	Refuse to Answer

If B118G is not equal to 1, then skip to instruction before B120.

B119. Please, specify the color of methamphetamine

____

If B118A + B118B + B118C + B118D + B118E + B118F + B118G is equal to 1 or B118 is equal to 8, then skip to instruction before B122.

B120. In the last 6 months when you injected methamphetamine in [LCOUNTRY], what colors was it most often? (Choose one)

1 White

2 Pink

3 Green

4 Black

5 Yellow

6 Clear (ice)

7 Other

8 Refuse to Answer

If B120 is equal to 1 and B118A is not equal to 1 or B120 is equal to 2 and B118B is not equal to 1 or B120 is equal to 3 and B118C is not equal to 1 or B120 is equal to 4 and B118D is not equal to 1 or B120 is equal to 5 and B118E is not equal to 1 or B120 is equal to 6 and B118F is not equal to 1 or B120 is equal to 7 and B118G is not equal to 1 then READ: "Participant could only chooce the most often color out of colors named before." and skip to B120.

If B120 is not equal to 7, then skip to instruction before B122.

B121. Please, specify the color of methamphetamine

____

If B100 is not equal to 1, then skip to instruction before B125.

B122. Have you ever injected meth/crystal and cocaine together?

1 Yes

0 No

8 Refuse to Answer

If B122 is not equal to 1, then skip to instruction before B125.

B123. How old were you when you first injected meth/crystal and cocaine together?

__ __	Years Old
98	Refuse to Answer

If B123 is less than B76 or B123 is greater than A1 and B123 is not equal to 98 then READ: "The response is either smaller than the age when the participant inject drugs for the first time or exceeds the participant's age. Please check the response." and skip to B123.

B124. During the last 6 months, on average how often have you injected meth/crystal and cocaine together in [LCOUNTRY]? (Choose one)

00	Never
01	One time per month or less
02	2 to 3 days per month
03	One time per week
04	2 to 3 days per week
05	4 to 6 days per week
06	One time per day
07	2-3 times per day
08	4 times or more per day
98	Refuse to Answer

If B86 is not equal to 1, then skip to B128.

B125. Have you ever injected meth/crystal and heroin/black tar together?

1	Yes
0	No
8	Refuse to Answer

If B125 is not equal to 1, then skip to B128.

B126. How old were you when you first injected meth/crystal and heroin together?

__ __	Years Old
98	Refuse to Answer

If B126 is less than B76 or B126 is greater than A1 and B126 is not equal to 98 then READ: "The response is either smaller than the age when the participant inject drugs for the first time or exceeds the participant's age. Please check the response." and skip to B126.

B127. During the last 6 months, on average how often have you injected meth/crystal and heroin together in [LCOUNTRY]? (Choose one)

00	Never
01	One time per month or less
02	2 to 3 days per month
03	One time per week
04	2 to 3 days per week
05	4 to 6 days per week
06	One time per day
07	2-3 times per day
08	4 times or more per day
98	Refuse to Answer

B128. What type(s) of drugs do you think are in China White? (Check all that apply)

__ Heroin

__ Fentanyl

__ Oxycontin

__ Prescription opioids (Vicodin, Darvon, Percocet)

__ Methamphetamine

__ Cocaine

__ Crocodile

__ Barbiturates

__ Cutting agents (e.g., baking soda, baby powder, powdered milk, caffeine, etc)

__ Poison

__ Other

__ Don't Know

__ Refuse to Answer

If B128K is not equal to 1, then skip to instruction before B130.

B129. Please, specify what type of drug or combination do you think China White is.

____

If B128B is equal to 1, then skip to B140.

B130. Have you ever used China White?

1 Yes

0 No

8 Refuse to Answer

If B130 is not equal to 1, then skip to B140.

B131. Have you ever smoked, snorted, inhaled or vaped China White?

1 Yes

0 No

8 Refuse to Answer

If B131 is not equal to 1, then skip to B134.

B132. How old were you when you first smoked, snorted, inhaled or vaped China White?

__ __ Years Old

98 Refuse to Answer

If B132 is less than B73 or B132 is greater than A1 and B132 is not equal to 98 then READ: "The response is either smaller than the age when the participant used drugs for the first time or exceeds the participant's age. Please check the response." and skip to B132.

B133. During the last 6 months, on average how often have you smoked, snorted, inhaled Chine White in [LCOUNTRY]? (Choose one)

00 Never

01 One time per month or less

02 2 to 3 days per month

03 One time per week

04 2 to 3 days per week

05 4 to 6 days per week

06 One time per day

07 2-3 times per day

08 4 times or more per day

98 Refuse to Answer

B134. Have you ever injected China White by itself?

1 Yes

0 No

8 Refuse to Answer

If B134 is not equal to 1, then skip to instruction before B137.

B135. How old were you when you <u>first</u> injected China White by itself? __ __ Years Old

98 Refuse to Answer

If B135 is less than B76 or B135 is greater than A1 and B135 is not equal to 98 then READ: "The response is either smaller than the age when the participant inject drugs for the first time or exceeds the participant's age. Please check the response." and skip to B135.

B136. During the last 6 months, on average how often have you injected Chine White by itself in [LCOUNTRY]? (Choose one)

00 Never

01 One time per month or less

02 2 to 3 days per month

03 One time per week

04 2 to 3 days per week

05 4 to 6 days per week

06 One time per day

07 2-3 times per day

08 4 times or more per day

98 Refuse to Answer

If B111 is not equal to 1, then skip to B140.

B137. Have you ever injected China White and meth/crystal together? 1 Yes

0 No

8 Refuse to Answer

If B137 is not equal to 1, then skip to B140.

B138. How old were you when you <u>first</u> injected China White and meth/crystal together?

__ __ Years Old

98 Refuse to Answer

If B138 is less than B76 or B138 is greater than A1 and B138 is not equal to 98 then READ: "The response is either smaller than the age when the participant inject drugs for the first time or exceeds the participant's age. Please check the response." and skip to B138.

B139. During the last 6 months, on average how often have you injected China White and Meth/Crystal together in [LCOUNTRY]? (Choose one)

00	Never
01	One time per month or less
02	2 to 3 days per month
03	One time per week
04	2 to 3 days per week
05	4 to 6 days per week
06	One time per day
07	2-3 times per day
08	4 times or more per day
98	Refuse to Answer

B140. Have you ever used Fentanyl?

1	Yes
0	No
7	Don't Know
8	Refuse to Answer

If B140 is not equal to 1, then skip to B154.

B141. Have you ever smoked, inhaled snorted or vaped Fentanyl ?

1	Yes
0	No
8	Refuse to Answer

If B141 is not equal to 1, then skip to B144.

B142. How old were you when you first smoked, inhaled snorted or vaped Fentanyl?

__ __	Years old
98	Refuse to Answer

If B142 is less than B73 or B142 is greater than A1 and B142 is not equal to 98 then READ: "The response is either smaller than the age when the participant used drugs for the first time or exceeds the participant's age. Please check the response." and skip to B142.

B143. During the last 6 months, on average how often have you smoked, inhaled snorted or vaped Fentanyl in [LCOUNTRY]? (Choose one)

00	Never
01	One time per month or less
02	2 to 3 days per month
03	One time per week
04	2 to 3 days per week
05	4 to 6 days per week
06	One time per day
07	2-3 times per day
08	4 times or more per day
98	Refuse to Answer

B144. Have you ever injected Fentanyl by itself?

1	Yes
0	No
8	Refuse to Answer

If B144 is not equal to 1, then skip to instruction before B151.

B145. How old were you when you first injected Fentanyl by itself?

__ __	Years Old
98	Refuse to Answer

If B145 is less than B76 or B145 is greater than A1 and B145 is not equal to 98 then READ: "The response is either smaller than the age when the participant inject drugs for the first time or exceeds the participant's age. Please check the response." and skip to B145.

B146. During the last 6 months, on average how often have you injected Fentalyl by itself in [LCOUNTRY]? (Choose one)

00	Never
01	One time per month or less
02	2 to 3 days per month
03	One time per week
04	2 to 3 days per week
05	4 to 6 days per week
06	One time per day
07	2-3 times per day
08	4 times or more per day
98	Refuse to Answer

If B146 is equal to 0 or B146 is equal to "skipped", then skip to instruction before B151.

B147. In the last 6 months when you injected Fentanyl (by itself or in combination with other drugs) in [LCOUNTRY], what colors was it? (Check all that apply)

__	White
__	Yellow
__	Orange
__	Brown
__	Blue
__	Other
__	Refuse to Answer

If B147F is not equal to 1, then skip to instruction before B149.

B148. Please, specify the color of Fentanyl

____

If B147A + B147B + B147C + B147D + B147E + B147F is equal to 1 or B147 is equal to 8, then skip to instruction before B151.

B149. In the last 6 months when you injected Fentanyl in [LCOUNTRY], what colors was it most often? (Choose one)

1	White
2	Yellow
3	Orange
4	Brown
5	Blue
6	Other
8	Refuse to Answer

If B149 is equal to 1 and B147A is not equal to 1 or B149 is equal to 2 and B147B is not equal to 1 or B149 is equal to 3 and B147C is not equal to 1 or B149 is equal to 4 and B147D is not equal to 1 or B149 is equal to 5 and B147E is not equal to 1 or B149 is equal to 6 and B147F is not equal to 1 then READ: "Participant could only chooce the most often color out of colors named before." and skip to B149.

If B149 is not equal to 6, then skip to instruction before B151.

B150. Please, specify the color of Fentanyl

____

If B111 is not equal to 1, then skip to B154.

B151. Have you ever injected fentanyl and meth/crystal together?

1	Yes
0	No
8	Refuse to Answer

If B151 is not equal to 1, then skip to B154.

B152. How old were you when you <u>first</u> injected fentanyl and meth/crystal together?

__ __	Years Old
98	Refuse to Answer

If B152 is less than B76 or B152 is greater than A1 and B152 is not equal to 98 then READ: "The response is either smaller than the age when the participant inject drugs for the first time or exceeds the participant's age. Please check the response." and skip to B152.

B153. During the last 6 months, on average how often have you injected fentanyl and meth/crystal together in [LCOUNTRY]? (Choose one)

00	Never
01	One time per month or less
02	2 to 3 days per month
03	One time per week
04	2 to 3 days per week
05	4 to 6 days per week
06	One time per day
07	2-3 times per day
08	4 times or more per day
98	Refuse to Answer

B154. Have you ever used PCP/Angel Dust?

1	Yes
0	No
8	Refuse to Answer

If B154 is not equal to 1, then skip to B158.

B155. Have you ever smoked or inhaled PCP/Angel Dust?

1	Yes
0	No
8	Refuse to Answer

If B155 is not equal to 1, then skip to B158.

B156. How old were you when you first smoked or inhaled PCP/Angel Dust?

__ __	Years Old
98	Refuse to Answer

If B156 is less than B73 or B156 is greater than A1 and B156 is not equal to 98 then READ: "The response is either smaller than the age when the participant used drugs for the first time or exceeds the participant's age. Please check the response." and skip to B156.

B157. During the last 6 months, on average how often have you smoked or inhaled PCP/Angel Dust in [LCOUNTRY]? (Choose one)

00	Never
01	One time per month or less
02	2 to 3 days per month
03	One time per week
04	2 to 3 days per week
05	4 to 6 days per week
06	One time per day
07	2-3 times per day
08	4 times or more per day
98	Refuse to Answer

B158. Have you ever used Ecstasy?

1	Yes
0	No
8	Refuse to Answer

If B158 is not equal to 1, then skip to B166.

B159. How old were you when you first used Ecstasy?

__ __	Years Old
98	Refuse to Answer

If B159 is less than B73 or B159 is greater than A1 and B159 is not equal to 98 then READ: "The response is either smaller than the age when the participant used drugs for the first time or exceeds the participant's age. Please check the response." and skip to B159.

B160. Have you ever injected Ecstasy?

1	Yes
0	No
8	Refuse to Answer

If B160 is not equal to 1, then skip to B162.

B161. How old were you when you first injected Ecstasy?

__ __	Years Old
98	Refuse to Answer

If B161 is less than B76 or B161 is greater than A1 and B161 is not equal to 98 then READ: "The response is either smaller than the age when the participant inject drugs for the first time or exceeds the participant's age. Please check the response." and skip to B161.

B162. During the last 6 months if you used ecstasy in [LCOUNTRY], how did you use it?
Interviewer: If the participant indicates that they did not use ecstasy in [LCOUNTRY] in the past 6 months, then check Not Applicable (Check all that apply)

__	Injecting
__	Smoking/chasing
__	Snorting
__	Inhaling
__	Swallowing
__	Rectally/bumping
__	Other
__	Not Applicable
__	Refuse to Answer

If B162 is greater than 1 and B162H is equal to 1 then READ: "You can only choose the response "Not Applicable" by itself. Please check responses." and skip to B162.

If B162G is not equal to 1, then skip to instruction before B164.

B163. Specify what other way you used Ecstasy in the last 6 months.

____

If B162A is not equal to 1, then skip to instruction before B165.

B164. During the last 6 months when you used ecstasy in the US, on average how often did you inject it in [LCOUNTRY]? (Choose one)

00	Never
01	One time per month or less
02	2 to 3 days per month
03	One time per week
04	2 to 3 days per week
05	4 to 6 days per week
06	One time per day
07	2-3 times per day
08	4 times or more per day
98	Refuse to Answer

If B162H is equal to 1 or B162 is equal to 9, then skip to B166.

B165. During the last 6 months when you used ecstasy in the US, on average how often did you use it in other ways (not injecting) in [LCOUNTRY]? (Choose one)

00	Never
01	One time per month or less
02	2 to 3 days per month
03	One time per week
04	2 to 3 days per week
05	4 to 6 days per week
06	One time per day
07	2-3 times per day
08	4 times or more per day
98	Refuse to Answer

B166. Have you ever used Ketamine (Special K, K)?	1	Yes
	0	No
	8	Refuse to Answer

If B166 is not equal to 1, then skip to B174.

B167. How old were you when you <u>first</u> used Ketamine?	__ __	Years Old
	98	Refuse to Answer

If B167 is less than B73 or B167 is greater than A1 and B167 is not equal to 98 then READ: "The response is either smaller than the age when the participant used drugs for the first time or exceeds the participant's age. Please check the response." and skip to B167.

B168. Have you ever injected Ketamine?	1	Yes
	0	No
	8	Refuse to Answer

If B168 is not equal to 1, then skip to B170.

B169. How old were you when you <u>first</u> injected Ketamine?	__ __	Years Old
	98	Refuse to Answer

If B169 is less than B76 or B169 is greater than A1 and B169 is not equal to 98 then READ: "The response is either smaller than the age when the participant inject drugs for the first time or exceeds the participant's age. Please check the response." and skip to B169.

B170. If you used ketamine in the last 6 months in [LCOUNTRY], how did you use it?
Interviewer: If the participant indicates that they did not use ketamine in [LCOUNTRY] in the past 6 months, then check Not Applicable (Check all that apply)

__ Injecting

__ Smoking/chasing

__ Snorting

__ Inhaling

__ Swallowing

__ Rectally/bumping

__ Other

__ Not Applicable

__ Refuse to Answer

If B170 is greater than 1 and B170H is equal to 1 then READ: "You can only choose the response "Not Applicable" by itself. Please check responses." and skip to B170.

If B170G is not equal to 1, then skip to instruction before B172.

B171. Please, specify what other way you used Ketamine in the last 6 months.

____

If B170A is not equal to 1, then skip to instruction before B173.

B172. During the last 6 months, on average how often have you injected Ketamine in [LCOUNTRY]? (Choose one)

00	Never
01	One time per month or less
02	2 to 3 days per month
03	One time per week
04	2 to 3 days per week
05	4 to 6 days per week
06	One time per day
07	2-3 times per day
08	4 times or more per day
98	Refuse to Answer
09	Not Applicable

If B170H is equal to 1 or B170 is equal to 98, then skip to B174.

B173. During the last 6 months when you used Ketamine in the US, on average how often did you use it in other ways (not injecting) in [LCOUNTRY]? (Choose one)

00	Never
01	One time per month or less
02	2 to 3 days per month
03	One time per week
04	2 to 3 days per week
05	4 to 6 days per week
06	One time per day
07	2-3 times per day
08	4 times or more per day
98	Refuse to Answer
09	Not Applicable

B174. Have you ever used OxyContin?

1	Yes
0	No
8	Refuse to Answer

If B174 is not equal to 1, then skip to B182.

B175. How old were you when you <u>first</u> used OxyContin?

__ __	Years Old
98	Refuse to Answer

If B175 is less than B73 or B175 is greater than A1 and B175 is not equal to 98 then READ: "The response is either smaller than the age when the participant used drugs for the first time or exceeds the participant's age. Please check the response." and skip to B175.

B176. Have you ever injected OxyContin?

1	Yes
0	No
8	Refuse to Answer

If B176 is not equal to 1, then skip to B178.

B177. How old were you when you <u>first</u> injected OxyContin? __ __ Years Old

98 Refuse to Answer

If B177 is less than B76 or B177 is greater than A1 and B177 is not equal to 98 then READ: "The response is either smaller than the age when the participant inject drugs for the first time or exceeds the participant's age. Please check the response." and skip to B177.

B178. If you used OxyContin in the last 6 months in [LCOUNTRY], how did you use it?
Interviewer: If the participant indicates that they did not use OxyContin in [LCOUNTRY] in the past 6 months, then check Not Applicable (Check all that apply)

__ Injecting

__ Smoking/chasing

__ Snorting

__ Inhaling

__ Swallowing

__ Rectally/bumping

__ Other

__ Not Applicable

__ Refuse to Answer

If B178 is greater than 1 and B178H is equal to 1 then READ: "You can only choose the response "Not Applicable" by itself. Please check responses." and skip to B178.

If B178G is not equal to 1, then skip to instruction before B180.

B179. Please, specify what other way you used OxyContin in the last 6 months.

____

If B178A is not equal to 1, then skip to instruction before B181.

B180. During the last 6 months, on average how often have you injected OxyContin in [LCOUNTRY]? (Choose one)

00	Never
01	One time per month or less
02	2 to 3 days per month
03	One time per week
04	2 to 3 days per week
05	4 to 6 days per week
06	One time per day
07	2-3 times per day
08	4 times or more per day
98	Refuse to Answer
09	Not Applicable

If B178H is equal to 1 or B178 is equal to 98, then skip to B182.

B181. During the last 6 months, on average how often have you used (not injecting) OxyContin in [LCOUNTRY]? (Choose one)

00	Never
01	One time per month or less
02	2 to 3 days per month
03	One time per week
04	2 to 3 days per week
05	4 to 6 days per week
06	One time per day
07	2-3 times per day
08	4 times or more per day
98	Refuse to Answer
09	Not Applicable

B182. Have you ever used other prescription opioids (Vicodin, Darvon, Percocet)?

1	Yes
0	No
8	Refuse to Answer

If B182 is not equal to 1, then skip to B190.

B183. How old were you when you first used other prescription opioids (Vicodin, Darvon, Percocet)?

__ __	Years Old
98	Refuse to Answer

If B183 is less than B73 or B183 is greater than A1 and B183 is not equal to 98 then READ: "The response is either smaller than the age when the participant used drugs for the first time or exceeds the participant's age. Please check the response." and skip to B183.

B184. Have you ever injected other prescription opioids (Vicodin, Darvon, Percocet) ?

1 Yes

0 No

8 Refuse to Answer

If B184 is not equal to 1, then skip to B186.

B185. How old were you when you <u>first</u> injected other prescription opioids (Vicodin, Darvon, Percocet) ?

__ __ Years Old

98 Refuse to Answer

If B185 is less than B76 or B185 is greater than A1 and B185 is not equal to 98 then READ: "The response is either smaller than the age when the participant inject drugs for the first time or exceeds the participant's age. Please check the response." and skip to B185.

B186. If you used other prescription opioids (Vicodin, Darvon, Percocet) in the last 6 months in [LCOUNTRY], how did you use it?
Interviewer: If the participant indicates that they did not use other prescription opioids in [LCOUNTRY] in the past 6 months, then check Not Applicable (Check all that apply)

__ Injecting

__ Smoking/chasing

__ Snorting

__ Inhaling

__ Swallowing

__ Rectally/bumping

__ Other

__ Not Applicable

__ Refuse to Answer

If B186 is greater than 1 and B186H is equal to 1 then READ: "You can only choose the response "Not Applicable" by itself. Please check responses." and skip to B186.

If B186G is not equal to 1, then skip to instruction before B188.

B187. Please, specify what other way you used prescription opioids (Vicodin, Darvon, Percocet) in the last 6 months.

____

If B186A is not equal to 1, then skip to instruction before B189.

B188. During the last 6 months, on average how often have you injected other prescription opioids (Vicodin, Darvon, Percocet) in [LCOUNTRY]? (Choose one)

00	Never
01	One time per month or less
02	2 to 3 days per month
03	One time per week
04	2 to 3 days per week
05	4 to 6 days per week
06	One time per day
07	2-3 times per day
08	4 times or more per day
98	Refuse to Answer
09	Not Applicable

If B186H is equal to 1 or B186 is equal to 98, then skip to B190.

B189. During the last 6 months, on average how often have you used (not injecting) other prescription opioids (Vicodin, Darvon, Percocet) in [LCOUNTRY]? (Choose one)

00	Never
01	One time per month or less
02	2 to 3 days per month
03	One time per week
04	2 to 3 days per week
05	4 to 6 days per week
06	One time per day
07	2-3 times per day
08	4 times or more per day
98	Refuse to Answer

B190. Have you ever used Hallucinogenic drugs like acid, LSD, peyote, mescaline, mushrooms?

1	Yes
0	No
8	Refuse to Answer

If B190 is not equal to 1, then skip to B193.

B191. How old were you when you first used Hallucinogenic drugs like acid, LSD, peyote, mescaline, mushrooms?

__ __	Years Old
98	Refuse to Answer

If B191 is less than B73 or B191 is greater than A1 and B191 is not equal to 98 then READ: "The response is either smaller than the age when the participant used drugs for the first time or exceeds the participant's age. Please check the response." and skip to B191.

B192. During the last 6 months, on average how often have you used Hallucinogenic drugs like acid, LSD, peyote, mescaline, mushrooms in [LCOUNTRY]? (Choose one)

00	Never
01	One time per month or less
02	2 to 3 days per month
03	One time per week
04	2 to 3 days per week
05	4 to 6 days per week
06	One time per day
07	2-3 times per day
08	4 times or more per day
98	Refuse to Answer

B193. Have you ever used Inhalants (glue or gasoline)?

1	Yes
0	No
8	Refuse to Answer

If B193 is not equal to 1, then skip to B196.

B194. How old were you when you <u>first</u> used Inhalants (glue or gasoline)?

__ __	Years Old
98	Refuse to Answer

If B194 is less than B73 or B194 is greater than A1 and B194 is not equal to 98 then READ: "The response is either smaller than the age when the participant used drugs for the first time or exceeds the participant's age. Please check the response." and skip to B194.

B195. During the last 6 months, on average how often have you used Inhalants (glue or gasoline) in [LCOUNTRY]? (Choose one)

00	Never
01	One time per month or less
02	2 to 3 days per month
03	One time per week
04	2 to 3 days per week
05	4 to 6 days per week
06	One time per day
07	2-3 times per day
08	4 times or more per day
98	Refuse to Answer

B196. Have you ever used tranquilizers (Diazepam, Valium, Ativan, Restori or Rivotril) ?

1	Yes
0	No
8	Refuse to Answer

If B196 is not equal to 1, then skip to B204.

B197. How old were you when you <u>first</u> used tranquilizers (Diazepam, Valium, Ativan, Restoril or Rivotril)?

__ __	Years Old
98	Refuse to Answer

If B197 is less than B73 or B197 is greater than A1 and B197 is not equal to 98 then READ: "The response is either smaller than the age when the participant used drugs for the first time or exceeds the participant's age. Please check the response." and skip to B197.

B198. Have you ever injected tranquilizers (Diazepam, Valium, Ativan, Restoril, or Rivotril)?

1 Yes

0 No

8 Refuse to Answer

If B198 is not equal to 1, then skip to B200.

B199. How old were you when you first injected tranquilizers (Diazepam, Valium, Ativan, Restoril, or Rivotril)?

__ __ Years Old

98 Refuse to Answer

If B199 is less than B76 or B199 is greater than A1 and B199 is not equal to 98 then READ: "The response is either smaller than the age when the participant inject drugs for the first time or exceeds the participant's age. Please check the response." and skip to B199.

B200. If you used tranquilizers (Diazepam, Valium, Ativan, Restoril or Rivotril) in the last 6 months in [LCOUNTRY], how did you use it?
Interviewer: If the participant indicates that they did not use tranquilizers in [LCOUNTRY] in the past 6 months, then check Not Applicable (Check all that apply)

__ Injecting

__ Smoking/chasing

__ Snorting

__ Inhaling

__ Swallowing

__ Rectally/bumping

__ Other

__ Not Applicable

__ Refuse to Answer

If B200 is greater than 1 and B200H is equal to 1 then READ: "You can only choose the response "Not Applicable" by itself. Please check responses." and skip to B200.

If B200G is not equal to 1, then skip to instruction before B202.

B201. Please, specify what other way you used tranquilizers (Diazepam, Valium, Ativan, Restoril or Rivotril) in the last 6 months.

____

If B200A is not equal to 1, then skip to instruction before B203.

B202. During the last 6 months, on average how often have you injected tranquilizers (Diazepam, Valium, Ativan ,Restoril or Rivotril) in [LCOUNTRY]? (Choose one)

00	Never
01	One time per month or less
02	2 to 3 days per month
03	One time per week
04	2 to 3 days per week
05	4 to 6 days per week
06	One time per day
07	2-3 times per day
08	4 times or more per day
98	Refuse to Answer

If B200H is equal to 1 or B200 is equal to 98, then skip to B204.

B203. During the last 6 months, on average how often have you used (not injecting) tranquilizers (Diazepam, Valium, Ativan ,Restoril or Rivotril) in [LCOUNTRY]? (Choose one)

00 Never

01 One time per month or less

02 2 to 3 days per month

03 One time per week

04 2 to 3 days per week

05 4 to 6 days per week

06 One time per day

07 2-3 times per day

08 4 times or more per day

98 Refuse to Answer

B204. Have you ever used barbiturates (Amytal, Nembutal, and Seconal)?

1 Yes

0 No

8 Refuse to Answer

If B204 is not equal to 1, then skip to B212.

B205. How old were you when you <u>first</u> used barbiturates (Amytal, Nembutal, and Seconal)?

__ __ Years Old

98 Refuse to Answer

If B205 is less than B73 or B205 is greater than A1 and B205 is not equal to 98 then READ: "The response is either smaller than the age when the participant used drugs for the first time or exceeds the participant's age. Please check the response." and skip to B205.

B206. Have you ever injected barbiturates (Amytal, Nembutal, and Seconal)?

1 Yes

0 No

8 Refuse to Answer

If B206 is not equal to 1, then skip to B208.

B207. How old were you when you first injected barbiturates (Amytal, Nembutal, and Seconal)?

__ __ Years Old

98 Refuse to Answer

If B207 is less than B76 or B207 is greater than A1 and B207 is not equal to 98 then READ: "The response is either smaller than the age when the participant inject drugs for the first time or exceeds the participant's age. Please check the response." and skip to B207.

B208. If you used barbiturates (Amytal, Nembutal, and Seconal) in the last 6 months in [LCOUNTRY], how did you use it?
Interviewer: If the participant indicates that they did not use barbiturates in [LCOUNTRY] in the past 6 months, then check Not Applicable (Check all that apply)

__ Injecting

__ Smoking/chasing

__ Snorting

__ Inhaling

__ Swallowing

__ Rectally/bumping

__ Other

__ Not Applicable

__ Refuse to Answer

If B208 is greater than 1 and B208H is equal to 1 then READ: "You can only choose the response "Not Applicable" by itself. Please check responses." and skip to B208.

If B208G is not equal to 1, then skip to instruction before B210.

B209. Please, specify what other way you used barbiturates (Amytal, Nembutal, and Seconal) in the last 6 months.

____

If B208A is not equal to 1, then skip to instruction before B211.

B210. During the last 6 months, on average how often have you injected barbiturates (Amytal, Nembutal, and Seconal) in [LCOUNTRY]? (Choose one)

00	Never
01	One time per month or less
02	2 to 3 days per month
03	One time per week
04	2 to 3 days per week
05	4 to 6 days per week
06	One time per day
07	2-3 times per day
08	4 times or more per day
98	Refuse to Answer

If B208H is equal to 1 or B208 is equal to 98, then skip to B212.

B211. During the last 6 months, on average how often have you used (not injecting) barbiturates (Amytal, Nembutal, and Seconal) in [LCOUNTRY]? (Choose one)

00	Never
01	One time per month or less
02	2 to 3 days per month
03	One time per week
04	2 to 3 days per week
05	4 to 6 days per week
06	One time per day
07	2-3 times per day
08	4 times or more per day
98	Refuse to Answer

B212. Have you ever used brain pills (Nootropic, modafinil, ritalin)?

1	Yes
0	No
8	Refuse to Answer

If B212 is not equal to 1, then skip to B220.

B213. How old were you when you first used brain pills (Nootropic, modafinil, ritalin)?

__ __	Years Old
98	Refuse to Answer

If B213 is less than B73 or B213 is greater than A1 and B213 is not equal to 98 then READ: "The response is either smaller than the age when the participant used drugs for the first time or exceeds the participant's age. Please check the response." and skip to B213.

B214. Have you ever injected brain pills (Nootropic, modafinil, ritalin)?

1	Yes
0	No
8	Refuse to Answer

If B214 is not equal to 1, then skip to B216.

B215. How old were you when you first injected brain pills (Nootropic, modafinil, ritalin)?

__ __	Years Old
98	Refuse to Answer

If B215 is less than B76 or B215 is greater than A1 and B215 is not equal to 98 then READ: "The response is either smaller than the age when the participant inject drugs for the first time or exceeds the participant's age. Please check the response." and skip to B215.

B216. If you used brain pills (Nootropic, modafinil, ritalin) in the last 6 months in [LCOUNTRY], how did you use it?
Interviewer: If the participant indicates that they did not use brain pills in [LCOUNTRY] in the past 6 months, then check Not Applicable (Check all that apply)

__ Injecting

__ Smoking/chasing

__ Snorting

__ Inhaling

__ Swallowing

__ Rectally/bumping

__ Other

__ Not Applicable

__ Refuse to Answer

If B216 is greater than 1 and B216H is equal to 1 then READ: "You can only choose the response "Not Applicable" by itself. Please check responses." and skip to B216.

If B216G is not equal to 1, then skip to instruction before B218.

B217. Please, specify what other way you used brain pills (Nootropic, modafinil, ritalin) the last 6 months.

____

If B216A is not equal to 1, then skip to instruction before B219.

B218. During the last 6 months, on average how often have you injected brain pills (Nootropic, modafinil, ritalin) in [LCOUNTRY]? (Choose one)

00	Never
01	One time per month or less
02	2 to 3 days per month
03	One time per week
04	2 to 3 days per week
05	4 to 6 days per week
06	One time per day
07	2-3 times per day
08	4 times or more per day
98	Refuse to Answer

If B216H is equal to 1 or B216 is equal to 98, then skip to B220.

B219. During the last 6 months, on average how often did you use (not injecting) brain pills (Nootropic, modafinil, ritalin) in [LCOUNTRY]? (Choose one)

00	Never
01	One time per month or less
02	2 to 3 days per month
03	One time per week
04	2 to 3 days per week
05	4 to 6 days per week
06	One time per day
07	2-3 times per day
08	4 times or more per day
98	Refuse to Answer

B220. Have you ever used another drug by itself (Not Mentioned Earlier)? **Interviewer, please prompt for bath salts.**	1	Yes
	0	No
	8	Refuse to Answer

If B220 is not equal to 1, then skip to B238.

B221. Please specify the other drug that you used by itself?

— —

B222. How old were you when you first used [Response to B221] by itself?	__ __	Years Old
	98	Refuse to Answer

If B222 is less than B73 or B222 is greater than A1 and B222 is not equal to 98 then READ: "The response is either smaller than the age when the participant used drugs for the first time or exceeds the participant's age. Please check the response." and skip to B222.

B223. Have you ever injected [Response to B221]?	1	Yes
	0	No
	8	Refuse to Answer

If B223 is not equal to 1, then skip to B225.

B224. How old were you when you first injected [Response to B221]?	__ __	Years Old
	98	Refuse to Answer

If B224 is less than B76 or B224 is greater than A1 and B224 is not equal to 98 then READ: "The response is either smaller than the age when the participant inject drugs for the first time or exceeds the participant's age. Please check the response." and skip to B224.

B225. If you used [Response to B221] in the last 6 months in [LCOUNTRY], how did you use it?
Interviewer: If the participant indicates that they did not use [Response to B221] **in [LCOUNTRY] in the past 6 months, then check Not Applicable** (Check all that apply)

__ Injecting

__ Smoking/chasing

__ Snorting

__ Inhaling

__ Swallowing

__ Rectally/bumping

__ Other

__ Not Applicable

__ Refuse to Answer

If B225 is greater than 1 and B225H is equal to 1 then READ: "You can only choose the response "Not Applicable" by itself. Please check responses." and skip to B225.

If B225G is not equal to 1, then skip to instruction before B227.

B226. Please, specify what other way you used [Response to B221] in the last 6 months.

____

If B225A is not equal to 1, then skip to instruction before B228.

B227. During the last 6 months, on average how often have you injected [Response to B221] in [LCOUNTRY]? (Choose one)

00	Never
01	One time per month or less
02	2 to 3 days per month
03	One time per week
04	2 to 3 days per week
05	4 to 6 days per week
06	One time per day
07	2-3 times per day
08	4 times or more per day
98	Refuse to Answer

If B225H is equal to 1 or B225 is equal to 98, then skip to B229.

B228. During the last 6 months, on average how often did you use (not injecting) [Response to B221] in [LCOUNTRY]? (Choose one)

00	Never
01	One time per month or less
02	2 to 3 days per month
03	One time per week
04	2 to 3 days per week
05	4 to 6 days per week
06	One time per day
07	2-3 times per day
08	4 times or more per day
98	Refuse to Answer

B229. Have you ever used another drug by itself (Not Mentioned Earlier)?

1 Yes

0 No

8 Refuse to Answer

If B229 is not equal to 1, then skip to B238.

B230. Please specify the other drug that you used by itself?

____

B231. How old were you when you first used [Response to B230] by itself? __ __ Years Old

98 Refuse to Answer

If B231 is less than B73 or B231 is greater than A1 and B231 is not equal to 98 then READ: "The response is either smaller than the age when the participant used drugs for the first time or exceeds the participant's age. Please check the response." and skip to B231.

B232. Have you ever injected [Response to B230]?

1 Yes

0 No

8 Refuse to Answer

If B232 is not equal to 1, then skip to B234.

B233. How old were you when you first injected [Response to B230]? __ __ Years Old

98 Refuse to Answer

If B233 is less than B76 or B233 is greater than A1 and B233 is not equal to 98 then READ: "The response is either smaller than the age when the participant inject drugs for the first time or exceeds the participant's age. Please check the response." and skip to B233.

B234. If you used [Response to B230] in the last 6 months in [LCOUNTRY], how did you use it? **Interviewer: If the participant indicates that they did not use** [Response to B230] **in [LCOUNTRY] in the past 6 months, then check Not Applicable** (Check all that apply)

__ Injecting

__ Smoking/chasing

__ Snorting

__ Inhaling

__ Swallowing

__ Rectally/bumping

__ Other

__ Not Applicable

__ Refuse to Answer

If B234 is greater than 1 and B234H is equal to 1 then READ: "You can only choose the response "Not Applicable" by itself. Please check responses." and skip to B234.

If B234G is not equal to 1, then skip to instruction before B236.

B235. Please, specify what other way you used [Response to B230] in the last 6 months.

____

If B234A is not equal to 1, then skip to instruction before B237.

B236. During the last 6 months, on average how often have you injected [Response to B230] in [LCOUNTRY]? (Choose one)

00	Never
01	One time per month or less
02	2 to 3 days per month
03	One time per week
04	2 to 3 days per week
05	4 to 6 days per week
06	One time per day
07	2-3 times per day
08	4 times or more per day
98	Refuse to Answer

If B234H is equal to 1 or B234 is equal to 98, then skip to B238.

B237. During the last 6 months, on average how often did you (not injecting) use [Response to B230] in [LCOUNTRY]? (Choose one)

00	Never
01	One time per month or less
02	2 to 3 days per month
03	One time per week
04	2 to 3 days per week
05	4 to 6 days per week
06	One time per day
07	2-3 times per day
08	4 times or more per day
98	Refuse to Answer

B238. Have you ever used another drug combination (Not Mentioned Earlier)?

1 Yes

0 No

8 Refuse to Answer

If B238 is not equal to 1, then skip to instruction before B256.

B239. Please specify the other drug combination you used by itself?

____

B240. How old were you when you <u>first</u> used [Response to B239] ?

__ __ Years Old

98 Refuse to Answer

If B240 is less than B73 or B240 is greater than A1 and B240 is not equal to 98 then READ: "The response is either smaller than the age when the participant used drugs for the first time or exceeds the participant's age. Please check the response." and skip to B240.

B241. Have you ever injected [Response to B239]?

1 Yes

0 No

8 Refuse to Answer

If B241 is not equal to 1, then skip to B243.

B242. How old were you when you <u>first</u> injected [Response to B239]?

__ __ Years Old

98 Refuse to Answer

If B242 is less than B76 or B242 is greater than A1 and B242 is not equal to 98 then READ: "The response is either smaller than the age when the participant inject drugs for the first time or exceeds the participant's age. Please check the response." and skip to B242.

B243. If you used [Response to B239] in the last 6 months in [LCOUNTRY], how did you use it?
Interviewer: If the participant indicates that they did not use [Response to B239] **in [LCOUNTRY] in the past 6 months, then check Not Applicable** (Check all that apply)

__ Injecting

__ Smoking/chasing

__ Snorting

__ Inhaling

__ Swallowing

__ Rectally/bumping

__ Other

__ Not Applicable

__ Refuse to Answer

If B243 is greater than 1 and B243H is equal to 1 then READ: "You can only choose the response "Not Applicable" by itself. Please check responses." and skip to B243.

If B243G is not equal to 1, then skip to instruction before B245.

B244. Please, specify what other way you used [Response to B239] in the last 6 months.

____

If B243A is not equal to 1, then skip to instruction before B246.

B245. During the last 6 months, on average how often have you injected [Response to B239] in [LCOUNTRY]? (Choose one)

00	Never
01	One time per month or less
02	2 to 3 days per month
03	One time per week
04	2 to 3 days per week
05	4 to 6 days per week
06	One time per day
07	2-3 times per day
08	4 times or more per day
98	Refuse to Answer

If B243H is equal to 1 or B243 is equal to 98, then skip to B247.

B246. During the last 6 months, on average how often did you use (not injecting) [Response to B239] in [LCOUNTRY]? (Choose one)

00	Never
01	One time per month or less
02	2 to 3 days per month
03	One time per week
04	2 to 3 days per week
05	4 to 6 days per week
06	One time per day
07	2-3 times per day
08	4 times or more per day
98	Refuse to Answer

B247. Have you ever used another drug combination (Not Mentioned Earlier)?

1	Yes
0	No
8	Refuse to Answer

If B247 is not equal to 1, then skip to instruction before B256.

B248. Please specify the other drug combination you used by itself?

____

B249. How old were you when you first used [Response to B248] ?

__ __	Years Old
98	Refuse to Answer

If B249 is less than B73 or B249 is greater than A1 and B249 is not equal to 98 then READ: "The response is either smaller than the age when the participant used drugs for the first time or exceeds the participant's age. Please check the response." and skip to B249.

B250. Have you ever injected [Response to B248]?

1	Yes
0	No
8	Refuse to Answer

If B250 is not equal to 1, then skip to B252.

B251. How old were you when you first injected [Response to B248]?

__ __	Years Old
98	Refuse to Answer

If B251 is less than B76 or B251 is greater than A1 and B251 is not equal to 98 then READ: "The response is either smaller than the age when the participant inject drugs for the first time or exceeds the participant's age. Please check the response." and skip to B251.

B252. If you used [Response to B248] in the last 6 months in [LCOUNTRY], how did you use it?
Interviewer: If the participant indicates that they did not use [Response to B248] **in [LCOUNTRY] in the past 6 months, then check Not Applicable** (Check all that apply)

__ Injecting

__ Smoking/chasing

__ Snorting

__ Inhaling

__ Swallowing

__ Rectally/bumping

__ Other

__ Not Applicable

__ Refuse to Answer

If B252 is greater than 1 and B252H is equal to 1 then READ: "You can only choose the response "Not Applicable" by itself. Please check responses." and skip to B252.

If B252G is not equal to 1, then skip to instruction before B254.

B253. Please, specify what other way you used [Response to B248] in the last 6 months.

____

If B252A is not equal to 1, then skip to instruction before B255.

B254. During the last 6 months, on average how often have you injected [Response to B248] in [LCOUNTRY]? (Choose one)

00	Never
01	One time per month or less
02	2 to 3 days per month
03	One time per week
04	2 to 3 days per week
05	4 to 6 days per week
06	One time per day
07	2-3 times per day
08	4 times or more per day
98	Refuse to Answer

If B252H is equal to 1 or B252 is equal to 98, then skip to instruction before B256.

B255. During the last 6 months, on average how often did you use (not injecting) [Response to B248] in [LCOUNTRY]? (Choose one)

00	Never
01	One time per month or less
02	2 to 3 days per month
03	One time per week
04	2 to 3 days per week
05	4 to 6 days per week
06	One time per day
07	2-3 times per day
08	4 times or more per day
98	Refuse to Answer

ANYINJ6M = If(B92 is not equal to 0 and B92 is not equal to 98 and B92 is not equal to "skipped" or B106 is not equal to 0 and B106 is not equal to 98 and B106 is not equal to "skipped" or B109 is not equal to 0 and B109 is not equal to 98 and B109 is not equal to "skipped" or B117 is not equal to 0 and B117 is not equal to 98 and B117 is not equal to "skipped" or B124 is not equal to 0 and B124 is not equal to 98 and B124 is not equal to "skipped" or B127 is not equal to 0 and B127 is not equal to 98 and B127 is not equal to "skipped" or B136 is not equal to 0 and B136 is not equal to 98 and B136 is not equal to "skipped" or B139 is not equal to 0 and B139 is not equal to 98 and B139 is not equal to "skipped" or B146 is not equal to 0 and B146 is not equal to 98 and B146 is not equal to "skipped" or B153 is not equal to 0 and B153 is not equal to 98 and B153 is not equal to "skipped" or B164 is not equal to 0 and B164 is not equal to 98 and B164 is not equal to "skipped" or B172 is not equal to 0 and B172 is not equal to 98 and B172 is not equal to "skipped" or B180 is not equal to 0 and B180 is not equal to 98 and B180 is not equal to "skipped" or B188 is not equal to 0 and B188 is not equal to 98 and B188 is not equal to "skipped" or B202 is not equal to 0 and B202 is not equal to 98 and B202 is not equal to "skipped" or B210 is not equal to 0 and B210 is not equal to 98 and B210 is not equal to "skipped" or B218 is not equal to 0 and B218 is not equal to 98 and B218 is not equal to "skipped" or B227 is not equal to 0 and B227 is not equal to 98 and B227 is not equal to "skipped" or B236 is not equal to 0 and B236 is not equal to 98 and B236 is not equal to "skipped" or B245 is not equal to 0 and B245 is not equal to 98 and B245 is not equal to "skipped" or B254 is not equal to 0 and B254 is not equal to 98 and B254 is not equal to "skipped", 1, 0)

If ANYINJ6M is equal to 0 then READ: "The summary of responses to drug questions disqualifies participant for enrollment into study. Please check the responses by reviewing each drug question again." and skip to B86.

READ: INTERVIEWER READ TO THE RESPONDENT: The Next Questions have to do with injection and sharing behaviors. I know I asked you before but can you remind me…

B256. In the past 6 months, on average in [LCOUNTRY], how often did you inject (shoot) any drug or drug combinations? (Choose one)

01	One time per month or less
02	2 to 3 days per month
03	One time per week
04	2 to 3 days per week
05	4 to 6 days per week
06	One time per day
07	2-3 times per day
08	4 times or more per day
98	Refuse to Answer

B257. Of the times you injected in the last 6 months in [LCOUNTRY], how often did you give, rent or lend a syringe that you already used to someone else? (Choose one)

1 Never

2 Sometimes

3 About half of the time

4 Often

5 Always

8 Refuse to Answer

B258. Of the times you injected in the last 6 months in [LCOUNTRY], how often did you use a syringe that you knew or suspected that it had been used before by someone else? (Choose one)

1 Never

2 Sometimes

3 About half of the time

4 Often

5 Always

8 Refuse to Answer

If B258 is equal to 1 or B258 is equal to 8, then skip to B261.

B259. The last time you injected drugs in [LCOUNTRY], did you use a syringe that you knew or suspected that it had been used before by someone else?

1 Yes

0 No

8 Refuse to Answer

B260. Of the times you injected with a syringe that had been used by somebody else in the last 6 months in [LCOUNTRY], how often did you clean it with bleach before you used it? (Choose one)

1 Never

2 Less than half the time

3 About half of the time

4 More than half the time

5 Always

8 Refuse to Answer

B261. Of the times you injected with other people in the last 6 months in [LCOUNTRY], how often did you divide up drugs with somebody else by using a syringe? This is also called back loading, piggybacking or splitting drugs wet. (Choose one)

1 Never

2 Less than half the time

3 About half of the time

4 More than half the time

5 Always

8 Refuse to Answer

B262. Of the times you injected in the last 6 months in [LCOUNTRY], how often did you use a cooker, cotton, or water with someone or after someone else used it? (Choose one)

1 Never

2 Less than half the time

3 About half of the time

4 More than half the time

5 Always

8 Refuse to Answer

B263. Of the times you injected in the last 6 months in [LCOUNTRY], how often did you buy drugs that came already prepared in a syringe? (Choose one)

1	Never
2	Less than half the time
3	About half of the time
4	More than half the time
5	Always
8	Refuse to Answer

B264. Of the times you injected in the last 6 months in [LCOUNTRY], how often did you inject with a new, sterile syringe? (Choose one)

1	Never
2	Less than half the time
3	About half of the time
4	More than half the time
5	Always
8	Refuse to Answer

B265. In the last 6 months in [LCOUNTRY], from where did you get the syringes you used to inject drugs? (Check all that apply)

__ Pharmacy

__ Spouse, family member, or sex partner

__ Friend

__ Acquaintance/Stranger

__ Drug Dealer

__ Hit Doctor

__ Shooting gallery

__ Syringe exchange program (you exchanged it yourself)

__ Someone who gets them from a syringe exchange program

__ Bought on the street

__ Doctor/ clinic/ hospital

__ Veterinary clinic/ pet store

__ Market/Store

__ Found on the ground or in the trash

__ Some other place

__ Refuse to Answer

If B265O is equal to 1 then skip to B266

If not, then skip to instruction before B267.

B266. Please specify the "other" place where you got syringes from in the past 6 months.

____

If B265A + B265B + B265C + B265D + B265E + B265F + B265G + B265H + B265I + B265J + B265K + B265L + B265M + B265N + B265O is not equal to 1 then skip to B267

If not, then skip to instruction before B269.

B267. In the last 6 months in [LCOUNTRY], where did you get your syringes most often from? (Choose one)

01	Pharmacy
02	Spouse, family member, or sex partner
03	Friend
04	Acquaintance/Stranger
05	Drug Dealer
06	Hit Doctor
07	Shooting gallery
08	Syringe exchange program (you exchanged it yourself)
09	Someone who gets them from a syringe exchange program
10	Bought on the street
11	Doctor/ clinic/ hospital
12	Veterinary clinic/ pet store
13	Market/Store
14	Found on the ground or in the trash
15	Some other place
98	Refuse to Answer

If B267 is equal to 1 and B265A is not equal to 1 or B267 is equal to 2 and B265B is not equal to 1 or B267 is equal to 3 and B265C is not equal to 1 or B267 is equal to 4 and B265D is not equal to 1 or B267 is equal to 5 and B265E is not equal to 1 or B267 is equal to 6 and B265F is not equal to 1 or B267 is equal to 7 and B265G is not equal to 1 or B267 is equal to 8 and B265H is not equal to 1 or B267 is equal to 9 and B265I is not equal to 1 or B267 is equal to 10 and B265J is not equal to 1 or B267 is equal to 11 and B265K is not equal to 1 or B267 is equal to 12 and B265L is not equal to 1 or B267 is equal to 13 and B265M is not equal to 1 or B267 is equal to 14 and B265N is not equal to 1 or B267 is equal to 15 and B265O is not equal to 1 then READ: "Participant could only chooce the most often place out of places named before." and skip to B267.

If B267 is equal to 15 then skip to B268

If not, then skip to instruction before B269.

B268. Please specify the place where you've got your syringes most often from , in the past 6 months.

____

If B267 is equal to 1 or B267 is equal to 8 then skip to B269

If not, then skip to instruction before B271.

B269. In the last 6 months in [LCOUNTRY], how many sterile, clean syringes per visit did you get from a Needle Exchange Program on average?

___

998 Refuse to Answer

If B269 is equal to 0 or B269 is equal to 998, then skip to instruction before B271.

B270. What was the name of the Needle Exchange Program you got syringes from most often?

____

If B265A is equal to 1 or B267 is equal to 1 then skip to B271

If not, then skip to B273.

B271. In the last 6 months in [LCOUNTRY], how many sterile, clean syringes per visit did you get from a pharmacy on average?

___

998 Refuse to Answer

If B271 is equal to 0 or B271 is equal to 998, then skip to B273.

B272. What was the name of the Pharmacy you bought syringes from most often?

____

B273. In the last 6 months in [LCOUNTRY], how easy or hard was it for you to get new, unused syringes when you injected drugs? (Choose one)

1 Never tried to get new, unused syringes

2 Very easy

3 Easy

4 Hard

5 Very hard

8 Refuse to Answer

If B273 is equal to 4 or B273 is equal to 5 then skip to B274

If not, then skip to instruction before B275.

B274. What was the main reason it was hard to get new, unused syringes in [LCOUNTRY]? (Choose one)

1 They cost too much/ No money to buy them

2 No place to legally buy them / store won't sell to me

3 No syringe exchange program nearby

4 I'm worried about getting caught / arrested by police with syringes

5 Pharmacy/store was closed

6 Other

7 Don't Know

8 Refuse to Answer

If B274 is equal to 6 then skip to B275

If not, then skip to B276.

B275. Please specify the main reason why it was hard to get new/unused syringes.

____

B276. Have you ever sought the help of someone to help you inject drugs, like a hit doctor in [LCOUNTRY]?

1	Yes
0	No
7	Don't Know
8	Refuse to Answer

If B276 is not equal to 1, then skip to instruction before B281.

B277. When was the last time someone helped you inject drugs in [LCOUNTRY]?

__ __ __	MONTHS
__ __ __	WEEKS
__ __ __	DAYS
998	Refuse to Answer (Months)

If B277 is greater than A1 and B277 is not equal to 998 then READ: "The response exceeds the participant's age. Please check the response." and skip to B277.

If B277 is greater than 6 and B277 is not equal to 98, then skip to instruction before B281.

B278. In the last 6 months in [LCOUNTRY] how often has someone else helped you inject? (Choose one)

1	Always
2	Often
3	Sometimes
4	Rarely
8	Refuse to Answer

B279. In the last 6 months when someone helped you inject drugs, what countries did this happen in? (Check all that apply)

__ the US

__ Mexico

__ Other

__ Refuse to Answer

If B279C is not equal to 1, then skip to instruction before B281.

B280. Please specify other country

____

DRUG Market Characteristics

READ: INTERVIEWER READ TO THE RESPONDENT: In the next section we will be talking about the price, purity and availability of drugs on the streets and whether there have been changes in [LCITY]. We will ask you about specific drugs and we will take about prices first and then about purity and finally about their availability. I will ask you about specific drugs and I would like your opinion even if you have not used a specific drug.

B281. Please, select the type of currency you know a drug cost. (Choose one)

1	Pesos
2	US Dollars
8	Refuse to Answer

If B281 is equal to 2 then skip to B282

If not, then skip to instruction before B297.

B282. In US dollars: How much does it cost a dose of black tar/heroin by itself in [LCITY]?

__ __ __	dollars
997	Don't Know
998	Refuse to Answer

B283. In US dollars: How much does it cost a dose of powder heroinr by itself in [LCITY]?

__ __ __	dollars
997	Don't Know
998	Refuse to Answer

B284. In US dollars: How much does it cost a dose of Crack cocaine by itself in [LCITY]?

__ __ __	dollars
997	Don't Know
998	Refuse to Answer

B285. In US dollars: How much does it cost a dose of Cocaine (powder) by itself in [LCITY]?

__ __ __	dollars
997	Don't Know
998	Refuse to Answer

B286. In US dollars: How much does it cost a dose of heroin and cocaine together (speedball) in [LCITY]?

__ __ __	dollars
997	Don't Know
998	Refuse to Answer

B287. In US dollars: How much does it cost a dose of Methamphetamine/crystal in [LCITY]?

__ __ __	dollars
997	Don't Know
998	Refuse to Answer

B288. In US dollars: How much does it cost a dose of China White in [LCITY]?

__ __ __	dollars
997	Don't Know
998	Refuse to Answer

B289. In US dollars: How much does it cost a dose of OxyContin, without a prescription in [LCITY]?

__ __ __	dollars
997	Don't Know
998	Refuse to Answer

B290. In US dollars: How much does it cost a dose of Other prescription opioids w/o prescription (Vicodin, Darvon, Percocet) in [LCITY]?

— — —	dollars
997	Don't Know
998	Refuse to Answer

B291. In US dollars: How much does it cost a dose of Tranquilizers (Rivotril, Diazepam, Valium, Ativan or Restoril) in [LCITY]?

— — —	dollars
997	Don't Know
998	Refuse to Answer

B292. In US dollars: How much does it cost a dose of Barbiturates without a prescription (Amytal, Nembutal, and Seconal) in [LCITY]?

— — —	dollars
997	Don't Know
998	Refuse to Answer

B293. In US dollars: How much does it cost a dose of Fentanyl in [LCITY]?

— — —	dollars
997	Don't Know
998	Refuse to Answer

B294. Do you know the cost of Another drug or combination that was not already mentioned in [LCITY]?

1	Yes
0	No
8	Refuse to Answer

If B294 is not equal to 1, then skip to instruction before B297.

B295. In US dollars: How much does it cost a dose of Another drug or combination that was not already mentioned in [LCITY]?

— — —	dollars
997	Don't Know
998	Refuse to Answer

If B295 is greater than 0 and B295 is not equal to 998 then skip to B296

If not, then skip to instruction before B297.

B296. Another drug or combination you named a cost for earlier.

— —

If B281 is equal to 1 then skip to B297

If not, then skip to B312.

B297. In pesos: How much does it cost a dose of black tar/heroin by itself in [LCITY]?

— — —	pesos
997	Don't Know
998	Refuse to Answer

B298. In pesos: How much does it cost a dose of powder heroin by itself in [LCITY]?

— — —	pesos
997	Don't Know
998	Refuse to Answer

B299. In pesos: How much does it cost a dose of Crack cocaine by itself in [LCITY]?

— — —	pesos
997	Don't Know
998	Refuse to Answer

B300. In pesos: How much does it cost a dose of Cocaine (powder) by itself in [LCITY]?

__ __ __ __	pesos
9997	Don't Know
9998	Refuse to Answer

B301. In pesos: How much does it cost a dose of heroin and cocaine together (speedball) in [LCITY]?

__ __ __ __	pesos
9997	Don't Know
9998	Refuse to Answer

B302. In pesos: How much does it cost a dose of Methamphetamine/crystal by itself in [LCITY]?

__ __ __ __	pesos
9997	Don't Know
9998	Refuse to Answer

B303. In pesos: How much does it cost a dose of China White in [LCITY]?

__ __ __ __	pesos
9997	Don't Know
9998	Refuse to Answer

B304. In pesos: How much does it cost a dose of OxyContin, without a prescription in [LCITY]?

__ __ __ __	pesos
9997	Don't Know
9998	Refuse to Answer

B305. In pesos: How much does it cost a dose of Other prescription opioids w/o prescription (Vicodin, Darvon, Percocet) in [LCITY]?

— — — —	pesos
9997	Don't Know
9998	Refuse to Answer

B306. In pesos: How much does it cost a dose of Tranquilizers (Rivotril, Diazepam, Valium, Ativan or Restoril) in [LCITY]?

— — — —	pesos
9997	Don't Know
9998	Refuse to Answer

B307. In pesos: How much does it cost a dose of Barbiturates without a prescription (Amytal, Nembutal, and Seconal) in [LCITY]?

— — — —	pesos
9997	Don't Know
9998	Refuse to Answer

B308. In pesos: How much does it cost a dose of Fentanyl in [LCITY]?

— — — —	pesos
9997	Don't Know
9998	Refuse to Answer

B309. Do you know the cost in pesos of Another drug or combination that was not already mentioned in [LCITY]?

1	Yes
0	No
8	Refuse to Answer

If B309 is not equal to 1, then skip to B312.

B310. In pesos: How much does it cost a dose of Another drug or combination that was not already mentioned in [LCITY]?

— — — —	pesos
9997	Don't Know
9998	Refuse to Answer

If B310 is greater than 0 and B310 is not equal to 998 then skip to B311

If not, then skip to B312.

B311. Another drug or combination you named a cost for earlier.

— —

B312. Do you think that the price of most street drugs is generally higher or lower in Tijuana compared to San Diego? (Choose one)

1	Higher
2	Lower
3	The same
7	Don't Know
8	Refuse to Answer

B313. Do you think the closure of the Mexico-US border during the COVID pandemic has affected the price of drugs in San Diego?

1	Yes
0	No
7	Don't Know
8	Refuse to Answer

B314. Do you think the closure of the Mexico-US border during the COVID pandemic has affected the price of drugs in Tijuana?

1	Yes
0	No
7	Don't Know
8	Refuse to Answer

B315. In your opinion, in the last 6 months, has the price of heroin increased, decreased or stayed the same in [LCITY]? (Choose one)

1	Increased
2	Decreased
3	Stayed the same
7	Don't Know
8	Refuse to Answer

B316. In your opinion, in the last 6 months, has the price of methamphetamine increased, decreased or stayed the same in [LCITY]? (Choose one)

1	Increased
2	Decreased
3	Stayed the same
7	Don't Know
8	Refuse to Answer

B317. In your opinion, in the last 6 months, has the price of powder cocaine increased, decreased or stayed the same in [LCITY]? (Choose one)

1	Increased
2	Decreased
3	Stayed the same
7	Don't Know
8	Refuse to Answer

B318. In your opinion, in the last 6 months, has the price of crack cocaine increased, decreased or stayed the same in [LCITY]? (Choose one)

1	Increased
2	Decreased
3	Stayed the same
7	Don't Know
8	Refuse to Answer

B319. In your opinion, in the last 6 months, has the price of OxyContin increased, decreased or stayed the same in [LCITY]? (Choose one)

1	Increased
2	Decreased
3	Stayed the same
7	Don't Know
8	Refuse to Answer

B320. In your opinion, in the last 6 months, has the price of Fentanyl increased, decreased or stayed the same in [LCITY]? (Choose one)

1 Increased

2 Decreased

3 Stayed the same

7 Don't Know

8 Refuse to Answer

B321. In your opinion, in the last 6 months, has the price of China White increased, decreased or stayed the same in [LCITY]? (Choose one)

1 Increased

2 Decreased

3 Stayed the same

7 Don't Know

8 Refuse to Answer

B322. In your opinion, in the last 6 months, has the price of other prescription opioids increased, decreased or stayed the same in [LCITY]? (Choose one)

1 Increased

2 Decreased

3 Stayed the same

7 Don't Know

8 Refuse to Answer

READ: INTERVIEWER READ TO THE RESPONDENT: Sometimes the purity of drugs on the street changes. So, I am going to ask you some questions about the changes you may have observed in the purity of drugs in the past 6 months.

B323. In your opinion, in the last 6 months, has the purity of heroin increased, decreased or stayed the same in [LCITY]? (Choose one)

1	Increased
2	Decreased
3	Stayed the same
7	Don't Know
8	Refuse to Answer

B324. In your opinion, in the last 6 months, has the purity of methamphetamine increased, decreased or stayed the same in [LCITY]? (Choose one)

1	Increased
2	Decreased
3	Stayed the same
7	Don't Know
8	Refuse to Answer

B325. In your opinion, in the last 6 months, has the purity of powder cocaine increased, decreased or stayed the same in [LCITY]? (Choose one)

1	Increased
2	Decreased
3	Stayed the same
7	Don't Know
8	Refuse to Answer

B326. In your opinion, in the last 6 months, has the purity of crack cocaine increased, decreased or stayed the same in [LCITY]? (Choose one)

1	Increased
2	Decreased
3	Stayed the same
7	Don't Know
8	Refuse to Answer

B327. In your opinion, in the last 6 months, has the purity of OxyContin increased, decreased or stayed the same in [LCITY]? (Choose one)

1	Increased
2	Decreased
3	Stayed the same
7	Don't Know
8	Refuse to Answer

B328. In your opinion, in the last 6 months, has the purity of Fentanyl increased, decreased or stayed the same in [LCITY]? (Choose one)

1	Increased
2	Decreased
3	Stayed the same
7	Don't Know
8	Refuse to Answer

B329. In your opinion, in the last 6 months, has the purity of China White increased, decreased or stayed the same in [LCITY]? (Choose one)

1	Increased
2	Decreased
3	Stayed the same
7	Don't Know
8	Refuse to Answer

B330. In your opinion, in the last 6 months, has the purity of other prescription opioids increased, decreased or stayed the same in [LCITY]? (Choose one)

1	Increased
2	Decreased
3	Stayed the same
7	Don't Know
8	Refuse to Answer

B331. Do you think the purity of drugs is generally better or worse in Tijuana compared to San Diego? (Choose one)

1	Better
2	Worse
3	The same
7	Don't Know
8	Refuse to Answer

B332. Do you think the closure of the Mexico-US border affects the purity of drugs in San Diego?

1	Yes
0	No
7	Don't Know
8	Refuse to Answer

If B332 is not equal to 1, then skip to B334.

B333. Do you think the closure of the Mexico-US border makes the purity of drugs in San Diego better or worse? (Choose one)

1 Better

2 Worse

3 The same

7 Don't Know

8 Refuse to Answer

B334. Do you think the closure of the Mexico-US border affects the purity of drugs in Tijuana?

1 Yes

0 No

7 Don't Know

8 Refuse to Answer

If B334 is not equal to 1, then skip to instruction before B336.

B335. Do you think the closure of the Mexico-US border makes the purity of drugs in Tijuana better or worse? (Choose one)

1 Better

2 Worse

3 The same

7 Don't Know

8 Refuse to Answer

READ: INTERVIEWER READ TO THE RESPONDENT: Now, Sometimes the availability of drugs on the street changes. So, I am going to ask you some questions about the changes you may have observed in the availability of drugs in the past 6 months. I will ask you about specific drugs and I would like your opinion even if you have not used a specific drug.

B336. In your opinion, in the last 6 months, has the availability of heroin increased, decreased or stayed the same in [LCITY]? (Choose one)

1 Increased

2 Decreased

3 Stayed the same

7 Don't Know

8 Refuse to Answer

B337. In your opinion, in the last 6 months, has the availability of methamphetamine increased, decreased or stayed the same in [LCITY]? (Choose one)

1 Increased

2 Decreased

3 Stayed the same

7 Don't Know

8 Refuse to Answer

B338. In your opinion, in the last 6 months, has the availability of powder cocaine increased, decreased or stayed the same in [LCITY]? (Choose one)

1 Increased

2 Decreased

3 Stayed the same

7 Don't Know

8 Refuse to Answer

B339. In your opinion, in the last 6 months, has the availability of crack cocaine increased, decreased or stayed the same in [LCITY]? (Choose one)

1	Increased
2	Decreased
3	Stayed the same
7	Don't Know
8	Refuse to Answer

B340. In your opinion, in the last 6 months, has the availability of OxyContin increased, decreased or stayed the same in [LCITY]? (Choose one)

1	Increased
2	Decreased
3	Stayed the same
7	Don't Know
8	Refuse to Answer

B341. In your opinion, in the last 6 months, has the availability of Fentanyl increased, decreased or stayed the same in [LCITY]? (Choose one)

1	Increased
2	Decreased
3	Stayed the same
7	Don't Know
8	Refuse to Answer

B342. In your opinion, in the last 6 months, has the availability of China White increased, decreased or stayed the same in [LCITY]? (Choose one)

1	Increased
2	Decreased
3	Stayed the same
7	Don't Know
8	Refuse to Answer

B343. In your opinion, in the last 6 months, has the availability of other prescription opioids increased, decreased or stayed the same in [LCITY]? (Choose one)

1	Increased
2	Decreased
3	Stayed the same
7	Don't Know
8	Refuse to Answer

B344. Do you think the availability of drugs is generally better or worse in Tijuana compared to San Diego? (Choose one)

1	Better
2	Worse
3	The same
7	Don't Know
8	Refuse to Answer

B345. Do you think the closure of the Mexico-US border affects the availability of drugs in San Diego?

1	Yes
0	No
7	Don't Know
8	Refuse to Answer

If B345 is not equal to 1, then skip to B347.

B346. Do you think the closure of the Mexico-US border makes the availability of drugs in San Diego better or worse? (Choose one)

1	Better
2	Worse
3	The same
7	Don't Know
8	Refuse to Answer

B347. Do you think the closure of the Mexico-US border affects the availability of drugs in Tijuana?

1	Yes
0	No
7	Don't Know
8	Refuse to Answer

If B347 is not equal to 1, then skip to B349.

B348. Do you think the closure of the Mexico-US border makes the availability of drugs in Tijuana better or worse? (Choose one)

1	Better
2	Worse
3	The same
7	Don't Know
8	Refuse to Answer

B349. Are there drugs that were available before the COVID pandemic that are no longer sold in **[LCITY]**?

1	Yes
0	No
7	Don't Know
8	Refuse to Answer

If B349 is not equal to 1, then skip to B364.

B350. Is there black tar/heroin by itself that is no longer sold in [LCITY]?

1	Yes
0	No
7	Don't Know
8	Refuse to Answer

B351. Is there Powder heroin by itself that is no longer sold in [LCITY]?

1	Yes
0	No
7	Don't Know
8	Refuse to Answer

B352. Is there Powder Cocaine by itself that is no longer sold in [LCITY]?

1	Yes
0	No
7	Don't Know
8	Refuse to Answer

B353. Are there Heroin and cocaine together (speedball) that are no longer sold in [LCITY]?

1	Yes
0	No
7	Don't Know
8	Refuse to Answer

B354. Is there Methamphetamine/crystal by itself that is no longer sold in [LCITY]?

1 Yes
0 No
7 Don't Know
8 Refuse to Answer

B355. Is there Crack by itself that is no longer sold in [LCITY]?

1 Yes
0 No
7 Don't Know
8 Refuse to Answer

B356. Is there China White that is no longer sold in [LCITY]?

1 Yes
0 No
7 Don't Know
8 Refuse to Answer

B357. Is there OxyContin, without a prescription that is no longer sold in [LCITY]?

1 Yes
0 No
7 Don't Know
8 Refuse to Answer

B358. Are there Other prescription opioids w/o prescription (Vicodin, Darvon, Percocet) that is no longer sold in [LCITY]?

1 Yes
0 No
7 Don't Know
8 Refuse to Answer

B359. Are there Tranquilizers (Rivotril, Diazepam, Valium, Ativan or Restoril) that are no longer sold in [LCITY]?

1 Yes

0 No

7 Don't Know

8 Refuse to Answer

B360. Are there Barbiturates without a prescription (Amytal, Nembutal, and Seconal) that is no longer sold in [LCITY]?

1 Yes

0 No

7 Don't Know

8 Refuse to Answer

B361. Is there Fentanyl that is no longer sold in [LCITY]?

1 Yes

0 No

7 Don't Know

8 Refuse to Answer

B362. Are there Another drug or combination that was not already mentioned that is no longer sold in [LCITY]?

1 Yes

0 No

7 Don't Know

8 Refuse to Answer

If B362 is equal to 1 then skip to B363

If not, then skip to B364.

B363. Please specify the name of Another drug or combination that was not already mentioned.

____

B364. Are there drugs or drug combinations that are available now in **[LCITY]**, that weren't available before the COVID pandemic?

1 Yes

0 No

7 Don't Know

8 Refuse to Answer

If B364 is not equal to 1, then skip to B379.

B365. Is there black tar/heroin by itself available now in [LCITY]?

1 Yes

0 No

7 Don't Know

8 Refuse to Answer

B366. Is there Powder heroin by itself available now in [LCITY]?

1 Yes

0 No

7 Don't Know

8 Refuse to Answer

B367. Is there Powder Cocaine by itself available now in [LCITY]?

1 Yes

0 No

7 Don't Know

8 Refuse to Answer

B368. Are there heroin and cocaine together (speedball) available now in [LCITY]?

1	Yes
0	No
7	Don't Know
8	Refuse to Answer

B369. Is there Methamphetamine/crystal available now in [LCITY]?

1	Yes
0	No
7	Don't Know
8	Refuse to Answer

B370. Is there Crack by itself available now in [LCITY]?

1	Yes
0	No
7	Don't Know
8	Refuse to Answer

B371. Is there China White by itself available now in [LCITY]?

1	Yes
0	No
7	Don't Know
8	Refuse to Answer

B372. Is there OxyContin, without a prescription available now in [LCITY]?

1	Yes
0	No
7	Don't Know
8	Refuse to Answer

B373. Are there other prescription opioids w/o prescription (Vicodin, Darvon, Percocet) available now in [LCITY]?

1 Yes

0 No

7 Don't Know

8 Refuse to Answer

B374. Are there Tranquilizers (Rivotril, Diazepam, Valium, Ativan or Restoril) available now in [LCITY]?

1 Yes

0 No

7 Don't Know

8 Refuse to Answer

B375. Are there Barbiturates without a prescription (Amytal, Nembutal, and Seconal) available now in [LCITY]?

1 Yes

0 No

7 Don't Know

8 Refuse to Answer

B376. Is there Fentanyl available now in [LCITY]?

1 Yes

0 No

7 Don't Know

8 Refuse to Answer

B377. Another drug or combination that was not already mentioned available now in [LCITY]?

1	Yes
0	No
7	Don't Know
8	Refuse to Answer

If B377 is equal to 1 then skip to B378

If not, then skip to B379.

B378. Please specify the name of Another drug or combination that was not already mentioned.

____

B379. Compared to before the COVID pandemic, in the past 6 months, have you been more or less likely to inject alone? (Choose one)

1	More
2	Less
3	Same
4	Not Applicable
8	Refuse to Answer

MOBILITY

If DT_SD is equal to 1 or NDT_TJ is equal to 1 then skip to B380

If not, then skip to instruction before B382.

B380. What country were you in, the first time you injected drugs? (Choose one)

1	US
2	Mexico
3	Other
8	Refuse to Answer

If B380 is not equal to 3, then skip to instruction before B382.

B381. Please specify other country

——————————————————————————————

If DT_SD is equal to 1 then skip to instruction before B382

If not, then skip to instruction before B433.

READ: INTERVIEWER READ TO THE RESPONDENT: The next set of questions will ask you about your experiences in Mexico. Remember, all of your responses are confidential.

If Q4 is equal to 1 then skip to B382

If not, then skip to instruction before B384.

B382. Can you remind me when was the last time you went to Mexico?
Interviewer: Ask for specific number of Days, months, years.

— — —	YEARS
— — —	MONTHS
— — —	DAYS
998	Refuse to Answer (Months)

B383. When was the previous time (before last time) that you travelled to Mexico? **Interviewer: Ask for specific number of Days, months, years.**

__ __ __	YEARS
__ __ __	MONTHS
__ __ __	DAYS
998	Refuse to Answer (Months)

If Q4 is equal to 2 then skip to B384

If not, then skip to instruction before B385.

B384. When was the previous time (before today) that you travelled to Mexico? **Interviewer: Ask for specific number of Days, months, years.**

__ __ __	YEARS
__ __ __	MONTHS
__ __ __	DAYS
998	Refuse to Answer (Months)

LSTMX = If(Q4 is equal to 2, 0, If(Q4 is equal to 1, B382, 999))

If LSTMX is greater than 6 and LSTMX is not equal to 998 then skip to B385

If not, then skip to instruction before B387.

B385. The last time you were **in Mexico** which of the following things did you do with people who live there? **Interviewer: Read out the options and check all that apply** (Check all that apply)

__ Visited friends

__ Visited family

__ Went to a pharmacy

__ Used drugs (not injected)

__ Injected drugs

__ Bought drugs

__ Sold drugs

__ Bought needles

__ Sold sex

__ Bought sex

__ Had sex with a female (not sex trade)

__ Had sex with a male (not sex trade)

__ Other

__ I did not interact with anyone from Mexico

__ Refuse to Answer

If B385N is equal to 1 and B385A + B385B + B385C + B385D + B385E + B385F + B385G + B385H + B385I + B385J + B385K + B385L + B385M is greater than 0 then READ: "You can only choose the response "I did not interact with anyone" by itself. Please check responses." and skip to B385.

If B385M is not equal to 1, then skip to instruction before B387.

B386. Please, specify

____

If LSTMX is less than 6 or LSTMX is equal to 6 then skip to B387

If not, then skip to B407.

B387. How many times did you go to Mexico in **the last 6 months**? __ __ __ times

998 Refuse to Answer

If B387 is equal to 0 or B387 is equal to 998, then skip to B407.

B388. What cities in Mexico did you go to? (Check all that apply)

__ Tijuana

__ Rosarito

__ Ensenada

__ Tecate

__ Mexicali

__ Ciudad Juarez

__ Other

__ Refuse to Answer

If B388G is not equal to 1, then skip to instruction before B390.

B389. Please specify what other city in Mexico

____

If B388A + B388B + B388C + B388D + B388E + B388F + B388G is equal to 1 or B388 is equal to 8, then skip to B392.

B390. What city in Mexico did you go to most frequently in the last 6 months? (Choose one)

1 Tijuana

2 Rosarito

3 Ensenada

4 Tecate

5 Mexicali

6 Ciudad Juarez

7 Other

8 Refuse to Answer

If B390 is equal to 1 and B388A is not equal to 1 or B390 is equal to 2 and B388B is not equal to 1 or B390 is equal to 3 and B388C is not equal to 1 or B390 is equal to 4 and B388D is not equal to 1 or B390 is equal to 5 and B388E is not equal to 1 or B390 is equal to 6 and B388F is not equal to 1 or B390 is equal to 7 and B388G is not equal to 1 then READ: "Participant could only choose the most frequent city out of cities named before." and skip to B390.

If B390 is not equal to 7, then skip to B392.

B391. Please specify what other city in Mexico

____

B392. Which of the following activities did you do when you went to Mexico in the last 6 months?
Interviewer: Read out the options and check all that apply (Check all that apply)

__ Visited friends

__ Visited family

__ Partied

__ Bought drugs

__ Used drugs

__ Went to a shooting gallery

__ Bought sex

__ Sold sex

__ Went to drug tratment

__ Went to a pharmacy

__ Other

__ Refuse to Answer

If B392K is not equal to 1, then skip to instruction before B394.

B393. Please, specify an activity

____

If B392E is equal to 1 then skip to B394

If not, then skip to B396.

B394. Why do you go to Mexico to use drugs? (Check all that apply)

__ Cheaper drugs

__ Better quality/better high

__ Less likely to be hasseled by police

__ More widely available

__ I know the scene and feel more comfortable there

__ I can party/sex workers

__ Other

__ Refuse to Answer

If B394G is not equal to 1, then skip to B396.

B395. Please, specify other reasons

____

B396. In the last 6 months, did you buy any of these things at a Pharmacy in Mexico?
Interviewer: Read out the options and check all that apply
(Check all that apply)

__ Diazepam

__ Viagra or Cialis

__ Rivotril

__ Fentanyl

__ Syringes

__ Antibiotics

__ Valium

__ Ativan

__ Restoril

__ Vicodin

__ Amytal

__ Seconal

__ Darvon

__ Percocet

__ Other

__ Refuse to Answer

__ Not Applicable

If B396O is not equal to 1, then skip to B398.

B397. Please specify other drugs

____

B398. In the last six months, did you pay someone like a jalador or a service to get drugs or buy sex in Mexico?

1 Yes

0 No

8 Refuse to Answer

B399. In the last six months, did you buy preloaded syringes with drugs in Mexico?

1 Yes

0 No

8 Refuse to Answer

If B388A is equal to 1 or B390 is equal to 1 then skip to B400

If not, then skip to B404.

B400. In the last six months, which part of town (i.e., Colonias) did you hang out when you went to TJ? **Interviewer: Look for locations in map as indicated by participant, check all that apply** (Check all that apply)

__ Zona Norte

__ La Mesa

__ El Florido

__ Playas de Tijuana

__ 5 y 10

__ Otay

__ Other

__ Don't Know

__ Refuse to Answer

If B400G is not equal to 1, then skip to instruction before B402.

B401. Please specify which other part of town/colonia:

____

If B400A + B400B + B400C + B400D + B400E + B400F + B400G is equal to 1 or B400 is equal to 97 or B400 is equal to 98 or B400 is equal to 99, then skip to B404.

B402. In the last six months, what part of town (i.e., Colonias) did you hang out most frequently when you went to TJ?
Interviewer: Look for location on map and have participant point location. (Choose one)

01	Zona Norte
02	La Mesa
03	El Florido
04	Playas de Tijuana
05	5 y 10
06	Otay
07	Other
97	Don't Know
98	Refuse to Answer

If B402 is equal to 1 and B400A is not equal to 1 or B402 is equal to 2 and B400B is not equal to 1 or B402 is equal to 3 and B400C is not equal to 1 or B402 is equal to 4 and B400D is not equal to 1 or B402 is equal to 5 and B400E is not equal to 1 or B402 is equal to 6 and B400F is not equal to 1 or B402 is equal to 7 and B400G is not equal to 1 then READ: "Participant could only choose the most frequent colonia out of colonias named before." and skip to B402.

If B402 is not equal to 7, then skip to B404.

B403. Please specify which other part of town/colonia:

____

B404. In the last six months, when you went to Mexico, what kinds of places did you spend time in?
Interviewer: Read out the options and check all that apply (Check all that apply)

__ My home (home defined in demographics section)

__ Someone else's home

__ Shooting gallery

__ Cafe

__ Restaurant

__ Hotel

__ Motel

__ Outside location (Construction site, Alleyway, on the street, vacant lot, park, freeway overpass/bridge, canal)

__ Bar/club

__ Brothel

__ Strip club

__ Public restroom

__ Park

__ Other

__ Refuse to Answer

If B404N is not equal to 1, then skip to B406.

B405. Please specify other place

____

B406. On average, how long did you stay in Mexico when you visited each time in the last 6 months?

__ __ __ MONTHS

__ __ __ DAYS

__ __ __ HOURS

998 Refuse to Answer (Days)

B407. Compared to before the COVID pandemic, in the past 6 months, how much more or less often do you travel to Mexico and use or buy drugs? (Choose one)

1 More

2 Less

3 Same

8 Refuse to Answer

If B387 is equal to 0 or B387 is equal to 998 or LSTMX is greater than 6, then skip to instruction before B433.

READ: INTERVIEWER READ TO THE RESPONDENT: Now I would like you to think about your experiences with people from Mexico while you were in Mexico in the last 6 months.

B408. In the last 6 months, which of the following have you done in Mexico with people who live there? **Interviewer: Read out the options and check all that apply** (Check all that apply)

__ Used drugs (not injected)

__ Injected drugs with them

__ Bought drugs

__ Sold drugs

__ Bought needles

__ Sold sex

__ Bought sex

__ Had sex with a female (not sex trade)

__ Had sex with a male (not sex trade)

__ Other

__ I did not interact with anyone from Mexico

__ Refuse to Answer

If B408K is equal to 1 and B408A + B408B + B408C + B408D + B408E + B408F + B408G + B408H + B408I + B408J is greater than 0 then READ: "You can only choose the response "I did not interact with anyone" by itself. Please check responses." and skip to B408.

If B408J is not equal to 1, then skip to instruction before B410.

B409. Please, specify an activity

____

If B408A is not equal to 1, then skip to instruction before B419.

B410. In the **last 6 months**, when you were in Mexico, how many different people did you **use drugs** (not inject) with that live there?

__ __	# of people
98	Refuse to Answer

If B410 is equal to 0 or B410 is equal to 98, then skip to instruction before B419.

READ: INTERVIEWER READ TO THE RESPONDENT: The next questions refer to the people who live in Mexico that you used non-injection drugs within the past 6 months in Mexico.

B411. What was your relationship to them?
Interviewer: Read out the options and check all that apply (Check all that apply)

__	Family member
__	Friend
__	Sex partner (not from sex trade)
__	Sex trade Client (paying partner)
__	Acquaintance
__	Stranger
__	Other
__	Refuse to Answer

If B411G is not equal to 1, then skip to B413.

B412. Please specify other.

____

B413. Where did you meet these people? (Check all that apply)

__ Their home (home defined in demographics section)

__ Shooting gallery

__ Cafe/Restaurant

__ Motel/Hotel

__ Outside location (Construction site, Alleyway, on the street, vacant lot, park, freeway overpass/bridge, canal)

__ Bar/club

__ Brothel

__ Strip club

__ Public restroom

__ Park

__ Other

__ Refuse to Answer

If B413K is not equal to 1, then skip to B415.

B414. Please specify other place

____

B415. What drugs did you use (not inject) with them? (Check all that apply)

__ Black tar/Heroin by itself

__ Powder heroin by itself

__ Cocaine (powder) by itself

__ Heroin and cocaine together (speedball)

__ Methamphetamine/crystal

__ Crack by itself

__ China White by itself

__ OxyContin, without a prescription

__ Other prescription opioids w/o prescription (Vicodin, Darvon, Percocet)

__ Tranquilizers (Rivotril, Diazepam, Valium, Ativan or Restoril)

__ Barbiturates without a prescription (Amytal, Nembutal, and Seconal)

__ Fentanyl

__ Another drug or combination that was not already mentioned

__ Refuse to Answer

If B415M is not equal to 1, then skip to B417.

B416. Please specify another drug or combination:

____

B417. Did you give or lend any drug use equipment to them, like a pipe or straw? (Choose one)

0 No

1 Yes

2 We didn't use a pipe or straw

8 Refuse to Answer

If B417 is equal to 2, then skip to instruction before B419.

B418. Did you use a pipe or straw after any of them?

1	Yes
0	No
8	Refuse to Answer

If B408B is not equal to 1, then skip to B429.

B419. In the **last 6 months**, when you were in Mexico, how many different people did you **inject drugs** with there?

__ __	# of people
98	Refuse to Answer

If B419 is equal to 0 or B419 is equal to 98, then skip to B429.

READ: INTERVIEWER READ TO THE RESPONDENT: The next questions refer to the people who live in Mexico that you <u>injected</u> drugs with in the past 6 months in Mexico.

B420. What was your relationship to them?
Interviewer: Read out the options and check all that apply (Check all that apply)

__	Family member
__	Friend
__	Sex partner (not from sex trade)
__	Sex trade Client (paying partner)
__	Acquaintance
__	Stranger
__	Other
__	Refuse to Answer

If B420G is not equal to 1, then skip to B422.

B421. Please specify other.

____

B422. Where did you meet these people? (Check all that apply)

__ Their home (home defined in demographics section)

__ Shooting gallery

__ Outside location (Construction site, Alleyway, on the street, vacant lot, park, freeway overpass/bridge, canal)

__ Bar/club

__ Brothel

__ Strip club

__ Public restroom

__ Park

__ Other

__ Refuse to Answer

If B422I is not equal to 1, then skip to B424.

B423. Please specify other place

____

B424. What drugs did you inject with them? (Check all that apply)

__ Black tar/Heroin by itself

__ Powder heroin by itself

__ Cocaine (powder) by itself

__ Heroin and cocaine together (speedball)

__ Methamphetamine/crystal

__ Crack by itself

__ China White by itself

__ OxyContin, without a prescription

__ Other prescription opioids w/o prescription (Vicodin, Darvon, Percocet)

__ Tranquilizers (Rivotril, Diazepam, Valium, Ativan or Restoril)

__ Barbiturates without a prescription (Amytal, Nembutal, and Seconal)

__ Fentanyl

__ Another drug or combination that was not already mentioned

__ Refuse to Answer

If B424M is not equal to 1, then skip to B426.

B425. Please specify another drug or combination:

____

B426. Did you give or lend your syringe to any of these people to inject with after you used it?

1 Yes

0 No

8 Refuse to Answer

B427. Did you inject with a syringe that any of these people used before you used it to inject?

1	Yes
0	No
8	Refuse to Answer

B428. Did you use a cooker, cotton, or water after or at the same time as the other people you were with?

1	Yes
0	No
8	Refuse to Answer

B429. In the **last 6 months**, when you went to Mexico how many people did you give drugs, money, food, shelter, transportation, alcohol, or anything else in exchange for having sex with you?

__ __	# of people
98	Refuse to Answer

B430. Compared to before the COVID pandemic, in the past 6 months when you were in Mexico, have you given drugs, money or anything else in exchange for having sex with you more or less often than before? (Choose one)

1	More
2	Less
3	Same
4	N/A (never)
8	Refuse to Answer

B431. In the **last 6 months**, when you went to Mexico how many people have given you drugs, money, food, shelter, transportation, alcohol, or anything else in exchange for sex?

__ __	# of people
98	Refuse to Answer

B432. Compared to before the COVID pandemic, in the past 6 months when you were in Mexico, has someone given you money, drugs or anything else in exchange for having sex with them more or less often? (Choose one)

1	More
2	Less
3	Same
4	N/A (never)
8	Refuse to Answer

DRUG USE by SD DT in Mexico

If DT_SD is not equal to 1, then skip to B625.

READ: INTERVIEWER READ TO THE RESPONDENT: Now I will ask you some questions about your drug use in Mexico. Some of these questions will ask you for details that can be difficult to remember. Please do your best to give an answer even if it is your best guess. Recall that we will not share your answers with the police or anyone else outside of this study.
If you were in prison/jail in the past 6 months please only think of the time when you were not in prison/jail to answer these questions.

If LSTMX is greater than 6 and LSTMX is not equal to 998, then skip to B434.

B433. During the last 6 months, when you were in Mexico, on average how often did you smoke, vape marijuana or hashish by itself? (Choose one)

00	Never
01	One time per month or less
02	2 to 3 days per month
03	One time per week
04	2 to 3 days per week
05	4 to 6 days per week
06	One time per day
07	2-3 times per day
08	4 times or more per day
98	Refuse to Answer

B434. Have you ever used heroin/black tar when you were in Mexico?

1	Yes
0	No
8	Refuse to Answer

If B434 is not equal to 1, then skip to B448.

B435. Have you ever smoked, snorted or inhaled heroin when you were in Mexico?

1	Yes
0	No
8	Refuse to Answer

If B435 is not equal to 1, then skip to B438.

B436. When was the last time you smoked, snorted, or inhaled heroin in Mexico?

— — —	YEARS
— — —	MONTHS
— — —	DAYS
998	Refuse to Answer (Months)
999	Not Applicable (Months)

If B436 is less than LSTMX then READ: "The response is smaller than the last time participant went to Mexico. Please check the response." and skip to B436.

If B436 is equal to 998 or B436 is equal to 999 or B436 is greater than 6, then skip to B438.

B437. During the last 6 months, on average how often have you smoked, snorted, or inhaled heroin when you were in Mexico? (Choose one)

00	Never
01	One time per month or less
02	2 to 3 days per month
03	One time per week
04	2 to 3 days per week
05	4 to 6 days per week
06	One time per day
07	2-3 times per day
08	4 times or more per day
98	Refuse to Answer

B438. Have you ever injected heroin by itself when you were in Mexico?

1	Yes
0	No
8	Refuse to Answer

If B438 is not equal to 1, then skip to instruction before B441.

B439. When was the last time you injected heroin by itself in Mexico?

— — —	YEARS
— — —	MONTHS
— — —	DAYS
998	Refuse to Answer (Months)
999	Not Applicable (Months)

If B439 is less than LSTMX then READ: "The response is smaller than the last time participant went to Mexico. Please check the response." and skip to B439.

If B439 is equal to 998 or B439 is equal to 999 or B439 is greater than 6, then skip to instruction before B441.

B440. During the last 6 months, on average how often have you injected heroin by itself when you were in Mexico? (Choose one)

00	Never
01	One time per month or less
02	2 to 3 days per month
03	One time per week
04	2 to 3 days per week
05	4 to 6 days per week
06	One time per day
07	2-3 times per day
08	4 times or more per day
98	Refuse to Answer

If (B436 is equal to 998 or B436 is equal to 999 or B436 is greater than 6) and (B439 is equal to 998 or B439 is equal to 999 or B439 is greater than 6), then skip to B448.

B441. In the last 6 months, what colors did the heroin you used come in when you were in Mexico? **Interviewer: Read out the options and check all that apply** (Check all that apply)

__	Black tar
__	Brown powder
__	White powder
__	Yellow powder
__	Orange powder
__	Other
__	Refuse to Answer
__	Not Applicable

If B441F is not equal to 1, then skip to instruction before B443.

B442. Please, specify the color of the heroin you used.

____

If B439 is equal to 998 or B439 is equal to 999 or B439 is greater than 6, then skip to B448.

B443. In the last 6 months, what color was the heroin you injected **most ofte**n when you were in Mexico? (Choose one)

1 Black tar

2 Brown powder

3 White powder

4 Yellow powder

5 Orange powder

6 Other

8 Refuse to Answer

9 Not Applicable

If B443 is not equal to 6, then skip to instruction before B445.

B444. Please, specify the color of the heroin you inject.

____

If B443 is not equal to 4, then skip to instruction before B446.

B445. During the last 6 months when you injected heroin when you were in Mexico, on average how often was it ***yellow***? (Choose one)

1 Never

2 Less than half the time

3 About half of the time

4 More than half the time

5 Always

8 Refuse to Answer

If B443 is not equal to 5, then skip to instruction before B447.

B446. During the last 6 months when you injected heroin when you were in Mexico, on average how often was it ***orange*** heroin? (Choose one)

1	Never
2	Less than half the time
3	About half of the time
4	More than half the time
5	Always
8	Refuse to Answer

If B443 is not equal to 6, then skip to B448.

B447. During the last 6 months when you injected heroin when you were in Mexico, on average how often was it [Response to B444]? (Choose one)

1	Never
2	Less than half the time
3	About half of the time
4	More than half the time
5	Always
8	Refuse to Answer

B448. Have you ever used cocaine or crack when you were in Mexico?

1	Yes
0	No
8	Refuse to Answer

If B448 is not equal to 1, then skip to B458.

B449. Have you ever smoked, snorted or inhaled crack or powder cocaine when you were in Mexico?

1	Yes
0	No
8	Refuse to Answer

If B449 is not equal to 1, then skip to B452.

B450. When was the last time you smoked, snorted, or inhaled cocaine in Mexico?

___ ___ ___	YEARS
___ ___ ___	MONTHS
___ ___ ___	DAYS
998	Refuse to Answer (Months)
999	Not Applicable (Months)

If B450 is less than LSTMX then READ: "The response is smaller than the last time participant went to Mexico. Please check the response." and skip to B450.

If B450 is equal to 998 or B450 is equal to 999 or B450 is greater than 6, then skip to B452.

B451. During the last 6 months, on average how often have you smoked, snorted, or inhaled cocaine when you were in Mexico? (Choose one)

00	Never
01	One time per month or less
02	2 to 3 days per month
03	One time per week
04	2 to 3 days per week
05	4 to 6 days per week
06	One time per day
07	2-3 times per day
08	4 times or more per day
98	Refuse to Answer

B452. Have you ever injected cocaine by itself when you were in Mexico?

1	Yes
0	No
8	Refuse to Answer

If B452 is not equal to 1, then skip to instruction before B455.

B453. When was the last time you injected cocaine by itself in Mexico?

__ __ __	YEARS
__ __ __	MONTHS
__ __ __	DAYS
998	Refuse to Answer (Months)
999	Not Applicable (Months)

If B453 is less than LSTMX then READ: "The response is smaller than the last time participant went to Mexico. Please check the response." and skip to B453.

If B453 is equal to 998 or B453 is equal to 999 or B453 is greater than 6, then skip to instruction before B455.

B454. During the last 6 months, on average how often have you injected cocaine by itself when you were in Mexico? (Choose one)

00	Never
01	One time per month or less
02	2 to 3 days per month
03	One time per week
04	2 to 3 days per week
05	4 to 6 days per week
06	One time per day
07	2-3 times per day
08	4 times or more per day
98	Refuse to Answer

If B434 is not equal to 1, then skip to B458.

B455. Have you ever injected heroin and cocaine together (speedball) when you were in Mexico?

1	Yes
0	No
8	Refuse to Answer

If B455 is not equal to 1, then skip to B458.

B456. When was the last time you injected heroin and cocaine together (speedball) when you were in Mexico?

__ __ __	YEARS
__ __ __	MONTHS
__ __ __	DAYS
998	Refuse to Answer (Months)
999	Not Applicable (Months)

If B456 is less than LSTMX then READ: "The response is smaller than the last time participant went to Mexico. Please check the response." and skip to B456.

If B456 is equal to 998 or B456 is equal to 999 or B456 is greater than 6, then skip to B458.

B457. During the last 6 months, on average how often have you injected heroin and cocaine together (speedball) when you were in Mexico? (Choose one)

00	Never
01	One time per month or less
02	2 to 3 days per month
03	One time per week
04	2 to 3 days per week
05	4 to 6 days per week
06	One time per day
07	2-3 times per day
08	4 times or more per day
98	Refuse to Answer

B458. Have you ever used methamphetamine/crystal when you were in Mexico?

1	Yes
0	No
8	Refuse to Answer

If B458 is not equal to 1, then skip to B475.

B459. Have you ever smoked, snorted, inhaled or vaped meth/crystal by itself when you were in Mexico?

1	Yes
0	No
8	Refuse to Answer

If B459 is not equal to 1, then skip to B462.

B460. When was the last time you smoked, snorted, or inhaled meth/crystal in Mexico?

___ ___ ___	YEARS
___ ___ ___	MONTHS
___ ___ ___	DAYS
998	Refuse to Answer (Months)
999	Not Applicable (Months)

If B460 is less than LSTMX then READ: "The response is smaller than the last time participant went to Mexico. Please check the response." and skip to B460.

If B460 is equal to 998 or B460 is equal to 999 or B460 is greater than 6, then skip to B462.

B461. During the last 6 months, on average how often have you smoked, snorted, or inhaled meth/crystal when you were in Mexico? (Choose one)

00	Never
01	One time per month or less
02	2 to 3 days per month
03	One time per week
04	2 to 3 days per week
05	4 to 6 days per week
06	One time per day
07	2-3 times per day
08	4 times or more per day
98	Refuse to Answer

B462. Have you ever injected meth/crystal by itself when you were in Mexico?

1	Yes
0	No
8	Refuse to Answer

If B462 is not equal to 1, then skip to instruction before B465.

B463. When was the last time you injected meth/crystal in Mexico?

— — —	YEARS
— — —	MONTHS
— — —	DAYS
998	Refuse to Answer (Months)
999	Not Applicable (Months)

If B463 is less than LSTMX then READ: "The response is smaller than the last time participant went to Mexico. Please check the response." and skip to B463.

If B463 is equal to 998 or B463 is equal to 999 or B463 is greater than 6, then skip to instruction before B465.

B464. During the last 6 months, on average how often have you injected meth/crystal by itself when you were in Mexico? (Choose one)

00	Never
01	One time per month or less
02	2 to 3 days per month
03	One time per week
04	2 to 3 days per week
05	4 to 6 days per week
06	One time per day
07	2-3 times per day
08	4 times or more per day
98	Refuse to Answer

If B448 is not equal to 1, then skip to instruction before B468.

B465. Have you ever injected meth/crystal and cocaine together when you were in Mexico?

1	Yes
0	No
8	Refuse to Answer

If B465 is not equal to 1, then skip to instruction before B468.

B466. When was the last time you injected meth/crystal and cocaine together when you were in Mexico?

___ ___ ___	YEARS
___ ___ ___	MONTHS
___ ___ ___	DAYS
998	Refuse to Answer (Months)
999	Not Applicable (Months)

If B466 is less than LSTMX then READ: "The response is smaller than the last time participant went to Mexico. Please check the response." and skip to B466.

If B466 is equal to 998 or B466 is equal to 999 or B466 is greater than 6, then skip to instruction before B468.

B467. During the last 6 months, on average how often have you injected meth/crystal and cocaine together when you were in Mexico? (Choose one)

00	Never
01	One time per month or less
02	2 to 3 days per month
03	One time per week
04	2 to 3 days per week
05	4 to 6 days per week
06	One time per day
07	2-3 times per day
08	4 times or more per day
98	Refuse to Answer

If B434 is not equal to 1, then skip to instruction before B471.

B468. Have you ever injected meth/crystal and heroin together when you were in Mexico?

1	Yes
0	No
8	Refuse to Answer

If B468 is not equal to 1, then skip to instruction before B471.

B469. When was the last time you injected meth/crystal and heroin together when you were in Mexico?

___ ___ ___	YEARS
___ ___ ___	MONTHS
___ ___ ___	DAYS
998	Refuse to Answer (Months)
999	Not Applicable (Months)

If B469 is less than LSTMX then READ: "The response is smaller than the last time participant went to Mexico. Please check the response." and skip to B469.

If B469 is equal to 998 or B469 is equal to 999 or B469 is greater than 6, then skip to instruction before B471.

B470. During the last 6 months, on average how often have you injected meth/crystal and heroin together when you were in Mexico? (Choose one)

00	Never
01	One time per month or less
02	2 to 3 days per month
03	One time per week
04	2 to 3 days per week
05	4 to 6 days per week
06	One time per day
07	2-3 times per day
08	4 times or more per day
98	Refuse to Answer

If (B460 is equal to 998 or B460 is equal to 999 or B460 is greater than 6) and (B463 is equal to 998 or B463 is equal to 999 or B463 is greater than 6) and (B466 is equal to 998 or B466 is equal to 999 or B466 is greater than 6) and (B469 is equal to 998 or B469 is equal to 999 or B469 is greater than 6), then skip to B475.

B471. In the last 6 months when you used methamphetamine when you were in Mexico, what color was it? (Check all that apply)

__ White

__ Pink

__ Green

__ Black

__ Yellow

__ Clear (ice)

__ Other

__ Refuse to Answer

__ Not Applicable

If B471G is not equal to 1, then skip to instruction before B473.

B472. Please, specify the color of methamphetamine when you were in Mexico:

____

If (B463 is equal to 998 or B463 is equal to 999 or B463 is greater than 6) and (B466 is equal to 998 or B466 is equal to 999 or B466 is greater than 6) and (B469 is equal to 998 or B469 is equal to 999 or B469 is greater than 6), then skip to B475.

B473. In the last 6 months when you injected methamphetamine when you were in Mexico, what was the color you injected **most often**? (Choose one)

1	White
2	Pink
3	Green
4	Black
5	Yellow
6	Clear (ice)
7	Other
8	Refuse to Answer
9	Not Applicable

If B473 is not equal to 7, then skip to B475.

B474. Please, specify the color of methamphetamine

____

B475. Have you ever used China White when you were in Mexico?

1	Yes
0	No
8	Refuse to Answer

If B475 is not equal to 1, then skip to B485.

B476. Have you ever smoked, snorted, inhaled or vaped China White when you were in Mexico?

1	Yes
0	No
8	Refuse to Answer

If B476 is not equal to 1, then skip to B479.

B477. When was the last time you smoked, snorted, or inhaled China White in Mexico?

— — —	YEARS
— — —	MONTHS
— — —	DAYS
998	Refuse to Answer (Months)
999	Not Applicable (Months)

If B477 is less than LSTMX then READ: "The response is smaller than the last time participant went to Mexico. Please check the response." and skip to B477.

If B477 is equal to 998 or B477 is equal to 999 or B477 is greater than 6, then skip to B479.

B478. During the last 6 months, on average how often have you smoked, snorted, or inhaled China White when you were in Mexico? (Choose one)

00	Never
01	One time per month or less
02	2 to 3 days per month
03	One time per week
04	2 to 3 days per week
05	4 to 6 days per week
06	One time per day
07	2-3 times per day
08	4 times or more per day
98	Refuse to Answer

B479. Have you ever injected China White by itself when you were in Mexico?

1	Yes
0	No
8	Refuse to Answer

If B479 is not equal to 1, then skip to instruction before B482.

B480. When was the last time you injected China White in Mexico?

___ ___ ___	YEARS
___ ___ ___	MONTHS
___ ___ ___	DAYS
998	Refuse to Answer (Months)
999	Not Applicable (Months)

If B480 is less than LSTMX then READ: "The response is smaller than the last time participant went to Mexico. Please check the response." and skip to B480.

If B480 is equal to 998 or B480 is equal to 999 or B480 is greater than 6, then skip to instruction before B482.

B481. During the last 6 months, on average how often have you injected China White when you were in Mexico? (Choose one)

00	Never
01	One time per month or less
02	2 to 3 days per month
03	One time per week
04	2 to 3 days per week
05	4 to 6 days per week
06	One time per day
07	2-3 times per day
08	4 times or more per day
98	Refuse to Answer

If B458 is not equal to 1, then skip to B485.

B482. Have you ever injected China White and meth/crystal together when you were in Mexico?

1	Yes
0	No
8	Refuse to Answer

If B482 is not equal to 1, then skip to B485.

B483. When was the last time you injected China White and meth/crystal together when you were in Mexico?

__ __ __	YEARS
__ __ __	MONTHS
__ __ __	DAYS
998	Refuse to Answer (Months)
999	Not Applicable (Months)

If B483 is less than LSTMX then READ: "The response is smaller than the last time participant went to Mexico. Please check the response." and skip to B483.

If B483 is equal to 998 or B483 is equal to 999 or B483 is greater than 6, then skip to B485.

B484. During the last 6 months, on average how often have you injected China White and meth/crystal together when you were in Mexico? (Choose one)

00	Never
01	One time per month or less
02	2 to 3 days per month
03	One time per week
04	2 to 3 days per week
05	4 to 6 days per week
06	One time per day
07	2-3 times per day
08	4 times or more per day
98	Refuse to Answer

B485. Have you ever used Fentanyl when you were in Mexico?

1	Yes
0	No
8	Refuse to Answer

If B485 is not equal to 1, then skip to B492.

B486. When was the last time you used Fentanyl in Mexico?

__ __ __	YEARS
__ __ __	MONTHS
__ __ __	DAYS
998	Refuse to Answer (Months)
999	Not Applicable (Months)

If B486 is less than LSTMX then READ: "The response is smaller than the last time participant went to Mexico. Please check the response." and skip to B486.

If B486 is equal to 998 or B486 is equal to 999 or B486 is greater than 6, then skip to B492.

B487. In the past 6 months, have you smoked, inhaled, snorted or vaped Fentanyl in Mexico?

1 Yes

0 No

8 Refuse to Answer

If B487 is not equal to 1, then skip to B489.

B488. During the last 6 months, on average how often have you smoked, snorted, or inhaled Fentanyl when you were in Mexico? (Choose one)

00 Never

01 One time per month or less

02 2 to 3 days per month

03 One time per week

04 2 to 3 days per week

05 4 to 6 days per week

06 One time per day

07 2-3 times per day

08 4 times or more per day

98 Refuse to Answer

B489. In the past 6 months, have you inject Fentanyl in Mexico?

1 Yes

0 No

8 Refuse to Answer

If B489 is not equal to 1, then skip to instruction before B491.

B490. During the last 6 months, on average how often have you inject Fentanyl by itself when you were in Mexico? (Choose one)

00	Never
01	One time per month or less
02	2 to 3 days per month
03	One time per week
04	2 to 3 days per week
05	4 to 6 days per week
06	One time per day
07	2-3 times per day
08	4 times or more per day
98	Refuse to Answer

If B458 is not equal to 1, then skip to B492.

B491. During the last 6 months, on average how often have you injected Fentanyl and Meth/Crystal together when you were in Mexico? (Choose one)

00	Never
01	One time per month or less
02	2 to 3 days per month
03	One time per week
04	2 to 3 days per week
05	4 to 6 days per week
06	One time per day
07	2-3 times per day
08	4 times or more per day
98	Refuse to Answer

B492. Have you ever used PCP/Angel Dust when you were in Mexico?

1	Yes
0	No
8	Refuse to Answer

If B492 is not equal to 1, then skip to B495.

B493. When was the last time you smoked, snorted or inhaled PCP/Angel Dust in Mexico?

— — —	YEARS
— — —	MONTHS
— — —	DAYS
998	Refuse to Answer (Months)
999	Not Applicable (Months)

If B493 is less than LSTMX then READ: "The response is smaller than the last time participant went to Mexico. Please check the response." and skip to B493.

If B493 is equal to 998 or B493 is equal to 999 or B493 is greater than 6, then skip to B495.

B494. During the last 6 months when you used PCP/Angel Dust in Mexico, on average how often did you smoke, inhale, or vape it? (Choose one)

00	Never
01	One time per month or less
02	2 to 3 days per month
03	One time per week
04	2 to 3 days per week
05	4 to 6 days per week
06	One time per day
07	2-3 times per day
08	4 times or more per day
98	Refuse to Answer

B495. Have you ever used Ecstasy when you were in Mexico?

1 Yes

0 No

8 Refuse to Answer

If B495 is not equal to 1, then skip to B501.

B496. When was the last time you used ecstasy in Mexico?

__ __ __ YEARS

__ __ __ MONTHS

__ __ __ DAYS

998 Refuse to Answer (Months)

999 Not Applicable (Months)

If B496 is less than LSTMX then READ: "The response is smaller than the last time participant went to Mexico. Please check the response." and skip to B496.

If B496 is equal to 998 or B496 is equal to 999 or B496 is greater than 6, then skip to B501.

B497. During the last 6 months when you used ecstasy in Mexico, how did you use it?
Interviewer: If the participant indicates that they did not use ecstasy in Mexico in the past 6 months, then check Not Applicable (Check all that apply)

__ Injecting

__ Smoking/chasing

__ Snorting

__ Inhaling

__ Swallowing

__ Rectally/bumping

__ Other

__ Not Applicable

__ Refuse to Answer

If B497 is greater than 1 and B497H is equal to 1 then READ: "You can only choose the response "Not Applicable" by itself. Please check responses." and skip to B497.

If B497G is not equal to 1, then skip to instruction before B499.

B498. Specify what other way you used Ecstasy in the last 6 months in Mexico.

____

If B497A is not equal to 1, then skip to instruction before B500.

B499. During the last 6 months when you used ecstasy in Mexico, on average how often did you inject it? (Choose one)

00	Never
01	One time per month or less
02	2 to 3 days per month
03	One time per week
04	2 to 3 days per week
05	4 to 6 days per week
06	One time per day
07	2-3 times per day
08	4 times or more per day
98	Refuse to Answer

If B497H is equal to 1 or B497 is equal to 98, then skip to B501.

B500. During the last 6 months when you used ecstasy in Mexico, on average how often did you use it in other ways (not inject it)? (Choose one)

00	Never
01	One time per month or less
02	2 to 3 days per month
03	One time per week
04	2 to 3 days per week
05	4 to 6 days per week
06	One time per day
07	2-3 times per day
08	4 times or more per day
98	Refuse to Answer

B501. Have you ever used Ketamine (Special K), K when you were in Mexico?

1	Yes
0	No
8	Refuse to Answer

If B501 is not equal to 1, then skip to B507.

B502. When was the last time you used Ketamine (Special K), K in Mexico?

— — —	YEARS
— — —	MONTHS
— — —	DAYS
998	Refuse to Answer (Months)
999	Not Applicable (Months)

If B502 is less than LSTMX then READ: "The response is smaller than the last time participant went to Mexico. Please check the response." and skip to B502.

If B502 is equal to 998 or B502 is equal to 999 or B502 is greater than 6, then skip to B507.

B503. During the last 6 months when you used Ketamine (Special K), K in Mexico, how did you use it? **Interviewer: If the participant indicates that they did not use ketamine in Mexico in the past 6 months, then check Not Applicable** (Check all that apply)

__ Injecting

__ Smoking/chasing

__ Snorting

__ Inhaling

__ Swallowing

__ Rectally/bumping

__ Other

__ Not Applicable

__ Refuse to Answer

If B503 is greater than 1 and B503H is equal to 1 then READ: "You can only choose the response "Not Applicable" by itself. Please check responses." and skip to B503.

If B503G is not equal to 1, then skip to instruction before B505.

B504. Specify what other way you used Ketamine in the last 6 months in Mexico.

____

If B503A is not equal to 1, then skip to instruction before B506.

B505. During the last 6 months when you used Ketamine (Special K, K) in Mexico, on average how often did you inject it? (Choose one)

00	Never
01	One time per month or less
02	2 to 3 days per month
03	One time per week
04	2 to 3 days per week
05	4 to 6 days per week
06	One time per day
07	2-3 times per day
08	4 times or more per day
98	Refuse to Answer

If B503H is equal to 1 or B503 is equal to 98, then skip to B507.

B506. During the last 6 months when you used Ketamine (Special K), K in Mexico, on average how often did you use it in other ways (not inject it)? (Choose one)

00	Never
01	One time per month or less
02	2 to 3 days per month
03	One time per week
04	2 to 3 days per week
05	4 to 6 days per week
06	One time per day
07	2-3 times per day
08	4 times or more per day
98	Refuse to Answer

B507. Have you ever used Oxycontin when you were in Mexico?

1 Yes

0 No

8 Refuse to Answer

If B507 is not equal to 1, then skip to B513.

B508. When was the last time you used Oxycontin in Mexico?

__ __ __ YEARS

__ __ __ MONTHS

__ __ __ DAYS

998 Refuse to Answer (Months)

999 Not Applicable (Months)

If B508 is less than LSTMX then READ: "The response is smaller than the last time participant went to Mexico. Please check the response." and skip to B508.

If B508 is equal to 998 or B508 is equal to 999 or B508 is greater than 6, then skip to B513.

B509. During the last 6 months when you used Oxycontin in Mexico, how did you use it?
Interviewer: If the participant indicates that they did not use oxycontin in Mexico in the past 6 months, then check Not Applicable (Check all that apply)

__ Injecting

__ Smoking/chasing

__ Snorting

__ Inhaling

__ Swallowing

__ Rectally/bumping

__ Other

__ Not Applicable

__ Refuse to Answer

If B509 is greater than 1 and B509H is equal to 1 then READ: "You can only choose the response "Not Applicable" by itself. Please check responses." and skip to B509.

If B509G is not equal to 1, then skip to instruction before B511.

B510. Specify what other way you used Oxycontin in the last 6 months in Mexico.

____

If B509A is not equal to 1, then skip to instruction before B512.

B511. During the last 6 months when you used Oxycontin in Mexico, on average how often did you inject it? (Choose one)

00	Never
01	One time per month or less
02	2 to 3 days per month
03	One time per week
04	2 to 3 days per week
05	4 to 6 days per week
06	One time per day
07	2-3 times per day
08	4 times or more per day
98	Refuse to Answer

If B509H is equal to 1 or B509 is equal to 98, then skip to B513.

B512. During the last 6 months when you used Oxycontin in Mexico, on average how often did you use it in other ways (not inject it)? (Choose one)

00	Never
01	One time per month or less
02	2 to 3 days per month
03	One time per week
04	2 to 3 days per week
05	4 to 6 days per week
06	One time per day
07	2-3 times per day
08	4 times or more per day
98	Refuse to Answer

B513. Have you ever used other prescription opioids (Vicodin, Darvon, Percocet) when you were in Mexico?

1	Yes
0	No
8	Refuse to Answer

If B513 is not equal to 1, then skip to B519.

B514. When was the last time you used opioids (Vicodin, Darvon, Percocet) in Mexico?

— — —	YEARS
— — —	MONTHS
— — —	DAYS
998	Refuse to Answer (Months)
999	Not Applicable (Months)

If B514 is less than LSTMX then READ: "The response is smaller than the last time participant went to Mexico. Please check the response." and skip to B514.

If B514 is equal to 998 or B514 is equal to 999 or B514 is greater than 6, then skip to B519.

B515. During the last 6 months when you used opioids (Vicodin, Darvon, Percocet) in Mexico, how did you use it?
Interviewer: If the participant indicates that they did not use opioids in Mexico in the past 6 months, then check Not Applicable (Check all that apply)

__ Injecting

__ Smoking/chasing

__ Snorting

__ Inhaling

__ Swallowing

__ Rectally/bumping

__ Other

__ Not Applicable

__ Refuse to Answer

If B515 is greater than 1 and B515H is equal to 1 then READ: "You can only choose the response "Not Applicable" by itself. Please check responses." and skip to B515.

If B515G is not equal to 1, then skip to instruction before B517.

B516. Specify what other way you used other prescription opioids (Vicodin, Darvon, Percocet) in the last 6 months.

____

If B515A is not equal to 1, then skip to instruction before B518.

B517. During the last 6 months when you used opioids (Vicodin, Darvon, Percocet) in Mexico, on average how often did you inject it? (Choose one)

00	Never
01	One time per month or less
02	2 to 3 days per month
03	One time per week
04	2 to 3 days per week
05	4 to 6 days per week
06	One time per day
07	2-3 times per day
08	4 times or more per day
98	Refuse to Answer

If B515H is equal to 1 or B515 is equal to 98, then skip to B519.

B518. During the last 6 months when you used opioids (Vicodin, Darvon, Percocet) in Mexico, on average how often did you use it in other ways (not inject it)? (Choose one)

00	Never
01	One time per month or less
02	2 to 3 days per month
03	One time per week
04	2 to 3 days per week
05	4 to 6 days per week
06	One time per day
07	2-3 times per day
08	4 times or more per day
98	Refuse to Answer

B519. Have you ever used other prescription Hallucinogenic drugs like acid, LSD, peyote, mescaline, mushrooms when you were in Mexico?

1 Yes

0 No

8 Refuse to Answer

If B519 is not equal to 1, then skip to B522.

B520. When was the last time you used Hallucinogenic drugs like acid, LSD, peyote, mescaline, mushrooms in Mexico?

__ __ __ YEARS

__ __ __ MONTHS

__ __ __ DAYS

998 Refuse to Answer (Months)

999 Not Applicable (Months)

If B520 is less than LSTMX then READ: "The response is smaller than the last time participant went to Mexico. Please check the response." and skip to B520.

If B520 is equal to 998 or B520 is equal to 999 or B520 is greater than 6, then skip to B522.

B521. During the last 6 months, on average how often have you used Hallucinogenic drugs like acid, LSD, peyote, mescaline, mushrooms when you were in Mexico? (Choose one)

00	Never
01	One time per month or less
02	2 to 3 days per month
03	One time per week
04	2 to 3 days per week
05	4 to 6 days per week
06	One time per day
07	2-3 times per day
08	4 times or more per day
98	Refuse to Answer

B522. Have you ever used other prescription inhalants (glue, gasoline) when you were in Mexico?

1	Yes
0	No
8	Refuse to Answer

If B522 is not equal to 1, then skip to B525.

B523. When was the last time you used inhalants (glue, gasoline) in Mexico?

__ __ __	YEARS
__ __ __	MONTHS
__ __ __	DAYS
998	Refuse to Answer (Months)
999	Not Applicable (Months)

If B523 is less than LSTMX then READ: "The response is smaller than the last time participant went to Mexico. Please check the response." and skip to B523.

If B523 is equal to 998 or B523 is equal to 999 or B523 is greater than 6, then skip to B525.

B524. During the last 6 months, on average how often have you used Inhalants (glue or gasoline) in Mexico? (Choose one)

00	Never
01	One time per month or less
02	2 to 3 days per month
03	One time per week
04	2 to 3 days per week
05	4 to 6 days per week
06	One time per day
07	2-3 times per day
08	4 times or more per day
98	Refuse to Answer

B525. Have you ever used tranquilizers (Diazepam, Valium, Ativan, Restoril or Rivotril) when you were in Mexico?

1	Yes
0	No
8	Refuse to Answer

If B525 is not equal to 1, then skip to B531.

B526. When was the last time you used tranquilizers (Diazepam, Valium, Ativan, Restoril or Rivotril) in Mexico?

___ ___ ___	YEARS
___ ___ ___	MONTHS
___ ___ ___	DAYS
998	Refuse to Answer (Months)
999	Not Applicable (Months)

If B526 is less than LSTMX then READ: "The response is smaller than the last time participant went to Mexico. Please check the response." and skip to B526.

If B526 is equal to 998 or B526 is equal to 999 or B526 is greater than 6, then skip to B531.

B527. If you used tranquilizers (Diazepam, Valium, Ativan, Restoril or Rivotril) in the last 6 months, how did you use it?
Interviewer: If the participant indicates that they did not use tranquilizers in Mexico in the past 6 months, then check Not Applicable (Check all that apply)

__ Injecting

__ Smoking/chasing

__ Snorting

__ Inhaling

__ Swallowing

__ Rectally/bumping

__ Other

__ Not Applicable

__ Refuse to Answer

If B527 is greater than 1 and B527H is equal to 1 then READ: "You can only choose the response "Not Applicable" by itself. Please check responses." and skip to B527.

If B527G is not equal to 1, then skip to instruction before B529.

B528. Specify what other way you used tranquilizers (Diazepam, Valium, Ativan, Restoril or Rivotril) in the last 6 months.

____

If B527A is not equal to 1, then skip to instruction before B530.

B529. During the last 6 months, on average how often have you injected tranquilizers (Diazepam, Valium, Ativan, Restoril or Rivotril) in Mexico? (Choose one)

00	Never
01	One time per month or less
02	2 to 3 days per month
03	One time per week
04	2 to 3 days per week
05	4 to 6 days per week
06	One time per day
07	2-3 times per day
08	4 times or more per day
98	Refuse to Answer

If B527H is equal to 1 or B527 is equal to 98, then skip to B531.

B530. During the last 6 months, on average how often have you used tranquilizers (Diazepam, Valium, Ativan, Restoril or Rivotril) in Mexico in other ways (not injecting them)? (Choose one)

00	Never
01	One time per month or less
02	2 to 3 days per month
03	One time per week
04	2 to 3 days per week
05	4 to 6 days per week
06	One time per day
07	2-3 times per day
08	4 times or more per day
98	Refuse to Answer

B531. Have you ever used barbiturates (Amytal, Nembutal, and Seconal) when you were in Mexico?

1	Yes
0	No
8	Refuse to Answer

If B531 is not equal to 1, then skip to B537.

B532. When was the last time you used barbiturates (Amytal, Nembutal, and Seconal) in Mexico?

— — —	YEARS
— — —	MONTHS
— — —	DAYS
998	Refuse to Answer (Months)
999	Not Applicable (Months)

If B532 is less than LSTMX then READ: "The response is smaller than the last time participant went to Mexico. Please check the response." and skip to B532.

If B532 is equal to 998 or B532 is equal to 999 or B532 is greater than 6, then skip to B537.

B533. If you used barbiturates (Amytal, Nembutal, and Seconal) in the last 6 months in Mexico, how did you use it?
Interviewer: If the participant indicates that they did not use barbiturates in Mexico in the past 6 months, then check Not Applicable (Check all that apply)

__ Injecting

__ Smoking/chasing

__ Snorting

__ Inhaling

__ Swallowing

__ Rectally/bumping

__ Other

__ Not Applicable

__ Refuse to Answer

If B533 is greater than 1 and B533H is equal to 1 then READ: "You can only choose the response "Not Applicable" by itself. Please check responses." and skip to B533.

If B533G is not equal to 1, then skip to instruction before B535.

B534. Specify what other way you used barbiturates (Amytal, Nembutal, and Seconal) in the last 6 months in Mexico.

____

If B533A is not equal to 1, then skip to instruction before B536.

B535. During the last 6 months, on average how often have you injected barbiturates (Amytal, Nembutal, and Seconal) in Mexico? (Choose one)

00	Never
01	One time per month or less
02	2 to 3 days per month
03	One time per week
04	2 to 3 days per week
05	4 to 6 days per week
06	One time per day
07	2-3 times per day
08	4 times or more per day
98	Refuse to Answer

If B533H is equal to 1 or B533 is equal to 98, then skip to B537.

B536. During the last 6 months, on average how often have you used barbiturates (Amytal, Nembutal, and Seconal) in Mexico in other ways (not injecting)? (Choose one)

00	Never
01	One time per month or less
02	2 to 3 days per month
03	One time per week
04	2 to 3 days per week
05	4 to 6 days per week
06	One time per day
07	2-3 times per day
08	4 times or more per day
98	Refuse to Answer

B537. Have you ever used brain pills (like modafinil, nootropic, ritalin) in Mexico?

1 Yes

0 No

8 Refuse to Answer

If B537 is not equal to 1, then skip to B543.

B538. When was the last time you used brain pills (like Modafinil, Nootropic, Ritalin) in Mexico?

__ __ __ YEARS

__ __ __ MONTHS

__ __ __ DAYS

998 Refuse to Answer (Months)

999 Not Applicable (Months)

If B538 is less than LSTMX then READ: "The response is smaller than the last time participant went to Mexico. Please check the response." and skip to B538.

If B538 is equal to 998 or B538 is equal to 999 or B538 is greater than 6, then skip to B543.

B539. If you used brain pills (Nootropic, modafinil, ritalin) in the last 6 months in Mexico, how did you use it? **Interviewer: If the participant indicates that they did not use brain pills in Mexico in the past 6 months, then check Not Applicable** (Check all that apply)

__ Injecting

__ Smoking/chasing

__ Snorting

__ Inhaling

__ Swallowing

__ Rectally/bumping

__ Other

__ Not Applicable

__ Refuse to Answer

If B539 is greater than 1 and B539H is equal to 1 then READ: "You can only choose the response "Not Applicable" by itself. Please check responses." and skip to B539.

If B539G is not equal to 1, then skip to instruction before B541.

B540. Specify what other way you used brain pills (Nootropic, modafinil, ritalin) in the last 6 months in Mexico.

____

If B539A is not equal to 1, then skip to instruction before B542.

B541. During the last 6 months, on average how often have you injected brain pills (Nootropic, modafinil, ritalin) in Mexico? (Choose one)

00	Never
01	One time per month or less
02	2 to 3 days per month
03	One time per week
04	2 to 3 days per week
05	4 to 6 days per week
06	One time per day
07	2-3 times per day
08	4 times or more per day
98	Refuse to Answer

If B539H is equal to 1 or B539 is equal to 98, then skip to B543.

B542. During the last 6 months, on average how often have you used brain pills (Nootropic, modafinil, ritalin) in Mexico in other ways (not injecting)? (Choose one)

00	Never
01	One time per month or less
02	2 to 3 days per month
03	One time per week
04	2 to 3 days per week
05	4 to 6 days per week
06	One time per day
07	2-3 times per day
08	4 times or more per day
98	Refuse to Answer

B543. Have you ever used another drug by itself (Not Mentioned Earlier) when you were in Mexico? **Interviewer: Please prompt for "bath salts".**

1	Yes
0	No
8	Refuse to Answer

If B543 is not equal to 1, then skip to B557.

B544. Please specify the 1st other drug that you used by itself when you were in Mexico?

____

B545. When was the last time you used [Response to B544] by itself in Mexico?

___ ___ ___	YEARS
___ ___ ___	MONTHS
___ ___ ___	DAYS
998	Refuse to Answer (Months)
999	Not Applicable (Months)

If B545 is less than LSTMX then READ: "The response is smaller than the last time participant went to Mexico. Please check the response." and skip to B545.

If B545 is equal to 998 or B545 is equal to 999 or B545 is greater than 6, then skip to B550.

B546. If you used [Response to B544] in the last 6 months in Mexico, how did you use it?
Interviewer: If the participant indicates that they did not use [Response to B544] **in Mexico in the past 6 months, then check Not Applicable** (Check all that apply)

___	Injecting
___	Smoking/chasing
___	Snorting
___	Inhaling
___	Swallowing
___	Rectally/bumping
___	Other
___	Not Applicable
___	Refuse to Answer

If B546 is greater than 1 and B546H is equal to 1 then READ: "You can only choose the response "Not Applicable" by itself. Please check responses." and skip to B546.

If B546G is not equal to 1, then skip to instruction before B548.

B547. Specify what other way you used [Response to B544] in the last 6 months in Mexico.

____

If B546A is not equal to 1, then skip to instruction before B549.

B548. During the last 6 months, on average how often have you injected [Response to B544] in Mexico? (Choose one)

00	Never
01	One time per month or less
02	2 to 3 days per month
03	One time per week
04	2 to 3 days per week
05	4 to 6 days per week
06	One time per day
07	2-3 times per day
08	4 times or more per day
98	Refuse to Answer

If B546H is equal to 1 or B546 is equal to 98, then skip to B550.

B549. During the last 6 months, on average how often have you used [Response to B544] in Mexico in other ways (not injecting)? (Choose one)

00	Never
01	One time per month or less
02	2 to 3 days per month
03	One time per week
04	2 to 3 days per week
05	4 to 6 days per week
06	One time per day
07	2-3 times per day
08	4 times or more per day
98	Refuse to Answer

B550. Have you ever used another drug by itself (Not Mentioned Earlier) when you were in Mexico?

1	Yes
0	No
8	Refuse to Answer

If B550 is not equal to 1, then skip to B557.

B551. Please specify the 2nd other drug that you used by itself when you were in Mexico?

____

B552. When was the last time you used [Response to B551] by itself in Mexico?

— — —	YEARS
— — —	MONTHS
— — —	DAYS
998	Refuse to Answer (Months)
999	Not Applicable (Months)

If B552 is less than LSTMX then READ: "The response is smaller than the last time participant went to Mexico. Please check the response." and skip to B552.

If B552 is equal to 998 or B552 is equal to 999 or B552 is greater than 6, then skip to B557.

B553. If you used [Response to B551] in the last 6 months in Mexico, how did you use it?
Interviewer: If the participant indicates that they did not use [Response to B551] **in Mexico in the past 6 months, then check Not Applicable** (Check all that apply)

__	Injecting
__	Smoking/chasing
__	Snorting
__	Inhaling
__	Swallowing
__	Rectally/bumping
__	Other
__	Not Applicable
__	Refuse to Answer

If B553 is greater than 1 and B553H is equal to 1 then READ: "You can only choose the response "Not Applicable" by itself. Please check responses." and skip to B553.

If B553G is not equal to 1, then skip to instruction before B555.

B554. Specify what other way you used [Response to B551] in the last 6 months in Mexico.

— —

If B553A is not equal to 1, then skip to instruction before B556.

B555. During the last 6 months, on average how often have you injected [Response to B551] in Mexico? (Choose one)

00	Never
01	One time per month or less
02	2 to 3 days per month
03	One time per week
04	2 to 3 days per week
05	4 to 6 days per week
06	One time per day
07	2-3 times per day
08	4 times or more per day
98	Refuse to Answer

If B553H is equal to 1 or B553 is equal to 98, then skip to B557.

B556. During the last 6 months, on average how often have you used [Response to B551] in Mexico in other ways (not injecting)? (Choose one)

00	Never
01	One time per month or less
02	2 to 3 days per month
03	One time per week
04	2 to 3 days per week
05	4 to 6 days per week
06	One time per day
07	2-3 times per day
08	4 times or more per day
98	Refuse to Answer

B557. Have you ever used another drug combination (Not Mentioned Earlier) when you were in Mexico?

1	Yes
0	No
8	Refuse to Answer

If B557 is not equal to 1, then skip to instruction before B571.

B558. Please specify the 1st other drug combination that you used when you were in Mexico?

____

B559. When was the last time you used [Response to B558] in Mexico?

__ __ __	YEARS
__ __ __	MONTHS
__ __ __	DAYS
998	Refuse to Answer (Months)
999	Not Applicable (Months)

If B559 is less than LSTMX then READ: "The response is smaller than the last time participant went to Mexico. Please check the response." and skip to B559.

If B559 is equal to 998 or B559 is equal to 999 or B559 is greater than 6, then skip to B564.

B560. If you used [Response to B558] in the last 6 months in Mexico, how did you use it?
Interviewer: If the participant indicates that they did not use [Response to B558] **in Mexico in the past 6 months, then check Not Applicable** (Check all that apply)

__	Injecting
__	Smoking/chasing
__	Snorting
__	Inhaling
__	Swallowing
__	Rectally/bumping
__	Other
__	Not Applicable
__	Refuse to Answer

If B560 is greater than 1 and B560H is equal to 1 then READ: "You can only choose the response "Not Applicable" by itself. Please check responses." and skip to B560.

If B560G is not equal to 1, then skip to instruction before B562.

B561. Specify what other way you used [Response to B558] in the last 6 months in Mexico.

__ __

If B560A is not equal to 1, then skip to instruction before B563.

B562. During the last 6 months, on average how often have you injected [Response to B558] in Mexico? (Choose one)

00	Never
01	One time per month or less
02	2 to 3 days per month
03	One time per week
04	2 to 3 days per week
05	4 to 6 days per week
06	One time per day
07	2-3 times per day
08	4 times or more per day
98	Refuse to Answer

If B560H is equal to 1 or B560 is equal to 98, then skip to B564.

B563. During the last 6 months, on average how often have you used [Response to B558] in Mexico in other ways (not injecting)? (Choose one)

00	Never
01	One time per month or less
02	2 to 3 days per month
03	One time per week
04	2 to 3 days per week
05	4 to 6 days per week
06	One time per day
07	2-3 times per day
08	4 times or more per day
98	Refuse to Answer

B564. Have you ever used another drug combination (Not Mentioned Earlier) when you were in Mexico?

1	Yes
0	No
8	Refuse to Answer

If B564 is not equal to 1, then skip to instruction before B571.

B565. Please specify the 2nd other drug combination that you used when you were in Mexico?

____

B566. When was the last time you used [Response to B565] by itself in Mexico?

__ __ __	YEARS
__ __ __	MONTHS
__ __ __	DAYS
998	Refuse to Answer (Months)
999	Not Applicable (Months)

If B566 is less than LSTMX then READ: "The response is smaller than the last time participant went to Mexico. Please check the response." and skip to B566.

If B566 is equal to 998 or B566 is equal to 999 or B566 is greater than 6, then skip to instruction before B571.

B567. If you used [Response to B565] in the last 6 months in Mexico, how did you use it?
Interviewer: If the participant indicates that they did not use [Response to B565] **in Mexico in the past 6 months, then check Not Applicable** (Check all that apply)

__	Injecting
__	Smoking/chasing
__	Snorting
__	Inhaling
__	Swallowing
__	Rectally/bumping
__	Other
__	Not Applicable
__	Refuse to Answer

If B567 is greater than 1 and B567H is equal to 1 then READ: "You can only choose the response "Not Applicable" by itself. Please check responses." and skip to B567.

If B567G is not equal to 1, then skip to instruction before B569.

B568. Specify what other way you used [Response to B565] in the last 6 months in Mexico.

__ __

If B567A is not equal to 1, then skip to instruction before B570.

B569. During the last 6 months, on average how often have you injected [Response to B565] in Mexico? (Choose one)

00	Never
01	One time per month or less
02	2 to 3 days per month
03	One time per week
04	2 to 3 days per week
05	4 to 6 days per week
06	One time per day
07	2-3 times per day
08	4 times or more per day
98	Refuse to Answer

If B567H is equal to 1 or B567 is equal to 98, then skip to instruction before B571.

B570. During the last 6 months, on average how often have you used [Response to B565] in Mexico in other ways (not injecting)? (Choose one)

00	Never
01	One time per month or less
02	2 to 3 days per month
03	One time per week
04	2 to 3 days per week
05	4 to 6 days per week
06	One time per day
07	2-3 times per day
08	4 times or more per day
98	Refuse to Answer

ANYINJMX = If(B440 is not equal to 0 and B440 is not equal to 98 and B440 is not equal to "skipped" or B454 is not equal to 0 and B454 is not equal to 98 and B454 is not equal to "skipped" or B457 is not equal to 0 and B457 is not equal to 98 and B457 is not equal to "skipped" or B464 is not equal to 0 and B464 is not equal to 98 and B464 is not equal to "skipped" or B467 is not equal to 0 and B467 is not equal to 98 and B467 is not equal to "skipped" or B470 is not equal to 0 and B470 is not equal to 98 and B470 is not equal to "skipped" or B481 is not equal to 0 and B481 is not equal to 98 and B481 is not equal to "skipped" or B484 is not equal to 0 and B484 is not equal to 98 and B484 is not equal to "skipped" or B490 is not equal to 0 and B490 is not equal to 98 and B490 is not equal to "skipped" or B491 is not equal to 0 and B491 is not equal to 98 and B491 is not equal to "skipped" or B499 is not equal to 0 and B499 is not equal to 98 and B499 is not equal to "skipped" or B505 is not equal to 0 and B505 is not equal to 98 and B505 is not equal to "skipped" or B511 is not equal to 0 and B511 is not equal to 98 and B511 is not equal to "skipped" or B517 is not equal to 0 and B517 is not equal to 98 and B517 is not equal to "skipped" or B529 is not equal to 0 and B529 is not equal to 98 and B529 is not equal to "skipped" or B535 is not equal to 0 and B535 is not equal to 98 and B535 is not equal to "skipped" or B541 is not equal to 0 and B541 is not equal to 98 and B541 is not equal to "skipped" or B548 is not equal to 0 and B548 is not equal to 98 and B548 is not equal to "skipped" or B555 is not equal to 0 and B555 is not equal to 98 and B555 is not equal to "skipped" or B562 is not equal to 0 and B562 is not equal to 98 and B562 is not equal to "skipped" or B569 is not equal to 0 and B569 is not equal to 98 and B569 is not equal to "skipped", 1, 0)

READ: INTERVIEWER READ TO THE RESPONDENT: The Next Questions have to do with the ways you may have injected drugs in Mexico. I know I asked you before but can you remind me…

If LSTMX is greater than 6 and LSTMX is not equal to 998, then skip to B591.

B571. In the past 6 months when you were in Mexico, on average, how often did you inject (shoot) any drug or drug combination? (Choose one)

00	Never
01	One time per month or less
02	2 to 3 days per month
03	One time per week
04	2 to 3 days per week
05	4 to 6 days per week
06	One time per day
07	2-3 times per day
08	4 times or more per day
98	Refuse to Answer

B572. Of the times you injected in the last 6 months when you were in Mexico, how often did you give, rent, sell or lend a syringe that you already used to someone else? (Choose one)

1	Never
2	Sometimes
3	About half of the time
4	Often
5	Always
8	Refuse to Answer

B573. Of the times you injected in the last 6 months when you were in Mexico, how often did you use a syringe that you knew or suspected that it had been used before by someone else? (Choose one)

1 Never

2 Sometimes

3 About half of the time

4 Often

5 Always

8 Refuse to Answer

If B573 is equal to 1 or B573 is equal to 8, then skip to B576.

B574. The last time you injected drugs when you were in Mexico, did you use a syringe that you knew or suspected that it had been used before by someone else?

1 Yes

0 No

8 Refuse to Answer

B575. Of the times you injected with a syringe that had been used by somebody else in the last 6 months when you were in Mexico, how often did you clean it with bleach before you used it? (Choose one)

1 Never

2 Sometimes

3 About half of the time

4 Often

5 Always

8 Refuse to Answer

B576. Of the times you injected with other people in the last 6 months when you were in Mexico, how often did you divide up drugs with somebody else by using a syringe? This is also called back loading, piggybacking or splitting drugs wet. (Choose one)

1 Never
2 Sometimes
3 About half of the time
4 Often
5 Always
8 Refuse to Answer

B577. Of the times you injected in the last 6 months when you were in Mexico, how often did you use a cooker, cotton, or water with someone or after someone else used it? (Choose one)

1 Never
2 Sometimes
3 About half of the time
4 Often
5 Always
8 Refuse to Answer

B578. Of the times you injected in the last 6 months when you were in Mexico, how often did you buy drugs that came already prepared in a syringe? (Choose one)

1 Never
2 Sometimes
3 About half of the time
4 Often
5 Always
8 Refuse to Answer

B579. Of the times you injected in the last 6 months when you were in Mexico, how often did you inject with a new, sterile syringe? (Choose one)

1 Never

2 Sometimes

3 About half of the time

4 Often

5 Always

8 Refuse to Answer

B580. In the last 6 months when you were in Mexico, from where did you get the syringes you used to inject drugs? (Check all that apply)

__ Pharmacy

__ Spouse, family member, or sex partner

__ Friend

__ Drug dealer

__ Hit doctor

__ Shooting gallery

__ Syringe exchange program (you exchanged it yourself)

__ Someone who gets them from a syringe exchange program

__ Bought on the street

__ Doctor/clinic/hospital

__ Veterinary clinic/ pet store

__ Market/store

__ Found on the ground or in the trash

__ Some other place

__ Refuse to Answer

If B580N is not equal to 1, then skip to instruction before B582.

B581. Please specify the "other" place where you got syringes from in the past 6 months when you were in Mexico.

____

If B580A + B580B + B580C + B580D + B580E + B580F + B580G + B580H + B580I + B580J + B580K + B580L + B580M + B580N is equal to 1 or B580 is equal to 98, then skip to instruction before B584.

B582. In the last 6 months when you were in Mexico, where did you get your syringes most often from? (Choose one)

01	Pharmacy
02	Spouse, family member, or sex partner
03	Friend
04	Drug dealer
05	Hit doctor
06	Shooting gallery
07	Syringe exchange program (you exchanged it yourself)
08	Someone who gets them from a syringe exchange program
09	Bought on the street
10	Doctor/ clinic/ hospital
11	Veterinary clinic/ pet store
12	Market/Store
13	Found on the ground or in the trash
14	Some other place
98	Refuse to Answer

If B582 is equal to 1 and B580A is not equal to 1 or B582 is equal to 2 and B580B is not equal to 1 or B582 is equal to 3 and B580C is not equal to 1 or B582 is equal to 4 and B580D is not equal to 1 or B582 is equal to 5 and B580E is not equal to 1 or B582 is equal to 6 and B580F is not equal to 1 or B582 is equal to 7 and B580G is not equal to 1 or B582 is equal to 8 and B580H is not equal to 1 or B582 is equal to 9 and B580I is not equal to 1 or B582 is equal to 10 and B580J is not equal to 1 or B582 is equal to 11 and B580K is not equal to 1 or B582 is equal to 12 and B580L is not equal to 1 or B582 is equal to 13 and B580M is not equal to 1 or B582 is equal to 14 and B580N is not equal to 1 then READ: "Participant could only chooce the most often place out of places named before." and skip to B582.

If B582 is not equal to 14, then skip to instruction before B584.

B583. Please specify the place where you've got your syringes most often from, in the past 6 months when you were in Mexico.

____

If B580G is equal to 1 or B582 is equal to 7 then skip to B584

If not, then skip to instruction before B586.

B584. In the last 6 months when you were in Mexico, how many sterile, clean syringes per visit did you get from a Needle Exchange Program on average?

___

998 Refuse to Answer

If B584 is equal to 0 or B584 is equal to 998, then skip to instruction before B586.

B585. What was the name of the Needle Exchange Program you got syringes from most often when you were in Mexico?

____

If B580A is equal to 1 or B582 is equal to 1 then skip to B586

If not, then skip to B588.

B586. In the last 6 months when you were in Mexico, how many sterile, clean syringes per visit did you get from a pharmacy on average?

___

998 Refuse to Answer

If B586 is equal to 0 or B586 is equal to 998, then skip to B588.

B587. What was the name of the Pharmacy you bought syringes from most often when you were in Mexico?

____

B588. In the last 6 months when you were in Mexico, how easy or hard was it for you to get new, unused syringes when you injected drugs? (Choose one)

1 Never tried to get new, unused syringes

2 Very Easy

3 Easy

4 Hard

5 Very hard

8 Refuse to Answer

If B588 is equal to 4 or B588 is equal to 5 then skip to B589

If not, then skip to B591.

B589. What was the main reason it was hard to get new, unused syringes when you were in Mexico? (Choose one)

01 I didn't know where to get them

02 They cost too much/ No money to buy them

03 No place to legally buy them / store won't sell to me

04 No syringe exchange program nearby

05 I am worried about getting caught / arrested by police with syringes

06 Pharmacy/store closed

07 Other

97 Don't Know

98 Refuse to Answer

If B589 is not equal to 7, then skip to B591.

B590. Please specify the main reason why it was hard to get new/unused syringes when you were in Mexico.

____

B591. Have you got any help with injecting drugs, from someone (like a hit doctor), when you were in Mexico?

1 Yes

0 No

8 Refuse to Answer

If B591 is not equal to 1, then skip to B594.

B592. When was the last time someone helped you inject drugs when you were in Mexico?

___ MONTHS

___ WEEKS

___ DAYS

998 Refuse to Answer (Months)

If B592 is less than LSTMX then READ: "The response is smaller than the last time participant went to Mexico. Please check the response." and skip to B592.

If B592 is equal to 998 or B592 is equal to 999 or B592 is greater than 6, then skip to B594.

B593. In the last 6 months when you were in Mexico how often has someone else helped you inject? (Choose one)

1 Always

2 Often

3 Sometimes

4 Rarely

8 Refuse to Answer

B594. Please, select the type of currency you know a drug cost in Mexico. (Choose one)

1 Pesos

2 US Dollars

8 Refuse to Answer

If B594 is equal to 2 then skip to B595

If not, then skip to instruction before B610.

B595. In US dollars: How much does it cost a dose of black tar/heroin by itself in Mexico?

— — — dollars

997 Don't Know

998 Refuse to Answer

B596. In US dollars: How much does it cost a dose of powder heroin by itself in Mexico?

— — — dollars

997 Don't Know

998 Refuse to Answer

B597. In US dollars: How much does it cost a dose of Crack cocaine by itself in Mexico?

— — — dollars

997 Don't Know

998 Refuse to Answer

B598. In US dollars: How much does it cost a dose of Cocaine (powder) by itself in Mexico?

— — — dollars

997 Don't Know

998 Refuse to Answer

B599. In US dollars: How much does it cost a dose of heroin and cocaine together (speedball) in Mexico?

__ __ __	dollars
997	Don't Know
998	Refuse to Answer

B600. In US dollars: How much does it cost a dose of Methamphetamine/crystal in Mexico?

__ __ __	dollars
997	Don't Know
998	Refuse to Answer

B601. In US dollars: How much does it cost a dose of China White in Mexico?

__ __ __	dollars
997	Don't Know
998	Refuse to Answer

B602. In US dollars: How much does it cost a dose of OxyContin, without a prescription in Mexico?

__ __ __	dollars
997	Don't Know
998	Refuse to Answer

B603. In US dollars: How much does it cost a dose of Other prescription opioids w/o prescription (Vicodin, Darvon, Percocet) in Mexico?

__ __ __	dollars
997	Don't Know
998	Refuse to Answer

B604. In US dollars: How much does it cost a dose of Tranquilizers (Rivotril, Diazepam, Valium, Ativan or Restoril) in Mexico?

__ __ __	dollars
997	Don't Know
998	Refuse to Answer

B605. In US dollars: How much does it cost a dose of Barbiturates without a prescription (Amytal, Nembutal, and Seconal) in Mexico?

__ __ __	dollars
997	Don't Know
998	Refuse to Answer

B606. In US dollars: How much does it cost a dose of Fentanyl in Mexico?

__ __ __	dollars
997	Don't Know
998	Refuse to Answer

B607. Do you know the cost in dollars of Another drug or combination that was not already mentioned in Mexico?

1	Yes
0	No
8	Refuse to Answer

If B607 is not equal to 1, then skip to instruction before B610.

B608. In US dollars: How much does it cost a dose of Another drug or combination that was not already mentioned in Mexico?

__ __ __	dollars
997	Don't Know
998	Refuse to Answer

If B608 is greater than 0 and B608 is not equal to 998 then skip to B609

If not, then skip to instruction before B610.

B609. Another drug or combination you named a cost for earlier.

____

If B594 is equal to 1 then skip to B610

If not, then skip to B625.

B610. In pesos: How much does it cost a dose of black tar/heroin by itself in Mexico?

__ __ __	pesos
997	Don't Know
998	Refuse to Answer

B611. In pesos: How much does it cost a dose of powder heroin by itself in Mexico?

__ __ __	pesos
997	Don't Know
998	Refuse to Answer

B612. In pesos: How much does it cost a dose of Crack cocaine by itself in Mexico?

__ __ __	pesos
997	Don't Know
998	Refuse to Answer

B613. In pesos: How much does it cost a dose of Cocaine (powder) by itself in Mexico?

__ __ __	pesos
997	Don't Know
998	Refuse to Answer

B614. In pesos: How much does it cost a dose of heroin and cocaine together (speedball) in Mexico?

___ ___ ___	pesos
997	Don't Know
998	Refuse to Answer

B615. In pesos: How much does it cost a dose of Methamphetamine/crystal in Mexico?

___ ___ ___	pesos
997	Don't Know
998	Refuse to Answer

B616. In pesos: How much does it cost a dose of China White in Mexico?

___ ___ ___	pesos
997	Don't Know
998	Refuse to Answer

B617. In pesos: How much does it cost a dose of OxyContin, without a prescription in Mexico?

___ ___ ___	pesos
997	Don't Know
998	Refuse to Answer

B618. In pesos: How much does it cost a dose of Other prescription opioids w/o prescription (Vicodin, Darvon, Percocet) in Mexico?

___ ___ ___	pesos
997	Don't Know
998	Refuse to Answer

B619. In pesos: How much does it cost a dose of Tranquilizers (Rivotril, Diazepam, Valium, Ativan or Restoril) in Mexico?

__ __ __	pesos
997	Don't Know
998	Refuse to Answer

B620. In pesos: How much does it cost a dose of Barbiturates without a prescription (Amytal, Nembutal, and Seconal) in Mexico?

__ __ __	pesos
997	Don't Know
998	Refuse to Answer

B621. In pesos: How much does it cost a dose of Fentanyl in Mexico?

__ __ __	pesos
997	Don't Know
998	Refuse to Answer

B622. Do you know the cost in pesos of Another drug or combination that was not already mentioned in Mexico?

1	Yes
0	No
8	Refuse to Answer

If B622 is not equal to 1, then skip to B625.

B623. In pesos: How much does it cost a dose of Another drug or combination that was not already mentioned in Mexico?

__ __ __	pesos
997	Don't Know
998	Refuse to Answer

If B623 is greater than 0 and B623 is not equal to 998 then skip to B624

If not, then skip to B625.

B624. Another drug or combination you named a cost for earlier.

____

INJECTION INITIATION QUESTIONS

B625. Have you ever helped someone inject who had never injected before?

1 Yes

0 No

8 Refuse to Answer

If B625 is not equal to 1, then skip to instruction before B641.

B626. How many different people? __ __ __ __

9998 Refuse to Answer

B627. What were the reasons why you helped someone inject who had never injected before? (Check all that apply)

__ They shared their drugs with me

__ They paid me to help them inject

__ I wanted to share the experience

__ They didn't know how to inject

__ They were too shaky/nervous to inject

__ Other

__ Refuse to Answer

If B627F is not equal to 1, then skip to B629.

B628. Please specify other reason.

__ __

B629. Who have you ever helped inject for the first time? (Check all that apply)

__	Sibling
__	Casual sex partner
__	Paid sex partner (I paid them)
__	Boyfriend, Girlfriend, or Partner
__	Son or daughter
__	Someone to whom I sold drugs to
__	Someone to whom I sold syringes to
__	Friend
__	Acquaintance
__	Stranger
__	Inmate
__	Other
__	Refuse to Answer

If B629L is not equal to 1, then skip to B631.

B630. Please specify other relationship.

____

B631. In the last 6 months, have you helped anybody inject who had never injected before?

1	Yes
0	No
8	Refuse to Answer

If B631 is not equal to 1, then skip to instruction before B641.

B632. How many different people?

__ __ __ __	people
9998	Refuse to Answer

If B632 is greater than B626 and B632 is not equal to 9998 then READ: "The response is bigger than the total number. Please check the response." and skip to B632.

B633. How many of these people you helped lived in Mexico? __ __ __ __ people

9998 Refuse to Answer

If B633 is greater than B632 and B633 is not equal to 9998 then READ: "The response is bigger than the total number. Please check the response." and skip to B633.

B634. How many of these people you helped lived in the US? __ __ __ __ people

9998 Refuse to Answer

If B633 is not equal to 9998 and B634 is not equal to 9998 and B633 + B634 is greater than B632 then READ: "The summ of two responses is bigger than the total number. Please check the responses." and skip to B634.

B635. What was the gender of these people? (Check all that apply)

__ Male

__ Female

__ Transgender

__ Refuse to Answer

B636. What were the reasons why, in the last six months, you helped someone inject who had never injected before?
Interviewer: Read out the options and check all that apply (Check all that apply)

__ They shared their drugs with me

__ They paid me to help them inject

__ I wanted to share the experience

__ They didn't know how to inject

__ They were too shaky/nervous to inject

__ Other

__ Refuse to Answer

If B636F is not equal to 1, then skip to B638.

B637. Please specify other reason(s).

__ __

B638. In the last six months, who have you helped inject for the first time? (Check all that apply)

__ Sibling

__ Casual sex partner

__ Paid sex partner (I paid them)

__ Boyfriend, Girlfriend, or Partner

__ Son or daughter

__ Someone to whom I sold drugs to

__ Someone to whom I sold syringes to

__ Friend

__ Acquaintance

__ Stranger

__ Inmate

__ Other

__ Refuse to Answer

If B638L is not equal to 1, then skip to B640.

B639. Please specify other relationship.

____

B640. In the last six months, did you share syringes with someone you helped inject who had never injected before?

1 Yes

0 No

8 Refuse to Answer

READ: INTERVIEWER READ TO THE RESPONDENT: The next few questions are about how able people are to practice certain behaviors. Please answer in the way that best fits you.

B641. I can avoid injecting with a needle someone else used, even if I am injecting with people I know well. (Choose one)

1 Absolutely sure I CANNOT

2 Pretty sure I CANNOT

3 Pretty sure I CAN

4 Absolutely sure I CAN

8 Refuse to Answer

B642. I can avoid injecting with a needle someone else used even if I am dope sick or in withdrawal. (Choose one)

1 Absolutely sure I CANNOT

2 Pretty sure I CANNOT

3 Pretty sure I CAN

4 Absolutely sure I CAN

8 Refuse to Answer

B643. I can avoid using cookers, cotton, or rinse water that someone else used, even if I am injecting with people I know well. (Choose one)

1 Absolutely sure I CANNOT

2 Pretty sure I CANNOT

3 Pretty sure I CAN

4 Absolutely sure I CAN

8 Refuse to Answer

B644. I can avoid using my injecting partner's needle, even if we have shared needles before. (Choose one)

1 Absolutely sure I CANNOT

2 Pretty sure I CANNOT

3 Pretty sure I CAN

4 Absolutely sure I CAN

8 Refuse to Answer

B645. I can avoid using my injecting partner's cooker, cotton, or rinse water, even if we've shared them before. (Choose one)

1 Absolutely sure I CANNOT

2 Pretty sure I CANNOT

3 Pretty sure I CAN

4 Absolutely sure I CAN

8 Refuse to Answer

B646. I can avoid injecting with a needle someone else used, even if I have had sex without condoms with that person. (Choose one)

1 Absolutely sure I CANNOT

2 Pretty sure I CANNOT

3 Pretty sure I CAN

4 Absolutely sure I CAN

8 Refuse to Answer

OVERDOSE EXPERIENCE

READ: INTERVIEWER READ TO THE RESPONDENT: The next few questions are about drug overdose. When I say overdose, I mean a situation where you passed out and could not wake up or your lips turned blue.

B647. How many times in your life have you overdosed?

__ __ __	times	
997	Don't Know	***Skip to instruction before Q1***
998	Refuse to Answer	***Skip to instruction before Q1***

If B647 is equal to 0, then skip to B677.

If NDT_TJ is equal to 1, then skip to instruction before B649.

B648. Of those times that you overdosed, How many times did it happen in the US?

__ __ __	times	
997	Don't Know	***Skip to instruction before Q1***
998	Refuse to Answer	***Skip to instruction before Q1***
999	Not Applicable	***Skip to instruction before Q1***

If B648 is greater than B647 and B648 is not equal to 997 and B648 is not equal to 998 and B648 is not equal to 999 then READ: "That response is greater than the number of overdoses in the participant's lifetime. Please check the answer." and skip to B648.

If NDT_SD is equal to 1, then skip to B650.

B649. Of those times that you overdosed, How many times did it happen in Mexico?

__ __ __	times	
997	Don't Know	***Skip to instruction before Q1***
998	Refuse to Answer	***Skip to instruction before Q1***
999	Not Applicable	***Skip to instruction before Q1***

If B649 is greater than B647 and B649 is not equal to 997 and B649 is not equal to 998 and B649 is not equal to 999 then READ: "That response is greater than the number of overdoses in the participant's lifetime. Please check the answer." and skip to B649.

B650. In the last 6 months, how many times have you overdosed?

__ __	times	
97	Don't Know	***Skip to instruction before Q1***
98	Refuse to Answer	***Skip to instruction before Q1***

If B650 is greater than B647 and B650 is not equal to 97 and B650 is not equal to 98 then READ: "That response is greater than the number of overdoses in the participant's lifetime. Please check the answer." and skip to B650.

If B650 is equal to 0, then skip to instruction before B659.

B651. What city/region were you in when your most recent overdose occurred? (Choose one)

1	San Diego County
2	Tijuana
3	Other city in the US
4	Other city in Mexico
5	Other country (not the US or Mexico)
7	Don't Know
8	Refuse to Answer

If B651 is equal to 5 then skip to B652

If not, then skip to B653.

B652. Please specify what other country (not the US or Mexico) did your most recent overdose occurred.

____

B653. What drugs were you using on the day of your **last** overdose? (Check all that apply)

__ Alcohol

__ Marijuana

__ Heroin/black tar by itself

__ Powder heroin

__ Crack cocaine by itself

__ Cocaine(powder) by itself

__ Heroin and cocaine together (speedball)

__ Methamphetamine/crystal by itself

__ Methamphetamine/crystal and heroin together

__ China White by itself

__ China White and heroin together

__ China White and fentanyl together

__ China White and crystal/meth together

__ PCP

__ Hallucinogenic drugs like acid, LSD, peyote, mescaline, mushrooms

__ Inhalants (for example, glue, gasoline, paint thinner, agua celeste (heavenly water) or other chemicals)

__ Tranquilizers without a prescription (Rivotril, Diazepam, Valium, Ativan or Restoril)

__ OxyContin, without a prescription

__ Other prescription opioids without a prescription (Vicodin, Darvon, Percocet)

__ Barbiturates without a prescription (Amytal, Nembutal, and Seconal)

__ Fentanyl and cocaine together

__ Fentanyl and heroin together

__ Fentanyl and Methamphetamine/crystal together

__ Another drug or combination that was not already mentioned

__ Don't Know

__ Refuse to Answer

If B653X is equal to 1 then skip to B654

If not, then skip to B655.

B654. Please specify what other drug or combination of drugs you were using on the day of your last overdose.

____

B655. Which of the drugs that you used on the day of your last overdose did you inject? (Check all that apply)

__ Heroin/black tar by itself

__ Powder heroin

__ Crack cocaine by itself

__ Cocaine(powder) by itself

__ Heroin and cocaine together (speedball)

__ Methamphetamine/crystal by itself

__ Methamphetamine/crystal and heroin together

__ China White by itself

__ China White and heroin together

__ China White and fentanyl together

__ China White and crystal/meth together

__ PCP

__ Hallucinogenic drugs like acid, LSD, peyote, mescaline, mushrooms

__ Inhalants (for example, glue, gasoline, paint thinner, agua celeste or other chemicals)

__ Tranquilizers without a prescription (Rivotril, Diazepam, Valium, Ativan or Restoril)

__ OxyContin, without a prescription

__ Other prescription opioids without a prescription (Vicodin, Darvon, Percocet)

__ Barbiturates without a prescription (Amytal, Nembutal, and Seconal)

__ Fentanyl and cocaine together

__ Fentanyl and heroin together

__ Fentanyl and Methamphetamine/crystal together

__ Fentanyl and China White

__ Another drug or combination that was not already mentioned

__ Don't Know

__ Refuse to Answer

If B655W is equal to 1 then skip to B656

If not, then skip to B657.

B656. Please specify the other drug/drugs combination that you injected on the day of your last overdose.

____

B657. In the last 30 days prior to the overdose you just described, had you been in custody (prison, jail, detention center) for 3 or more days?

1 Yes

0 No

8 Refuse to Answer

B658. In the last 30 days prior to the overdose you just described, had you stopped using drugs for 3 or more days because you were in drug treatment or for some other reason?

1 Yes

0 No

8 Refuse to Answer

READ: INTERVIEWER READ TO THE RESPONDENT: Naloxone is a medicine that can be used to reverse an overdose.

B659. Have you ever heard of naloxone before today? (Choose one)

1 Yes

2 No

3 Unsure

8 Refuse to Answer

If B659 is not equal to 1, then skip to B676.

B660. Have you ever had to use Naloxone on yourself? (Choose one)

1	Yes
2	No
3	Unsure
8	Refuse to Answer

If B660 is not equal to 1, then skip to B662.

B661. How many times did you have to use Naloxone on yourself in the last 6 months?

— — —

998	Refuse to Answer

B662. Has anyone else ever had to use Naloxone on you? (Choose one)

1	Yes
2	No
3	Unsure
8	Refuse to Answer

If B662 is not equal to 1, then skip to B664.

B663. How many times has anyone else had to use Naloxone on you in the last 6 months?

— — —

998	Refuse to Answer

B664. Have you ever had to use Naloxone on someone else? (Choose one)

1	Yes
2	No
3	Unsure
8	Refuse to Answer

If B664 is not equal to 1, then skip to B666.

B665. How many times did you have to use Naloxone on someone else in the last 6 months?

— — —

998	Refuse to Answer

B666. Do you have naloxone on you right now?

1	Yes
0	No
8	Refuse to Answer

B667. Where did you get the last Naloxone? (Choose one)

01	Needle Exchange Program
02	On Point
03	PrevenCasa
04	Community Health Clinic
05	Pharmacy
06	Drug treatment program
07	N/A (never got naloxone)
08	Other
98	Refuse to Answer

If B667 is not equal to 8, then skip to B669.

B668. Please specify where did you get Naloxone

____

B669. How did you pay for the last Naloxone you got? (Choose one)

1	In US Dollars
2	In Pesos
3	In other form of payment
4	Did not pay (Zero)
8	Refuse to Answer

If B669 is not equal to 1, then skip to instruction before B671.

B670. How much did you pay for the last Naloxone in US Dollars?

__ __ __	Dollars
998	Refuse to Answer

If B669 is not equal to 2, then skip to instruction before B672.

B671. How much did you pay for the last Naloxone in Pesos? __ __ __ __ __ Pesos

99998 Refuse to Answer

If B669 is not equal to 3, then skip to B673.

B672. Please specify other form of payment

____

B673. In the past 6 months, how often have you had naloxone on <u>you</u> where you could get it within 5 minutes? (Choose one)

1 Always

2 Mostly

3 Sometimes

4 Never

8 Refuse to Answer

B674. In the past 6 months, how often have you injected around <u>other people</u> who had naloxone that they could give to you within 5 minutes? (Choose one)

1 Always

2 Mostly

3 Sometimes

4 Never

8 Refuse to Answer

B675. Do you know where you could go to get free naloxone <u>today</u>? (Choose one)

1 Yes

2 No

3 Unsure

8 Refuse to Answer

B676. In the last 6 months, have you had an abscess (cuerazo) at a location on your body where you injected drugs?

1	Yes
0	No
8	Refuse to Answer

B677. What is the primary kind of health insurance or health care plan that you have now? (Choose one)

0	I do not have health insurance
1	Private (purchased directely or through employment)
2	Public (e.g., Medicare, Medicaid, Tricare)
7	Don't Know
8	Refuse to Answer

B678. Did you lose health coverage because of the COVID-19 pandemic?

1	Yes
0	No
7	Don't Know
8	Refuse to Answer

B679. Do you have a disability that interferes with your ability to carry out daily activities? Examples of daily activities include walking, climbing stairs, shopping, balancing a checkbook, bathing or dressing.

1	Yes
0	No
8	Refuse to Answer

DRUG REHAB EXPERIENCE

READ: INTERVIEWER READ TO THE RESPONDENT: Now, I am going to ask you some questions about getting help for your drug use.

B680. To what extent would you say that you currently need help for your drug use? (Choose one)

0	No need
1	Some need
2	Great need
3	Urgent need
8	Refuse to Answer

B681. Have you ever received <u>any type of professional help</u> for your use of alcohol or drugs? By help I mean a rehabilitation center, methadone or other medication use, or any other type of program or meeting that helps you reduce or stop your alcohol or drug use.

1	Yes
0	No
8	Refuse to Answer

If B681 is not equal to 1, then skip to instruction before B770.

B682. For which of the following, you received professional help? (Choose one)

1	Drugs only
2	Alcohol only
3	Both, drugs and alcohol
8	Don't Know
9	Refuse to Answer

If B682 is equal to 1 or B682 is equal to 3 then skip to B683

If not, then skip to instruction before B686.

B683. How many times in your life have you received any type of professional help for your use of drugs?

__ __ # of Times

97 Don't Know

98 Refuse to Answer

B684. Of those [Response to B683] times, how many times did you receive help in the U.S. for your use of drugs?

__ __ # of Times

98 Refuse to Answer

If B684 is greater than B683 and B684 is not equal to 98 then READ: "That response is greater than the total number of times that the participant received professional help for drug use." and skip to B684.

B685. Of those [Response to B683] times, how many times did you receive help in Mexico for your use of drugs?

__ __ # of Times

98 Refuse to Answer

If B685 is greater than B683 and B685 is not equal to 98 then READ: "That response is greater than the total number of times that the participant received professional help for drug use." and skip to B685.

If B682 is equal to 2 or B682 is equal to 3 then skip to B686

If not, then skip to B689.

B686. How many times in your life have you received any type of professional help for your use of alcohol?

__ __ # of Times

-1 Don't Know

98 Refuse to Answer

B687. Of those [Response to B686] times, how many times did you receive help in the U.S. for your use of alcohol?

__ __ # of Times

98 Refuse to Answer

If B687 is greater than B686 and B687 is not equal to 98 then READ: "That response is greater than the total number of times that the participant received professional help for alcohol use." and skip to B687.

B688. Of those [Response to B686] times, how many times did you receive help in Mexico for your use of alcohol?

__ __	# of Times
98	Refuse to Answer

If B688 is greater than B686 and B688 is not equal to 98 then READ: "That response is greater than the total number of times that the participant received professional help for alcohol use." and skip to B688.

B689. Have you ever enrolled in a rehabilitation center? By rehabilitation center, I mean a place where you went and stayed for help with your drug or alcohol problems.

1	Yes
0	No
8	Refuse to Answer

If B689 is not equal to 1, then skip to B720.

B690. How many times have you enrolled in a rehabilitation center?

__ __	# of Times
98	Refuse to Answer

If B690 is greater than B683 + B686 and B690 is not equal to 98 then READ: "That response is greater than the total number of times that the participant received professional help for drug use." and skip to B690.

READ: INTERVIEWER READ TO THE RESPONDENT: Now I would like to ask you some questions about your experiences in the rehabilitation centers that you have gone to.

B691. What was the name of the most recent rehab/drug treatment center in which you enrolled?

____

B692. In what city was this rehab/drug treatment center is located? (Choose one)

1 San Diego County

2 Tijuana

3 Other city in the US

4 Other city in Mexico

5 Other

8 Refuse to Answer

If B692 is equal to 5 then skip to B693

If not, then skip to B694.

B693. Please specify the city where this rehab/drug treatment center located

____

B694. When did you enter this rehab/drug treatment center the last time? (Choose one)

1 less than one month ago

2 1-6 months ago

3 > 6 months ago

8 Refuse to Answer

If B694 is equal to 3, then skip to B720.

B695. What type of program was it? (Choose one)

1 Ayuda mutua or 12-step meetings
2 Faith/ Religious based
3 Government-run institution
4 Private Hospital institution
5 Methadone/Buprenorphine/Suboxone clinic
6 Other
7 Don't Know
8 Refuse to Answer

If B695 is equal to 6 then skip to B696

If not, then skip to B697.

B696. Please specify the type of rehab/drug treatment program.

____

B697. Was there a cost to enroll in the rehab/drug treatment program?

1 Yes
0 No
8 Refuse to Answer

If B697 is not equal to 1, then skip to B703.

B698. Please, select the type of currency you know the rehab program costs. (Choose one)

1 Pesos
2 US Dollars
8 Refuse to Answer
9 Not Applicable

If B698 is equal to 2 then skip to B699

If not, then skip to instruction before B701.

B699. In dollars: What was the cost of the rehab/drug program? __ __ __ __ __ dollars

99998 Refuse to Answer

B700. Please, specify the pay period of the rehab program the cost like per day, week, or month. (Choose one)

1 per day

2 per week

3 per month

8 Refuse to Answer

If B698 is equal to 1 then skip to B701

If not, then skip to B703.

B701. In pesos: What was the cost of the rehab/drug program? __ __ __ __ __ pesos

99998 Refuse to Answer

B702. Please, specify the pay period of the rehab program the cost like per day, week, or month. (Choose one)

1 per day

2 per week

3 per month

8 Refuse to Answer

B703. Are you still enrolled in a drug treatment program? 1 Yes

0 No

8 Refuse to Answer

B704. Which drugs did you go to get help for at this most recent rehab center? (Check all that apply)

__ Alcohol

__ Marijuana

__ Heroin

__ Crack cocaine

__ Cocaine (powder)

__ Methamphetamine/crystal

__ China White

__ PCP

__ Hallucinogenic drugs like acid, LSD, peyote, mescaline, mushrooms

__ Inhalants (for example, glue, gasoline, paint thinner, agua celeste or other chemicals)

__ Tranquilizers without a prescription (Rivotril, Diazepam, Valium, Ativan or Restoril)

__ OxyContin, without a prescription

__ Other prescription opioids without a prescription (Vicodin, Darvon, Percocet)

__ Barbiturates without a prescription (Amytal, Nembutal, and Seconal)

__ Fentanyl

__ Another drug or combination that was not already mentioned

__ Refuse to Answer

If B704P is equal to 1 then skip to B705

If not, then skip to B706.

B705. Please specify other drug(s) or drug combination(s) that you sought help for.

____

B706. How long did you stay at this most recent rehab center?

__ __ __	YEARS
__ __ __	MONTHS
__ __ __	DAYS
997	Don't Know (Months)
998	Refuse to Answer (Months)

If Trunc(B706/12) is greater than A1 then READ: "The response must be less than the participant's age. Please check the response." and skip to B706.

B707. Did you go voluntarily to this most recent rehab center?

1	Yes
0	No
8	Refuse to Answer

If B707 is equal to 0 then skip to B708

If not, then skip to instruction before B709.

B708. Who made you go to this most recent rehab center? (Check all that apply)

- __ Parents
- __ Steady partner
- __ Children
- __ Siblings
- __ Other family
- __ Judge - because this was the third time the report was filed with the health authority.
- __ Judge - not related to the three report rule.
- __ Police raid
- __ City workers clearing homeless camps
- __ Social worker
- __ Other
- __ Refuse to Answer

If B708K is not equal to 1, then skip to instruction before B710.

B709. Please specify the other person who made you go to this most recent rehab center:

____

If B707 is equal to 1 then skip to B710

If not, then skip to instruction before B711.

B710. What are all the reasons that you decided to enroll in this most recent rehab center? (Check all that apply)

__ I had a bad withdrawal

__ I was tired of using/wanted to do more with my life

__ I wanted to avoid getting arrested

__ I was given a choice between treatment and incarceration, so I chose treatment

__ I was forced by law enforcement officials

__ My partner encouraged me to go

__ My partner was getting clean too

__ I was pregnant/had a child

__ My kids were taken away

__ Encouragement from family

__ I was sick and needed medical attention

__ I had mental health issues

__ I overdosed

__ Religious reasons

__ I couldn't afford my drug use anymore

__ Other

__ Refuse to Answer

If B710P is not equal to 1, then skip to B712.

B711. Please specify the other reason(s) why you enrolled in this most recent rehab center.

____

B712. Did you receive medication to detox at this most recent rehab center?

1 Yes

0 No

8 Refuse to Answer

If B712 is equal to 1 then skip to B713

If not, then skip to B715.

B713. What medications did you receive at this most recent rehab center? (Check all that apply)

__ Rivotril

__ Darvon

__ Methadone

__ Buprenorphine/Suboxone

__ Naltrexone

__ Modafinil

__ Other

__ Don't Know

__ Refuse to Answer

If B713G is equal to 1 then skip to B714

If not, then skip to B715.

B714. Please specify the other medication(s) that you received.

____

B715. What services did you have access to at this most recent rehab center?
Interviewer: Read out the options and check all that apply

(Check all that apply)

__ Ayuda mutua or 12-step meetings

__ Individual psychological counseling

__ Family psychological counseling

__ Spiritual counseling

__ HIV testing

__ STI testing

__ HCV testing

__ COVID-19 testing

__ Medical treatment from a doctor

__ Legal services

__ Health education

__ Clothing and other personal items

__ Job training or skill development

__ Other

__ Refuse to Answer

If B715N is equal to 1 then skip to B716

If not, then skip to B717.

B716. Please specify what other services you had access to.

____

B717. The last time you were in drug treatment were you mistreated by any of the staff?

1	Yes
0	No
8	Refuse to Answer

If B717 is equal to 1 then skip to B718

If not, then skip to instruction before B719.

B718. What kinds of mistreatment did you experience from the staff?
Interviewer: <u>Do not read out list</u>. Mark all that respondent says
(Check all that apply)

—	Verbal abuse, [illegible]
—	Lack of respe[illegible]
—	Mental/psych[illegible]
—	Physical abus[illegible]
—	Physically res[illegible]
—	Prevented fro[illegible]
—	Physical puni[illegible]
—	Sexual abuse [illegible]
—	Refused to pr[illegible]
—	Had to work [illegible]
—	Was robbed [illegible]
—	Wasn't allowe[illegible]
—	Didn't have e[illegible]
—	Was sick, but [illegible]
—	Other
—	Refuse to An[illegible]

If B718O is equal to 1 then skip to B719

If not, then skip to B720.

B719. Please specify the other kind of mistreatment that you experienced.

____

METHADONE EXPERIENCES

B720. Have you ever been enrolled in a methadone program?
INTERVIEWER: If participant says Do not know, read: Methadone is an oral replacement therapy, which means the medication is given to you orally to help you deal with your addiction.

1	Yes
0	No
8	Refuse to Answer

If B720 is equal to 0 then skip to B721

If not, then skip to instruction before B723.

B721. Why have you never been enrolled in a methadone program? (Choose one)

01	It does not work
02	I didn't want my family to find out I was addicted
03	I relapsed
04	It is too expensive
05	It is a hassle to go to the clinic everyday
06	The clinic was too far away
07	It causes bad withdrawal (malilla)
08	The dose is too strong
09	The dose is too weak
10	I still want to use, I do not see the point
11	It is more addictive
12	No one recommended it to me
13	I do not know what methadone is
14	Other
98	Refuse to Answer

If B721 is not equal to 14, then skip to instruction before B723.

B722. Please, specify other reason.

____

If B720 is not equal to 1, then skip to B745.

B723. How many times in your life have you started a methadone program?
INTERVIEWER: If participant says Do not know, ask them for their best guess or estimate

__ __	times
98	Refuse to Answer

If B723 is greater than B683 and B723 is not equal to 98 then READ: "That response is greater than the total number of times that the participant received professional help for drug use." and skip to B723.

B724. How old were you the first time?
INTERVIEWER: If participant says Do not know, ask them for their best guess or best estimate.

__ __	Years Old
98	Refuse to Answer

If B724 is greater than A1 and B724 is not equal to 98 then READ: "That response exceeds the participant's current age. Please check the response." and skip to B724.

B725. Are you currently enrolled in a methadone program?

1	Yes
0	No
8	Refuse to Answer

B726. When was the last time you were enrolled in a methadone program?
INTERVIEWER: If participant says Do not know, ask them for their best guess or best estimate.
(Choose one)

1	Within the past 6 months
2	Longer than 6 months ago, but within the past year
3	More than 1 year ago, but less than 3 years ago
4	Three to five years ago
5	More than 5 years ago
8	Refuse to Answer

B727. In what city was this methadone program? (Choose one)

1 San Diego County
2 Tijuana
3 Other city in the US
4 Other city in Mexico
5 Other
7 Don't Know
8 Refuse to Answer

If B727 is equal to 5 then skip to B728

If not, then skip to B729.

B728. Please specify the city where this methadone program located

____

B729. Have you ever enrolled in a Methadone treatment program that was ordered by a law enforcement official or a judge?

1 Yes
0 No
8 Refuse to Answer

If B729 is not equal to 1, then skip to B732.

B730. In what city was this methadone program located? (Check all that apply)

__ San Diego County
__ Tijuana
__ Other city in the US
__ Other city in Mexico
__ Other
__ Refuse to Answer

If B730E is equal to 1 then skip to B731

If not, then skip to B732.

B731. Please specify the city where this methadone program ordered by a judge located

____

B732. Please, select the type of currency you know the methadone program costs. (Choose one)

1	Pesos
2	US Dollars
8	Refuse to Answer
9	Not Applicable

If B732 is equal to 2 then skip to B733

If not, then skip to instruction before B735.

B733. What was the cost of the methadone program? _ _ _ _ _ $

99998	Refuse to Answer

B734. Please, specify the pay period of the methadone program the cost like per day, week, or month. (Choose one)

1	per day
2	per week
3	per month
8	Refuse to Answer

If B732 is equal to 1 then skip to B735

If not, then skip to instruction before B737.

B735. What was the cost of the methadone program? _ _ _ _ _ pesos

99998	Refuse to Answer

B736. Please, specify the pay period of the methadone program the cost like per day, week, or month. (Choose one)

1	per day
2	per week
3	per month
8	Refuse to Answer

If B725 is equal to 1 then skip to B737

If not, then skip to instruction before B738.

B737. Are you still taking methadone?

1	Yes
0	No
8	Refuse to Answer

If B737 is equal to 1 then skip to B738

If not, then skip to instruction before B740.

B738. What dosage did you start on?

__ __ __	mg
998	Refuse to Answer

B739. What dosage are you on now?

__ __ __	mg
998	Refuse to Answer

If B737 is equal to 0 then skip to B740

If not, then skip to B742.

B740. What are all the reasons that you stopped using methadone?
Interviewer: <u>Do not read out list</u>, check all that apply (Check all that apply)

__ It didn't work

__ I relapsed

__ It was too expensive

__ It was a hassle to go to the clinic everyday

__ I felt in danger using the clinic

__ The clinic was too far away

__ It gave me worse withdrawal (malilla) than Black tar

__ The dose was too strong

__ The dose was too weak

__ I did not like the staff at the clinic

__ I was still using other drugs so I didn't see the point in continuing

__ It is more addictive than Black tar/heroin

__ It was confiscated by the police

__ I am afraid of being hassled by the police for taking it

__ Other

__ Refuse to Answer

If B740O is equal to 1 then skip to B741

If not, then skip to B742.

B741. Please specify the other reason(s) that you stopped using methadone.

____

B742. How long were you enrolled in the methadone program for that last time?

___ ___ ___	MONTHS
___ ___ ___	WEEKS
___ ___ ___	DAYS
998	Refuse to Answer (Months)

If Trunc(B742/12) is greater than A1 then READ: "This response exceeds the participant's current age. Please check the response." and skip to B742.

If B723 is greater than 1 and B723 is not equal to 98 then skip to B743

If not, then skip to B744.

B743. What is the longest period of time that you've ever been enrolled in a methadone program?

___ ___ ___	MONTHS
___ ___ ___	WEEKS
___ ___ ___	DAYS
998	Refuse to Answer (Months)

If Trunc(B743/12) is greater than A1 then READ: "This response exceeds the participant's current age. Please check the response." and skip to B743.

B744. What did you think about the dosage of methadone you were given? (Choose one)

1	It was too high
2	It was too low
3	It was the right amount
7	Don't Know
8	Refuse to Answer

BUPRENORPHINE EXPERIENCES

B745. Have you ever been enrolled in a Buprenorphine program?
Interviewer, if participant says Do not know, read: Buprenorphine is an oral replacement therapy similar to methadone, which means the medication is given to you orally to help you deal with your addiction

1	Yes
0	No
8	Refuse to Answer

If B745 is equal to 0 then skip to B746

If not, then skip to instruction before B748.

B746. Why have you never been enrolled in a Buprenorphine/Suboxone program? (Choose one)

01	It does not work
02	I didn't want my family to find out I was addicted
03	I relapsed
04	It is too expensive
05	It is a hassle to go to the clinic everyday
06	The clinic was too far away
07	It causes bad withdrawal (malilla)
08	The dose is too strong
09	The dose is too weak
10	I still want to use, I do not see the point
11	It is more addictive
12	No one recommended it to me
13	I do not know what Buprenorphine/Suboxone is
14	Other
98	Refuse to Answer

If B746 is not equal to 14, then skip to instruction before B748.

B747. Please, specify other reason.

____

If B745 is not equal to 1, then skip to instruction before B770.

B748. How many times in your life have you been on Buprenorphine/Suboxone?
Interviewer, if participant says Do not know, ask them for their best guess or best estimate.

__ __ __	Times
-01	Refuse to Answer

If B748 is greater than B683 and B748 is not equal to 98 then READ: "That response is greater than the total number of times that the participant received professional help for drug use." and skip to B748.

B749. How old were you the first time you used Buprenorphine/Suboxone?
Interviewer, if participant says Do not know, ask them for their best guess or best estimate.

__ __	Years Old
98	Refuse to Answer

If B749 is greater than A1 and B749 is not equal to 98 then READ: "That response exceeds the participant's current age. Please check the response." and skip to B749.

B750. Are you currently enrolled in a Buprenorphine/Suboxone program?

1	Yes
0	No
8	Refuse to Answer

B751. When was the last time you were enrolled in a Buprenorphine/Suboxone program?
Interviewer: If participant says Do not know, ask them for their best guess or estimate (Choose one)

1 Within the past 6 months

2 Longer than 6 months ago, but within the past year

3 More than 1 year ago, but less than 3 years ago

4 Three to five years ago

5 More than 5 years ago

7 Don't Know

8 Refuse to Answer

B752. In what city was this Buprenorphine/Suboxone program located? (Choose one)

1 San Diego County

2 Tijuana

3 Other city in the US

4 Other city in Mexico

5 Other

8 Refuse to Answer

If B752 is equal to 5 then skip to B753

If not, then skip to B754.

B753. Please specify the city where this Buprenorphine/Suboxone program located

____

B754. Have you ever enrolled in a Buprenorphine/Suboxone treatment program that was ordered by a law enforcement official or a judge?

1 Yes

0 No

8 Refuse to Answer

If B754 is not equal to 1, then skip to B757.

B755. In what city was the Buprenorphine/Suboxone treatment program that was ordered by a law enforcement official or a judge located? (Check all that apply)

__ San Diego County

__ Tijuana

__ Other city in the US

__ Other city in Mexico

__ Other

__ Refuse to Answer

If B755E is equal to 1 then skip to B756

If not, then skip to B757.

B756. Please specify the city where this Buprenorphine/Suboxone program ordered by a judge located

____

B757. Please, select the type of currency you know the Buprenorphine/Suboxone program costs. (Choose one)

1 Pesos

2 US Dollars

8 Refuse to Answer

9 Not Applicable

If B757 is equal to 2 then skip to B758

If not, then skip to instruction before B760.

B758. What was the cost of the Buprenorphine/Suboxone program (in US dollars)?

— — — — —	$
99998	Refuse to Answer

B759. Please, specify the pay period of the Buprenorphine/Suboxone program the cost like per day or week or month (in US dollars). (Choose one)

1	per day
2	per week
3	per month
8	Refuse to Answer

If B757 is equal to 1 then skip to B760

If not, then skip to instruction before B762.

B760. What was the cost of the Buprenorphine/Suboxone program (in pesos)?

— — — — —	pesos
99998	Refuse to Answer

B761. Please, specify the pay period of the Buprenorphine/Suboxone program the cost like per day or week or month (in pesos). (Choose one)

1	per day
2	per week
3	per month
8	Refuse to Answer

If B750 is equal to 1 then skip to B762

If not, then skip to instruction before B763.

B762. Are you still taking Buprenorphine?

1	Yes
0	No
8	Refuse to Answer

If B762 is equal to 1 then skip to B763

If not, then skip to instruction before B765.

B763. What dosage did you start on? ___ ___ ___ mg

998 Refuse to Answer

B764. What dosage are you on now? ___ ___ ___ mg

998 Refuse to Answer

If B762 is equal to 0 then skip to B765

If not, then skip to B767.

B765. What are all the reasons that you stopped using Buprenorphine/Suboxone?
Interviewer: Do not read out list, check all that apply (Check all that apply)

__ It didn't work

__ I relapsed

__ It was too expensive

__ It was a hassle to go to the clinic everyday

__ I felt in danger using the clinic

__ The clinic was too far away

__ It gave me worse withdrawal (malilla) than Black tar

__ The dose was too strong

__ The dose was too weak

__ I did not like the staff at the clinic

__ I was still using other drugs so I didn't see the point in continuing

__ Other

__ Refuse to Answer

If B765L is equal to 1 then skip to B766

If not, then skip to B767.

B766. Please specify the other reason(s) that you stopped using Buprenorphine/Suboxone.

____

B767. How long were you enrolled in the Buprenorphine/Suboxone program for that last time?

__ __ __	MONTHS
__ __ __	WEEKS
__ __ __	DAYS
998	Refuse to Answer (Months)

If Trunc(B767/12) is greater than A1 then READ: "This response exceeds the participant's current age. Please check the response." and skip to B767.

B768. What is the longest period of time that you've ever been enrolled in a Buprenorphine/Suboxone program?
Interviewer, if participant says Do not know, ask them for their best guess or estimate

__ __ __	MONTHS
__ __ __	WEEKS
__ __ __	DAYS
998	Refuse to Answer (Months)

If Trunc(B768/12) is greater than A1 then READ: "This response exceeds the participant's current age. Please check the response." and skip to B768.

B769. What did you think about the dosage of Buprenorphine/Suboxone you were given? (Choose one)

1	It was too high
2	It was too low
3	It was the right amount
7	Don't Know
8	Refuse to Answer

If B681 is not equal to 1 then skip to B770

If not, then skip to instruction before B772.

B770. Please tell me all the reasons why you have never received any kind of help or treatment for your drug use. (Check all that apply)

__ Fear of withdrawal

__ Cost/financial

__ Did not have child care

__ Spouse or partner does not want participant to go or causes problems in relationship

__ Did not meet admission criteria

__ Dislikes center

__ Dislikes treatment regimen

__ Too many rules/restrictions at treatment center

__ Doesn't like feeling locked up

__ Job problems (like getting time off from work)

__ Too far to travel/not nearby

__ Didn't know where to go

__ Afraid of being treated badly by the staff

__ Afraid of others knowing that participant is in treatment

__ Doesn't have the willpower to stop

__ Drug use isn't a problem

__ Tried a program before but it didn't work

__ Doesn't think treatment works

__ Can cut down/quit using drugs by him/herself

__ Not ready to stop using drugs

__ Haven't thought about going to treatment

__ Happy with current lifestyle

__ Afraid for safety when accessing the program

__ Afraid of being hassled by police when accessing program

__ Refuse to Answer

B771. Do you have other reasons why you have never received any kind of help or treatment for your drug use.

1 Yes

0 No

8 Refuse to Answer

If B771 is equal to 1 then skip to B772

If not, then skip to instruction before B773.

B772. Please specify other reason(s) you have not sought help or treatment for drug use.

____

If B725 is equal to 1, then skip to instruction before B774.

B773. How likely are you to start a methadone program within the next 6 months?
INTERVIEWER: If participant says Do not know, read: Methadone is an oral replacement therapy, which means the medication is given to you orally to help you deal with your addiction.
(Choose one)

1 Very Unlikely

2 Unlikely

3 Likely

4 Very Likely

8 Refuse to Answer

If B750 is equal to 1, then skip to instruction before B775.

B774. How likely are you to start a Buprenorphine/Suboxone program within the next 6 months? **Interviewer, if participant says Do not know, read: Buprenorphine/suboxone is an oral replacement therapy similar to methadone, which means the medication is given to you orally to help you deal with your addiction** (Choose one)

1	Very Unlikely
2	Unlikely
3	Likely
4	Very Likely
8	Refuse to Answer

LAW ENFORCEMENT ENCOUNTERS

READ: INTERVIEWER READ TO THE RESPONDENT: The following questions refer to the actual laws and criminal justice experiences. Please answer the following to the best of your knowledge about the laws, regardless of whether or not you think the police follow these laws.

If NDT_SD is equal to 1 or DT_SD is equal to 1 then skip to B775

If not, then skip to instruction before B776.

B775. In your opinion, is it currently legal to carry new, unused syringes in San Diego?

1	Yes
0	No
7	Don't Know
8	Refuse to Answer

If NDT_TJ is equal to 1 or DT_SD is equal to 1 then skip to B776

If not, then skip to instruction before B777.

B776. In your opinion, is it currently legal to carry new, unused syringes in Tijuana?

1	Yes
0	No
7	Don't Know
8	Refuse to Answer

READ: INTERVIEWER READ TO THE RESPONDENT: Now I would like to ask you a few questions about encounters you have had with law enforcement. When I say law enforcement, this term includes <u>all types of municipal, state, or federal police or army officers, investigators, or prosecutors</u>. This section is just about interactions with law enforcement in Mexico or the US. I know some of the questions we will ask may be a little uncomfortable for you to answer, but the interview is confidential, and we will not give any of your personal information to anyone outside of this project. Nothing that you tell us can be traced back to you as an individual.

B777. Have you ever been stopped by law enforcement? By this, we mean anytime you were stopped by law enforcement (e.g., you were stopped and let go or you were stopped and got arrested).

1 Yes

0 No

8 Refuse to Answer

If B777 is not equal to 1, then skip to B797.

B778. In the last 6 months, Have you been stopped by law enforcement? By this, we mean anytime you were stopped by law enforcement (e.g., you were stopped and let go or you were stopped and got arrested).

1 Yes

0 No

8 Refuse to Answer

If B778 is not equal to 1, then skip to B797.

B779. In the last 6 months, in what city were you when you were stopped by law enforcement? (Check all that apply)

__ San Diego County

__ Tijuana

__ Other city in the US

__ Other city in Mexico

__ Other

__ Refuse to Answer

If B779E is equal to 1 then skip to B780

If not, then skip to B781.

B780. Please specify the city where you were stopped by law enforcement in the last 6 months

____

B781. In the last 6 months, when you were stopped by the law enforcement, which of the following occurred? (Check all that apply)

__ You were stopped and arrested

__ You were stopped and let go

__ Don't Know

__ Refuse to Answer

If B781B is equal to 1 then skip to instruction before B782

If not, then skip to B790.

READ: INTERVIEWER READ TO THE RESPONDENT: The following questions will address the times you were stopped but NOT arrested by law enforcement in the last 6 months.

B782. How many times were you stopped (but not arrested) by the law enforcement in the past 6 months? **Interviewer: If participant has difficulty calculating a response for the 6-month period, please assist by promoting for an estimate of frequency of events (times per week or month); then calculate an estimate based on frequency of encounters per month or per week (e.g. once a week multiplied by 26 weeks = 26 times, or twice per month multiplied by 6 months = 12 times)**

__ __ __ # of Times

998 Refuse to Answer ***Skip to instruction before Q1***

If B782 is equal to 998 then skip to instruction before B783

If not, then skip to instruction before B784.

READ: INTERVIEWER, if a participant refuses to answer this question, reiterate the following statement: I know some of the questions we will ask may be a little uncomfortable for you to answer, but the interview is confidential, and we will not give any of your personal information to anyone outside of this project. Nothing that you tell us can be traced back to you as an individual.

B783. How many times were you stopped (but not arrested) by the law enforcement in the past 6 months? **Interviewer: If participant has difficulty calculating a response for the 6-month period, please assist by promoting for an estimate of frequency of events (times per week or month); then calculate an estimate based on frequency of encounters per month or per week (e.g. once a week multiplied by 26 weeks = 26 times, or twice per month multiplied by 6 months = 12 times)**

__ __ __ # of Times

998 Refuse to Answer ***Skip to instruction before Q1***

STOPPED = If(B782 is not equal to 998, B782, If(B782 is equal to 998, B783, 0))

If B782 is equal to 0 or B783 is equal to 0 or B783 is equal to 998, then skip to B790.

B784. In what city were you when you were stopped (but not arrested) by law enforcement in the last 6 months? (Check all that apply)

__ San Diego County

__ Tijuana

__ Other city in the US

__ Other city in Mexico

__ Other

__ Refuse to Answer

If B784E is equal to 1 then skip to B785

If not, then skip to B786.

B785. Please specify the city where you were stopped (but not arrested) by law enforcement in the last 6 months

____

B786. Of the **[STOPPED]** times you were stopped in the last 6 months, how many times did the law enforcement demand money from you as a form of bribe?

___ Times

998 Refuse to Answer

If B786 is greater than STOPPED and B786 is not equal to 998 then READ: "The participant's response should be less than or equal to the number of times they were stopped by the law enforcement in the past 6 months. Please check the responses." and skip to B786.

B787. Of the **[STOPPED]** times you were stopped in the last 6 months, how many times did the law enforcement confiscate and never return your money or other valuables?

___ Times

998 Refuse to Answer

If B787 is greater than STOPPED and B787 is not equal to 998 then READ: "The participant's response should be less than or equal to the number of times they were stopped by the law enforcement in the past 6 months. Please check the responses." and skip to B787.

B788. Of the **[STOPPED]** times you were stopped in the last 6 months, how many times have law enforcement confiscated your clean syringes without arrest?

__ __ __	Times
998	Refuse to Answer

If B788 is greater than STOPPED and B788 is not equal to 998 then READ: "The participant's response should be less than or equal to the number of times they were stopped by the law enforcement in the past 6 months. Please check the responses." and skip to B788.

B789. Of the **[STOPPED]** times you were stopped in the last 6 months, how many times have law enforcement confiscated your used syringes without arrest?

__ __ __	# of Times
998	Refuse to Answer

If B789 is greater than STOPPED and B789 is not equal to 998 then READ: "The participant's response should be less than or equal to the number of times they were stopped by the law enforcement in the past 6 months. Please check the responses." and skip to B789.

B790. Have you ever been arrested by law enforcement?
By arrest I mean experiences when police have taken you to the police station and there was a formal record made of this event.

1	Yes
0	No
8	Refuse to Answer

If B790 is not equal to 1, then skip to B797.

B791. In what city were you when you were arrested by law enforcement? (Check all that apply)

__	San Diego County
__	Tijuana
__	Other city in the US
__	Other city in Mexico
__	Other
__	Refuse to Answer

If B791E is equal to 1 then skip to B792

If not, then skip to instruction before B793.

B792. Please specify the city where you were arrested by law enforcement

____

READ: INTERVIEWER READ TO THE RESPONDENT: Now I will ask you specifically about arrest experiences you have had.

B793. In the last 6 months, how many times were you arrested?
Interviewer: if participant has difficulty calculating a response for the 6-month period, please assist by promoting for an estimate of frequency of events (times per week or month); then calculate an estimate based on frequency of encounters per month or per week (e.g. once a week multiplied by 26 weeks = 26 times, or twice per month multiplied by 6 months = 12 times)]

___ times

998 Refuse to Answer ***Skip to instruction before Q1***

If B793 is equal to 0 or B793 is equal to 998, then skip to B797.

B794. In what city were you when you were arrested by law enforcement in the last 6 months? (Check all that apply)

__ San Diego County

__ Tijuana

__ Other city in the US

__ Other city in Mexico

__ Other

__ Refuse to Answer

If B794E is equal to 1 then skip to B795

If not, then skip to B796.

B795. Please specify the city where you were arrested by law enforcement In the last 6 months

____

B796. Of the [Response to B793] times you were arrested in the last 6 months, how many times were you sentenced to jail time?

__ __ __	times
997	Don't Know
998	Refuse to Answer

If B796 is greater than B793 and B796 is not equal to 997 and B796 is not equal to 998 then READ: "The participant's response should be less than or equal to the number of times they were arrested in the past 6 months. Please check the responses." and skip to B796.

B797. In the last 6 months how many times have you been physically beaten (hit, punched, or kicked) by anyone?

__ __ __	# of Times
998	Refuse to Answer

If B797 is equal to 0 or B797 is equal to 998, then skip to instruction before B802.

B798. Of those [Response to B797] times you were beaten in the last 6 months, how many times were you beaten by law enforcement officials?

__ __ __	# of Times
998	Refuse to Answer

If B798 is greater than B797 and B798 is not equal to 998 then READ: "The participant's response should be less than or equal to the number of times they were physically beaten (hit, punched, or kicked) by anyone in the past 6 months. Please check the responses." and skip to B798.

If B798 is greater than 0 and B798 is not equal to 998 then skip to B799

If not, then skip to B801.

B799. In what city have you been physically beaten by law enforcement officials? (Check all that apply)

__	San Diego County
__	Tijuana
__	Other city in the US
__	Other city in Mexico
__	Other
__	Refuse to Answer

If B799E is equal to 1 then skip to B800

If not, then skip to B801.

B800. Please specify the city where you have been physically beaten by law enforcement officials

____

B801. Of those [Response to B797] times you were beaten in the last 6 months, how many times were you beaten by your intimate partner?

__ __ __	# of Times
998	Refuse to Answer

If B801 is greater than B797 and B801 is not equal to 998 then READ: "The participant's response should be less than or equal to the number of times they were beaten in the past 6 months. Please check the responses." and skip to B801.

Attitudes

READ: INTERVIEWER READ TO THE RESPONDENT: The next set of questions will ask you for your opinion about a number of different scenarios in the US and in Mexico. I would like to know how likely you think it is that these things might happen to you in the future.

B802. How likely do you think it is that you could get arrested for drug possession if you are stopped by the police when you are carrying drugs in [LCOUNTRY]? (Choose one)

1 Very unlikely

2 Somewhat unlikely

3 Somewhat likely

4 Very likely

8 Refuse to Answer

B803. How likely do you think it is that you could get arrested for paraphernalia possession if you are stopped by the police when you are carrying new, unused syringes in [LCOUNTRY]? (Choose one)

1 Very unlikely

2 Somewhat unlikely

3 Somewhat likely

4 Very likely

8 Refuse to Answer

B804. How likely do you think it is that you could get arrested for paraphernalia possession if you are stopped by the police when you are carrying used syringes in [LCOUNTRY]? (Choose one)

1 Very unlikely

2 Somewhat unlikely

3 Somewhat likely

4 Very likely

8 Refuse to Answer

B805. How likely do you think it is that police would demand a bribe from you if they stop you when you are carrying new, unused syringes in [LCOUNTRY]? (Choose one)

1 Very unlikely

2 Somewhat unlikely

3 Somewhat likely

4 Very likely

8 Refuse to Answer

B806. How likely do you think it is that police would demand a bribe from you if they stop you when you are carrying used syringes in [LCOUNTRY]? (Choose one)

1 Very unlikely

2 Somewhat unlikely

3 Somewhat likely

4 Very likely

8 Refuse to Answer

B807. How likely do you think it is that you will be arrested in [LCOUNTRY] just because what you look like or what you are wearing? (Choose one)

1 Very unlikely

2 Somewhat unlikely

3 Somewhat likely

4 Very likely

8 Refuse to Answer

INCARCERATION EXPERIENCES

READ: INTERVIEWER READ TO THE RESPONDENT: Now I'm going to ask you some questions about your experiences with prisons and jails.
For the next few questions, prisons refers to federal level institutions and jails refer to state- or County-level institutions (not municipal-level) where you were held with or without sentencing.
In Mexico Centros de Readapacion Social Penitenciaria La Mesa o El Hongo and
in the US The Federal Bureau of Prisons classifies prisons into several categories: United States Penitentiaries; Federal correctional institutions; Private correctional institutions; Federal prison camps; Administrative facilities; Federal correctional complexes.

B808. Have you ever been in prison?

1 Yes

0 No

8 Refuse to Answer

If B808 is not equal to 1, then skip to B812.

B809. Since you started injecting drugs, how many times have you been in prison?

— — — times

998 Refuse to Answer

If B809 is equal to 0 or B809 is equal to 998, then skip to B811.

B810. Since you started injecting, what is the total time you have spent in prison?

— — YEARS

— — MONTHS

— — DAYS

98 Refuse to Answer (Years)

99 Not Applicable (Years)

B811. Were you in prison the first time you injected drugs?

1 Yes

0 No

8 Refuse to Answer

B812. Have you ever been in jail?

1	Yes
0	No
8	Refuse to Answer

If B812 is not equal to 1, then skip to instruction before B816.

B813. Since you started injecting drugs, how many times have you been in jail?

__ __ __	times
998	Refuse to Answer

If B813 is equal to 0 or B813 is equal to 998, then skip to B815.

B814. Since you started injecting, what is the total time you have spent in jail?

__ __	YEARS
__ __	MONTHS
__ __	DAYS
98	Refuse to Answer (Years)
99	Not Applicable (Years)

B815. Were you in jail the first time you injected drugs?

1	Yes
0	No
8	Refuse to Answer

If B811 is equal to 1 and B815 is equal to 1 then READ: "The participant indicated the first time the participant injected was in Prison. Now the participant indicated the first time was in Jail. Please, revise accordingly." and skip to B815.

If B808 is not equal to 1 and B812 is not equal to 1, then skip to instruction before B837.

READ: INTERVIEWER READ TO THE RESPONDENT: Interviewer read: Now I'm going to ask you about your jail/prison experience in the past 6 months.

B816. In the last 6 MONTHS, how many times have you been in jail/prison?

___ ___ ___	times
998	Refuse to Answer

If B816 is equal to 0 or B816 is equal to 998, then skip to instruction before B837.

B817. In what city were you in jail/prison in the last 6 months? (Check all that apply)

__	San Diego County
__	Tijuana
__	Other city in the US
__	Other city in Mexico
__	Other
__	Refuse to Answer

If B817E is equal to 1 then skip to B818

If not, then skip to B819.

B818. Please specify the city where you were in jail/prison in the last 6 months

___ ___

B819. In the last 6 MONTHS, what is the total time you spent in jail/prison?

___ ___	YEARS
___ ___	MONTHS
___ ___	DAYS
98	Refuse to Answer (Years)

READ: INTERVIEWER READ TO THE RESPONDENT: Now I'm going to ask you some questions regarding drug use during the last time you went to jail/prison, in the past 6 months.

B820. In what city was the jail/prison you were at the last time? (Choose one)

1	San Diego County
2	Tijuana
3	Other city in the US
4	Other city in Mexico
5	Other
8	Refuse to Answer

If B820 is equal to 5 then skip to B821

If not, then skip to B822.

B821. Please specify the city where you were in the jail/prison the last time

____

B822. What was the name of this jail/prison? (Choose one)

1	Otay Mesa Min Sec
2	Las Colinas
3	Donovan
4	La Mesa
5	El Hongo
6	Other
7	Don't Know
8	Refuse to Answer

If B822 is equal to 6 then skip to B823

If not, then skip to B824.

B823. Please, specify the name of other jail/prison

____

B824. The last time you were in jail/prison, did you use any drugs?

1 Yes

0 No

8 Refuse to Answer

B825. The last time you were in jail/prison, did you receive methadone, buprenorphine, or suboxone?

1 Yes

0 No

8 Refuse to Answer

If B825 is equal to 1 and B824 is equal to 0 then skip to B826

If not, then skip to instruction before B827.

B826. How long were you on this treatment?

__ __ __ MONTHS

__ __ __ WEEKS

__ __ __ DAYS

998 Refuse to Answer (Months)

If B824 is not equal to 1, then skip to instruction before B836.

B827. What drugs did you use while you were in jail/prison? (Check all that apply)

__ Alcohol

__ Marijuana

__ Heroin by itself

__ Crack cocaine by itself

__ Cocaine (powder) by itself

__ Heroin and cocaine together (speedball)

__ Methamphetamine/crystal by itself

__ Methamphetamine/crystal and heroin together

__ China White by itself

__ China White and heroin together

__ PCP

__ China White and crystal/meth together

__ Hallucinogenic drugs like acid, LSD, peyote, mescaline, mushrooms

__ Inhalants (for example, glue, gasoline, paint thinner, agua celeste or other chemicals)

__ Tranquilizers without a prescription (Rivotril, Diazepam, Valium, Ativan or Restoril)

__ OxyContin, without a prescription

__ Other prescription opioids without a prescription (Vicodin, Darvon, Percocet)

__ Barbiturates without a prescription (Amytal, Nembutal, and Seconal)

__ Fentanyl by itself

__ Fentanyl and cocaine together

__ Fentanyl and heroin together

__ Fentanyl and Methamphetamine/crystal together

__ Fentanyl and China White together

__ Another drug or combination that was not already mentioned

__ Refuse to Answer

If B827X is not equal to 1, then skip to B829.

B828. Please specify what drug(s) you used while in jail/prison

____

B829. Did you inject drugs while you were in jail/prison	1	Yes
	0	No
	8	Refuse to Answer

If B829 is not equal to 1, then skip to instruction before B836.

B830. Did you use any new syringes while you were in jail/prison?	1	Yes
	0	No
	8	Refuse to Answer

If B830 is equal to 1 then skip to B831

If not, then skip to B832.

B831. On average, how many new syringes did you get in a month while in jail/prison?

___	syringes
998	Refuse to Answer

B832. Did you use any 'used' or old needles or syringes while you were in jail/prison?

1	Yes
0	No
8	Refuse to Answer

If B832 is equal to 1 then skip to B833

If not, then skip to instruction before B836.

B833. When injecting drugs while in jail/prison, how often did you use a syringe that you knew or suspected that it had been used before by someone else? (Choose one)

1 Never

2 Sometime

3 About half of the time

4 Often

5 Always

8 Refuse to Answer

B834. Of the times you injected with other people in jail/prison, how often did you divide up drugs with somebody else by using a syringe? This is also called back loading, piggybacking or splitting drugs wet. (Choose one)

1 Never

2 Less than half of the time

3 About half of the time

4 More than half of the time

5 Always

8 Refuse to Answer

If B833 is not equal to 1 and B833 is not equal to 8 then skip to B835

If not, then skip to instruction before B836.

B835. Of the times you injected with a syringe that had been used by somebody else in jail/prison, how often did you clean it with bleach before you used it? (Choose one)

1 Never

2 Less than half of the time

3 About half of the time

4 More than half of the time

5 Always

8 Refuse to Answer

If B825 is equal to 1, then skip to instruction before B837.

B836. When you got released from jail/prison, did you receive any of the following?
Interviewer: Read out the options and check all that apply (Check all that apply)

__ Methadone, suboxone or buprenorphine (Opiate agonist therapy) (OAT)

__ Referral to a Methadone, suboxone or buprenorphine program

__ Referral to other drug treatment services

__ Naloxone/Narcan

__ Referral to a needle exchange

__ Refuse to Answer

READ: INTERVIEWER READ TO THE RESPONDENT: Now I'm going to ask you some questions about your experiences in detention centers. For the purposes of these questions, consider a detention center as a place where you can be held for up to 72 hrs or a center where migrants who have started political asylum proceedings in the US are held in Mexico until resolution of their cases (for example: Fuerzas especiales, La 20 o Carcel Municipal o Centro Municipal de Infractores, Separos de la Judicial).

READ: INTERVIEWER READ TO THE RESPONDENT: I will be asking you first about experiences in detention centers in your lifetime and then in the last 6 months

B837. Have you ever been in a detention center?

1 Yes

0 No

8 Refuse to Answer

If B837 is not equal to 1, then skip to instruction before B842.

READ: INTERVIEWER READ TO THE RESPONDENT: Now, I'm going to ask you about your experience in a detention center in the past 6 months. By detention center we mean a place where they can detain you for up to 72 hours.

B838. In the last 6 months, how many times have you been in a detention center?

___ ___ ___	times
998	Refuse to Answer

If B838 is equal to 0 or B838 is equal to 998, then skip to instruction before B842.

B839. In the last 6 months, how many days have you been in a detention center?

___ ___	days
98	Refuse to Answer

If B839 is less than 2, then skip to instruction before B842.

B840. Where was this institution located? (Check all that apply)

__	San Diego County
__	Tijuana
__	Other city in the US
__	Other city in Mexico
__	Other
__	Refuse to Answer

If B840E is not equal to 1, then skip to instruction before B842.

B841. Please specify where was this institution located.

____

If B816 is greater than 0 and B816 is not equal to 998 or B839 is greater than 0 and B839 is not equal to 998 then skip to B842

If not, then skip to instruction before B843.

B842. Have you been released from a prison, jail, or detention center in the last 30 days?

1	Yes
0	No
8	Refuse to Answer

HCV TESTING AND TREATMENT

READ: INTERVIEWER READ TO THE RESPONDENT: The following questions are related to Hepatitis C

B843. Have you ever been tested for Hepatitis C before today?

1	Yes
0	No
7	Don't Know
8	Refuse to Answer

If B843 is equal to 1 then skip to B844

If not, then skip to instruction before B845.

B844. When did you last get tested for HCV ?
Interviewer: If respondent does not reply immediately, probe until you reach an approximate date.

__ __ / __ __ / __ __ __ __	mm / dd / yyyy
2097	Don't Know (Year)
2098	Refuse to Answer (Year)
2099	Not Applicable (Year)

If B843 is equal to 0 then skip to B845

If not, then skip to instruction before B848.

B845. Compared to other drug users in this city, how likely do you think you are to get (infected with) Hepatitis C? (Choose one)

1	Much more likely
2	A bit more likely
3	About the same
4	A bit less likely
5	Much less likely
8	Refuse to Answer

B846. Why have you never had an HCV test? (Check all that apply)

__ I have never heard of Hep C

__ I did not know there was a test

__ I did not want to pay the cost of the test

__ I never, or almost never, go to clinics or hospitals

__ I did not want to know if I have HCV

__ I do not care if I have HCV

__ I do not know where to go to have an HCV test

__ Other

__ Don't Know

__ Refuse to Answer

If B846H is not equal to 1, then skip to instruction before B848.

B847. Please specify why have you never had an HCV test:

____

If B843 is equal to 1 then skip to B848

If not, then skip to B859.

B848. Where were you last tested for HCV? (Choose one)

0	As part of a research study
1	Drug treatment center
2	Private clinic/doctors office
3	Public clinic/hospital
4	Other
7	Don't Know
8	Refuse to Answer

If B848 is equal to 0 then skip to B849

If not, then skip to instruction before B850.

B849. Please specify the study:

____

If B848 is equal to 4 then skip to B850

If not, then skip to B851.

B850. Please specify the location:

____

B851. What was your last HCV test result? (Choose one)

0	Negative
1	Positive
2	Never got result
7	Don't Know
8	Refuse to Answer

If B851 is not equal to 0 then skip to B852

If not, then skip to B859.

B852. Has a doctor/ health care worker ever told you that you had HCV?

1 Yes

0 No

7 Don't Know

8 Refuse to Answer

If B852 is equal to 1 then skip to B853

If not, then skip to B859.

B853. When did you first test positive for HCV?
Interviewer: If respondent does not reply immediately, probe until you reach an approximate date.

__ __ / __ __ __ __ mm / yyyy

2097 Don't Know (Year)

2098 Refuse to Answer (Year)

B854. Has a doctor/ health care worker ever offered you treatment for HCV?

1 Yes

0 No

7 Don't Know

8 Refuse to Answer

If B854 is not equal to 1, then skip to B859.

B855. Have you ever taken medicines given to you by a doctor to prevent you from getting sick from HCV?

1 Yes

0 No

7 Don't Know

8 Refuse to Answer

If B855 is not equal to 1, then skip to B859.

B856. Did you finish your treatment?

1 Yes

0 No

7 Don't Know

8 Refuse to Answer

If B856 is not equal to 0, then skip to B859.

B857. Why did you not finish your treatment?
Interviewer: Read out the options and check all that apply
(Check all that apply)

__ I feel good/healthy

__ I have other medical conditions

__ I don't have insurance

__ I don't have money

__ I'm doing drugs/drinking

__ I missed my appointment

__ My doctor prescribed some pills but I refused

__ My doctor said I don't need any medicines

__ I stopped seeing my doctor/ never went back

__ I have no doctor/not in care

__ I didn't know there is a treatment available

__ I didn't want to get injections

__ Other

__ Don't Know

__ Refuse to Answer

If B857M is equal to 1 then skip to B858

If not, then skip to B859.

B858. Please specify the reason for not taking the medicine

____

B859. Treatments are now available that are a pill a day for 8-12 weeks which can cure >90% of people of their HCV with little to no side effects. Would you be willing to be treated?

1	Yes
0	No
7	Don't Know
8	Refuse to Answer

If B859 is equal to 0 then skip to B860

If not, then skip to instruction before B861.

B860. Can you tell me why you would not be willing to be treated for HCV?

____

HIV/AIDS

READ: INTERVIEWER READ TO THE RESPONDENT: We are doing very well. Now we are going to talk about HIV/AIDS. Im going to ask you some general questions and then some questions about your experience with HIV testing and treatment.

B861. Compared to other drug users in this city, how likely do you think you are to get (infected with) HIV/AIDS? (Choose one)

1	Much more likely
2	A bit more likely
3	About the same
4	A bit less likely
5	Much less likely
6	Not Applicable (already HIV positive)
8	Refuse to Answer

If B861 is equal to 6, then skip to B865.

B862. Have you ever been tested for HIV/AIDS before today?

1	Yes
0	No
7	Don't Know
8	Refuse to Answer

If B862 is equal to 0 then skip to B863

If not, then skip to instruction before B865.

B863. Why have you never had an HIV test?
Interviewer: Read out the options and check all that apply (Check all that apply)

__ I didn't want to pay the cost of the test

__ I never, or almost never, go to clinics or hospitals

__ I don't want to know if I have HIV/AIDS

__ I don't care if I have HIV/AIDS

__ I don't know where to go to have an HIV test

__ Other

__ Don't Know

__ Refuse to Answer

If B863F is not equal to 1, then skip to instruction before B865.

B864. Please specify why have you never had an HIV test:

____

If B862 is equal to 1 then skip to B865

If not, then skip to instruction before B870.

B865. Where were you last tested for HIV?
Interviewer: Read out the options and choose only one.
(Choose one)

0 As part of a research study

1 Drug treatment center

2 Private clinic/doctors office

3 Public clinic/hospital

4 Other

7 Don't Know

8 Refuse to Answer

If B865 is equal to 0 then skip to B866

If not, then skip to instruction before B867.

B866. Please specify the study:

____

If B865 is equal to 4 then skip to B867

If not, then skip to B868.

B867. Please specify the location:

____

B868. When were you last tested for HIV?
Interviewer: If respondent does not reply immediately, probe until you reach an approximate date.

__ __ / __ __ __ __	mm / yyyy
2097	Don't Know (Year)
2098	Refuse to Answer (Year)

B869. What was your last HIV/AIDS test result? (Choose one)

0	Negative
1	Positive
2	Never got result
7	Don't Know
8	Refuse to Answer

If B869 is equal to 1 then skip to B870

If not, then skip to instruction before B871.

B870. When did you first test positive for HIV?
Interviewer: If respondent does not reply immediately, probe until you reach an approximate date.

__ __ / __ __ __ __	mm / yyyy
2097	Don't Know (Year)
2098	Refuse to Answer (Year)

If B869 is equal to 1 or B869 is equal to 2 then skip to B871

If not, then skip to B878.

B871. Has a doctor/ health care worker ever told you that you had HIV/AIDS?

1 Yes

0 No

7 Don't Know

8 Refuse to Answer

If B871 is not equal to 1, then skip to B878.

B872. Has a doctor/ health care worker ever offered you treatment for HIV/AIDS?

1 Yes

0 No

7 Don't Know

8 Refuse to Answer

If B872 is not equal to 1, then skip to B878.

B873. Have you ever taken medicines given to you by a doctor to prevent you from getting sick from HIV/AIDS?

1 Yes

0 No

7 Don't Know

8 Refuse to Answer

If B873 is not equal to 1, then skip to B878.

B874. Are you still taking these medicines?

1 Yes

0 No

7 Don't Know

8 Refuse to Answer

If B874 is not equal to 0, then skip to B878.

B875. How long did you take these medicines for?

__ __ __	YEARS
__ __ __	MONTHS
__ __ __	DAYS
997	Don't Know (Months)
998	Refuse to Answer (Months)

B876. Why are you not taking these medicines?
Interviewer: Read out the options and check all that apply
(Check all that apply)

__	I feel good/healthy
__	I have other medical conditions
__	I don't have insurance
__	I don't have money
__	I'm doing drugs/drinking
__	I missed my appointment
__	My doctor prescribed some pills but I refused
__	My doctor said I don't need any medicines
__	I stopped seeing my doctor/ never went back
__	I have no doctor/not in care
__	I didn't want to take pills
__	The medicines made me feel unwell
__	I didnt want my family/others to know I was ill
__	I couldnt keep hold of my pills as I was moving places too frequently
__	Other
__	Don't Know
__	Refuse to Answer

If B876O is equal to 1 then skip to B877

If not, then skip to B878.

B877. Please specify the reason for not taking the medicine

____

B878. Have you known anyone personally who has HIV/AIDS?

1	Yes
0	No
7	Don't Know
8	Refuse to Answer

If B878 is equal to 1 then skip to B879

If not, then skip to instruction before B881.

B879. How many people?

__ __ __	persons
997	Don't Know
998	Refuse to Answer

B880. How many people do you personally know who have died of HIV/AIDS

__ __ __	persons
997	Don't Know
998	Refuse to Answer

PrEP SECTION

READ: INTERVIEWER READ TO THE RESPONDENT:
Pre-exposure prophylaxis or PrEP is a medicine people at risk for HIV take to prevent getting HIV from sex or injection drug use. When taken daily as prescribed, PrEP works very well to prevent HIV. PrEP is not yet widely available in Mexico, but it has been available in the US for several years. PrEP is safe, but some people experience side effects like diarrhea, nausea, and headaches. These side effects usually go away with time. PrEP does not protect against other sexually transmitted infections (STIs), so it is important to also use condoms while taking PrEP. Because PrEP does not treat HIV, it is also important to see a doctor every three months for HIV/STI testing and other laboratory tests to make sure everything is OK.

B881. Before today, had you ever heard of HIV-negative people taking HIV medications or PrEP **before** being exposed to HIV to protect against HIV infection?

1 Yes

0 No

8 Refuse to Answer

If B881 is not equal to 1, then skip to B889.

B882. Have you ever taken HIV medications or PrEP **before** being exposed to HIV to protect against HIV infection?

1 Yes

0 No

8 Refuse to Answer

If B882 is not equal to 1, then skip to B889.

B883. In what city did a doctor prescribed you PrEP therapy? (Check all that apply)

__ San Diego County

__ Tijuana

__ Other city in the US

__ Other city in Mexico

__ Other

__ Don't Know

__ Refuse to Answer

If B883E is not equal to 1, then skip to B885.

B884. Please specify the city

____

B885. Are you currently taking PrEP therapy?

1	Yes
0	No
7	Don't Know
8	Refuse to Answer

If B885 is not equal to 1, then skip to B889.

B886. When did you start your current PrEP therapy?
Interviewer: If respondent does not reply immediately, probe until you reach an approximate date.

__ __ / __ __ __ __	mm / yyyy
2097	Don't Know (Year)
2098	Refuse to Answer (Year)

B887. Which PrEP therapy are you currently taking? (Choose one)

01	Truvada (once daily, orally)
02	Maraviroc (once daily, orally)
03	Rilpivirine (once monthly, injection)
04	Dapivirine (vaginal ring worn 4 weeks at a time)
05	Tenofovir (gelinserted rectally/vaginally)
06	Cabotegravir (injectable PrEP)
07	Other
97	Don't Know
98	Refuse to Answer

If B887 is not equal to 7, then skip to B889.

B888. Please specify which other PrEP Therapy you are taking.

____

B889. What kind of PrEP product would you be interested in taking?
Interviewer: Read out the options and check all that apply (Check all that apply)

__ A pill I needed to take every day

__ An injection I needed to get every 2 months

__ (for women) A vaginal gel I needed to use before sex

__ None

__ Refuse to Answer

__ Not Applicable

If B889D is equal to 1 and B889A + B889B + B889C is greater than 0 then READ: "The participant indicated None along with other options. Please, revise the responce." and skip to B889.

READ: INTERVIEWER READ TO THE RESPONDENT: Now I'm going to ask your sexual health in the past 6 months.

B890. In the past 6 months, have you had any of the following symptoms?
Interviewer: Read out the options and check all that apply (Check all that apply)

__ Painful or very frequent urination

__ Painful sore or blisters on your genitals

__ Warts on your genitals

__ Dripping or oozing from your genitals

__ Itching in your genital area

__ Bleeding after intercourse

__ I have not had any of these symptoms

__ Don't Know

__ Refuse to Answer

If B890G is equal to 1 and B890A + B890B + B890C + B890D + B890E + B890F is greater than 0 then READ: "The participant indicated "I have not had any of these symptoms" along with other options. Please, revise the responce." and skip to B890.

B891. In the past 6 months, have you been diagnosed with any of the following health problems?
Interviewer: Read out the options and check all that apply (Check all that apply)

__ Gonorrhea (the clap or the drip)

__ Chlamydia, Non-Gonococal Urethritis (NGU), Non-specific Urethritis (NSU)

__ Syphilis (VDRL or RPR)

__ Genital or anal warts (in, on, or around the vagina or anus)

__ Genital or anal herpes (in, on, or around the vagina or anus)

__ Chancroid (sore or ulcer in or around the vagina or anus)

__ Hepatitis B

__ Hepatitis C

__ Trichomoniasis

__ Pelvic inflammatory disease

__ Vaginal candidiasis (yeast infection)

__ Cervical cancer

__ Unknown STD

__ Other female problems

__ I have not been diagnosed with any sexually transmitted disease

__ Don't Know

__ Refuse to Answer

If B891O is equal to 1 and B891A + B891B + B891C + B891D + B891E + B891F + B891G + B891H + B891I + B891J + B891K + B891L + B891M + B891N is greater than 0 then READ: "The participant indicated "I have not had any of these symptoms" along with other options. Please, revise the responce." and skip to B891.

If B891N is not equal to 1, then skip to B893.

B892. Please, specify other female problems

____

B893. In the past 6 months, did you receive treatment or medication by a doctor or nurse to treat any symptoms in your genital area?

1 Yes

0 No

8 Refuse to Answer

SEXUAL BEHAVIOR

READ: INTERVIEWER READ TO THE RESPONDENT: You are doing a great job. I will now ask you about your sexual experiences in the past 6 months. If you have been in prison/jail during this period, these questions refer to the time when you haven't been in prison/jail. Again, I know some of these questions may be uncomfortable to answer, but they will be very helpful to us in preventing diseases among you and other people like you. Please try to be as truthful and honest as you can. For these questions, when I say "sex", I mean vaginal or anal or oral sex. When we talk about using protection we mean using a male or female condom or a barrier to prevent the mixing of body fluids during sexual activity.

B894. How old were you the **first time** you had consensual anal, vaginal, or oral sex? By consensual sex, I mean you willingly agreed to have sex.
Interviewer: Please enter 0 for Never had consensual sex

__ __	years old
98	Refuse to Answer

If B894 is greater than A1 and B894 is not equal to 98 then READ: "Participant has responded with a length of time greater than his or her age. Please check the responses." and skip to B894.

If B894 is equal to 0 or B894 is equal to 98, then skip to B896.

B895. The first time you had consensual sex, was it with a male or female? (Choose one)

1	Male
2	Female
3	Both
8	Refuse to Answer

B896. Do you think of yourself as: (Choose one)

1	Heterosexual/Straight
2	Homosexual/Gay/Lesbian
3	Bisexual
4	Other
8	Refuse to Answer

If B896 is equal to 4 then skip to B897

If not, then skip to instruction before B898.

B897. Please specify other:

____

If B26 is equal to 1 then skip to instruction before B898

If not, then skip to B899.

READ: INTERVIEWER READ TO THE RESPONDENT: Next I am going to show you some pictures. This picture is of a penis that has been circumcised (the skin that covers the tip of the penis was removed,) and this one is of a penis that is uncircumcised (the skin that covers the tip is still there).

B898. Are you circumcised?

1	Yes
0	No
8	Refuse to Answer

B899. How many women have you **ever** had sex with?
Remember that, by sex, I mean vaginal, anal, or oral sex.
Interviewer, please enter 0 for Never had sex with women

__ __ __ __	women
9998	Refuse to Answer

B900. How many men have you **ever** had sex with?
Remember that, by sex, I mean vaginal, anal, or oral sex.
Interviewer, please enter 0 for Never had sex with men

__ __ __ __	men
9998	Refuse to Answer

B901. Have you had any sex during the **past 6 months**?

1	Yes
0	No
8	Refuse to Answer

If B901 is not equal to 1, then skip to B944.

B902. In what city have you had sex in the last 6 months? (Check all that apply)

__ San Diego County

__ Tijuana

__ Another location in the US

__ Another location in Mexico

__ Other

__ Don't Know

__ Refuse to Answer

If B902E is equal to 1 then skip to B903

If not, then skip to B904.

B903. Please specify the location where you had sex in the last 6 months

____

B904. During the past 6 months, have you had sex with men, women or both? (Choose one)

1 Men

2 Women

3 Both

8 Refuse to Answer

READ: INTERVIEWER READ TO THE RESPONDENT: Now I will ask you about your regular partner. By regular, we mean someone that you feel committed to above anyone else and with whom you have sex

B905. In the **past 6 months** did you have a **regular partner** that you feel committed to above anyone else and with whom you had/have sex? (If you have more than one regular partner, think about the one you have sex with the most)

1 Yes

0 No

8 Refuse to Answer

If B905 is not equal to 1, then skip to instruction before B916.

B906. What is the gender of this sex partner? (Choose one)

1 Male

2 Female

3 Transgender

8 Refuse to Answer

If B906 is not equal to 3, then skip to B908.

B907. If Transgender, select which. (Choose one)

1 Female to Male (FTM)

2 Male to Female (MTF)

8 Refuse to Answer

B908. Was this **regular partner** a client?

1 Yes

0 No

8 Refuse to Answer

If B906 is equal to 1 or B907 is equal to 2 then skip to B909

If not, then skip to B910.

B909. Is your regular partner circumcised?

1 Yes

0 No

7 Don't Know

8 Refuse to Answer

B910. What country does this person live in? (Choose one)

1 US

2 Mexico

3 Other

8 Refuse to Answer

If B910 is not equal to 3, then skip to B912.

B911. Please, specify other country

____

B912. In the past 6 months, how often did you have vaginal sex with your regular partner? (Choose one)

1 Never
2 Once or twice
3 Every few months
4 Every few weeks
5 At least once a week
6 Several times a week
8 Refuse to Answer

B913. In the past 6 months, how often did you have anal sex with your regular partner? (Choose one)

1 Never
2 Once or twice
3 Every few months
4 Every few weeks
5 At least once a week
6 Several times a week
8 Refuse to Answer

B914. In the **past 6 months** when you had sex with your **regular partner**, how often was a condom used? (Choose one)

1 Never

2 Sometimes

3 About half of the time

4 Often

5 Always

8 Refuse to Answer

B915. Did you have **UNPROTECTED** (without a condom) sex the last time you had sex with your **regular partner?**

1 Yes

0 No

8 Refuse to Answer

9 Not Applicable

READ: INTERVIEWER READ TO THE RESPONDENT: Now I will ask you about your casual partners. By casual, we mean partners with whom you have sex with but do not receive goods or services in exchange for sex, apart from your regular partner.

B916. In the **past 6 months**, how many **casual** partners did you have sex with?

— — — — partners

9998 Refuse to Answer

If B916 is equal to 0 or B916 is equal to 9998, then skip to B940.

B917. Were these **casual partners** clients that someone gave you money, drugs or something else in exchange for sex?

1 Yes

0 No

8 Refuse to Answer

B918. How many of those **casual** partners ([Response to B916]) were males?

__ __ __ __

9998 Refuse to Answer

If B918 is greater than B916 and B918 is not equal to 9998 then READ: "The participant's response should be less than or equal to the number of casual partners with whom you have sex besides regular partner. Please check the responses." and skip to B916.

If B918 is equal to 0 or B918 is equal to 9998, then skip to instruction before B922.

B919. How many male casual partners were from the US? __ __ __ __

9998 Refuse to Answer

If B919 is greater than B918 and B919 is not equal to 9998 then READ: "The participant's response should be less than or equal to the number of male casual partners with whom you have sex besides regular partner. Please check the responses." and skip to B919.

If B919 is equal to B918, then skip to instruction before B922.

B920. How many male casual partners were from Mexico? __ __ __ __

9998 Refuse to Answer

If B919 + B920 is greater than B918 and B919 is not equal to 9998 and B920 is not equal to 9998 then READ: "The participant's response should be less than or equal to the number of male casual partners with whom you have sex besides regular partner. Please check the responses." and skip to B920.

If B919 + B920 is equal to B918, then skip to instruction before B922.

B921. How many male casual partners were from other countries? __ __ __ __

9998 Refuse to Answer

If B919 + B920 + B921 is greater than B918 and B919 is not equal to 9998 and B920 is not equal to 9998 and B921 is not equal to 9998 then READ: "The participant's response should be less than or equal to the number of male casual partners with whom you have sex besides regular partner. Please check the responses." and skip to B921.

If B918 is equal to B916, then skip to instruction before B930.

B922. How many of those **casual** partners ([Response to B916]) were females?

__ __ __ __

9998 Refuse to Answer

If B918 + B922 is greater than B916 and B918 is not equal to 9998 and B922 is not equal to 9998 then READ: "The participant's response should be less than or equal to the number of casual partners with whom you have sex besides regular partner. Please check the responses." and skip to B916.

If B922 is equal to 0 or B922 is equal to 9998, then skip to instruction before B926.

B923. How many female casual partners were from the US? __ __ __ __

9998 Refuse to Answer

If B923 is greater than B922 and B923 is not equal to 9998 then READ: "The participant's response should be less than or equal to the number of female casual partners with whom you have sex besides regular partner. Please check the responses." and skip to B923.

If B923 is equal to B922, then skip to instruction before B926.

B924. How many female casual partners were from Mexico? __ __ __ __

9998 Refuse to Answer

If B923 + B924 is greater than B922 and B923 is not equal to 9998 and B924 is not equal to 9998 then READ: "The participant's response should be less than or equal to the number of female casual partners with whom you have sex besides regular partner. Please check the responses." and skip to B924.

If B923 + B924 is equal to B922, then skip to instruction before B926.

B925. How many female casual partners were from other countries? __ __ __ __

9998 Refuse to Answer

If B923 + B924 + B925 is greater than B922 and B923 is not equal to 9998 and B924 is not equal to 9998 and B925 is not equal to 9998 then READ: "The participant's response should be less than or equal to the number of female casual partners with whom you have sex besides regular partner. Please check the responses." and skip to B925.

If B918 + B922 is equal to B916, then skip to instruction before B930.

B926. How many of those **casual** partners ([Response to B916]) were transgender?

__ __ __ __

9998 Refuse to Answer

If B918 + B922 + B926 is not equal to B916 and B918 is not equal to 9998 and B922 is not equal to 9998 and B926 is not equal to 9998 then READ: "The participant's response should be less than or equal to the number of transgender casual partners with whom you have sex besides regular partner. Please check the responses." and skip to B916.

If B926 is equal to 0 or B926 is equal to 9998, then skip to instruction before B930.

B927. How many transgender casual partners were from the US? __ __ __ __

9998 Refuse to Answer

If B927 is greater than B926 and B927 is not equal to 9998 then READ: "The participant's response should be less than or equal to the number of transgender casual partners with whom you have sex besides regular partner. Please check the responses." and skip to B927.

If B927 is equal to B926, then skip to instruction before B930.

B928. How many transgender casual partners were from Mexico? __ __ __ __

9998 Refuse to Answer

If B927 + B928 is greater than B926 and B927 is not equal to 9998 and B928 is not equal to 9998 then READ: "The participant's response should be less than or equal to the number of transgender casual partners with whom you have sex besides regular partner. Please check the responses." and skip to B928.

If B927 + B928 is equal to B926, then skip to instruction before B930.

B929. How many transgender casual partners were from other countries?

__ __ __ __

9998 Refuse to Answer

If B927 + B928 + B929 is greater than B926 and B927 is not equal to 9998 and B928 is not equal to 9998 and B929 is not equal to 9998 then READ: "The participant's response should be less than or equal to the number of transgender casual partners with whom you have sex besides regular partner. Please check the responses." and skip to B929.

If B922 is greater than 0 and B922 is not equal to 9998 then skip to B930

If not, then skip to instruction before B934.

B930. In the past 6 months, how often did you have vaginal sex with your female casual partners? (Choose one)

1 Never

2 Once or twice

3 Every few months

4 Every few weeks

5 At least once a week

8 Refuse to Answer

If B930 is equal to 1 or B930 is equal to 8, then skip to B932.

B931. Did you have UNPROTECTED (without a condom) vaginal sex the last time you had sex with your female casual partner?

1 Yes

0 No

8 Refuse to Answer

9 Not Applicable

B932. In the past 6 months, how often did you have anal sex with your female casual partners? (Choose one)

1 Never

2 Once or twice

3 Every few months

4 Every few weeks

5 At least once a week

8 Refuse to Answer

If B932 is equal to 1 or B932 is equal to 8, then skip to instruction before B934.

B933. Did you have UNPROTECTED (without a condom) anal sex the last time you had sex with your female casual partner?

1 Yes

0 No

8 Refuse to Answer

9 Not Applicable

If B918 is greater than 0 and B918 is not equal to 9998 then skip to B934

If not, then skip to instruction before B938.

B934. In the past 6 months, how often did you have vaginal sex with your male casual partners? (Choose one)

1 Never

2 Once or twice

3 Every few months

4 Every few weeks

5 At least once a week

8 Refuse to Answer

If B934 is equal to 1 or B934 is equal to 8, then skip to B936.

B935. Did you have UNPROTECTED (without a condom) vaginal sex the last time you had sex with your male casual partner?

1 Yes

0 No

8 Refuse to Answer

9 Not Applicable

B936. In the past 6 months, how often did you have anal sex with your male casual partners? (Choose one)

1	Never
2	Once or twice
3	Every few months
4	Every few weeks
5	At least once a week
8	Refuse to Answer

If B936 is equal to 1 or B936 is equal to 8, then skip to instruction before B938.

B937. Did you have UNPROTECTED (without a condom) anal sex the last time you had sex with your male casual partner?

1	Yes
0	No
8	Refuse to Answer
9	Not Applicable

If B926 is greater than 0 and B926 is not equal to 9998 then skip to B938

If not, then skip to B940.

B938. In the past 6 months, how often did you have sex with your transgender casual partners? (Choose one)

1	Never
2	Once or twice
3	Every few months
4	Every few weeks
5	At least once a week
8	Refuse to Answer

If B938 is equal to 1 or B938 is equal to 8, then skip to B940.

B939. Did you have UNPROTECTED (without a condom) sex the last time you had sex with your transgender casual partner?

1 Yes

0 No

8 Refuse to Answer

9 Not Applicable

B940. In the **past 6 months**, when you had sex, how often were you drunk **within 2 hours before or during sex**? ("buzzed" on alcohol) (Choose one)

1 Never

2 Sometimes

3 About half of the time

4 Often

5 Always

8 Refuse to Answer

B941. In the **past 6 months, when you had sex,** how often had you been using drugs **within 2 hours before or during sex**? (Choose one)

1 Never

2 Sometimes

3 About half of the time

4 Often

5 Always

8 Refuse to Answer

B942. In the last 6 months, have you had sex with someone who you knew at the time had HIV/AIDS?

1 Yes

0 No

8 Refuse to Answer

If B942 is equal to 1 then skip to B943

If not, then skip to B944.

B943. How often did you use a condom? (Choose one)

1 Never

2 Sometimes

3 About half of the time

4 Often

5 Always

8 Refuse to Answer

B944. Has anyone ever given you anything you needed (such as money, drugs, alcohol, shelter, food transportation or protection) in exchange for sex?

1 Yes

0 No

8 Refuse to Answer

If B917 is equal to 1 then skip to B945

If not, then skip to instruction before B946.

B945. You said earlier that you casual partner(s) gave you money, drugs or something else in exchange for sex. Could you, please, reconsider the question: Has anyone ever given you anything you needed (such as money, drugs, alcohol, shelter, food transportation or protection) in exchange for sex?

1 Yes

0 No

8 Refuse to Answer

If B944 is not equal to 1 and B945 is not equal to 1, then skip to instruction before B973.

B946. When someone has given you anything you needed in exchange for sex, what did they give you?
Interviewer: Read out the options and check all that apply (Check all that apply)

__	Money
__	Drugs
__	Syringes
__	Alcohol
__	Shelter
__	Transportation
__	Protection
__	Food
__	A job
__	Clothes
__	Gifts
__	Other
__	Refuse to Answer

If B946L is equal to 1 then skip to B947

If not, then skip to B948.

B947. Please specify other:

____

B948. How old were you the first time someone gave you something you needed in exchange for sex?

__ __	years old
98	Refuse to Answer

If B948 is greater than A1 and B948 is not equal to 98 then READ: "Participant has responded with a length of time greater than his or her age. Please check the responses." and skip to B948.

If B901 is not equal to 1, then skip to instruction before B973.

B949. In the **last 6 months**, how many people gave you something you needed in exchange for having sex with them?

__ __ __ __	People
9998	Refuse to Answer

If B949 is equal to 0 or B949 is equal to 9998, then skip to instruction before B973.

B950. In what cities did someone give you something in exchange for sex? (Check all that apply)

__	San Diego County
__	Tijuana
__	Other city in the US
__	Other city in Mexico
__	Other
__	Refuse to Answer

If B950E is equal to 1 then skip to B951

If not, then skip to B952.

B951. Please specify the other city/country

__ __

B952. In the **last 6 months**, on average, how many times per month did someone give you something you needed in exchange for having sex with them?
Interviewer: If the participant says Do not know, ask them what is their best guess or estimate.

__ __ __	
998	Refuse to Answer

B953. Which of the following has someone given you in the **last 6 months** in exchange for having sex with them?
Interviewer: Read out the options and check all that apply (Check all that apply)

__ Money

__ Drugs

__ Syringes

__ Alcohol

__ Shelter

__ Transportation

__ Protection

__ Food

__ A job

__ Clothes

__ Gifts

__ Other

__ Refuse to Answer

If B953L is equal to 1 then skip to B954

If not, then skip to B955.

B954. Please specify other:

____

B955. In the **last 6 months**, when someone gave you something you needed in exchange for sex, how many of these people ([Response to B949]) were males?

____

9998 Refuse to Answer

If B955 is greater than B949 and B955 is not equal to 9998 then READ: "The participant's response should be less than or equal to the number of people that gave them something in exchange for sex! Please check the responses and make corrections!" and skip to B955.

If B955 is equal to 0 or B955 is equal to 9998, then skip to instruction before B959.

B956. How many males gave you something you needed in exchange for sex were from the US?

— — — —

9998 Refuse to Answer

If B956 is greater than B955 and B956 is not equal to 9998 then READ: "The participant's response should be less than or equal to the number of males gave them something they needed in exchange for sex. Please check the responses." and skip to B956.

If B956 is equal to B955, then skip to instruction before B959.

B957. How many males gave you something you needed in exchange for sex were from Mexico?

— — — —

9998 Refuse to Answer

If B956 + B957 is greater than B955 and B956 is not equal to 9998 and B955 is not equal to 9998 then READ: "The participant's response should be less than or equal to the number of males gave them something they needed in exchange for sex. Please check the responses." and skip to B957.

If B956 + B957 is equal to B955, then skip to instruction before B959.

B958. How many males gave you something you needed in exchange for sex were from other countries?

— — — —

9998 Refuse to Answer

If B956 + B957 + B958 is greater than B955 and B956 is not equal to 9998 and B957 is not equal to 9998 and B958 is not equal to 9998 then READ: "The participant's response should be less than or equal to the number of males gave them something they needed in exchange for sex. Please check the responses." and skip to B958.

If B955 is equal to B949, then skip to B963.

B959. In the **last 6 months**, when someone gave you something you needed in exchange for sex, how many of these people ([Response to B949]) were females?

— — — —

9998 Refuse to Answer

If B955 + B959 is greater than B949 and B955 is not equal to 9998 and B959 is not equal to 9998 then READ: "The participant's response should be less than or equal to the number of people gave them something in exchange for sex! Please check the responses and make corrections." and skip to B959.

If B959 is equal to 0 or B959 is equal to 9998, then skip to B963.

B960. How many females gave you something you needed in exchange for sex were from the US?

— — — —

9998 Refuse to Answer

If B960 is greater than B959 and B960 is not equal to 9998 then READ: "The participant's response should be less than or equal to the number of females gave them something they needed in exchange for sex. Please check the responses." and skip to B960.

If B960 is equal to B959, then skip to B963.

B961. How many females gave you something you needed in exchange for sex were from Mexico?

— — — —

9998 Refuse to Answer

If B960 + B961 is greater than B959 and B960 is not equal to 9998 and B959 is not equal to 9998 then READ: "The participant's response should be less than or equal to the number of females gave them something they needed in exchange for sex. Please check the responses." and skip to B961.

If B960 + B961 is equal to B959, then skip to B963.

B962. How many females gave you something you needed in exchange for sex were from other countries?

— — — —

9998 Refuse to Answer

If B960 + B961 + B962 is greater than B959 and B960 is not equal to 9998 and B961 is not equal to 9998 and B962 is not equal to 9998 then READ: "The participant's response should be less than or equal to the number of females gave them something they needed in exchange for sex. Please check the responses." and skip to B962.

B963. In the **last 6 months**, when someone has given you something you needed in exchange for sex, where did you meet or know them from?
Interviewer: Read out the options and check all that apply
(Check all that apply)

__ Street
__ Work
__ Shooting gallery
__ Neighborhood
__ School
__ Bar/club
__ Hotel
__ Newspaper/Magazine/Internet
__ Friends/ Acquaintances
__ Pimp
__ Other
__ Refuse to Answer

If B963K is not equal to 1, then skip to B965.

B964. Please, specify other

____

B965. Since the COVID epidemic began in March 2020, has the number of people who have given you something in exchange for sex changed? (Choose one)

1 More people
2 Fewer people
3 The same
8 Refuse to Answer

B966. In the **last 6 months**, how often did you have sex in exchange for something you needed? (Choose one)

1	Once or twice
2	Once or twice in a month
3	At least once a week
4	Several times a week
5	Several times a day
8	Refuse to Answer

B967. In the past 6 months, of the times you had sex in exchange for something you needed, how often did you have vaginal sex? (Choose one)

1	Never
2	Sometimes
3	About half of the time
4	Often
5	Always
8	Refuse to Answer

If B967 is equal to 1 or B967 is equal to 8, then skip to B969.

B968. Did you have UNPROTECTED (without a condom) sex the last time you had vaginal sex in exchange for something you needed?

1	Yes
0	No
8	Refuse to Answer
9	Not Applicable

B969. In the past 6 months, of the times you had sex in exchange for something you needed, how often did you have anal sex? (Choose one)

1	Never
2	Sometimes
3	About half of the time
4	Often
5	Always
8	Refuse to Answer

If B969 is equal to 1 or B969 is equal to 8, then skip to B971.

B970. Did you have UNPROTECTED (without a condom) sex the last time you had anal sex in exchange for something you needed?

1	Yes
0	No
8	Refuse to Answer
9	Not Applicable

B971. In the past 6 months, when someone has given you something you needed in exchange for sex, how frequently did you inject drugs with them? (Choose one)

1	Never
2	Sometimes
3	About half of the time
4	Often
5	Always
8	Refuse to Answer

If B971 is equal to 1 or B971 is equal to 8, then skip to instruction before B973.

B972. How frequently did you share the same syringe with them? (Choose one)

1 Never

2 Sometimes

3 About half of the time

4 Often

5 Always

8 Refuse to Answer

READ: NOTE OF CAUTION TO INTERVIEWER: It is possible that following questions could be misunderstood by some women, to where giving…in exchange for having sex with you was mistaken for receiving…in exchange for having sex with you.

B973. Have you ever ***given*** someone something **they** needed (such as money, drugs, alcohol, shelter, food transportation or protection) in exchange for sex?

1 Yes

0 No

8 Refuse to Answer

If B973 is not equal to 1, then skip to B992.

B974. Was the person/people you gave money, drugs, alcohol, shelter, food, transportation or protection to in exchange for sex male, female, or transgender? (Check all that apply)

__ Male

__ Female

__ Transgender

__ Refuse to Answer

B975. In what cities were you in when you gave someone something in exchange for sex? (Check all that apply)

__	San Diego County
__	Tijuana
__	Other city in the US
__	Other city in Mexico
__	Other
__	Refuse to Answer

If B975E is equal to 1 then skip to B976

If not, then skip to instruction before B977.

B976. Please specify the other city/country

____

If B901 is not equal to 1, then skip to B992.

B977. In the **last 6 months**, how many people did **<u>you</u>** give drugs, money, food, shelter, transportation, alcohol, or anything else in exchange for having sex with you?

__ __ __ __	People
9998	Refuse to Answer

If B977 is equal to 0 or B977 is equal to 9998, then skip to B992.

B978. In what cities were you in when you gave someone something in exchange for sex in the last 6 months? (Check all that apply)

__ San Diego County

__ Tijuana

__ Other city in the US

__ Other city in Mexico

__ Other

__ Refuse to Answer

If B978E is equal to 1 then skip to B979

If not, then skip to B980.

B979. Please specify the other city/country

____

B980. How many of those [Response to B977] people were males? __ __ __ __

9998 Refuse to Answer

If B980 is greater than B977 and B980 is not equal to 9998 then READ: "The participant's response should be less than or equal to the number of people whom you give drugs, money, food, shelter, transportation, alcohol, or anything else to in exchange for having sex with you? Please check the responses." and skip to B980.

If B980 is equal to 0 or B980 is equal to 9998, then skip to instruction before B984.

B981. How many male partners were from the US? __ __ __ __

9998 Refuse to Answer

If B981 is greater than B980 and B981 is not equal to 9998 then READ: "The participant's response should be less than or equal to the number of males whom you give drugs, money, food, shelter, transportation, alcohol, or anything else to in exchange for having sex with you. Please check the responses." and skip to B981.

If B981 is equal to B980, then skip to instruction before B984.

B982. How many male partners were from Mexico? __ __ __ __

9998 Refuse to Answer

If B981 + B982 is greater than B980 and B981 is not equal to 9998 and B982 is not equal to 9998 then READ: "The participant's response should be less than or equal to the number of males whom you give drugs, money, food, shelter, transportation, alcohol, or anything else to in exchange for having sex with you. Please check the responses." and skip to B982.

If B981 + B982 is equal to B980, then skip to instruction before B984.

B983. How many male partners were from other countries? __ __ __ __

9998 Refuse to Answer

If B981 + B982 + B983 is greater than B980 and B981 is not equal to 9998 and B982 is not equal to 9998 and B983 is not equal to 9998 then READ: "The participant's response should be less than or equal to the number of males whom you give drugs, money, food, shelter, transportation, alcohol, or anything else to in exchange for having sex with you. Please check the responses." and skip to B983.

If B980 is equal to B977, then skip to B992.

B984. How many of those [Response to B977] people were females? __ __ __ __

9998 Refuse to Answer

If B980 + B984 is greater than B977 and B980 is not equal to 9998 and B984 is not equal to 9998 then READ: "The participant's response should be less than or equal to the number of people whom you give drugs, money, food, shelter, transportation, alcohol, or anything else to in exchange for having sex with you. Please check the responses." and skip to B984.

If B984 is equal to 0 or B984 is equal to 9998, then skip to instruction before B988.

B985. How many female partners were from the US? __ __ __ __

9998 Refuse to Answer

If B985 is greater than B984 and B985 is not equal to 9998 then READ: "The participant's response should be less than or equal to the number of females whom you give drugs, money, food, shelter, transportation, alcohol, or anything else to in exchange for having sex with you. Please check the responses." and skip to B985.

If B985 is equal to B984, then skip to instruction before B988.

B986. How many female partners were from Mexico? __ __ __ __

9998 Refuse to Answer

If B985 + B986 is greater than B984 and B985 is not equal to 9998 and B986 is not equal to 9998 then READ: "The participant's response should be less than or equal to the number of females whom you give drugs, money, food, shelter, transportation, alcohol, or anything else to in exchange for having sex with you. Please check the responses." and skip to B986.

If B985 + B986 is equal to B984, then skip to instruction before B988.

B987. How many female partners were from other countries? __ __ __ __

9998 Refuse to Answer

If B985 + B986 + B987 is greater than B984 and B985 is not equal to 9998 and B986 is not equal to 9998 and B987 is not equal to 9998 then READ: "The participant's response should be less than or equal to the number of females whom you give drugs, money, food, shelter, transportation, alcohol, or anything else to in exchange for having sex with you. Please check the responses." and skip to B987.

If B980 + B984 is equal to B977, then skip to B992.

B988. How many of those [Response to B977] people were transgender?

__ __ __ __

9998 Refuse to Answer

If B980 + B984 + B988 is greater than B977 and B980 is not equal to 9998 and B984 is not equal to 9998 and B988 is not equal to 9998 then READ: "The participant's response should be less than or equal to the number of people whom you give drugs, money, food, shelter, transportation, alcohol, or anything else to in exchange for having sex with you. Please check the responses." and skip to B988.

If B988 is equal to 0 or B988 is equal to 9998, then skip to B992.

B989. How many transgender partners were from the US? __ __ __ __

9998 Refuse to Answer

If B989 is greater than B988 and B989 is not equal to 9998 then READ: "The participant's response should be less than or equal to the number of transgender whom you give drugs, money, food, shelter, transportation, alcohol, or anything else to in exchange for having sex with you. Please check the responses." and skip to B989.

If B989 is equal to B988, then skip to B992.

B990. How many transgender partners were from Mexico? __ __ __ __

9998 Refuse to Answer

If B989 + B990 is greater than B988 and B989 is not equal to 9998 and B990 is not equal to 9998 then READ: "The participant's response should be less than or equal to the number of transgender whom you give drugs, money, food, shelter, transportation, alcohol, or anything else to in exchange for having sex with you. Please check the responses." and skip to B990.

If B989 + B990 is equal to B988, then skip to B992.

B991. How many transgender partners were from other countries? __ __ __ __

9998 Refuse to Answer

If B989 + B990 + B991 is greater than B988 and B989 is not equal to 9998 and B990 is not equal to 9998 and B991 is not equal to 9998 then READ: "The participant's response should be less than or equal to the number of transgender whom you give drugs, money, food, shelter, transportation, alcohol, or anything else to in exchange for having sex with you. Please check the responses." and skip to B991.

B992. Has anyone EVER forced you to have sex with them using either physical force or emotional pressure? (Remember: By sex I mean anal, oral, or vaginal sex)

1 Yes

0 No

8 Refuse to Answer

If B992 is not equal to 1, then skip to instruction before B1001.

B993. How old were you the FIRST time someone forced you to have sex? __ __ years old

98 Refuse to Answer

If B993 is greater than A1 and B993 is not equal to 98 then READ: "Participant has responded with a length of time greater than his or her age. Please check the responses." and skip to B993.

B994. For the first time, you were forced to have sex, who forced you to have sex with them?
Interviewer: Read out the options and check all that apply (Check all that apply)

__	Family member
__	Spouse
__	Friend
__	Acquaintance
__	Stranger
__	Other Sexual Partner
__	Sex Trade Client
__	Pimp
__	Police/Authority
__	Other
__	Refuse to Answer

If B994J is not equal to 1, then skip to instruction before B996.

B995. Please, specify other

____

If B901 is not equal to 1, then skip to instruction before B1001.

B996. In the **last 6 months,** has anyone ever forced you to have sex with them using either physical force or emotional pressure? (Remember: By sex I mean anal, oral, or vaginal sex)

1	Yes
0	No
8	Refuse to Answer

If B996 is not equal to 1, then skip to instruction before B1001.

B997. In what city were you forced to have sex in the last 6 months? (Check all that apply)

__ San Diego County

__ Tijuana

__ Other city in the US

__ Other city in Mexico

__ Other

__ Refuse to Answer

If B997E is not equal to 1, then skip to B999.

B998. Please, specify other

____

B999. In the last 6 months, who forced you to have sex with them?
Interviewer: Read out the options and check all that apply (Check all that apply)

__ Family member

__ Spouse

__ Friend

__ Acquaintance

__ Stranger

__ Other Sexual Partner

__ Sex Trade Client

__ Pimp

__ Police/Authority

__ Other

__ Refuse to Answer

If B999J is not equal to 1, then skip to instruction before B1001.

B1000. Please, specify other

____

READ: INTERVIEWER READ TO THE RESPONDENT: Now I'm going to ask you about times you may have had sex with law enforcement. These may have been encounters that were voluntary or involuntary, where you were forced to have sex or you had sex in exchange for not being arrested. These questions are only about encounters that occurred in Mexico and not in the US or any other country.

B1001. Have you ever had sex with a law enforcement officer?

1	Yes
0	No
8	Refuse to Answer

If B1001 is not equal to 1 and B901 is not equal to 1, then skip to end of questionnaire.

B1002. How many times have you had sex with a law enforcement officer in **the last 6 months?**

____	# times
9998	Refuse to Answer

If B1002 is equal to 0 or B1002 is equal to 9998, then skip to end of questionnaire.

B1003. Of the [Response to B1002] times you had sex with a law enforcement officer in **the last 6 months,** how many times was this to avoid arrest or other punishment?

____	# times
9998	Refuse to Answer

If B1003 is greater than B1002 and B1003 is not equal to 9998 then READ: "The participant's response should be less than or equal to the number of times you had sex with a law enforcement officer. Please check the responses." and skip to B1003.

If B1003 is equal to 0 or B1003 is equal to 9998, then skip to end of questionnaire.

B1004. Of these [Response to B1003] number of times, how many times did this happen in the US?

____	
9998	Refuse to Answer

If B1004 is greater than B1003 and B1004 is not equal to 9998 then READ: "The participant's response should be less than or equal to the number of times you had sex with a law enforcement officer to avoid arrest or other punishment. Please check the responses." and skip to B1004.

If B1004 is equal to B1003, then skip to end of questionnaire.

B1005. Of these [Response to B1003] number of times, how many times did this happen in Mexico?

— — — —

9998 Refuse to Answer

If B1004 + B1005 is greater than B1003 and B1004 is not equal to 9998 and B1005 is not equal to 9998 then READ: "The participant's response should be less than or equal to the number of times you had sex with a law enforcement officer to avoid arrest or other punishment. Please check the responses." and skip to B1005.

READ: END OF INTERVIEW.
INTERVIEWER READ TO THE RESPONDENT: That is the end of the questionnaire. Thank you very much for your valuable time and cooperation.

DISQ1 = If(A1 is less than 18 or A1 is equal to 998 or A3 is greater than 30 or A3 is equal to 9998 or A7 is equal to 3 or A7 is equal to 8, 1, 0)

DISQ2 = If(A7 is equal to 1 and (A8 is equal to 7 or A8 is equal to 8) or A7 is equal to 2 and A12 is not equal to 1, 1, 0)

DISQ3 = If(GROUP is equal to 0, 1, 0)

DISQALL = DISQ1 + DISQ2 + DISQ3

If DISQALL is greater than 0 then skip to end of questionnaire

If not, then skip to end of questionnaire.

READ: INTERVIEWER: PARTICIPANT IS NOT ELIGIBLE! END INTERVIEW. Thank them for their time.

READ: End of survey.

Proyecto La Frontera

Entrevista de Base

Descargo de responsabilidad: Esta entrevista ha sido adaptada para su programación en QDS y no ha sido adaptada para ser administrada con papel y lápiz

IP: Steffanie Strathdee
10-18-2021

LaFrontera.Quant1.20210616 (Español)

LEA: La Frontera
Entrevista Cuantitativa (Base)
Español

VERSION = 20210616

Q1. Por favor ingresar la fecha de entrevista __ __ / __ __ / __ __ __ __ dd / mm / aaaa

If Q1 is greater than TODAY and TODAY is not equal to "Falta" then LEA: "Esa fecha ocurre en el futuro. Verificar respuesta." and siga con P Q1.

Q2. Iniciales del entrevistador. __ __ __ __

Q3. Numero de visita. (Escoja una) 1 Base/Visita 1

Q4. Ciudad de entrevista.
Entrevistador, ¡es crucial que ingrese correctamente el lugar de la entrevista!
(Escoja una)

1 San Diego

2 Tijuana

If Q4 is equal to 1 then siga con P Q5

If not, then siga con P instrucción antes de Q7.

Q5. Lugar de entrevista. (Escoja una)

1 San Diego 1

2 San Diego 2

3 San Diego 3

4 Centro de Rehabilitacion

5 Cárcel

6 Otro

If Q5 is not equal to 6, then siga con P instrucción antes de Q7.

Q6. Por favor especifica lugar de entrevista.

____

If Q4 is equal to 2 then siga con P Q7

If not, then siga con P Q9.

Q7. Lugar de entrevista. (Escoja una)

1 Tijuana 1 Zona Norte - El Mapa

2 Tijuana 2 Oficina Coahuila

3 Tijuana 3

4 Centro de Rehabilitacion

5 Cárcel

6 Otro

If Q7 is not equal to 6, then siga con P Q9.

Q8. Por favor especifica lugar de entrevista.

____

Q9. Entrevistador, ¿cómo se realizará esta entrevista? (Escoja una)

1 En persona, adentro

2 En persona, afuera

3 Por video llamada (por ejemplo: Zoom o Skype)

4 Por audio llamada (por ejemplo telefono, Zoom)

5 Otro

8 Se rehúsa a contestar

If Q9 is not equal to 5, then siga con P Q11.

Q10. Por favor especifique de qué otra manera.

— —

Q11. ID de Participante (e.g., LF001): — — — — —

ID1 = SubString(Q11, 0, 1)

ID2 = SubString(Q11, 1, 1)

ID3 = SubString(Q11, 2, 1)

ID4 = SubString(Q11, 3, 1)

ID5 = SubString(Q11, 4, 1)

IDSTUDY = Concatenated(ID1, ID2)

If IDSTUDY is not equal to LF then LEA: "Verificar ID" and siga con P Q11.

If ID3 is not equal to 0 and ID3 is not equal to 1 and ID3 is not equal to 2 and ID3 is not equal to 3 and ID3 is not equal to 4 and ID3 is not equal to 5 and ID3 is not equal to 6 and ID3 is not equal to 7 and ID3 is not equal to 8 and ID3 is not equal to 9 or ID4 is not equal to 0 and ID4 is not equal to 1 and ID4 is not equal to 2 and ID4 is not equal to 3 and ID4 is not equal to 4 and ID4 is not equal to 5 and ID4 is not equal to 6 and ID4 is not equal to 7 and ID4 is not equal to 8 and ID4 is not equal to 9 or ID5 is not equal to 0 and ID5 is not equal to 1 and ID5 is not equal to 2 and ID5 is not equal to 3 and ID5 is not equal to 4 and ID5 is not equal to 5 and ID5 is not equal to 6 and ID5 is not equal to 7 and ID5 is not equal to 8 and ID5 is not equal to 9 then LEA: "Verificar ID" and siga con P Q11.

Q12. Por favor ingresar ID de SCREENER del participante (ej. LF0001) **NOTA PARA ENTREVISTADOR: Por favor no confundir el número de screener con el ID de Participante.**

— — — — — —

SCRID1 = SubString(Q12, 0, 1)

SCRID2 = SubString(Q12, 1, 1)

SCRID3 = SubString(Q12, 2, 1)

SCRID4 = SubString(Q12, 3, 1)

SCRID5 = SubString(Q12, 4, 1)

SCRID6 = SubString(Q12, 5, 1)

IDSCREEN = Concatenated(SCRID1, SCRID2)

If IDSCREEN is not equal to LF then LEA: "Verificar ID de SCREENER" and siga con P Q12.

If SCRID3 is not equal to 0 and SCRID3 is not equal to 1 and SCRID3 is not equal to 2 and SCRID3 is not equal to 3 and SCRID3 is not equal to 4 and SCRID3 is not equal to 5 and SCRID3 is not equal to 6 and SCRID3 is not equal to 7 and SCRID3 is not equal to 8 and SCRID3 is not equal to 9 or SCRID4 is not equal to 0 and SCRID4 is not equal to 1 and SCRID4 is not equal to 2 and SCRID4 is not equal to 3 and SCRID4 is not equal to 4 and SCRID4 is not equal to 5 and SCRID4 is not equal to 6 and SCRID4 is not equal to 7 and SCRID4 is not equal to 8 and SCRID4 is not equal to 9 or SCRID5 is not equal to 0 and SCRID5 is not equal to 1 and SCRID5 is not equal to 2 and SCRID5 is not equal to 3 and SCRID5 is not equal to 4 and SCRID5 is not equal to 5 and SCRID5 is not equal to 6 and SCRID5 is not equal to 7 and SCRID5 is not equal to 8 and SCRID5 is not equal to 9 or SCRID6 is not equal to 0 and SCRID6 is not equal to 1 and SCRID6 is not equal to 2 and SCRID6 is not equal to 3 and SCRID6 is not equal to 4 and SCRID6 is not equal to 5 and SCRID6 is not equal to 6 and SCRID6 is not equal to 7 and SCRID6 is not equal to 8 and SCRID6 is not equal to 9 then LEA: "Verificar ID de SCREENER" and siga con P Q11.

Q13. ¿Qué lenguajes hablas, aun si no lo(s) hablas muy bien? **Entrevistador: Leer la lista en voz alta y escoger todas las que apliquen.** (Marque todas las que apliquen)

__ Español

__ Ingles

__ Dialecto/Lenguaje indígena

__ Otro

__ Se rehúsa a contestar

If Q13D is not equal to 1, then siga con P instrucción antes de Q15.

Q14. Por favor especifica otro lenguaje. __

If Q13B is not equal to 1, then siga con P instrucción antes de Q17.

Q15. ¿Qué tan bien **hablas** el inglés? (Escoja una)

0 Fluido/Lengua Materna
1 Muy bien
2 OK
3 No muy bien
8 Se rehúsa a contestar

Q16. ¿Qué tan bien **lees/escribes** el inglés? (Escoja una)

0 Perfectamente
1 Muy bien
2 OK
3 No muy bien
4 Para nada
8 Se rehúsa a contestar

If Q13A is not equal to 1, then siga con P instrucción antes de A1.

Q17. ¿Qué tan bien **hablas** el español? (Escoja una)

0 Fluido/Lengua Materna
1 Muy bien
2 OK
3 No muy bien
8 Se rehúsa a contestar

Q18. ¿Qué tan bien **lees/escribes** el español? (Escoja una)

0	Perfectamente
1	Muy bien
2	OK
3	No muy bien
4	Para nada
8	Se rehúsa a contestar

LEA: LEA: ANTES DE INICIAR LA ENTREVISTA, POR FAVOR LEA EL SIGUIENTE TEXTO EN VOZ ALTA AL PARTICIPANTE: Esta entrevista es parte de un estudio de investigación sobre VIH/ VHC (Hepatitis C) y sobredosis entre usuarios de drogas inyectables en San Diego y Tijuana. Le haremos preguntas que están relacionadas con la información básica acerca de usted, historia de su uso de drogas, experiencias en San Diego y Tijuana, su conducta sexual, utilización de centros de rehabilitación y otros servicios y su actitud hacia algunos de estos temas Sé que algunas preguntas que le haré pueden ser un poco incómodas para contestar, pero esta entrevista es confidencial y no vamos a compartir su información personal con ninguna otra persona fuera del estudio. Nada de lo que usted nos diga lo podrá identificar directamente. Es importante que usted sepa que su participación en esta entrevista es completamente voluntaria. No es necesario responder a una pregunta si no quiere, y usted puede dejar la entrevista en cualquier momento. ¿Tiene alguna pregunta antes de empezar?

LEA: ENTREVISTADOR LEER AL PARTICIPANTE: Primero, te voy a preguntar sobre información básica sobre ti.

A1. ¿Cuál es tu edad en años?

__ __ __	años
998	Se rehúsa a contestar

A2. ¿Cuál es su fecha de nacimiento? __ __ / __ __ / __ __ __ __

	dd / mm / aaaa
2098	Se rehúsa a contestar (Año)

If Age(A2, TODAY) is not equal to A1 and A1 is not equal to 998 and A2 is not equal to 2098 then LEA: "Por favor verificar respuesta. La edad y fecha de nacimiento que indicaste no coinciden. Por favor verificar respuesta. " and siga con P A1.

If A1 is less than 18 or A1 is equal to 998, then siga con P final de la entrevista.

A3. ¿Cuándo fue la última vez que se inyectó drogas? **Entrevistador marque HOY como 0 días**

__ __ __ __	MESES
__ __ __ __	DíAS
9998	Se rehúsa a contestar (Días)

If A3 is greater than 30 or A3 is equal to 9998, then siga con P final de la entrevista.

A4. ¿Has estado inscrito anteriormente en cualquier proyecto de investigación como El Cuete, Mujer Segura, Mujer Mas Segura?

1	Si
0	No
8	Se rehúsa a contestar

If A4 is not equal to 1, then siga con P instrucción antes de A6.

A5. Si la respuesta es sí, ¿en qué estudio participaste? (Marque todas las que apliquen)

__	El Cuete
__	Mujer Saludable Siempre
__	Proyecto EPICO
__	Otro
__	Se rehúsa a contestar

If A5D is not equal to 1, then siga con P A7.

A6. Por favor especifica en que proyecto participaste.

__ __

A7. ¿En qué país vives? (Escoja una)

1 Estados Unidos

2 México

3 Otro

8 Se rehúsa a contestar

If A7 is equal to 3 or A7 is equal to 8, then siga con P final de la entrevista.

If A7 is equal to 1 then siga con P A8

If not, then siga con P instrucción antes de A12.

A8. ¿En qué región del condado de San Diego vives? (Escoja una)

1 Ciudad de San Diego

2 Región este (East County)

3 Región norte de la costa (North County Coastal)

4 Región norte tierra adentro (North County Inland)

5 Bahía del sur (South Bay)

6 Otra región de San Diego

7 No vivo en el condado de San Diego

8 Se rehúsa a contestar

If A8 is not equal to 6, then siga con P instrucción antes de A10.

A9. Por favor especifica la región donde vives.

— —

If A8 is equal to 7 or A8 is equal to 8, then siga con P final de la entrevista.

A10. ¿En qué ciudad/ área no incorporada vives? (Escoja una)

01	Carlsbad
02	Chula Vista
03	Coronado
04	Del Mar
05	El Cajón
06	Encinitas
07	Escondido
08	Imperial Beach
09	La Mesa
10	Lemon Grove
11	National City
12	Oceanside
13	Poway
14	San Diego
15	San Marcos
16	Santee
17	Solana Beach
18	Vista
19	Otro
98	Se rehúsa a contestar

If A10 is not equal to 19, then siga con P instrucción antes de A12.

A11. Por favor especifica la otra ciudad/ área no incorporada en la que vives.

____

If A7 is equal to 2 then siga con P A12

If not, then siga con P instrucción antes de A14.

A12. ¿En qué ciudad vives? (Escoja una)

1	Tijuana
2	Mexicali
3	Ensenada
4	Rosarito
5	Otro
8	Se rehúsa a contestar

If A12 is not equal to 5, then siga con P instrucción antes de A14.

A13. Por favor especifica la otra ciudad en la que vives.

____

If A12 is not equal to 1, then siga con P final de la entrevista.

If A7 is equal to 1 then siga con P instrucción antes de A14

If not, then siga con P instrucción antes de A19.

If Q4 is equal to 1 then siga con P A14

If not, then siga con P A16.

A14. ¿Alguna vez has estado en México?

1	Si
0	No
8	Se rehúsa a contestar

If A14 is not equal to 1, then siga con P instrucción antes de A19.

A15. ¿Cuando fue la ultima vez que fue a México?

__ __ / __ __ / __ __ __ __	dd / mm / aaaa
2098	Se rehúsa a contestar (Año)

If A15 is less than A2 and A15 is not equal to 2098 then LEA: "Ha indicado que el participante visitó México antes de su fecha de nacimiento. Por favor verificar respuesta. " and siga con P A15.

A16. ¿Alguna vez has usado alguna droga en México?

1	Si
0	No
8	Se rehúsa a contestar

If A16 is not equal to 1, then siga con P instrucción antes de A19.

A17. ¿Alguna vez te has inyectado alguna droga en México?

1	Si
0	No
8	Se rehúsa a contestar

If A17 is not equal to 1, then siga con P instrucción antes de A19.

A18. ¿Cuándo fue la última vez que te inyectaste alguna droga en México?

__ __ / __ __ / __ __ __ __	dd / mm / aaaa
2098	Se rehúsa a contestar (Año)

If A18 is less than A2 and A18 is not equal to 2098 then LEA: "Has indicaado que el participante se inyecto drogas antes de su fecha de nacimiento. Por favor verificar respuesta. " and siga con P A18.

If Q4 is equal to 2 and A7 is equal to 2 then siga con P A19

If not, then siga con P instrucción antes de A24.

A19. ¿Alguna vez has estado en Estados Unidos?

1 Si

0 No

8 Se rehúsa a contestar

If A19 is not equal to 1, then siga con P instrucción antes de A24.

A20. ¿Cuando fue la ultima vez que Ud fue a los Estado Unidos?

__ __ / __ __ / __ __ __ __ dd / mm / aaaa

2098 Se rehúsa a contestar (Año)

If A20 is less than A2 and A20 is not equal to 2098 then LEA: "Ha indicado que el participante visitó EUA antes de su fecha de nacimiento. Por favor verificar respuesta. " and siga con P A20.

A21. ¿Alguna vez has usado alguna droga en Estados Unidos?

1 Si

0 No

8 Se rehúsa a contestar

If A21 is not equal to 1, then siga con P instrucción antes de A24.

A22. ¿Alguna vez te has inyectado alguna droga en Estados Unidos?

1 Si

0 No

8 Se rehúsa a contestar

If A22 is not equal to 1, then siga con P instrucción antes de A24.

A23. ¿Cuándo fue la última vez que te inyectaste alguna droga en Estados Unidos?

__ __ / __ __ / __ __ __ __ dd / mm / aaaa

2098 Se rehúsa a contestar (Año)

If A23 is less than A2 and A23 is not equal to 2098 then LEA: "Has indicaado que el participante se inyecto drogas antes de su fecha de nacimiento. Por favor verificar respuesta. " and siga con P A23.

If A7 is equal to 1 or A12 is equal to 1 and A19 is equal to 1 then siga con P A24

If not, then siga con P instrucción antes de B1.

A24.	¿Eres ciudadano/a Americano/a o residente legal de EUA?	1	Si
		0	No
		8	Se rehúsa a contestar

LCOUNTRY = If(A7 is equal to 1, the US, If(A7 is equal to 2, Mexico,))

LCITY = If(A8 is equal to 1 or A8 is equal to 2 or A8 is equal to 3 or A8 is equal to 4 or A8 is equal to 5 or A8 is equal to 6, San Diego County, If(A12 is equal to 1, Tijuana,))

DT_SD = If((A8 is equal to 1 or A8 is equal to 2 or A8 is equal to 3 or A8 is equal to 4 or A8 is equal to 5 or A8 is equal to 6) and A16 is equal to 1 and A17 is equal to 1 and A18 is not equal to 2098 and TODAY - A18 is less than 366, 1, 0)

NDT_SD = If(Q4 is equal to 1 and (A8 is equal to 1 or A8 is equal to 2 or A8 is equal to 3 or A8 is equal to 4 or A8 is equal to 5 or A8 is equal to 6) and (A14 is equal to 0 or A14 is equal to 1 and TODAY - A15 is greater than 730), 1, 0)

NDT_TJ = If(Q4 is equal to 2 and A12 is equal to 1 and (A19 is equal to 0 or A19 is equal to 1 and TODAY - A20 is greater than 730), 1, 0)

GROUP = If(DT_SD is equal to 1, 1, If(NDT_SD is equal to 1, 2, If(NDT_TJ is equal to 1, 3, 0)))

If GROUP is equal to 0, then siga con P final de la entrevista.

LEA: ENTREVISTADOR LEER AL PARTICIPANTE: Ahora te voy a preguntar sobre tu vida y de dónde vienes.

If A7 is equal to 1 then siga con P B1

If not, then siga con P instrucción antes de B3.

B1.	¿Cuál es tu estatura?	__ __	pulgadas
		98	Se rehúsa a contestar
B2.	¿Cuánto pesas?	__ __ __	libras
		998	Se rehúsa a contestar

If A7 is equal to 2 then siga con P B3

If not, then siga con P B5.

B3.	¿Cuál es tu estatura?	__ __ __	cm
		998	Se rehúsa a contestar
B4.	¿Cuánto pesas?	__ __ __	kg
		998	Se rehúsa a contestar
B5.	¿En qué país naciste? (Escoja una)	1	Estados Unidos
		2	México
		3	Otro
		7	No sé
		8	Se rehúsa a contestar

If B5 is equal to 2, then siga con P B8.

If B5 is equal to 1, then siga con P B11.

If B5 is equal to 7 or B5 is equal to 8, then siga con P B15.

B6. Por favor específica el país en el que naciste.

__ __

B7. ¿En qué estado naciste?

____

If 1 is equal to 1, then siga con P B15.

B8. ¿En qué estado naciste? **ENTREVISTADOR: Por favor selecciona el botón que corresponde a la primera letra del nombre del estado de nacimiento y luego selecciona el nombre.** (Escoja una)

1 A - M

2 N - Z

8 Se rehúsa a contestar

If B8 is not equal to 1, then siga con P instrucción antes de B10.

B9. **ENTREVISTADOR: Por favor selecciona el nombre del estado de nacimiento del participante.** (Escoja una)

01	Aguascalientes (Ags)
02	Baja California (BC)
03	Baja California Sur (BCS)
04	Chihuahua (Chih)
05	Colima (Col)
06	Campeche (Camp)
07	Coahuila (Coah)
08	Chiapas (Chis)
09	Distrito Federal (DF)
10	Durango (Dgo)
11	Guerrero (Gro)
12	Guanajuato (Gto)
13	Hidalgo (Hgo)
14	Jalisco (Jal)
15	Michoacán (Mich)
16	Morelos (Mor)
17	México (Mex or Edomex)
98	Se rehúsa a contestar

If B8 is not equal to 2, then siga con P instrucción antes de B11.

B10. **ENTREVISTADOR: Por favor selecciona el nombre del estado de nacimiento del participante.** (Escoja una)

01	Nayarit (Nay)
02	Nuevo León (NL)
03	Oaxaca (Oax)
04	Puebla (Pue)
05	Quintana Roo (Q Roo)
06	Querétaro (Qro.)
07	Sinaloa (Sin)
08	San Luis Potosí (SLP)
09	Sonora (Son)
10	Tabasco (Tab)
11	Tlaxcala (Tlax)
12	Tamaulipas (Tamps)
13	Veracruz (Ver)
14	Yucatán (Yuc)
15	Zacatecas (Zac)
98	Se rehúsa a contestar

If 1 is equal to 1, then siga con P B15.

B11. ¿En qué estado naciste? **ENTREVISTADOR: Por favor selecciona el botón que corresponde a la primera letra del nombre del estado de nacimiento y luego selecciona el nombre.** (Escoja una)

1	A - I
2	K - R
3	S - Z
8	Se rehúsa a contestar

If B11 is not equal to 1, then siga con P instrucción antes de B13.

B12. **ENTREVISTADOR: Por favor selecciona el nombre del estado de nacimiento del participante.** (Escoja una)

01	ALABAMA
02	ALASKA
03	ARIZONA
04	ARKANSAS
05	CALIFORNIA
06	COLORADO
07	CONNECTICUT
08	DELAWARE
09	FLORIDA
10	GEORGIA
11	HAWAII
12	IDAHO
13	ILLINOIS
14	INDIANA
15	IOWA
98	Se rehúsa a contestar

If B11 is not equal to 2, then siga con P instrucción antes de B14.

B13. **ENTREVISTADOR: Por favor selecciona el nombre del estado de nacimiento del participante.** (Escoja una)

01	KANSAS
02	KENTUCKY
03	LOUISIANA
04	MAINE
05	MARYLAND
06	MASSACHUSETTS
07	MICHIGAN
08	MINNESOTA
09	MISSISSIPPI
10	MISSOURI
11	MONTANA
12	NEBRASKA
13	NEVADA
14	NEW HAMPSHIRE
15	NEW JERSEY
16	NEW MEXICO
17	NEW YORK
18	NORTH CAROLINA
19	NORTH DAKOTA
20	OHIO

21	OKLAHOMA
22	OREGON
23	PENNSYLVANIA
24	RHODE ISLAND
98	Se rehúsa a contestar

If B11 is not equal to 3, then siga con P B15.

B14. **ENTREVISTADOR: Por favor selecciona el nombre del estado de nacimiento del participante.** (Escoja una)

01	SOUTH CAROLINA
02	SOUTH DAKOTA
03	TENNESSEE
04	TEXAS
05	UTAH
06	VERMONT
07	VIRGINIA
08	WASHINGTON STATE
09	Washington DC
10	WEST VIRGINIA
11	WISCONSIN
12	WYOMING
98	Se rehúsa a contestar

B15. Por favor dime en que ciudad naciste.

____

B16. ¿Qué tipo de identificación tienes? (Marque todas las que apliquen)

__ Acta de nacimiento de Estados Unidos

__ Acta de nacimiento de México

__ Pasaporte

__ Pase Sentri

__ Pase Global entry

__ Pase Ready lane

__ Visa de Turista Estados Unidos

__ Visa de Trabajo Estados Unidos

__ Credencial de elector (INE) ID Mexicana

__ Licencia de conducir Estados Unidos

__ Otro documento para cruce de frontera

__ Ninguna de las anteriores

__ Otro (especifica)

__ Se rehúsa a contestar

If B16M is equal to 1 and B16A + B16B + B16C + B16D + B16E + B16F + B16G + B16H + B16I + B16J + B16K + B16L is greater than 0 then LEA: "Solo puedes escoger "ninguno" por si solo. Verificar respuesta." and siga con P B16.

If B16L is not equal to 1, then siga con P instrucción antes de B18.

B17. Por favor, especifica el otro documento

____

If B16C is not equal to 1, then siga con P instrucción antes de B20.

B18. ¿Qué tipo de pasaportes tienes? **Entrevistador: cualquier tipo de pasaporte está bien, aun si es ilegal** (Escoja una)

1	Mexicano
2	Estados Unidos
3	Los dos
4	No tengo pasaporte
5	Otro (especifica)
8	Se rehúsa a contestar

If B18 is not equal to 5, then siga con P instrucción antes de B20.

B19. Por favor, especifica otro pasaporte

____

If B5 is not equal to 1 and (B18 is equal to 2 or B18 is equal to 3) then siga con P B20

If not, then siga con P B21.

B20. ¿Cuándo te volviste ciudadano americano? Por favor ingresar fecha de naturalización

__ __ / __ __ / __ __ __ __	dd / mm / aaaa
2098	Se rehúsa a contestar (Año)

If B20 is less than A2 and B20 is not equal to "Salto" and B20 is not equal to 2098 then LEA: "El participante ha respondido con un período de tiempo mayor que su edad. Por favor revise las respuestas." and siga con P B20.

B21. ¿Te consideras hispano/latino/mexicano?

1 Si

0 No

8 Se rehúsa a contestar

B22. ¿A qué grupo o grupos raciales consideras tu que perteneces? **Entrevistador: Lea la lista y marque todas las respuestas que apliquen**. (Marque todas las que apliquen)

__ Blanco

__ Afroamericano

__ Asiático

__ Nativo Hawaiiano u otro isleño del pacifico

__ Indígena/ Indio Americano / Nativo de Alaska

__ Raza mixta

__ Otro

__ Se rehúsa a contestar

If B22G is not equal to 1, then siga con P B24.

B23. Por favor especifica otro grupo racial:

__ __

B24. Entrevistador: Si es necesario, preguntar: ¿Cuál consideras tú que es tu género? (Escoja una)

1 Hombre

2 Mujer

3 Mujer transgenero o trasfemenina

4 Hombre transgenero o transmasculino

5 Nobinario

6 Otro

8 Se rehúsa a contestar

If B24 is not equal to 6, then siga con P B26.

B25. Por favor especifica otro género con que identificas como el tuyo.

____

B26. ¿Qué sexo esta listado en tu acta de nacimiento? (Escoja una)

1 Hombre

2 Mujer

3 Intersexual

8 Se rehúsa a contestar

B27. ¿Cuál es su estado civil? (Escoja una)

0 Soltero/ nunca se ha casado

1 Casado / unión libre

2 Divorciado

3 Separado

4 Viudo

8 Se rehúsa a contestar

B28. ¿Cuál es el nivel más alto de educación que completaste? (Escoja una)

00 Ninguno, no educación formal

01 Primaria incompleta

02 Primaria completa

03 Secundaria incompleta

04 Secundaria completa

05 Preparatoria incompleta

06 Preparatoria completa

07 Escuela Técnica

08 Universidad/Colegio

09 Otro

98 Se rehúsa a contestar

If B28 is not equal to 9, then siga con P instrucción antes de B30.

B29. Por favor especifica otro nivel de educación completado.

____

If B28 is equal to 0 or B28 is equal to 98, then siga con P instrucción antes de B31.

B30. En total, ¿Cuántos años de educación has completado?
ENTREVISTADOR: Si participante dice, No sé, preguntarle por un aproximado

__ __	años
98	Se rehúsa a contestar

If B30 is greater than A1 and B30 is not equal to 98 then LEA: "El participante contesto una cantidad mayor a su edad, verificar respuesta." and siga con P B30.

B31. En los últimos 6 meses, dígame si ha vivido o dormido en alguno de los siguientes lugares. **(Entrevistador: Lea la lista y marque todas las respuestas que apliquen.)** (Marque todas las que apliquen)

__ Casa o departamento de sus padres

__ Su casa o departamento

__ Casa de su esposo (a)/pareja sexual o departamento

__ Casa o departamento de su Familia

__ Casa o departamento de un amigo(a)

__ En un campamento migrante

__ Asilo, residencia de beneficencia, albergue

__ Albergue, residencia de bienestar

__ Lugar de trabajo

__ Cuarto de renta (en un hotel o casa de huéspedes)

__ Carro, Autobús, camión, u otro vehículo

__ Edificio abandonado

__ En un campamento/albergue para deportados

__ Institución Correccional (cárcel, Centro de detención de menores, etc.)

__ Centro de rehabilitación para el uso de drogas

__ En las calles

__ Picadero

__ Instalaciones de servicios médicos (ej., hospital, hospicio, o asilo)

__ Otro

__ Se rehúsa a contestar

If B31S is not equal to 1, then siga con P instrucción antes de B33.

B32. Por favor especifica el otro lugar donde has dormido o vivido en los últimos 6 meses.

____

If B31A + B31B + B31C + B31D + B31E + B31F + B31G + B31H + B31I + B31J + B31K + B31L + B31M + B31N + B31O + B31P + B31Q + B31R + B31S is equal to 1 or B31 is equal to 98, then siga con P B35.

B33. En los últimos 6 meses, ¿en qué lugar durmió <u>la mayor parte del tiempo</u>? (Escoja una)

01	Casa o departamento de sus padres
02	Su casa o departamento
03	Casa de su esposo (a)/pareja sexual o departamento
04	Casa o departamento de su Familia
05	Casa o departamento de un amigo(a)
06	En un campamento migrante
07	Asilo, residencia de beneficencia, albergue
08	Albergue, residencia de bienestar
09	Lugar de trabajo
10	Cuarto de renta (en un hotel o casa de huéspedes)
11	Carro, Autobús, camión, u otro vehículo
12	Edificio abandonado
13	En un campamento/albergue para deportados
14	Institución Correccional (cárcel, Centro de detención de menores, etc.)
15	Centro de rehabilitación para el uso de drogas
16	En las calles
17	Picadero
18	Instalaciones de servicios médicos (ej., hospital, hospicio, o asilo)
19	Otro
98	Se rehúsa a contestar

If B33 is equal to 1 and B31A is not equal to 1 or B33 is equal to 2 and B31B is not equal to 1 or B33 is equal to 3 and B31C is not equal to 1 or B33 is equal to 4 and B31D is not equal to 1 or B33 is equal to 5 and B31E is not equal to 1 or B33 is equal to 6 and B31F is not equal to 1 or B33 is equal to 7 and B31G is not equal to 1 or B33 is equal to 8 and B31H is not equal to 1 or B33 is equal to 9 and B31I is not equal to 1 or B33 is equal to 10 and B31J is not equal to 1 or B33 is equal to 11 and B31K is not equal to 1 or B33 is equal to 12 and B31L is not equal to 1 or B33 is equal to 13 and B31M is not equal to 1 or B33 is equal to 14 and B31N is not equal to 1 or B33 is equal to 15 and B31O is not equal to 1 or B33 is equal to 16 and B31P is not equal to 1 or B33 is equal to 17 and B31Q is not equal to 1 or B33 is equal to 18 and B31R is not equal to 1 or B33 is equal to 19 and B31S is not equal to 1 then LEA: "El participante solo puede elegir el lugar con más frecuencia de entre los lugares nombrados anteriormente." and siga con P B33.

If B33 is not equal to 19, then siga con P B35.

B34. Por favor, especifique el lugar donde dormía la mayor parte de su tiempo en los últimos 6 meses.

— —

B35. ¿Ha sido afectada su situación de vivienda por la pandemia de COVID / coronavirus que comenzó en Marzo de 2020? (Escoja una)

1 Si

2 No

3 No estoy seguro-a

8 Se rehúsa a contestar

If B35 is not equal to 1, then siga con P B37.

B36. ¿Su situación de vivienda es mejor o peor desde que comenzó COVID? (Escoja una)

1 Peor

2 Mejor

8 Se rehúsa a contestar

9 No corresponde

B37. Aproximadamente, ¿cuántas personas (incluyendo a usted) viven actualmente en su casa o, si no tiene hogar, en el lugar donde se queda la mayor parte de su tiempo? **Entrevistador: Si el participante vive solo, entre 1.**

__ __ __	Personas
998	Se rehúsa a contestar

B38. En los últimos 6 meses, en un día común, ¿cuántas horas pasa en la calle? Esto incluye buscando o vendiendo drogas, u otras formas de ganar dinero, consumiendo drogas y durmiendo en la calle.

__ __	horas al día
98	Se rehúsa a contestar

If B16A is equal to 1 or B16J is equal to 1 or B18 is equal to 2 or B18 is equal to 3 or A24 is equal to 1 then siga con P B39

If not, then siga con P instrucción antes de B41.

B39. ¿Alguna vez fuiste miembro de la militar de Estados Unidos?

1	Si
0	No
8	Se rehúsa a contestar

If B39 is not equal to 1, then siga con P instrucción antes de B41.

B40. ¿Cuándo dejaste la militar?

__ __	AñOS
__ __	MESES
98	Se rehúsa a contestar (Años)

If B40 is greater than A1 and B40 is not equal to 98 then LEA: "El participante ha respondido con un período de tiempo mayor que su edad. Por favor revise las respuestas." and siga con P B40.

B41. En los últimos 6 meses, ¿Cuáles han sido tus fuentes de ingreso? **Entrevistador: Decir recuerda que tus respuestas son confidenciales. Lee la lista y escoja todas las que apliquen.** (Marque todas las que apliquen)

__ Un empleo legal con salario

__ Un empleo informal, trabajo eventual

__ Militar

__ Encargado de un picadero

__ Vendiendo drogas

__ Prostitución/Trabajo sexual

__ Trabajo sexual (padrote)

__ Otras fuentes ilegales

__ Parientes/Amigos(as)

__ Otro

__ Ninguno

__ Se rehúsa a contestar

If B41K is equal to 1 and B41A + B41B + B41C + B41D + B41E + B41F + B41G + B41H + B41I + B41J is greater than 0 then LEA: "Solo puedes escoger la opcion "ninguno" por si sola, verificar respuesta." and siga con P B41.

If B41H is not equal to 1, then siga con P instrucción antes de B43.

B42. Por favor especifique otra fuente de ingresos ilegal:

____

If B41J is not equal to 1, then siga con P instrucción antes de B44.

B43. Por favor especifique otra fuente de ingresos:

____

If B41A + B41B + B41C + B41D + B41E + B41F + B41G + B41H + B41I + B41J + B41K is equal to 1 or B41 is equal to 98, then siga con P B47.

B44. En el ultimo año, ¿Cuál ha sido tu principal fuente de ingreso? (Escoja una)

01	Un empleo legal con salario
02	Un empleo informal, trabajo eventual
03	Militar
04	Vendiendo drogas
05	Encargado de un picadero
06	Prostitución/Trabajo sexual
07	Trabajo sexual (padrote)
08	Otras fuentes ilegales
09	Parientes/Amigos(as)
10	Otro
11	Ninguno
98	Se rehúsa a contestar

If B44 is equal to 1 and B41A is not equal to 1 or B44 is equal to 2 and B41B is not equal to 1 or B44 is equal to 3 and B41C is not equal to 1 or B44 is equal to 4 and B41E is not equal to 1 or B44 is equal to 5 and B41D is not equal to 1 or B44 is equal to 6 and B41F is not equal to 1 or B44 is equal to 7 and B41G is not equal to 1 or B44 is equal to 8 and B41H is not equal to 1 or B44 is equal to 9 and B41I is not equal to 1 or B44 is equal to 10 and B41J is not equal to 1 or B44 is equal to 11 and B41K is not equal to 1 then LEA: "El participante solo puede elegir la principal fuente de ingresos entre las fuentes mencionadas anteriormente." and siga con P B44.

If B44 is not equal to 8, then siga con P instrucción antes de B46.

B45. Por favor especifique otra fuente ilegal de ingresos:

____

If B44 is not equal to 10, then siga con P B47.

B46. Por favor especifique otra fuente de ingresos:

____

B47. Por favor, especifica el tipo de moneda en el que recibes tus ingresos. (Escoja una)

1 Pesos

2 Dólares

8 Se rehúsa a contestar

If B47 is not equal to 1, then siga con P instrucción antes de B49.

B48. ¿Cuál fue su ingreso mensual promedio durante el **año pasado**, antes de pagar impuestos, incluyendo ingresos legales e ilegales? (Escoja una)

0 Nada

1 Menos de $1000 pesos

2 $1000 - $1499 pesos

3 $1500 - $1999 pesos

4 $2000 - $2499 pesos

5 $2500 - $2999 pesos

6 $3000 - $3499 pesos

7 Más de $3500 pesos

8 Se rehúsa a contestar

If B47 is not equal to 2, then siga con P B50.

B49. ¿Cuál fue su ingreso mensual promedio durante el **año pasado**, antes de pagar impuestos, incluyendo ingresos legales e ilegales? (Escoja una)

00	Nada
01	Menos de $500 dólares
02	$500-$999 dólares
03	$1000-$1499 dólares
04	$1500-$1999 dólares
05	$2000-$2499 dólares
06	$2500-$2999 dólares
07	$3000-$3499 dólares
08	Más de $3500 dólares
98	Se rehúsa a contestar

B50. ¿Su nivel de ingresos se ha visto afectado por la pandemia de COVID / coronavirus que comenzó en Marzo de 2020? (Escoja una)

1	Si
2	No
3	No estoy seguro-a
8	Se rehúsa a contestar

If B50 is not equal to 1, then siga con P instrucción antes de B52.

B51. ¿Su nivel de ingresos es mejor o peor desde que comenzó COVID? (Escoja una)

1 Peor

2 Mejor

8 Se rehúsa a contestar

9 No corresponde

LEA: LEER: Te voy a preguntar sobre la ubicación de dónde vives, trabajas y te inyectas. Estas preguntas tienen como propósito ayudar a planificar ubicaciones de servicios de salud. Esta información no será ligada a ninguna información personal tuya, como tu nombre.
ENTREVISTADOR - TEN EL SAN DIEGO THOMAS BOOK O T MAPS PARA TIJUANA LISTOS Y DISPONIBLES PARA MOSTRAR AL PARTICIPANTE Y JUNTOS IDENTIFICAR EL BARRIO/LA COLONIA

B52. ¿Cuál es el nombre de la colonia/vecindad donde vives actualmente?

____

B53. ¿Cuál es el nombre de la colonia donde trabajas/ganas dinero **más seguido**?

____

B54. En los últimos 6 meses, ¿en qué colonia compraste u obtuviste drogas **más seguido**?

____

B55. En los últimos 6 meses, ¿en qué colonia te inyectaste drogas **más seguido**?

____

LEA: ENTREVISTADOR: Ahora te voy a preguntar sobre algunos de los lugares donde has vivido.

If A7 is equal to 2 then siga con P B56

If not, then siga con P instrucción antes de B60.

B56. ¿Has vivido en Tijuana toda tu vida?

1 Si

0 No

8 Se rehúsa a contestar

If B56 is equal to 1, then siga con P instrucción antes de B60.

B57. ¿Cuánto tiempo llevas de vivir en Tijuana ?

__ __ AñOS

__ __ MESES

__ __ SEMANAS

98 Se rehúsa a contestar (Años)

If B57 is greater than A1 and B57 is not equal to 98 then LEA: "El participante ha respondido con un período de tiempo mayor que su edad. Por favor revise las respuestas." and siga con P B57.

B58. ¿Cuál fue la razón principal por la cual te moviste a Tijuana? (Escoja una)

0 Buscando trabajo/ una vida mejor

1 Mi familia se vino a vivir aquí

2 Transferido/Reubicación de trabajo

3 Para estar cerca de un familiar

4 Fui deportado/a a Tijuana

5 Otro

8 Se rehúsa a contestar

If B58 is not equal to 5, then siga con P instrucción antes de B60.

B59. Por favor especifica la razón por la cual te moviste a Tijuana.

__ __

If A7 is equal to 1 then siga con P B60

If not, then siga con P instrucción antes de B64.

B60. ¿Has vivido en San Diego toda tu vida?

1	Si
0	No
8	Se rehúsa a contestar

If B60 is equal to 1, then siga con P instrucción antes de B64.

B61. ¿Cuánto tiempo llevas de vivir en San Diego?

__ __	AñOS
__ __	MESES
__ __	SEMANAS
98	Se rehúsa a contestar (Años)

If B61 is greater than A1 and B61 is not equal to 98 then LEA: "El participante ha respondido con un período de tiempo mayor que su edad. Por favor revise las respuestas." and siga con P B61.

B62. ¿Cuál fue la razón principal por la cual te moviste a San Diego? (Escoja una)

0	Buscando trabajo/ una vida mejor en San Diego
1	Mi familia se vino a vivir aquí
2	Transferido/Reubicación de trabajo
3	Para estar cerca de un familiar
4	Otro
8	Se rehúsa a contestar

If B62 is not equal to 4, then siga con P instrucción antes de B64.

B63. Por favor especifica la razón por la cual te moviste a San Diego.

____

USO DE DROGAS

LEA: LEER: Ahora te voy a preguntar algunas preguntas sobre tu uso de alcohol y drogas. Algunas de estas preguntas te van a pedir detalles que pueden ser difíciles de recordar. Por favor haz lo mejor que puedas para dar una repuesta aun si no es el mejor estimado. Recuerda que nosotros no compartiremos tus respuestas con la policía o con cualquier otra persona fuera de este estudio.

B64. ¿Con qué frecuencia consume alguna bebida alcohólica? (Escoja una)

0 Nunca

1 Mensualmente o menos

2 2-4 veces al mes

3 2-3 veces por semana

4 4 o más veces por semana

8 Se rehúsa a contestar

If B64 is equal to 0 or B64 is equal to 8, then siga con P B68.

B65. ¿Cuántos tragos de bebidas alcohólicas suele tomar en un día típico? (Escoja una)

1 1 to 2

2 3 to 4

3 5 to 6

4 7 to 9

5 10 o mas

8 Se rehúsa a contestar

If B26 is equal to 2 or B26 is equal to 3 then siga con P B66

If not, then siga con P instrucción antes de B67.

B66. Que tan seguido tomas 6 o mas bebidas (alcoholicas)regulares en una sola ocasion (vez)? (Escoja una)

0	Nunca
1	Menos de una vez al mes
2	Mensualmente
3	Semanalmente
4	A diario o casi a diario
8	Se rehúsa a contestar

If B26 is equal to 1 then siga con P B67

If not, then siga con P B68.

B67. Que tan seguido tomas 8 o mas bebidas (alcoholicas)regulares en una sola ocasion (vez)? (Escoja una)

0	Nunca
1	Menos de una vez al mes
2	Mensualmente
3	Semanalmente
4	A diario o casi a diario
8	Se rehúsa a contestar

B68. ¿Alguna vez fumaste cigarrillos?

1	Si
0	No
8	Se rehúsa a contestar

If B68 is not equal to 1, then siga con P B73.

B69. Actualmente ¿fumas cigarros, aunque una fumada?

1	Si
0	No
8	Se rehúsa a contestar

If B69 is equal to 0 then siga con P B70

If not, then siga con P B71.

B70. ¿Más o menos cuanto hace que fumabas cigarros? **Entrevistador: Ingresar 0 si la respuesta es Menos de un año.**

__ __ AñOS

98 Se rehúsa a contestar (Años)

If B70 is greater than A1 and B70 is not equal to 98 then LEA: "Esa respuesta excede la edad del participante. Por favor verificar respuesta." and siga con P B70.

B71. ¿Qué edad tenías cuando comenzaste a fumar cigarros? __ __

98 Se rehúsa a contestar

If B71 is greater than A1 and B71 is not equal to 98 then LEA: "Esa respuesta excede la edad del participante. Por favor verificar respuesta." and siga con P B71.

If B69 is equal to 1 then siga con P B72

If not, then siga con P B73.

B72. ¿Cuantos cigarros fuma por día? **Entrevistador: Hay 20 cigaros en un paquete.**

__ __ __ cigarros por día

998 Se rehúsa a contestar

B73. ¿Qué edad tenías la primera vez que usaste una droga ilegal?

__ __ años de edad

98 Se rehúsa a contestar

If B73 is greater than A1 then LEA: "Esa respuesta excede la edad del participante. Por favor verificar respuesta." and siga con P B73.

B74. ¿Qué droga consumiste la primera vez que usaste droga? (Marque todas las que apliquen)

__ Heroina en goma/Goma negra (la negra) sola

__ Heroína en polvo sola

__ Cocaina (en polvo) sola

__ Heroina y cocaina juntas (speedball)

__ Metanfetamina/cristal

__ Crack sola

__ China White sola

__ OxyContin, sin receta

__ Otro opioide/sustancia controlada sin receta (Vicodin, Darvon, Percocet)

__ Tranquilizantes (Rivotril, Diazepam, Valium, Ativan o Restoril)

__ Barbituricos sin receta (Amytal, Nembutal, o Seconal)

__ Fentanilo (Fentanyl)

__ Otra droga o combinación de drogas no mencionada anteriormente

__ Se rehúsa a contestar

If B74M is not equal to 1, then siga con P B76.

B75. Por favor especifica otra droga o combinación:

__ __

B76. ¿Qué edad tenías la primera vez que te inyectaste drogas? __ __ años de edad

98 Se rehúsa a contestar

If B76 is less than B73 and B73 is not equal to 98 or B76 is greater than A1 and B76 is not equal to 98 then LEA: "La respuesta es menor que la edad en que el participante usó drogas por primera vez o supera la edad del participante. Por favor revise la respuesta." and siga con P B76.

B77. ¿Qué droga o combinación de drogas te inyectaste la primera vez que te inyectaste? (Marque todas las que apliquen)

__ Heroina en goma/Goma negra (la negra) sola

__ Heroína en polvo sola

__ Cocaina (en polvo) sola

__ Heroina y cocaina juntas (speedball)

__ Metanfetamina/cristal

__ Crack sola

__ China White sola

__ OxyContin, sin receta

__ Otro opioide/sustancia controlada sin receta (Vicodin, Darvon, Percocet)

__ Tranquilizantes (Rivotril, Diazepam, Valium, Ativan o Restoril)

__ Barbituricos sin receta (Amytal, Nembutal, o Seconal)

__ Fentanilo (Fentanyl)

__ Otra droga o combinación de drogas no mencionada anteriormente

__ Se rehúsa a contestar

If B77M is not equal to 1, then siga con P B79.

B78. Por favor especifica otra droga o combinación:

__ __

B79. ¿Con quién te inyectaste la primera vez que inyectaste? (Marque todas las que apliquen)

- __ Nadie (solo)
- __ Amigo/a
- __ Esposo/a
- __ Pareja
- __ Hermano
- __ Hermana
- __ Primo/a
- __ Tía
- __ Tío
- __ Pareja sexual que no es esposo/a
- __ Conocido
- __ Desconocido
- __ Otro
- __ Se rehúsa a contestar

If B79A is equal to 1 and B79B + B79C + B79D + B79E + B79F + B79G + B79H + B79I + B79J + B79K + B79L + B79M is greater than 0 then LEA: "Solo puedes escoger la respuesta "Nadie (solo)" por si sola." and siga con P B79.

If B79M is not equal to 1, then siga con P instrucción antes de B81.

B80. Por favor especifica otra persona con la que te inyectaste la primera vez:

____

LEA: LEER: Ahora, te voy a preguntar sobre diferentes drogas que hayas usado en [LCOUNTRY]. Por favor dime si alguna vez has usado cualquiera de estas drogas en los últimos 6 meses. Si estuviste en prisión/cárcel en los últimos 6 meses por favor piensa en la ocasión cuando estuviste fuera de prisión/cárcel para responder estas preguntas.

B81. ¿Alguna vez has fumado o vapeado marihuana o hachís por si sola en el condado de [LCOUNTRY]?

1	Si
0	No
8	Se rehúsa a contestar

If B81 is not equal to 1, then siga con P B86.

B82. ¿Qué edad tenías la primera vez que fumaste o vapeaste marihuana o hachís por si sola?

__ __	años de edad
98	Se rehúsa a contestar

If B82 is less than B73 or B82 is greater than A1 and B82 is not equal to 98 then LEA: "La respuesta es menor que la edad en que el participante usó drogas por primera vez o supera la edad del participante. Por favor revise la respuesta." and siga con P B82.

B83. En los últimos 6 meses, en promedio ¿qué tan seguido fumaste marihuana o hachís por sola mientras estabas en [LCOUNTRY]? (Escoja una)

00	Nunca
01	1 vez al mes o menos
02	2-3 días al mes
03	Una vez a la semana
04	2-3 días a la semana
05	4-6 días a la semana
06	Una vez al día
07	2-3 veces al día
08	4 veces o mas por día
98	Se rehúsa a contestar

B84. ¿Alguna vez has vapeado marihuana o hachís por si sola?

1 Si

0 No

8 Se rehúsa a contestar

If B84 is not equal to 1, then siga con P B86.

B85. En los últimos 6 meses, en promedio ¿qué tan seguido vapeaste marihuana o hachís por sola mientras estabas en el condado de [LCOUNTRY]? (Escoja una)

00 Nunca

01 1 vez al mes o menos

02 2-3 días al mes

03 Una vez a la semana

04 2-3 días a la semana

05 4-6 días a la semana

06 Una vez al día

07 2-3 veces al día

08 4 veces o mas por día

98 Se rehúsa a contestar

B86. ¿Alguna vez has usado heroína?

1 Si

0 No

8 Se rehúsa a contestar

If B86 is not equal to 1, then siga con P B100.

B87. ¿Alguna vez has fumado, aspirado o inhalado heroína?

1 Si

0 No

8 Se rehúsa a contestar

If B87 is not equal to 1, then siga con P B90.

B88. ¿Qué edad tenías la primera vez que fumaste, aspiraste o inhalaste heroína?

__ __	años de edad
98	Se rehúsa a contestar

If B88 is less than B73 or B88 is greater than A1 and B88 is not equal to 98 then LEA: "La respuesta es menor que la edad en que el participante usó drogas por primera vez o supera la edad del participante. Por favor revise la respuesta." and siga con P B88.

B89. En los últimos 6 meses, en promedio ¿qué tan seguido fumaste, aspiraste o inhalaste heroína mientras estabas en [LCOUNTRY]? (Escoja una)

00	Nunca
01	1 vez al mes o menos
02	2-3 días al mes
03	Una vez a la semana
04	2-3 días a la semana
05	4-6 días a la semana
06	Una vez al día
07	2-3 veces al día
08	4 veces o mas por día
98	Se rehúsa a contestar

B90. ¿Alguna vez te has inyectado heroína sola?

1	Si
0	No
8	Se rehúsa a contestar

If B90 is not equal to 1, then siga con P instrucción antes de B93.

B91. ¿Qué edad tenías la primera vez que te inyectaste heroína sola?

__ __	años de edad
98	Se rehúsa a contestar

If B91 is less than B76 or B91 is greater than A1 and B91 is not equal to 98 then LEA: "La respuesta es menor que la edad en que el participante se inyecta drogas por primera vez o supera la edad del participante. Por favor revise la respuesta." and siga con P B91.

B92. En los últimos 6 meses, en promedio ¿Qué tan seguido te inyectaste heroína sola en [LCOUNTRY]? (Escoja una)

00	Nunca
01	1 vez al mes o menos
02	2-3 días al mes
03	Una vez a la semana
04	2-3 días a la semana
05	4-6 días a la semana
06	Una vez al día
07	2-3 veces al día
08	4 veces o mas por día
98	Se rehúsa a contestar

If (B89 is equal to 0 or B89 is equal to 98 or B89 is equal to "Salto") and (B92 is equal to 0 or B92 is equal to 98 or B92 is equal to "Salto"), then siga con P B100.

B93. En los últimos 6 meses, cuando usaste heroína en [LCOUNTRY], ¿de qué color era?
Entrevistador: Leer en voz alta todas las opciones y escoja todas las que apliquen
(Marque todas las que apliquen)

__	Goma negra
__	Polvo café
__	Polvo blanco
__	Polvo amarillo
__	Polvo naranja
__	Otro
__	Se rehúsa a contestar

If B93F is not equal to 1, then siga con P instrucción antes de B95.

B94. Por favor especifica el color de heroína que usaste.

____

If B92 is equal to 0 or B92 is equal to 98 or B92 is equal to "Salto", then siga con P B100.

B95. En los últimos 6 meses, cuando te inyectaste heroína en [LCOUNTRY], de qué color fue más seguido? (Escoja una)

1	Goma negra
2	Polvo café
3	Polvo blanco
4	Polvo amarillo
5	Polvo naranja
6	Otro
8	Se rehúsa a contestar

If B95 is not equal to 6, then siga con P instrucción antes de B97.

B96. Por favor especifica el color de heroína que usaste.

____

If B95 is not equal to 4, then siga con P instrucción antes de B98.

B97. En los últimos 6 meses, cuando te inyectaste heroína en [LCOUNTRY], ¿Qué tan seguido fue color ***amarillo***? (Escoja una)

1 Nunca
2 Menos de la mitad del tiempo
3 Casi la mitad del tiempo
4 Más de la mitad del tiempo
5 Siempre
8 Se rehúsa a contestar

If B95 is not equal to 5, then siga con P instrucción antes de B99.

B98. En los últimos 6 meses, cuando te inyectaste heroína en [LCOUNTRY], ¿Qué tan seguido fue color ***naranja***? (Escoja una)

1 Nunca
2 Menos de la mitad del tiempo
3 Casi la mitad del tiempo
4 Más de la mitad del tiempo
5 Siempre
8 Se rehúsa a contestar

If B95 is not equal to 6, then siga con P B100.

B99. En los últimos 6 meses, cuando te inyectaste heroína en [LCOUNTRY], ¿Qué tan seguido fue [Respuesta a P B96]? (Escoja una)

1 Nunca

2 Menos de la mitad del tiempo

3 Casi la mitad del tiempo

4 Más de la mitad del tiempo

5 Siempre

8 Se rehúsa a contestar

B100. ¿Alguna vez usaste cocaína o crack?

1 Si

0 No

8 Se rehúsa a contestar

If B100 is not equal to 1, then siga con P B111.

B101. ¿Alguna vez as fumado, aspirado o inhalado crack o cocaína en polvo?

1 Si

0 No

8 Se rehúsa a contestar

If B101 is not equal to 1, then siga con P B104.

B102. ¿Qué edad tenías la primera vez que fumaste, aspiraste o inhalaste crack o cocaína en polvo?

__ __ años de edad

98 Se rehúsa a contestar

If B102 is less than B73 or B102 is greater than A1 and B102 is not equal to 98 then LEA: "La respuesta es menor que la edad en que el participante usó drogas por primera vez o supera la edad del participante. Por favor revise la respuesta." and siga con P B102.

B103. En los últimos 6 meses, en promedio ¿Qué tan seguido fumaste, aspiraste o inhalaste crack o cocaína en polvo cuando estabas en [LCOUNTRY]? (Escoja una)

00	Nunca
01	1 vez al mes o menos
02	2-3 días al mes
03	Una vez a la semana
04	2-3 días a la semana
05	4-6 días a la semana
06	Una vez al día
07	2-3 veces al día
08	4 veces o mas por día
98	Se rehúsa a contestar

B104. ¿Alguna vez te has inyectado cocaína sola?

1	Si
0	No
8	Se rehúsa a contestar

If B104 is not equal to 1, then siga con P instrucción antes de B107.

B105. ¿Qué edad tenías la primera vez que te inyectaste cocaína sola?

__ __	años de edad
98	Se rehúsa a contestar

If B105 is less than B76 or B105 is greater than A1 and B105 is not equal to 98 then LEA: "La respuesta es menor que la edad en que el participante se inyecta drogas por primera vez o supera la edad del participante. Por favor revise la respuesta." and siga con P B105.

B106. En los últimos 6 meses, en promedio ¿Qué tan seguido te inyectaste cocaína sola cuando estuviste en [LCOUNTRY]? (Escoja una)

00	Nunca
01	1 vez al mes o menos
02	2-3 días al mes
03	Una vez a la semana
04	2-3 días a la semana
05	4-6 días a la semana
06	Una vez al día
07	2-3 veces al día
08	4 veces o mas por día
98	Se rehúsa a contestar

If B86 is not equal to 1, then siga con P B111.

B107. ¿Alguna vez te has inyectado heroína/heroína en goma y cocaína juntas (speedball)?

1	Si
0	No
8	Se rehúsa a contestar

If B107 is not equal to 1, then siga con P B111.

B108. ¿Qué edad tenías la primera vez que te inyectaste heroína y cocaína juntas (speedball)?

__ __	años de edad
98	Se rehúsa a contestar

If B108 is less than B76 or B108 is greater than A1 and B108 is not equal to 98 then LEA: "La respuesta es menor que la edad en que el participante se inyecta drogas por primera vez o supera la edad del participante. Por favor revise la respuesta." and siga con P B108.

B109. En los últimos 6 meses, en promedio ¿Qué tan seguido te inyectaste heroína y cocaína juntas (speedball) cuando estuviste en [LCOUNTRY]? (Escoja una)

00	Nunca
01	1 vez al mes o menos
02	2-3 días al mes
03	Una vez a la semana
04	2-3 días a la semana
05	4-6 días a la semana
06	Una vez al día
07	2-3 veces al día
08	4 veces o mas por día
98	Se rehúsa a contestar

If B109 is equal to 0 or B109 is equal to 98, then siga con P B111.

B110. En los últimos 6 meses, en promedio cuantas veces al dia ¿Te inyectaste heroína y cocaína juntas (speedball)? **ENTREVISTADOR: si el participante dice No se preguntarle por un estimado.**

__ __	veces al día
97	No sé
98	Se rehúsa a contestar

B111. ¿Alguna vez has usado metanfetamina (cristal)?

1	Si
0	No
8	Se rehúsa a contestar

If B111 is not equal to 1, then siga con P B128.

B112. ¿Alguna vez has fumado, aspirado, inhalado o vapeado metanfetamina (cristal) sola?

1	Si
0	No
8	Se rehúsa a contestar

If B112 is not equal to 1, then siga con P B115.

B113. ¿Qué edad tenías la primera vez que fumaste, aspiraste, inhalaste o vapeaste metanfetamina /cristal?

__ __	años de edad
98	Se rehúsa a contestar

If B113 is less than B73 or B113 is greater than A1 and B113 is not equal to 98 then LEA: "La respuesta es menor que la edad en que el participante usó drogas por primera vez o supera la edad del participante. Por favor revise la respuesta." and siga con P B113.

B114. En los últimos 6 meses, en promedio ¿Qué tan seguido fumaste, aspiraste, inhalaste o vapeaste metanfetamina (cristal) en [LCOUNTRY]? (Escoja una)

00	Nunca
01	1 vez al mes o menos
02	2-3 días al mes
03	Una vez a la semana
04	2-3 días a la semana
05	4-6 días a la semana
06	Una vez al día
07	2-3 veces al día
08	4 veces o mas por día
98	Se rehúsa a contestar

B115. ¿Alguna vez te has inyectado metanfetamina/cristal solo?

1	Si
0	No
8	Se rehúsa a contestar

If B115 is not equal to 1, then siga con P instrucción antes de B122.

B116. ¿Qué edad tenías la primera vez que te inyectaste cristal solo?

__ __	años de edad
98	Se rehúsa a contestar

If B116 is less than B76 or B116 is greater than A1 and B116 is not equal to 98 then LEA: "La respuesta es menor que la edad en que el participante se inyecta drogas por primera vez o supera la edad del participante. Por favor revise la respuesta." and siga con P B116.

B117. En los últimos 6 meses, en promedio ¿Qué tan seguido te inyectaste metanfetaminas/cristal solo cuando estuviste en el condado de [LCOUNTRY]? (Escoja una)

00	Nunca
01	1 vez al mes o menos
02	2-3 días al mes
03	Una vez a la semana
04	2-3 días a la semana
05	4-6 días a la semana
06	Una vez al día
07	2-3 veces al día
08	4 veces o mas por día
98	Se rehúsa a contestar

If B117 is equal to 0 or B117 is equal to 98, then siga con P instrucción antes de B122.

B118. En los últimos 6 meses cuando te inyectaste metanfetaminas (sola o en combinación con otras drogas) en [LCOUNTRY], de qué color era? (Marque todas las que apliquen)

__	Blanco
__	Rosa
__	Verde
__	Negro
__	Amarillo
__	Hielo (transparente)
__	Otro
__	Se rehúsa a contestar

If B118G is not equal to 1, then siga con P instrucción antes de B120.

B119. Por favor especifica el color de metanfetaminas

____

If B118A + B118B + B118C + B118D + B118E + B118F + B118G is equal to 1 or B118 is equal to 8, then siga con P instrucción antes de B122.

B120. En los últimos 6 meses, cuando te inyectaste metanfetaminas en [LCOUNTRY], de qué color fue más seguido? (Escoja una)

1	Blanco
2	Rosa
3	Verde
4	Negro
5	Amarillo
6	Hielo (transparente)
7	Otro
8	Se rehúsa a contestar

If B120 is equal to 1 and B118A is not equal to 1 or B120 is equal to 2 and B118B is not equal to 1 or B120 is equal to 3 and B118C is not equal to 1 or B120 is equal to 4 and B118D is not equal to 1 or B120 is equal to 5 and B118E is not equal to 1 or B120 is equal to 6 and B118F is not equal to 1 or B120 is equal to 7 and B118G is not equal to 1 then LEA: "El participante solo puede elegir el color más frecuente de los colores mencionados anteriormente." and siga con P B120.

If B120 is not equal to 7, then siga con P instrucción antes de B122.

B121. Por favor especifica el color de metanfetaminas

__ __

If B100 is not equal to 1, then siga con P instrucción antes de B125.

B122. ¿Alguna vez te inyectaste metanfetaminas/cristal y cocaína juntas?

1 Si

0 No

8 Se rehúsa a contestar

If B122 is not equal to 1, then siga con P instrucción antes de B125.

B123. ¿Qué edad tenías la primera vez que te inyectaste metanfetaminas/cristal y cocaína juntas?

__ __ años de edad

98 Se rehúsa a contestar

If B123 is less than B76 or B123 is greater than A1 and B123 is not equal to 98 then LEA: "La respuesta es menor que la edad en que el participante se inyecta drogas por primera vez o supera la edad del participante. Por favor revise la respuesta." and siga con P B123.

B124. En los últimos 6 meses, en promedio, ¿qué tan seguido te inyectaste metanfetaminas/cristal y cocaína juntas cuando estuviste en [LCOUNTRY]? (Escoja una)

00	Nunca
01	1 vez al mes o menos
02	2-3 días al mes
03	Una vez a la semana
04	2-3 días a la semana
05	4-6 días a la semana
06	Una vez al día
07	2-3 veces al día
08	4 veces o mas por día
98	Se rehúsa a contestar

If B86 is not equal to 1, then siga con P B128.

B125. ¿Alguna vez te has inyectado metanfetaminas/cristal y heroína juntas?

1	Si
0	No
8	Se rehúsa a contestar

If B125 is not equal to 1, then siga con P B128.

B126. ¿Qué edad tenías la primera vez que te inyectaste metanfetaminas/cristal y heroína juntas?

__ __	años de edad
98	Se rehúsa a contestar

If B126 is less than B76 or B126 is greater than A1 and B126 is not equal to 98 then LEA: "La respuesta es menor que la edad en que el participante se inyecta drogas por primera vez o supera la edad del participante. Por favor revise la respuesta." and siga con P B126.

B127. En los últimos 6 meses, en promedio, ¿qué tan seguido te inyectaste metanfetaminas/cristal y heroína juntas en [LCOUNTRY]? (Escoja una)

00	Nunca
01	1 vez al mes o menos
02	2-3 días al mes
03	Una vez a la semana
04	2-3 días a la semana
05	4-6 días a la semana
06	Una vez al día
07	2-3 veces al día
08	4 veces o mas por día
98	Se rehúsa a contestar

B128. ¿Qué tipo/s de drogas crees que están presentes en China White? (Marque todas las que apliquen)

__ Heroína

__ Fentanilo

__ Oxycontin

__ Opioides recetados (Vicodin, Darvon, Percocet)

__ Metanfetamina

__ Cocaína

__ Crocodilo

__ Barbitúricos

__ Agente de corte (ej. Talco de bebe, leche en polvo, cafeína, bicarbonato de sodio, etc)

__ Veneno

__ Otro (epecifica)

__ No sé

__ Se rehúsa a contestar

If B128K is not equal to 1, then siga con P instrucción antes de B130.

B129. Especifica qué tipo de droga o combinación de drogas sea China White.

__ __

If B128B is equal to 1, then siga con P B140.

B130. ¿Alguna vez has usado China White?

1 Si

0 No

8 Se rehúsa a contestar

If B130 is not equal to 1, then siga con P B140.

B131. ¿Alguna vez has fumado, aspirado, inhalado o vapeado China White?

1	Si
0	No
8	Se rehúsa a contestar

If B131 is not equal to 1, then siga con P B134.

B132. ¿Qué edad tenías la primera vez que fumaste, aspiraste, inhalaste o vapeaste China White?

__ __	años de edad
98	Se rehúsa a contestar

If B132 is less than B73 or B132 is greater than A1 and B132 is not equal to 98 then LEA: "La respuesta es menor que la edad en que el participante usó drogas por primera vez o supera la edad del participante. Por favor revise la respuesta." and siga con P B132.

B133. En los últimos 6 meses, ¿qué tan seguido fumaste, aspiraste, o inhalaste China White en [LCOUNTRY]? (Escoja una)

00	Nunca
01	1 vez al mes o menos
02	2-3 días al mes
03	Una vez a la semana
04	2-3 días a la semana
05	4-6 días a la semana
06	Una vez al día
07	2-3 veces al día
08	4 veces o mas por día
98	Se rehúsa a contestar

B134. ¿Alguna vez te inyectaste China White sola?

1	Si
0	No
8	Se rehúsa a contestar

If B134 is not equal to 1, then siga con P instrucción antes de B137.

B135. ¿Qué edad tenías la primera vez que te inyectaste China White sola?

__ __	años de edad
98	Se rehúsa a contestar

If B135 is less than B76 or B135 is greater than A1 and B135 is not equal to 98 then LEA: "La respuesta es menor que la edad en que el participante se inyecta drogas por primera vez o supera la edad del participante. Por favor revise la respuesta." and siga con P B135.

B136. En los últimos 6 meses, cuando usaste China White en [LCOUNTRY], en promedio, ¿qué tan seguido te la inyectaste sola? (Escoja una)

00	Nunca
01	1 vez al mes o menos
02	2-3 días al mes
03	Una vez a la semana
04	2-3 días a la semana
05	4-6 días a la semana
06	Una vez al día
07	2-3 veces al día
08	4 veces o mas por día
98	Se rehúsa a contestar

If B111 is not equal to 1, then siga con P B140.

B137. ¿Alguna vez te has inyectado China White y metanfetaminas/cristal juntas?

1	Si
0	No
8	Se rehúsa a contestar

If B137 is not equal to 1, then siga con P B140.

B138. ¿Qué edad tenías la primera vez que te inyectaste China White y metanfetaminas/cristal juntas?

__ __	años de edad
98	Se rehúsa a contestar

If B138 is less than B76 or B138 is greater than A1 and B138 is not equal to 98 then LEA: "La respuesta es menor que la edad en que el participante se inyecta drogas por primera vez o supera la edad del participante. Por favor revise la respuesta." and siga con P B138.

B139. En los últimos 6 meses, ¿cuándo usaste China White y metanfetaminas/cristal juntas en [LCOUNTRY], en promedio que tan seguido te inyectaste las dos juntas? (Escoja una)

00	Nunca
01	1 vez al mes o menos
02	2-3 días al mes
03	Una vez a la semana
04	2-3 días a la semana
05	4-6 días a la semana
06	Una vez al día
07	2-3 veces al día
08	4 veces o mas por día
98	Se rehúsa a contestar

B140. ¿Alguna vez has usado fentanilo (fentanyl)?

1 Si

0 No

7 No sé

8 Se rehúsa a contestar

If B140 is not equal to 1, then siga con P B154.

B141. ¿Alguna vez fumaste, inhalaste, aspiraste o vapeaste fentanilo (fentanyl)?

1 Si

0 No

8 Se rehúsa a contestar

If B141 is not equal to 1, then siga con P B144.

B142. ¿Qué edad tenías la primera vez que fumaste, inhalaste, aspiraste o vapeaste fentanilo (fentanyl)?

__ __ años de edad

98 Se rehúsa a contestar

If B142 is less than B73 or B142 is greater than A1 and B142 is not equal to 98 then LEA: "La respuesta es menor que la edad en que el participante usó drogas por primera vez o supera la edad del participante. Por favor revise la respuesta." and siga con P B142.

B143. Durante los últimos 6 meses, en promedio ¿qué tan seguido fumaste, inhalaste, aspiraste o vapeaste fentanilo (fentanyl) cuando estuviste en [LCOUNTRY]? (Escoja una)

00	Nunca
01	1 vez al mes o menos
02	2-3 días al mes
03	Una vez a la semana
04	2-3 días a la semana
05	4-6 días a la semana
06	Una vez al día
07	2-3 veces al día
08	4 veces o mas por día
98	Se rehúsa a contestar

B144. ¿Alguna vez te has inyectado fentanilo (fentanyl) solo?

1	Si
0	No
8	Se rehúsa a contestar

If B144 is not equal to 1, then siga con P instrucción antes de B151.

B145. ¿Qué edad tenías la <u>primera</u> vez que inyectaste fentanilo (fentanyl) solo?

__ __	años de edad
98	Se rehúsa a contestar

If B145 is less than B76 or B145 is greater than A1 and B145 is not equal to 98 then LEA: "La respuesta es menor que la edad en que el participante se inyecta drogas por primera vez o supera la edad del participante. Por favor revise la respuesta." and siga con P B145.

B146. Durante los últimos 6 meses cuando estuviste en [LCOUNTRY], en promedio ¿Qué tan seguido te inyectaste fentanilo (fentanyl) solo? (Escoja una)

00	Nunca
01	1 vez al mes o menos
02	2-3 días al mes
03	Una vez a la semana
04	2-3 días a la semana
05	4-6 días a la semana
06	Una vez al día
07	2-3 veces al día
08	4 veces o mas por día
98	Se rehúsa a contestar

If B146 is equal to 0 or B146 is equal to 98 or B146 is equal to "Salto", then siga con P instrucción antes de B151.

B147. En los últimos 6 meses, cuando estuviste en [LCOUNTRY], de que colores era el Fentanilo (Fentanyl) que te inyectaste (sea por si solo o en combinación con otra droga)? (Marque todas las que apliquen)

__	Blanco
__	Amarillo
__	Naranja
__	Café
__	Azul
__	Otro
__	Se rehúsa a contestar

If B147F is not equal to 1, then siga con P instrucción antes de B149.

B148. Especifica otro color de fentanilo (Fentanyl)

____

If B147A + B147B + B147C + B147D + B147E + B147F is equal to 1 or B147 is equal to 8, then siga con P instrucción antes de B151.

B149. En los últimos 6 meses, cuando te inyectaste Fentanilo (Fentanyl) en [LCOUNTRY] de que color era mas seguido? (Escoja una)

1 Blanco

2 Amarillo

3 Naranja

4 Café

5 Azul

6 Otro

8 Se rehúsa a contestar

If B149 is equal to 1 and B147A is not equal to 1 or B149 is equal to 2 and B147B is not equal to 1 or B149 is equal to 3 and B147C is not equal to 1 or B149 is equal to 4 and B147D is not equal to 1 or B149 is equal to 5 and B147E is not equal to 1 or B149 is equal to 6 and B147F is not equal to 1 then LEA: "El participante solo puede elegir el color más frecuente de los colores mencionados anteriormente." and siga con P B149.

If B149 is not equal to 6, then siga con P instrucción antes de B151.

B150. Especifica el color de fentanilo (Fentanyl)

____

If B111 is not equal to 1, then siga con P B154.

B151. ¿Alguna vez te has inyectado fentanilo (fentanyl) y metanfetaminas/cristal juntos?

1 Si

0 No

8 Se rehúsa a contestar

If B151 is not equal to 1, then siga con P B154.

B152. ¿Qué edad tenías la primera vez que inyectaste fentanilo (fentanyl) y metanfetaminas/cristal juntos?

__ __	años de edad
98	Se rehúsa a contestar

If B152 is less than B76 or B152 is greater than A1 and B152 is not equal to 98 then LEA: "La respuesta es menor que la edad en que el participante se inyecta drogas por primera vez o supera la edad del participante. Por favor revise la respuesta." and siga con P B152.

B153. En los últimos 6 meses, cuando estuviste en [LCOUNTRY], en promedio, ¿Qué tan seguido te inyectaste fentanilo (fentanyl) y metanfetaminas/cristal juntos? (Escoja una)

00	Nunca
01	1 vez al mes o menos
02	2-3 días al mes
03	Una vez a la semana
04	2-3 días a la semana
05	4-6 días a la semana
06	Una vez al día
07	2-3 veces al día
08	4 veces o mas por día
98	Se rehúsa a contestar

B154. ¿Alguna vez has usado PCP/Polvo de Angel?

1	Si
0	No
8	Se rehúsa a contestar

If B154 is not equal to 1, then siga con P B158.

B155. ¿Alguna vez has fumado/inhalado PCP/Polvo de Ángel?

1 Si

0 No

8 Se rehúsa a contestar

If B155 is not equal to 1, then siga con P B158.

B156. ¿Cuantos años tenías cuando fumaste o inhalaste PCP/Polvo de Ángel por la primera vez?

__ __ años de edad

98 Se rehúsa a contestar

If B156 is less than B73 or B156 is greater than A1 and B156 is not equal to 98 then LEA: "La respuesta es menor que la edad en que el participante usó drogas por primera vez o supera la edad del participante. Por favor revise la respuesta." and siga con P B156.

B157. En los últimos 6 meses, cuando usaste PCP/Polvo de Ángel en [LCOUNTRY], en promedio, ¿qué tan seguido lo fumaste o inhalaste? (Escoja una)

00 Nunca

01 1 vez al mes o menos

02 2-3 días al mes

03 Una vez a la semana

04 2-3 días a la semana

05 4-6 días a la semana

06 Una vez al día

07 2-3 veces al día

08 4 veces o mas por día

98 Se rehúsa a contestar

B158. ¿Alguna vez has usado éxtasis?

1 Si

0 No

8 Se rehúsa a contestar

If B158 is not equal to 1, then siga con P B166.

B159. ¿Qué edad tenías la primera vez que usaste éxtasis?

__ __	años de edad
98	Se rehúsa a contestar

If B159 is less than B73 or B159 is greater than A1 and B159 is not equal to 98 then LEA: "La respuesta es menor que la edad en que el participante usó drogas por primera vez o supera la edad del participante. Por favor revise la respuesta." and siga con P B159.

B160. ¿Alguna vez te has inyectado éxtasis?

1	Si
0	No
8	Se rehúsa a contestar

If B160 is not equal to 1, then siga con P B162.

B161. ¿Qué edad tenías la primera vez que te inyectaste éxtasis?

__ __	años de edad
98	Se rehúsa a contestar

If B161 is less than B76 or B161 is greater than A1 and B161 is not equal to 98 then LEA: "La respuesta es menor que la edad en que el participante se inyecta drogas por primera vez o supera la edad del participante. Por favor revise la respuesta." and siga con P B161.

B162. En los últimos 6 meses si usaste éxtasis en [LCOUNTRY], ¿Cómo los usaste?
Entrevistador: Si el participante indica que no usó éxtasis **en** [LCOUNTRY] **en los últimos 6 meses, marque No corresponde.**
(Marque todas las que apliquen)

__ Inyectado

__ Fumado (chasing)

__ Aspirado

__ Inhalado

__ Tomado

__ Rectal (bumping)

__ Otro

__ No corresponde

__ Se rehúsa a contestar

If B162 is greater than 1 and B162H is equal to 1 then LEA: "Solo puedes escoger la opcion "No corresponde" por si sola, verificar respuesta." and siga con P B162.

If B162G is not equal to 1, then siga con P instrucción antes de B164.

B163. Especifica de que otra manera has usado éxtasis en los últimos 6 meses en Estados Unidos.

____

If B162A is not equal to 1, then siga con P instrucción antes de B165.

B164. En los últimos 6 meses, cuando usaste éxtasis en [LCOUNTRY], en promedio, ¿qué tan seguido te lo inyectaste? (Escoja una)

00	Nunca
01	1 vez al mes o menos
02	2-3 días al mes
03	Una vez a la semana
04	2-3 días a la semana
05	4-6 días a la semana
06	Una vez al día
07	2-3 veces al día
08	4 veces o mas por día
98	Se rehúsa a contestar

If B162H is equal to 1 or B162 is equal to 9, then siga con P B166.

B165. En los últimos 6 meses, cuando usaste éxtasis en [LCOUNTRY], en promedio, ¿qué tan seguido lo usaste de otra manera (no inyectada)? (Escoja una)

00	Nunca
01	1 vez al mes o menos
02	2-3 días al mes
03	Una vez a la semana
04	2-3 días a la semana
05	4-6 días a la semana
06	Una vez al día
07	2-3 veces al día
08	4 veces o mas por día
98	Se rehúsa a contestar

B166. ¿Alguna vez ha usado Ketamina (Special K, K)?	1	Si
	0	No
	8	Se rehúsa a contestar

If B166 is not equal to 1, then siga con P B174.

B167. ¿Qué edad tenías la primera vez que usaste Ketamina?	__ __	años de edad
	98	Se rehúsa a contestar

If B167 is less than B73 or B167 is greater than A1 and B167 is not equal to 98 then LEA: "La respuesta es menor que la edad en que el participante usó drogas por primera vez o supera la edad del participante. Por favor revise la respuesta." and siga con P B167.

B168. ¿Alguna vez te has inyectado Ketamina?	1	Si
	0	No
	8	Se rehúsa a contestar

If B168 is not equal to 1, then siga con P B170.

B169. ¿Qué edad tenías la primera vez que te inyectaste Ketamina?

	__ __	años de edad
	98	Se rehúsa a contestar

If B169 is less than B76 or B169 is greater than A1 and B169 is not equal to 98 then LEA: "La respuesta es menor que la edad en que el participante se inyecta drogas por primera vez o supera la edad del participante. Por favor revise la respuesta." and siga con P B169.

B170. Si usaste ketamina en los últimos 6 meses en [LCOUNTRY], ¿Cómo los usaste?
Entrevistador: Si el participante indica que no usó ketamina **en** [LCOUNTRY] **en los últimos 6 meses, marque No corresponde.**
(Marque todas las que apliquen)

__ Inyectado

__ Fumado (chasing)

__ Aspirado

__ Inhalado

__ Tomado

__ Rectal (bumping)

__ Otro

__ No corresponde

__ Se rehúsa a contestar

If B170 is greater than 1 and B170H is equal to 1 then LEA: "Solo puedes escoger la opcion "No corresponde" por si sola, verificar respuesta." and siga con P B170.

If B170G is not equal to 1, then siga con P instrucción antes de B172.

B171. Especifica de que otra manera usaste Ketamina en los últimos 6 meses.

____

If B170A is not equal to 1, then siga con P instrucción antes de B173.

B172. Durante los últimos 6 meses cuando usaste ketamina en [LCOUNTRY], en promedio, ¿Qué tan seguido te la inyectaste? (Escoja una)

00	Nunca
01	1 vez al mes o menos
02	2-3 días al mes
03	Una vez a la semana
04	2-3 días a la semana
05	4-6 días a la semana
06	Una vez al día
07	2-3 veces al día
08	4 veces o mas por día
98	Se rehúsa a contestar
09	No corresponde

If B170H is equal to 1 or B170 is equal to 98, then siga con P B174.

B173. Durante los últimos 6 meses cuando usaste ketamina en [LCOUNTRY], en promedio, ¿Qué tan seguido la usaste de otras maneras (no inyectadas)? (Escoja una)

00	Nunca
01	1 vez al mes o menos
02	2-3 días al mes
03	Una vez a la semana
04	2-3 días a la semana
05	4-6 días a la semana
06	Una vez al día
07	2-3 veces al día
08	4 veces o mas por día
98	Se rehúsa a contestar
09	No corresponde

B174. ¿Alguna vez has usado OxyContin?

1	Si
0	No
8	Se rehúsa a contestar

If B174 is not equal to 1, then siga con P B182.

B175. ¿Qué edad tenías la primera vez que usaste Oxycontin? __ __

__ __	años de edad
98	Se rehúsa a contestar

If B175 is less than B73 or B175 is greater than A1 and B175 is not equal to 98 then LEA: "La respuesta es menor que la edad en que el participante usó drogas por primera vez o supera la edad del participante. Por favor revise la respuesta." and siga con P B175.

B176. ¿Alguna vez te has inyectado oxycontin?

1	Si
0	No
8	Se rehúsa a contestar

If B176 is not equal to 1, then siga con P B178.

B177. ¿Qué edad tenías la primera vez que te inyectaste oxycontin?

__ __ años de edad

98 Se rehúsa a contestar

If B177 is less than B76 or B177 is greater than A1 and B177 is not equal to 98 then LEA: "La respuesta es menor que la edad en que el participante se inyecta drogas por primera vez o supera la edad del participante. Por favor revise la respuesta." and siga con P B177.

B178. Si usaste oxycontin en los últimos 6 meses en [LCOUNTRY], ¿Cómo los usaste?
Entrevistador: Si el participante indica que no usó oxycontin **en [LCOUNTRY] en los últimos 6 meses, marque No corresponde.**
(Marque todas las que apliquen)

__ Inyectado

__ Fumado (chasing)

__ Aspirado

__ Inhalado

__ Tomado

__ Rectal (bumping)

__ Otro

__ No corresponde

__ Se rehúsa a contestar

If B178 is greater than 1 and B178H is equal to 1 then LEA: "Solo puedes escoger la opcion "No corresponde" por si sola, verificar respuesta." and siga con P B178.

If B178G is not equal to 1, then siga con P instrucción antes de B180.

B179. Especifica de que otra manera usaste Oxycontin en los últimos 6 meses.

__ __

If B178A is not equal to 1, then siga con P instrucción antes de B181.

B180. En los últimos 6 meses cuando usaste oxycontin en [LCOUNTRY], en promedio, ¿Qué tan seguido te lo inyectaste? (Escoja una)

00	Nunca
01	1 vez al mes o menos
02	2-3 días al mes
03	Una vez a la semana
04	2-3 días a la semana
05	4-6 días a la semana
06	Una vez al día
07	2-3 veces al día
08	4 veces o mas por día
98	Se rehúsa a contestar
09	No corresponde

If B178H is equal to 1 or B178 is equal to 98, then siga con P B182.

B181. En los últimos 6 meses cuando usaste oxycontin en [LCOUNTRY], en promedio, ¿Qué tan seguido lo usaste de otras maneras (no inyectado)? (Escoja una)

00	Nunca
01	1 vez al mes o menos
02	2-3 días al mes
03	Una vez a la semana
04	2-3 días a la semana
05	4-6 días a la semana
06	Una vez al día
07	2-3 veces al día
08	4 veces o mas por día
98	Se rehúsa a contestar
09	No corresponde

B182. ¿Alguna vez has usado otros opioides de receta (Vicodin, Darvon, Percocet)?

1	Si
0	No
8	Se rehúsa a contestar

If B182 is not equal to 1, then siga con P B190.

B183. ¿Qué edad tenías la primera vez que usaste opioides de receta (Vicodin, Darvon, Percocet)?

__ __	años de edad
98	Se rehúsa a contestar

If B183 is less than B73 or B183 is greater than A1 and B183 is not equal to 98 then LEA: "La respuesta es menor que la edad en que el participante usó drogas por primera vez o supera la edad del participante. Por favor revise la respuesta." and siga con P B183.

B184. ¿Alguna vez te has inyectado opioides de receta (Vicodin, Darvon, Percocet)?

1	Si
0	No
8	Se rehúsa a contestar

If B184 is not equal to 1, then siga con P B186.

B185. ¿Qué edad tenías la primera vez que te inyectaste opioides de receta (Vicodin, Darvon, Percocet)?

__ __	años de edad
98	Se rehúsa a contestar

If B185 is less than B76 or B185 is greater than A1 and B185 is not equal to 98 then LEA: "La respuesta es menor que la edad en que el participante se inyecta drogas por primera vez o supera la edad del participante. Por favor revise la respuesta." and siga con P B185.

B186. Si usaste opioides de receta (Vicodin, Darvon, Percocet) en los últimos 6 meses en [LCOUNTRY], ¿Cómo los usaste?
Entrevistador: Si el participante indica que no usó opioides de receta **en [LCOUNTRY] en los últimos 6 meses, marque No corresponde.**
(Marque todas las que apliquen)

__	Inyectado
__	Fumado (chasing)
__	Aspirado
__	Inhalado
__	Tomado
__	Rectal (bumping)
__	Otro
__	No corresponde
__	Se rehúsa a contestar

If B186 is greater than 1 and B186H is equal to 1 then LEA: "Solo puedes escoger la opcion "No corresponde" por si sola, verificar respuesta." and siga con P B186.

If B186G is not equal to 1, then siga con P instrucción antes de B188.

B187. Por favor, especifica de que otra manera has usado opioides de receta (Vicodin, Darvon, Percocet) en los últimos 6 meses.

____

If B186A is not equal to 1, then siga con P instrucción antes de B189.

B188. En los últimos 6 meses cuando usaste opioides de receta (Vicodin, Darvon, Percocet) en [LCOUNTRY], en promedio, ¿Qué tan seguido te los inyectaste? (Escoja una)

00	Nunca
01	1 vez al mes o menos
02	2-3 días al mes
03	Una vez a la semana
04	2-3 días a la semana
05	4-6 días a la semana
06	Una vez al día
07	2-3 veces al día
08	4 veces o mas por día
98	Se rehúsa a contestar
09	No corresponde

If B186H is equal to 1 or B186 is equal to 98, then siga con P B190.

B189. En los últimos 6 meses cuando usaste opioides de receta (Vicodin, Darvon, Percocet) en [LCOUNTRY], en promedio, ¿Qué tan seguido lo usaste de otra manera (no inyectado)? (Escoja una)

00	Nunca
01	1 vez al mes o menos
02	2-3 días al mes
03	Una vez a la semana
04	2-3 días a la semana
05	4-6 días a la semana
06	Una vez al día
07	2-3 veces al día
08	4 veces o mas por día
98	Se rehúsa a contestar

B190. ¿Alguna vez has usado drogas alucinógenas como ácido, LSD, peyote, mezcalina, hongos?

1	Si
0	No
8	Se rehúsa a contestar

If B190 is not equal to 1, then siga con P B193.

B191. ¿Qué edad tenías la <u>primera</u> vez que usaste drogas alucinógenas como ácido, LSD, peyote, mezcalina, hongos?

__ __	años de edad
98	Se rehúsa a contestar

If B191 is less than B73 or B191 is greater than A1 and B191 is not equal to 98 then LEA: "La respuesta es menor que la edad en que el participante usó drogas por primera vez o supera la edad del participante. Por favor revise la respuesta." and siga con P B191.

B192. En los últimos 6 meses, en promedio, ¿qué tan seguido usaste drogas alucinógenas como ácido, LSD, peyote, mezcalina, hongos mientras estabas en [LCOUNTRY]? (Escoja una)

00	Nunca
01	1 vez al mes o menos
02	2-3 días al mes
03	Una vez a la semana
04	2-3 días a la semana
05	4-6 días a la semana
06	Una vez al día
07	2-3 veces al día
08	4 veces o mas por día
98	Se rehúsa a contestar

B193. ¿Alguna vez has usado inhalantes (goma resistol o gasolina)?

1	Si
0	No
8	Se rehúsa a contestar

If B193 is not equal to 1, then siga con P B196.

B194. ¿Qué edad tenías la primera vez que usaste inhalantes (goma resistol o gasolina)?

__ __	años de edad
98	Se rehúsa a contestar

If B194 is less than B73 or B194 is greater than A1 and B194 is not equal to 98 then LEA: "La respuesta es menor que la edad en que el participante usó drogas por primera vez o supera la edad del participante. Por favor revise la respuesta." and siga con P B194.

B195. Durante los últimos 6 meses, en promedio que tan seguido usaste inhalantes (goma resistol o gasolina) cuando estuviste en [LCOUNTRY]? (Escoja una)

00	Nunca
01	1 vez al mes o menos
02	2-3 días al mes
03	Una vez a la semana
04	2-3 días a la semana
05	4-6 días a la semana
06	Una vez al día
07	2-3 veces al día
08	4 veces o mas por día
98	Se rehúsa a contestar

B196. ¿Alguna vez has usado tranquilizantes (Diazepam, Valium, Ativan, Restori o Rivotril)?

1	Si
0	No
8	Se rehúsa a contestar

If B196 is not equal to 1, then siga con P B204.

B197. ¿Qué edad tenías la primera vez que usaste tranquilizantes (Diazepam, Valium, Ativan, Restori o Rivotril)?

__ __	años de edad
98	Se rehúsa a contestar

If B197 is less than B73 or B197 is greater than A1 and B197 is not equal to 98 then LEA: "La respuesta es menor que la edad en que el participante usó drogas por primera vez o supera la edad del participante. Por favor revise la respuesta." and siga con P B197.

B198. ¿Alguna vez te has inyectado tranquilizantes (Diazepam, Valium, Ativan, Restori o Rivotril)?

1	Si
0	No
8	Se rehúsa a contestar

If B198 is not equal to 1, then siga con P B200.

B199. ¿Qué edad tenías la primera vez que te inyectaste tranquilizantes (Diazepam, Valium, Ativan Restori o Rivotril)?

__ __	años de edad
98	Se rehúsa a contestar

If B199 is less than B76 or B199 is greater than A1 and B199 is not equal to 98 then LEA: "La respuesta es menor que la edad en que el participante se inyecta drogas por primera vez o supera la edad del participante. Por favor revise la respuesta." and siga con P B199.

B200. Si usaste tranquilizantes (Diazepam, Valium, Ativan, Restori o Rivotril) en los últimos 6 meses cuando estuviste en [LCOUNTRY], ¿Cómo los usaste?
Entrevistador: Si el participante indica que no usó tranquilizantes **en [LCOUNTRY] en los últimos 6 meses, marque No corresponde.**
(Marque todas las que apliquen)

__	Inyectado
__	Fumado (chasing)
__	Aspirado
__	Inhalado
__	Tomado
__	Rectal (bumping)
__	Otro
__	No corresponde
__	Se rehúsa a contestar

If B200 is greater than 1 and B200H is equal to 1 then LEA: "Solo puedes escoger la opcion "No corresponde" por si sola, verificar respuesta." and siga con P B200.

If B200G is not equal to 1, then siga con P instrucción antes de B202.

B201. Especifica de que otra manera has usado tranquilizantes (Diazepam, Valium, Ativan, Restori o Rivotril) en los últimos 6 meses.

____

If B200A is not equal to 1, then siga con P instrucción antes de B203.

B202. Durante los últimos 6 meses, cuando usaste tranquilizantes (Diazepam, Valium, Ativan, Restori o Rivotril) en promedio ¿Qué tan seguido te inyectaste cuando estuviste en [LCOUNTRY]? (Escoja una)

00	Nunca
01	1 vez al mes o menos
02	2-3 días al mes
03	Una vez a la semana
04	2-3 días a la semana
05	4-6 días a la semana
06	Una vez al día
07	2-3 veces al día
08	4 veces o mas por día
98	Se rehúsa a contestar

If B200H is equal to 1 or B200 is equal to 98, then siga con P B204.

B203. Durante los últimos 6 meses, cuando usaste tranquilizantes (Diazepam, Valium, Ativan o Restori, Rivotril) en promedio ¿Qué tan seguido los usaste de otras maneras cuando estuviste en [LCOUNTRY]? (Escoja una)

00	Nunca
01	1 vez al mes o menos
02	2-3 días al mes
03	Una vez a la semana
04	2-3 días a la semana
05	4-6 días a la semana
06	Una vez al día
07	2-3 veces al día
08	4 veces o mas por día
98	Se rehúsa a contestar

B204. ¿Alguna vez haz usado barbitúricos (Amytal, Nembutal, o Seconal)?

1	Si
0	No
8	Se rehúsa a contestar

If B204 is not equal to 1, then siga con P B212.

B205. ¿Qué edad tenías la primera vez que usaste barbitúricos (Amytal, Nembutal, o Seconal)?

__ __	años de edad
98	Se rehúsa a contestar

If B205 is less than B73 or B205 is greater than A1 and B205 is not equal to 98 then LEA: "La respuesta es menor que la edad en que el participante usó drogas por primera vez o supera la edad del participante. Por favor revise la respuesta." and siga con P B205.

B206. ¿Alguna vez te has inyectado barbitúricos (Amytal, Nembutal, o Seconal)?

1 Si

0 No

8 Se rehúsa a contestar

If B206 is not equal to 1, then siga con P B208.

B207. ¿Qué edad tenías la primera vez que te inyectaste barbitúricos (Amytal, Nembutal, o Seconal)?

__ __ años de edad

98 Se rehúsa a contestar

If B207 is less than B76 or B207 is greater than A1 and B207 is not equal to 98 then LEA: "La respuesta es menor que la edad en que el participante se inyecta drogas por primera vez o supera la edad del participante. Por favor revise la respuesta." and siga con P B207.

B208. Si usaste barbitúricos (Amytal, Nembutal, o Seconal) en los últimos 6 meses mientras estuviste en [LCOUNTRY], ¿Cómo los usaste?
Entrevistador: Si el participante indica que no usó barbitúricos **en [LCOUNTRY] en los últimos 6 meses, marque No corresponde.**
(Marque todas las que apliquen)

__ Inyectado

__ Fumado (chasing)

__ Aspirado

__ Inhalado

__ Tomado

__ Rectal (bumping)

__ Otro

__ No corresponde

__ Se rehúsa a contestar

If B208 is greater than 1 and B208H is equal to 1 then LEA: "Solo puedes escoger la opcion "No corresponde" por si sola, verificar respuesta." and siga con P B208.

If B208G is not equal to 1, then siga con P instrucción antes de B210.

B209. Especifica de que otra manera usaste barbitúricos (Amytal, Nembutal, o Seconal) en los últimos 6 meses.

____

If B208A is not equal to 1, then siga con P instrucción antes de B211.

B210. Durante los últimos 6 meses, cuando usaste barbitúricos (Amytal, Nembutal, o Seconal) cuando estuviste en [LCOUNTRY], en promedio ¿Qué tan seguido te los inyectaste? (Escoja una)

00	Nunca
01	1 vez al mes o menos
02	2-3 días al mes
03	Una vez a la semana
04	2-3 días a la semana
05	4-6 días a la semana
06	Una vez al día
07	2-3 veces al día
08	4 veces o mas por día
98	Se rehúsa a contestar

If B208H is equal to 1 or B208 is equal to 98, then siga con P B212.

B211. Durante los últimos 6 meses, cuando usaste barbitúricos (Amytal, Nembutal, o Seconal) cuando estuviste en [LCOUNTRY], en promedio ¿Qué tan seguido lo usaste de otra manera (no inyectada)? (Escoja una)

00	Nunca
01	1 vez al mes o menos
02	2-3 días al mes
03	Una vez a la semana
04	2-3 días a la semana
05	4-6 días a la semana
06	Una vez al día
07	2-3 veces al día
08	4 veces o mas por día
98	Se rehúsa a contestar

B212. ¿Alguna vez has usado píldoras del cerebro (nootropicos, modafilino, ritalin)?

1	Si
0	No
8	Se rehúsa a contestar

If B212 is not equal to 1, then siga con P B220.

B213. ¿Qué edad tenías la primera vez que usaste píldoras del cerebro (nootropicos, modafilino, ritalin)?

__ __	años de edad
98	Se rehúsa a contestar

If B213 is less than B73 or B213 is greater than A1 and B213 is not equal to 98 then LEA: "La respuesta es menor que la edad en que el participante usó drogas por primera vez o supera la edad del participante. Por favor revise la respuesta." and siga con P B213.

B214. ¿Alguna vez te has inyectado píldoras del cerebro (nootropicos, modafilino, ritalin)?

1	Si
0	No
8	Se rehúsa a contestar

If B214 is not equal to 1, then siga con P B216.

B215. ¿Qué edad tenías la primera vez que te inyectaste píldoras del cerebro (nootropicos, modafilino, ritalin)?

__ __	años de edad
98	Se rehúsa a contestar

If B215 is less than B76 or B215 is greater than A1 and B215 is not equal to 98 then LEA: "La respuesta es menor que la edad en que el participante se inyecta drogas por primera vez o supera la edad del participante. Por favor revise la respuesta." and siga con P B215.

B216. Si usaste píldoras del cerebro (nootropicos, modafilino, ritalin) en los últimos 6 meses cuando estuviste en [LCOUNTRY], ¿Cómo los usaste?
Entrevistador: Si el participante indica que no usó píldoras del cerebro **en [LCOUNTRY] en los últimos 6 meses, marque No corresponde.**
(Marque todas las que apliquen)

__	Inyectado
__	Fumado (chasing)
__	Aspirado
__	Inhalado
__	Tomado
__	Rectal (bumping)
__	Otro
__	No corresponde
__	Se rehúsa a contestar

If B216 is greater than 1 and B216H is equal to 1 then LEA: "Solo puedes escoger la opcion "No corresponde" por si sola, verificar respuesta." and siga con P B216.

If B216G is not equal to 1, then siga con P instrucción antes de B218.

B217. Especifica otras maneras en las que usaste píldoras del cerebro (nootropicos, modafilino, ritalin) en los últimos 6 meses.

____

If B216A is not equal to 1, then siga con P instrucción antes de B219.

B218. En los últimos 6 meses, cuando usaste píldoras del cerebro (nootropicos, modafilino, ritalin) en [LCOUNTRY], en promedio ¿Qué tan seguido te inyectabas? (Escoja una)

00	Nunca
01	1 vez al mes o menos
02	2-3 días al mes
03	Una vez a la semana
04	2-3 días a la semana
05	4-6 días a la semana
06	Una vez al día
07	2-3 veces al día
08	4 veces o mas por día
98	Se rehúsa a contestar

If B216H is equal to 1 or B216 is equal to 98, then siga con P B220.

B219. En los últimos 6 meses, cuando usaste píldoras del cerebro (nootropicos, modafilino, ritalin) en [LCOUNTRY], en promedio ¿Qué tan seguido las usaste de otra manera (no inyectada)? (Escoja una)

00	Nunca
01	1 vez al mes o menos
02	2-3 días al mes
03	Una vez a la semana
04	2-3 días a la semana
05	4-6 días a la semana
06	Una vez al día
07	2-3 veces al día
08	4 veces o mas por día
98	Se rehúsa a contestar

B220. ¿Alguna vez has usado otra droga sola (no mencionada anteriormente)? **Entrevistador, por favor pregunte sobre sales de baño**

1	Si
0	No
8	Se rehúsa a contestar

If B220 is not equal to 1, then siga con P B238.

B221. Por favor especifica que otra droga has usado sola.

__ __
__ __ __ __ __ __ __ __ __ __ __ __ __ __

B222. ¿Qué edad tenías la primera vez que usaste [Respuesta a P B221] sola?

__ __	años de edad
98	Se rehúsa a contestar

If B222 is less than B73 or B222 is greater than A1 and B222 is not equal to 98 then LEA: "La respuesta es menor que la edad en que el participante usó drogas por primera vez o supera la edad del participante. Por favor revise la respuesta." and siga con P B222.

B223. ¿Alguna vez te has inyectado [Respuesta a P B221]?

1	Si
0	No
8	Se rehúsa a contestar

If B223 is not equal to 1, then siga con P B225.

B224. ¿Qué edad tenías la primera vez que te inyectaste [Respuesta a P B221]?

__ __	años de edad
98	Se rehúsa a contestar

If B224 is less than B76 or B224 is greater than A1 and B224 is not equal to 98 then LEA: "La respuesta es menor que la edad en que el participante se inyecta drogas por primera vez o supera la edad del participante. Por favor revise la respuesta." and siga con P B224.

B225. Si usaste [Respuesta a P B221] en los últimos 6 meses mientras estuviste en [LCOUNTRY], ¿Cómo los usaste?
Entrevistador: Si el participante indica que no usó [Respuesta a P B221] **en [LCOUNTRY] en los últimos 6 meses, marque No corresponde.**
(Marque todas las que apliquen)

__	Inyectado
__	Fumado (chasing)
__	Aspirado
__	Inhalado
__	Tomado
__	Rectal (bumping)
__	Otro
__	No corresponde
__	Se rehúsa a contestar

If B225 is greater than 1 and B225H is equal to 1 then LEA: "Solo puedes escoger la opcion "No corresponde" por si sola, verificar respuesta." and siga con P B225.

If B225G is not equal to 1, then siga con P instrucción antes de B227.

B226. Especifica de que otra manera usaste [Respuesta a P B221] en los últimos 6 meses.

____

If B225A is not equal to 1, then siga con P instrucción antes de B228.

B227. Durante los últimos 6 meses, cuando usaste [Respuesta a P B221] cuando estuviste en [LCOUNTRY], en promedio ¿Qué tan seguido te la inyectaste? (Escoja una)

00	Nunca
01	1 vez al mes o menos
02	2-3 días al mes
03	Una vez a la semana
04	2-3 días a la semana
05	4-6 días a la semana
06	Una vez al día
07	2-3 veces al día
08	4 veces o mas por día
98	Se rehúsa a contestar

If B225H is equal to 1 or B225 is equal to 98, then siga con P B229.

B228. Durante los últimos 6 meses, cuando usaste [Respuesta a P B221] cuando estuviste en [LCOUNTRY], en promedio ¿Qué tan seguido lo usaste de otra manera (no inyectada)? (Escoja una)

00	Nunca
01	1 vez al mes o menos
02	2-3 días al mes
03	Una vez a la semana
04	2-3 días a la semana
05	4-6 días a la semana
06	Una vez al día
07	2-3 veces al día
08	4 veces o mas por día
98	Se rehúsa a contestar

B229. ¿Alguna vez has usado otra droga sola (no mencionada anteriormente)?

1	Si
0	No
8	Se rehúsa a contestar

If B229 is not equal to 1, then siga con P B238.

B230. Por favor especifica otra droga que has usado sola.

__ __

B231. ¿Qué edad tenías la primera vez que usaste [Respuesta a P B230] sola?

__ __	años de edad
98	Se rehúsa a contestar

If B231 is less than B73 or B231 is greater than A1 and B231 is not equal to 98 then LEA: "La respuesta es menor que la edad en que el participante usó drogas por primera vez o supera la edad del participante. Por favor revise la respuesta." and siga con P B231.

B232. ¿Alguna vez te has inyectado [Respuesta a P B230]?

1 Si

0 No

8 Se rehúsa a contestar

If B232 is not equal to 1, then siga con P B234.

B233. ¿Qué edad tenías la primera vez que te inyectaste [Respuesta a P B230]?

__ __ años de edad

98 Se rehúsa a contestar

If B233 is less than B76 or B233 is greater than A1 and B233 is not equal to 98 then LEA: "La respuesta es menor que la edad en que el participante se inyecta drogas por primera vez o supera la edad del participante. Por favor revise la respuesta." and siga con P B233.

B234. Si usaste [Respuesta a P B230] en los últimos 6 meses mientras estuviste en [LCOUNTRY], ¿Cómo los usaste?
Entrevistador: Si el participante indica que no usó [Respuesta a P B230] **en [LCOUNTRY] en los últimos 6 meses, marque No corresponde.**
(Marque todas las que apliquen)

__ Inyectado

__ Fumado (chasing)

__ Aspirado

__ Inhalado

__ Tomado

__ Rectal (bumping)

__ Otro

__ No corresponde

__ Se rehúsa a contestar

If B234 is greater than 1 and B234H is equal to 1 then LEA: "Solo puedes escoger la opcion "No corresponde" por si sola, verificar respuesta." and siga con P B234.

If B234G is not equal to 1, then siga con P instrucción antes de B236.

B235. Especifica de que otra manera usaste [Respuesta a P B230] en los últimos 6 meses.

____

If B234A is not equal to 1, then siga con P instrucción antes de B237.

B236. Durante los últimos 6 meses, cuando usaste [Respuesta a P B230] cuando estuviste en [LCOUNTRY], en promedio ¿Qué tan seguido te lo inyectaste? (Escoja una)

00	Nunca
01	1 vez al mes o menos
02	2-3 días al mes
03	Una vez a la semana
04	2-3 días a la semana
05	4-6 días a la semana
06	Una vez al día
07	2-3 veces al día
08	4 veces o mas por día
98	Se rehúsa a contestar

If B234H is equal to 1 or B234 is equal to 98, then siga con P B238.

B237. Durante los últimos 6 meses, cuando usaste [Respuesta a P B230] cuando estuviste en [LCOUNTRY], en promedio ¿Qué tan seguido lo usaste de otra manera (no inyectada)? (Escoja una)

00	Nunca
01	1 vez al mes o menos
02	2-3 días al mes
03	Una vez a la semana
04	2-3 días a la semana
05	4-6 días a la semana
06	Una vez al día
07	2-3 veces al día
08	4 veces o mas por día
98	Se rehúsa a contestar

B238. ¿Alguna vez has usado otra combinación de droga (no mencionada anteriormente)?

1	Si
0	No
8	Se rehúsa a contestar

If B238 is not equal to 1, then siga con P instrucción antes de B256.

B239. Por favor especifica otra combinación de droga has usado.

____

B240. ¿Qué edad tenías la primera vez que usaste [Respuesta a P B239]?

__ __	años de edad
98	Se rehúsa a contestar

If B240 is less than B73 or B240 is greater than A1 and B240 is not equal to 98 then LEA: "La respuesta es menor que la edad en que el participante usó drogas por primera vez o supera la edad del participante. Por favor revise la respuesta." and siga con P B240.

B241. ¿Alguna vez te has inyectado [Respuesta a P B239]?

1	Si
0	No
8	Se rehúsa a contestar

If B241 is not equal to 1, then siga con P B243.

B242. ¿Qué edad tenías la primera vez que te inyectaste [Respuesta a P B239]?

__ __	años de edad
98	Se rehúsa a contestar

If B242 is less than B76 or B242 is greater than A1 and B242 is not equal to 98 then LEA: "La respuesta es menor que la edad en que el participante se inyecta drogas por primera vez o supera la edad del participante. Por favor revise la respuesta." and siga con P B242.

B243. Si usaste [Respuesta a P B239] en los últimos 6 meses mientras estuviste en [LCOUNTRY], ¿Cómo los usaste?
Entrevistador: Si el participante indica que no usó [Respuesta a P B239] **en [LCOUNTRY] en los últimos 6 meses, marque No corresponde.**
(Marque todas las que apliquen)

__	Inyectado
__	Fumado (chasing)
__	Aspirado
__	Inhalado
__	Tomado
__	Rectal (bumping)
__	Otro
__	No corresponde
__	Se rehúsa a contestar

If B243 is greater than 1 and B243H is equal to 1 then LEA: "Solo puedes escoger la opcion "No corresponde" por si sola, verificar respuesta." and siga con P B243.

If B243G is not equal to 1, then siga con P instrucción antes de B245.

B244. Especifica de que otra manera usaste [Respuesta a P B239] en los últimos 6 meses.

____

If B243A is not equal to 1, then siga con P instrucción antes de B246.

B245. Durante los últimos 6 meses, cuando usaste [Respuesta a P B239] cuando estuviste en [LCOUNTRY], en promedio ¿Qué tan seguido te los inyectaste? (Escoja una)

00	Nunca
01	1 vez al mes o menos
02	2-3 días al mes
03	Una vez a la semana
04	2-3 días a la semana
05	4-6 días a la semana
06	Una vez al día
07	2-3 veces al día
08	4 veces o mas por día
98	Se rehúsa a contestar

If B243H is equal to 1 or B243 is equal to 98, then siga con P B247.

B246. Durante los últimos 6 meses, cuando usaste [Respuesta a P B239] cuando estuviste en [LCOUNTRY], en promedio ¿Qué tan seguido lo usaste de otra manera (no inyectada)? (Escoja una)

00	Nunca
01	1 vez al mes o menos
02	2-3 días al mes
03	Una vez a la semana
04	2-3 días a la semana
05	4-6 días a la semana
06	Una vez al día
07	2-3 veces al día
08	4 veces o mas por día
98	Se rehúsa a contestar

B247. ¿Alguna vez has usado otra droga sola (no mencionada anteriormente)?

1	Si
0	No
8	Se rehúsa a contestar

If B247 is not equal to 1, then siga con P instrucción antes de B256.

B248. Por favor especifica otra droga que has usado sola.

____

B249. ¿Qué edad tenías la primera vez que usaste [Respuesta a P B248]?

__ __	años de edad
98	Se rehúsa a contestar

If B249 is less than B73 or B249 is greater than A1 and B249 is not equal to 98 then LEA: "La respuesta es menor que la edad en que el participante usó drogas por primera vez o supera la edad del participante. Por favor revise la respuesta." and siga con P B249.

B250. ¿Alguna vez te has inyectado [Respuesta a P B248]?

1	Si
0	No
8	Se rehúsa a contestar

If B250 is not equal to 1, then siga con P B252.

B251. ¿Qué edad tenías la primera vez que te inyectaste [Respuesta a P B248]?

__ __	años de edad
98	Se rehúsa a contestar

If B251 is less than B76 or B251 is greater than A1 and B251 is not equal to 98 then LEA: "La respuesta es menor que la edad en que el participante se inyecta drogas por primera vez o supera la edad del participante. Por favor revise la respuesta." and siga con P B251.

B252. Si usaste [Respuesta a P B248] en los últimos 6 meses mientras estuviste en [LCOUNTRY], ¿Cómo los usaste?
Entrevistador: Si el participante indica que no usó [Respuesta a P B248] **en [LCOUNTRY] en los últimos 6 meses, marque No corresponde.**
(Marque todas las que apliquen)

__	Inyectado
__	Fumado (chasing)
__	Aspirado
__	Inhalado
__	Tomado
__	Rectal (bumping)
__	Otro
__	No corresponde
__	Se rehúsa a contestar

If B252 is greater than 1 and B252H is equal to 1 then LEA: "Solo puedes escoger la opcion "No corresponde" por si sola, verificar respuesta." and siga con P B252.

If B252G is not equal to 1, then siga con P instrucción antes de B254.

B253. Por favor, especifica de que otra manera usaste [Respuesta a P B248] en los últimos 6 meses.

____

If B252A is not equal to 1, then siga con P instrucción antes de B255.

B254. Durante los últimos 6 meses, cuando usaste [Respuesta a P B248] cuando estuviste en [LCOUNTRY], en promedio ¿Qué tan seguido te los inyectaste? (Escoja una)

00	Nunca
01	1 vez al mes o menos
02	2-3 días al mes
03	Una vez a la semana
04	2-3 días a la semana
05	4-6 días a la semana
06	Una vez al día
07	2-3 veces al día
08	4 veces o mas por día
98	Se rehúsa a contestar

If B252H is equal to 1 or B252 is equal to 98, then siga con P instrucción antes de B256.

B255. Durante los últimos 6 meses, cuando usaste [Respuesta a P B248] cuando estuviste en [LCOUNTRY], en promedio ¿Qué tan seguido lo usaste de otra manera (no inyectada)? (Escoja una)

00	Nunca
01	1 vez al mes o menos
02	2-3 días al mes
03	Una vez a la semana
04	2-3 días a la semana
05	4-6 días a la semana
06	Una vez al día
07	2-3 veces al día
08	4 veces o mas por día
98	Se rehúsa a contestar

ANYINJ6M = If(B92 is not equal to 0 and B92 is not equal to 98 and B92 is not equal to "Salto" or B106 is not equal to 0 and B106 is not equal to 98 and B106 is not equal to "Salto" or B109 is not equal to 0 and B109 is not equal to 98 and B109 is not equal to "Salto" or B117 is not equal to 0 and B117 is not equal to 98 and B117 is not equal to "Salto" or B124 is not equal to 0 and B124 is not equal to 98 and B124 is not equal to "Salto" or B127 is not equal to 0 and B127 is not equal to 98 and B127 is not equal to "Salto" or B136 is not equal to 0 and B136 is not equal to 98 and B136 is not equal to "Salto" or B139 is not equal to 0 and B139 is not equal to 98 and B139 is not equal to "Salto" or B146 is not equal to 0 and B146 is not equal to 98 and B146 is not equal to "Salto" or B153 is not equal to 0 and B153 is not equal to 98 and B153 is not equal to "Salto" or B164 is not equal to 0 and B164 is not equal to 98 and B164 is not equal to "Salto" or B172 is not equal to 0 and B172 is not equal to 98 and B172 is not equal to "Salto" or B180 is not equal to 0 and B180 is not equal to 98 and B180 is not equal to "Salto" or B188 is not equal to 0 and B188 is not equal to 98 and B188 is not equal to "Salto" or B202 is not equal to 0 and B202 is not equal to 98 and B202 is not equal to "Salto" or B210 is not equal to 0 and B210 is not equal to 98 and B210 is not equal to "Salto" or B218 is not equal to 0 and B218 is not equal to 98 and B218 is not equal to "Salto" or B227 is not equal to 0 and B227 is not equal to 98 and B227 is not equal to "Salto" or B236 is not equal to 0 and B236 is not equal to 98 and B236 is not equal to "Salto" or B245 is not equal to 0 and B245 is not equal to 98 and B245 is not equal to "Salto" or B254 is not equal to 0 and B254 is not equal to 98 and B254 is not equal to "Salto", 1, 0)

If ANYINJ6M is equal to 0 then LEA: "El resumen de respuestas a las preguntas sobre drogas descalifican al/la participante de inscribirse al estudio. Por favor verificar respuestas al verificar cada pregunta sobre drogas de nuevo.
" and siga con P B86.

LEA: ENTREVISTADOR LEA AL PARTICIPANTE: Las siguientes preguntas tienen que ver con inyectarse y comportamiento de compartir. Yo sé que te pregunte antes pero, me podrías recordar...

B256. Durante los últimos 6 meses, en promedio en [LCOUNTRY]? ¿Qué tan seguido te inyectaste cualquier droga o combinación de drogas? (Escoja una)

01 1 vez al mes o menos

02 2-3 días al mes

03 Una vez a la semana

04 2-3 días a la semana

05 4-6 días a la semana

06 Una vez al día

07 2-3 veces al día

08 4 veces o mas por día

98 Se rehúsa a contestar

B257. De las veces que se inyectó en los **últimos 6 meses cuando estuviste** en [LCOUNTRY]**,** ¿qué tan seguido le dio, rentó, prestó a alguien una jeringa que usted ya había usado? (Escoja una)

1 Nunca

2 A veces

3 Casi la mitad de las ocasiones

4 Seguido

5 Siempre

8 Se rehúsa a contestar

B258. De las veces que se inyectó en los **últimos 6 meses cuando estuviste** en [LCOUNTRY], ¿qué tan seguido uso una jeringa que usted sabía o sospechaba que alguien más ya la había usado? (Escoja una)

1 Nunca

2 A veces

3 Casi la mitad de las ocasiones

4 Seguido

5 Siempre

8 Se rehúsa a contestar

If B258 is equal to 1 or B258 is equal to 8, then siga con P B261.

B259. Durante la última vez que se inyecto drogas en [LCOUNTRY], ¿uso una jeringa que usted sabia o sospechaba hubiera sido usada por alguien más?

1 Si

0 No

8 Se rehúsa a contestar

B260. De las veces en los **últimos 6 meses** que se inyectó en [LCOUNTRY] con una jeringa que ya había sido usada por alguien más, ¿qué tan seguido limpió la jeringa con cloro antes de usarla? (Escoja una)

1 Nunca

2 Menos de la mitad del tiempo

3 Casi la mitad del tiempo

4 Más de la mitad del tiempo

5 Siempre

8 Se rehúsa a contestar

B261. De las veces que se inyectó en [LCOUNTRY] con otras personas en los **últimos 6 meses**, ¿qué tan seguido ha dividido drogas con alguien más usando una jeringa? Esto también es conocido como backloading, piggybacking o dividir drogas mojadas. (Escoja una)

1 Nunca

2 Menos de la mitad del tiempo

3 Casi la mitad del tiempo

4 Más de la mitad del tiempo

5 Siempre

8 Se rehúsa a contestar

B262. De las veces que se inyectó en los **últimos 6 meses mientras estuvo** en [LCOUNTRY], ¿qué tan seguido usó un cacharro, algodón o agua de enjuague con alguien o después que alguien más ya lo había usado? (Escoja una)

1 Nunca

2 Menos de la mitad del tiempo

3 Casi la mitad del tiempo

4 Más de la mitad del tiempo

5 Siempre

8 Se rehúsa a contestar

B263. De las veces que se inyectó en los **últimos 6 meses mientras estuviste** en [LCOUNTRY], ¿qué tan seguido compró drogas que ya venían preparadas en la jeringa? (Escoja una)

1 Nunca

2 Menos de la mitad del tiempo

3 Casi la mitad del tiempo

4 Más de la mitad del tiempo

5 Siempre

8 Se rehúsa a contestar

B264. De las veces que se inyectó en los **últimos 6 meses mientras estuviste** en [LCOUNTRY], ¿qué tan seguido se inyectó con una jeringa nueva, estéril? (Escoja una)

1 Nunca

2 Menos de la mitad del tiempo

3 Casi la mitad del tiempo

4 Más de la mitad del tiempo

5 Siempre

8 Se rehúsa a contestar

B265. En los últimos 6 meses que se inyectó drogas mientras estuvo en [LCOUNTRY], ¿dónde consiguió la jeringa que Ud. uso para inyectarse drogas? (Marque todas las que apliquen)

__ Farmacia

__ Esposo (a), familiar, o pareja sexual

__ Amigo (a)

__ Conocido/desconocido

__ Un Traficante

__ Doctor que ayuda a inyectarse a cambio de drogas o dinero

__ Picadero

__ Intercambio de Jeringas (usted lo cambió)

__ De alguien que los obtiene de un intercambio de Jeringas

__ Comprada en la calle

__ Doctor / clínica / hospital

__ Clínica Veterinaria/ tienda de mascotas

__ Mercado/Tienda

__ Encontrada en el suelo o la basura

__ Otro lugar

__ Se rehúsa a contestar

If B265O is equal to 1 then siga con P B266

If not, then siga con P instrucción antes de B267.

B266. Por favor especifique en qué otro lugar usted consiguió las jeringas en los últimos 6 meses.

____

If B265A + B265B + B265C + B265D + B265E + B265F + B265G + B265H + B265I + B265J + B265K + B265L + B265M + B265N + B265O is not equal to 1 then siga con P B267

If not, then siga con P instrucción antes de B269.

B267. En los últimos 6 meses mientras estuvo en [LCOUNTRY], ¿dónde consiguió sus jeringas más seguido? (Escoja una)

01	Farmacia
02	Esposo (a), familiar, o pareja sexual
03	Amigo (a)
04	Conocido/desconocido
05	Un Traficante
06	Doctor que ayuda a inyectarse a cambio de drogas o dinero
07	Picadero
08	Intercambio de Jeringas (usted lo cambió)
09	De alguien que los obtiene de un intercambio de Jeringas
10	Comprada en la calle
11	Doctor / clínica / hospital
12	Clínica Veterinaria/ tienda de mascotas
13	Mercado/Tienda
14	Encontrada en el suelo o la basura
15	Otro lugar
98	Se rehúsa a contestar

If B267 is equal to 1 and B265A is not equal to 1 or B267 is equal to 2 and B265B is not equal to 1 or B267 is equal to 3 and B265C is not equal to 1 or B267 is equal to 4 and B265D is not equal to 1 or B267 is equal to 5 and B265E is not equal to 1 or B267 is equal to 6 and B265F is not equal to 1 or B267 is equal to 7 and B265G is not equal to 1 or B267 is equal to 8 and B265H is not equal to 1 or B267 is equal to 9 and B265I is not equal to 1 or B267 is equal to 10 and B265J is not equal to 1 or B267 is equal to 11 and B265K is not equal to 1 or B267 is equal to 12 and B265L is not equal to 1 or B267 is equal to 13 and B265M is not equal to 1 or B267 is equal to 14 and B265N is not equal to 1 or B267 is equal to 15 and B265O is not equal to 1 then LEA: "El participante solo puede elegir el lugar con más frecuencia de entre los lugares nombrados anteriormente." and siga con P B267.

If B267 is equal to 15 then siga con P B268

If not, then siga con P instrucción antes de B269.

B268. Por favor especifique en qué lugar usted consiguió sus jeringas más seguido, en los últimos 6 meses.

____

If B267 is equal to 1 or B267 is equal to 8 then siga con P B269

If not, then siga con P instrucción antes de B271.

B269. En los últimos 6 meses, cuando estuviste en [LCOUNTRY], ¿cuantas jeringas estériles y limpias obtenías por visita al intercambio de jeringas en promedio?

___ ___ ___

998 Se rehúsa a contestar

If B269 is equal to 0 or B269 is equal to 998, then siga con P instrucción antes de B271.

B270. ¿Cuál es el nombre del programa de intercambio de jeringa de donde obtuviste tus jeringas mas seguido?

____

If B265A is equal to 1 or B267 is equal to 1 then siga con P B271

If not, then siga con P B273.

B271. En los últimos 6 meses, cuando estuviste en [LCOUNTRY], ¿cuantas jeringas estériles y limpias obtuviste en cada visita a la farmacia en promedio?

___ ___ ___

998 Se rehúsa a contestar

If B271 is equal to 0 or B271 is equal to 998, then siga con P B273.

B272. ¿Cuál es el nombre de la farmacia en la que compraste jeringas mas seguido?

____

B273. En los últimos 6 meses mientras estuvo en [LCOUNTRY], cuando se inyectaba drogas ¿qué tan difícil o fácil fue conseguir jeringas nuevas, que no han sido usadas? (Escoja una)

1 Nunca ha tratado de conseguir jeringas nuevas que no han sido usadas

2 Bastante fácil

3 Fácil

4 Difícil

5 Bastante difícil

8 Se rehúsa a contestar

If B273 is equal to 4 or B273 is equal to 5 then siga con P B274

If not, then siga con P instrucción antes de B275.

B274. ¿Cuál fue la razón principal por la que fue difícil conseguir jeringas nuevas, que no han sido usadas mientras estuvo en [LCOUNTRY]? (Escoja una)

1 Cuestan demasiado/ No tengo dinero para comprarlas

2 No hay ningún sitio donde comprar jeringas legalmente/ la tienda no me los vende a mi

3 No hay ningún lugar de intercambio de jeringas

4 Me da miedo que me encuentren /arresten la policía por llevar jeringas

5 La farmacia/tienda estaba cerrada

6 Otro

7 No sé

8 Se rehúsa a contestar

If B274 is equal to 6 then siga con P B275

If not, then siga con P B276.

B275. Por favor especifique cuál fue la otra razón por la cual fue difícil conseguir jeringas nuevas, que no han sido usadas.

____

B276. Mientras estuvo en [LCOUNTRY], ¿le ha pedido ayuda a alguien como a un ¨doctor¨ para ayudarle a inyectarse? **(alguien que le ayuda a inyectarse a cambio de drogas o dinero)**

1	Si
0	No
7	No sé
8	Se rehúsa a contestar

If B276 is not equal to 1, then siga con P instrucción antes de B281.

B277. ¿Cuándo fue la última vez que alguien, como un "doctor", le ayudó a inyectarse drogas en [LCOUNTRY]?

__ __ __	MESES
__ __ __	SEMANAS
__ __ __	DíAS
998	Se rehúsa a contestar (Meses)

If B277 is greater than A1 and B277 is not equal to 998 then LEA: "La respuesta supera la edad del participante. Por favor verificar respuesta." and siga con P B277.

If B277 is greater than 6 and B277 is not equal to 98, then siga con P instrucción antes de B281.

B278. En los últimos 6 meses cuando estuvo en [LCOUNTRY], ¿qué tan seguido se ha inyectado con la ayuda de alguien? (Escoja una)

1	Siempre
2	Con frecuencia
3	A veces
4	Casi nunca
8	Se rehúsa a contestar

B279. En los ultimos 6 meses, cuando alguien te ayudo a inyectarte, ¿en qué países ocurrió? (Marque todas las que apliquen)

__ EUA

__ Mexico

__ Otro

__ Se rehúsa a contestar

If B279C is not equal to 1, then siga con P instrucción antes de B281.

B280. Especifique en qué otro pais

____

SECCION C: USO DE DROGAS

LEA: ENTREVISTADOR LEA: En la siguiente sección vamos a hablar sobre el precio, pureza y disponibilidad de drogas en las calles y si ha habido cambios en [LCITY]. Te preguntaremos sobre drogas específicas y vamos a hablar sobre precios primero y luego sobre la pureza y al final sobre la disponibilidad. Te voy a preguntar sobre drogas específicas y me gustaría que me proporciones tu opinión aunque no hayas usado alguna droga en específico.

B281. **Por favor selecciona el tipo de moneda con la que sabes el precio la/las droga/s** (Escoja una)

1	Pesos
2	Dolares Estadunidenses
8	Se rehúsa a contestar

If B281 is equal to 2 then siga con P B282

If not, then siga con P instrucción antes de B297.

B282. En dólares: ¿Cuánto cuesta comprar una dosis de Heroína en goma sola en [LCITY]?

__ __ __	dollars
997	No sé
998	Se rehúsa a contestar

B283. En dólares: ¿Cuánto cuesta comprar una dosis de Heroína en polvo sola en el condado de [LCITY]?

__ __ __	dollars
997	No sé
998	Se rehúsa a contestar

B284. En dólares: ¿Cuánto cuesta comprar una dosis de Crack sola en [LCITY]?

__ __ __	dollars
997	No sé
998	Se rehúsa a contestar

B285. En dólares: ¿Cuánto cuesta comprar una dosis de Cocaína (polvo) sola en [LCITY]?

__ __ __	dollars
997	No sé
998	Se rehúsa a contestar

B286. En dólares: ¿Cuánto cuesta comprar una dosis de Heroína y cocaína juntas (speedball) en [LCITY]?

__ __ __	dollars
997	No sé
998	Se rehúsa a contestar

B287. En dólares: ¿Cuánto cuesta comprar una dosis de Metanfetamina/cristal sola en [LCITY]?

__ __ __	dollars
997	No sé
998	Se rehúsa a contestar

B288. En dólares: ¿Cuánto cuesta comprar una dosis de China White en [LCITY]?

__ __ __	dollars
997	No sé
998	Se rehúsa a contestar

B289. En dólares: ¿Cuánto cuesta comprar una dosis de OxyContin, sin receta en [LCITY]?

__ __ __	dollars
997	No sé
998	Se rehúsa a contestar

B290. En dólares: ¿Cuánto cuesta comprar una dosis de Otros opiáceos (medicamentos controlados sin receta (Vicodin, Darvon, Percocet) en [LCITY]?

__ __ __	dollars
997	No sé
998	Se rehúsa a contestar

B291. En dólares: ¿Cuánto cuesta comprar una dosis de Tranquilizantes (Rivotril, Diazepam, Valium, Ativan o Restoril) en [LCITY]?

__ __ __	dollars
997	No sé
998	Se rehúsa a contestar

B292. En dólares: ¿Cuánto cuesta comprar una dosis de Barbitúricos sin receta (Amytal, Nembutal y Seconal) en [LCITY]?

__ __ __	dollars
997	No sé
998	Se rehúsa a contestar

B293. En dólares: ¿Cuánto cuesta comprar una dosis de Fentanilo (Fentanyl) en [LCITY]?

__ __ __	
997	No sé
998	Se rehúsa a contestar

B294. ¿Conoce el costo en dólares de Otras drogas o combinaciones que no se haya mencionado en la lista anterior en [LCITY]?

1	Si
0	No
8	Se rehúsa a contestar

If B294 is not equal to 1, then siga con P instrucción antes de B297.

B295. En dólares: ¿Cuánto cuesta comprar una dosis de Otras drogas o combinaciones que no se haya mencionado en la lista anterior en [LCITY]?

__ __ __	dollars
997	No sé
998	Se rehúsa a contestar

If B295 is greater than 0 and B295 is not equal to 998 then siga con P B296

If not, then siga con P instrucción antes de B297.

B296. Alguna otra droga o combinación de drogas para la que menciono un costo anteriormente.

__ __

If B281 is equal to 1 then siga con P B297

If not, then siga con P B312.

B297. En pesos: ¿Cuánto cuesta comprar una dosis de Heroína en goma sola en [LCITY]?

__ __ __	pesos
997	No sé
998	Se rehúsa a contestar

B298. En pesos: ¿Cuánto cuesta comprar una dosis de Heroína en polvo sola en [LCITY]?

__ __ __	pesos
997	No sé
998	Se rehúsa a contestar

B299. En pesos: ¿Cuánto cuesta comprar una dosis de Crack sola en [LCITY]?

__ __ __	pesos
997	No sé
998	Se rehúsa a contestar

B300. En pesos: ¿Cuánto cuesta comprar una dosis de Cocaína (polvo) sola en [LCITY]?

__ __ __ __	pesos
9997	No sé
9998	Se rehúsa a contestar

B301. En pesos: ¿Cuánto cuesta comprar una dosis de Heroína y cocaína juntas (speedball) en [LCITY]?

__ __ __ __	pesos
9997	No sé
9998	Se rehúsa a contestar

B302. En pesos: ¿Cuánto cuesta comprar una dosis de Metanfetamina/cristal sola en [LCITY]?

__ __ __ __	pesos
9997	No sé
9998	Se rehúsa a contestar

B303. En pesos: ¿Cuánto cuesta comprar una dosis de China White en [LCITY]?

__ __ __ __	pesos
9997	No sé
9998	Se rehúsa a contestar

B304. En pesos: ¿Cuánto cuesta comprar una dosis de OxyContin, sin receta en [LCITY]?

__ __ __ __	pesos
9997	No sé
9998	Se rehúsa a contestar

B305. En pesos: ¿Cuánto cuesta comprar una dosis de Otros opiáceos controlados sin receta (Vicodin, Darvon, Percocet) en [LCITY]?

__ __ __ __	pesos
9997	No sé
9998	Se rehúsa a contestar

B306. En pesos: ¿Cuánto cuesta comprar una dosis de Tranquilizantes (Rivotril, Diazepam, Valium, Ativan o Restoril)) en [LCITY]?

__ __ __ __	pesos
9997	No sé
9998	Se rehúsa a contestar

B307. En pesos: ¿Cuánto cuesta comprar una dosis de Barbitúricos sin receta (Amytal, Nembutal o Seconal) en [LCITY]?

__ __ __ __	pesos
9997	No sé
9998	Se rehúsa a contestar

B308. En pesos: ¿Cuánto cuesta comprar una dosis de Fentanilo (Fentanyl) en [LCITY]?

__ __ __ __	pesos
9997	No sé
9998	Se rehúsa a contestar

B309. ¿Conoce el costo en pesos de Otras drogas o combinaciones que no se haya mencionado en la lista anterior en [LCITY]?

1	Si
0	No
8	Se rehúsa a contestar

If B309 is not equal to 1, then siga con P B312.

B310. En pesos: ¿Cuánto cuesta comprar una dosis de Otras drogas o combinaciones que no se haya mencionado en la lista anterior en [LCITY]?

__ __ __ __	pesos
9997	No sé
9998	Se rehúsa a contestar

If B310 is greater than 0 and B310 is not equal to 998 then siga con P B311

If not, then siga con P B312.

B311. Alguna otra droga o combinación de drogas para la que menciono un costo anteriormente.

__ __

B312. ¿Crees que el precio de la mayoría de drogas de la calle es generalmente más alto o más bajo en Tijuana a comparación de San Diego? (Escoja una)

1	Más alto
2	Más bajo
3	El mismo
7	No sé
8	Se rehúsa a contestar

B313. ¿Cree que el cierre de la frontera México-Estados Unidos afectó el **precio** de las drogas en San Diego?

1	Si
0	No
7	No sé
8	Se rehúsa a contestar

B314. ¿Cree que el cierre de la frontera México-Estados Unidos afectó el **precio** de las drogas en Tijuana?

1 Si

0 No

7 No sé

8 Se rehúsa a contestar

B315. En tu opinión, en los últimos 6 meses ¿el precio de la heroína aumento, disminuyo, o se mantuvo igual en [LCITY]? (Escoja una)

1 Aumentó

2 Dismunuyó

3 Se mantuvo igual

7 No sé

8 Se rehúsa a contestar

B316. En tu opinión, en los últimos 6 meses ¿el precio de metanfetamina aumentó, disminuyó, o se mantuvo igual en [LCITY]? (Escoja una)

1 Aumentó

2 Dismunuyó

3 Se mantuvo igual

7 No sé

8 Se rehúsa a contestar

B317. En tu opinión, en los últimos 6 meses ¿el precio de cocaína en polvo aumento, disminuyo, o se mantuvo igual en [LCITY]? (Escoja una)

1 Aumentó

2 Dismunuyó

3 Se mantuvo igual

7 No sé

8 Se rehúsa a contestar

B318. En tu opinión, en los últimos 6 meses ¿el precio de cocaína crack aumento, disminuyo, o se mantuvo igual en [LCITY]? (Escoja una)

1 Aumentó

2 Dismunuyó

3 Se mantuvo igual

7 No sé

8 Se rehúsa a contestar

B319. En tu opinión, en los últimos 6 meses ¿el precio de Oxycontin aumento, disminuyo, o se mantuvo igual en [LCITY]? (Escoja una)

1 Aumentó

2 Dismunuyó

3 Se mantuvo igual

7 No sé

8 Se rehúsa a contestar

B320. En tu opinión, en los últimos 6 meses ¿el precio de fentanilo (Fentanyl) aumento, disminuyo, o se mantuvo igual en [LCITY]? (Escoja una)

1 Aumentó

2 Dismunuyó

3 Se mantuvo igual

7 No sé

8 Se rehúsa a contestar

B321. En tu opinión, en los últimos 6 meses ¿el precio de China White aumento, disminuyo, o se mantuvo igual en [LCITY]? (Escoja una)

1 Aumentó

2 Dismunuyó

3 Se mantuvo igual

7 No sé

8 Se rehúsa a contestar

B322. En tu opinión, en los últimos 6 meses ¿el precio de otros opiáceos controlados sin receta aumento, disminuyo, o se mantuvo igual en [LCITY]? (Escoja una)

1 Aumentó

2 Dismunuyó

3 Se mantuvo igual

7 No sé

8 Se rehúsa a contestar

LEA: LEA: Hay ocasiones en que la pureza de las drogas en la calle cambia. Ahora le voy hacer unas preguntas sobre los cambios que usted podría haber observado en la pureza de las drogas en los últimos 6 meses.

B323. En tu opinión, en los últimos 6 meses ¿la pureza de heroína aumento, disminuyo, o se mantuvo igual en [LCITY]? (Escoja una)

1 Aumentó

2 Disminuyó

3 Se mantuvo igual

7 No sé

8 Se rehúsa a contestar

B324. En tu opinión, en los últimos 6 meses ¿la pureza de metanfetaminas aumento, disminuyo, o se mantuvo igual en [LCITY]? (Escoja una)

1 Aumentó

2 Disminuyó

3 Se mantuvo igual

7 No sé

8 Se rehúsa a contestar

B325. En tu opinión, en los últimos 6 meses ¿la pureza de cocaína en polvo aumento, disminuyo, o se mantuvo igual en [LCITY]? (Escoja una)

1 Aumentó

2 Disminuyó

3 Se mantuvo igual

7 No sé

8 Se rehúsa a contestar

B326. En tu opinión, en los últimos 6 meses ¿la pureza de cocaína crack aumento, disminuyo, o se mantuvo igual en [LCITY]? (Escoja una)

1 Aumentó

2 Disminuyó

3 Se mantuvo igual

7 No sé

8 Se rehúsa a contestar

B327. En tu opinión, en los últimos 6 meses ¿la pureza de OxyContin aumento, disminuyo, o se mantuvo igual en [LCITY]? (Escoja una)

1 Aumentó

2 Disminuyó

3 Se mantuvo igual

7 No sé

8 Se rehúsa a contestar

B328. En tu opinión, en los últimos 6 meses ¿la pureza de fentanilo (Fentanyl) aumento, disminuyo, o se mantuvo igual en [LCITY]? (Escoja una)

1 Aumentó

2 Disminuyó

3 Se mantuvo igual

7 No sé

8 Se rehúsa a contestar

B329. En tu opinión, en los últimos 6 meses ¿la pureza de China White aumento, disminuyo, o se mantuvo igual en [LCITY]? (Escoja una)

1 Aumentó

2 Disminuyó

3 Se mantuvo igual

7 No sé

8 Se rehúsa a contestar

B330. En tu opinión, en los últimos 6 meses ¿el precio de otros opiáceos controlados sin receta aumento, disminuyo, o se mantuvo igual en [LCITY]? (Escoja una)

1 Aumentó

2 Disminuyó

3 Se mantuvo igual

7 No sé

8 Se rehúsa a contestar

B331. ¿Crees que la pureza de drogas es generalmente mejor o peor en Tijuana a comparación de San Diego? (Escoja una)

1 Mejor

2 Peor

3 Igual

7 No sé

8 Se rehúsa a contestar

B332. ¿Cree que el cierre de la frontera México-Estados Unidos afectó la pureza de las drogas en San Diego?

1 Si

0 No

7 No sé

8 Se rehúsa a contestar

If B332 is not equal to 1, then siga con P B334.

B333. ¿Cree que el cierre de la frontera México-Estados Unidos hace que la pureza de las drogas en San Diego sea mejor o peor? (Escoja una)

1 Mejor

2 Peor

3 Igual

7 No sé

8 Se rehúsa a contestar

B334. ¿Cree que el cierre de la frontera México-Estados Unidos afectó la pureza de las drogas en Tijuana?

1 Si

0 No

7 No sé

8 Se rehúsa a contestar

If B334 is not equal to 1, then siga con P instrucción antes de B336.

B335. ¿Cree que el cierre de la frontera México-Estados Unidos hace que la pureza de las drogas en Tijuana sea mejor o peor? (Escoja una)

1 Mejor
2 Peor
3 Igual
7 No sé
8 Se rehúsa a contestar

LEA: ENTREVISTADOR LEA: Ahora, a veces la disponibilidad de drogas en la calle cambia. Así que te hare unas preguntas sobre los cambios que tu hayas podido observar en la disponibilidad de drogas en los últimos 6 meses. Te voy a preguntar sobre algunas drogas en específico y me gustaría tu opinión aunque no hayas usado una de ellas en específico.

B336. En tu opinión, en los últimos 6 meses, ¿la disponibilidad de heroína ha aumentado, disminuido, o se mantuvo igual en [LCITY]? (Escoja una)

1 Aumentó
2 Disminuyó
3 Se mantuvo igual
7 No sé
8 Se rehúsa a contestar

B337. En tu opinión, en los últimos 6 meses, ¿la disponibilidad de metanfetamina ha aumentado, disminuido, o se mantuvo igual en [LCITY]? (Escoja una)

1 Aumentó
2 Disminuyó
3 Se mantuvo igual
7 No sé
8 Se rehúsa a contestar

B338. En tu opinión, en los últimos 6 meses, ¿la disponibilidad de cocaína en polvo ha aumentado, disminuido, o se mantuvo igual en [LCITY]? (Escoja una)

1	Aumentó
2	Disminuyó
3	Se mantuvo igual
7	No sé
8	Se rehúsa a contestar

B339. En tu opinión, en los últimos 6 meses, ¿la disponibilidad de cocaína crack ha aumentado, disminuido, o se mantuvo igual en [LCITY]? (Escoja una)

1	Aumentó
2	Disminuyó
3	Se mantuvo igual
7	No sé
8	Se rehúsa a contestar

B340. En tu opinión, en los últimos 6 meses, ¿la disponibilidad de OxyContin ha aumentado, disminuido, o se mantuvo igual en [LCITY]? (Escoja una)

1	Aumentó
2	Disminuyó
3	Se mantuvo igual
7	No sé
8	Se rehúsa a contestar

B341. En tu opinión, en los últimos 6 meses, ¿la disponibilidad de fentanilo (Fentanyl) ha aumentado, disminuido, o se mantuvo igual en [LCITY]? (Escoja una)

1 Aumentó

2 Disminuyó

3 Se mantuvo igual

7 No sé

8 Se rehúsa a contestar

B342. En tu opinión, en los últimos 6 meses, ¿la disponibilidad de china white ha aumentado, disminuido, o se mantuvo igual en [LCITY]? (Escoja una)

1 Aumentó

2 Disminuyó

3 Se mantuvo igual

7 No sé

8 Se rehúsa a contestar

B343. En tu opinión, en los últimos 6 meses, ¿la disponibilidad de otros opiáceos (medicamentos controlados sin receta) ha aumentado, disminuido o se mantuvo igual en [LCITY]? (Escoja una)

1 Aumentó

2 Disminuyó

3 Se mantuvo igual

7 No sé

8 Se rehúsa a contestar

B344. ¿Crees que la disponibilidad de drogas en general es mejor o peor en Tijuana a comparación de San Diego? (Escoja una)

1 Mejor

2 Peor

3 La misma

7 No sé

8 Se rehúsa a contestar

B345. ¿Cree que el cierre de la frontera México-Estados Unidos afectó la **disponibilidad** de drogas en San Diego?

1 Si

0 No

7 No sé

8 Se rehúsa a contestar

If B345 is not equal to 1, then siga con P B347.

B346. ¿Cree que el cierre de la frontera México-Estados Unidos hace que la **disponibilidad** de drogas en San Diego sea mejor o peor? (Escoja una)

1 Mejor

2 Peor

3 La misma

7 No sé

8 Se rehúsa a contestar

B347. ¿Cree que el cierre de la frontera México-Estados Unidos afectó la **disponibilidad** de drogas en Tijuana?

1 Si

0 No

7 No sé

8 Se rehúsa a contestar

If B347 is not equal to 1, then siga con P B349.

B348. ¿Cree que el cierre de la frontera México-Estados Unidos hace que la **disponibilidad** de drogas en Tijuana sea mejor o peor? (Escoja una)

1 Mejor

2 Peor

3 La misma

7 No sé

8 Se rehúsa a contestar

B349. ¿Hay drogas que estaban disponibles antes de la pandemia de COVID que ya no se están vendiendo en **[LCITY]**?

1 Si

0 No

7 No sé

8 Se rehúsa a contestar

If B349 is not equal to 1, then siga con P B364.

B350. ¿Hay Heroina en goma/Goma negra (la negra) sola que ya no se está vendiendo en el condado de [LCITY]?

1 Si

0 No

7 No sé

8 Se rehúsa a contestar

B351. ¿Hay Heroína en polvo sola que ya no se está vendiendo en [LCITY]?

1 Si

0 No

7 No sé

8 Se rehúsa a contestar

B352. ¿Hay Cocaína (polvo) sola que ya no se está vendiendo en [LCITY]?

1 Si

0 No

7 No sé

8 Se rehúsa a contestar

B353. ¿Hay Heroína y cocaína juntas (speedball) que ya no se está vendiendo en [LCITY]?

1 Si

0 No

7 No sé

8 Se rehúsa a contestar

B354. ¿Hay Metanfetamina/cristal sola que ya no se está vendiendo en [LCITY]?

1 Si
0 No
7 No sé
8 Se rehúsa a contestar

B355. ¿Hay Crack sola que ya no se está vendiendo en [LCITY]?

1 Si
0 No
7 No sé
8 Se rehúsa a contestar

B356. ¿Hay China White que ya no se está vendiendo en [LCITY]?

1 Si
0 No
7 No sé
8 Se rehúsa a contestar

B357. ¿Hay OxyContin, sin receta que ya no se está vendiendo en [LCITY]?

1 Si
0 No
7 No sé
8 Se rehúsa a contestar

B358. ¿Hay Otros opiáceos controlados sin receta (Vicodin, Darvon, Percocet) que ya no se están vendiendo en [LCITY]?

1 Si
0 No
7 No sé
8 Se rehúsa a contestar

B359. ¿Hay Tranquilizantes (Rivotril, Diazepam, Valium, Ativan o Restoril) que ya no se están vendiendo en [LCITY]?

1 Si

0 No

7 No sé

8 Se rehúsa a contestar

B360. ¿Hay Barbitúricos sin receta (Amytal, Nembutal o Seconal) que ya no se están vendiendo en [LCITY]?

1 Si

0 No

7 No sé

8 Se rehúsa a contestar

B361. ¿Hay Fentanilo que ya no se está vendiendo en el condado de [LCITY]?

1 Si

0 No

7 No sé

8 Se rehúsa a contestar

B362. ¿Hay Otras drogas o combinaciones que no se haya mencionado en la lista anterior que ya no se están vendiendo en [LCITY]?

1 Si

0 No

7 No sé

8 Se rehúsa a contestar

If B362 is equal to 1 then siga con P B363

If not, then siga con P B364.

B363. Por favor especifica el nombre de otra droga o combinación de drogas que no se haya mencionado.

____

B364. ¿Hay algunas drogas o combinación de droga que están disponibles ahora en San Diego, que no estaban disponibles antes de la pandemia de COVID?

1 Si

0 No

7 No sé

8 Se rehúsa a contestar

If B364 is not equal to 1, then siga con P B379.

B365. ¿Hay Heroina en goma/Goma negra (la negra) sola que ahora está disponible en el condado de [LCITY]?

1 Si

0 No

7 No sé

8 Se rehúsa a contestar

B366. ¿Hay Heroína en polvo sola que ahora está disponible en [LCITY]?

1 Si

0 No

7 No sé

8 Se rehúsa a contestar

B367. ¿Hay Cocaína (polvo) sola que ahora está disponible en [LCITY]?

1 Si

0 No

7 No sé

8 Se rehúsa a contestar

B368. ¿Hay Heroína y cocaína juntas (speedball) que ahora están disponible en [LCITY]?

1 Si

0 No

7 No sé

8 Se rehúsa a contestar

B369. ¿Hay Metanfetamina/cristal sola que ahora está disponible en [LCITY]?

1 Si

0 No

7 No sé

8 Se rehúsa a contestar

B370. ¿Hay Crack sola que ahora está disponible en [LCITY]?

1 Si

0 No

7 No sé

8 Se rehúsa a contestar

B371. ¿Hay China White que ahora está disponible en [LCITY]?

1 Si

0 No

7 No sé

8 Se rehúsa a contestar

B372. ¿Hay OxyContin, sin receta que ahora está disponible en [LCITY]?

1 Si

0 No

7 No sé

8 Se rehúsa a contestar

B373. ¿Hay Otros opiáceos controlados sin receta (Vicodin, Darvon, Percocet) que ahora están disponible en [LCITY]?

1 Si

0 No

7 No sé

8 Se rehúsa a contestar

B374. ¿Hay Otros opiáceos (medicamentos controlados sin receta (Vicodin, Darvon, Percocet) que ahora están disponible en [LCITY]?

1 Si

0 No

7 No sé

8 Se rehúsa a contestar

B375. ¿Hay Barbitúricos sin receta (Amytal, Nembutal o Seconal) que ahora están disponible en [LCITY]?

1 Si

0 No

7 No sé

8 Se rehúsa a contestar

B376. ¿Hay Fentanilo (Fentanyl) que ahora está disponible en [LCITY]?

1 Si

0 No

7 No sé

8 Se rehúsa a contestar

B377. ¿Hay Otras drogas o combinaciones que no se haya mencionado en la lista que ahora están disponible en el condado de [LCITY]?

1 Si

0 No

7 No sé

8 Se rehúsa a contestar

If B377 is equal to 1 then siga con P B378

If not, then siga con P B379.

B378. Por favor especifica el nombre de otra droga o combinación de drogas que no se haya mencionado.

____

B379. En comparación con antes de la pandemia de COVID, en los últimos 6 meses, ¿usted ha sido más o menos propenso a inyectarse solo? (Escoja una)

1 Más

2 Menos

3 Igual

4 No aplica

8 Se rehúsa a contestar

Movilidad

If DT_SD is equal to 1 or NDT_TJ is equal to 1 then siga con P B380

If not, then siga con P instrucción antes de B382.

B380. ¿En qué ciudad estuviste la primera vez que te inyectaste drogas? (Escoja una)

1 EUA

2 México

3 Otro

8 Se rehúsa a contestar

If B380 is not equal to 3, then siga con P instrucción antes de B382.

B381. Por favor especifica otro país:

____

If DT_SD is equal to 1 then siga con P instrucción antes de B382

If not, then siga con P instrucción antes de B433.

LEA: LEA: Esta siguiente serie de preguntas le preguntara de sus experiencias en los Estados Unidos.

If Q4 is equal to 1 then siga con P B382

If not, then siga con P instrucción antes de B384.

B382. Me puedes recordar, ¿Cuándo fue la última vez que fuiste a México?
ENTREVISTADOR: Preguntar por un número específico de días, meses, años.

__ __ __ AñOS

__ __ __ MESES

__ __ __ DíAS

998 Se rehúsa a contestar (Meses)

B383. ¿Cuándo fue la vez anterior (antes de la última vez) que Ud. viajó a México?
ENTREVISTADOR: Preguntar por un número específico de días, meses, años.

__ __ __	AñOS
__ __ __	MESES
__ __ __	DíAS
998	Se rehúsa a contestar (Meses)

If Q4 is equal to 2 then siga con P B384

If not, then siga con P instrucción antes de B385.

B384. ¿Cuándo fue la vez anterior (antes de hoy) que Ud. viajó a México? ***ENTREVISTADOR: Preguntar por un número específico de días, meses, años.***

__ __ __	AñOS
__ __ __	MESES
__ __ __	DíAS
998	Se rehúsa a contestar (Meses)

LSTMX = If(Q4 is equal to 2, 0, If(Q4 is equal to 1, B382, 999))

If LSTMX is greater than 6 and LSTMX is not equal to 998 then siga con P B385

If not, then siga con P instrucción antes de B387.

B385. La última vez que estuviste en México, ¿cuál de las siguientes cosas hiciste con personas que viven ahí? **(Entrevistador lea en voz alta la lista, escoja todas las que apliquen)** (Marque todas las que apliquen)

__ Visitar amigos

__ Visitar familia

__ Fui a la farmacia

__ Use drogas (no inyectadas)

__ Me inyecte drogas

__ Compre drogas

__ Vendi drogas

__ Compre jeringas

__ Vendi sexo

__ Compre sexo

__ Tuve sexo con una mujer (no intercambio por sexo)

__ Tuve sexo con un hombre (no intercambio por sexo)

__ Otra cosa

__ No interactúe con alguien de México

__ Se rehúsa a contestar

If B385N is equal to 1 and B385A + B385B + B385C + B385D + B385E + B385F + B385G + B385H + B385I + B385J + B385K + B385L + B385M is greater than 0 then LEA: "Solo puede elegir la respuesta "No interactué con nadie" por sí sola. Por favor revise las respuestas." and siga con P B385.

If B385M is not equal to 1, then siga con P instrucción antes de B387.

B386. Especifique, por favor

____

If LSTMX is less than 6 or LSTMX is equal to 6 then siga con P B387

If not, then siga con P B407.

B387. ¿Cuántas veces fuiste a México en los últimos 6 meses?

__ __ __	veces
998	Se rehúsa a contestar

If B387 is equal to 0 or B387 is equal to 998, then siga con P B407.

B388. ¿A qué ciudades fuiste en México? (Marque todas las que apliquen)

__	Tijuana
__	Rosarito
__	Ensenada
__	Tecate
__	Mexicali
__	Ciudad Juárez
__	Otro
__	Se rehúsa a contestar

If B388G is not equal to 1, then siga con P instrucción antes de B390.

B389. Por favor especifique que otra ciudad en México:

__ __

If B388A + B388B + B388C + B388D + B388E + B388F + B388G is equal to 1 or B388 is equal to 8, then siga con P B392.

B390. ¿A qué ciudad fuiste más seguido en México en los últimos 6 meses? (Escoja una)

1 Tijuana

2 Rosarito

3 Ensenada

4 Tecate

5 Mexicali

6 Ciudad Juárez

7 Otro

8 Se rehúsa a contestar

If B390 is equal to 1 and B388A is not equal to 1 or B390 is equal to 2 and B388B is not equal to 1 or B390 is equal to 3 and B388C is not equal to 1 or B390 is equal to 4 and B388D is not equal to 1 or B390 is equal to 5 and B388E is not equal to 1 or B390 is equal to 6 and B388F is not equal to 1 or B390 is equal to 7 and B388G is not equal to 1 then LEA: "El participante solo puede elegir la ciudad más frecuente de las ciudades mencionadas anteriormente." and siga con P B390.

If B390 is not equal to 7, then siga con P B392.

B391. Por favor especifique que otra ciudad en México:

____

B392. ¿Cuál de las siguientes actividades hiciste cuando fuiste a México en los últimos 6 meses? (entrevistador, leer en voz alta y escoger todas las que apliquen) (Marque todas las que apliquen)

__ Visitar amigos

__ Visitar familia

__ De fiesta

__ Compre drogas

__ Use drogas

__ Fui a un picadero

__ Compre sexo

__ Vendi sexo

__ Fui a un tratamiento para drogas

__ Fui a la farmacia

__ Otro especifica

__ Se rehúsa a contestar

If B392K is not equal to 1, then siga con P instrucción antes de B394.

B393. Por favor, especifica

____

If B392E is equal to 1 then siga con P B394

If not, then siga con P B396.

B394. ¿Por qué vas a México a usar drogas? (Marque todas las que apliquen)

__ Drogas más baratas

__ Mejor calidad de droga/mejor viaje

__ Menos posibilidad de ser molestado por la policía

__ Mas disponibilidad de droga

__ Conozco mejor y me siento más cómodo/a allí

__ Puedo salir de fiesta/trabajadoras sexuales

__ Otro especifica

__ Se rehúsa a contestar

If B394G is not equal to 1, then siga con P B396.

B395. Por favor especifica otras razones

____

B396. ***En los últimos 6 meses,*** ¿compraste alguna de estas cosas en una farmacia en México? (Marque todas las que apliquen)

__ Diazepam

__ Viagra o Cialis

__ Rivotril

__ Fentanilo

__ Jeringas

__ Antibioticos

__ Valium

__ Ativan

__ Restoril

__ Vicodin

__ Amytal

__ Seconal

__ Darvon

__ Percocet

__ Otro

__ Se rehúsa a contestar

__ No corresponde

If B396O is not equal to 1, then siga con P B398.

B397. Por favor, especifica

__ __

B398. En los últimos 6 meses, ¿le pagaste a alguien como a un jalador o a un servicio para obtener drogas o comprar sexo en México?

1 Si

0 No

8 Se rehúsa a contestar

B399. En los últimos 6 meses, ¿compraste jeringa que ya vinieran preparada/llenas con droga en México?

1 Si

0 No

8 Se rehúsa a contestar

If B388A is equal to 1 or B390 is equal to 1 then siga con P B400

If not, then siga con P B404.

B400. En los últimos 6 meses, ¿a qué parte de la ciudad (que colonia) fuiste cuando estuviste en Tijuana? (Entrevistador: Buscar ubicaciones en mapa con indicaciones del participante, escoja todas las que apliquen) (Marque todas las que apliquen)

__ Zona Norte

__ La Mesa

__ El Florido

__ Playas de Tijuana

__ 5 y 10

__ Otay

__ Otro

__ No sé

__ Se rehúsa a contestar

If B400G is not equal to 1, then siga con P instrucción antes de B402.

B401. Por favor especifica en que otra parte de la ciudad/colonia:

____

If B400A + B400B + B400C + B400D + B400E + B400F + B400G is equal to 1 or B400 is equal to 97 or B400 is equal to 98 or B400 is equal to 99, then siga con P B404.

B402. En los últimos 6 meses, ¿a qué parte de la ciudad (que colonia) fuiste ***más seguido*** cuando estuviste en Tijuana? **(Entrevistador: Buscar ubicaciones en mapa con indicaciones del participante)** (Escoja una)

01	Zona Norte
02	La Mesa
03	El Florido
04	Playas de Tijuana
05	5 y 10
06	Otay
07	Otro
97	No sé
98	Se rehúsa a contestar

If B402 is equal to 1 and B400A is not equal to 1 or B402 is equal to 2 and B400B is not equal to 1 or B402 is equal to 3 and B400C is not equal to 1 or B402 is equal to 4 and B400D is not equal to 1 or B402 is equal to 5 and B400E is not equal to 1 or B402 is equal to 6 and B400F is not equal to 1 or B402 is equal to 7 and B400G is not equal to 1 then LEA: "El participante solo pudo elegir la colonia más frecuente de las colonias mencionadas anteriormente." and siga con P B402.

If B402 is not equal to 7, then siga con P B404.

B403. Por favor especifica en que otra parte de la ciudad/colonia:

____

B404. En los últimos 6 meses, cuando fuiste a México, ¿En qué tipo de lugares pasaste tu tiempo? (Entrevistador leer la lista, escoja todas las que apliquen) (Marque todas las que apliquen)

__ Mi c

__ En c

__ Pica

__ Cafe

__ Rest

__ Hote

__ Mote

__ Ubic

__ Bar/

__ Burd

__ Strip

__ Bañ

__ Parq

__ Otro

__ Se r

If B404N is not equal to 1, then siga con P B406.

B405. Por favor especifica otra lugar

____

B406. En promedio, ¿Cuánto tiempo te quedaste en México cuando visitabas en cada ocasión en los últimos 6 meses?

__ __ __	MESES
__ __ __	DíAS
__ __ __	HOURS
998	Se rehúsa a contestar (Días)

B407. Comparado con antes de la pandemia de COVID, en los últimos 6 meses, ¿Qué tan mas o menos seguido viajas a México y usas o compras drogas? (Escoja una)

1	mas
2	menos
3	igual
8	Se rehúsa a contestar

If B387 is equal to 0 or B387 is equal to 998 or LSTMX is greater than 6, then siga con P instrucción antes de B433.

LEA: Ahora me gustaría que pienses sobre tus experiencias con personas de México mientras tu estuviste en México en los últimos 6 meses.

B408. En los últimos 6 meses, ¿cuálesl de las siguientes cosas hiciste en México con personas que viven ahí? **(Entrevistador lea en voz alta la lista, escoja todas la que apliquen)** (Marque todas las que apliquen)

__ Uso drogas (no inyectadas)

__ Se inyecto drogas

__ Compro drogas

__ Vendió drogas

__ Compro jeringas

__ Vendió sexo

__ Compro sexo

__ Tuvo sexo con una mujer (no intercambio por sexo)

__ Tuvo sexo con un hombre (no intercambio por sexo)

__ Otro

__ No interactúe con alguien de México

__ Se rehúsa a contestar

If B408K is equal to 1 and B408A + B408B + B408C + B408D + B408E + B408F + B408G + B408H + B408I + B408J is greater than 0 then LEA: "Solo puede elegir la respuesta "No interactué con nadie" por sí sola. Por favor revise las respuestas." and siga con P B408.

If B408J is not equal to 1, then siga con P instrucción antes de B410.

B409. Por favor especifica la actividad.

__ __

If B408A is not equal to 1, then siga con P instrucción antes de B419.

B410. En los últimos 6 meses cuando estuviste en México, ¿Con cuantas personas diferentes que viven ahí usaste drogas (no inyectadas)?

__ __ # de personas

98 Se rehúsa a contestar

If B410 is equal to 0 or B410 is equal to 98, then siga con P instrucción antes de B419.

LEA: ENTREVISTADOR, LEA AL PARTICIPANTE: La siguientes preguntas se refieren a las personas que viven en México con las que tú has usado drogas no inyectables en los últimos 6 meses en México.

B411. ¿Cuál fue tu relación con ellos? (Marque todas las que apliquen)

__ Familiar

__ Amigo/a

__ Pareja sexual (no por intercambio por sexo)

__ Cliente de intercambio sexual (pareja por pago)

__ Conocido

__ Desconocido

__ Otro

__ Se rehúsa a contestar

If B411G is not equal to 1, then siga con P B413.

B412. Por favor especifica otro.

____

B413. ¿Donde conociste a estas personas? (Marque todas las que apliquen)

__ Su c
__ Pica
__ Café
__ Hote
__ Ubic
__ Bar/
__ Burc
__ Strip
__ Bañ
__ Parc
__ Otro
__ Se r

If B413K is not equal to 1, then siga con P B415.

B414. Por favor especifica otro.

____

B415. ¿Qué tipo de drogas usaste (no inyectada) con ellos? (Marque todas las que apliquen)

__ Heroina en goma/Goma negra (la negra) sola

__ Heroína en polvo sola

__ Cocaina (en polvo) sola

__ Heroina y cocaina juntas (speedball)

__ Metanfetamina/cristal

__ Crack sola

__ China White sola

__ OxyContin, sin receta

__ Otro opioide/sustancia controlada sin receta (Vicodin, Darvon, Percocet)

__ Tranquilizantes (Rivotril, Diazepam, Valium, Ativan o Restoril)

__ Barbituricos sin receta (Amytal, Nembutal, o Seconal)

__ Fentanilo (Fentanyl)

__ Otra droga o combinación de drogas no mencionada anteriormente

__ Se rehúsa a contestar

If B415M is not equal to 1, then siga con P B417.

B416. Por favor especifica otra droga o combinación de drogas:

— —

B417. ¿Les regalaste o prestaste cualquier tipo de equipo de uso de droga, como pipa o popote? (Escoja una)

0 No

1 Si

2 No usamos pipa o popote

8 Se rehúsa a contestar

If B417 is equal to 2, then siga con P instrucción antes de B419.

B418. ¿Usaste una pipa o popote después de cualquiera de ellos?

1 Si

0 No

8 Se rehúsa a contestar

If B408B is not equal to 1, then siga con P B429.

B419. En los últimos 6 meses, cuando estuviste en Mexico ¿con cuantas personas diferentes te inyectaste drogas?

__ __

98 Se rehúsa a contestar

If B419 is equal to 0 or B419 is equal to 98, then siga con P B429.

LEA: LEER: La siguientes preguntas se refieren a las personas que viven en México con las que tú has usado drogas inyectables en los últimos 6 meses en México.

B420. ¿Cuál fue tu relación con ellos?
ENTREVISTADOR: LEA las opciones y escoja todas las que apliquen (Marque todas las que apliquen)

__ Familiar

__ Amigo/a

__ Pareja sexual (no por intercambio por sexo)

__ Cliente de intercambio sexual (pareja por pago)

__ Conocido

__ Desconocido

__ Otro

__ Se rehúsa a contestar

If B420G is not equal to 1, then siga con P B422.

B421. Por favor especifica otro.

____

B422. ¿Donde conociste a estas personas? (Marque todas las que apliquen)

__ Su c[illegible]

__ Pica[illegible]

__ Ubic[illegible]

__ Bar/[illegible]

__ Burd[illegible]

__ Strip[illegible]

__ Bañ[illegible]

__ Parq[illegible]

__ Otro[illegible]

__ Se r[illegible]

If B422I is not equal to 1, then siga con P B424.

B423. Por favor especifica otro.

____

B424. ¿Qué tipo de drogas te inyectaste con ellos? (Marque todas las que apliquen)

__ Heroina en goma/Goma negra (la negra) sola

__ Heroína en polvo sola

__ Cocaina (en polvo) sola

__ Heroina y cocaina juntas (speedball)

__ Metanfetamina/cristal

__ Crack sola

__ China White sola

__ OxyContin, sin receta

__ Otro opioide/sustancia controlada sin receta (Vicodin, Darvon, Percocet)

__ Tranquilizantes (Rivotril, Diazepam, Valium, Ativan o Restoril)

__ Barbituricos sin receta (Amytal, Nembutal, o Seconal)

__ Fentanilo (Fentanyl)

__ Otra droga o combinación de drogas no mencionada anteriormente

__ Se rehúsa a contestar

If B424M is not equal to 1, then siga con P B426.

B425. Por favor especifique otra droga o combinación de drogas.

____

B426. ¿Regalaste o prestaste tu jeringa a cualquiera de estas personas para inyectarse después de que tú la usaras?

1 Si

0 No

8 Se rehúsa a contestar

B427. ¿Te inyectaste con una jeringa que cualquiera de estas personas haya usado antes para inyectarse antes de ti?

1 Si

0 No

8 Se rehúsa a contestar

B428. ¿Usó un cacharro, algodón, o agua de enjuague después o a la misma vez que otras personas con las que estaba?

1 Si

0 No

8 Se rehúsa a contestar

B429. En los **últimos 6 meses, cuando fuiste a México,** ¿a cuántas personas les ha dado drogas, dinero, comida, hospedaje, transportación, alcohol, o algo a cambio de tener relaciones sexuales con usted?

__ __

98 Se rehúsa a contestar

B430. Comparado con antes de la pandemia, en los últimos 6 meses, cuando estuvo en México, ¿ha dado dinero, drogas o alguna otra cosa a cambio de tener relaciones sexuales con usted más seguido o menos seguido que antes? (Escoja una)

1 Mas

2 Menos

3 Igual

4 Nunca

8 Se rehúsa a contestar

B431. En los **últimos 6 meses, cuando fuiste a México,** ¿cuántas personas le dieron a Ud. drogas, dinero, comida, refugio, transporte, alcohol, o cualquier cosa a cambio de sexo?

__ __

98 Se rehúsa a contestar

B432. Comparado con antes de la pandemia, en los últimos 6 meses, cuando estuvo en México, ¿le han dado dinero, drogas o algún otra cosa a cambio de tener relaciones sexuales con usted más seguido o menos seguido que antes? (Escoja una)

1	Mas
2	Menos
3	Igual
4	Nunca
8	Se rehúsa a contestar

USO DE DROGAS en Mexico por TD SD.

If DT_SD is not equal to 1, then siga con P B625.

LEA: LEER: ENTREVISTADOR, LEA AL PARTICIPANTE: Ahora te voy a preguntar algunas preguntas sobre tu uso de drogas en México. Algunas de estas preguntas te van a pedir detalles que pueden ser difíciles de recordar. Por favor haz lo mejor que puedas para dar una repuesta aun si no es el mejor estimado. Recuerda que nosotros no compartiremos tus respuestas con la policía o con cualquier otra persona fuera de este estudio. Si estuviste en la cárcel o prisión en los últimos 6 meses, por favor piensa en el periodo en que estuviste fuera de la cárcel o prisión para responder a las preguntas.

If LSTMX is greater than 6 and LSTMX is not equal to 998, then siga con P B434.

B433. En los últimos 6 meses, en promedio ¿qué tan seguido fumaste o vapeaste marihuana o hachís por sola mientras estabas en México? (Escoja una)

00	Nunca
01	1 vez al mes o menos
02	2-3 días al mes
03	Una vez a la semana
04	2-3 días a la semana
05	4-6 días a la semana
06	Una vez al día
07	2-3 veces al día
08	4 veces o mas por día
98	Se rehúsa a contestar

B434. ¿Alguna vez has usado goma negra/heroína mientras estuviste en México?

1	Si
0	No
8	Se rehúsa a contestar

If B434 is not equal to 1, then siga con P B448.

B435. ¿Alguna vez fumaste, aspiraste, o inhalaste heroína cuando estuviste en México?

1	Si
0	No
8	Se rehúsa a contestar

If B435 is not equal to 1, then siga con P B438.

B436. ¿Cuándo fue la última vez que fumaste, aspiraste o inhalaste heroína en México?

__ __ __	AñOS
__ __ __	MESES
__ __ __	DíAS
998	Se rehúsa a contestar (Meses)
999	No corresponde (Meses)

If B436 is less than LSTMX then LEA: "Esa respuesta es menor que la última vez que el participante fue a México. Por favor verificar respuesta." and siga con P B436.

If B436 is equal to 998 or B436 is equal to 999 or B436 is greater than 6, then siga con P B438.

B437. En los últimos 6 meses, en promedio ¿qué tan seguido fumaste, aspiraste o inhalaste heroína mientras estabas en México? (Escoja una)

00	Nunca
01	1 vez al mes o menos
02	2-3 días al mes
03	Una vez a la semana
04	2-3 días a la semana
05	4-6 días a la semana
06	Una vez al día
07	2-3 veces al día
08	4 veces o mas por día
98	Se rehúsa a contestar

B438. ¿Alguna vez te has inyectado heroína (sola) cuando estuviste en México?

1	Si
0	No
8	Se rehúsa a contestar

If B438 is not equal to 1, then siga con P instrucción antes de B441.

B439. ¿Cuándo fue la última vez que te inyectaste heroína sola en México?

__ __ __	AñOS
__ __ __	MESES
__ __ __	DíAS
998	Se rehúsa a contestar (Meses)
999	No corresponde (Meses)

If B439 is less than LSTMX then LEA: "Esa respuesta es menor que la última vez que el participante fue a México. Por favor verificar respuesta." and siga con P B439.

If B439 is equal to 998 or B439 is equal to 999 or B439 is greater than 6, then siga con P instrucción antes de B441.

B440. En los últimos 6 meses, en promedio ¿Qué tan seguido te inyectaste heroína sola cuando estuviste en México? (Escoja una)

00	Nunca
01	1 vez al mes o menos
02	2-3 días al mes
03	Una vez a la semana
04	2-3 días a la semana
05	4-6 días a la semana
06	Una vez al día
07	2-3 veces al día
08	4 veces o mas por día
98	Se rehúsa a contestar

If (B436 is equal to 998 or B436 is equal to 999 or B436 is greater than 6) and (B439 is equal to 998 or B439 is equal to 999 or B439 is greater than 6), then siga con P B448.

B441. En los últimos 6 meses, cuando usaste heroína en México, ¿de qué color era? **Leer en voz alta la lista. Escoja todas las que apliquen.** (Marque todas las que apliquen)

__	Goma negra
__	Polvo café
__	Polvo blanco
__	Polvo amarillo
__	Polvo naranja
__	Otro
__	Se rehúsa a contestar
__	No corresponde

If B441F is not equal to 1, then siga con P instrucción antes de B443.

B442. Por favor especifica el color de heroína que usaste.

____

If B439 is equal to 998 or B439 is equal to 999 or B439 is greater than 6, then siga con P B448.

B443. En los últimos 6 meses, cuando te inyectaste heroína en México, ¿de qué color fue **más seguido**? (Escoja una)

1	Goma negra
2	Polvo café
3	Polvo blanco
4	Polvo amarillo
5	Polvo naranja
6	Otro
8	Se rehúsa a contestar
9	No corresponde

If B443 is not equal to 6, then siga con P instrucción antes de B445.

B444. Por favor especificar el color de heroína que te inyectaste más seguido.

____

If B443 is not equal to 4, then siga con P instrucción antes de B446.

B445. En los últimos 6 meses, cuando te inyectaste heroína en Mexico, ¿Qué tan seguido fue color ***amarillo***? (Escoja una)

1 Nunca

2 Menos de la mitad del tiempo

3 Casi la mitad del tiempo

4 Más de la mitad del tiempo

5 Siempre

8 Se rehúsa a contestar

If B443 is not equal to 5, then siga con P instrucción antes de B447.

B446. En los últimos 6 meses, cuando te inyectaste heroína en México, ¿Qué tan seguido fue color ***naranja***? (Escoja una)

1 Nunca

2 Menos de la mitad del tiempo

3 Casi la mitad del tiempo

4 Más de la mitad del tiempo

5 Siempre

8 Se rehúsa a contestar

If B443 is not equal to 6, then siga con P B448.

B447. En los últimos 6 meses, cuando te inyectaste heroína en México, ¿Qué tan seguido fue [Respuesta a P B444]? (Escoja una)

1 Nunca

2 Menos de la mitad del tiempo

3 Casi la mitad del tiempo

4 Más de la mitad del tiempo

5 Siempre

8 Se rehúsa a contestar

B448. ¿Alguna vez usaste cocaína o crack cuando estuviste en México?

1 Si

0 No

8 Se rehúsa a contestar

If B448 is not equal to 1, then siga con P B458.

B449. ¿Alguna vez has fumado, aspirado o inhalado crack o cocaína en polvo cuando estuviste en México?

1 Si

0 No

8 Se rehúsa a contestar

If B449 is not equal to 1, then siga con P B452.

B450. ¿Cuándo fue la última vez que fumaste, aspiraste o inhalaste crack o cocaína en polvo cuando estuviste en México?

__ __ __	AñOS
__ __ __	MESES
__ __ __	DíAS
998	Se rehúsa a contestar (Meses)
999	No corresponde (Meses)

If B450 is less than LSTMX then LEA: "Esa respuesta es menor que la última vez que el participante fue a México. Por favor verificar respuesta." and siga con P B450.

If B450 is equal to 998 or B450 is equal to 999 or B450 is greater than 6, then siga con P B452.

B451. En los últimos 6 meses, en promedio ¿Qué tan seguido fumaste, aspiraste o inhalaste crack o cocaína en polvo cuando estabas en México? (Escoja una)

00	Nunca
01	1 vez al mes o menos
02	2-3 días al mes
03	Una vez a la semana
04	2-3 días a la semana
05	4-6 días a la semana
06	Una vez al día
07	2-3 veces al día
08	4 veces o mas por día
98	Se rehúsa a contestar

B452. ¿Alguna vez te has inyectado cocaína sola cuando estabas en México?

1	Si
0	No
8	Se rehúsa a contestar

If B452 is not equal to 1, then siga con P instrucción antes de B455.

B453. ¿Cuándo fue la última vez que te inyectaste cocaína sola cuando estuviste en México?

__ __ __	AñOS
__ __ __	MESES
__ __ __	DíAS
998	Se rehúsa a contestar (Meses)
999	No corresponde (Meses)

If B453 is less than LSTMX then LEA: "Esa respuesta es menor que la última vez que el participante fue a México. Por favor verificar respuesta." and siga con P B453.

If B453 is equal to 998 or B453 is equal to 999 or B453 is greater than 6, then siga con P instrucción antes de B455.

B454. En los últimos 6 meses, en promedio ¿Qué tan seguido te inyectaste cocaína sola cuando estuviste en México? (Escoja una)

00	Nunca
01	1 vez al mes o menos
02	2-3 días al mes
03	Una vez a la semana
04	2-3 días a la semana
05	4-6 días a la semana
06	Una vez al día
07	2-3 veces al día
08	4 veces o mas por día
98	Se rehúsa a contestar

If B434 is not equal to 1, then siga con P B458.

B455. ¿Alguna vez te has inyectado heroína y cocaína juntas (speedball) cuando estuviste en México?

1	Si
0	No
8	Se rehúsa a contestar

If B455 is not equal to 1, then siga con P B458.

B456. ¿Cuándo fue la última vez que te inyectaste heroína y cocaína juntas (speedball) cuando estuviste en México?

__ __ __	AñOS
__ __ __	MESES
__ __ __	DíAS
998	Se rehúsa a contestar (Meses)
999	No corresponde (Meses)

If B456 is less than LSTMX then LEA: "Esa respuesta es menor que la última vez que el participante fue a México. Por favor verificar respuesta." and siga con P B456.

If B456 is equal to 998 or B456 is equal to 999 or B456 is greater than 6, then siga con P B458.

B457. En los últimos 6 meses, en promedio ¿Qué tan seguido te inyectaste heroína y cocaína juntas (speedball) cuando estuviste en México? (Escoja una)

00	Nunca
01	1 vez al mes o menos
02	2-3 días al mes
03	Una vez a la semana
04	2-3 días a la semana
05	4-6 días a la semana
06	Una vez al día
07	2-3 veces al día
08	4 veces o mas por día
98	Se rehúsa a contestar

B458. ¿Alguna vez has usado metanfetamina/cristal cuando estuviste en México?

1	Si
0	No
8	Se rehúsa a contestar

If B458 is not equal to 1, then siga con P B475.

B459. ¿Alguna vez has fumado, aspirado, inhalado o vapeado metanfetamina/cristal sola cuando estuviste en México?

1	Si
0	No
8	Se rehúsa a contestar

If B459 is not equal to 1, then siga con P B462.

B460. ¿Cuándo fue la última vez que fumaste, aspiraste, o inhalaste metanfetaminas/cristal sola cuando estuviste en México?

__ __ __	AñOS
__ __ __	MESES
__ __ __	DíAS
998	Se rehúsa a contestar (Meses)
999	No corresponde (Meses)

If B460 is less than LSTMX then LEA: "Esa respuesta es menor que la última vez que el participante fue a México. Por favor verificar respuesta." and siga con P B460.

If B460 is equal to 998 or B460 is equal to 999 or B460 is greater than 6, then siga con P B462.

B461. En los últimos 6 meses, en promedio ¿Qué tan seguido fumaste, aspiraste, inhalaste o vapeaste metanfetaminas/cristal cuando estuviste en México? (Escoja una)

00	Nunca
01	1 vez al mes o menos
02	2-3 días al mes
03	Una vez a la semana
04	2-3 días a la semana
05	4-6 días a la semana
06	Una vez al día
07	2-3 veces al día
08	4 veces o mas por día
98	Se rehúsa a contestar

B462. ¿Alguna vez te has inyectado metanfetaminas/cristal sola cuando estuviste en México?

1	Si
0	No
8	Se rehúsa a contestar

If B462 is not equal to 1, then siga con P instrucción antes de B465.

B463. ¿Cuándo fue la última vez que te inyectaste metanfetaminas/cristal sola cuando estuviste en México?

__ __ __	AñOS
__ __ __	MESES
__ __ __	DíAS
998	Se rehúsa a contestar (Meses)
999	No corresponde (Meses)

If B463 is less than LSTMX then LEA: "Esa respuesta es menor que la última vez que el participante fue a México. Por favor verificar respuesta." and siga con P B463.

If B463 is equal to 998 or B463 is equal to 999 or B463 is greater than 6, then siga con P instrucción antes de B465.

B464. En los últimos 6 meses, en promedio ¿Qué tan seguido te inyectaste metanfetaminas/cristal solo cuando estuviste en México? (Escoja una)

00	Nunca
01	1 vez al mes o menos
02	2-3 días al mes
03	Una vez a la semana
04	2-3 días a la semana
05	4-6 días a la semana
06	Una vez al día
07	2-3 veces al día
08	4 veces o mas por día
98	Se rehúsa a contestar

If B448 is not equal to 1, then siga con P instrucción antes de B468.

B465. ¿Alguna vez te inyectaste metanfetaminas/cristal y cocaína juntas cuando estuviste en México?

1	Si
0	No
8	Se rehúsa a contestar

If B465 is not equal to 1, then siga con P instrucción antes de B468.

B466. ¿Cuándo fue la última vez que te inyectaste metanfetaminas/cristal y cocaína juntas cuando estuviste en México?

__ __ __	AñOS
__ __ __	MESES
__ __ __	DíAS
998	Se rehúsa a contestar (Meses)
999	No corresponde (Meses)

If B466 is less than LSTMX then LEA: "Esa respuesta es menor que la última vez que el participante fue a México. Por favor verificar respuesta." and siga con P B466.

If B466 is equal to 998 or B466 is equal to 999 or B466 is greater than 6, then siga con P instrucción antes de B468.

B467. En los últimos 6 meses, en promedio, ¿qué tan seguido te inyectaste metanfetaminas/cristal y cocaína juntas cuando estuviste en México? (Escoja una)

00	Nunca
01	1 vez al mes o menos
02	2-3 días al mes
03	Una vez a la semana
04	2-3 días a la semana
05	4-6 días a la semana
06	Una vez al día
07	2-3 veces al día
08	4 veces o mas por día
98	Se rehúsa a contestar

If B434 is not equal to 1, then siga con P instrucción antes de B471.

B468. ¿Alguna vez te has inyectado metanfetaminas/cristal y heroína juntas cuando estuviste en México?

1	Si
0	No
8	Se rehúsa a contestar

If B468 is not equal to 1, then siga con P instrucción antes de B471.

B469. ¿Cuándo fue la última vez que te inyectaste metanfetaminas/cristal y heroína juntas cuando estuviste en México?

__ __ __	AñOS
__ __ __	MESES
__ __ __	DíAS
998	Se rehúsa a contestar (Meses)
999	No corresponde (Meses)

If B469 is less than LSTMX then LEA: "Esa respuesta es menor que la última vez que el participante fue a México. Por favor verificar respuesta." and siga con P B469.

If B469 is equal to 998 or B469 is equal to 999 or B469 is greater than 6, then siga con P instrucción antes de B471.

B470. En los últimos 6 meses, en promedio, ¿qué tan seguido te inyectaste metanfetaminas/cristal y heroína juntas cuando estuviste en México? (Escoja una)

00	Nunca
01	1 vez al mes o menos
02	2-3 días al mes
03	Una vez a la semana
04	2-3 días a la semana
05	4-6 días a la semana
06	Una vez al día
07	2-3 veces al día
08	4 veces o mas por día
98	Se rehúsa a contestar

If (B460 is equal to 998 or B460 is equal to 999 or B460 is greater than 6) and (B463 is equal to 998 or B463 is equal to 999 or B463 is greater than 6) and (B466 is equal to 998 or B466 is equal to 999 or B466 is greater than 6) and (B469 is equal to 998 or B469 is equal to 999 or B469 is greater than 6), then siga con P B475.

B471. En los últimos 6 meses, cuando usaste metanfetamina en México, ¿de qué color era? (Marque todas las que apliquen)

__ Blanco

__ Rosa

__ Verde

__ Negro

__ Amarillo

__ Hielo (transparente)

__ Otro

__ Se rehúsa a contestar

__ No corresponde

If B471G is not equal to 1, then siga con P instrucción antes de B473.

B472. Por favor especifica el color de metanfetaminas mientras estuviste en Mexico:

__ __

If (B463 is equal to 998 or B463 is equal to 999 or B463 is greater than 6) and (B466 is equal to 998 or B466 is equal to 999 or B466 is greater than 6) and (B469 is equal to 998 or B469 is equal to 999 or B469 is greater than 6), then siga con P B475.

B473. En los últimos 6 meses, cuando te inyectaste metanfetamina en México, ¿de qué color fue más seguido? (Escoja una)

1	Blanco
2	Rosa
3	Verde
4	Negro
5	Amarillo
6	Hielo (transparente)
7	Otro
8	Se rehúsa a contestar
9	No corresponde

If B473 is not equal to 7, then siga con P B475.

B474. Por favor especifica el color de metanfetaminas.

____

B475. ¿Alguna vez has usado China White cuando estuviste en México?

1	Si
0	No
8	Se rehúsa a contestar

If B475 is not equal to 1, then siga con P B485.

B476. ¿Alguna vez has fumado, aspirado o inhalado China White cuando estuviste en México?

1	Si
0	No
8	Se rehúsa a contestar

If B476 is not equal to 1, then siga con P B479.

B477. ¿Cuándo fue la última vez que fumaste, aspiraste o inhalaste China White en México?

__ __ __	AñOS
__ __ __	MESES
__ __ __	DíAS
998	Se rehúsa a contestar (Meses)
999	No corresponde (Meses)

If B477 is less than LSTMX then LEA: "Esa respuesta es menor que la última vez que el participante fue a México. Por favor verificar respuesta." and siga con P B477.

If B477 is equal to 998 or B477 is equal to 999 or B477 is greater than 6, then siga con P B479.

B478. En los últimos 6 meses, que tan seguido fumaste, aspiraste o inhalaste China White cuando estuviste en México? (Escoja una)

00	Nunca
01	1 vez al mes o menos
02	2-3 días al mes
03	Una vez a la semana
04	2-3 días a la semana
05	4-6 días a la semana
06	Una vez al día
07	2-3 veces al día
08	4 veces o mas por día
98	Se rehúsa a contestar

B479. ¿Alguna vez te has inyectado China White sola en México?

1	Si
0	No
8	Se rehúsa a contestar

If B479 is not equal to 1, then siga con P instrucción antes de B482.

B480. ¿Cuándo fue la última vez que te inyectaste China White en México?

__ __ __	AñOS
__ __ __	MESES
__ __ __	DíAS
998	Se rehúsa a contestar (Meses)
999	No corresponde (Meses)

If B480 is less than LSTMX then LEA: "Esa respuesta es menor que la última vez que el participante fue a México. Por favor verificar respuesta." and siga con P B480.

If B480 is equal to 998 or B480 is equal to 999 or B480 is greater than 6, then siga con P instrucción antes de B482.

B481. En los últimos 6 meses, en promedio, ¿qué tan seguido te inyectaste China White cuando estuviste en México? (Escoja una)

00	Nunca
01	1 vez al mes o menos
02	2-3 días al mes
03	Una vez a la semana
04	2-3 días a la semana
05	4-6 días a la semana
06	Una vez al día
07	2-3 veces al día
08	4 veces o mas por día
98	Se rehúsa a contestar

If B458 is not equal to 1, then siga con P B485.

B482. ¿Alguna vez te has inyectado China White y metanfetaminas/cristal juntas en México?

1 Si

0 No

8 Se rehúsa a contestar

If B482 is not equal to 1, then siga con P B485.

B483. ¿Cuándo fue la última vez que te inyectaste China White y metanfetaminas/cristal juntas cuando estuviste en México?

__ __ __ AñOS

__ __ __ MESES

__ __ __ DíAS

998 Se rehúsa a contestar (Meses)

999 No corresponde (Meses)

If B483 is less than LSTMX then LEA: "Esa respuesta es menor que la última vez que el participante fue a México. Por favor verificar respuesta." and siga con P B483.

If B483 is equal to 998 or B483 is equal to 999 or B483 is greater than 6, then siga con P B485.

B484. En los últimos 6 meses, ¿cuándo usaste China White y metanfetaminas/cristal juntas en México, en promedio que tan seguido te inyectaste las dos juntas? (Escoja una)

00	Nunca
01	1 vez al mes o menos
02	2-3 días al mes
03	Una vez a la semana
04	2-3 días a la semana
05	4-6 días a la semana
06	Una vez al día
07	2-3 veces al día
08	4 veces o mas por día
98	Se rehúsa a contestar

B485. ¿Alguna vez has usado fentanilo (Fentanyl) en México?

1	Si
0	No
8	Se rehúsa a contestar

If B485 is not equal to 1, then siga con P B492.

B486. ¿Cuándo fue la última vez que usaste fentanilo (Fentanyl) en México?

__ __ __	AñOS
__ __ __	MESES
__ __ __	DíAS
998	Se rehúsa a contestar (Meses)
999	No corresponde (Meses)

If B486 is less than LSTMX then LEA: "Esa respuesta es menor que la última vez que el participante fue a México. Por favor verificar respuesta." and siga con P B486.

If B486 is equal to 998 or B486 is equal to 999 or B486 is greater than 6, then siga con P B492.

B487. En los últimos 6 meses, ¿fumaste, inhalaste, aspiraste o vapeaste fentanilo en México?

1	Si
0	No
8	Se rehúsa a contestar

If B487 is not equal to 1, then siga con P B489.

B488. Durante los últimos 6 meses, en promedio que tan seguido fumaste, inhalaste, o aspiraste fentanilo (Fentanyl) cuando estuviste en México? (Escoja una)

00	Nunca
01	1 vez al mes o menos
02	2-3 días al mes
03	Una vez a la semana
04	2-3 días a la semana
05	4-6 días a la semana
06	Una vez al día
07	2-3 veces al día
08	4 veces o mas por día
98	Se rehúsa a contestar

B489. ¿En los últimos 6 meses, te has inyectado fentanilo en México?

1	Si
0	No
8	Se rehúsa a contestar

If B489 is not equal to 1, then siga con P instrucción antes de B491.

B490. Durante los últimos 6 meses cuando estuviste en México, en promedio ¿Qué tan seguido te inyectaste fentanilo (fentanyl) solo? (Escoja una)

00 Nunca

01 1 vez al mes o menos

02 2-3 días al mes

03 Una vez a la semana

04 2-3 días a la semana

05 4-6 días a la semana

06 Una vez al día

07 2-3 veces al día

08 4 veces o mas por día

98 Se rehúsa a contestar

If B458 is not equal to 1, then siga con P B492.

B491. En los últimos 6 meses, cuando estuviste en Mexico, en promedio, ¿Qué tan seguido te inyectaste fentanilo y metanfetaminas/cristal juntos? (Escoja una)

00 Nunca

01 1 vez al mes o menos

02 2-3 días al mes

03 Una vez a la semana

04 2-3 días a la semana

05 4-6 días a la semana

06 Una vez al día

07 2-3 veces al día

08 4 veces o mas por día

98 Se rehúsa a contestar

B492. ¿Alguna vez has usado PCP/Polvo de Ángel cuando estabas en México?

1	Si
0	No
8	Se rehúsa a contestar

If B492 is not equal to 1, then siga con P B495.

B493. ¿Cuándo fue la última vez que fumaste, aspiraste o inhalaste PCP/Polvo de Ángel en México?

__ __ __	AñOS
__ __ __	MESES
__ __ __	DíAS
998	Se rehúsa a contestar (Meses)
999	No corresponde (Meses)

If B493 is less than LSTMX then LEA: "Esa respuesta es menor que la última vez que el participante fue a México. Por favor verificar respuesta." and siga con P B493.

If B493 is equal to 998 or B493 is equal to 999 or B493 is greater than 6, then siga con P B495.

B494. En los últimos 6 meses, cuando usaste PCP en México, en promedio, que tan seguido lo fumaste, inhalaste o vapeaste? (Escoja una)

00	Nunca
01	1 vez al mes o menos
02	2-3 días al mes
03	Una vez a la semana
04	2-3 días a la semana
05	4-6 días a la semana
06	Una vez al día
07	2-3 veces al día
08	4 veces o mas por día
98	Se rehúsa a contestar

B495. ¿Alguna vez has usado éxtasis cuando estuviste en México?

1	Si
0	No
8	Se rehúsa a contestar

If B495 is not equal to 1, then siga con P B501.

B496. ¿Cuándo fue la última vez que usaste éxtasis en México?

__ __ __	AñOS
__ __ __	MESES
__ __ __	DíAS
998	Se rehúsa a contestar (Meses)
999	No corresponde (Meses)

If B496 is less than LSTMX then LEA: "Esa respuesta es menor que la última vez que el participante fue a México. Por favor verificar respuesta." and siga con P B496.

If B496 is equal to 998 or B496 is equal to 999 or B496 is greater than 6, then siga con P B501.

B497. En los últimos 6 meses cuando usaste éxtasis en México, ¿Cómo los usaste?
Entrevistador: Si el participante indica que no usó éxtasis **en México en los últimos 6 meses, marque No corresponde.**
(Marque todas las que apliquen)

__ Inyectado

__ Fumado (chasing)

__ Aspirado

__ Inhalado

__ Tomado

__ Rectal (bumping)

__ Otro

__ No corresponde

__ Se rehúsa a contestar

If B497 is greater than 1 and B497H is equal to 1 then LEA: "Solo puedes escoger la opcion "No corresponde" por si sola, verificar respuesta." and siga con P B497.

If B497G is not equal to 1, then siga con P instrucción antes de B499.

B498. Especifica de que otra manera has usado éxtasis en los últimos 6 meses en México.

____

If B497A is not equal to 1, then siga con P instrucción antes de B500.

B499. En los últimos 6 meses, cuando usaste éxtasis en México, en promedio, ¿qué tan seguido te lo inyectaste? (Escoja una)

00	Nunca
01	1 vez al mes o menos
02	2-3 días al mes
03	Una vez a la semana
04	2-3 días a la semana
05	4-6 días a la semana
06	Una vez al día
07	2-3 veces al día
08	4 veces o mas por día
98	Se rehúsa a contestar

If B497H is equal to 1 or B497 is equal to 98, then siga con P B501.

B500. En los últimos 6 meses, cuando usaste éxtasis en México, en promedio, ¿qué tan seguido lo usaste de otra manera (no inyectada)? (Escoja una)

00	Nunca
01	1 vez al mes o menos
02	2-3 días al mes
03	Una vez a la semana
04	2-3 días a la semana
05	4-6 días a la semana
06	Una vez al día
07	2-3 veces al día
08	4 veces o mas por día
98	Se rehúsa a contestar

B501. ¿Alguna vez ha usado Ketamine (Special K, K) en México?

1	Si
0	No
8	Se rehúsa a contestar

If B501 is not equal to 1, then siga con P B507.

B502. ¿Cuándo fue la última vez que usaste Ketamine (Special K, K) en México?

__ __ __	AñOS
__ __ __	MESES
__ __ __	DíAS
998	Se rehúsa a contestar (Meses)
999	No corresponde (Meses)

If B502 is less than LSTMX then LEA: "Esa respuesta es menor que la última vez que el participante fue a México. Por favor verificar respuesta." and siga con P B502.

If B502 is equal to 998 or B502 is equal to 999 or B502 is greater than 6, then siga con P B507.

B503. Cuando usaste Ketamine (Special K, K) en los últimos 6 meses en México, ¿Cómo los usaste?
Entrevistador: Si el participante indica que no usó Ketamine **en México en los últimos 6 meses, marque No corresponde.**
(Marque todas las que apliquen)

__ Inyectado

__ Fumado (chasing)

__ Aspirado

__ Inhalado

__ Tomado

__ Rectal (bumping)

__ Otro

__ No corresponde

__ Se rehúsa a contestar

If B503 is greater than 1 and B503H is equal to 1 then LEA: "Solo puedes escoger la opcion "No corresponde" por si sola, verificar respuesta." and siga con P B503.

If B503G is not equal to 1, then siga con P instrucción antes de B505.

B504. Especifica de que otra manera usaste Ketamine en los últimos 6 meses en México.

____

If B503A is not equal to 1, then siga con P instrucción antes de B506.

B505. Durante los últimos 6 meses cuando usaste Ketamine (Special K, K) en México, en promedio, ¿Qué tan seguido te la inyectaste? (Escoja una)

00	Nunca
01	1 vez al mes o menos
02	2-3 días al mes
03	Una vez a la semana
04	2-3 días a la semana
05	4-6 días a la semana
06	Una vez al día
07	2-3 veces al día
08	4 veces o mas por día
98	Se rehúsa a contestar

If B503H is equal to 1 or B503 is equal to 98, then siga con P B507.

B506. Durante los últimos 6 meses cuando usaste Ketamine (Special K, K) en México, en promedio, ¿Qué tan seguido la usaste de otras maneras (no inyectadas)? (Escoja una)

00	Nunca
01	1 vez al mes o menos
02	2-3 días al mes
03	Una vez a la semana
04	2-3 días a la semana
05	4-6 días a la semana
06	Una vez al día
07	2-3 veces al día
08	4 veces o mas por día
98	Se rehúsa a contestar

B507. ¿Alguna vez has usado OxyContin en México?

1	Si
0	No
8	Se rehúsa a contestar

If B507 is not equal to 1, then siga con P B513.

B508. ¿Cuándo fue la última vez que usaste OxyContin en México?

__ __ __	AñOS
__ __ __	MESES
__ __ __	DíAS
998	Se rehúsa a contestar (Meses)
999	No corresponde (Meses)

If B508 is less than LSTMX then LEA: "Esa respuesta es menor que la última vez que el participante fue a México. Por favor verificar respuesta." and siga con P B508.

If B508 is equal to 998 or B508 is equal to 999 or B508 is greater than 6, then siga con P B513.

B509. En los últimos 6 meses, cuando usaste oxycontin en México ¿Cómo los usaste?
Entrevistador: Si el participante indica que no usó oxycontin **en México en los últimos 6 meses, marque No corresponde.**
(Marque todas las que apliquen)

__ Inyectado

__ Fumado (chasing)

__ Aspirado

__ Inhalado

__ Tomado

__ Rectal (bumping)

__ Otro

__ No corresponde

__ Se rehúsa a contestar

If B509 is greater than 1 and B509H is equal to 1 then LEA: "Solo puedes escoger la opcion "No corresponde" por si sola, verificar respuesta." and siga con P B509.

If B509G is not equal to 1, then siga con P instrucción antes de B511.

B510. Especifica de que otra manera usaste Oxycontin en los últimos 6 meses en México.

____

If B509A is not equal to 1, then siga con P instrucción antes de B512.

B511. En los últimos 6 meses cuando usaste oxycontin en México, en promedio, ¿Qué tan seguido te lo inyectaste? (Escoja una)

00	Nunca
01	1 vez al mes o menos
02	2-3 días al mes
03	Una vez a la semana
04	2-3 días a la semana
05	4-6 días a la semana
06	Una vez al día
07	2-3 veces al día
08	4 veces o mas por día
98	Se rehúsa a contestar

If B509H is equal to 1 or B509 is equal to 98, then siga con P B513.

B512. En los últimos 6 meses cuando usaste oxycontin en México, en promedio, ¿Qué tan seguido lo usaste de otras maneras (no inyectado)? (Escoja una)

00	Nunca
01	1 vez al mes o menos
02	2-3 días al mes
03	Una vez a la semana
04	2-3 días a la semana
05	4-6 días a la semana
06	Una vez al día
07	2-3 veces al día
08	4 veces o mas por día
98	Se rehúsa a contestar

B513. ¿Alguna vez has usado opioides de receta (Vicodin, Darvon, Percocet) cuando estuviste en México?

1 Si

0 No

8 Se rehúsa a contestar

If B513 is not equal to 1, then siga con P B519.

B514. ¿Cuándo fue la última vez que tomaste opioides de receta (Vicodin, Darvon, Percocet) en México?

__ __ __ AñOS

__ __ __ MESES

__ __ __ DíAS

998 Se rehúsa a contestar (Meses)

999 No corresponde (Meses)

If B514 is less than LSTMX then LEA: "Esa respuesta es menor que la última vez que el participante fue a México. Por favor verificar respuesta." and siga con P B514.

If B514 is equal to 998 or B514 is equal to 999 or B514 is greater than 6, then siga con P B519.

B515. En los últimos 6 meses, cuando usaste opioides de receta (Vicodin, Darvon, Percocet) en México, ¿Cómo los usaste?
Entrevistador: Si el participante indica que no usó opioides de receta **en México en los últimos 6 meses, marque No corresponde.**
(Marque todas las que apliquen)

__ Inyectado

__ Fumado (chasing)

__ Aspirado

__ Inhalado

__ Tomado

__ Rectal (bumping)

__ Otro

__ No corresponde

__ Se rehúsa a contestar

If B515 is greater than 1 and B515H is equal to 1 then LEA: "Solo puedes escoger la opcion "No corresponde" por si sola, verificar respuesta." and siga con P B515.

If B515G is not equal to 1, then siga con P instrucción antes de B517.

B516. Especifica de que otra manera has usado opioides de receta (Vicodin, Darvon, Percocet) en los últimos 6 meses en México.

__ __

If B515A is not equal to 1, then siga con P instrucción antes de B518.

B517. En los últimos 6 meses cuando usaste opioides de receta (Vicodin, Darvon, Percocet) en México, en promedio, ¿Qué tan seguido te los inyectaste? (Escoja una)

00	Nunca
01	1 vez al mes o menos
02	2-3 días al mes
03	Una vez a la semana
04	2-3 días a la semana
05	4-6 días a la semana
06	Una vez al día
07	2-3 veces al día
08	4 veces o mas por día
98	Se rehúsa a contestar

If B515H is equal to 1 or B515 is equal to 98, then siga con P B519.

B518. En los últimos 6 meses cuando usaste opioides de receta (Vicodin, Darvon, Percocet) en México, en promedio, ¿Qué tan seguido lo usaste de otra manera (no inyectado)? (Escoja una)

00	Nunca
01	1 vez al mes o menos
02	2-3 días al mes
03	Una vez a la semana
04	2-3 días a la semana
05	4-6 días a la semana
06	Una vez al día
07	2-3 veces al día
08	4 veces o mas por día
98	Se rehúsa a contestar

B519. ¿Alguna vez has usado drogas alucinógenas como ácido, LSD, peyote, mezcalina, o hongos en México?

1	Si
0	No
8	Se rehúsa a contestar

If B519 is not equal to 1, then siga con P B522.

B520. ¿Cuándo fue la última vez que usaste drogas alucinógenas como ácido, LSD, peyote, mezcalina, o hongos en México?

__ __ __	AñOS
__ __ __	MESES
__ __ __	DíAS
998	Se rehúsa a contestar (Meses)
999	No corresponde (Meses)

If B520 is less than LSTMX then LEA: "Esa respuesta es menor que la última vez que el participante fue a México. Por favor verificar respuesta." and siga con P B520.

If B520 is equal to 998 or B520 is equal to 999 or B520 is greater than 6, then siga con P B522.

B521. En los últimos 6 meses, en promedio, que tan seguido usaste drogas alucinógenas como ácido, LSD, peyote, mezcalina, o hongos mientras estabas en México? (Escoja una)

00	Nunca
01	1 vez al mes o menos
02	2-3 días al mes
03	Una vez a la semana
04	2-3 días a la semana
05	4-6 días a la semana
06	Una vez al día
07	2-3 veces al día
08	4 veces o mas por día
98	Se rehúsa a contestar

B522. ¿Alguna vez has usado inhalantes (goma de pegamento o gasolina) en México?

1	Si
0	No
8	Se rehúsa a contestar

If B522 is not equal to 1, then siga con P B525.

B523. ¿Cuándo fue la última vez que usaste inhalantes (goma de pegamento o gasolina) en México?

__ __ __	AñOS
__ __ __	MESES
__ __ __	DíAS
998	Se rehúsa a contestar (Meses)
999	No corresponde (Meses)

If B523 is less than LSTMX then LEA: "Esa respuesta es menor que la última vez que el participante fue a México. Por favor verificar respuesta." and siga con P B523.

If B523 is equal to 998 or B523 is equal to 999 or B523 is greater than 6, then siga con P B525.

B524. Durante los últimos 6 meses, en promedio, ¿qué tan seguido usaste inhalantes como goma de pegamento o gasolina) cuando estuviste en México? (Escoja una)

00	Nunca
01	1 vez al mes o menos
02	2-3 días al mes
03	Una vez a la semana
04	2-3 días a la semana
05	4-6 días a la semana
06	Una vez al día
07	2-3 veces al día
08	4 veces o mas por día
98	Se rehúsa a contestar

B525. ¿Alguna vez has usado tranquilizantes (Diazepam, Valium, Ativan, Restoril o Rivotril) en México?

1	Si
0	No
8	Se rehúsa a contestar

If B525 is not equal to 1, then siga con P B531.

B526. ¿Cuándo fue la última vez que usaste tranquilizantes (Diazepam, Valium, Ativan, Restoril o Rivotril) en México?

__ __ __	AñOS
__ __ __	MESES
__ __ __	DíAS
998	Se rehúsa a contestar (Meses)
999	No corresponde (Meses)

If B526 is less than LSTMX then LEA: "Esa respuesta es menor que la última vez que el participante fue a México. Por favor verificar respuesta." and siga con P B526.

If B526 is equal to 998 or B526 is equal to 999 or B526 is greater than 6, then siga con P B531.

B527. Si usaste tranquilizantes (Diazepam, Valium, Ativan, Restoril o Rivotril) en los últimos 6 meses cuando estuviste en México, ¿Cómo los usaste?
Entrevistador: Si el participante indica que no usó tranquilizantes **en México en los últimos 6 meses, marque No corresponde.**
(Marque todas las que apliquen)

__ Inyectado

__ Fumado (chasing)

__ Aspirado

__ Inhalado

__ Tomado

__ Rectal (bumping)

__ Otro

__ No corresponde

__ Se rehúsa a contestar

If B527 is greater than 1 and B527H is equal to 1 then LEA: "Solo puedes escoger la opcion "No corresponde" por si sola, verificar respuesta." and siga con P B527.

If B527G is not equal to 1, then siga con P instrucción antes de B529.

B528. Especifica de que otra manera has usado tranquilizantes (Diazepam, Valium, Ativan, , Restoril o Rivotril) en los últimos 6 meses en México.

__ __

If B527A is not equal to 1, then siga con P instrucción antes de B530.

B529. Durante los últimos 6 meses, cuando usaste tranquilizantes (Diazepam, Valium, Ativan, Restoril o Rivotril) en promedio ¿Qué tan seguido te inyectaste cuando estuviste en México? (Escoja una)

00	Nunca
01	1 vez al mes o menos
02	2-3 días al mes
03	Una vez a la semana
04	2-3 días a la semana
05	4-6 días a la semana
06	Una vez al día
07	2-3 veces al día
08	4 veces o mas por día
98	Se rehúsa a contestar

If B527H is equal to 1 or B527 is equal to 98, then siga con P B531.

B530. Durante los últimos 6 meses, cuando usaste tranquilizantes (Diazepam, Valium, Ativan, Restoril o Rivotril) en promedio ¿Qué tan seguido los usaste de otras maneras (no inyectada) cuando estuviste en México? (Escoja una)

00	Nunca
01	1 vez al mes o menos
02	2-3 días al mes
03	Una vez a la semana
04	2-3 días a la semana
05	4-6 días a la semana
06	Una vez al día
07	2-3 veces al día
08	4 veces o mas por día
98	Se rehúsa a contestar

B531. ¿Alguna vez haz usado barbitúricos (Amytal, Nembutal, o Seconal) en México?

1	Si
0	No
8	Se rehúsa a contestar

If B531 is not equal to 1, then siga con P B537.

B532. ¿Cuándo fue la última vez que tomaste barbitúricos (Amytal, Nembutal, o Seconal) en México?

__ __ __	AñOS
__ __ __	MESES
__ __ __	DíAS
998	Se rehúsa a contestar (Meses)
999	No corresponde (Meses)

If B532 is less than LSTMX then LEA: "Esa respuesta es menor que la última vez que el participante fue a México. Por favor verificar respuesta." and siga con P B532.

If B532 is equal to 998 or B532 is equal to 999 or B532 is greater than 6, then siga con P B537.

B533. Si usaste barbitúricos (Amytal, Nembutal, o Seconal) en los últimos 6 meses mientras estuviste en México, ¿Cómo los usaste?
Entrevistador: Si el participante indica que no usó barbitúricos **en México en los últimos 6 meses, marque No corresponde.**
(Marque todas las que apliquen)

__ Inyectado

__ Fumado (chasing)

__ Aspirado

__ Inhalado

__ Tomado

__ Rectal (bumping)

__ Otro

__ No corresponde

__ Se rehúsa a contestar

If B533 is greater than 1 and B533H is equal to 1 then LEA: "Solo puedes escoger la opcion "No corresponde" por si sola, verificar respuesta." and siga con P B533.

If B533G is not equal to 1, then siga con P instrucción antes de B535.

B534. Especifica de que otra manera usaste barbitúricos (Amytal, Nembutal, o Seconal) en los últimos 6 meses en México.

____

If B533A is not equal to 1, then siga con P instrucción antes de B536.

B535. Durante los últimos 6 meses, cuando usaste barbitúricos (Amytal, Nembutal, o Seconal) cuando estuviste en México, en promedio ¿Qué tan seguido te los inyectaste? (Escoja una)

00	Nunca
01	1 vez al mes o menos
02	2-3 días al mes
03	Una vez a la semana
04	2-3 días a la semana
05	4-6 días a la semana
06	Una vez al día
07	2-3 veces al día
08	4 veces o mas por día
98	Se rehúsa a contestar

If B533H is equal to 1 or B533 is equal to 98, then siga con P B537.

B536. Durante los últimos 6 meses, cuando usaste barbitúricos (Amytal, Nembutal, o Seconal) cuando estuviste en México, en promedio ¿Qué tan seguido lo usaste de otra manera (no inyectada)? (Escoja una)

00	Nunca
01	1 vez al mes o menos
02	2-3 días al mes
03	Una vez a la semana
04	2-3 días a la semana
05	4-6 días a la semana
06	Una vez al día
07	2-3 veces al día
08	4 veces o mas por día
98	Se rehúsa a contestar

B537. ¿Alguna vez has usado píldoras del cerebro (modafilino, nootropicos, ritalin) en México?

1	Si
0	No
8	Se rehúsa a contestar

If B537 is not equal to 1, then siga con P B543.

B538. ¿Cuándo fue la última vez que usaste píldoras del cerebro (modafilino, nootropicos, ritalin) en México?

__ __ __	AñOS
__ __ __	MESES
__ __ __	DíAS
998	Se rehúsa a contestar (Meses)
999	No corresponde (Meses)

If B538 is less than LSTMX then LEA: "Esa respuesta es menor que la última vez que el participante fue a México. Por favor verificar respuesta." and siga con P B538.

If B538 is equal to 998 or B538 is equal to 999 or B538 is greater than 6, then siga con P B543.

B539. Si usaste píldoras del cerebro (nootropicos, modafilino, ritalin) en los últimos 6 meses cuando estuviste en México, ¿Cómo los usaste?
Entrevistador: Si el participante indica que no usó píldoras del cerebro **en México en los últimos 6 meses, marque No corresponde.**
(Marque todas las que apliquen)

__ Inyectado

__ Fumado (chasing)

__ Aspirado

__ Inhalado

__ Tomado

__ Rectal (bumping)

__ Otro

__ No corresponde

__ Se rehúsa a contestar

If B539 is greater than 1 and B539H is equal to 1 then LEA: "Solo puedes escoger la opcion "No corresponde" por si sola, verificar respuesta." and siga con P B539.

If B539G is not equal to 1, then siga con P instrucción antes de B541.

B540. Especifica otras maneras en las que usaste píldoras del cerebro (nootropicos, modafilino, ritalin) en los últimos 6 meses cuando estuviste en México.

__ __

If B539A is not equal to 1, then siga con P instrucción antes de B542.

B541. En los últimos 6 meses, cuando usaste píldoras del cerebro (nootropicos, modafilino, ritalin) en México, en promedio ¿Qué tan seguido te inyectabas? (Escoja una)

00	Nunca
01	1 vez al mes o menos
02	2-3 días al mes
03	Una vez a la semana
04	2-3 días a la semana
05	4-6 días a la semana
06	Una vez al día
07	2-3 veces al día
08	4 veces o mas por día
98	Se rehúsa a contestar

If B539H is equal to 1 or B539 is equal to 98, then siga con P B543.

B542. En los últimos 6 meses, cuando usaste píldoras del cerebro (nootropicos, modafilino, ritalin) en México, en promedio ¿Qué tan seguido las usaste de otra manera (no inyectada)? (Escoja una)

00	Nunca
01	1 vez al mes o menos
02	2-3 días al mes
03	Una vez a la semana
04	2-3 días a la semana
05	4-6 días a la semana
06	Una vez al día
07	2-3 veces al día
08	4 veces o mas por día
98	Se rehúsa a contestar

B543. ¿Alguna vez has usado otra droga sola (no mencionada anteriormente) en México?**Entrevistador, por favor pregunte sobre sales de baño**

1	Si
0	No
8	Se rehúsa a contestar

If B543 is not equal to 1, then siga con P B557.

B544. Por favor especifica la **primera** otra droga que has usado sola cuando estuviste en México.

____

B545. ¿Cuándo fue la última vez que usaste [Respuesta a P B544] sola en México?

__ __ __	AñOS
__ __ __	MESES
__ __ __	DíAS
998	Se rehúsa a contestar (Meses)
999	No corresponde (Meses)

If B545 is less than LSTMX then LEA: "Esa respuesta es menor que la última vez que el participante fue a México. Por favor verificar respuesta." and siga con P B545.

If B545 is equal to 998 or B545 is equal to 999 or B545 is greater than 6, then siga con P B550.

B546. Si usaste [Respuesta a P B544] en los últimos 6 meses mientras estuviste en México, ¿Cómo los usaste?
Entrevistador: Si el participante indica que no usó [Respuesta a P B544] **en México en los últimos 6 meses, marque No corresponde.**
(Marque todas las que apliquen)

__	Inyectado
__	Fumado (chasing)
__	Aspirado
__	Inhalado
__	Tomado
__	Rectal (bumping)
__	Otro
__	No corresponde
__	Se rehúsa a contestar

If B546 is greater than 1 and B546H is equal to 1 then LEA: "Solo puedes escoger la opcion "No corresponde" por si sola, verificar respuesta." and siga con P B546.

If B546G is not equal to 1, then siga con P instrucción antes de B548.

B547. Especifica de que otra manera usaste [Respuesta a P B544] en los últimos 6 meses mientras estuviste en México.

____

If B546A is not equal to 1, then siga con P instrucción antes de B549.

B548. Durante los últimos 6 meses, cuando usaste [Respuesta a P B544] en México, en promedio ¿Qué tan seguido te la inyectaste? (Escoja una)

00	Nunca
01	1 vez al mes o menos
02	2-3 días al mes
03	Una vez a la semana
04	2-3 días a la semana
05	4-6 días a la semana
06	Una vez al día
07	2-3 veces al día
08	4 veces o mas por día
98	Se rehúsa a contestar

If B546H is equal to 1 or B546 is equal to 98, then siga con P B550.

B549. Durante los últimos 6 meses, cuando usaste [Respuesta a P B544] cuando estuviste en México, en promedio ¿Qué tan seguido lo usaste de otra manera (no inyectada)? (Escoja una)

00	Nunca
01	1 vez al mes o menos
02	2-3 días al mes
03	Una vez a la semana
04	2-3 días a la semana
05	4-6 días a la semana
06	Una vez al día
07	2-3 veces al día
08	4 veces o mas por día
98	Se rehúsa a contestar

B550. ¿Alguna vez has usado otra droga sola (no mencionada anteriormente) cuando estuviste en México?

1	Si
0	No
8	Se rehúsa a contestar

If B550 is not equal to 1, then siga con P B557.

B551. ¿Alguna vez has usado otra droga sola (no mencionada anteriormente) cuando estuviste en México?

____

B552. ¿Cuándo fue la última vez que usaste [Respuesta a P B551] sola en México?

__ __ __	AñOS
__ __ __	MESES
__ __ __	DíAS
998	Se rehúsa a contestar (Meses)
999	No corresponde (Meses)

If B552 is less than LSTMX then LEA: "Esa respuesta es menor que la última vez que el participante fue a México. Por favor verificar respuesta." and siga con P B552.

If B552 is equal to 998 or B552 is equal to 999 or B552 is greater than 6, then siga con P B557.

B553. Si usaste [Respuesta a P B551] en los últimos 6 meses mientras estuviste en México, ¿Cómo los usaste?
Entrevistador: Si el participante indica que no usó [Respuesta a P B551] **en México en los últimos 6 meses, marque No corresponde.**
(Marque todas las que apliquen)

__	Inyectado
__	Fumado (chasing)
__	Aspirado
__	Inhalado
__	Tomado
__	Rectal (bumping)
__	Otro
__	No corresponde
__	Se rehúsa a contestar

If B553 is greater than 1 and B553H is equal to 1 then LEA: "Solo puedes escoger la opcion "No corresponde" por si sola, verificar respuesta." and siga con P B553.

If B553G is not equal to 1, then siga con P instrucción antes de B555.

B554. Especifica de que otra manera usaste [Respuesta a P B551] en los últimos 6 meses mientras estuviste en México.

____

If B553A is not equal to 1, then siga con P instrucción antes de B556.

B555. Durante los últimos 6 meses, cuando usaste [Respuesta a P B551] cuando estuviste en México, en promedio ¿Qué tan seguido te lo inyectaste? (Escoja una)

00	Nunca
01	1 vez al mes o menos
02	2-3 días al mes
03	Una vez a la semana
04	2-3 días a la semana
05	4-6 días a la semana
06	Una vez al día
07	2-3 veces al día
08	4 veces o mas por día
98	Se rehúsa a contestar

If B553H is equal to 1 or B553 is equal to 98, then siga con P B557.

B556. Durante los últimos 6 meses, cuando usaste [Respuesta a P B551] cuando estuviste en México, en promedio ¿Qué tan seguido lo usaste de otra manera (no inyectada)? (Escoja una)

00	Nunca
01	1 vez al mes o menos
02	2-3 días al mes
03	Una vez a la semana
04	2-3 días a la semana
05	4-6 días a la semana
06	Una vez al día
07	2-3 veces al día
08	4 veces o mas por día
98	Se rehúsa a contestar

B557. ¿Alguna vez has usado otra combinación de drogas (no mencionada anteriormente) en Mexico?

1	Si
0	No
8	Se rehúsa a contestar

If B557 is not equal to 1, then siga con P instrucción antes de B571.

B558. Por favor especifica la **primera** otra combinación de drogas que usaste cuando estuviste en México.

____

B559. ¿Cuándo fue la última vez que usaste [Respuesta a P B558] sola en México?

__ __ __	AñOS
__ __ __	MESES
__ __ __	DíAS
998	Se rehúsa a contestar (Meses)
999	No corresponde (Meses)

If B559 is less than LSTMX then LEA: "Esa respuesta es menor que la última vez que el participante fue a México. Por favor verificar respuesta." and siga con P B559.

If B559 is equal to 998 or B559 is equal to 999 or B559 is greater than 6, then siga con P B564.

B560. Cuando usaste [Respuesta a P B558] en los últimos 6 meses mientras estuviste en México, ¿Cómo los usaste?
Entrevistador: Si el participante indica que no usó [Respuesta a P B558] **en México en los últimos 6 meses, marque No corresponde.**
(Marque todas las que apliquen)

__	Inyectado
__	Fumado (chasing)
__	Aspirado
__	Inhalado
__	Tomado
__	Rectal (bumping)
__	Otro
__	No corresponde
__	Se rehúsa a contestar

If B560 is greater than 1 and B560H is equal to 1 then LEA: "Solo puedes escoger la opcion "No corresponde" por si sola, verificar respuesta." and siga con P B560.

If B560G is not equal to 1, then siga con P instrucción antes de B562.

B561. Especifica de que otra manera usaste [Respuesta a P B558] en los últimos 6 meses mientras estuviste en México.

____

If B560A is not equal to 1, then siga con P instrucción antes de B563.

B562. Durante los últimos 6 meses, cuando usaste [Respuesta a P B558] en México, en promedio ¿Qué tan seguido te lo inyectaste? (Escoja una)

00	Nunca
01	1 vez al mes o menos
02	2-3 días al mes
03	Una vez a la semana
04	2-3 días a la semana
05	4-6 días a la semana
06	Una vez al día
07	2-3 veces al día
08	4 veces o mas por día
98	Se rehúsa a contestar

If B560H is equal to 1 or B560 is equal to 98, then siga con P B564.

B563. Durante los últimos 6 meses, cuando usaste [Respuesta a P B558] cuando estuviste en México, en promedio ¿Qué tan seguido lo usaste de otra manera (no inyectada)? (Escoja una)

00	Nunca
01	1 vez al mes o menos
02	2-3 días al mes
03	Una vez a la semana
04	2-3 días a la semana
05	4-6 días a la semana
06	Una vez al día
07	2-3 veces al día
08	4 veces o mas por día
98	Se rehúsa a contestar

B564. ¿Alguna vez has usado otra droga sola (no mencionada anteriormente) cuando estuviste en México?

1	Si
0	No
8	Se rehúsa a contestar

If B564 is not equal to 1, then siga con P instrucción antes de B571.

B565. Por favor especifica la **segunda** otra combinación de drogas que usaste cuando estuviste en México.

____

B566. ¿Cuándo fue la última vez que usaste [Respuesta a P B565] en México?

__ __ __ AñOS

__ __ __ MESES

__ __ __ DíAS

998 Se rehúsa a contestar (Meses)

999 No corresponde (Meses)

If B566 is less than LSTMX then LEA: "Esa respuesta es menor que la última vez que el participante fue a México. Por favor verificar respuesta." and siga con P B566.

If B566 is equal to 998 or B566 is equal to 999 or B566 is greater than 6, then siga con P instrucción antes de B571.

B567. Cuando usaste [Respuesta a P B565] en los últimos 6 meses mientras estuviste en México, ¿Cómo los usaste?
Entrevistador: Si el participante indica que no usó [Respuesta a P B565] **en México en los últimos 6 meses, marque No corresponde.**
(Marque todas las que apliquen)

__ Inyectado

__ Fumado (chasing)

__ Aspirado

__ Inhalado

__ Tomado

__ Rectal (bumping)

__ Otro

__ No corresponde

__ Se rehúsa a contestar

If B567 is greater than 1 and B567H is equal to 1 then LEA: "Solo puedes escoger la opcion "No corresponde" por si sola, verificar respuesta." and siga con P B567.

If B567G is not equal to 1, then siga con P instrucción antes de B569.

B568. Especifica de que otra manera usaste [Respuesta a P B565] en los últimos 6 meses mientras estuviste en México.

____

If B567A is not equal to 1, then siga con P instrucción antes de B570.

B569. Durante los últimos 6 meses, cuando usaste [Respuesta a P B565] en México, en promedio ¿Qué tan seguido te lo inyectaste? (Escoja una)

00	Nunca
01	1 vez al mes o menos
02	2-3 días al mes
03	Una vez a la semana
04	2-3 días a la semana
05	4-6 días a la semana
06	Una vez al día
07	2-3 veces al día
08	4 veces o mas por día
98	Se rehúsa a contestar

If B567H is equal to 1 or B567 is equal to 98, then siga con P instrucción antes de B571.

B570. Durante los últimos 6 meses, cuando usaste [Respuesta a P B565] cuando estuviste en México, en promedio ¿Qué tan seguido lo usaste de otra manera (no inyectada)? (Escoja una)

00	Nunca
01	1 vez al mes o menos
02	2-3 días al mes
03	Una vez a la semana
04	2-3 días a la semana
05	4-6 días a la semana
06	Una vez al día
07	2-3 veces al día
08	4 veces o mas por día
98	Se rehúsa a contestar

ANYINJMX = If(B440 is not equal to 0 and B440 is not equal to 98 and B440 is not equal to "Salto" or B454 is not equal to 0 and B454 is not equal to 98 and B454 is not equal to "Salto" or B457 is not equal to 0 and B457 is not equal to 98 and B457 is not equal to "Salto" or B464 is not equal to 0 and B464 is not equal to 98 and B464 is not equal to "Salto" or B467 is not equal to 0 and B467 is not equal to 98 and B467 is not equal to "Salto" or B470 is not equal to 0 and B470 is not equal to 98 and B470 is not equal to "Salto" or B481 is not equal to 0 and B481 is not equal to 98 and B481 is not equal to "Salto" or B484 is not equal to 0 and B484 is not equal to 98 and B484 is not equal to "Salto" or B490 is not equal to 0 and B490 is not equal to 98 and B490 is not equal to "Salto" or B491 is not equal to 0 and B491 is not equal to 98 and B491 is not equal to "Salto" or B499 is not equal to 0 and B499 is not equal to 98 and B499 is not equal to "Salto" or B505 is not equal to 0 and B505 is not equal to 98 and B505 is not equal to "Salto" or B511 is not equal to 0 and B511 is not equal to 98 and B511 is not equal to "Salto" or B517 is not equal to 0 and B517 is not equal to 98 and B517 is not equal to "Salto" or B529 is not equal to 0 and B529 is not equal to 98 and B529 is not equal to "Salto" or B535 is not equal to 0 and B535 is not equal to 98 and B535 is not equal to "Salto" or B541 is not equal to 0 and B541 is not equal to 98 and B541 is not equal to "Salto" or B548 is not equal to 0 and B548 is not equal to 98 and B548 is not equal to "Salto" or B555 is not equal to 0 and B555 is not equal to 98 and B555 is not equal to "Salto" or B562 is not equal to 0 and B562 is not equal to 98 and B562 is not equal to "Salto" or B569 is not equal to 0 and B569 is not equal to 98 and B569 is not equal to "Salto", 1, 0)

LEA: ENTREVISTADOR LEER EN VOZ ALTA: Las siguientes preguntas tienen que ver con las maneras en las que hayas podido inyectarte drogas cuando estuviste en México. Yo sé que ya te había preguntado antes pero me podrías recordar…

If LSTMX is greater than 6 and LSTMX is not equal to 998, then siga con P B591.

B571. Durante los últimos 6 meses cuando estuviste en México, en promedio ¿Qué tan seguido te inyectaste cualquier droga o combinación de drogas? (Escoja una)

00	Nunca
01	1 vez al mes o menos
02	2-3 días al mes
03	Una vez a la semana
04	2-3 días a la semana
05	4-6 días a la semana
06	Una vez al día
07	2-3 veces al día
08	4 veces o mas por día
98	Se rehúsa a contestar

B572. De las veces que se inyectó en los **últimos 6 meses cuando estuviste en México,** ¿qué tan seguido le dio, rentó, prestó a alguien una jeringa que usted ya había usado? (Escoja una)

1	Nunca
2	A veces
3	Casi la mitad de las veces
4	Seguido
5	Siempre
8	Se rehúsa a contestar

B573. De las veces que se inyectó en los **últimos 6 meses cuando estuviste en México**, ¿qué tan seguido uso una jeringa que usted sabía o sospechaba que alguien más ya la había usado? (Escoja una)

1	Nunca
2	A veces
3	Casi la mitad de las veces
4	Seguido
5	Siempre
8	Se rehúsa a contestar

If B573 is equal to 1 or B573 is equal to 8, then siga con P B576.

B574. Durante la última vez que se inyecto drogas en México, ¿uso una jeringa que usted sabia o sospechaba hubiera sido usada por alguien más?

1	Si
0	No
8	Se rehúsa a contestar

B575. De las veces en los **últimos 6 meses** que se inyectó en México con una jeringa que ya había sido usada por alguien más, ¿qué tan seguido limpió la jeringa con cloro antes de usarla? (Escoja una)

1	Nunca
2	A veces
3	Casi la mitad del tiempo
4	Seguido
5	Siempre
8	Se rehúsa a contestar

B576. De las veces que se inyectó en México con otras personas en los **últimos 6 meses**, ¿qué tan seguido ha dividido drogas con alguien más usando una jeringa? Esto también es conocido como backloading, piggybacking o dividir drogas mojadas. (Escoja una)

1 Nunca

2 A veces

3 Casi la mitad del tiempo

4 Seguido

5 Siempre

8 Se rehúsa a contestar

B577. De las veces que se inyectó en los **últimos 6 meses mientras estuvo en** México, ¿qué tan seguido usó un cacharro, algodón o agua de enjuague con alguien o después que alguien más ya lo había usado?
(Escoja una)

1 Nunca

2 A veces

3 Casi la mitad del tiempo

4 Seguido

5 Siempre

8 Se rehúsa a contestar

B578. De las veces que se inyectó en los **últimos 6 meses mientras estuviste en** México, ¿qué tan seguido compró drogas que ya venían preparadas en la jeringa? (Escoja una)

1 Nunca

2 A veces

3 Casi la mitad del tiempo

4 Seguido

5 Siempre

8 Se rehúsa a contestar

B579. De las veces que se inyectó en los **últimos 6 meses mientras estuviste en** México, ¿qué tan seguido se inyectó con una jeringa nueva, estéril? (Escoja una)

1 Nunca

2 A veces

3 Casi la mitad del tiempo

4 Seguido

5 Siempre

8 Se rehúsa a contestar

B580. En los últimos 6 meses que se inyectó drogas mientras estuvo en México, ¿dónde consiguió la jeringa que Ud. uso para inyectarse drogas? (Marque todas las que apliquen)

__ Farmacia

__ Esposo (a), familiar, o pareja sexual

__ Amigo (a)

__ Un Traficante

__ Doctor que ayuda a inyectarse a cambio de drogas o dinero

__ Picadero

__ Intercambio de Jeringas (usted lo cambió)

__ De alguien que los obtiene de un intercambio de Jeringas

__ Comprada en la calle

__ Doctor / clínica / hospital

__ Clínica Veterinaria/ tienda de mascotas

__ Mercado/Tienda

__ Encontrada en el suelo o la basura

__ Otro lugar

__ Se rehúsa a contestar

If B580N is not equal to 1, then siga con P instrucción antes de B582.

B581. Por favor especifique en qué otro lugar usted consiguió las jeringas en los últimos 6 meses mientras estuvo en México.

____

If B580A + B580B + B580C + B580D + B580E + B580F + B580G + B580H + B580I + B580J + B580K + B580L + B580M + B580N is equal to 1 or B580 is equal to 98, then siga con P instrucción antes de B584.

B582. En los últimos 6 meses mientras estuvo en México, ¿dónde consiguió sus jeringas más seguido? (Escoja una)

01	Farmacia
02	Esposo (a), familiar, o pareja sexual
03	Amigo (a)
04	Un Traficante
05	Doctor que ayuda a inyectarse a cambio de drogas o dinero
06	Picadero
07	Intercambio de Jeringas (usted lo cambió)
08	De alguien que los obtiene de un intercambio de Jeringas
09	Comprada en la calle
10	Doctor / clínica / hospital
11	Clínica Veterinaria/ tienda de mascotas
12	Mercado/Tienda
13	Encontrada en el suelo o la basura
14	Otro lugar
98	Se rehúsa a contestar

If B582 is equal to 1 and B580A is not equal to 1 or B582 is equal to 2 and B580B is not equal to 1 or B582 is equal to 3 and B580C is not equal to 1 or B582 is equal to 4 and B580D is not equal to 1 or B582 is equal to 5 and B580E is not equal to 1 or B582 is equal to 6 and B580F is not equal to 1 or B582 is equal to 7 and B580G is not equal to 1 or B582 is equal to 8 and B580H is not equal to 1 or B582 is equal to 9 and B580I is not equal to 1 or B582 is equal to 10 and B580J is not equal to 1 or B582 is equal to 11 and B580K is not equal to 1 or B582 is equal to 12 and B580L is not equal to 1 or B582 is equal to 13 and B580M is not equal to 1 or B582 is equal to 14 and B580N is not equal to 1 then LEA: "El participante solo puede elegir el lugar con más frecuencia de entre los lugares nombrados anteriormente." and siga con P B582.

If B582 is not equal to 14, then siga con P instrucción antes de B584.

B583. Por favor especifique en qué lugar usted consiguió sus jeringas más seguido, en los últimos 6 meses mientras estuvo en México.

____

If B580G is equal to 1 or B582 is equal to 7 then siga con P B584

If not, then siga con P instrucción antes de B586.

B584. En los últimos 6 meses, cuando estuviste en México, ¿cuantas jeringas estériles y limpias obtenías por visita al intercambio de jeringas en promedio?

__ __ __

998 Se rehúsa a contestar

If B584 is equal to 0 or B584 is equal to 998, then siga con P instrucción antes de B586.

B585. ¿Cuál es el nombre del programa de intercambio de jeringa de donde obtuviste tus jeringas más seguido en México?

____

If B580A is equal to 1 or B582 is equal to 1 then siga con P B586

If not, then siga con P B588.

B586. En los últimos 6 meses, cuando estuviste en México, ¿cuantas jeringas estériles y limpias obtuviste en cada visita a la farmacia en promedio?

__ __ __

998 Se rehúsa a contestar

If B586 is equal to 0 or B586 is equal to 998, then siga con P B588.

B587. ¿Cuál es el nombre de la farmacia en la que compraste jeringas más seguido en México?

____

B588. En los últimos 6 meses mientras estuvo en México, cuando se inyectaba drogas ¿qué tan difícil o fácil fue conseguir jeringas nuevas, que no han sido usadas? (Escoja una)

1 Nunca ha tratado de conseguir jeringas nuevas que no han sido usadas

2 Bastante fácil

3 Fácil

4 Difícil

5 Bastante difícil

8 Se rehúsa a contestar

If B588 is equal to 4 or B588 is equal to 5 then siga con P B589

If not, then siga con P B591.

B589. ¿Cuál fue la razón principal por la que fue difícil conseguir jeringas nuevas, que no han sido usadas mientras estuvo en México? (Escoja una)

01 No sabia donde obtenerlas

02 Cuestan demasiado/ No tengo dinero para comprarlas

03 No hay ningún sitio donde comprar jeringas legalmente/ la tienda no me las vende

04 No hay ningún lugar de intercambio de jeringas

05 Me da miedo que me encuentren /arresten la policía por llevar jeringas

06 La farmacia/tienda estaba cerrada

07 Otro

97 No sé

98 Se rehúsa a contestar

If B589 is not equal to 7, then siga con P B591.

B590. Por favor especifique cuál fue la otra razón por la cual fue difícil conseguir jeringas nuevas, que no han sido usadas mientras estuvo en México.

__ __

B591. ¿Ha tenido alguna ayuda de alguien más (como un hit doctor) para inyectarse drogas cuando Ud. estaba en México?

1 Si

0 No

8 Se rehúsa a contestar

If B591 is not equal to 1, then siga con P B594.

B592. ¿Cuándo fue la última vez que alguien le ayudó a inyectarse drogas mientras estuvo en México?

__ __ __ MESES

__ __ __ SEMANAS

__ __ __ DíAS

998 Se rehúsa a contestar (Meses)

If B592 is less than LSTMX then LEA: "Esa respuesta es menor que la última vez que el participante fue a México. Por favor verificar respuesta." and siga con P B592.

If B592 is equal to 998 or B592 is equal to 999 or B592 is greater than 6, then siga con P B594.

B593. En los últimos 6 meses cuando estuvo en México, ¿qué tan seguido se ha inyectado con la ayuda de alguien? (Escoja una)

1 Siempre

2 Con frecuencia

3 A veces

4 Casi nunca

8 Se rehúsa a contestar

B594. Por favor, seleccione el tipo de moneda en el que usted sabe que cuesta la droga en Mexico. (Escoja una)

1 Pesos

2 Dólares

8 Se rehúsa a contestar

If B594 is equal to 2 then siga con P B595

If not, then siga con P instrucción antes de B610.

B595. En dólares ¿Cuánto cuesta comprar una dosis de Heroina en goma/Goma negra (la negra) sola en México?

__ __ __ dólares

997 No sé

998 Se rehúsa a contestar

B596. En dólares ¿Cuánto cuesta comprar una dosis de heroína en polvo sola en México?

__ __ __ dólares

997 No sé

998 Se rehúsa a contestar

B597. En dólares ¿Cuánto cuesta comprar una dosis de cocaína (crack) sola en México?

__ __ __	dólares
997	No sé
998	Se rehúsa a contestar

B598. En dólares ¿Cuánto cuesta comprar una dosis de cocaína (polvo) en México?

__ __ __	dólares
997	No sé
998	Se rehúsa a contestar

B599. En dólares ¿Cuánto cuesta comprar una dosis de heroína y cocaína juntas (speedball) en México?

__ __ __	dólares
997	No sé
998	Se rehúsa a contestar

B600. En dólares ¿Cuánto cuesta comprar una dosis de metanfetamina/cristal sola en México?

__ __ __	dólares
997	No sé
998	Se rehúsa a contestar

B601. En dólares ¿Cuánto cuesta comprar una dosis de China White en México?

__ __ __	dólares
997	No sé
998	Se rehúsa a contestar

B602. En dólares ¿Cuánto cuesta comprar una dosis de OxyContin sin receta en México?

__ __ __	dólares
997	No sé
998	Se rehúsa a contestar

B603. En dólares ¿Cuánto cuesta comprar una dosis de otros opiáceos controlados sin receta (Vicodin, Darvon, Percocet) en México?

__ __ __	dólares
997	No sé
998	Se rehúsa a contestar

B604. En dólares ¿Cuánto cuesta comprar una dosis de tranquilizantes (Rivotril, Diazepam, Valium, Ativan o Restoril) en México?

__ __ __	dólares
997	No sé
998	Se rehúsa a contestar

B605. En dólares ¿Cuánto cuesta comprar una dosis de barbitúricos sin receta (Amytal, Nembutal y Seconal)
en México?

__ __ __	dólares
997	No sé
998	Se rehúsa a contestar

B606. En dólares ¿Cuánto cuesta comprar una dosis de fentanilo (fentanyl) en México?

__ __ __	dólares
997	No sé
998	Se rehúsa a contestar

B607. ¿Conoce el costo en dólares de Otras drogas o combinaciones que no se haya mencionado en la lista anterior en México?

1 Si

0 No

8 Se rehúsa a contestar

If B607 is not equal to 1, then siga con P instrucción antes de B610.

B608. En dólares ¿Cuánto cuesta comprar una dosis de otras drogas o combinaciones que no se haya mencionado en la lista anterior en México?

__ __ __ dólares

997 No sé

998 Se rehúsa a contestar

If B608 is greater than 0 and B608 is not equal to 998 then siga con P B609

If not, then siga con P instrucción antes de B610.

B609. Otra droga o combinacion de la que diste costo anteriormente

__ __

If B594 is equal to 1 then siga con P B610

If not, then siga con P B625.

B610. En pesos ¿Cuánto cuesta comprar una dosis de Heroina en goma/Goma negra (la negra) sola en México?

__ __ __ pesos

997 No sé

998 Se rehúsa a contestar

B611. En pesos ¿Cuánto cuesta comprar una dosis de heroína en polvo sola en México?

__ __ __	pesos
997	No sé
998	Se rehúsa a contestar

B612. En pesos ¿Cuánto cuesta comprar una dosis de cocaína (crack) sola en México?

__ __ __	pesos
997	No sé
998	Se rehúsa a contestar

B613. En pesos ¿Cuánto cuesta comprar una dosis de cocaína (polvo) en México?

__ __ __	pesos
997	No sé
998	Se rehúsa a contestar

B614. En pesos ¿Cuánto cuesta comprar una dosis de heroína y cocaína juntas (speedball) en México?

__ __ __	pesos
997	No sé
998	Se rehúsa a contestar

B615. En pesos ¿Cuánto cuesta comprar una dosis de metanfetamina/cristal sola en México?

__ __ __	pesos
997	No sé
998	Se rehúsa a contestar

B616. En pesos ¿Cuánto cuesta comprar una dosis de China White en México?

__ __ __ pesos

997 No sé

998 Se rehúsa a contestar

B617. En pesos ¿Cuánto cuesta comprar una dosis de OxyContin sin receta en México?

__ __ __ pesos

997 No sé

998 Se rehúsa a contestar

B618. En pesos ¿Cuánto cuesta comprar una dosis de otros opiáceos controlados sin receta (Vicodin, Darvon, Percocet) en México?

__ __ __ pesos

997 No sé

998 Se rehúsa a contestar

B619. En pesos ¿Cuánto cuesta comprar una dosis de tranquilizantes (Rivotril, Diazepam, Valium, Ativan o Restoril) en México?

__ __ __ pesos

997 No sé

998 Se rehúsa a contestar

B620. En pesos ¿Cuánto cuesta comprar una dosis de barbitúricos sin receta (Amytal, Nembutal o Seconal)
en México?

__ __ __ pesos

997 No sé

998 Se rehúsa a contestar

B621. En pesos ¿Cuánto cuesta comprar una dosis de fentanilo (fentanyl) en México?

__ __ __	pesos
997	No sé
998	Se rehúsa a contestar

B622. ¿Conoce el costo en pesos de Otras drogas o combinaciones que no se haya mencionado en la lista anterior en México?

1	Si
0	No
8	Se rehúsa a contestar

If B622 is not equal to 1, then siga con P B625.

B623. En pesos ¿Cuánto cuesta comprar una dosis de otras drogas o combinaciones que no se haya mencionado en la lista anterior en México?

__ __ __	pesos
997	No sé
998	Se rehúsa a contestar

If B623 is greater than 0 and B623 is not equal to 998 then siga con P B624

If not, then siga con P B625.

B624. Otra droga o combinacion de la que diste costo anteriormente

__ __

PREGUNTAS DE INICIACION DE INJECCION

B625. ¿Alguna vez le has ayudado a inyectarse alguien que nunca antes se había inyectado?

1 Si

0 No

8 Se rehúsa a contestar

If B625 is not equal to 1, then siga con P instrucción antes de B641.

B626. ¿A cuántas personas has ayudado a inyectarse por primera vez?

__ __ __ __

9998 Se rehúsa a contestar

B627. ¿Cuáles han sido las razones por las que has ayudado a alguien a inyectarse por primera vez? (Marque todas las que apliquen)

__ Compartí drogas con ellos

__ "Hit doctor" (me pagaron para ayudarlos a inyectarse)

__ Quería compartir la experiencia

__ Ellos no sabían cómo inyectarse

__ Ellos estaban muy tembloroso o nerviosos para inyectarse

__ Otro

__ Se rehúsa a contestar

If B627F is not equal to 1, then siga con P B629.

B628. Por favor especifique, otra razón.

__ __

B629. A quien has ayudado a inyectarse por primera vez? (Marque todas las que apliquen)

__ Hermano(a)

__ Pareja sexual casual

__ Pareja sexual comercial

__ Novio(a) o esposo(a)

__ Hijo(a)

__ Les vendí drogas

__ Les vendí jeringas

__ Amigo(a)

__ Conocido(a)

__ Desconocido(a)

__ Compañero en la Prisión

__ Otro

__ Se rehúsa a contestar

If B629L is not equal to 1, then siga con P B631.

B630. Por favor especifique, otra relación

____

B631. ¿En los últimos seis meses has ayudado a alguien a inyectarse por primera vez? (Escoja una)

1 Si

0 No

8 Se rehúsa a contestar

If B631 is not equal to 1, then siga con P instrucción antes de B641.

B632. ¿A cuántas diferentes personas?

__ __ __ __ personas

9998 Se rehúsa a contestar

If B632 is greater than B626 and B632 is not equal to 9998 then LEA: "La respuesta es mayor que el número total. Por favor, verifique las respuesta." and siga con P B632.

B633. ¿Cuántas de las personas a las que les ayudaste viven en México?

__ __ __ __ personas

9998 Se rehúsa a contestar

If B633 is greater than B632 and B633 is not equal to 9998 then LEA: "La respuesta es mayor que el número total. Por favor, verifique las respuesta." and siga con P B633.

B634. ¿Cuántas de las personas a las que les ayudaste viven en Estados Unidos?

__ __ __ __ personas

9998 Se rehúsa a contestar

If B633 is not equal to 9998 and B634 is not equal to 9998 and B633 + B634 is greater than B632 then LEA: "La suma de dos respuestas es mayor que el número total. Por favor, verifique las respuesta." and siga con P B634.

B635. ¿Cuál era el sexo de esta(s) persona(s)? (Marque todas las que apliquen)

__ Hombre

__ Mujer

__ Transgenero

__ Se rehúsa a contestar

B636. ¿Cuáles fueron los motivos por que en los últimos 6 meses has ayudado a alguien a inyectarse que nunca se habia inyectado? **Lea las lista y marque todas las que apliquen.** (Marque todas las que apliquen)

__ Compartí drogas con ellos

__ "Hit doctor" (me pagaron para ayudarlos a inyectarse)

__ Quería compartir la experiencia

__ No sabían como inyectarse

__ Estaban muy temblorosos o nerviosos para inyectarse

__ Otro

__ Se rehúsa a contestar

If B636F is not equal to 1, then siga con P B638.

B637. Por favor especifique, otra razón.

____

B638. Si en los últimos 6 meses ayudaste a alguien a inyectarse por primera vez, ¿cuál era tu relación con esa(s) persona(s)? (Marque todas las que apliquen)

__ Hermano(a)

__ Pareja sexual casual

__ Pareja sexual comercial

__ Novio(a) o esposo(a)

__ Hijo(a)

__ Les vendí drogas

__ Les vendí jeringas

__ Amigo(a)

__ Conocido(a)

__ Desconocido(a)

__ Compañero en la Prisión

__ Otro

__ Se rehúsa a contestar

If B638L is not equal to 1, then siga con P B640.

B639. Por favor especifique, otra relación

__ __

B640. En los últimos 6 meses, ¿compartiste jeringas con alguien a quien le ayudaste a inyectarse que nunca se había inyectado?

1 Si

0 No

8 Se rehúsa a contestar

LEA: LEER: Las siguientes preguntas son sobre qué tan hábiles son las personas para practicar ciertos comportamientos. Por favor responde de la manera que sea de acuerdo a ti.

B641. Puedo evitar inyectarme con una jeringa que alguien más usó, incluso si estoy inyectándome con personas que conozco bien. (Escoja una)

1 Absolutamente seguro que NO PUEDO

2 Muy seguro que NO PUEDO

3 Muy seguro que SI PUEDO

4 Absolutamente seguro que SI PUEDO

8 Se rehúsa a contestar

B642. Puedo evitar inyectarme con una jeringa que alguien más usó, incluso si estoy dopado, enfermo con la malilla. (Escoja una)

1 Absolutamente seguro que NO PUEDO

2 Muy seguro que NO PUEDO

3 Muy seguro que SI PUEDO

4 Absolutamente seguro que SI PUEDO

8 Se rehúsa a contestar

B643. Puedo evitar usar un cacharro, algodón, o agua de enjuague que alguien más usó, incluso si estoy inyectándome con personas que conozco bien. (Escoja una)

1 Absolutamente seguro que NO PUEDO

2 Muy seguro que NO PUEDO

3 Muy seguro que SI PUEDO

4 Absolutamente seguro que SI PUEDO

8 Se rehúsa a contestar

B644. Puedo evitar inyectarme con la jeringa de mi pareja de inyeccion, incluso si antes hemos compartido jeringas. (Escoja una)

1 Absolutamente seguro que NO PUEDO

2 Muy seguro que NO PUEDO

3 Muy seguro que SI PUEDO

4 Absolutamente seguro que SI PUEDO

8 Se rehúsa a contestar

B645. Puedo evitar usar el cacharro, algodón o agua de enjuague de mi pareja, incluso si antes los hemos compartido. (Escoja una)

1 Absolutamente seguro que NO PUEDO

2 Muy seguro que NO PUEDO

3 Muy seguro que SI PUEDO

4 Absolutamente seguro que SI PUEDO

8 Se rehúsa a contestar

B646. Puedo evitar inyectarme con una jeringa que alguien más usó, incluso si he tenido sexo sin condón con esa persona. (Escoja una)

1 Absolutamente seguro que NO PUEDO

2 Muy seguro que NO PUEDO

3 Muy seguro que SI PUEDO

4 Absolutamente seguro que SI PUEDO

8 Se rehúsa a contestar

EXPERIENCIAS DE SOBREDOSIS

LEA: LEA: Las siguientes preguntas son acerca de sobredosis. Cuando digo sobredosis, me refiero a una situación en la que usted haya desmayado y no haya podido despertarse o sus labios se le pusieron azules.

B647. En toda su vida, ¿cuántas veces ha tenido una sobredosis?

__ __ __	veces	
997	No sé	***Siga con P instrucción antes de Q1***
998	Se rehúsa a contestar	***Siga con P instrucción antes de Q1***

If B647 is equal to 0, then siga con P B677.

If NDT_TJ is equal to 1, then siga con P instrucción antes de B649.

B648. De esas veces que tuvo sobredosis, ¿Cuántas veces ocurrieron en EUA?

__ __ __	veces	
997	No sé	***Siga con P instrucción antes de Q1***
998	Se rehúsa a contestar	***Siga con P instrucción antes de Q1***
999	No corresponde	***Siga con P instrucción antes de Q1***

If B648 is greater than B647 and B648 is not equal to 997 and B648 is not equal to 998 and B648 is not equal to 999 then LEA: "Esa respuesta es mayor que la cantidad de sobredosis en la vida del participante. Por favor revise la respuesta." and siga con P B648.

If NDT_SD is equal to 1, then siga con P B650.

B649. De esas veces que tuvo sobredosis, ¿Cuántas veces ocurrieron en México?

__ __ __	veces	
997	No sé	***Siga con P instrucción antes de Q1***
998	Se rehúsa a contestar	***Siga con P instrucción antes de Q1***
999	No corresponde	***Siga con P instrucción antes de Q1***

If B649 is greater than B647 and B649 is not equal to 997 and B649 is not equal to 998 and B649 is not equal to 999 then LEA: "Esa respuesta es mayor que la cantidad de sobredosis en la vida del participante. Por favor revise la respuesta." and siga con P B649.

B650. En los últimos **6 MESES**, ¿cuántas veces ha tenido una sobredosis?

__ __	veces	
97	No sé	*Siga con P instrucción antes de Q1*
98	Se rehúsa a contestar	*Siga con P instrucción antes de Q1*

If B650 is greater than B647 and B650 is not equal to 97 and B650 is not equal to 98 then LEA: "Esa respuesta es mayor que la cantidad de sobredosis en la vida del participante. Por favor revise la respuesta." and siga con P B650.

If B650 is equal to 0, then siga con P instrucción antes de B659.

B651. ¿En qué ciudad tuvo la sobredosis más reciente? (Escoja una)

1	Condado de San Diego
2	Tijuana
3	Otra ciudad en E.E.U.U
4	Otra ciudad en México
5	Otro país (no EUA o México)
7	No sé
8	Se rehúsa a contestar

If B651 is equal to 5 then siga con P B652

If not, then siga con P B653.

B652. Por favor especifique en qué otro país (no EUA o Mexico) tuvo esta sobredosis más reciente

__ __

B653. ¿Qué drogas estaba usando la **última vez** que tuvo una sobredosis? (Marque todas las que apliquen)

__ Alcohol

__ Marihuana

__ Heroína/Goma negra (la negra) sola

__ Heroína en polvo

__ Cocaína crack sola

__ Cocaína sola (en polvo)

__ Heroína y cocaína juntas (speedball)

__ Metanfetamina/cristal sola

__ Metanfetamina/cristal y heroína juntas

__ China White sola

__ China White y heroína juntas

__ China White y fentanilo juntas

__ China White y cristal/metanfetamina juntas

__ PCP

__ Drogas alucinógenas como ácido, LSD, peyote, mezcalina, hongos

__ Inhalantes (por ejemplo, goma, gasolina, diluyente de pintura, agua celeste u otros químicos)

__ Tranquilizantes sin receta (Rivotril, Diazepam, Valium, Ativan o Restoril)

__ OxyContin, Sin receta

__ Otros opioides controlados sin receta (Vicodin, Darvono Percocet)

__ Barbitúricos sin receta (Amytal, Nembutal, o Seconal)

__ Fentanilo and cocaína juntas

__ Fentanilo and heroína juntas

__ Fentanilo and Metanfetaminas/cristal juntas

__ Otra droga o combinación de drogas que no se ha mencionado aún

__ No sé

__ Se rehúsa a contestar

If B653X is equal to 1 then siga con P B654

If not, then siga con P B655.

B654. Por favor especifique qué otra droga o combinación de drogas usó la última vez que tuvo una sobredosis.

__ __

B655. ¿Cuál de las drogas que Ud. uso el dia de su última sobredosis se inyecto? (Marque todas las que apliquen)

__ Heroína negra/Goma negra (la negra) sola

__ Heroína en polvo

__ Cocaína crack sola

__ Cocaína sola (en polvo)

__ Heroína y cocaína juntas (speedball)

__ Metanfetamina/cristal sola

__ Metanfetamina/cristal y heroína juntas

__ China White sola

__ China White y heroína juntas

__ China White y fentanilo juntas

__ China White y cristal/metanfetamina juntas

__ PCP

__ Drogas alucinógenas como ácido, LSD, peyote, mezcalina, hongos

__ Inhalantes (por ejemplo, goma, gasolina, diluyente de pintura, agua celeste u otros químicos)

__ Tranquilizantes sin receta (Rivotril, Diazepam, Valium, Ativan o Restoril)

__ OxyContin, Sin receta

__ Otros opioides controlados sin receta (Vicodin, Darvono Percocet)

__ Barbitúricos sin receta (Amytal, Nembutal, y Seconal)

__ Fentanilo and cocaína juntas

__ Fentanilo and heroína juntas

__ Fentanilo and Metanfetaminas/cristal juntas

__ Fentanilo and china white juntas

__ Otra droga o combinación de drogas que no se ha mencionado aún

__ No sé

__ Se rehúsa a contestar

If B655W is equal to 1 then siga con P B656

If not, then siga con P B657.

B656. Por favor especifique qué otra droga o combinación de drogas se inyectó la última vez que tuvo una sobredosis.

____

B657. En los últimos 30 días antes a la sobredosis que usted acaba de describir ¿había estado en custodia (prison, cárcel, o centro de detención) por 3 días o más?

1 Si

0 No

8 Se rehúsa a contestar

B658. En los últimos 30 días antes a la sobredosis que usted acaba de describir ¿había dejado de usar drogas por 3 días o más porque estaba en tratamiento para su uso de drogas o por otra razón?

1 Si

0 No

8 Se rehúsa a contestar

LEA: LEER: Naoloxona es una medicina que se puede usar para revertir una sobredosis.

B659. **¿Habías escuchado sobre naloxona antes del día de hoy?**
(Escoja una)

1 Si

2 No

3 No estoy seguro-a

8 Se rehúsa a contestar

If B659 is not equal to 1, then siga con P B676.

B660. ¿Alguna vez en su vida ha tenido que usar naloxona en usted mismo? (Escoja una)

1 Si

2 No

3 No estoy seguro-a

8 Se rehúsa a contestar

If B660 is not equal to 1, then siga con P B662.

B661. ¿Cuántas veces le tuvo que usar naloxona en usted mismo en los últimos 6 meses?

__ __ __ veces

998 Se rehúsa a contestar

B662. ¿Alguien vez en su vida más le ha tenido que usar Naloxone en usted? (Escoja una)

1 Si

2 No

3 No estoy seguro-a

8 Se rehúsa a contestar

If B662 is not equal to 1, then siga con P B664.

B663. ¿Cuántas veces alguien más le tuvo que usarle Naloxona a Ud. en los últimos 6 meses?

__ __ __ veces

998 Se rehúsa a contestar

B664. ¿Alguna vez en su vida Ud. le tuvo que user Naloxona a alguien más? (Escoja una)

1 Si

2 No

3 No estoy seguro-a

8 Se rehúsa a contestar

If B664 is not equal to 1, then siga con P B666.

B665. ¿Cuántas veces le tuvo que usar naloxona a alguien más en los últimos 6 meses?

__ __ __	veces
998	Se rehúsa a contestar

B666. ¿Trae con Ud. Naloxona ahora mismo?

1	Si
0	No
8	Se rehúsa a contestar

B667. ¿Dónde consiguió la última naloxona? (Escoja una)

01	Programa de intercambio de Jeringas
02	En Punto
03	PrevenCasa
04	Centro de Salud de la Comunidad
05	Farmacia
06	Centro de rehabilitación de drogas
07	N/C (nunca conseguí Naloxona)
08	Otro
98	Se rehúsa a contestar

If B667 is not equal to 8, then siga con P B669.

B668. Por favor especifique de dónde consiguió la última naloxona

__ __

B669. ¿Cómo pagó por la última naloxona que consiguió? (Escoja una)

1 en dólares estadounidenses

2 en pesos

3 en otra forma de pago

4 no pagó (cero)

8 Se rehúsa a contestar

If B669 is not equal to 1, then siga con P instrucción antes de B671.

B670. ¿Cuánto pagó por la última naloxona en dólares? __ __ __

998 Se rehúsa a contestar

If B669 is not equal to 2, then siga con P instrucción antes de B672.

B671. ¿Cuánto pagó por la última naloxona en pesos? __ __ __ __ __

99998 Se rehúsa a contestar

If B669 is not equal to 3, then siga con P B673.

B672. Por favor especifique otra forma de pago.

__ __

B673. **En los últimos 6 meses, que tan seguido tuviste naloxona contigo donde pudieras obtenerla dentro/en menos de 5 minutos?** (Escoja una)

1 Siempre

2 La mayoría del tiempo

3 A veces

4 Nunca

8 Se rehúsa a contestar

B674. **En los últimos 6 meses, ¿Qué tan seguido te has inyectado alrededor de otras personas que tenían naloxona que te pudieran dar dentro de 5 minutos?** (Escoja una)

1 Siempre

2 La mayoría del tiempo

3 A veces

4 Nunca

8 Se rehúsa a contestar

B675. ¿Sabe a dónde ir para conseguir Naloxona gratis hoy día? (Escoja una)

1 Si

2 No

3 No estoy seguro-a

8 Se rehúsa a contestar

B676. *En los últimos 6 meses, has tenido algún absceso (cuerazo) en una ubicación en tu cuerpo donde te inyectas drogas?*

1 Si

0 No

8 Se rehúsa a contestar

B677. ¿Cuál es el tipo de seguranza o plan de salud principal que tiene Ud. ahora? (Escoja una)

0 No tengo seguranza de salud

1 Privada (contratada directamente o a través de mi trabajo)

2 Pública (Medicare, MediCal, Tricare)

7 No sé

8 Se rehúsa a contestar

B678. Perdió Ud. su seguranza médica a causa de la pandemia del COVID-19?

1 Si

0 No

7 No sé

8 Se rehúsa a contestar

B679. ¿Tiene Ud. una discapacidad que interfiere con su habilidad para realizar tareas cotidianas? Ejemplos de actividades cotidianas incluirían el caminar, subir escaleras, ir de compras, balancear su chequera, bañarse, o vestirse.

1 Si

0 No

8 Se rehúsa a contestar

EXPERIENCIA DE REHABILITACIÓN DE DROGAS

LEA: ENTREVISTADOR: Ahora, le voy a preguntar algunas preguntas sobre el conseguir ayuda para su uso de drogas.

B680. ¿Hasta qué punto diría usted que actualmente necesita ayuda para su uso de drogas? (Escoja una)

0 No necesita ayuda

1 Algo de ayuda

2 Mucha ayuda

3 Ayuda urgente

8 Se rehúsa a contestar

B681. Alguna vez, ¿ha recibido cualquier tipo de ayuda profesional para su uso de alcohol o drogas? Por ayuda me refiero a un centro de rehabilitación, la metadona o el uso de otros medicamentos, o cualquier otro tipo de programa o reunión que le ayuda a reducir o detener su uso de alcohol o drogas.

1 Si

0 No

8 Se rehúsa a contestar

If B681 is not equal to 1, then siga con P instrucción antes de B770.

B682. ¿Para cuál de las siguientes condiciones recibió ayuda profesional? (Escoja una)

1 Drogas solamente

2 Alcohol solamente

3 Los dos, alcohol y drogas

8 No sé

9 Se rehúsa a contestar

If B682 is equal to 1 or B682 is equal to 3 then siga con P B683

If not, then siga con P instrucción antes de B686.

B683. En toda su vida, ¿cuántas veces ha recibido algún tipo de ayuda profesional para su uso de drogas?

__ __	# de veces
97	No sé
98	Se rehúsa a contestar

B684. De esas [Respuesta a P B683] veces ¿cuántas veces recibió ayuda en los Estados Unidos para su uso de drogas?

__ __	# de veces
98	Se rehúsa a contestar

If B684 is greater than B683 and B684 is not equal to 98 then LEA: "Esa respuesta es mayor que el número total de veces que el participante recibió ayuda profesional por consumo de drogas y alcohol." and siga con P B684.

B685. De esas [Respuesta a P B683] veces ¿cuántas veces recibió ayuda en México para su uso de drogas?

__ __	# de veces
98	Se rehúsa a contestar

If B685 is greater than B683 and B685 is not equal to 98 then LEA: "Esa respuesta es mayor que el número total de veces que el participante recibió ayuda profesional por consumo de drogas y alcohol." and siga con P B685.

If B682 is equal to 2 or B682 is equal to 3 then siga con P B686

If not, then siga con P B689.

B686. En toda su vida, ¿cuántas veces ha recibido algún tipo de ayuda profesional por consumir alcohol?

__ __	# de veces
-1	No sé
98	Se rehúsa a contestar

B687. De esas [Respuesta a P B686] veces ¿cuántas veces recibió ayuda en los Estados Unidos para su consumo de alcohol?

__ __	# de veces
98	Se rehúsa a contestar

If B687 is greater than B686 and B687 is not equal to 98 then LEA: "Esa respuesta es mayor que el número total de veces que el participante recibió ayuda profesional por consumo de drogas y alcohol." and siga con P B687.

B688. De esas [Respuesta a P B686] veces ¿cuántas veces recibió ayuda en México para su consumo de alcohol?

__ __	# de veces
98	Se rehúsa a contestar

If B688 is greater than B686 and B688 is not equal to 98 then LEA: "Esa respuesta es mayor que el número total de veces que el participante recibió ayuda profesional por consumo de drogas y alcohol." and siga con P B688.

B689. ¿Alguna vez se ha inscrito en un centro de rehabilitación? Por centro de rehabilitación, me refiero a un lugar a donde fue y pasó la noche para recibir ayuda por sus problemas con las drogas o alcohol.

1	Si
0	No
8	Se rehúsa a contestar

If B689 is not equal to 1, then siga con P B720.

B690. ¿Cuántas veces se ha inscrito en un centro de rehabilitación?

__ __	# de veces
98	Se rehúsa a contestar

If B690 is greater than B683 + B686 and B690 is not equal to 98 then LEA: "Esa respuesta es mayor que el número total de veces que el participante recibió ayuda profesional por consumo de drogas y alcohol." and siga con P B690.

LEA: LEA: LEA: Ahora me gustaría hacerle preguntas sobre sus experiencias en los centros de rehabilitación en los que ha estado.

B691. ¿Cuál es el nombre del centro de rehabilitación en el cual se inscribió más recientemente y pasó la noche?

____

B692. ¿En qué ciudad o ciudades esta ubicado(s) este centro(s) de rehabilitación o tratamiento para drogas? (Escoja una)

1 Condado de San Diego

2 Tijuana

3 Otra ciudad en EUA

4 Otra ciudad en México

5 Otro

8 Se rehúsa a contestar

If B692 is equal to 5 then siga con P B693

If not, then siga con P B694.

B693. Por favor especifica la ciudad donde esta ubicado este centro de tratamiento para el uso de drogas/ rehabilitación.

____

B694. ¿Cuándo fue la última vez que entró a este centro de tratamiento para el uso drogas? (Escoja una)

1 Hace menos de un mes

2 Hace 1-6 meses

3 > 6 meses

8 Se rehúsa a contestar

If B694 is equal to 3, then siga con P B720.

B695. ¿Qué tipo de programa era? (Escoja una)

1 Ayuda mutua o programa de 12 pasos

2 Fe/Basado en Religión

3 Instituto del Gobierno

4 Instituto de Hospital Privado

5 Clínica de Metadona/ Buprenorfina/Suboxone

6 Otro

7 No sé

8 Se rehúsa a contestar

If B695 is equal to 6 then siga con P B696

If not, then siga con P B697.

B696. Por favor especifique el tipo de programa.

____

B697. ¿Había un costo para inscribirse en el centro de tratamiento para el uso de drogas/ rehabilitación?

1 Si

0 No

8 Se rehúsa a contestar

If B697 is not equal to 1, then siga con P B703.

B698. Por favor, seleccione el tipo de moneda que sabe que cuesta el programa de rehabilitación. (Escoja una)

1	Pesos
2	Dólares
8	Se rehúsa a contestar
9	No corresponde

If B698 is equal to 2 then siga con P B699

If not, then siga con P instrucción antes de B701.

B699. ¿Cuál era el costo del programa de rehabilitación en dólares?

__ __ __ __ __	dólares
99998	Se rehúsa a contestar

B700. Por favor especifique el período de pago (diario, semanal, mensual) del costo del programa de rehabilitación. (Escoja una)

1	Por dia
2	Por semana
3	Por mes
8	Se rehúsa a contestar

If B698 is equal to 1 then siga con P B701

If not, then siga con P B703.

B701. ¿Cuál era el costo del programa de rehabilitación en pesos?

__ __ __ __ __	pesos
99998	Se rehúsa a contestar

B702. Por favor especifique el período de pago (diario, semanal, mensual) del costo del programa de rehabilitación. (Escoja una)

1	Por dia
2	Por semana
3	Por mes
8	Se rehúsa a contestar

B703. ¿Sigue inscrito en el programa de tratamiento para drogas?

1	Si
0	No
8	Se rehúsa a contestar

B704. A este centro de rehabilitación más reciente ¿para la rehabilitación de qué drogas fue a pedir ayuda? (Marque todas las que apliquen)

__ Alcohol

__ Marihuana

__ Heroína

__ Crack

__ Cocaína (en polvo)

__ Metanfetamina/cristal

__ China White

__ PCP

__ Drogas alucinógenas como ácido, LSD, peyote, mezcalina, hongos

__ Inhalantes (por ejemplo, goma, gasolina, diluyente de pintura, agua celeste u otros químicos)

__ Tranquilizantes (Rivotril, Diazepam, Valium, Ativan o Restoril)

__ OxyContin, Sin receta

__ Opioides controlados sin receta (Vicodin, Darvono o Percocet)

__ Barbitúricos sin receta (Amytal, Nembutal, o Seconal)

__ Fentanilo (Fentanyl)

__ Otra droga o combinación de drogas que no se ha mencionado aún

__ Se rehúsa a contestar

If B704P is equal to 1 then siga con P B705

If not, then siga con P B706.

B705. Por favor especifique qué otra droga o combinación de drogas fue a pedir ayuda.

____

B706. ¿Cuánto tiempo estuvo en el centro de rehabilitación más reciente?

__ __ __	AñOS
__ __ __	MESES
__ __ __	DíAS
997	No sé (Meses)
998	Se rehúsa a contestar (Meses)

If Trunc(B706/12) is greater than A1 then LEA: "La respuesta debe ser menor que la edad del participante. Por favor revise la respuesta." and siga con P B706.

B707. ¿Fue usted por su propia voluntad al centro de rehabilitación más reciente?

1	Si
0	No
8	Se rehúsa a contestar

If B707 is equal to 0 then siga con P B708

If not, then siga con P instrucción antes de B709.

B708. Quien te hizo ir a este más reciente centro de rehabilitacion? (Marque todas las que apliquen)

__ Padr
__ Pare
__ Hijo
__ Herr
__ Otra
__ Juez
__ Juez
__ Fue
__ Trab
__ Trab
__ Otro
__ Se r

If B708K is not equal to 1, then siga con P instrucción antes de B710.

B709. Por favor especifique qué otra persona lo hizo ir a este centro de rehabilitación

____

If B707 is equal to 1 then siga con P B710

If not, then siga con P instrucción antes de B711.

B710. ¿Cuáles son todas las razones por las que usted decidió inscribirse en el centro de rehabilitación más reciente? (Marque todas las que apliquen)

__ Tuve una muy mala malilla

__ Estaba cansado (a) de usar drogas/quería hacer algo más con mi vida

__ Quería evitar que me arrestaran

__ Me dieron de escoger entre ir a tratamiento o ir a la cárcel, elegí ir a tratamiento

__ Los agentes del orden me forzaron a ir

__ Mi pareja quería que yo fuera (a rehabilitarme)

__ Mi pareja también se estaba limpiando/rehabilitando

__ Estaba embarazada/tuve un bebé

__ Me quitaron a mis hijos (as)

__ Mi familia me animó/motivó

__ Estaba enfermo(a) y necesitaba atención médica

__ Tenía problemas mentales

__ Tuve una sobredosis

__ Razones religiosas

__ Ya no podía pagar las drogas que usaba

__ Otra

__ Se rehúsa a contestar

If B710P is not equal to 1, then siga con P B712.

B711. Por favor especifique qué otra razón (es) tuvo para inscribirse en este centro de rehabilitación más reciente.

____

B712. ¿Recibió medicamentos para la desintoxicación en este centro de rehabilitación más reciente?

1 Si

0 No

8 Se rehúsa a contestar

If B712 is equal to 1 then siga con P B713

If not, then siga con P B715.

B713. ¿Qué medicamento(s) recibió en este centro de rehabilitación más reciente? (Marque todas las que apliquen)

__ Rivotril

__ Darvon

__ Metadona

__ Buprenorfina/Suboxone

__ Naltrexone

__ Modafinilo

__ Otro

__ No sé

__ Se rehúsa a contestar

If B713G is equal to 1 then siga con P B714

If not, then siga con P B715.

B714. Por favor especifique qué otro(s) medicamento(s) recibió.

____

B715. ¿Cuáles fueron los servicios a los que usted tuvo acceso en este centro de rehabilitación más reciente? **Entrevistador: Lea las opciones y marque todas las que apliquen.** (Marque todas las que apliquen)

__ Ayuda mutua o reuniones de 12 pasos

__ Asesoría individual psicológica

__ Asesoría psicológica familiar

__ Asesoría espiritual

__ Prueba del VIH

__ Prueba de ITS

__ Prueba de VHC

__ Prueba de COVID-19

__ Tratamiento médico de un doctor

__ Servicios legales

__ Educación de salud

__ Ropa y otras cosas personales

__ Entrenamiento de trabajo o desarrollo de habilidades

__ Otro

__ Se rehúsa a contestar

If B715N is equal to 1 then siga con P B716

If not, then siga con P B717.

B716. Por favor especifique a qué otros servicios tuvo acceso

____

B717. ¿La última vez que estuvo en el centro de rehabilitación fue maltratado por parte del personal?

1 Si

0 No

8 Se rehúsa a contestar

If B717 is equal to 1 then siga con P B718

If not, then siga con P instrucción antes de B719.

B718. ¿Qué tipo de maltrato ha recibido usted por parte del personal? **(Entrevistador: No lea la lista en voz alta. Marque todas las que el participante diga.)** (Marque todas las que apliquen)

__ Abuso verbal, ser insultado(a)

__ Falta de respeto

__ Abuso mental/psicológico

__ Abuso físico (golpes, puñetazos, patadas)

__ Físicamente restringido (atado a una cama o impedimento del movimiento)

__ Impedir que se vaya, no permitirle salirse del centro

__ Castigo físico por recaer

__ Abuso sexual

__ Se han rehusado a darle la medicina para el síndrome de abstinencia (la malilla)

__ Ud. tuvo que trabajar fuera del programa o hacer servicio, pero no le pagaron

__ Le robaron dinero/propiedad

__ No le permitían tener visitas

__ No tenía suficiente comida/se sentía enfermo con la comida

__ Estaba enfermo, pero no recibió atención médica de un doctor

__ Otro

__ Se rehúsa a contestar

If B718O is equal to 1 then siga con P B719

If not, then siga con P B720.

B719. Por favor especifique qué otro tipo de maltrato recibió Ud

____

EXPERIENCIAS DE METADONA

B720. ¿Alguna vez se ha inscrito en un programa de metadona? **Entrevistador: Si el participante dice: "No sé", LEA: La Metadona es una terapia de sustitución oral, lo que significa que el medicamento se le administra por vía oral para ayudar a lidiar con su adicción.)**

1	Si
0	No
8	Se rehúsa a contestar

If B720 is equal to 0 then siga con P B721

If not, then siga con P instrucción antes de B723.

B721. ¿Por qué nunca se ha inscrito en un programa de metadona? (Escoja una)

01	No funciona
02	No quería que mi familia se diera cuenta de que era adicto
03	Recaí
04	Es muy caro
05	Es mucho problema ir a la clínica todos los días
06	La clínica estaba muy lejos
07	Te da más síntomas abstinencia (malilla)
08	La dosis es muy alta
09	La dosis es muy baja
10	Todavía quiero usar, no le veo propósito
11	Es más adictiva
12	Nadie me la recomendó
13	No sé qué es la metadona
14	Otro
98	Se rehúsa a contestar

If B721 is not equal to 14, then siga con P instrucción antes de B723.

B722. Por favor, especifique otra razon.

— —

If B720 is not equal to 1, then siga con P B745.

B723. ¿Cuantas veces ha iniciado un programa de metadona?
(ENTREVISTADOR: Si el participante dice, "No se" pregúntele por la respuesta más aproximada)

— —

98 Se rehúsa a contestar

If B723 is greater than B683 and B723 is not equal to 98 then LEA: "Esa respuesta es mayor que el número total de veces que el participante recibió ayuda profesional por consumo de drogas y alcohol." and siga con P B723.

B724. ¿Qué edad tenías la primera vez que entraste a un programa de metadona? **(Entrevistador: Si el participante dice: "No sé pídale su mejor cálculo o estimación.)**

__ __	años
98	Se rehúsa a contestar

If B724 is greater than A1 and B724 is not equal to 98 then LEA: "La respuesta es mayor que la edad del participante. Por favor verifique la respuesta." and siga con P B724.

B725. ¿Actualmente se encuentra inscrito en un programa de metadona?

1	Si
0	No
8	Se rehúsa a contestar

B726. ¿Cuándo fue la última vez que entró a este centro de tratamiento para el uso drogas? (Escoja una)

1	Dentro de los últimos 6 meses
2	Hace ya más de 6 meses, pero dentro del último año
3	Hace más de 1 año, pero menos de 3 años
4	Hace 3-5 años
5	Hace más de 5 años
8	Se rehúsa a contestar

B727. ¿En qué ciudad fue este programa de metadona? (Escoja una)

1	Condado de San Diego
2	Tijuana
3	Otra ciudad en EUA
4	Otra ciudad en México
5	Otro
7	No sé
8	Se rehúsa a contestar

If B727 is equal to 5 then siga con P B728

If not, then siga con P B729.

B728. Por favor especifica la ciudad donde se ubica la clinica de metadona

____

B729. Alguna vez, ¿se ha inscrito en un programa de tratamiento con metadona por orden de un agente del orden o un juez?

1	Si
0	No
8	Se rehúsa a contestar

If B729 is not equal to 1, then siga con P B732.

B730. ¿En qué ciudad fue este programa de metadona? (Marque todas las que apliquen)

__	Condado de San Diego
__	Tijuana
__	Otra ciudad en EUA
__	Otra ciudad en México
__	Otro
__	Se rehúsa a contestar

If B730E is equal to 1 then siga con P B731

If not, then siga con P B732.

B731. Por favor especifica la ciudad donde se ubica la clinica/el programa de metadona

__ __

B732. Por favor, seleccione el tipo de moneda que sabe que cuesta el programa de metadona. (Escoja una)

1	Pesos
2	Dólares
8	Se rehúsa a contestar
9	No corresponde

If B732 is equal to 2 then siga con P B733

If not, then siga con P instrucción antes de B735.

B733. ¿Cuál fue el costo del programa de metadona? __ __ __ __ __ dólares

99998	Se rehúsa a contestar

B734. Por favor especifique el período de pago (diario, semanal, mensual) del costo del programa de metadona. (Escoja una)

1	Por dia
2	Por semana
3	Por mes
8	Se rehúsa a contestar

If B732 is equal to 1 then siga con P B735

If not, then siga con P instrucción antes de B737.

B735. ¿Cuál fue el costo del programa de metadona? __ __ __ __ __ pesos

99998	Se rehúsa a contestar

B736. Por favor especifique el período de pago (diario, semanal, mensual) del costo del programa de metadona. (Escoja una)

1	por dia
2	por semana
3	por mes
8	Se rehúsa a contestar

If B725 is equal to 1 then siga con P B737

If not, then siga con P instrucción antes de B738.

B737. ¿Todavía está usando metadona?

1	Si
0	No
8	Se rehúsa a contestar

If B737 is equal to 1 then siga con P B738

If not, then siga con P instrucción antes de B740.

B738. ¿Cuál fue la dosis con la que lo/la iniciaron? __ __ __ mg

998 Se rehúsa a contestar

B739. ¿Cuál es tu dosis actualmente? __ __ __ mg

998 Se rehúsa a contestar

If B737 is equal to 0 then siga con P B740

If not, then siga con P B742.

B740. ¿Cuáles son todas las razones por las que usted dejó de usar la metadona? **(Entrevistador: <u>No lea la lista en voz alta.)</u>** (Marque todas las que apliquen)

__ No funcionó

__ Recaí

__ Era muy caro

__ Era muy complicado ir a la clínica todos los días

__ Me sentí en peligro usando la clínica

__ La clínica quedaba muy retirada, lejos

__ Me daba peores síntomas de abstinencia (malilla) que la heroína/ goma negra/ la negra

__ La dosis era muy alta

__ La dosis era muy baja

__ No me caía bien el personal de la clínica

__ Todavía estaba usando otras drogas y la verdad que ya no le veía sentido a seguir yendo

__ Es más adictiva que la heroína de goma/heroína

__ Me la confisco la policía

__ Tengo temor de ser molestado por la policía por tomarla

__ Otra

__ Se rehúsa a contestar

If B740O is equal to 1 then siga con P B741

If not, then siga con P B742.

B741. Por favor especifique la(s) otra razón(es) por las que dejó de usar metadona

____

B742. ¿Cuánto tiempo duro inscrito en el programa de metadona esta ultima vez?

__ __ __	MESES
__ __ __	SEMANAS
__ __ __	DíAS
998	Se rehúsa a contestar (Meses)

If Trunc(B742/12) is greater than A1 then LEA: "La respuesta es mayor que la edad del participante. Por favor verifique la respuesta." and siga con P B742.

If B723 is greater than 1 and B723 is not equal to 98 then siga con P B743

If not, then siga con P B744.

B743. ¿Cuál es el período más largo que ha estado inscrito en un programa de metadona?

__ __ __	MESES
__ __ __	SEMANAS
__ __ __	DíAS
998	Se rehúsa a contestar (Meses)

If Trunc(B743/12) is greater than A1 then LEA: "La respuesta es mayor que la edad del participante. Por favor verifique la respuesta." and siga con P B743.

B744. ¿Qué le pareció la dosis de metadona que le dieron? (Escoja una)

1	Era muy alta
2	Era muy baja
3	Era la cantidad precisa
7	No sé
8	Se rehúsa a contestar

EXPERIENCIAS CON BUPRENORFINA

B745. ¿Alguna vez ha estado inscrito en un programa de Buprenorfina? **(Entrevistador: Si el participante dice: "No sé", LEA: La buprenorfina es una terapia de sustitución oral similar a la metadona, lo que significa que el medicamento se le administra por vía oral para ayudar a lidiar con su adicción.)**

1 Si

0 No

8 Se rehúsa a contestar

If B745 is equal to 0 then siga con P B746

If not, then siga con P instrucción antes de B748.

B746. ¿Por qué no se ha inscrito en un programa de Buprenorfina/Saboxone? (Escoja una)

01 No funciona

02 No quería que mi familia se diera cuenta de que era adicto

03 Recaí

04 Es muy caro

05 Es mucho problema ir a la clínica todos los días

06 La clínica estaba muy lejos

07 Te dá más síntomas de abstinencia (malilla)

08 La dosis es muy alta

09 La dosis es muy baja

10 Todavía quiero usar (drogas), no le veo propósito

11 Es más adictiva

12 Nadie me la recomendó

13 No sé qué es la Buprenorfina/Saboxone

14 Otro

98 Se rehúsa a contestar

If B746 is not equal to 14, then siga con P instrucción antes de B748.

B747. Por favor especifique la(s) otra(s) razón(es) por las que dejó de usar Buprenorfina /Saboxone

— —

If B745 is not equal to 1, then siga con P instrucción antes de B770.

B748. ¿Cuántas veces en tu vida has estado en un programa de Buprenorfina/Saboxone?
(Entrevistador: Si el participante dice: "No sé pídale su mejor cálculo o estimación.)

__ __ __ veces

-01 Se rehúsa a contestar

If B748 is greater than B683 and B748 is not equal to 98 then LEA: "Esa respuesta es mayor que el número total de veces que el participante recibió ayuda profesional por consumo de drogas y alcohol." and siga con P B748.

B749. ¿Qué edad tenías la primera vez que usaste Buprenorfina/Saboxone? **(Entrevistador: Si el participante dice: "No sé pídale su mejor cálculo o estimación.)**

__ __	veces
98	Se rehúsa a contestar

If B749 is greater than A1 and B749 is not equal to 98 then LEA: "Esa respuesta supera la edad actual del participante. Por favor revise la respuesta." and siga con P B749.

B750. ¿Está inscrito en un programa de Buprenorfina-Suboxona actualmente?

1	Si
0	No
8	Se rehúsa a contestar

B751. ¿Cuándo fue la última vez que se inscribió en un programa de Buprenorfina/Suboxona? **Entrevistador: Si el participante dice No Sé, pídale que le dé su mejor estimación** (Escoja una)

1	Dentro de los últimos 6 meses
2	Hace ya más de 6 meses, pero dentro del último año
3	Hace más de 1 año, pero menos de 3 años
4	Hace 3-5 años
5	Hace más de 5 años
7	No sé
8	Se rehúsa a contestar

B752. ¿En qué ciudad se ubicaba este programa de Buprenorfina/Saboxone? (Escoja una)

1	Condado de San Diego
2	Tijuana
3	Otra ciudad en EUA
4	Otra ciudad en México
5	Otro
8	Se rehúsa a contestar

If B752 is equal to 5 then siga con P B753

If not, then siga con P B754.

B753. Por favor especifique en qué otra ciudad se ubicaba el programa de buprenorfina-Suboxona

____

B754. ¿Alguna vez se ha inscrito en un programa de tratamiento con Buprenorfina/Saboxone por orden de un agente del orden o un juez?

1	Si
0	No
8	Se rehúsa a contestar

If B754 is not equal to 1, then siga con P B757.

B755. ¿En qué ciudad se ubicaba este programa de Buprenorfina/Saboxone ordenado de un agente del orden o un juez? (Marque todas las que apliquen)

__ Condado de San Diego

__ Tijuana

__ Otra ciudad en EUA

__ Otra ciudad en México

__ Otro

__ Se rehúsa a contestar

If B755E is equal to 1 then siga con P B756

If not, then siga con P B757.

B756. Por favor especifique en qué otra ciudad se ubicaba el programa de buprenorfina-Suboxona

__ __

B757. Por favor especificque el tipo de moneda en que costaba el programa de Buprenorfina-Suboxona. (Escoja una)

1 Pesos Mexicanos

2 Dólar americano

8 Se rehúsa a contestar

9 No corresponde

If B757 is equal to 2 then siga con P B758

If not, then siga con P instrucción antes de B760.

B758. ¿Cuál fue el costo del programa de Buprenorfina/Suboxona (en dólar americano)?

__ __ __ __ __

99998 Se rehúsa a contestar

B759. Por favor especifique el período de pago (diario, semanal, mensual) del costo del programa de Buprenorfina/Suboxona (en dólar americano). (Escoja una)

1	Por día
2	Por semana
3	Por mes
8	Se rehúsa a contestar

If B757 is equal to 1 then siga con P B760

If not, then siga con P instrucción antes de B762.

B760. ¿Cuál fue el costo del programa de Buprenorfina/Suboxona (en Pesos Mexicanos)?

_ _ _ _ _

99998	Se rehúsa a contestar

B761. Por favor especifique el período de pago (diario, semanal, mensual) del costo del programa de Buprenorfina/Suboxona (en Pesos Mexicanos). (Escoja una)

1	Por día
2	Por semana
3	Por mes
8	Se rehúsa a contestar

If B750 is equal to 1 then siga con P B762

If not, then siga con P instrucción antes de B763.

B762. ¿Todavía está usando Buprenorfina?

1	Si
0	No
8	Se rehúsa a contestar

If B762 is equal to 1 then siga con P B763

If not, then siga con P instrucción antes de B765.

B763. ¿Cuál fue la dosis con la que lo/la iniciaron? __ __ __ mg

998 Se rehúsa a contestar

B764. ¿Cuál es tu dosis actualmente? __ __ __ mg

998 Se rehúsa a contestar

If B762 is equal to 0 then siga con P B765

If not, then siga con P B767.

B765. ¿Cuáles son todas las razones por las que usted dejó de usar Buprenorfina/Suboxone? **(Entrevistador: No lea la lista en voz alta. Marque todas las que el participante diga.)** (Marque todas las que apliquen)

__ No funcionó

__ Recaí

__ Era muy caro

__ Era muy complicado ir a la clínica todos los días

__ Me sentí en peligro usando la clínica

__ La clínica quedaba muy retirada, lejos

__ Me daba peores síntomas de abstinencia (malilla) que la heroína

__ La dosis era muy alta

__ La dosis era muy baja

__ No me caía bien el personal de la clínica

__ Todavía estaba usando otras drogas y la verdad que ya no le veía sentido a seguir yen

__ Otra razón

__ Se rehúsa a contestar

If B765L is equal to 1 then siga con P B766

If not, then siga con P B767.

B766. Por favor especifique la otra razón por la que ha dejado su programa de Buprenorfina-Suboxona.

__ __

B767. ¿Cuánto tiempo estuvo inscrito en el programa de Buprenorfina/Suboxona la última vez?

__ __ __	MESES
__ __ __	SEMANAS
__ __ __	DíAS
998	Se rehúsa a contestar (Meses)

If Trunc(B767/12) is greater than A1 then LEA: "Esa respuesta supera la edad actual del participante. Por favor revise la respuesta." and siga con P B767.

B768. ¿Cuál es el período más largo de tiempo que ha estado inscrito en un programa de Buprenorfina/Suboxone?

__ __ __	MESES
__ __ __	SEMANAS
__ __ __	DíAS
998	Se rehúsa a contestar (Meses)

If Trunc(B768/12) is greater than A1 then LEA: "Esa respuesta supera la edad actual del participante. Por favor revise la respuesta." and siga con P B768.

B769. ¿Qué le pareció la dosis de Buprenorfina/Suboxone que le dieron? (Escoja una)

1	Era muy alta
2	Era muy baja
3	Era la cantidad precisa
7	No sé
8	Se rehúsa a contestar

If B681 is not equal to 1 then siga con P B770

If not, then siga con P instrucción antes de B772.

B770. Por favor indique todas las razones por qué no se ha inscrito en un programa de Buprenorfina/Saboxone. (Marque todas las que apliquen)

__ Por miedo a la malilla

__ Costo/Económico

__ No tenía quien me cuidara los niños

__ Esposo o pareja no quiere que el/la participante vaya o cause problemas en la relación

__ No reunió los requisitos para ser ingresado

__ No le gustan los centros

__ No le gustan los regímenes de tratamiento

__ Demasiadas reglas/restricciones de los centros de rehabilitación

__ No le gusta sentirse encerrado

__ Problemas de trabajo (como el pedir permiso en el trabajo)

__ Demasiado lejos para ir/no estaba cerca

__ No sabía a dónde tenía que ir

__ Tenía temor que lo fueran a maltratar los empleados del centro

__ Tenía temor que otros se enteraran que el participante estaba en tratamiento

__ No tiene la fuerza de voluntad para dejarla (la droga)

__ El uso de drogas no es el problema

__ Intenté un programa de rehabilitación antes y no funcionó

__ No cree que la rehabilitación funcione

__ Puedo reducir o dejar de usar drogas por mi cuenta

__ No estoy listo para dejar de usar drogas

__ Todavía no he pensado en ir a rehabilitación

__ Estoy feliz con mi estilo de vida actual

__ Miedo por su seguridad al acudir al programa

__ Miedo de ser molestado por la policía cuando acuda al programa

__ Se rehúsa a contestar

B771. Tiene alguna otra razón por la que no ha recibido ningún tipo de ayuda o tratamiento para su uso de drogas.

1 Si

0 No

8 Se rehúsa a contestar

If B771 is equal to 1 then siga con P B772

If not, then siga con P instrucción antes de B773.

B772. Por favor especifique otra(s) razón(es) por la(s) que no ha buscado ayuda o tratamiento para su uso de drogas.

____

If B725 is equal to 1, then siga con P instrucción antes de B774.

B773. ¿Qué tan probable es que Ud. inicie un programa de metadona dentro de los próximos 6 meses?
ENTREVISTADOR: Si el participante dice que No Sabe, lea lo siguiente: La Metadona es una terapia de reemplazo oral, lo que quiere decir que es una medicina que se toma para lidiar con su adicción. (Escoja una)

1 Muy Improbable

2 Poco probable

3 Probable

4 Muy Probable

8 Se rehúsa a contestar

If B750 is equal to 1, then siga con P instrucción antes de B775.

B774. ¿Qué tan probable es que Ud. inicie un programa de Buprenorfina/Suboxone dentro de los próximos 6 meses?
ENTREVISTADOR: Si el participante dice que No Sabe, lea lo siguiente: La Buprenorfina/Suboxone es una terapia de reemplazo oral similar a la metadona, lo que quiere decir que es una medicina que se le dá para lidiar con su adicción.
(Escoja una)

1 Muy Improbable

2 Poco probable

3 Probable

4 Muy Probable

8 Se rehúsa a contestar

ENCUENTROS CON LAS FUERZAS DEL ORDEN

LEA: ENTREVISTADOR LEA AL PARTICIPANTE: Las siguientes preguntas se refieren a las leyes vigentes y las experiencias con la justicia criminal. Por favor responda según su mejor conocimiento de las leyes, mas allá de lo que Ud. opine sobre si la policía cumple con estas leyes

If NDT_SD is equal to 1 or DT_SD is equal to 1 then siga con P B775

If not, then siga con P instrucción antes de B776.

B775. En tu opinión actualmente en San Diego ¿es legal traer jeringas nuevas, que no han sido usadas?

1	Si
0	No
7	No sé
8	Se rehúsa a contestar

If NDT_TJ is equal to 1 or DT_SD is equal to 1 then siga con P B776

If not, then siga con P instrucción antes de B777.

B776. En tu opinión actualmente en Tijuana ¿es legal traer jeringas nuevas, que no han sido usadas?

1	Si
0	No
7	No sé
8	Se rehúsa a contestar

LEA: ENTREVISTADOR LEA: Ahora me gustaría hacerle algunas preguntas acerca de los encuentros que ha tenido con agentes del orden. Cuando digo los agentes del orden, este término incluye <u>todo tipo de policías municipales, estatales o federales, u oficiales del ejército, investigadores o procuradores.</u> Esta sección es sólo acerca de las interacciones con los agentes del orden en México o en los Estados Unidos. Sé que algunas de estas preguntas pueden ser algo incomodas para responder, pero la entrevista es confidencial, y nosotros no daremos ningún tipo de información a nadie fuera de este proyecto. Nada de lo que nos digas puede ser ligado a ti como individuo.

B777. ¿Alguna vez ha sido parado por agentes del orden? Por esto, nos referimos a alguna vez que fue parado por agentes del orden por ejemplo que lo pararon y lo dejaron ir o que lo pararon y arrestaron.

1 Si

0 No

8 Se rehúsa a contestar

If B777 is not equal to 1, then siga con P B797.

B778. Durante los últimos 6 meses, ¿ha sido parado por agentes del orden? Por esto, nos referimos a alguna vez que fue parado por agentes del orden por ejemplo que lo pararon y lo dejaron ir o que lo pararon y arrestaron.

1 Si

0 No

8 Se rehúsa a contestar

If B778 is not equal to 1, then siga con P B797.

B779. En los últimos 6 meses, ¿en que ciudad estuviste cuando te pararon los agentes del orden? (Marque todas las que apliquen)

__ Condado de San Diego

__ Tijuana

__ Otra ciudad en EUA

__ Otra ciudad en México

__ Otro

__ Se rehúsa a contestar

If B779E is equal to 1 then siga con P B780

If not, then siga con P B781.

B780. Por favor especifique en qué otra ciudad te pararon los agentes del orden en los últimos 6 meses

__ __

B781. Durante los últimos 6 meses, cuando fue parado por agentes del orden, ¿qué fue lo que le sucedió? (Marque todas las que apliquen)

__ Lo pararon y lo arrestaron

__ Lo pararon y lo dejaron ir

__ No sé

__ Se rehúsa a contestar

If B781B is equal to 1 then siga con P instrucción antes de B782

If not, then siga con P B790.

LEA: LEA: Las siguientes preguntas se refieren a las ocasiones que fuiste parado pero NO arrestado por un agente del orden en los últimos 6 meses.

B782. ¿Cuántas veces ha sido parado (pero no arrestado) por agentes del orden en los últimos 6 meses? **Entrevistador: si el participante tiene dificultad para calcular la respuesta para el período de 6 meses, por favor, ayúdelo mediante una estimación de la frecuencia de eventos (veces por semana o mes); y luego calcule una estimación basada en la frecuencia de los encuentros por mes o por semana (por ejemplo, una vez por semana multiplicado por 26 semanas = 26 veces, o dos veces al mes, multiplicado por 6 meses = 12 veces)]**

__ __ __ # de veces

998 Se rehúsa a contestar *Siga con P instrucción antes de Q1*

If B782 is equal to 998 then siga con P instrucción antes de B783

If not, then siga con P instrucción antes de B784.

LEA: ENTREVISTADOR LEA SI EL PARTICIPANTE SE REHUSA A RESPONDER ESTA PREGUNTA: Yo sé que algunas de estas preguntas pueden ser un poco incomodas para ti para responder, pero la entrevista es confidencial, y nosotros no compartiremos ningún tipo de información personal con nadie fuera de este proyecto. Nada de lo que nos digas puede ser ligado a ti como individuo.

B783. ¿Cuántas veces ha sido parado (pero no arrestado) por agentes del orden en los últimos 6 meses? **Entrevistador: si el participante tiene dificultad para calcular la respuesta para el período de 6 meses, por favor, ayúdelo mediante una estimación de la frecuencia de eventos (veces por semana o mes); y luego calcule una estimación basada en la frecuencia de los encuentros por mes o por semana (por ejemplo, una vez por semana multiplicado por 26 semanas = 26 veces, o dos veces al mes, multiplicado por 6 meses = 12 veces)]**

__ __ __ # de veces

998 Se rehúsa a contestar *Siga con P instrucción antes de Q1*

STOPPED = If(B782 is not equal to 998, B782, If(B782 is equal to 998, B783, 0))

If B782 is equal to 0 or B783 is equal to 0 or B783 is equal to 998, then siga con P B790.

B784. ¿En qué ciudad estabas cuando fuiste parado (no arrestado) por agentes del orden en los último 6 meses? (Marque todas las que apliquen)

__ Condado de San Diego

__ Tijuana

__ Otra ciudad en EUA

__ Otra ciudad en México

__ Otro

__ Se rehúsa a contestar

If B784E is equal to 1 then siga con P B785

If not, then siga con P B786.

B785. Por favor especifique en qué otra ciudad te pararon pero no te arrestaron los agentes del orden en los últimos 6 meses

__ __

B786. De las **[STOPPED]** veces que usted fue parado por agentes del orden en los últimos 6 meses, ¿cuántas veces un agente del orden le exigió dinero como una forma de soborno (mordida)?

__ __ __ veces

998 Se rehúsa a contestar

If B786 is greater than STOPPED and B786 is not equal to 998 then LEA: "Need translation" and siga con P B786.

B787. De las **[STOPPED]** veces que usted fue parado por agentes del orden en los últimos 6 meses, ¿cuántas veces un agente del orden le confiscó y nunca le regresó dinero u otras cosas de valor?

__ __ __	veces
998	Se rehúsa a contestar

If B787 is greater than STOPPED and B787 is not equal to 998 then LEA: "La respuesta del participante debe ser menor o igual al número de veces que fue detenido por la policía en los últimos 6 meses. Por favor revise las respuestas." and siga con P B787.

B788. De las **[STOPPED]** veces que usted fue parado por agentes del orden en los últimos 6 meses, ¿cuántas veces un agente del orden le confiscó sus jeringas limpias, sin arrestarlo?

__ __ __	veces
998	Se rehúsa a contestar

If B788 is greater than STOPPED and B788 is not equal to 998 then LEA: "La respuesta del participante debe ser menor o igual al número de veces que fue detenido por la policía en los últimos 6 meses. Por favor revise las respuestas." and siga con P B788.

B789. De las **[STOPPED]** veces que usted fue parado por agentes del orden en los últimos 6 meses, ¿cuántas veces un agente del orden le confiscó sus jeringas usadas sin arrestarlo?

__ __ __	veces
998	Se rehúsa a contestar

If B789 is greater than STOPPED and B789 is not equal to 998 then LEA: "La respuesta del participante debe ser menor o igual al número de veces que fue detenido por la policía en los últimos 6 meses. Por favor revise las respuestas." and siga con P B789.

B790. ¿Alguna vez has sido arrestado/a por agentes del orden? **Por arresto me refiero a experiencias cuando la policía te ha llevado a la estación de policía y hubo un reporte formal hecho sobre este evento.**

1	Si
0	No
8	Se rehúsa a contestar

If B790 is not equal to 1, then siga con P B797.

B791. ¿En qué ciudad fuiste arrestado/a por agentes del orden? (Marque todas las que apliquen)

__ Condado de San Diego

__ Tijuana

__ Otra ciudad en EUA

__ Otra ciudad en México

__ Otro

__ Se rehúsa a contestar

If B791E is equal to 1 then siga con P B792

If not, then siga con P instrucción antes de B793.

B792. Por favor especifique en qué otra ciudad te arrestaron los agentes del orden.

__ __

LEA: ENTREVISTADOR LEA AL PARTICIPANTE: Ahora le voy a preguntar especificamente sobre las experiencias de arrestos que haya tenido.

B793. ¿Cuántas veces ha sido arrestado por agentes del orden en los últimos 6 meses? **[Entrevistador: si el participante tiene dificultad para calcular la respuesta para el período de 6 meses, por favor, ayúdelo mediante una estimación de la frecuencia de eventos (veces por semana o mes), y luego calcule una estimación basada en la frecuencia de los encuentros por mes o por semana (por ejemplo, una vez por semana multiplicado por 26 semanas = 26 veces, o dos veces al mes, multiplicado por 6 meses = 12 veces)]**

__ __ __ Veces

998 Se rehúsa a contestar *Siga con P instrucción antes de Q1*

If B793 is equal to 0 or B793 is equal to 998, then siga con P B797.

B794. ¿En qué ciudad estabas cuando fuiste arrestado por agentes del orden en los últimos 6 meses? (Marque todas las que apliquen)

__	Condado de San Diego
__	Tijuana
__	Otra ciudad en EUA
__	Otra ciudad en México
__	Otro
__	Se rehúsa a contestar

If B794E is equal to 1 then siga con P B795

If not, then siga con P B796.

B795. Por favor especifique en qué otra ciudad te arrestaron los agentes del orden en los últimos 6 meses

__ __

B796. De las [Respuesta a P B793] veces que fuiste arrestado en los últimos 6 meses, ¿cuantas veces te sentenciaron a tiempo en la cárcel?

__ __ __	Veces
997	No sé
998	Se rehúsa a contestar

If B796 is greater than B793 and B796 is not equal to 997 and B796 is not equal to 998 then LEA: "La respuesta del participante debe ser menor o igual al número de veces que fue arrestado en los últimos 6 meses. Por favor revise las respuestas." and siga con P B796.

B797. En los últimos 6 meses ¿cuántas veces ha sido físicamente golpeado (golpe, puñetazo, patada) por quién sea?

__ __ __	# de veces
998	Se rehúsa a contestar

If B797 is equal to 0 or B797 is equal to 998, then siga con P instrucción antes de B802.

B798. De las [Respuesta a P B797] veces en los últimos 6 meses que fue golpeado, ¿cuántas veces fue golpeado por agentes del orden?

__ __ __ # de veces

998 Se rehúsa a contestar

If B798 is greater than B797 and B798 is not equal to 998 then LEA: "La respuesta del participante debe ser menor o igual al número de veces que alguien lo golpeó físicamente (golpeó, golpeó o pateó) en los últimos 6 meses. Por favor revise las respuestas." and siga con P B798.

If B798 is greater than 0 and B798 is not equal to 998 then siga con P B799

If not, then siga con P B801.

B799. ¿En qué ciudad lo golpearon físicamente (golpe, puñetazo, patada) los agentes del orden? (Marque todas las que apliquen)

__ Condado de San Diego

__ Tijuana

__ Otra ciudad en EUA

__ Otra ciudad en México

__ Otro

__ Se rehúsa a contestar

If B799E is equal to 1 then siga con P B800

If not, then siga con P B801.

B800. Por favor especifique en qué otra ciudad lo golpearon físicamente los agentes del orden en los últimos 6 meses

__ __

B801. De las [Respuesta a P B797] veces en los últimos 6 meses en que fue golpeado, ¿cuántas veces fue golpeado por su pareja íntima?

__ __ __ # de veces

998 Se rehúsa a contestar

If B801 is greater than B797 and B801 is not equal to 998 then LEA: "La respuesta del participante debe ser menor o igual al número de veces que fue golpeado en los últimos 6 meses. Por favor revise las respuestas." and siga con P B801.

SECCIÓN D: ACTITUDES

LEA: LEA: El siguiente grupo de preguntas le pedirá su opinión sobre una serie de escenarios diferentes. Me gustaría saber qué tan probable cree que es que estas cosas le puedan pasar en el futuro.

B802. ¿Qué tan probable piensa que sea qué usted pueda ser arrestado en [LCOUNTRY] por posesión de drogas si lo/a para la policía cuando lleva drogas? (Escoja una)

1 Muy improbable

2 Un poco improbable

3 Un poco probable

4 Muy probable

8 Se rehúsa a contestar

B803. ¿Qué tan probable piensa que es que usted pueda ser arrestado en [LCOUNTRY] por posesión de parafernalia si lo/la para la policía cuando lleva jeringas nuevas, sin usar? (Escoja una)

1 Muy improbable

2 Un poco improbable

3 Un poco probable

4 Muy probable

8 Se rehúsa a contestar

B804. ¿Qué tan probable piensa que es que usted pueda ser arrestado en [LCOUNTRY] por posesión de parafernalia si lo/la para la policía cuando lleva jeringas usadas? (Escoja una)

1 Muy improbable

2 Un poco improbable

3 Un poco probable

4 Muy probable

8 Se rehúsa a contestar

B805. ¿Qué tan probable piensa que es que la policía le exija un soborno (mordida) en [LCOUNTRY] si lo/la para cuando lleva jeringas nuevas que no han sido usadas? (Escoja una)

1 Muy improbable
2 Un poco improbable
3 Un poco probable
4 Muy probable
8 Se rehúsa a contestar

B806. ¿Qué tan probable piensa que es que la policía le exija un soborno (mordida) en [LCOUNTRY] si lo/la para cuando lleva jeringas usadas? (Escoja una)

1 Muy improbable
2 Un poco improbable
3 Un poco probable
4 Muy probable
8 Se rehúsa a contestar

B807. ¿Qué tan probable piensa que es que sea arrestado en [LCOUNTRY] sólo porque parece un consumidor de drogas? (Escoja una)

1 Muy improbable
2 Un poco improbable
3 Un poco probable
4 Muy probable
8 Se rehúsa a contestar

Experiencias De Encarcelamiento

LEA: LEER: Ahora te voy a preguntar algunas preguntas sobre tus experiencias con prisiones y cárceles. Para las siguientes preguntas, prisiones se refieren a instituciones a nivel federal y cárceles se refiere a instituciones estatales o del condado (no a nivel municipal) donde te detienen con o sin ser sentenciado (en México Centros de Readaptación Social Penitenciaria La Mesa o El Hongo y en EUA The Federal Bureau of Prisions clasifica las prisiones en varias categorías: Penitenciaria de EUA, Instituciones correccionales federales, instituciones correccionales privadas, campamentos de prisión federal, instalaciones administrativa, complejos de correccionales federales.)

B808. ¿Has estado alguna vez en tu vida en la cárcel/prisión?

1	Si
0	No
8	Se rehúsa a contestar

If B808 is not equal to 1, then siga con P B812.

B809. Desde que empezaste a usar drogas, ¿cuántas veces estuviste en la cárcel/prisión?

__ __ __	veces
998	Se rehúsa a contestar

If B809 is equal to 0 or B809 is equal to 998, then siga con P B811.

B810. Desde que tú empezaste a inyectarte, ¿cuál es el total del tiempo que has pasado en la cárcel/prisión?

__ __	AñOS
__ __	MESES
__ __	DíAS
98	Se rehúsa a contestar (Años)
99	No corresponde (Años)

B811. ¿Estuvo usted encarcelado o en prisión la primera vez que usted se inyectó drogas?

1	Si
0	No
8	Se rehúsa a contestar

B812. ¿Alguna vez en su vida estuvo en la cárcel?

1 Si

0 No

8 Se rehúsa a contestar

If B812 is not equal to 1, then siga con P instrucción antes de B816.

B813. De que empezaste a inyectarte drogas, ¿Cuántas veces has estado en la cárcel?

__ __ __ veces

998 Se rehúsa a contestar

If B813 is equal to 0 or B813 is equal to 998, then siga con P B815.

B814. De que empezaste a inyectarte drogas, ¿Cuál es el tiempo total que has pasado en la cárcel

__ __ AñOS

__ __ MESES

__ __ DíAS

98 Se rehúsa a contestar (Años)

99 No corresponde (Años)

B815. ¿Estuvo usted en la cárcel la primera vez que usted se inyecto drogas?

1 Si

0 No

8 Se rehúsa a contestar

If B811 is equal to 1 and B815 is equal to 1 then LEA: "El participante indicó que la primera vez que el participante inyectado fue en prisión. Ahora el participante indicó que la primera vez estuvo en la cárcel. Por favor, revise en consecuencia." and siga con P B815.

If B808 is not equal to 1 and B812 is not equal to 1, then siga con P instrucción antes de B837.

LEA: ENTREVISTADOR LEA: Ahora te voy a preguntar sobre tu experiencia en prisión en los últimos 6 meses.

B816. En los últimos 6 MESES, ¿cuantas veces ha estado en prisión/cárcel?

__ __ __ veces

998 Se rehúsa a contestar

If B816 is equal to 0 or B816 is equal to 998, then siga con P instrucción antes de B837.

B817. ¿En qué ciudad estuviste en la cárcel en los últimos 6 meses? (Marque todas las que apliquen)

__ Condado de San Diego

__ Tijuana

__ Otra ciudad en EUA

__ Otra ciudad en México

__ Otro

__ Se rehúsa a contestar

If B817E is equal to 1 then siga con P B818

If not, then siga con P B819.

B818. Por favor especifique en qué otra ciudad estaba ubicada esta cárcel

__ __

B819. En los últimos 6 meses, ¿cuánto tiempo en total ha estado en la cárcel/prisión

__ __ AñOS

__ __ MESES

__ __ DíAS

98 Se rehúsa a contestar (Años)

LEA: LEA: Ahora le voy a preguntar sobre su uso de drogas la última vez que estuvo en prisión/cárcel, en los últimos 6 meses.

B820. ¿En que ciudad estaba la cárcel/prisión en la que estuviste la última vez? (Escoja una)

1 Condado de San Diego

2 Tijuana

3 Otra ciudad en EUA

4 Otra ciudad en México

5 Otro

8 Se rehúsa a contestar

If B820 is equal to 5 then siga con P B821

If not, then siga con P B822.

B821. Por favor especifique en qué otra ciudad estaba ubicada esta cárcel más reciente

____

B822. ¿Cuál es el nombre de la prisión/cárcel en donde estuvo recluído? (Escoja una)

1 Mínima seguridad de Otay Mesa

2 Las Colinas

3 Donovan

4 La Mesa

5 El Hongo

6 Otra

7 No sé

8 Se rehúsa a contestar

If B822 is equal to 6 then siga con P B823

If not, then siga con P B824.

B823. Por favor nos puede dar el nombre de la otra prisión /cárcel

__ __

B824. La última vez que estuviste en la cárcel/prisión, ¿usaste drogas (de cualquier tipo)?

1 Si

0 No

8 Se rehúsa a contestar

B825. La última vez que estuviste en la cárcel/prisión, ¿recibiste metadona, buprenorfina o suboxone?

1 Si

0 No

8 Se rehúsa a contestar

If B825 is equal to 1 and B824 is equal to 0 then siga con P B826

If not, then siga con P instrucción antes de B827.

B826. ¿Por cuánto tiempo estuviste en tratamiento? __ __ __ MESES

__ __ __ SEMANAS

__ __ __ DíAS

998 Se rehúsa a contestar (Meses)

If B824 is not equal to 1, then siga con P instrucción antes de B836.

B827. ¿Qué drogas usaste mientras estabas en la cárcel/prisión? (Marque todas las que apliquen)

__ Alcohol

__ Marijuana

__ Heroína sola

__ Cocaína crack sola

__ Cocaína en polvo sola

__ Heroína y cocaína juntas (speedball)

__ Metanfetamina/cristal sola

__ Metanfetamina/cristal y heroína juntas

__ China white sola

__ China white y heroína juntas

__ PCP

__ China white y cristal/metanfetamina juntas

__ Drogas halucinógenas como ácido, LSD, peyote, mezcalina

__ Inhalantes (pegamento, gasolina, tinner de pintura)

__ Tranquilizantes sin receta (Rivotril, diazepan, ativan)

__ Oxycontin sin receta

__ Otros opioides controlados sin receta (vicodin, darvon, percocet)

__ Barbitúricos sin receta (amytal, nembutal, seconal)

__ Fentanyl solo

__ Fentanyl y cocaína juntos

__ Fentanyl y heroína juntos

__ Fentanyl y metanfetamina/cristal juntos

__ Fentanyl y China White juntos

__ Otra droga o combinación de drogas que no se mencionó antes

__ Se rehúsa a contestar

If B827X is not equal to 1, then siga con P B829.

B828. Por favor especifique que droga(s) uso mientras estuvo en prisión/cárcel.

—————————————————————————————

B829. ¿Te inyectaste drogas mientras estuviste en la cárcel/prisión?

1 Si

0 No

8 Se rehúsa a contestar

If B829 is not equal to 1, then siga con P instrucción antes de B836.

B830. ¿Utilizó alguna jeringa nueva mientras estuvo en prisión / cárcel?

1 Si

0 No

8 Se rehúsa a contestar

If B830 is equal to 1 then siga con P B831

If not, then siga con P B832.

B831. En promedio, ¿cuántas jeringas nuevas utilizó en un mes en la prisión / cárcel?

__ __ __ jeringas

998 Se rehúsa a contestar

B832. ¿Utilizó jeringas usadas o jeringas viejas mientras estuvo en la prisión/cárcel?

1 Si

0 No

8 Se rehúsa a contestar

If B832 is equal to 1 then siga con P B833

If not, then siga con P instrucción antes de B836.

B833. Cuando se inyectó drogas en prisión/cárcel, ¿qué tan seguido utilizó una jeringa que usted sabia o sospechaba que había sido usada antes por otra persona? (Escoja una)

1 Nunca

2 A veces

3 Como la mitad de las veces

4 A menudo

5 Siempre

8 Se rehúsa a contestar

B834. De las veces que se inyectó con otras personas en prisión/cárcel, ¿qué tan seguido ha dividido drogas con alguien más usando una jeringa? Esto también es conocido como backloading, piggybacking o dividir drogas mojadas. (Escoja una)

1 Nunca

2 Menos de la mitad de las veces

3 Como la mitad de las veces

4 Más de la mitad de las veces

5 Siempre

8 Se rehúsa a contestar

If B833 is not equal to 1 and B833 is not equal to 8 then siga con P B835

If not, then siga con P instrucción antes de B836.

B835. De las veces que se inyectó con una jeringa usada por alguien más en la prisión/cárcel, ¿qué tan seguido la limpió con cloro antes de usarla? (Escoja una)

1 Nunca

2 Menos de la mitad de las veces

3 Como la mitad de las veces

4 Más de la mitad de las veces

5 Siempre

8 Se rehúsa a contestar

If B825 is equal to 1, then siga con P instrucción antes de B837.

B836. ***Cuando saliste te prisión/ cárcel, ¿recibiste cualquiera de las siguientes...*** (Marque todas las que apliquen)

__ Metadona, suboxona o buprenorfina (Terapia de Antagonista de Opioides

__ Referencia a un programa de metadona, suboxona o burprenorfina

__ Referencia a otros tratamientos de drogas o servicios

__ Naloxona/Narcan

__ Referencia a un intercambio de jeringa

__ Se rehúsa a contestar

LEA: LEA: Ahora le voy a hacer algunas preguntas acerca de sus experiencias en centros de detención en donde estuvo en custodia). Para el propósito de estas preguntas, se considera un centro de detención como un lugar en donde puede estar detenido hasta 72 horas (por ejemplo, Fuerzas especiales, La 20 o Cárcel Municipal o Centro Municipal de Infractores, Separos de la Judicial).

LEA: LEA: Te voy a preguntar primero sobre tus experiencias en centros de detención a lo largo de tu vida y en los últimos 6 meses.

B837. ¿Alguna vez has estado en un centro de detención (no la cárcel)?

1 Si

0 No

8 Se rehúsa a contestar

If B837 is not equal to 1, then siga con P instrucción antes de B842.

LEA: LEA: Ahora le voy a hacer algunas preguntas acerca de sus experiencias en centros de detención en los últimos 6 meses. Para el propósito de estas preguntas, se considera un centro de detención como un lugar en donde puede estar detenido hasta 72 horas.

B838. En los últimos seis meses, ¿cuántas veces ha estado en un centro de detención?

__ __ __ veces

998 Se rehúsa a contestar

If B838 is equal to 0 or B838 is equal to 998, then siga con P instrucción antes de B842.

B839. En los últimos 6 meses, ¿Cuantos días estuvo usted en un centro de detención en los últimos 6 meses?

__ __ veces

98 Se rehúsa a contestar

If B839 is less than 2, then siga con P instrucción antes de B842.

B840. En los últimos 6 meses, ¿En dónde se ubica este centro de detención (Marque todas las que apliquen)

__ Condado de San Diego

__ Tijuana

__ Otra ciudad en EUA

__ Otra ciudad en México

__ Otro

__ Se rehúsa a contestar

If B840E is not equal to 1, then siga con P instrucción antes de B842.

B841. Por favor especifique en dónde se ubicaba estaba ubicada este centro de detención

____

If B816 is greater than 0 and B816 is not equal to 998 or B839 is greater than 0 and B839 is not equal to 998 then siga con P B842

If not, then siga con P instrucción antes de B843.

B842. ¿Saliste de prisión, cárcel, o un centro de detención en los últimos 30 días?

1 Si

0 No

8 Se rehúsa a contestar

TRATAMIENTO Y PRUEBA DE HEPATITIS C (VHC)

LEA: LEA: Las siguientes preguntas son sobre Hepatitis C.

B843. ¿Alguna vez le han hecho la prueba de la Hepatitis C?

1 Si

0 No

7 No sé

8 Se rehúsa a contestar

If B843 is equal to 1 then siga con P B844

If not, then siga con P instrucción antes de B845.

B844. ¿Cuándo se hizo la prueba para Hepatitis C? [ENTREVISTADOR: SI PARTICIPANTE NO RESPONDE INMEDIATAMENTE, PREGUNTAR HASTA LLEGAR A UNA FECHA APROXIMADA]

__ __ / __ __ / __ __ __ __ dd / mm / aaaa

2097 No sé (Año)

2098 Se rehúsa a contestar (Año)

2099 No corresponde (Año)

If B843 is equal to 0 then siga con P B845

If not, then siga con P instrucción antes de B848.

B845. ¿ Comparado con otros usuarios de droga en esta ciudad que tan probable cree usted que se pueda contagiar con Hepatitis C (VHC)? (Escoja una)

1 Mucho más probable

2 Muy probable

3 Igual

4 Muy improbable

5 Mucho más improbable

8 Se rehúsa a contestar

B846. ¿Por qué nunca te has hecho una prueba VHC? (Marque todas las que apliquen)

__ Nunca he escuchado de Hep C

__ No sabía que había una prueba

__ No quiera pagar el costo de le prueba

__ Yo nunca, o casi nunca, voy a las clínicas u hospitales

__ No quiero saber si tengo VHC

__ No me importa si tengo VHC

__ No sé a dónde ir para obtener prueba de VHC

__ Otro

__ No sé

__ Se rehúsa a contestar

If B846H is not equal to 1, then siga con P instrucción antes de B848.

B847. Por favor especifica porque nunca has tenido una prueba de VHC:

__ __

If B843 is equal to 1 then siga con P B848

If not, then siga con P B859.

B848. ¿Dónde le hicieron la prueba de VHC fuera de este estudio? (Escoja una)

0 Como parte de un estudio de investigación

1 Centro de rehabilitación de drogas

2 Clínica privada/oficina del doctor

3 Clínica pública/Hospital

4 Otro lugar

7 No sé

8 Se rehúsa a contestar

If B848 is equal to 0 then siga con P B849

If not, then siga con P instrucción antes de B850.

B849. Por favor especifica en qué estudio:

____

If B848 is equal to 4 then siga con P B850

If not, then siga con P B851.

B850. Por favor especifica la ubicación:

____

B851. ¿Cuál fue su último resultado de la Prueba de VHC? (Escoja una)

0 Negativo

1 Positivo

2 Nunca obtuvo el resultado

7 No sé

8 Se rehúsa a contestar

If B851 is not equal to 0 then siga con P B852

If not, then siga con P B859.

B852. ¿Un doctor o un trabajador de salud le dijo que usted tiene Hepatitis C?

1	Si
0	No
7	No sé
8	Se rehúsa a contestar

If B852 is equal to 1 then siga con P B853

If not, then siga con P B859.

B853. ¿Cuándo fue la primera vez que dió positivo en una prueba de Hepatitis C?

__ __ / __ __ __ __	mm / aaaa
2097	No sé (Año)
2098	Se rehúsa a contestar (Año)

B854. ¿Un doctor enfermera(o) o trabajador de salud le ofreció tratamiento para el Hepatitis C?

1	Si
0	No
7	No sé
8	Se rehúsa a contestar

If B854 is not equal to 1, then siga con P B859.

B855. ¿Alguna vez en su vida Usted tomó medicamentos recetados por un doctor para previnir que se enferme por la Hepatitis C?

1	Si
0	No
7	No sé
8	Se rehúsa a contestar

If B855 is not equal to 1, then siga con P B859.

B856. ¿Terminaste tu tratamiento?

1	Si
0	No
7	No sé
8	Se rehúsa a contestar

If B856 is not equal to 0, then siga con P B859.

B857. ¿Porqué no se está tomando sus medicamentos? (Marque todas las que apliquen)

__ Me siento bien/ saludable

__ Tengo otras condiciones médicas

__ No tengo seguro de salud

__ No tengo dinero

__ Estoy consumiendo drogas/tomando alcohol

__ Perdí mi cita

__ Mi doctor me recetó pastillas pero me negué tomarlas

__ Mi doctor me dijo que no necesito medicamento

__ Dejé de ir a mi médico/nunca regresé

__ No tengo doctor

__ No quería tomar el medicamento disponible

__ No quería que me pusieran inyecciones

__ Otro

__ No sé

__ Se rehúsa a contestar

If B857M is equal to 1 then siga con P B858

If not, then siga con P B859.

B858. Por favor, especifique la razón por la que no está tomando el medicamento.

____

B859. Ahora hay tratamientos disponibles que son una pastilla al día por 8-12 semana las cuales pueden curar >90% del VHC de las personas con pocos o sin efectos secundarios. ¿Estarías dispuesto/a ser tratado/a?

1 Si

0 No

7 No sé

8 Se rehúsa a contestar

If B859 is equal to 0 then siga con P B860

If not, then siga con P instrucción antes de B861.

B860. ¿Me puedes decir por qué no estarías dispuesto/a a ser tratado/a para VHC?

____

VIH/SIDA

LEA: ENTREVISTADOR LEA EN VOZ ALTA: Vamos muy bien. Ahora vamos a hablar sobre VIH/SIDA. Le haré algunas preguntas generales y luego algunas preguntas sobre su experiencia con pruebas y tratamientos para VIH.

B861. Comparado con otros usuarios de drogas en esta ciudad, ¿qué tan probable cree Ud. que se pueda contagiar con VIH/SIDA? (Escoja una)

1 Muy Probable

2 Un poco más probable

3 Igual

4 Menos Probable

5 Mucho menos probable

6 No aplicable (por ejemplo: ya es VIH-positiva)

8 Se rehúsa a contestar

If B861 is equal to 6, then siga con P B865.

B862. ¿Alguna vez en la vida le han hecho la prueba de VIH antes de hoy?

1 Si

0 No

7 No sé

8 Se rehúsa a contestar

If B862 is equal to 0 then siga con P B863

If not, then siga con P instrucción antes de B865.

B863. ¿Por qué nunca te has hecho una prueba VIH/SIDA? (Marque todas las que apliquen)

__ No quiero pagar el costo de le prueba

__ Yo nunca, o casi nunca, voy a las clínicas u hospitales

__ No quiero saber si tengo VIH

__ No me importa si tengo VIH

__ No sé a dónde ir para obtener prueba de VIH

__ Otro

__ No sé

__ Se rehúsa a contestar

If B863F is not equal to 1, then siga con P instrucción antes de B865.

B864. Por favor especifica porqué nunca te has hecho una prueba de VIH:

__ __

If B862 is equal to 1 then siga con P B865

If not, then siga con P instrucción antes de B870.

B865. ¿Dónde le hicieron la prueba de VIH la última vez? (Escoja una)

0 Como parte de un estudio de investigación

1 Centro de rehabilitación de drogas

2 Clínica privada/oficina del doctor

3 Clínica pública/Hospital

4 Otro lugar

7 No sé

8 Se rehúsa a contestar

If B865 is equal to 0 then siga con P B866

If not, then siga con P instrucción antes de B867.

B866. Por favor especifica el nombre del estudio:

__ __

If B865 is equal to 4 then siga con P B867

If not, then siga con P B868.

B867. Por favor especifica la ubicación:

__ __

B868. ¿Cuándo fue la última vez que le hicieron una prueba de VIH/SIDA?
[ENTREVISTADOR: PON UNA FECHA APROXIMADA SI EL PARTICIPANTE NO RECUERDA EXACTAMENTE CUANDO FUE]

__ __ / __ __ __ __	mm / aaaa
2097	No sé (Año)
2098	Se rehúsa a contestar (Año)

B869. ¿Cuál fue su ultimo resultado de la Prueba de VIH/SIDA? (Escoja una)

0	Negativo
1	Positivo
2	Nunca obtuve el resultado
7	No sé
8	Se rehúsa a contestar

If B869 is equal to 1 then siga con P B870

If not, then siga con P instrucción antes de B871.

B870. ¿Cuándo fue la primera vez que dió positivo en una prueba de VIH/SIDA?
[ENTREVISTADOR: PON UNA FECHA APROXIMDADA SI EL PARTICIPANTE NO RECUERDA EXACTAMENTE CUANDO FUE]

__ __ / __ __ __ __	mm / aaaa
2097	No sé (Año)
2098	Se rehúsa a contestar (Año)

If B869 is equal to 1 or B869 is equal to 2 then siga con P B871

If not, then siga con P B878.

B871. ¿Un doctor o un trabajador de salud le dijo que usted tiene VIH/SIDA?

1	Si
0	No
7	No sé
8	Se rehúsa a contestar

If B871 is not equal to 1, then siga con P B878.

B872. ¿Un doctor enfermera(o) o trabajador de salud le ofreció tratamiento para el VIH/SIDA?

1	Si
0	No
7	No sé
8	Se rehúsa a contestar

If B872 is not equal to 1, then siga con P B878.

B873. Alguna vez en la vida, ¿algún doctor o enfermera le dió algún tratamiento o medicina para evitar que se enfermara de VIH/SIDA?

1 Si

0 No

7 No sé

8 Se rehúsa a contestar

If B873 is not equal to 1, then siga con P B878.

B874. ¿Todavia sigue tomando estos medicamentos?

1 Si

0 No

7 No sé

8 Se rehúsa a contestar

If B874 is not equal to 0, then siga con P B878.

B875. ¿Por cuánto tiempo tomaste estos medicamentos?

__ __ __ AñOS

__ __ __ MESES

__ __ __ DíAS

997 No sé (Meses)

998 Se rehúsa a contestar (Meses)

B876. ¿Porqué no se esta tomando estos medicamentos? (Marque todas las que apliquen)

__ Me siento bien/ saludable

__ Tengo otras condiciones médicas

__ No tengo seguro de salud

__ No tengo dinero

__ Estoy consumiendo drogas / tomando alcohol

__ Perdí mi cita

__ Mi doctor me recetó pastillas pero me negué tomarlas

__ Mi doctor me dijo que no necesito medicamento

__ Dejé de ir a mi médico/nunca regresé

__ No tengo doctor

__ No quería tomar el medicamento disponible

__ Las medicinas me hacían sentir enfermo

__ No quería que mi familia/otros supieran que estaba enfermo

__ No podía conseguir mis medicinas ya que me andaba mudando a menudo

__ Otro

__ No sé

__ Se rehúsa a contestar

If B876O is equal to 1 then siga con P B877

If not, then siga con P B878.

B877. Por favor, especifique la razón por la que no está tomando el medicamento.

____

B878.	¿Ha conocido personalmente a alguien con VIH/SIDA?	1	Si
		0	No
		7	No sé
		8	Se rehúsa a contestar

If B878 is equal to 1 then siga con P B879

If not, then siga con P instrucción antes de B881.

B879.	¿A cuántas personas?	__ __ __	personas
		997	No sé
		998	Se rehúsa a contestar

B880. ¿A cuántas personas conoce personalmente que hayan fallecido por VIH/SIDA?

__ __ __	personas
997	No sé
998	Se rehúsa a contestar

SECCION PrEP

LEA: ENTREVISTADOR LEA EN VOZ ALTA:
La profilaxis antes de la exposición o PrEP es una medicina que las personas en riesgo de infectarse con el VIH por relaciones sexuales o uso de drogas inyectables. Cuando la medicina se toma a diario como se la ha recetado, el PrEP funciona muy bien para prevenir la infección por VIH. El PrEP aún no está disponible ampliamente en México, pero si está disponible en EUA desde hace varios años ya. El PrEP es seguro, pero algunas personas tienen efectos secundarios como diarrea, náuseas y Dolores de cabeza. Estos efectos secundarios usualmente se desaparecen gradualmente con el tiempo. El PrEP no protege contra otras infecciones de transmisión sexual (ITSs), por lo que es importante que también siga usando condones cuando está tomando PrEP. Como el PrEP no trata la infección de VIH, es también muy importante que vea a un doctor cada tres meses para hacerse las pruebas para VIH y otras ITSs cada tres meses y otros exámenes de laboratorio para asegurarse que todo está bien con su salud.

B881. Antes de hoy, ¿Habías escuchado sobre personas que son VIH negativo tomando medicamento para VIH o PrEP **antes** de ser expuestos a VIH para proteger de la infección de VIH?

1 Si

0 No

8 Se rehúsa a contestar

If B881 is not equal to 1, then siga con P B889.

B882. ¿Has tomando medicamento para VIH o PrEP **antes** de ser expuesto a VIH para protegerte de la infección de VIH?

1 Si

0 No

8 Se rehúsa a contestar

If B882 is not equal to 1, then siga con P B889.

B883. ¿En qué ciudad te prescribió un doctor terapia PrEP? (Marque todas las que apliquen)

__ Condado de San Diego

__ Tijuana

__ Otra ciudad en EUA

__ Otra ciudad en México

__ Otro

__ No sé

__ Se rehúsa a contestar

If B883E is not equal to 1, then siga con P B885.

B884. Por favor, especifique en qué ciudad le recetaron su terapia PrEP

__ __

B885. ¿Actualmente estas tomando terapia PrEP?

1 Si

0 No

7 No sé

8 Se rehúsa a contestar

If B885 is not equal to 1, then siga con P B889.

B886. ¿Cuándo comenzaste tu terapia PrEP actual? **[ENTREVISTADOR: PON UNA FECHA APROXIMDADA SI EL PARTICIPANTE NO RECUERDA EXACTAMENTE CUANDO FUE]**

__ __ / __ __ __ __ mm / aaaa

2097 No sé (Año)

2098 Se rehúsa a contestar (Año)

B887. ¿Cuál terapia PrEP estas tomando actualmente? (Escoja una)

01	Truvada (una vez al día, oral)
02	Maraviroc (una vez al día, oral)
03	Rilpivirine (una vez al mes, inyección)
04	Dapivirine (anillo vaginal se usa 4 semanas a la vez)
05	Tenofovir (gel insertado rectal/vaginal)
06	Cabotegravir (PrEP inyectable)
07	Otro
97	No sé
98	Se rehúsa a contestar

If B887 is not equal to 7, then siga con P B889.

B888. Por favor especifica qué otra terapia PrEP estas tomando.

__ __

B889. ¿Qué tipo de producto PrEP estarías interesado/a en tomar? (Marque todas las que apliquen)

__ Una pastilla diaria

__ Una inyección cada 2 semanas

__ (para mujeres) Gel vaginal que debo usar antes de tener relaciones sexuales

__ Ninguno

__ Se rehúsa a contestar

__ No corresponde

If B889D is equal to 1 and B889A + B889B + B889C is greater than 0 then LEA: "El participante indicó Ninguno junto con otras opciones. Por favor, revise la respuesta." and siga con P B889.

LEA: LEA: Ahora le haré preguntas sobre su salud sexual en los últimos 6 meses.

B890. En los ultimos 6 meses, ha tenido alguno de los siguientes síntomas? **Lea en voz alta la lista** (Marque todas las que apliquen)

__ Orinar frecuentemente o orinar con dolor

__ Ulceras dolorosas o ampollas en sus genitales

__ Verrugas en sus genitales

__ Desecho o goteo por sus genitales

__ Comezón en su área genital

__ Sangrado después de tener sexo

__ No he tenido ninguno de estos síntomas

__ No sé

__ Se rehúsa a contestar

If B890G is equal to 1 and B890A + B890B + B890C + B890D + B890E + B890F is greater than 0 then LEA: "El participante indicó "No he tenido ninguno de estos síntomas" junto con otras opciones. Por favor, revise la respuesta." and siga con P B890.

B891. **En los ultimos 6 meses, lo han diagnosticado con alguno de los siguientes problemas de salud? Lea en voz alta la lista** (Marque todas las que apliquen)

__ Gonorrea o goteo por genitales

__ Clamidia, uretritis no gonocócica, uretritis no especifica

__ Sífilis (RPR,VDRL)

__ Verrugas genitales, (en, sobre o alrededor del área genital)

__ Verrugas anales, (en, sobre o alrededor del ano)

__ Chancro (lesión o ulcera en o alrededor de la vagina, pene o ano

__ Hepatitis B

__ Hepatitis C

__ Tricomoniasis

__ Enfermedad Pélvica Inflamatoria

__ Candidiasis Vaginal

__ Cáncer Cervical

__ Infección de Transmisión Sexual no sabe cual

__ Otros problemas de la mujer

__ Nunca he sido diagnosticada con ninguna infección de transmisión sexual

__ No sé

__ Se rehúsa a contestar

If B891O is equal to 1 and B891A + B891B + B891C + B891D + B891E + B891F + B891G + B891H + B891I + B891J + B891K + B891L + B891M + B891N is greater than 0 then LEA: "El participante indicó "No he tenido ninguno de estos síntomas" junto con otras opciones. Por favor, revise la respuesta." and siga con P B891.

If B891N is not equal to 1, then siga con P B893.

B892. Por favor díganos sobre qué otro problema femenino

____

B893. En los últimos 6 meses, ¿algún doctor o enfermera le dió algún tratamiento o medicina para tratar cualquier síntoma en sus genitales?

1	Si
0	No
8	Se rehúsa a contestar

COMPORTAMIENTO SEXUAL

LEA: LEA: Le agradezco su cooperación, buen trabajo. Casi terminamos. Ahora le preguntaré sobre sus experiencias sexuales en los últimos seis meses. Si usted ha estado en prisión/cárcel durante ese tiempo, estas preguntas se refieren al tiempo en que usted NO estaba en prisión/cárcel. Nuevamente, sé que estas preguntas pueden ser incomodas para contestar, pero serán de mucha utilidad para ayudarnos a prevenir enfermedades a usted y personas como usted. Por favor trate de ser lo más honesto que pueda. Para estas preguntas, cuando yo diga sexo se refiere a sexo oral, vaginal o anal. Cuando decimos usar protección nos referimos a usar un condón de hombre o uno de mujer o una barrera para prevenir el contacto de fluidos corporales durante la actividad sexual.

B894. ¿Qué edad tenías la **primera vez** que tuviste sexo vaginal o anal consensual. Por sexo consensual, quiero decir que tú aceptaste tener sexo por tu propia cuenta. (Entrevistador: Por favor ingresar 0 para Nunca he tenido sexo consensual.)

__ __	años
98	Se rehúsa a contestar

If B894 is greater than A1 and B894 is not equal to 98 then LEA: "El participante ha respondido con un período de tiempo mayor que su edad. Por favor revise las respuestas." and siga con P B894.

If B894 is equal to 0 or B894 is equal to 98, then siga con P B896.

B895. La primera vez que tuviste sexo, ¿fue con un hombre o una mujer? (Escoja una)

1	Hombre
2	Mujer
3	Los dos
8	Se rehúsa a contestar

B896. ¿Cómo te identificas (sexo)? (Escoja una)

1	Heterosexual
2	Homosexual/Gay/Lesbiana
3	Bisexual
4	Otro
8	Se rehúsa a contestar

If B896 is equal to 4 then siga con P B897

If not, then siga con P instrucción antes de B898.

B897. Por favor especifica de qué otra manera te identificas

____

If B26 is equal to 1 then siga con P instrucción antes de B898

If not, then siga con P B899.

LEA: LEA: A continuación le voy a mostrar algunas fotos. Esta foto es de un pene que ha sido circuncidado (le han quitado la piel que cubre la punta del pene), y ésta es de un pene que no ha sido circuncidado (la piel que cubre la punta todavía sigue allí).

B898. ¿Ha sido usted circuncidado? ***Entrevistador: LEER: Esta foto es de un pene que ha sido circuncidado (le han quitado la piel que cubre la punta del pene), y ésta es de un pene que no ha sido circuncidado (la piel que cubre la punta todavía sigue allí).***

1 Si

0 No

8 Se rehúsa a contestar

B899. ¿Con cuantas mujeres has tenido sexo en toda tu vida? **(LEER: Recuerda que, por sexo, me refiero a sexo vaginal, anal, u oral) (ENTREVISTADOR: Por favor usar 0 si el participante Nunca ha tenido sexo con mujeres**

__ __ __ __ Mujeres

9998 Se rehúsa a contestar

B900. ¿Con cuántos hombres has tenido sexo en toda tu vida? **(LEER: Recuerda que, por sexo, me refiero a sexo vaginal, anal, u oral) (ENTREVISTADOR: Por favor usar 0 si el participante Nunca ha tenido sexo con hombres.)**

__ __ __ __ Hombres

9998 Se rehúsa a contestar

B901. En los últimos **6 meses**, ¿ha tenido relaciones sexuales?

1 Si

0 No

8 Se rehúsa a contestar

If B901 is not equal to 1, then siga con P B944.

B902. ¿En qué ciudad tuviste sexo en los últimos 6 meses? (Marque todas las que apliquen)

__ Condado de San Diego

__ Tijuana

__ Otra ubicación en USA-EUA

__ Otra ubicación en México

__ Otro lugar

__ No sé

__ Se rehúsa a contestar

If B902E is equal to 1 then siga con P B903

If not, then siga con P B904.

B903. Por favor especifique en qué lugar tuvo relaciones sexuales en los últimos 6 meses

____

B904. Durante los **últimos 6 meses**, ¿ha tenido relaciones sexuales con hombres, mujeres o ambos? (Escoja una)

1 Hombres

2 Mujeres

3 Los dos

8 Se rehúsa a contestar

LEA: LEA: Ahora le voy a preguntar de su pareja regular. Por regular, nos referimos a alguien con quien se siente comprometido/a más que ninguna otra persona y con quien usted tiene relaciones sexuales

B905. En los **últimos 6 meses,** ¿tuvo una **pareja regular** con la cual se sintiera comprometida más que con ninguna otra persona y con quien usted tiene relaciones sexuales?

1 Si

0 No

8 Se rehúsa a contestar

If B905 is not equal to 1, then siga con P instrucción antes de B916.

B906. ¿Cuál es el sexo de esta pareja sexual? (Escoja una)

1 Hombre

2 Mujer

3 Transgénero

8 Se rehúsa a contestar

If B906 is not equal to 3, then siga con P B908.

B907. Si es transgénero, selecciona cuál (Escoja una)

1 Mujer a hombre

2 Hombre a Mujer

8 Se rehúsa a contestar

B908. ¿Era esta pareja regular un cliente?

1 Si

0 No

8 Se rehúsa a contestar

If B906 is equal to 1 or B907 is equal to 2 then siga con P B909

If not, then siga con P B910.

B909. ¿Está circuncidada su pareja regular?

1 Si

0 No

7 No sé

8 Se rehúsa a contestar

B910. ¿En qué país vive esta persona? (Escoja una)

1 EUA-USA

2 México

3 Otro

8 Se rehúsa a contestar

If B910 is not equal to 3, then siga con P B912.

B911. Por favor especifique en qué otro país vive esta persona

____

B912. En los últimos 6 meses, ¿qué tan seguido tuvo sexo vaginal con su pareja estable? (Escoja una)

1 Nunca

2 Una o dos veces

3 Cada dos o tres meses

4 Cada dos o tres semanas

5 Por lo menos una vez a la semana

6 Varias veces a la semana

8 Se rehúsa a contestar

B913. En los últimos 6 meses, ¿qué tan seguido tuvo sexo anal con su pareja estable? (Escoja una)

1 Nunca

2 Una o dos veces

3 Cada dos o tres meses

4 Cada dos o tres semanas

5 Por lo menos una vez a la semana

6 Varias veces a la semana

8 Se rehúsa a contestar

B914. En los **últimos 6 meses**, cuando usted tuvo relaciones sexuales con su **pareja regular**, ¿qué tan seguido usó condón? (Escoja una)

1	Nunca
2	A veces
3	Casi la mitad de las ocasiones
4	Seguido
5	Siempre
8	Se rehúsa a contestar

B915. **En los últimos seis meses Tuvo usted sexo anal SIN PROTECCIÓN (sin condón) la última vez que estuvo con su pareja regular?**

1	Si
0	No
8	Se rehúsa a contestar
9	No corresponde

LEA: LEA: Ahora le voy a preguntar de su pareja casual. Por casual, nos referimos a parejas con quien tiene relaciones sexuales pero no recibe bienes o servicios a cambio de sexo, aparte de su pareja regular.

B916. En los **últimos 6 meses**, ¿con cuántas parejas **casuales** tuvo relaciones sexuales aparte de su pareja regular?

__ __ __ __	parejas
9998	Se rehúsa a contestar

If B916 is equal to 0 or B916 is equal to 9998, then siga con P B940.

B917. ¿Estas parejas casuales fueron clientes/alguien que te dio dinero, drogas o algo más a cambio de sexo?

1	Si
0	No
8	Se rehúsa a contestar

B918. ¿Cuantos de estas parejas casuales ([Respuesta a P B916]) eran hombres?

__ __ __ __

9998	Se rehúsa a contestar

If B918 is greater than B916 and B918 is not equal to 9998 then LEA: "La respuesta del participante debe ser menor o igual al número de parejas casuales con las que tiene relaciones sexuales además de la pareja habitual. Por favor revise las respuestas." and siga con P B916.

If B918 is equal to 0 or B918 is equal to 9998, then siga con P instrucción antes de B922.

B919. ¿Cuántas parejas hombres eran de EUA-USA**?** __ __ __ __

9998	Se rehúsa a contestar

If B919 is greater than B918 and B919 is not equal to 9998 then LEA: "La respuesta del participante debe ser menor o igual al número de parejas casuales masculinas con las que tiene relaciones sexuales además de la pareja habitual. Por favor revise las respuestas." and siga con P B919.

If B919 is equal to B918, then siga con P instrucción antes de B922.

B920. ¿Cuántas parejas hombres eran de México**?** __ __ __ __

9998	Se rehúsa a contestar

If B919 + B920 is greater than B918 and B919 is not equal to 9998 and B920 is not equal to 9998 then LEA: "La respuesta del participante debe ser menor o igual al número de parejas casuales masculinas con las que tiene relaciones sexuales además de la pareja habitual. Por favor revise las respuestas." and siga con P B920.

If B919 + B920 is equal to B918, then siga con P instrucción antes de B922.

B921. ¿Cuántas parejas hombres eran de otro país**?** __ __ __ __

9998	Se rehúsa a contestar

If B919 + B920 + B921 is greater than B918 and B919 is not equal to 9998 and B920 is not equal to 9998 and B921 is not equal to 9998 then LEA: "La respuesta del participante debe ser menor o igual al número de parejas casuales masculinas con las que tiene relaciones sexuales además de la pareja habitual. Por favor revise las respuestas." and siga con P B921.

If B918 is equal to B916, then siga con P instrucción antes de B930.

B922. ¿Cuántas de estas parejas casuales mujeres ([Respuesta a P B916]) eran mujeres?

__ __ __ __

9998 Se rehúsa a contestar

If B918 + B922 is greater than B916 and B918 is not equal to 9998 and B922 is not equal to 9998 then LEA: "La respuesta del participante debe ser menor o igual al número de parejas casuales con las que tiene relaciones sexuales además de la pareja habitual. Por favor revise las respuestas." and siga con P B916.

If B922 is equal to 0 or B922 is equal to 9998, then siga con P instrucción antes de B926.

B923. ¿Cuántas parejas mujeres eran de EUA-USA**?** __ __ __ __

9998 Se rehúsa a contestar

If B923 is greater than B922 and B923 is not equal to 9998 then LEA: "La respuesta del participante debe ser menor o igual al número de parejas ocasionales femeninas con las que tiene relaciones sexuales además de su pareja habitual. Por favor revise las respuestas." and siga con P B923.

If B923 is equal to B922, then siga con P instrucción antes de B926.

B924. ¿Cuántas parejas mujeres eran de México**?** __ __ __ __

9998 Se rehúsa a contestar

If B923 + B924 is greater than B922 and B923 is not equal to 9998 and B924 is not equal to 9998 then LEA: "La respuesta del participante debe ser menor o igual al número de parejas ocasionales femeninas con las que tiene relaciones sexuales además de su pareja habitual. Por favor revise las respuestas." and siga con P B924.

If B923 + B924 is equal to B922, then siga con P instrucción antes de B926.

B925. ¿Cuántas parejas mujeres eran de otro país**?** __ __ __ __

9998 Se rehúsa a contestar

If B923 + B924 + B925 is greater than B922 and B923 is not equal to 9998 and B924 is not equal to 9998 and B925 is not equal to 9998 then LEA: "La respuesta del participante debe ser menor o igual al número de parejas ocasionales femeninas con las que tiene relaciones sexuales además de su pareja habitual. Por favor revise las respuestas." and siga con P B925.

If B918 + B922 is equal to B916, then siga con P instrucción antes de B930.

B926. ¿Cuántas de estas parejas casuales mujeres ([Respuesta a P B916]) eran transgéneros?

__ __ __ __

9998 Se rehúsa a contestar

If B918 + B922 + B926 is not equal to B916 and B918 is not equal to 9998 and B922 is not equal to 9998 and B926 is not equal to 9998 then LEA: "La respuesta del participante debe ser menor o igual a la cantidad de parejas ocasionales transgénero con las que tiene relaciones sexuales además de su pareja habitual. Por favor revise las respuestas." and siga con P B916.

If B926 is equal to 0 or B926 is equal to 9998, then siga con P instrucción antes de B930.

B927. ¿Cuántas parejas transgéneros eran de EUA-USA**?** __ __ __ __

9998 Se rehúsa a contestar

If B927 is greater than B926 and B927 is not equal to 9998 then LEA: "La respuesta del participante debe ser menor o igual a la cantidad de parejas ocasionales transgénero con las que tiene relaciones sexuales además de su pareja habitual. Por favor revise las respuestas." and siga con P B927.

If B927 is equal to B926, then siga con P instrucción antes de B930.

B928. ¿Cuántas parejas transgéneros eran de México**?** __ __ __ __

9998 Se rehúsa a contestar

If B927 + B928 is greater than B926 and B927 is not equal to 9998 and B928 is not equal to 9998 then LEA: "La respuesta del participante debe ser menor o igual a la cantidad de parejas ocasionales transgénero con las que tiene relaciones sexuales además de su pareja habitual. Por favor revise las respuestas." and siga con P B928.

If B927 + B928 is equal to B926, then siga con P instrucción antes de B930.

B929. ¿Cuántas parejas transgéneros eran de otros países**?**

__ __ __ __

9998 Se rehúsa a contestar

If B927 + B928 + B929 is greater than B926 and B927 is not equal to 9998 and B928 is not equal to 9998 and B929 is not equal to 9998 then LEA: "La respuesta del participante debe ser menor o igual a la cantidad de parejas ocasionales transgénero con las que tiene relaciones sexuales además de su pareja habitual. Por favor revise las respuestas." and siga con P B929.

If B922 is greater than 0 and B922 is not equal to 9998 then siga con P B930

If not, then siga con P instrucción antes de B934.

B930. En los últimos 6 meses, ¿qué tan seguido tuviste sexo vaginal con tus parejas casuales mujeres? (Escoja una)

1 Nunca

2 Una o dos veces

3 Cada dos o tres meses

4 Cada dos o tres semanas

5 Por lo menos una vez a la semana

8 Se rehúsa a contestar

If B930 is equal to 1 or B930 is equal to 8, then siga con P B932.

B931. En los últimos seis meses, ¿tuvo sexo vaginal SIN PROTECCIÓN (sin condón) la última vez que tuvo sexo con su pareja casual mujer?

1 Si

0 No

8 Se rehúsa a contestar

9 No corresponde

B932. En los últimos 6 meses, que tan seguido tuvo sexo anal con sus parejas casuales mujeres? (Escoja una)

1 Nunca

2 Una o dos veces

3 Cada dos o tres meses

4 Cada dos o tres semanas

5 Por lo menos una vez a la semana

8 Se rehúsa a contestar

If B932 is equal to 1 or B932 is equal to 8, then siga con P instrucción antes de B934.

B933. **¿Tuvo sexo anal SIN PROTECCIÓN (sin condón) la última vez que tuvo sexo con su pareja casual mujer?**

1 Si

0 No

8 Se rehúsa a contestar

9 No corresponde

If B918 is greater than 0 and B918 is not equal to 9998 then siga con P B934

If not, then siga con P instrucción antes de B938.

B934. En los últimos 6 meses, que tan seguido tuvo usted sexo vaginal con sus parejas casuales hombres? (Escoja una)

1 Nunca

2 Una o dos veces

3 Cada dos o tres meses

4 Cada dos o tres semanas

5 Por lo menos una vez a la semana

8 Se rehúsa a contestar

If B934 is equal to 1 or B934 is equal to 8, then siga con P B936.

B935. **¿Tuvo sexo vaginal SIN PROTECCIÓN (sin condón) la última vez que tuvo sexo con su pareja casual hombre?**

1 Si

0 No

8 Se rehúsa a contestar

9 No corresponde

B936. En los últimos 6 meses, ¿que tan frecuentemente tuvo sexo anal con tus parejas casuales hombres? (Escoja una)

1 Nunca

2 Una o dos veces

3 Cada dos o tres meses

4 Cada dos o tres semanas

5 Por lo menos una vez a la semana

8 Se rehúsa a contestar

If B936 is equal to 1 or B936 is equal to 8, then siga con P instrucción antes de B938.

B937. **¿Tuvo sexo anal SIN PROTECCIÓN (sin condón) la última vez que tuvo sexo con su pareja casual hombre?**

1 Si

0 No

8 Se rehúsa a contestar

9 No corresponde

If B926 is greater than 0 and B926 is not equal to 9998 then siga con P B938

If not, then siga con P B940.

B938. En los últimos 6 meses, ¿qué tan frecuentemente tuvo sexo con sus parejas casuales transgénero? (Escoja una)

1 Nunca

2 Uno o dos veces

3 Cada dos o tres meses

4 Cada dos o tres semanas

5 Por lo menos una vez a la semana

8 Se rehúsa a contestar

If B938 is equal to 1 or B938 is equal to 8, then siga con P B940.

B939. **¿Tuvo sexo SIN PROTECCIÓN (sin condón) la última vez que tuvo sexo con su pareja casual transgénero?**

1 Si

0 No

8 Se rehúsa a contestar

9 No corresponde

B940. En los últimos **6 meses,** cuando tuvo relaciones sexuales, ¿qué tan seguido había estado borracho(a) **dentro de un periodo de 2 horas antes o durante el sexo**? (Escoja una)

1 Nunca

2 A veces

3 Casi la mitad de las ocasiones

4 Seguido

5 Siempre

8 Se rehúsa a contestar

B941. En los **últimos 6 meses, cuando tuvo relaciones sexuales**, ¿qué tan seguido había usado drogas **dentro de un periodo de 2 horas antes o durante el sexo**? (Escoja una)

1 Nunca

2 A veces

3 Casi la mitad de las ocasiones

4 Seguido

5 Siempre

8 Se rehúsa a contestar

B942. ¿Alguna vez ha tenido relaciones sexuales con alguien que usted en ese tiempo sabía que estaba infectado con VIH/SIDA?

1 Si

0 No

8 Se rehúsa a contestar

If B942 is equal to 1 then siga con P B943

If not, then siga con P B944.

B943. Si es así ¿con qué frecuencia usó un condón? (Escoja una)

1 Nunca

2 A veces

3 Casi la mitad de las ocasiones

4 Seguido

5 Siempre

8 Se rehúsa a contestar

B944. ¿Alguien le ha dado algo que usted necesitaba (como dinero, drogas, albergue, comida o transportación o protección) a cambio de sexo?

1 Si

0 No

8 Se rehúsa a contestar

If B917 is equal to 1 then siga con P B945

If not, then siga con P instrucción antes de B946.

B945. Antes dijiste que tu(s) pareja(s) ocasional(s) te daban dinero, drogas u otra cosa a cambio de sexo. ¿Podría, por favor, reconsiderar la pregunta: Alguien le ha dado algo que usted necesitaba (como dinero, drogas, albergue, comida o transportación o protección) a cambio de sexo?

1 Si

0 No

8 Se rehúsa a contestar

If B944 is not equal to 1 and B945 is not equal to 1, then siga con P instrucción antes de B973.

B946. Cuando alguien le ha dado algo que usted necesitaba a cambio de sexo, ¿qué le dió? **(Entrevistador: Lea la lista y marque todas las respuestas que apliquen.)** (Marque todas las que apliquen)

__	Dinero
__	Drogas
__	Jeringas
__	Alcohol
__	Albergue
__	Transportación
__	Protección
__	Comida
__	Trabajo
__	Ropa
__	Regalos
__	Otro
__	Se rehúsa a contestar

If B946L is equal to 1 then siga con P B947

If not, then siga con P B948.

B947. Por favor especifique qué otra cosa le dió:

__ __

B948. ¿Qué edad tenías la primera vez que alguien te dio algo que tu necesitabas a cambio de sexo?

__ __	años
98	Se rehúsa a contestar

If B948 is greater than A1 and B948 is not equal to 98 then LEA: "El participante ha respondido con un período de tiempo mayor que su edad. Por favor revise las respuestas." and siga con P B948.

If B901 is not equal to 1, then siga con P instrucción antes de B973.

B949. En los **últimos 6 meses**, ¿cuántas personas le han dado algo que usted necesitaba a cambio de tener relaciones sexuales con usted?

__ __ __ __ personas

9998 Se rehúsa a contestar

If B949 is equal to 0 or B949 is equal to 9998, then siga con P instrucción antes de B973.

B950. *¿En qué ciudad (ciudades) alguien te dió algo a cambio de sexo?* (Marque todas las que apliquen)

__ Condado de San Diego

__ Tijuana

__ Otra ciudad en EUA

__ Otra ciudad en México

__ Otro

__ Se rehúsa a contestar

If B950E is equal to 1 then siga con P B951

If not, then siga con P B952.

B951. Por favor especifique en qué otra ciudad *alguien te dió algo a cambio de sexo*:

__ __

B952. En los últimos 6 meses, en promedio, ¿cuantas veces al mes alguien le dio algo que necesitaba a cambio de tener sexo con ellos? **Entrevistador: Si el participante dice "No Se" pregúntele por la respuesta más aproximada.**

__ __ __

998 Se rehúsa a contestar

B953. Durante los últimos 6 meses, ¿Cuál de los siguientes le ha dado alguien a cambio de tener sexo con ellos? **Entrevistador: Lea las opciones y marque las que apliquen.** (Marque todas las que apliquen)

__	Dinero
__	Drogas
__	Jeringas
__	Alcohol
__	Albergue
__	Transportación
__	Protección
__	Comida
__	Trabajo
__	Ropa
__	Regalos
__	Otro
__	Se rehúsa a contestar

If B953L is equal to 1 then siga con P B954

If not, then siga con P B955.

B954. Por favor especifique qué otra cosa ***alguien te dió algo a cambio de sexo***:

— —

B955. En los últimos 6 meses, cuando alguien te doy algo que necesitabas a cambio de sexo, ¿cuántas de estas ([Respuesta a P B949]) personas eran hombres?

— — — —

9998	Se rehúsa a contestar

If B955 is greater than B949 and B955 is not equal to 9998 then LEA: "La respuesta del participante debe ser menor o igual a la cantidad de personas que les dieron algo a cambio de sexo. ¡Por favor revise las respuestas y haga las correcciones!" and siga con P B955.

If B955 is equal to 0 or B955 is equal to 9998, then siga con P instrucción antes de B959.

B956. ¿Cuántos hombres que te dieron algo que necesitabas a cambio de sexo eran de: EUA-USA**?**

— — — —

9998 Se rehúsa a contestar

If B956 is greater than B955 and B956 is not equal to 9998 then LEA: "La respuesta del participante debe ser menor o igual a la cantidad de hombres que les dieron algo que necesitaban a cambio de sexo. Por favor revise las respuestas." and siga con P B956.

If B956 is equal to B955, then siga con P instrucción antes de B959.

B957. ¿Cuántos hombres que te dieron algo que necesitabas a cambio de sexo eran de México**?**

— — — —

9998 Se rehúsa a contestar

If B956 + B957 is greater than B955 and B956 is not equal to 9998 and B955 is not equal to 9998 then LEA: "La respuesta del participante debe ser menor o igual a la cantidad de hombres que les dieron algo que necesitaban a cambio de sexo. Por favor revise las respuestas." and siga con P B957.

If B956 + B957 is equal to B955, then siga con P instrucción antes de B959.

B958. ¿Cuántos hombres que te dieron algo que necesitabas a cambio de sexo eran de otros países**?**

— — — —

9998 Se rehúsa a contestar

If B956 + B957 + B958 is greater than B955 and B956 is not equal to 9998 and B957 is not equal to 9998 and B958 is not equal to 9998 then LEA: "La respuesta del participante debe ser menor o igual a la cantidad de hombres que les dieron algo que necesitaban a cambio de sexo. Por favor revise las respuestas." and siga con P B958.

If B955 is equal to B949, then siga con P B963.

B959. En los últimos 6 meses, cuando alguien te dió algo a cambio de sexo, ¿cuántas de estas ([Respuesta a P B949]) personas eran mujeres?

— — — —

9998 Se rehúsa a contestar

If B955 + B959 is greater than B949 and B955 is not equal to 9998 and B959 is not equal to 9998 then LEA: "La respuesta del participante debe ser menor o igual a la cantidad de personas que les dieron algo a cambio de sexo. Verifique las respuestas y haga las correcciones." and siga con P B959.

If B959 is equal to 0 or B959 is equal to 9998, then siga con P B963.

B960. ¿Cuántas mujeres que te dieron algo que necesitabas a cambio de sexo eran de: EUA-USA**?**

— — — —

9998 Se rehúsa a contestar

If B960 is greater than B959 and B960 is not equal to 9998 then LEA: "La respuesta del participante debe ser menor o igual al número de mujeres que les dieron algo que necesitaban a cambio de sexo. Por favor revise las respuestas." and siga con P B960.

If B960 is equal to B959, then siga con P B963.

B961. ¿Cuántas mujeres que te dieron algo que necesitabas a cambio de sexo eran de: México**?**

— — — —

9998 Se rehúsa a contestar

If B960 + B961 is greater than B959 and B960 is not equal to 9998 and B959 is not equal to 9998 then LEA: "La respuesta del participante debe ser menor o igual al número de mujeres que les dieron algo que necesitaban a cambio de sexo. Por favor revise las respuestas." and siga con P B961.

If B960 + B961 is equal to B959, then siga con P B963.

B962. ¿Cuántas mujeres que te dieron algo que necesitabas a cambio de sexo eran de otros países**?**

— — — —

9998 Se rehúsa a contestar

If B960 + B961 + B962 is greater than B959 and B960 is not equal to 9998 and B961 is not equal to 9998 and B962 is not equal to 9998 then LEA: "La respuesta del participante debe ser menor o igual al número de mujeres que les dieron algo que necesitaban a cambio de sexo. Por favor revise las respuestas." and siga con P B962.

B963. En los **últimos 6 meses,** cuando alguien le dio algo que usted necesitaba a cambio de sexo, ¿dónde conoció a esta persona? (Marque todas las que apliquen)

__ Calle

__ Trabajo

__ Picadero

__ Vecindario

__ Escuela

__ Bar/Antro

__ Hotel

__ Periódico/Revista/Internet

__ Amigos/Conocidos

__ Padrote

__ Otro

__ Se rehúsa a contestar

If B963K is not equal to 1, then siga con P B965.

B964. Por favor especifica qué otro lugar:

_ _

B965. Desde que comenzó la pandemia de COVID en Marzo de 2020, ¿el número de personas que le ha dado dinero, drogas o cualquier otra cosa a cambio de tener relaciones sexuales con ellos a cambiado? (Escoja una)

1	Más gente
2	Menos gente
3	Igual
8	Se rehúsa a contestar

B966. En los últimos 6 meses, ¿Qué tan seguido tuviste sexo a cambio de algo que tú necesitabas? (Escoja una)

1	Una o dos veces al mes
2	Una o dos veces al mes
3	Al menos una vez a la semana
4	Varias veces a la semana
5	Varias veces al día
8	Se rehúsa a contestar

B967. En los últimos 6 meses, cuando tuvo relaciones sexuales a cambio de algo que necesitaba, ¿qué tan seguido tuvo sexo vaginal? (Escoja una)

1	Nunca
2	A veces
3	Casi la mitad de las ocasiones
4	Seguido
5	Siempre
8	Se rehúsa a contestar

If B967 is equal to 1 or B967 is equal to 8, then siga con P B969.

B968. ¿Tuvo sexo vaginal SIN PROTECCIÓN (sin condón) la última vez que tuvo sexo a cambio de algo que necesitaba?

1 Si

0 No

8 Se rehúsa a contestar

9 No corresponde

B969. En los últimos 6 meses, cuando tuvo relaciones sexuales a cambio de algo que necesitaba, ¿qué tan seguido tuvo sexo anal? (Escoja una)

1 Nunca

2 A veces

3 Casi la mitad de las ocasiones

4 Seguido

5 Siempre

8 Se rehúsa a contestar

If B969 is equal to 1 or B969 is equal to 8, then siga con P B971.

B970. ¿Tuvo sexo anal SIN PROTECCIÓN (sin condón) la última vez que tuvo sexo a cambio de algo que necesitaba?

1 Si

0 No

8 Se rehúsa a contestar

9 No corresponde

B971. En los últimos 6 meses, cuando alguien le dió algo que usted necesitaba a cambio de sexo, ¿qué tan seguido se inyectaba drogas con ellos? (Escoja una)

1 Nunca

2 A veces

3 Casi la mitad de las ocasiones

4 Seguido

5 Siempre

8 Se rehúsa a contestar

If B971 is equal to 1 or B971 is equal to 8, then siga con P instrucción antes de B973.

B972. ¿Qué tan seguido compartió la misma jeringa con ellos? (Escoja una)

1 Nunca

2 A veces

3 Casi la mitad de las ocasiones

4 Seguido

5 Siempre

8 Se rehúsa a contestar

LEA: NOTA DE PRECAUCIÓN PARA ENTREVISTADOR: Es posible que las siguientes preguntas haya sido malinterpretadas por algunas mujeres, donde dar.. a cambio de tener sexo contigo se haya confundido con recibir… a cambio de tener sexo contigo.

B973. ¿Alguna vez les ha dado a alguien algo que necesitaban (drogas, dinero, comida, hospedaje, transportación, alcoho o protección)a cambio de tener relaciones sexuales con usted?

1 Si

0 No

8 Se rehúsa a contestar

If B973 is not equal to 1, then siga con P B992.

B974. La/s persona/personas a la que les dió drogas, dinero, comida, hospedaje, transportación, alcohol, o algo que necesitaban eran ¿hombres, mujeres o transgenero? (Marque todas las que apliquen)

__ Hombre

__ Mujer

__ Transgénero

__ Se rehúsa a contestar

B975. ¿En qué ciudad (ciudades) estaba cuando le dió drogas, dinero, comida, hospedaje, transportación, alcohol, o algo que necesitaba a alguien? (Marque todas las que apliquen)

__ Condado de San Diego

__ Tijuana

__ Otra ciudad en EUA

__ Otra ciudad en México

__ Otro

__ Se rehúsa a contestar

If B975E is equal to 1 then siga con P B976

If not, then siga con P instrucción antes de B977.

B976. Por favor especifique en qué otra ciudad (ciudades) estaba cuando le dió drogas, dinero, comida, hospedaje, transportación, alcohol, o algo que necesitaba a alguien?

__ __

If B901 is not equal to 1, then siga con P B992.

B977. En los **últimos 6 meses,** ¿a cuántas personas les ha dado drogas, dinero, comida, hospedaje, transportación, alcohol, o algo a cambio de tener relaciones sexuales con usted?

__ __ __ __ personas

9998 Se rehúsa a contestar

If B977 is equal to 0 or B977 is equal to 9998, then siga con P B992.

B978. En los últimos 6 meses, ¿en qué ciudad (ciudades) estaba cuando le dió drogas, dinero, comida, hospedaje, transportación, alcohol, o algo que necesitaba a alguien? (Marque todas las que apliquen)

__ Condado de San Diego

__ Tijuana

__ Otra ciudad en EUA

__ Otra ciudad en México

__ Otro

__ Se rehúsa a contestar

If B978E is equal to 1 then siga con P B979

If not, then siga con P B980.

B979. Por favor especifique en qué otra ciudad (ciudades) estaba cuando le dió drogas, dinero, comida, hospedaje, transportación, alcohol, o algo que necesitaba a alguien?

__ __

B980. De estas [Respuesta a P B977] personas, ¿cuántas eran hombres?

__ __ __ __

9998 Se rehúsa a contestar

If B980 is greater than B977 and B980 is not equal to 9998 then LEA: "La respuesta del participante debe ser menor o igual a la cantidad de personas a las que les da drogas, dinero, comida, refugio, transporte, alcohol o cualquier otra cosa a cambio de tener relaciones sexuales con usted. Por favor revise las respuestas." and siga con P B980.

If B980 is equal to 0 or B980 is equal to 9998, then siga con P instrucción antes de B984.

B981. ¿Cuántas de estas parejas hombres eran de:EUA-USA**?**

__ __ __ __

9998 Se rehúsa a contestar

If B981 is greater than B980 and B981 is not equal to 9998 then LEA: "La respuesta del participante debe ser menor o igual a la cantidad de hombres a los que les da drogas, dinero, comida, refugio, transporte, alcohol o cualquier otra cosa a cambio de tener relaciones sexuales con usted. Por favor revise las respuestas." and siga con P B981.

If B981 is equal to B980, then siga con P instrucción antes de B984.

B982. ¿Cuántas de estas parejas hombres eran de México**?**

__ __ __ __

9998 Se rehúsa a contestar

If B981 + B982 is greater than B980 and B981 is not equal to 9998 and B982 is not equal to 9998 then LEA: "La respuesta del participante debe ser menor o igual a la cantidad de hombres a los que les da drogas, dinero, comida, refugio, transporte, alcohol o cualquier otra cosa a cambio de tener relaciones sexuales con usted. Por favor revise las respuestas." and siga con P B982.

If B981 + B982 is equal to B980, then siga con P instrucción antes de B984.

B983. ¿Cuántas de estas parejas hombres eran de otros países**?**

__ __ __ __

9998 Se rehúsa a contestar

If B981 + B982 + B983 is greater than B980 and B981 is not equal to 9998 and B982 is not equal to 9998 and B983 is not equal to 9998 then LEA: "La respuesta del participante debe ser menor o igual a la cantidad de hombres a los que les da drogas, dinero, comida, refugio, transporte, alcohol o cualquier otra cosa a cambio de tener relaciones sexuales con usted. Por favor revise las respuestas." and siga con P B983.

If B980 is equal to B977, then siga con P B992.

B984. De estas [Respuesta a P B977] personas, ¿cuántas eran mujeres?

__ __ __ __

9998 Se rehúsa a contestar

If B980 + B984 is greater than B977 and B980 is not equal to 9998 and B984 is not equal to 9998 then LEA: "La respuesta del participante debe ser menor o igual a la cantidad de personas a las que les da drogas, dinero, comida, refugio, transporte, alcohol o cualquier otra cosa a cambio de tener relaciones sexuales con usted. Por favor revise las respuestas." and siga con P B984.

If B984 is equal to 0 or B984 is equal to 9998, then siga con P instrucción antes de B988.

B985. ¿Cuántas de estas parejas mujeres eran de EUA-USA**?**

__ __ __ __

9998 Se rehúsa a contestar

If B985 is greater than B984 and B985 is not equal to 9998 then LEA: "La respuesta del participante debe ser menor o igual a la cantidad de mujeres a las que les da drogas, dinero, comida, refugio, transporte, alcohol o cualquier otra cosa a cambio de tener relaciones sexuales con usted. Por favor revise las respuestas." and siga con P B985.

If B985 is equal to B984, then siga con P instrucción antes de B988.

B986. ¿Cuántas de estas parejas mujeres eran de México**?** __ __ __ __

9998 Se rehúsa a contestar

If B985 + B986 is greater than B984 and B985 is not equal to 9998 and B986 is not equal to 9998 then LEA: "La respuesta del participante debe ser menor o igual a la cantidad de mujeres a las que les da drogas, dinero, comida, refugio, transporte, alcohol o cualquier otra cosa a cambio de tener relaciones sexuales con usted. Por favor revise las respuestas." and siga con P B986.

If B985 + B986 is equal to B984, then siga con P instrucción antes de B988.

B987. ¿Cuántas de estas parejas mujeres eran de otros países**?**

__ __ __ __

9998 Se rehúsa a contestar

If B985 + B986 + B987 is greater than B984 and B985 is not equal to 9998 and B986 is not equal to 9998 and B987 is not equal to 9998 then LEA: "La respuesta del participante debe ser menor o igual a la cantidad de mujeres a las que les da drogas, dinero, comida, refugio, transporte, alcohol o cualquier otra cosa a cambio de tener relaciones sexuales con usted. Por favor revise las respuestas." and siga con P B987.

If B980 + B984 is equal to B977, then siga con P B992.

B988. De estas [Respuesta a P B977] personas, ¿cuántas eran transgéneros?

__ __ __ __

9998 Se rehúsa a contestar

If B980 + B984 + B988 is greater than B977 and B980 is not equal to 9998 and B984 is not equal to 9998 and B988 is not equal to 9998 then LEA: "La respuesta del participante debe ser menor o igual a la cantidad de personas a las que les da drogas, dinero, comida, refugio, transporte, alcohol o cualquier otra cosa a cambio de tener relaciones sexuales con usted. Por favor revise las respuestas." and siga con P B988.

If B988 is equal to 0 or B988 is equal to 9998, then siga con P B992.

B989. ¿Cuántas de estas parejas transgéneros eran de EUA-USA**?**

— — — —

9998 Se rehúsa a contestar

If B989 is greater than B988 and B989 is not equal to 9998 then LEA: "La respuesta del participante debe ser menor o igual a la cantidad de personas transgénero a las que le da drogas, dinero, comida, refugio, transporte, alcohol o cualquier otra cosa a cambio de tener relaciones sexuales con usted. Por favor revise las respuestas." and siga con P B989.

If B989 is equal to B988, then siga con P B992.

B990. ¿Cuántas de estas parejas transgéneros eran de México**?**

— — — —

9998 Se rehúsa a contestar

If B989 + B990 is greater than B988 and B989 is not equal to 9998 and B990 is not equal to 9998 then LEA: "La respuesta del participante debe ser menor o igual a la cantidad de personas transgénero a las que le da drogas, dinero, comida, refugio, transporte, alcohol o cualquier otra cosa a cambio de tener relaciones sexuales con usted. Por favor revise las respuestas." and siga con P B990.

If B989 + B990 is equal to B988, then siga con P B992.

B991. ¿Cuántas de estas parejas transgéneros eran de otros países**?**

— — — —

9998 Se rehúsa a contestar

If B989 + B990 + B991 is greater than B988 and B989 is not equal to 9998 and B990 is not equal to 9998 and B991 is not equal to 9998 then LEA: "La respuesta del participante debe ser menor o igual a la cantidad de personas transgénero a las que le da drogas, dinero, comida, refugio, transporte, alcohol o cualquier otra cosa a cambio de tener relaciones sexuales con usted. Por favor revise las respuestas." and siga con P B991.

B992. ¿Alguna vez alguien lo/la ha forzado a tener relaciones sexuales por medio de fuerza física o presión emocional? (Recuerde, puede ser sexo vaginal, oral o anal)

1 Si

0 No

8 Se rehúsa a contestar

If B992 is not equal to 1, then siga con P instrucción antes de B1001.

B993. ¿Qué edad tenías la PRIMERA VEZ que alguien te forzó a tener sexo?

__ __ años

98 Se rehúsa a contestar

If B993 is greater than A1 and B993 is not equal to 98 then LEA: "El participante ha respondido con un período de tiempo mayor que su edad. Por favor revise las respuestas." and siga con P B993.

B994. La PRIMERA VEZ que alguien lo/la forzó a tener sexo ¿quién lo forzó a tener relaciones sexuales? (Marque todas las que apliquen)

__ Familiar

__ Esposo (a)

__ Amigo (a)

__ Conocido

__ Desconocido

__ Otra Pareja Sexual

__ Cliente Sexual

__ Padrote

__ Policía / Figura Autoritaria

__ Otro

__ Se rehúsa a contestar

If B994J is not equal to 1, then siga con P instrucción antes de B996.

B995. Por favor especifique quién otro:

____

If B901 is not equal to 1, then siga con P instrucción antes de B1001.

B996. En los **últimos 6 meses,** ¿alguien lo ha forzado a tener relaciones sexuales por medio de fuerza física o presión emocional? (Recuerde, puede ser sexo vaginal, oral o anal)

1 Si

0 No

8 Se rehúsa a contestar

If B996 is not equal to 1, then siga con P instrucción antes de B1001.

B997. ¿En qué ciudad fuiste forzado/a a tener sexo en los últimos 6 meses? (Marque todas las que apliquen)

__ Condado de San Diego

__ Tijuana

__ Otra ciudad en EUA

__ Otra ciudad en México

__ Otro

__ Se rehúsa a contestar

If B997E is not equal to 1, then siga con P B999.

B998. Por favor especifique en qué otra ciudad:

____

B999. En los últimos 6 meses, ¿quién lo/a forzó a tener relaciones sexuales? (Marque todas las que apliquen)

__ Familiar

__ Esposo (a)

__ Amigo (a)

__ Conocido

__ Desconocido

__ Otra Pareja Sexual

__ Cliente Sexual

__ Padrote

__ Policía / Figura Autoritaria

__ Otro

__ Se rehúsa a contestar

If B999J is not equal to 1, then siga con P instrucción antes de B1001.

B1000. Por favor especifique quién otro lo ha forzado a tener relaciones sexuales por medio de fuerza física o presión emocional:

____

LEA: LEA: Ahora le voy a preguntar sobre las veces que ha tenido relaciones sexuales con agentes del orden. Estos pueden haber sido encuentros que fueron voluntarios o involuntarios. Estas preguntas son sobre encuentros ocurrieron en México y no en los Estados Unidos o cualquier otro país.

B1001. ¿Alguna vez ha tenido sexo con un agente del orden?

1 Si

0 No

8 Se rehúsa a contestar

If B1001 is not equal to 1 and B901 is not equal to 1, then siga con P final de la entrevista.

B1002. ¿Cuántas veces ha tenido relaciones sexuales con agentes del orden en los **últimos 6 meses**?

__ __ __ __ # de veces

9998 Se rehúsa a contestar

If B1002 is equal to 0 or B1002 is equal to 9998, then siga con P final de la entrevista.

B1003. De las [Respuesta a P B1002] veces que ha tenido relaciones sexuales con agentes del orden **en los últimos 6 meses,** ¿cuántas veces fue para evadir arresto u otro castigo?

__ __ __ __ # de veces

9998 Se rehúsa a contestar

If B1003 is greater than B1002 and B1003 is not equal to 9998 then LEA: "La respuesta del participante debe ser menor o igual al número de veces que tuvo relaciones sexuales con un agente de la ley. Por favor revise las respuestas." and siga con P B1003.

If B1003 is equal to 0 or B1003 is equal to 9998, then siga con P final de la entrevista.

B1004. De esas [Respuesta a P B1002] veces, ¿cuántas veces ocurrió esto en EUA-USA?

__ __ __ __

9998 Se rehúsa a contestar

If B1004 is greater than B1003 and B1004 is not equal to 9998 then LEA: "La respuesta del participante debe ser menor o igual al número de veces que tuvo relaciones sexuales con un oficial de la ley para evitar el arresto u otro castigo. Por favor revise las respuestas." and siga con P B1004.

If B1004 is equal to B1003, then siga con P final de la entrevista.

B1005. De esas [Respuesta a P B1002] veces, ¿cuántas veces ocurrió esto en México?

__ __ __ __

9998 Se rehúsa a contestar

If B1004 + B1005 is greater than B1003 and B1004 is not equal to 9998 and B1005 is not equal to 9998 then LEA: "La respuesta del participante debe ser menor o igual al número de veces que tuvo relaciones sexuales con un oficial de la ley para evitar el arresto u otro castigo. Por favor revise las respuestas." and siga con P B1005.

LEA: FINAL DE LA ENTREVISTA.
LEA: Este es el final del cuestionario. Muchas gracias por su valioso tiempo y cooperación.

DISQ1 = If(A1 is less than 18 or A1 is equal to 998 or A3 is greater than 30 or A3 is equal to 9998 or A7 is equal to 3 or A7 is equal to 8, 1, 0)

DISQ2 = If(A7 is equal to 1 and (A8 is equal to 7 or A8 is equal to 8) or A7 is equal to 2 and A12 is not equal to 1, 1, 0)

DISQ3 = If(GROUP is equal to 0, 1, 0)

DISQALL = DISQ1 + DISQ2 + DISQ3

If DISQALL is greater than 0 then siga con P final de la entrevista

If not, then siga con P final de la entrevista.

LEA: ENTREVISTADOR: EL PARTICIPANTE NO CALIFICA! FINAL DE LA ENTREVISTA. Agradezca al participante por su tiempo.

LEA: Fin de entrevista.

La Frontera Project

Supplemental Survey

Disclaimer: This survey has been formatted for programming into QDS and is not intended to be administered on pencil and paper

PI: Steffanie Strathdee
10-18-2021

LaFrontera.Supplemental.20210820 (English)

READ: La Frontera
Supplemental Interview
English

VERSION = 20210820

Q1. Interviewer: Please enter interview date. __ __ / __ __ / __ __ __ __ mm / dd / yyyy

If Q1 is greater than TODAY and TODAY is not equal to "missing" then READ: "That date occurs in the future. Please check the entry." and skip to Q1.

Q2. Interviewer Initials. __

Q3. City of the Interview
Interviewer, it is crucial that you enter the interview location correctly!
(Choose one)

1 San Diego

2 Tijuana

Q4. Interviewer, how will this interview be conducted? (Choose one)

1 In Person, Inside

2 In Person, Outside

3 Video call (e.g., zoom, skype)

4 Audio Call (e.g., phone, zoom)

5 Other

8 Refuse to Answer

If Q4 is not equal to 5, then skip to Q6.

Q5. Please, specify Other.

__ __

Q6. Participant ID Number (e.g., LF001). — — — — —

ID1 = SubString(Q6, 0, 1)

ID2 = SubString(Q6, 1, 1)

ID3 = SubString(Q6, 2, 1)

ID4 = SubString(Q6, 3, 1)

ID5 = SubString(Q6, 4, 1)

IDSTUDY = Concatenated(ID1, ID2)

If IDSTUDY is not equal to LF then READ: "Check ID." and skip to Q6.

If ID3 is not equal to 0 and ID3 is not equal to 1 and ID3 is not equal to 2 and ID3 is not equal to 3 and ID3 is not equal to 4 and ID3 is not equal to 5 and ID3 is not equal to 6 and ID3 is not equal to 7 and ID3 is not equal to 8 and ID3 is not equal to 9 or ID4 is not equal to 0 and ID4 is not equal to 1 and ID4 is not equal to 2 and ID4 is not equal to 3 and ID4 is not equal to 4 and ID4 is not equal to 5 and ID4 is not equal to 6 and ID4 is not equal to 7 and ID4 is not equal to 8 and ID4 is not equal to 9 or ID5 is not equal to 0 and ID5 is not equal to 1 and ID5 is not equal to 2 and ID5 is not equal to 3 and ID5 is not equal to 4 and ID5 is not equal to 5 and ID5 is not equal to 6 and ID5 is not equal to 7 and ID5 is not equal to 8 and ID5 is not equal to 9 then READ: "Check ID." and skip to Q6.

Q7. Please enter the SCREENER ID of the Subject (e.g., LF0001).
Note to the Interviewer: Please do not confuse the screener number with the Subject ID number!

— — — — — —

SCRID1 = SubString(Q7, 0, 1)

SCRID2 = SubString(Q7, 1, 1)

SCRID3 = SubString(Q7, 2, 1)

SCRID4 = SubString(Q7, 3, 1)

SCRID5 = SubString(Q7, 4, 1)

SCRID6 = SubString(Q7, 5, 1)

IDSCREEN = Concatenated(SCRID1, SCRID2)

If IDSCREEN is not equal to LF then READ: "Check SCRID." and skip to Q7.

If SCRID3 is not equal to 0 and SCRID3 is not equal to 1 and SCRID3 is not equal to 2 and SCRID3 is not equal to 3 and SCRID3 is not equal to 4 and SCRID3 is not equal to 5 and SCRID3 is not equal to 6 and SCRID3 is not equal to 7 and SCRID3 is not equal to 8 and SCRID3 is not equal to 9 or SCRID4 is not equal to 0 and SCRID4 is not equal to 1 and SCRID4 is not equal to 2 and SCRID4 is not equal to 3 and SCRID4 is not equal to 4 and SCRID4 is not equal to 5 and SCRID4 is not equal to 6 and SCRID4 is not equal to 7 and SCRID4 is not equal to 8 and SCRID4 is not equal to 9 or SCRID5 is not equal to 0 and SCRID5 is not equal to 1 and SCRID5 is not equal to 2 and SCRID5 is not equal to 3 and SCRID5 is not equal to 4 and SCRID5 is not equal to 5 and SCRID5 is not equal to 6 and SCRID5 is not equal to 7 and SCRID5 is not equal to 8 and SCRID5 is not equal to 9 or SCRID6 is not equal to 0 and SCRID6 is not equal to 1 and SCRID6 is not equal to 2 and SCRID6 is not equal to 3 and SCRID6 is not equal to 4 and SCRID6 is not equal to 5 and SCRID6 is not equal to 6 and SCRID6 is not equal to 7 and SCRID6 is not equal to 8 and SCRID6 is not equal to 9 then READ: "Check ID." and skip to Q6.

Q8. Interview Location **(Choose one)** (Choose one)

1 Tijuana

2 San Diego

3 Other

If Q8 is equal to 3 then skip to Q9

If not, then skip to instruction before Q11.

Q9. Specify the Interview Location (i.e. name of city).

____

Q10. Specify the Interview Place in [Response to Q9].

____

If Q8 is equal to 1 then skip to Q11

If not, then skip to instruction before Q13.

Q11. Interview Place in Tijuana. **(Choose one)** (Choose one)

1 Tijuana 1 Zone Norte - El Mapa

2 Tijuana 2 Coahuila Office

3 Tijuana 3

4 Rehab

5 Jail

6 Other

If Q11 is equal to 6 then skip to Q12

If not, then skip to instruction before Q13.

Q12. Specify the Interview Place in Tijuana.

— —
— —
— — — — — — — — — — — — — — — — — — — —

If Q8 is equal to 2 then skip to Q13

If not, then skip to Q15.

Q13. Interview Place in San Diego. **(Choose one)** (Choose one)

1 San Diego 1

2 San Diego 2

3 San Diego 3

4 Rehab

5 Jail

6 Other

If Q13 is equal to 6 then skip to Q14

If not, then skip to Q15.

Q14. Specify the Interview Place in San Diego.

— —
— —
— — — — — — — — — — — — — — — — — — — —

Q15. In what country do you live? **(Choose one)** (Choose one)

1 United States

2 Mexico

3 Other

8 Refuse to Answer

If Q15 is equal to 3 then skip to Q16

If not, then skip to instruction before Q17.

Q16. Specify the country in which you live.

— —
— —
— — — — — — — — — — — — — — — — — — — —

If Q15 is equal to 1 then skip to Q17

If not, then skip to instruction before Q19.

Q17. In what region of San Diego County do you live? **(Choose one)** (Choose one)

1 City of San Diego

2 East County

3 North County Coastal

4 North County Inland

5 South Bay

6 Other San Diego region

7 I do not live in San Diego County

8 Refuse to Answer

If Q17 is not equal to 6, then skip to instruction before Q19.

Q18. Please specify what region you live in.

____

If Q15 is equal to 2 then skip to Q19

If not, then skip to instruction before Q21.

Q19. What city do you live in? (Choose one) (Choose one)

1 Tijuana

2 Mexicali

3 Ensenada

4 Rosarito

5 Other

8 Refuse to Answer

If Q19 is not equal to 5, then skip to instruction before Q21.

Q20. Please specify the name of the city where you reside.

— —
— —
— —
— —
— —

If Q15 is equal to 1 then skip to instruction before Q21

If not, then skip to instruction before Q25.

If Q8 is equal to 2 then skip to Q21

If not, then skip to Q22.

Q21.	Have you ever been to Mexico?	1	Yes
		0	No
		8	Refuse to Answer

If Q21 is not equal to 1, then skip to instruction before Q25.

Q22.	Have you ever used any drugs in Mexico?	1	Yes
		0	No
		8	Refuse to Answer

If Q22 is not equal to 1, then skip to instruction before Q24.

Q23.	Have you ever injected drugs in Mexico?	1	Yes
		0	No
		8	Refuse to Answer

If Q23 is not equal to 1, then skip to instruction before Q25.

Q24. When was the last time you injected drugs in Mexico?

__ __ / __ __ / __ __ __ __	mm / dd / yyyy
2098	Refuse to Answer (Year)

If Q24 is not equal to 2098 and Q24 is not equal to "skipped" then skip to instruction before Q25

If not, then skip to instruction before Q25.

INJTMEX = TODAY - Q24

If Q8 is equal to 1 and Q15 is equal to 2 then skip to Q25

If not, then skip to instruction before Q29.

Q25. Have you ever been to the United States?

1	Yes
0	No
8	Refuse to Answer

If Q25 is not equal to 1, then skip to instruction before Q29.

Q26. Have you ever used any drugs in the US?

1	Yes
0	No
8	Refuse to Answer

If Q26 is not equal to 1, then skip to instruction before Q28.

Q27. Have you ever injected drugs in the US?

1	Yes
0	No
8	Refuse to Answer

If Q27 is not equal to 1, then skip to instruction before Q29.

Q28. When was the last time you injected drugs in the US?

__ __ / __ __ / __ __ __ __	mm / dd / yyyy
2098	Refuse to Answer (Year)

If Q28 is not equal to 2098 and Q28 is not equal to "skipped" then skip to instruction before Q29

If not, then skip to instruction before Q29.

INJTUS = TODAY - Q28

NDT_SD = If(Q8 is equal to 2 and (Q17 is equal to 1 or Q17 is equal to 2 or Q17 is equal to 3 or Q17 is equal to 4 or Q17 is equal to 5 or Q17 is equal to 6) and (Q21 is equal to 0 or Q22 is equal to 0), 1, 0)

NDT_TJ = If(Q8 is equal to 1 and Q19 is equal to 1 and (Q25 is equal to 0 or Q26 is equal to 0), 1, 0)

DT_SD = If(NDT_SD is equal to 0 and NDT_TJ is equal to 0, 1, 0)

READ: INTERVIEWER, PLEASE READ THE FOLLOWING TEXT ALOUD TO THE RESPONDENT: This supplemental interview is part of the research study about HIV/HCV, SARSCoV-2 and overdose among people who inject drugs in San Diego and Tijuana. The questions we will be asking complement the questions we asked you during your first visit and are related to some basic information about you, your SARSCoV-2 and TB health, your friends and people you know and utilization of health and other services, and your attitudes towards some of these issues. I know some of the questions we will ask may be a little uncomfortable for you to answer. I want to remind you like in your first visit, this interview is confidential, and we will not give any of your personal information to anyone outside of this project. Nothing that you tell us can be traced back to you as an individual. It is important that you know that your participation in this interview is entirely voluntary. You do not need to answer a question if you do not want to, and you may stop the interview at any point. Do you have any questions before we begin?

Q29. What is your date of birth?

__ __ / __ __ / __ __ __ __	mm / dd / yyyy
2098	Refuse to Answer (Year)

AGE = Age(Q29, TODAY)

Q30. What sex was listed on your birth certificate? (Choose one) (Choose one)

1	Male
2	Female
3	Intersex
8	Refuse to Answer

WATER

READ: READ TO THE RESPONDENT: Now I am going to ask some questions about your use and access to water in your daily life.

Q31. Over the past 6 months, which one of these sources of drinking water you used? **Interviewer: show images of water sources and select all that apply.** (Check all that apply)

__ Private Tap (piped to your household)

__ Private tap (piped to a neighbor, family member, friend)

__ Public tap (park/gas station/outside store)

__ Irrigation water (public parks)

__ Unprotected well

__ Protected well

__ Unprotected spring

__ Protected spring

__ Rainwater

__ Delivered water (truck)

__ Packaged (bottled/sachet/water dispenser)

__ Surface water (river/lake/canal)

__ Tubes/holes inside the Tijuana river canal (lloraderos)

__ No water sources

__ Other

__ Refuse to Answer

If Q31N is equal to 1 and Q31 is not equal to 98 and Q31 is greater than 1 then READ: "You can only choose the response "No water sources" by itself. Please check responses." and skip to Q31.

If Q31O is equal to 1 then skip to Q32

If not, then skip to Q33.

Q32. You said that you had access to other water sources in the past 6 months. Please specify.

— —

Q33. What is your main source for drinking water (most common)? **Interviewer: show images of water sources and select only one**. (Choose one)

01 Private tap (piped to your household)

02 Private tap (piped to a neighbor, family member, friend)

03 Public tap (park/gas station/outside store)

04 Irrigation water (public parks)

05 Unprotected well

06 Protected well

07 Unprotected spring

08 Protected spring

09 Rainwater

10 Delivered water (truck)

11 Packaged (bottled/sachet/water dispenser)

12 Surface water (river/lake/canal)

13 Tubes/holes inside the Tijuana river canal (lloraderos)

14 No water sources

15 Other

98 Refuse to Answer

If Q33 is equal to 15 then skip to Q34

If not, then skip to Q35.

Q34. Please specify your main source for drinking water.

— —
— —
— —

Q35. Is this main source of drinking water always available to you? (Choose one) (Choose one)

1 Yes, always available

2 Most of the time

3 Some of the time

4 No, water is rarely available

8 Refuse to Answer

Q36. How long does it take you to secure drinking water from your main drinking water source (go there, get water, and come back)? **Interviewer, if the respondent indicates that water is always available, enter 0.**

— — — HOURS

— — — MINUTES

998 Refuse to Answer (Minutes)

Q37. Since the COVID-19 epidemic started in March 2020, did water availability change for you? (Choose one) (Choose one)

0 No, it is the same

1 Yes, it got worse

2 Yes, it improved

8 Refuse to Answer

Q38. In the last week, what was your main (most common) beverage when you felt thirsty? (Choose one)

01	Regular Water
02	Sparkling Water
03	Milk
04	Coffee
05	Tea
06	Juice/sweetened flavored drink
07	Soda
08	Alcoholic beverages
09	None
10	Other
98	Refuse to Answer

If Q38 is equal to 10 then skip to Q39

If not, then skip to Q40.

Q39. Please specify your main beverage.

— —
— —
— — — — — — — — — — — — — — — — — — — —

Q40. In the last week, how often did you feel thirsty and were without water? (Choose one) (Choose one)

1	Multiple times per day
2	Daily
3	Multiple times in the week
4	Once
5	Never
8	Refuse to Answer

Q41. In the last week, how many glasses of water did you drink per day? (Choose one) (Choose one)

01	1 glass=250 ml/8 oz
02	2 glasses=500 ml/16 oz
03	3 glasses=750 ml/24 oz
04	4 glasses=1 ltr/32 oz
05	5 glasses=1.25 ltr/40 oz
06	6 glasses=1.5 ltr/48 oz
07	7 glasses=1.75 ltr/52oz
08	8 glasses=2 ltr/64 oz
09	9 glasses=2.25 ltr/72 oz
10	More than 9 glasses per day
98	Refuse to Answer

If Q41 is equal to 10 then skip to Q42

If not, then skip to Q43.

Q42. Please specify how many glasses of water did you drink per day? __ __ glasses

98 Refuse to Answer

Q43. In the last week, how much money did you spend on drinking water? **Interviewer, please enter the amount and on the next screen you'll be prompted to enter the currency.**

__ __ __ __ __

99998 Refuse to Answer

If Q43 is not equal to 99998 and Q43 is not equal to 0 then skip to Q44

If not, then skip to Q45.

Q44. Interviewer, please enter the **currency.** (Choose one)

1	US Dollars
2	Mexican Pesos
8	Refuse to Answer

Q45. Over the past 6 months, what water sources did you use for handwashing? **Interviewer: show images of water sources and select all that apply.** (Check all that apply)

__ Private tap (piped to your household)

__ Private tap (piped to a neighbor, family member, friend)

__ Public tap (park/gas station/outside store)

__ Irrigation water (public parks)

__ Unprotected well

__ Protected well

__ Unprotected spring

__ Protected spring

__ Rainwater

__ Delivered water (truck)

__ Packaged (bottled/sachet/water dispenser)

__ Surface water (river/lake/canal)

__ Tubes/holes inside the Tijuana river canal (lloraderos)

__ No water sources

__ Other

__ Refuse to Answer

If Q45N is equal to 1 and Q45 is not equal to 98 and Q45 is greater than 1 then READ: "You can only choose the response "No water sources" by itself. Please check responses." and skip to Q45.

If Q45O is equal to 1 then skip to Q46

If not, then skip to Q47.

Q46. You said that you use another source of water for handwashing. Please specify.

____

Q47. What is your main source of water for handwashing (most common)? **Interviewer: read out loud to the respondent and select only one** (Choose one)

01	Private tap (piped to your household)
02	Private tap (piped to a neighbor, family member, friend)
03	Public tap (park/gas station/outside store)
04	Irrigation water (public parks)
05	Unprotected well
06	Protected well
07	Unprotected spring
08	Protected spring
09	Rainwater
10	Delivered water (truck)
11	Packaged (bottled/sachet/water dispenser)
12	Surface water (river/lake/canal)
13	Tubes/holes inside the Tijuana river canal (lloraderos)
14	No water sources
15	Other
98	Refuse to Answer

If Q47 is equal to 15 then skip to Q48

If not, then skip to Q49.

Q48. Please specify your main source of water for handwashing.

— —
— —
— — — — — — — — — — — — — — — — — — — —

Q49. How often is this main source for handwashing available to you? (Choose one)

1	Always
2	Usually
3	Sometimes
4	Rarely
5	Never
8	Refuse to Answer

Q50. How often is soap available to you? (Choose one)

1	Always
2	Usually
3	Sometimes
4	Rarely
5	Never
8	Refuse to Answer

Q51. How often are you able to wash your hands with soap and water on an average day? (Choose one)

1	Always
2	Usually
3	Sometimes
4	Rarely
5	Never
8	Refuse to Answer

Q52. Since the COVID-19 epidemic started, did soap availability change for you? (Choose one)

0	No, it is the same
1	Yes, it got worse
2	Yes, it improved
8	Refuse to Answer

Q53. How often is alcohol-based hand rub available to you? (Choose one)

1	Always
2	Usually
3	Sometimes
4	Rarely
5	Never
8	Refuse to Answer

Q54. In the last week, how often did you wash your hands (with soap and water) after a bowel movement? (Choose one)

1	Always
2	Usually
3	Sometimes
4	Rarely
5	Never
8	Refuse to Answer

Q55. In the last week, how often did you wash your hands (soap and water) before eating? (Choose one)

1	Always
2	Usually
3	Sometimes
4	Rarely
5	Never
8	Refuse to Answer

Q56. In the last week, how often did you wash your hands (soap and water) before preparing food? (Choose one)

1	Always
2	Usually
3	Sometimes
4	Rarely
5	Never
8	Refuse to Answer

Q57. What is your main source of water for bathing (most common)? **Interviewer: show images of water sources and select only one.** (Choose one)

01	Private tap (piped to your household)
02	Private tap (piped to a neighbor, family member, friend)
03	Public tap (park/gas station/outside store)
04	Irrigation water (public parks)
05	Unprotected well
06	Protected well
07	Unprotected spring
08	Protected spring
09	Rainwater
10	Delivered water (truck)
11	Packaged (bottled/sachet/water dispenser)
12	Surface water (river/lake/canal)
13	Tubes/holes inside the Tijuana river canal (lloraderos)
14	No water sources
15	Other
98	Refuse to Answer

If Q57 is equal to 15 then skip to Q58

If not, then skip to Q59.

Q58. Please specify your main source of water for bathing.

____

Q59. In the last week, how many times did you shower/bathe? __ __ times

98 Refuse to Answer

Q60. What is your main water source for cleaning wounds/abscesses? **Interviewer: show images of water sources.** (Choose one) (Choose one)

01	Private tap (piped to your household)
02	Private tap (piped to a neighbor, family member, friend)
03	Public tap (park/gas station/outside store)
04	Irrigation water (public parks)
05	Unprotected well
06	Protected well
07	Unprotected spring
08	Protected spring
09	Rainwater
10	Delivered water (truck)
11	Packaged (bottled/sachet/water dispenser)
12	Surface water (river/lake/canal)
13	Tubes/holes inside the Tijuana river canal (lloraderos)
14	No water sources
15	Other
98	Refuse to Answer

If Q60 is equal to 15 then skip to Q61

If not, then skip to Q62.

Q61. You said that you use another main source of water for cleaning wounds/abscesses. Please specify.

— —
— —
— — — — — — — — — — — — — — — — — — — —

Q62. What is your main water source for preparing drugs for injection? **Interviewer: show images of water sources and select one** (Choose one) (Choose one)

01 Private tap (piped to your household)

02 Private tap (piped to a neighbor, family member, friend)

03 Public tap (park/gas station/outside store)

04 Irrigation water (public parks)

05 Unprotected well

06 Protected well

07 Unprotected spring

08 Protected spring

09 Rainwater

10 Delivered water (truck)

11 Packaged (bottled/sachet/water dispenser)

12 Surface water (river/lake/canal)

13 Tubes/holes inside the Tijuana river canal (lloraderos)

14 No water sources

15 Other

98 Refuse to Answer

If Q62 is equal to 15 then skip to Q63

If not, then skip to Q64.

Q63. You said that you use another main source of water for preparing drugs for injection. Please specify.

— —
— —
— — — — — — — — — — — — — — — — — — — —

Q64. In the last week, how much money did you spend on personal hygiene (soap and water for hygiene, bathing costs, laundry costs, menstrual hygiene)? **Interviewer, please enter the amount and on the next screen you'll be prompted to enter the currency.**

— — — — —	
99998	Refuse to Answer

If Q64 is not equal to 99998 and Q64 is not equal to 0 then skip to Q65

If not, then skip to instruction before Q66.

Q65. Interviewer, please enter the **currency.** (Choose one)

1	US Dollars
2	Mexican Pesos
8	Refuse to Answer

If Q30 is equal to 2 or Q30 is equal to 3 then skip to Q66

If not, then skip to instruction before Q67.

Q66. Have you ever had menstrual periods?

1	Yes
0	No
8	Refuse to Answer

If Q66 is equal to 1 then skip to Q67

If not, then skip to instruction before Q69.

Q67. When (how long ago) was your last menstrual period? Interviewer, if the participant says that they are currently on their period, enter 0 in the "days" field.

— — —	YEARS
— — —	MONTHS
— — —	DAYS
997	Don't Know (Months)
998	Refuse to Answer (Months)

Q68. During your last menstrual period, which hygiene materials did you use? **Interviewer, check all that apply.** (Check all that apply)

__ Cloth

__ Reusable sanitary pads

__ Disposable sanitary pads

__ Tampons

__ Menstrual cup

__ Toilet paper

__ Underwear alone

__ Nothing

__ Other

__ N/A (I don't have a period)

__ Refuse to Answer

If Q68I is equal to 1 then skip to Q69

If not, then skip to Q70.

Q69. Please specify which hygiene materials you used during your last menstrual period.

____
____
____

Q70. In the last 6 months, what kind of toilet did you use? **Interviewer, check all that apply.** (Check all that apply)

__ Flush/Pour-flush

__ Pit latrine with slab

__ Pit latrine without slab/open pit

__ Composting toilet

__ Container based sanitation

__ Bucket

__ Hanging toilet/latrine

__ No facility (open defecation)

__ Other

__ Refuse to Answer

If Q70I is equal to 1 then skip to Q71

If not, then skip to Q72.

Q71. Please specify what kind of toilet did you use in the past 6 months.

____

Q72. What kind of toilet do you use most commonly? (Choose one)

01	Flush/Pour-flush
02	Pit latrine with slab
03	Pit latrine without slab/open pit
04	Composting toilet
05	Container based sanitation
06	Bucket
07	Hanging toilet/latrine
08	No facility (open defecation)
09	Other
98	Refuse to Answer

If Q72 is equal to 9 then skip to Q73

If not, then skip to Q74.

Q73. Please specify what kind of toilet you use most commonly.

———

Q74. Is this main toilet shared with members of other households that you know or the general public? (Choose one)

1	Shared with more than one household that I know
2	General public
3	Not shared
8	Refuse to Answer
9	Not Applicable

Q75. How often is this main toilet available (open and not locked)? (Choose one)

1	Always
2	Usually
3	Sometimes
4	Rarely
5	Never
8	Refuse to Answer
9	Not Applicable

Q76. How often is this main toilet functional? (Choose one)

1	Always
2	Usually
3	Sometimes
4	Rarely
5	Never
8	Refuse to Answer
9	Not Applicable

Q77. How often is this main toilet private? (Choose one)

1	Always
2	Usually
3	Sometimes
4	Rarely
5	Never
8	Refuse to Answer
9	Not Applicable

Q78. How long does it take to walk to the main toilet sanitation type? **Interviewer, if the respondent indicates that they don't have to walk to the main sanitation facility, enter 0.**

__ __ __	HOURS
__ __ __	MINUTES
998	Refuse to Answer (Minutes)
999	Not Applicable (Minutes)

Q79. Have you experienced harassment or violence when using the toilet?

1	Yes
0	No
8	Refuse to Answer
9	Not Applicable

If Q79 is equal to 1 then skip to Q80

If not, then skip to instruction before Q81.

Q80. What types of harassment or violence have you had when using the toilet? **Interviewer, select all that apply.** (Check all that apply)

__	physical assault
__	sexual assault
__	verbal assault
__	robbery
__	arrest
__	other type of harassment/violence
__	Refuse to Answer
__	Not Applicable

If Q80F is equal to 1 then skip to Q81

If not, then skip to Q82.

Q81. Please specify what other types of harassment or violence have you had when using the toilet?

—
—
— — — — — — — — — — — — — — — — — — — —

Q82. In the last week, how many times did you defecate in the open? __ __ __ times

998 Refuse to Answer

Q83. In the last week, how much money did you spend on sanitation? **Interviewer, please enter the amount and on the next screen, you'll be prompted to enter to currency.**

— — — — —

99998 Refuse to Answer

If Q83 is not equal to 99998 and Q83 is not equal to 0 then skip to Q84

If not, then skip to instruction before Q85.

Q84. Interviewer, please enter the **currency.** (Choose one)

1 US Dollars

2 Mexican Pesos

8 Refuse to Answer

SARSCoV-2 COMPONENT: General Impact

READ: READ TO THE RESPONDENT: COVID-19 is a new disease, caused be a novel (or new) coronavirus that has not previously been seen in humans. I'm going to ask you questions about signs or symptoms you might have experienced in the last two weeks. We will also ask you questions about your daily activities, employment and adjusting to the current situation.

READ: READ TO THE RESPONDENT: Now we are going to ask you some questions about how COVID-19 and changes in your community has affected your life and your health.

Q85. What are you doing/did you do during the COVID-19 pandemic to protect yourself or other people? **Interviewer: Read the options out loud to the respondent and select all that apply.** (Check all that apply)

__ Prac[illegible]

__ Isola[illegible]

__ Cari[illegible]

__ Wor[illegible]

__ Not [illegible]

__ Follo[illegible]

__ Char[illegible]

__ Incre[illegible]

__ Cove[illegible]

__ Avoi[illegible]

__ No c[illegible]

__ Refu[illegible]

If Q85L is equal to 1 and Q85 is greater than 1 then READ: ""No changes to my life or behavior" cannot be selected in conjunction with other options. You are redirected to make corrections." and skip to Q85.

Q86. How much is/did the COVID-19 pandemic impact your day-to-day life? (Choose one)

1 Not at all

2 A little

3 Much

4 Very Much

5 Extremely

8 Refuse to Answer

Q87. In the last 2 weeks, because of the COVID-19 pandemic, I have:
Interviewer read out and select all that apply. If none of the options apply, move on to the next screen. (Check all that apply)

__ Stocked up on alcohol

__ Stocked up on illicit drugs

__ Stocked up on harm reduction supplies (clean needles, works)

__ Avoided sharing cigarettes, joints or e-cigarettes/vapes (more than usual)

__ Avoided sharing crack pipes, meth pipes or bongs (more than usual)

__ Avoid sharing drinks with others (more than usual)

__ Avoided shelters

__ Avoided needle exchange sites

__ Avoided picking up methadone or buprenorphine

__ Avoided picking up other medications

__ Avoided healthcare (e.g., clinical care appointments, hospitals, health clinics)

__ Stopped smoking

__ Accessed treatments for alcohol use disorder

__ Other

__ Refuse to Answer

If Q87N is equal to 1 then skip to Q88

If not, then skip to Q89.

Q88. Please specify what else you have done in the last 2 weeks because of the COVID-19 pandemic.

____

Q89. Has your access to food been impacted in the last 2 weeks? (Check all that apply)

__ No, [illegible]

__ Yes, [illegible]

__ Yes, [illegible]

__ Yes, [illegible]

__ Yes, [illegible]

__ Refu[illegible]

If Q89A is equal to 1 and Q89 is greater than 1 then READ: "You selected the "No" option and also an "Yes" option. Please go back and revise the answer." and skip to Q89.

If Q89F is equal to 1 then skip to Q90

If not, then skip to instruction before Q91.

Q90. Please specify how else has your access to food has been impacted.

____

SARSCoV-2 COMPONENT: Food Insecurity from USDA Adult Food Insecurity Survey

READ: READ TO THE RESPONDENT: Now I'm going to read you several statements that people have made about their food situation. For these statements, please tell me whether the statement was often true, sometimes true, or never true for (you/your household), since the COVID epidemic began in March 2020.

Q91. The first statement is: I worried whether (my/our) food would run out before I got money to buy more. Was that often true, sometimes true, or never true for you since the COVID epidemic began in March 2020? (Choose one)

1 Often true

2 Sometimes true

3 Never true

8 Refuse to Answer

Q92. The food that I bought just didn't last, and I didn't have money to get more. (Choose one)

1 Often true

2 Sometimes true

3 Never true

8 Refuse to Answer

Q93. I couldn't afford to eat balanced meals. (Choose one)

1 Often true

2 Sometimes true

3 Never true

8 Refuse to Answer

Q94. Since the COVID epidemic began in March 2020, did you ever cut the size of your meals or skip meals because there wasn't enough money for food?

1 Yes

0 No

8 Refuse to Answer

If Q94 is equal to 1 then skip to Q95

If not, then skip to Q96.

Q95. How often did this happen? (Choose one)

1 Almost every month

2 Some months but not every month

3 Only 1 or 2 months

8 Refuse to Answer

Q96. Since the COVID epidemic began in March 2020, did you ever eat less than you felt you should because there wasn't enough money for food?

1 Yes

0 No

8 Refuse to Answer

Q97. Since the COVID epidemic began in March 2020, were you ever hungry but didn't eat because there wasn't enough money for food?

1 Yes

0 No

8 Refuse to Answer

Q98. Since the COVID epidemic began in March 2020, did you lose weight because there wasn't enough money for food?

1 Yes

0 No

8 Refuse to Answer

Q99. Since the COVID epidemic began in March 2020, did you ever not eat for a whole day because there wasn't enough money for food?

1 Yes

0 No

8 Refuse to Answer

If Q99 is equal to 1 then skip to Q100

If not, then skip to Q101.

Q100. How often did this happen? (Choose one)

1 Almost every month

2 Some months but not every month

3 Only 1 or 2 months

8 Refuse to Answer

Q101. Do you follow a special diet (e.g. low cholesterol, vegan, vegetarian, paleo, weight loss, organic, etc.)?

1 Yes

0 No

8 Refuse to Answer

If Q101 is equal to 1 then skip to Q102

If not, then skip to Q103.

Q102. Please, specify a diet.

____

Q103. Do you have any of the following chronic conditions?
Interviewer, read the options to the respondent and check all that apply. If none apply, don't check anything and move on to the next screen. (Check all that apply)

__ Seasonal allergies

__ Asthma or other lung problems

__ Heart problems

__ Kidney problems

__ Immune disorder

__ Diabetes or high blood sugar

__ Cancer

__ Arthritis

__ Frequent or very bad headaches

__ Epilepsy or seizures

__ Serious stomach or bowel problems

__ Serious acne or skin problems

__ Hypertension

__ Chronic Obstructive Pulmonary Disease (COPD)

__ Congestive Heart Failure

__ Atrial Fibrillation or Atrial Flutter

__ Heart disease / Myocardial infarction

__ Stroke

__ Any autoimmune disease

__ HIV

__ Deep vein thrombosis

__ Pulmonary embolism

__ Refuse to Answer

Q104. Have you used antibiotics in past 6 months?

1	Yes
0	No
8	Refuse to Answer

If Q104 is equal to 1 then skip to Q105

If not, then skip to Q106.

Q105. Please, specify what condition were you taking antibiotics for?

____

Q106. Have you been exposed to someone likely to have Coronavirus/COVID-19? (Check all that apply)

__ Yes, someone with positive test

__ Yes, someone with medical diagnosis, but no test

__ Yes, someone with possible symptoms, but no diagnosis by doctor

__ No

__ Refuse to Answer

If Q106D is equal to 1 and Q106A + Q106B + Q106C is greater than 0 then READ: "You can only choose the response "No" by itself. Please check responses." and skip to Q106.

COVID-19 KNOWLEDGE

Q107. Where do you get information about COVID19/the coronavirus? **(Interviewer: check all that apply and if none apply, move on to the next screen)** (Check all that apply)

__ Friends

__ Doctors/health professionals

__ Conservative TV/radio (e.g. Fox, One America Network, Drudge Report)

__ Liberal TV/radio (e.g. CNN, MSNBC, Huffington Post, NPR)

__ Social Media (e.g., Facebook, YouTube, Twitter)

__ Refuse to Answer

Q108. Which of these sources of COVID19 information is most important to you? (Choose one)

1 Friends

2 Doctors/health professionals

3 Conservative TV/radio (e.g. Fox, One America Network, Drudge Report)

4 Liberal TV/radio (e.g., CNN, MSNBC, Huffington Post, NPR)

5 Social Media (e.g., Facebook, YouTube, Twitter)

8 Refuse to Answer

READ: Im going to read a few statements about COVID-19. Please tell me if you think each statement is true, false or if you are unsure.

Q109. The pharmaceutical industry created the COVID-19 virus. (Choose one)

1 True

2 False

3 Unsure

8 Refuse to Answer

Q110. The coronavirus was created by the Chinese government as a biological weapon. (Choose one)

1 True
2 False
3 Unsure
8 Refuse to Answer

Q111. Some people in the US government are exaggerating the danger posed by COVID19. (Choose one)

1 True
2 False
3 Unsure
8 Refuse to Answer

Q112. Vaccines given to children for diseases like measles and mumps cause autism. (Choose one)

1 True
2 False
3 Unsure
8 Refuse to Answer

Q113. The virus that causes COVID19 can be easily spread from one person to another. (Choose one)

1 True
2 False
3 Unsure
8 Refuse to Answer

Q114. Many thousands of people have died from COVID19. (Choose one)

1 True
2 False
3 Unsure
8 Refuse to Answer

Q115. A vaccine is available to prevent COVID19. (Choose one)

1 True
2 False
3 Unsure
8 Refuse to Answer

Q116. Most people already have immunity to COVID19. (Choose one)

1 True
2 False
3 Unsure
8 Refuse to Answer

Q117. You can tell someone has COVID19 by looking at them. (Choose one)

1 True
2 False
3 Unsure
8 Refuse to Answer

Q118. There are effective treatments for COVID19 that can cure most people. (Choose one)

1 True
2 False
3 Unsure
8 Refuse to Answer

Q119. Having COVID19 is about as dangerous as having the flu. (Choose one)

1 True
2 False
3 Unsure
8 Refuse to Answer

Q120. COVID vaccines being offered to people like me are not as safe as other COVID vaccines. (Choose one)

1 True

2 False

3 Unsure

8 Refuse to Answer

Q121. COVID vaccines include a tracking device. (Choose one)

1 True

2 False

3 Unsure

8 Refuse to Answer

Q122. COVID vaccines are safe for pregnant women. (Choose one)

1 True

2 False

3 Unsure

8 Refuse to Answer

Q123. Some COVID vaccines could change my DNA. (Choose one)

1 True

2 False

3 Unsure

8 Refuse to Answer

COVID-19 TESTING AND VACCINATION

Q124. If you were offered a free test for COVID today that would give you results in 20 minutes from a nose swab, would you want to get it done? (Choose one)

0	No
1	Yes
2	Unsure
7	Don't Know
8	Refuse to Answer

If Q124 is equal to 1 and Q15 is equal to 1 then skip to Q125

If not, then skip to Q126.

Q125. Would you still agree to this test if the San Diego government required that we report your results by name?

1	Yes
0	No
7	Don't Know
8	Refuse to Answer
9	Not Applicable

Q126. Do you think you've had COVID-19? (Choose one)

1	Yes
2	No
3	Unsure
8	Refuse to Answer

Q127. On a scale of 1 to 10, how worried are you about getting COVID-19 (or getting it again)? (Note: 1 being not worried at all, and 10 being extremely worried.) **Interviewer, please ask the question even if participant says they had COVID already or had the vaccine.**

01	Low
02	
03	
04	
05	
06	
07	
08	
09	
10	High
98	Refuse to Answer

If Q126 is equal to 1 then skip to Q128

If not, then skip to Q129.

Q128. How long ago you think you had COVID-19?

__ __	MONTHS
__ __	WEEKS
__ __	DAYS
98	Refuse to Answer (Months)

Q129. Have you been tested for COVID-19 (outside of our study)? (Choose one)

1 Yes, I received a test

2 No, I have not been tested

3 I tried to get tested but couldn't

4 I am not sure if I got tested for COVID-19

8 Refuse to Answer

If Q129 is equal to 1 then skip to Q130

If not, then skip to Q158.

Q130. How many times were you tested for COVID-19, outside of our study?

__ __ times

98 Refuse to Answer

Q131. In what country/countries were you tested for COVID-19, outside of our study? **(Interviewer, check all that apply)** (Check all that apply)

__ US

__ Mexico

__ Other country

__ Refuse to Answer

Q132. When you were tested for COVID-19 outside of our study, what kind of test was it? **(Check all that apply)** (Check all that apply)

__ Nasal swab test

__ Blood (antibody) test

__ Saliva

__ Another type of COVID test

__ Refuse to Answer

If Q132D is equal to 1 then skip to Q133

If not, then skip to Q134.

Q133. Please specify what type of COVID-19 test was it.

____

Q134. Where did you get tested for COVID-19, outside of our study? **(Interviewer, read out list and check all that apply)** (Check all that apply)

__ Doctor's office

__ Community health clinic/Health fair

__ Needle Exchange

__ Research Study (other than our study)

__ Self-tested with a rapid home test kit

__ Other

__ Refuse to Answer

If Q134F is equal to 1 then skip to Q135

If not, then skip to Q136.

Q135. Specify where you got tested for COVID-19.

____

Q136. When was the LAST time you got tested for COVID-19 outside of our study?

__ __ / __ __ / __ __ __ __ mm / dd / yyyy

2097 Don't Know (Year)

2098 Refuse to Answer (Year)

Q137. Did you test positive for COVID-19? (Choose one)

1	Yes, I tested positive
2	No, I tested negative
3	I was tested, but did not get the result
4	Don't know/Didn't understand the result
8	Refuse to Answer

If Q137 is equal to 1 then skip to Q138

If not, then skip to instruction before Q139.

Q138. When did you test positive for COVID-19, outside of our study?

__ __ / __ __ / __ __ __ __	mm / dd / yyyy
2097	Don't Know (Year)
2098	Refuse to Answer (Year)

If Q126 is equal to 1 or Q137 is equal to 1 then skip to Q139

If not, then skip to Q158.

Q139. When you had or you think you had COVID, which of the following symptoms did you have? (Check all that apply) (Check all that apply)

__ Fever >100.4F (38C)

__ Subjective fever (felt feverish, unconfirmed)

__ Moderate fever or chills

__ Night sweats (wet sheets more than or equal to 3 times per week)

__ Repeated shaking with chills

__ Runny nose

__ Cough

__ Sore throat

__ Hoarseness

__ Shortness of breath

__ Nausea or vomiting

__ Headache

__ Abdominal pain

__ Diarrhea (three or more loose/looser than normal stools in a 24 hr period)

__ Sudden loss of smell

__ Sudden loss of taste

__ Joint pain

__ Fatigue/extreme tiredness

__ Refuse to Answer

If Q139A is equal to 1 then skip to Q140

If not, then skip to instruction before Q141.

Q140. For how long have you had Fever >100.4F (38C)? (Choose one)

1	Less than 8 days
2	8-13 days
3	14-20 days
4	More than 20 days
7	Don't Know
8	Refuse to Answer

If Q139B is equal to 1 then skip to Q141

If not, then skip to instruction before Q142.

Q141. For how long have you had Subjective fever? (Choose one)

1	Less than 8 days
2	8-13 days
3	14-20 days
4	More than 20 days
7	Don't Know
8	Refuse to Answer

If Q139C is equal to 1 then skip to Q142

If not, then skip to instruction before Q143.

Q142. For how long have you had Moderate fever or chills? (Choose one)

1	Less than 8 days
2	8-13 days
3	14-20 days
4	More than 20 days
7	Don't Know
8	Refuse to Answer

If Q139D is equal to 1 then skip to Q143

If not, then skip to instruction before Q144.

Q143. For how long have you had Night sweats? (Choose one)

1 Less than 8 days
2 8-13 days
3 14-20 days
4 More than 20 days
7 Don't Know
8 Refuse to Answer

If Q139E is equal to 1 then skip to Q144

If not, then skip to instruction before Q145.

Q144. For how long have you had Repeated shaking with chills? (Choose one)

1 Less than 8 days
2 8-13 days
3 14-20 days
4 More than 20 days
7 Don't Know
8 Refuse to Answer

If Q139F is equal to 1 then skip to Q145

If not, then skip to instruction before Q146.

Q145. For how long have you had Runny nose? (Choose one)

1 Less than 8 days
2 8-13 days
3 14-20 days
4 More than 20 days
7 Don't Know
8 Refuse to Answer

If Q139G is equal to 1 then skip to Q146

If not, then skip to instruction before Q147.

Q146. For how long have you had Cough? (Choose one)

1	Less than 8 days
2	8-13 days
3	14-20 days
4	More than 20 days
7	Don't Know
8	Refuse to Answer

If Q139H is equal to 1 then skip to Q147

If not, then skip to instruction before Q148.

Q147. For how long have you had Sore throat? (Choose one)

1	Less than 8 days
2	8-13 days
3	14-20 days
4	More than 20 days
7	Don't Know
8	Refuse to Answer

If Q139I is equal to 1 then skip to Q148

If not, then skip to instruction before Q149.

Q148. For how long have you had Hoarseness? (Choose one)

1	Less than 8 days
2	8-13 days
3	14-20 days
4	More than 20 days
7	Don't Know
8	Refuse to Answer

If Q139J is equal to 1 then skip to Q149

If not, then skip to instruction before Q150.

Q149. For how long have you had Shortness of breath? (Choose one)

1	Less than 8 days
2	8-13 days
3	14-20 days
4	More than 20 days
7	Don't Know
8	Refuse to Answer

If Q139K is equal to 1 then skip to Q150

If not, then skip to instruction before Q151.

Q150. For how long have you had Nausea or vomiting? (Choose one)

1	Less than 8 days
2	8-13 days
3	14-20 days
4	More than 20 days
7	Don't Know
8	Refuse to Answer

If Q139L is equal to 1 then skip to Q151

If not, then skip to instruction before Q152.

Q151. For how long have you had Headache? (Choose one)

1	Less than 8 days
2	8-13 days
3	14-20 days
4	More than 20 days
7	Don't Know
8	Refuse to Answer

If Q139M is equal to 1 then skip to Q152

If not, then skip to instruction before Q153.

Q152. For how long have you had Abdominal pain? (Choose one)

1	Less than 8 days
2	8-13 days
3	14-20 days
4	More than 20 days
7	Don't Know
8	Refuse to Answer

If Q139N is equal to 1 then skip to Q153

If not, then skip to instruction before Q154.

Q153. For how long have you had Diarrhea? (Choose one)

1	Less than 8 days
2	8-13 days
3	14-20 days
4	More than 20 days
7	Don't Know
8	Refuse to Answer

If Q139O is equal to 1 then skip to Q154

If not, then skip to instruction before Q155.

Q154. For how long have you had Sudden loss of smell? (Choose one)

1	Less than 8 days
2	8-13 days
3	14-20 days
4	More than 20 days
7	Don't Know
8	Refuse to Answer

If Q139P is equal to 1 then skip to Q155

If not, then skip to instruction before Q156.

Q155. For how long have you had Sudden loss of taste? (Choose one)

1	Less than 8 days
2	8-13 days
3	14-20 days
4	More than 20 days
7	Don't Know
8	Refuse to Answer

If Q139Q is equal to 1 then skip to Q156

If not, then skip to instruction before Q157.

Q156. For how long have you had Joint pain? (Choose one)

1	Less than 8 days
2	8-13 days
3	14-20 days
4	More than 20 days
7	Don't Know
8	Refuse to Answer

If Q139R is equal to 1 then skip to Q157

If not, then skip to Q158.

Q157. For how long have you had Fatigue/extreme tiredness? (Choose one)

1	Less than 8 days
2	8-13 days
3	14-20 days
4	More than 20 days
7	Don't Know
8	Refuse to Answer

Q158. Have you been hospitalized due to COVID-19? (Choose one)

0 No

1 Yes, I have been hospitalized

8 Refuse to Answer

If Q158 is equal to 1 then skip to Q159

If not, then skip to Q160.

Q159. When were you last hospitalized for COVID-19? __ __ / __ __ / __ __ __ __ mm / dd / yyyy

2097 Don't Know (Year)

Q160. How many people do you know who have died from COVID-19? __ __ __ people

998 Refuse to Answer

Q161. How many people do you know who have had a COVID19 vaccine?

__ __ __ __ people

9998 Refuse to Answer

Q162. Have you ever received a flu vaccination?

1 Yes

0 No

7 Don't Know

8 Refuse to Answer

Q163. Have you been offered a COVID-19 vaccine? (Choose one)

1 Yes

2 No

3 Unsure

8 Refuse to Answer

Q164. Have you received a COVID-19 vaccine? (Choose one)

1 Yes

2 No

3 Unsure

8 Refuse to Answer

If Q164 is equal to 1 then skip to Q165

If not, then skip to instruction before Q172.

Q165. In what country have you received the COVID-19 vaccine? (Choose one)

1	US
2	Mexico
3	Other
8	Refuse to Answer

If Q165 is equal to 3 then skip to Q166

If not, then skip to Q167.

Q166. Specify the country where you received the COVID-19 vaccine.

____

Q167. What type of COVID-19 vaccine did you get? (Choose one)

01	Moderna
02	Pfizer
03	Johnson and Johnson
04	Astrazeneca
05	CanSino
06	Sinovac
07	Sputnik
08	Other
97	Don't Know
98	Refuse to Answer

If Q167 is equal to 8 then skip to Q168

If not, then skip to Q169.

Q168. Specify the COVID-19 vaccine.

____

Q169. How many shots of the COVID-19 vaccine did you get? (Choose one)

1	One
2	Two
3	I forgot
8	Refuse to Answer

Q170. When did you received the first shot of the COVID-19 vaccine?

__ __ / __ __ / __ __ __ __	mm / dd / yyyy
2097	Don't Know (Year)
2098	Refuse to Answer (Year)

If Q169 is equal to 2 then skip to Q171

If not, then skip to instruction before Q172.

Q171. When did you received the second shot of the COVID-19 vaccine?

__ __ / __ __ / __ __ __ __	mm / dd / yyyy
2097	Don't Know (Year)
2098	Refuse to Answer (Year)

If Q164 is not equal to 1 then skip to Q172

If not, then skip to Q186.

Q172. Have you tried to get the COVID vaccine but could not get it?

1	Yes
0	No
8	Refuse to Answer

If Q172 is equal to 1 then skip to Q173

If not, then skip to instruction before Q174.

Q173. What problems have you had trying to get the COVID vaccine? **(Interviewer, read out list and check all that apply)** (Check all that apply)

__ I dont know where to go

__ Too far away/transportation issues

__ I didnt have an appointment

__ I dont have access to a computer or phone for making an appointment

__ I dont like the place or the people at the places that are offering vaccine

__ Line was too long

__ The staff mistreated me/treated me badly

__ I didnt meet the criteria (e.g., age, pre-existing condition)

__ Dont have insurance/money

__ Didnt have identification/ID

__ Only CanSino, Sinovac, or Sputnik were offered

__ Other

__ Refuse to Answer

If Q173L is equal to 1 then skip to Q174

If not, then skip to Q175.

Q174. Specify what problems have you had trying to get the COVID vaccine?

____

Q175. If you were offered a free vaccine available to prevent you from getting COVID 19 or getting it again, would you agree to get it? (Choose one)

1 Yes

2 No

3 Unsure

8 Refuse to Answer

If Q175 is equal to 2 or Q175 is equal to 3 then skip to Q176

If not, then skip to Q186.

Q176. Why would you not want to get a vaccine to prevent you from getting COVID (or from getting it again)? (Check all that apply)

__ Don't like shots/needles

__ I'm allergic to vaccines

__ I'm not concerned about getting really sick from COVID19

__ I don't think vaccines work really well

__ I don't trust that the vaccine will be safe

__ I don't believe that the COVID19 pandemic is as bad as some people say it is

__ I don't want to pay for it

__ I don't know enough about how a COVID19 vaccine works

__ Worried I would get COVID19 from the vaccine

__ I don't care about my health

__ I'm going to get COVID anyway/fate

__ Worried that I would test positive for COVID from the vaccine

__ Takes too much time/too far

__ I'm concerned about side effects from the vaccine/will make me sick

__ I already had COVID and I don't think I can get it again

__ I have a strong immune system/think I am immune

__ I am taking vitamins and natural remedies to protect me from getting COVID

__ I am afraid of getting it because I have pre-existing health conditions

__ Other

__ Refuse to Answer

If Q176S is equal to 1 then skip to Q177

If not, then skip to Q178.

Q177. Please specify why would you not want to get a vaccine to prevent you from getting COVID.

____

Q178. Which of the following places would you be willing to go for a free COVID-19 vaccine? **(check all that apply)** (Check all that apply)

__ Doctor's office

__ Community health clinic

__ Pharmacy

__ Jail/Prison (if you were incarcerated)

__ Needle exchange van

__ Other mobile van

__ Shelter

__ Soup kitchen

__ Drop-in center

__ Church

__ Health fair

__ Other

__ I would not be willing to go to any place

__ Refuse to Answer

If Q178M is equal to 1 and Q178 is greater than 1 then READ: "The "I would not be willing to go to any place" option may not be chosen in conjunction with other options. Please review and revise the answer." and skip to Q178.

If Q178L is equal to 1 then skip to Q179

If not, then skip to Q180.

Q179. Please specify which place/places would you be willing to go to for a free COVID-19 vaccine?

— —

Q180. How much time would you be willing to take out of your day to get a free COVID-19 vaccine? (Choose one)

0 No amount of time (zero minutes)

1 Less than 1 hr

2 1-3 hours

3 Any amount of time

8 Refuse to Answer

Q181. Which of the following might make you more willing to get a free COVID vaccine? (Check all that apply)

__ If it was only one shot instead of 2

__ If I could inhale/swallow the vaccine

__ If I could get it close by where I live/hang out

__ If I could get it at a place I already go

__ If my friends already got it

__ If someone I trusted told me it was safe

__ Other

__ Nothing could convince me

__ Refuse to Answer

If Q181H is equal to 1 and Q181 is greater than 1 then READ: "The "Nothing could convince me" option may not be chosen in conjunction with other options. Please review and revise the answer." and skip to Q181.

Q182. Would you be more or less willing to get a COVID vaccine if you received $10? (Choose one)

1 More likely
2 Less likely
3 No difference
8 Refuse to Answer

Q183. Would you be more or less willing to get a COVID vaccine if you received $25? (Choose one)

1 More likely
2 Less likely
3 No difference
8 Refuse to Answer

Q184. Would you be more or less willing to get a COVID vaccine if you received $50? (Choose one)

1 More likely
2 Less likely
3 No difference
8 Refuse to Answer

Q185. Would you be more or less willing to get a COVID vaccine if you received $100? (Choose one)

1 More likely
2 Less likely
3 No difference
8 Refuse to Answer

Q186. If you were to test positive for COVID-19, would you be able to isolate?

1 Yes
0 No
7 Don't Know
8 Refuse to Answer

Q187. If you were to be exposed to someone with COVID-19, would you be able to quarantine (i.e., separate yourself from other people for 10 days)?

1	Yes
0	No
7	Don't Know
8	Refuse to Answer

READ: To what extent do you agree with the following two statements:

Q188. I know where I can get COVID-19 testing in my community. (Choose one)

0	Strongly disagree
1	Disagree
2	Neither disagree or agree
3	Agree
4	Stronlgy agree
8	Refuse to Answer

Q189. It is easy to get tested for COVID-19. (Choose one)

0	Strongly disagree
1	Disagree
2	Neither disagree or agree
3	Agree
4	Stronlgy agree
8	Refuse to Answer

Q190. If I get a negative test result, it means...**(Interviewer read the options to the respondent and check all that apply)** (Check all that apply)

__ I don't have to worry about getting COVID-19

__ I don't have COVID-19 now

__ I can be around others without giving the virus to them

__ I can be around others without getting virus from them

__ Refuse to Answer

SARSCoV-2 Component (Health Questions)

READ: READ TO THE RESPONDENT: Now I would like to ask you some questions about your personal health. Remember that all the information you give me is private and will not be shared outside of the study. We will start with a series of signs and symptoms, I will ask you if you currently have it, if so, for how long you've had it.

CURRENT SYMPTOMS ASSESSMENT

Q191. Do you have: **Fever >100.4F (38C)**

1	Yes
0	No
7	Don't Know
8	Refuse to Answer

If Q191 is equal to 1 then skip to Q192

If not, then skip to Q193.

Q192. For how long have you had it? (Choose one)

1	Less than 8 days
2	8-13 days
3	14-20 days
4	More than 20 days
7	Don't Know
8	Refuse to Answer

Q193. Do you have**: Subjective fever (felt feverish, unconfirmed)?**

1	Yes
0	No
7	Don't Know
8	Refuse to Answer

If Q193 is equal to 1 then skip to Q194

If not, then skip to Q195.

Q194. For how long have you had it? (Choose one)

1	Less than 8 days
2	8-13 days
3	14-20 days
4	More than 20 days
7	Don't Know
8	Refuse to Answer

Q195. Do you have: **Moderate fever or chills?**

1	Yes
0	No
7	Don't Know
8	Refuse to Answer

If Q195 is equal to 1 then skip to Q196

If not, then skip to Q197.

Q196. For how long have you had it? (Choose one)

1	Less than 8 days
2	8-13 days
3	14-20 days
4	More than 20 days
7	Don't Know
8	Refuse to Answer

Q197. Do you have**: Night sweats (wet sheets more than or equal to 3 times per week)?**

1	Yes
0	No
7	Don't Know
8	Refuse to Answer

If Q197 is equal to 1 then skip to Q198

If not, then skip to Q199.

Q198. For how long have you had it? (Choose one)	1	Less than 8 days
	2	8-13 days
	3	14-20 days
	4	More than 20 days
	7	Don't Know
	8	Refuse to Answer
Q199. Do you have**: Repeated shaking with chills?**	1	Yes
	0	No
	7	Don't Know
	8	Refuse to Answer

If Q199 is equal to 1 then skip to Q200

If not, then skip to Q201.

Q200. For how long have you had it? (Choose one)	1	Less than 8 days
	2	8-13 days
	3	14-20 days
	4	More than 20 days
	7	Don't Know
	8	Refuse to Answer
Q201. Do you have**: Runny nose**	1	Yes
	0	No
	7	Don't Know
	8	Refuse to Answer

If Q201 is equal to 1 then skip to Q202

If not, then skip to Q203.

Q202. For how long have you had it? (Choose one)

1 Less than 8 days

2 8-13 days

3 14-20 days

4 More than 20 days

7 Don't Know

8 Refuse to Answer

Q203. Do you have**: Cough?**

1 Yes

0 No

7 Don't Know

8 Refuse to Answer

If Q203 is equal to 1 then skip to Q204

If not, then skip to Q205.

Q204. For how long have you had it? (Choose one)

1 Less than 8 days

2 8-13 days

3 14-20 days

4 More than 20 days

7 Don't Know

8 Refuse to Answer

Q205. Do you have**: Sore throat?**

1 Yes

0 No

7 Don't Know

8 Refuse to Answer

If Q205 is equal to 1 then skip to Q206

If not, then skip to Q207.

Q206. For how long have you had it? (Choose one)

1 Less than 8 days

2 8-13 days

3 14-20 days

4 More than 20 days

7 Don't Know

8 Refuse to Answer

Q207. Do you have**: Hoarseness?**

1 Yes

0 No

7 Don't Know

8 Refuse to Answer

If Q207 is equal to 1 then skip to Q208

If not, then skip to Q209.

Q208. For how long have you had it? (Choose one)

1 Less than 8 days

2 8-13 days

3 14-20 days

4 More than 20 days

7 Don't Know

8 Refuse to Answer

Q209. Do you have**: Shortness of breath**

1	Yes
0	No
7	Don't Know
8	Refuse to Answer

If Q209 is equal to 1 then skip to Q210

If not, then skip to Q211.

Q210. For how long have you had it? (Choose one)

1	Less than 8 days
2	8-13 days
3	14-20 days
4	More than 20 days
7	Don't Know
8	Refuse to Answer

Q211. Do you have**: enlarged lymph nodes**

1	Yes
0	No
7	Don't Know
8	Refuse to Answer

If Q211 is equal to 1 then skip to Q212

If not, then skip to Q213.

Q212. For how long have you had it? (Choose one)

1	Less than 8 days
2	8-13 days
3	14-20 days
4	More than 20 days
7	Don't Know
8	Refuse to Answer

Q213. Do you have: **Nausea or vomiting?**

1	Yes
0	No
7	Don't Know
8	Refuse to Answer

If Q213 is equal to 1 then skip to Q214

If not, then skip to Q215.

Q214. For how long have you had it? (Choose one)

1	Less than 8 days
2	8-13 days
3	14-20 days
4	More than 20 days
7	Don't Know
8	Refuse to Answer

Q215. Do you have: **Headache**

1	Yes
0	No
7	Don't Know
8	Refuse to Answer

If Q215 is equal to 1 then skip to Q216

If not, then skip to Q217.

Q216. For how long have you had it? (Choose one)

1 Less than 8 days
2 8-13 days
3 14-20 days
4 More than 20 days
7 Don't Know
8 Refuse to Answer

Q217. Do you have: **Abdominal pain**

1 Yes
0 No
7 Don't Know
8 Refuse to Answer

If Q217 is equal to 1 then skip to Q218

If not, then skip to Q219.

Q218. For how long have you had it? (Choose one)

1 Less than 8 days
2 8-13 days
3 14-20 days
4 More than 20 days
7 Don't Know
8 Refuse to Answer

Q219. Do you have: **Diarrhea (three or more loose/looser than normal stools in a 24 hr period)**

1 Yes
0 No
7 Don't Know
8 Refuse to Answer

If Q219 is equal to 1 then skip to Q220

If not, then skip to Q221.

Q220. For how long have you had it? (Choose one)	1	Less than 8 days
	2	8-13 days
	3	14-20 days
	4	More than 20 days
	7	Don't Know
	8	Refuse to Answer
Q221. Do you have: **Sudden loss of smell**	1	Yes
	0	No
	7	Don't Know
	8	Refuse to Answer

If Q221 is equal to 1 then skip to Q222

If not, then skip to Q223.

Q222. For how long have you had it? (Choose one)	1	Less than 8 days
	2	8-13 days
	3	14-20 days
	4	More than 20 days
	7	Don't Know
	8	Refuse to Answer
Q223. Do you have: **Sudden loss of taste**	1	Yes
	0	No
	7	Don't Know
	8	Refuse to Answer

If Q223 is equal to 1 then skip to Q224

If not, then skip to Q225.

Q224. For how long have you had it? (Choose one)

1	Less than 8 days
2	8-13 days
3	14-20 days
4	More than 20 days
7	Don't Know
8	Refuse to Answer

Q225. Do you have: **Joint pain**

1	Yes
0	No
7	Don't Know
8	Refuse to Answer

If Q225 is equal to 1 then skip to Q226

If not, then skip to Q227.

Q226. For how long have you had it? (Choose one)

1	Less than 8 days
2	8-13 days
3	14-20 days
4	More than 20 days
7	Don't Know
8	Refuse to Answer

Q227. Do you have: **fatigue/extreme tiredness**

1	Yes
0	No
7	Don't Know
8	Refuse to Answer

If Q227 is equal to 1 then skip to Q228

If not, then skip to Q229.

Q228. For how long have you had it? (Choose one)

1	Less than 8 days
2	8-13 days
3	14-20 days
4	More than 20 days
7	Don't Know
8	Refuse to Answer

Q229. Do you have: **hemoptysis (coughed up blood in phlegm)**

1	Yes
0	No
7	Don't Know
8	Refuse to Answer

If Q229 is equal to 1 then skip to Q230

If not, then skip to Q231.

Q230. For how long have you had it? (Choose one)

1	Less than 8 days
2	8-13 days
3	14-20 days
4	More than 20 days
7	Don't Know
8	Refuse to Answer

Q231. Do you have: **chest pain or tightness in chest**

1	Yes
0	No
7	Don't Know
8	Refuse to Answer

If Q231 is equal to 1 then skip to Q232

If not, then skip to Q233.

Q232. For how long have you had it? (Choose one)

1	Less than 8 days
2	8-13 days
3	14-20 days
4	More than 20 days
7	Don't Know
8	Refuse to Answer

Q233. Do you have: **lost weight unintentionally**

1	Yes
0	No
7	Don't Know
8	Refuse to Answer

If Q233 is equal to 1 then skip to Q234

If not, then skip to instruction before Q235.

Q234. For how long have you had it? (Choose one)

1	Less than 8 days
2	8-13 days
3	14-20 days
4	More than 20 days
7	Don't Know
8	Refuse to Answer

COVID Intersectional Stigma Questions

READ: READ TO THE RESPONDENT: The following questions are going to ask about whether you were treated differently during the COVID-19 pandemic, when the government asked people to shelter in place, stay at home, and to limit the number of people hanging out in public.
By COVID-19, we mean the novel corona virus which is also called corona, the Rona, and Miss Rona.
The first few questions will ask how worried you were that you might be treated differently, and the last few questions will ask about different ways you were actually treated differently.

Q235. When the COVID-19 pandemic began in March 2020 were you worried that you might be verbally or physically harassed by others (including the police) because there were fewer people were in public?

1	Yes
0	No
8	Refuse to Answer

If Q235 is equal to 1 then skip to instruction before Q236

If not, then skip to Q242.

READ: READ TO THE RESPONDENT: On a scale from 1-5, with 1 being not at all and 5 being a lot, how much was this worry that you would be threatened or harassed due to the following:

Q236. Worried that you would be threatened or harassed due to: **Your gender** (Choose one)

1	Not at all
2	A little bit
3	Somewhat
4	Quiet a bit
5	A lot
8	Refuse to Answer

Q237. Worried that you would be threatened or harassed due to: **The color of your skin** (Choose one)

1	Not at all
2	A little bit
3	Somewhat
4	Quiet a bit
5	A lot
8	Refuse to Answer

Q238. Worried that you would be threatened or harassed due to: **Visible tattoos or other physical markings** (Choose one)

1	Not at all
2	A little bit
3	Somewhat
4	Quiet a bit
5	A lot
8	Refuse to Answer

Q239. Worried that you would be threatened or harassed due to: **The clothes you wear** (Choose one)

1	Not at all
2	A little bit
3	Somewhat
4	Quiet a bit
5	A lot
8	Refuse to Answer

Q240. Worried that you would be threatened or harassed due to: **Having track marks or other signs of active drug use** (Choose one)

1	Not at all
2	A little bit
3	Somewhat
4	Quiet a bit
5	A lot
8	Refuse to Answer

Q241. Worried that you would be threatened or harassed due to: **Your sexual orientation** (Choose one)

1	Not at all
2	A little bit
3	Somewhat
4	Quiet a bit
5	A lot
8	Refuse to Answer

Q242. When the COVID-19 pandemic began in March 2020 were you worried that you might be verbally or physically harassed by others (including the police) if you wore a mask?

1	Yes
0	No
8	Refuse to Answer

If Q242 is equal to 1 then skip to instruction before Q243

If not, then skip to Q249.

READ: READ TO THE RESPONDENT: On a scale from 1-5, with 1 being not at all and 5 being a lot, how much was this worry that you would be threatened or harassed for wearing a mask due to the following:

Q243. Worried that you would be threatened or harassed for **wearing a mask** due to: **Your gender** (Choose one)

1 Not at all

2 A little bit

3 Somewhat

4 Quiet a bit

5 A lot

8 Refuse to Answer

Q244. Worried that you would be threatened or harassed for **wearing a mask** due to: **The color of your skin** (Choose one)

1 Not at all

2 A little bit

3 Somewhat

4 Quiet a bit

5 A lot

8 Refuse to Answer

Q245. Worried that you would be threatened or harassed for **wearing a mask** due to: **Visible tattoos or other physical markings** (Choose one)

1 Not at all

2 A little bit

3 Somewhat

4 Quiet a bit

5 A lot

8 Refuse to Answer

Q246. Worried that you would be threatened or harassed for **wearing a mask** due to: **The clothes you wear** (Choose one)

1 Not at all

2 A little bit

3 Somewhat

4 Quiet a bit

5 A lot

8 Refuse to Answer

Q247. Worried that you would be threatened or harassed for **wearing a mask** due to: **Having track marks or other signs of active drug use** (Choose one)

1 Not at all

2 A little bit

3 Somewhat

4 Quiet a bit

5 A lot

8 Refuse to Answer

Q248. Worried that you would be threatened or harassed for **wearing a mask** due to: **Your sexual orientation** (Choose one)

1 Not at all

2 A little bit

3 Somewhat

4 Quiet a bit

5 A lot

8 Refuse to Answer

Q249. During the COVID-19 pandemic did you experience more verbal harassment than before (called names or insulted)?

1 Yes

0 No

8 Refuse to Answer

If Q249 is equal to 1 then skip to instruction before Q250

If not, then skip to Q256.

READ: READ TO THE RESPONDENT: On a scale from 1-5, with 1 being not at all and 5 being a lot, how much was this treatment due to the following:

Q250. Experienced more verbal harassment than before due to: **Your gender** (Choose one)

1 Not at all

2 A little bit

3 Somewhat

4 Quiet a bit

5 A lot

8 Refuse to Answer

Q251. Experienced more verbal harassment than before due to: **The color of your skin** (Choose one)

1 Not at all

2 A little bit

3 Somewhat

4 Quiet a bit

5 A lot

8 Refuse to Answer

Q252. Experienced more <u>verbal harassment</u> than before due to: **Visible tattoos or other physical markings** (Choose one)

1	Not at all
2	A little bit
3	Somewhat
4	Quiet a bit
5	A lot
8	Refuse to Answer

Q253. Experienced more <u>verbal harassment</u> than before due to: **The clothes you wear** (Choose one)

1	Not at all
2	A little bit
3	Somewhat
4	Quiet a bit
5	A lot
8	Refuse to Answer

Q254. Experienced more <u>verbal harassment</u> than before due to: **Having track marks or other signs of active drug use** (Choose one)

1	Not at all
2	A little bit
3	Somewhat
4	Quiet a bit
5	A lot
8	Refuse to Answer

Q255. Experienced more verbal harassment than before due to: **Your sexual orientation** (Choose one)

1	Not at all
2	A little bit
3	Somewhat
4	Quiet a bit
5	A lot
8	Refuse to Answer

Q256. During the COVID-19 pandemic were you physically threatened or harmed more than before?

1	Yes
0	No
8	Refuse to Answer

If Q256 is equal to 1 then skip to instruction before Q257

If not, then skip to instruction before Q263.

READ: READ TO THE RESPONDENT: On a scale from 1-5, with 1 being not at all and 5 being a lot, how much was this due to the following:

Q257. Physically threatened or harmed more than before due to: **Your gender** (Choose one)

1	Not at all
2	A little bit
3	Somewhat
4	Quiet a bit
5	A lot
8	Refuse to Answer

Q258. Physically threatened or harmed more than before due to: **The color of your skin** (Choose one)

1 Not at all

2 A little bit

3 Somewhat

4 Quiet a bit

5 A lot

8 Refuse to Answer

Q259. Physically threatened or harmed more than before due to: **Visible tattoos or other physical markings** (Choose one)

1 Not at all

2 A little bit

3 Somewhat

4 Quiet a bit

5 A lot

8 Refuse to Answer

Q260. Physically threatened or harmed more than before due to: **The clothes you wear** (Choose one)

1 Not at all

2 A little bit

3 Somewhat

4 Quiet a bit

5 A lot

8 Refuse to Answer

Q261. Physically threatened or harmed more than before due to: **Having track marks or other signs of active drug use** (Choose one)

1 Not at all

2 A little bit

3 Somewhat

4 Quiet a bit

5 A lot

8 Refuse to Answer

Q262. Physically threatened or harmed more than before due to: **Your sexual orientation** (Choose one)

1 Not at all

2 A little bit

3 Somewhat

4 Quiet a bit

5 A lot

8 Refuse to Answer

GAD-7 ANXIETY SCALE ITEMS

READ: READ TO THE RESPONDENT: The next few questions are about your mood.

GAD-7 ANXIETY SCALE ITEMS (COVBO1-COVBO7)

Q263. Over the last 2 weeks, how often have you been bothered by the following problems: **Feeling nervous, anxious, or on edge?** (Choose one)

1	Not at all
2	Several days
3	Over half the days
4	Nearly every day
7	Don't Know
8	Refuse to Answer

Q264. Over the last 2 weeks, how often have you been bothered by: **Not being able to stop or control worrying?** (Choose one)

1	Not at all
2	Several days
3	Over half the days
4	Nearly every day
7	Don't Know
8	Refuse to Answer

Q265. Over the last 2 weeks, how often have you been bothered by: **Worrying too much about different things?** (Choose one)

1	Not at all
2	Several days
3	Over half the days
4	Nearly every day
7	Don't Know
8	Refuse to Answer

Q266. Over the last 2 weeks, how often have you been bothered by: **Trouble relaxing?** (Choose one)

1	Not at all
2	Several days
3	Over half the days
4	Nearly every day
7	Don't Know
8	Refuse to Answer

Q267. Over the last 2 weeks, how often have you been bothered by: **Being so restless that it's hard to sit still?** (Choose one)

1	Not at all
2	Several days
3	Over half the days
4	Nearly every day
7	Don't Know
8	Refuse to Answer

Q268. Over the last 2 weeks, how often have you been bothered by: **Being easily annoyed or irritable?** (Choose one)

1 Not at all

2 Several days

3 Over half the days

4 Nearly every day

7 Don't Know

8 Refuse to Answer

Q269. Over the last 2 weeks, how often have you been bothered by: **Feeling afraid as if something awful might happen?** (Choose one)

1 Not at all

2 Several days

3 Over half the days

4 Nearly every day

7 Don't Know

8 Refuse to Answer

READ: READ TO THE RESPONDENT: Please indicate the extent to which you agree with each of the following statements:

BRIEF RESILIENCE SCALE ITEMS (COVCOP1-COVCOP6)

Q270. I tend to bounce back quickly after hard times. (Choose one)

1 Strongly Disagree

2 Disagree

3 Neutral

4 Agree

5 Strongly Agree

8 Refuse to Answer

Q271. I have a hard time making it through stressful events. (Choose one)

1 Strongly Disagree
2 Disagree
3 Neutral
4 Agree
5 Strongly Agree
8 Refuse to Answer

Q272. It does not take me long to recover from a stressful event. (Choose one)

1 Strongly Disagree
2 Disagree
3 Neutral
4 Agree
5 Strongly Agree
8 Refuse to Answer

Q273. It is hard for me to snap back when something bad happens. (Choose one)

1 Strongly Disagree
2 Disagree
3 Neutral
4 Agree
5 Strongly Agree
8 Refuse to Answer

Q274. I usually come through difficult times with little trouble. (Choose one)

1 Strongly Disagree

2 Disagree

3 Neutral

4 Agree

5 Strongly Agree

8 Refuse to Answer

Q275. I tend to take a long time to get over set-backs in my life. (Choose one)

1 Strongly Disagree

2 Disagree

3 Neutral

4 Agree

5 Strongly Agree

8 Refuse to Answer

Q276. When was your most recent visit to a doctor, nurse, or other health care provider? Includes telemedicine and remote visits. (Choose one)

1 Within the last two weeks

2 Between 2 weeks and a month

3 Over a month but within 6 months

4 6-12 months ago

5 More than 12 months ago

8 Refuse to Answer

Q277. In the last 7 days, have you missed taking any prescribed medications? (Choose one)

1 Yes,

2 Yes,

3 Yes,

4 No

8 Refu

Tuberculosis, Perceptions, Testing, and Treatment

READ: READ TO THE RESPONDENT: The following questions are about your understanding of and feelings about TB. Please answer "Yes" or "No" to each question. If you don't know the answer, that is alright; just tell me you don't know.

Q278. Can people have TB in their body that is sleeping and not an active form of disease?

1 Yes

0 No

7 Don't Know

8 Refuse to Answer

Q279. Does sharing dishes, bottles, or a toothbrush with someone who has TB increase a person's chance of getting TB?

1 Yes

0 No

7 Don't Know

8 Refuse to Answer

Q280. Can TB be spread from person to person through the air?

1 Yes

0 No

7 Don't Know

8 Refuse to Answer

Q281. Can TB be cured by taking medicines?

1 Yes

0 No

7 Don't Know

8 Refuse to Answer

Q282. Do you have a BCG scar? (by BCG scar I mean that BCG is administered in the bicep, unlike TB skin test (PPD) that is administered in the forearm)

1 Yes

0 No

7 Don't Know

8 Refuse to Answer

Q283. Have you ever had a skin test for TB?

1 Yes

0 No

7 Don't Know

8 Refuse to Answer

If Q283 is equal to 1 then skip to Q284

If not, then skip to Q285.

Q284. What was the result of your last test? (Choose one)

0 Negative

1 Positive

2 Indeterminate

7 Don't Know

8 Refuse to Answer

Q285. Have you ever had a chest X-ray because a medical person thought you might have TB?

1 Yes

0 No

7 Don't Know

8 Refuse to Answer

If Q285 is equal to 1 then skip to Q286

If not, then skip to Q287.

Q286. When was the last time you had a chest X-ray for TB? (Choose one)

1 Within the past month

2 2-6 Months ago

3 7-12 Months ago

4 More than a year ago

5 Don't Know

6 Refuse to Answer

Q287. Have you ever been told by a doctor or health care provider that you have active TB? This does not include having a positive skin test for TB but no symptoms.

1 Yes

0 No

7 Don't Know

8 Refuse to Answer

If Q287 is equal to 1 then skip to Q288

If not, then skip to Q293.

Q288. In which CITY were you told that you have active TB?

____

Q289. In which STATE were you told that you have active TB?

____

Q290. In which COUNTRY were you told that you have active TB? (Choose one)

0 Mexico

1 US

2 Other

7 Don't Know

8 Refuse to Answer

If Q290 is not equal to 2, then skip to Q292.

Q291. Specify other country:

____

Q292. When were you told that you had an active TB test?

__ __ / __ __ __ __	mm / yyyy
2097	Don't Know (Year)
2098	Refuse to Answer (Year)

Q293. Have you ever taken medication for TB disease or sleeping TB?

1	Yes
0	No
7	Don't Know
8	Refuse to Answer

If Q293 is equal to 1 then skip to Q294

If not, then skip to Q298.

Q294. For how many months did you take TB medicines?

__ __	months
97	Don't Know
98	Refuse to Answer

Q295. Have you stopped taking your medicine sooner than your doctor asked you to?

1	Yes
0	No
7	Don't Know
8	Refuse to Answer

If Q295 is equal to 1 then skip to Q296

If not, then skip to instruction before Q297.

Q296. What was your reason(s) for stopping early? **(Interviewer: Read out the listed reasons and check all that apply)** (Check all that apply)

__	The drugs made me feel sick
__	The drugs made me feel sick
__	I could not afford to buy the medicine
__	My doctor told me to stop
__	I was deported back to Mexico
__	Other
__	Refuse to Answer

If Q296F is equal to 1 then skip to Q297

If not, then skip to Q298.

Q297. Specify other reason for stopping early:

____

Q298. Have you ever known anyone who had TB?

1	Yes
0	No
7	Don't Know
8	Refuse to Answer

If Q298 is equal to 1 then skip to Q299

If not, then skip to instruction before Q301.

Q299. How many people have you known who had TB?

__ __ __ __	people
9997	Don't Know
9998	Refuse to Answer

Q300. What was your relationship to that person or persons? (**Interviewer: Read out the listed options and check all that apply)** (Check all that apply)

__ Adult family member living with you including spouse

__ Child family member living with you

__ Non-family member living with you

__ Family member NOT living with you

__ Sex partner not living with you

__ Close friend

__ Someone you work with

__ Acquaintance (someone you met with once or twice)

__ Other drug user

__ Other

__ Don't Know

__ Refuse to Answer

If Q300J is equal to 1 then skip to Q301

If not, then skip to Q302.

Q301. Specify relationship to that person:

____

Q302. In the last **6 months**, did you drink raw milk or unpasteurized cheese? This is usually with milk products that haven't been processed in a factory or store like **queso fresco.**

1 Yes

0 No

7 Don't Know

8 Refuse to Answer

Q303. Have you **ever** lived or worked on a dairy, ranch, slaughterhouse, or other place where cows are raised or butchered?

1	Yes
0	No
7	Don't Know
8	Refuse to Answer

If Q191 is equal to 1 or Q193 is equal to 1 or Q195 is equal to 1 or Q197 is equal to 1 or Q199 is equal to 1 or Q201 is equal to 1 or Q203 is equal to 1 or Q205 is equal to 1 or Q207 is equal to 1 or Q209 is equal to 1 or Q213 is equal to 1 or Q215 is equal to 1 or Q217 is equal to 1 or Q219 is equal to 1 or Q221 is equal to 1 or Q223 is equal to 1 or Q227 is equal to 1 then READ: "Inteviewer, at the end of the interview, you will be prompted to provide the participant with a Covid-19 referral.".

If Q204 is equal to 4 and (Q195 is equal to 1 or Q197 is equal to 1 or Q207 is equal to 1 or Q209 is equal to 1 or Q211 is equal to 1 or Q225 is equal to 1 or Q227 is equal to 1 or Q231 is equal to 1) or Q229 is equal to 1 then READ: "Inteviewer, at the end of the interview, you will be prompted to provide the participant with a TB referral.".

HIV Self-Tests Section

READ: READ TO THE RESPONDENT: The next set of questions asks about HIV self-tests. An HIV self-test refers to a rapid HIV test that you can give yourself at home or any convenient location by collecting your own saliva or blood via a finger prick with a small needle. If you test HIV-positive on an HIV self-test, you will still need to take a standard HIV test at a clinic or another community-based organization that performs HIV testing to confirm your test result. HIV self-tests are currently available in other countries, including the United States. To plan for the future use of HIV self-tests in Mexico, we would like to know what you think about HIV self-tests.

Q304. Have you ever used an HIV self-test to test yourself for HIV infection? (Choose one)

0 No

1 Yes

2 Not Applicable (already HIV positive)

8 Refuse to Answer

If Q304 is equal to 2, then skip to instruction before Q314.

Q305. Would you be willing to use an HIV self-test to test yourself for HIV infection? (Choose one)

1 Definitely not

2 Probably not

3 Not sure, do not know

4 Probably

5 Definitely

8 Refuse to Answer

Q306. Would you be willing to give your <u>sexual partners</u> HIV self-tests to test themselves for HIV infection? (Choose one)

1	Definitely not
2	Probably not
3	Not sure, do not know
4	Probably
5	Definitely
8	Refuse to Answer

Q307. Would you be willing to give your <u>drug use partners</u> HIV self-tests to test themselves for HIV infection? (Choose one)

1	Definitely not
2	Probably not
3	Not sure, do not know
4	Probably
5	Definitely
8	Refuse to Answer

If Q305 is equal to 1 or Q305 is equal to 2 or Q305 is equal to 3 then skip to instruction before Q308

If not, then skip to instruction before Q311.

READ: READ TO THE RESPONDENT: Now I am going to read to you a list of reasons some people do <u>not</u> want to use HIV self-tests. Please indicate the extent to which you agree or disagree with each statement.

Q308. I would <u>not</u> want to use an HIV self-test because **I would be worried that HIV self-tests are less accurate than standard HIV tests.**
(Choose one)

1	Strongly disagree
2	Disagree
3	Neither agree nor disagree
4	Agree
5	Strongly agree
8	Refuse to Answer

Q309. I would <u>not</u> want to use an HIV self-test because **I would be worried about using the test incorrectly.**
(Choose one)

1	Strongly disagree
2	Disagree
3	Neither agree nor disagree
4	Agree
5	Strongly agree
8	Refuse to Answer

Q310. I would <u>not</u> want to use an HIV self-test because **I would be worried about misinterpreting the test result.**
(Choose one)

1	Strongly disagree
2	Disagree
3	Neither agree nor disagree
4	Agree
5	Strongly agree
8	Refuse to Answer

If Q305 is equal to 3 or Q305 is equal to 4 or Q305 is equal to 5 then skip to instruction before Q311

If not, then skip to instruction before Q314.

READ: READ TO THE RESPONDENT: Now I am going to read to you a list of reasons that motivate some people to use HIV self-tests. Please indicate the extent to which you agree or disagree with each statement.

Q311. I would be motivated to use HIV self-tests **because I would be able to test for HIV more regularly.** (Choose one)

1 Strongly disagree

2 Disagree

3 Neither agree nor disagree

4 Agree

5 Strongly agree

8 Refuse to Answer

Q312. I would be motivated to use an HIV self-test **because it would be more convenient than going to a clinic or community-based organization for a standard HIV test.** (Choose one)

1 Strongly disagree

2 Disagree

3 Neither agree nor disagree

4 Agree

5 Strongly agree

8 Refuse to Answer

Q313. I would be motivated to use an HIV self-test **because it would give me more privacy and help ensure the confidentiality of my HIV test result.** (Choose one)

1 Strongly disagree

2 Disagree

3 Neither agree nor disagree

4 Agree

5 Strongly agree

8 Refuse to Answer

Methadone, Buprenorphine, Suboxone Stigma Section

READ: READ TO THE RESPONDENT: The next questions will be about access to drug rehabilitation treatment. We talked about this in your initial visit a bit. Can you remind of your experiences with drug rehabilitation programs?

Q314. Have you ever been enrolled in a Methadone program? **(Interviewer: If participant says, "Don't know," READ: Methadone is an oral replacement therapy, which means the medication is given to you orally to help you deal with your addiction).**

1 Yes

0 No

8 Refuse to Answer

If Q314 is equal to 1 then skip to Q315

If not, then skip to Q316.

Q315. Are you currently enrolled in a Methadone program?

1 Yes

0 No

8 Refuse to Answer

Q316. Have you ever been enrolled in a Buprenorphine/ Suboxone program? **(Interviewer: If participant says, "Don't know," READ: Buprenorphine/Suboxone is an oral replacement therapy similar to methadone, which means the medication is given to you orally to help you deal with your addiction)**

1 Yes

0 No

8 Refuse to Answer

If Q316 is equal to 1 then skip to Q317

If not, then skip to instruction before Q318.

Q317. Are you currently enrolled in a Buprenorphine/Suboxone program?

1 Yes

0 No

8 Refuse to Answer

Methadone Maintenance Treatment Stigma Mechanism Scale (MMT-SMS)

If Q315 is equal to 1 and Q15 is equal to 1 then skip to instruction before Q318

If not, then skip to instruction before Q343.

READ: READ TO THE RESPONDENT: The following questions ask about receiving medications for opioid dependence like methadone, maintenance treatment. Please think about each question and circle your answer. The first group of questions asks you about how people will treat you in the future because of your methadone maintenance treatment. The second group of questions asks you about how people have treated you in the past because of your methadone maintenance treatment. The third group of questions asks about how you feel about your methadone maintenance treatment.

READ: READ THE FOLLOWING TO THE RESPONDENT AND THEN MOVE ON TO THE NEXT SCREEN AND READ THE RELATED STATEMENTS: How often have people treated you this way in the past because you receive methadone?

ENACTED SCALE ITEMS (ES1-ES9)

Q318. Family members have thought that I'm still a drug user. (Choose one)

1 Never

2 Not often

3 Somewhat often

4 Often

5 Very often

8 Refuse to Answer

Q319. Family members have not supported my methadone treatment. (Choose one)

1 Never

2 Not often

3 Somewhat often

4 Often

5 Very often

8 Refuse to Answer

Q320. Family members have thought that I cannot recover. (Choose one)

1 Never
2 Not often
3 Somewhat often
4 Often
5 Very often
8 Refuse to Answer

Q321. Employers have thought that I'm still a drug user. (Choose one)

1 Never
2 Not often
3 Somewhat often
4 Often
5 Very often
8 Refuse to Answer

Q322. Employers have thought that I'm a bad employee. (Choose one)

1 Never
2 Not often
3 Somewhat often
4 Often
5 Very often
8 Refuse to Answer

Q323. Employers have thought that managing my methadone dosage schedule will be a problem. (Choose one)

1 Never
2 Not often
3 Somewhat often
4 Often
5 Very often
8 Refuse to Answer

Q324. Healthcare workers have thought that I'm still a drug user. (Choose one)

1 Never
2 Not often
3 Somewhat often
4 Often
5 Very often
8 Refuse to Answer

Q325. Healthcare workers have given me poor treatment. (Choose one)

1 Never
2 Not often
3 Somewhat often
4 Often
5 Very often
8 Refuse to Answer

Q326. Healthcare workers have not prescribed me medication that I needed. (Choose one)

1 Never
2 Not often
3 Somewhat often
4 Often
5 Very often
8 Refuse to Answer

READ: READ THE FOLLOWING TO THE ESPONDENT AND THEN MOVE ON TO THE NEXT SCREEN AND READ THE RELATED STATEMENTS: How likely is it that people will treat you in the following ways in the future because you receive methadone?

ANTICIPATED SCALE ITEMS (AS1-AS9)

Q327. Family members will think that I'm still a drug user. (Choose one)

1 Never
2 Not often
3 Somewhat often
4 Often
5 Very often
8 Refuse to Answer

Q328. Family members will not support my methadone treatment. (Choose one)

1 Never
2 Not often
3 Somewhat often
4 Often
5 Very often
8 Refuse to Answer

Q329.	Family members will think that I cannot recover. (Choose one)	1	Never
		2	Not often
		3	Somewhat often
		4	Often
		5	Very often
		8	Refuse to Answer
Q330.	Employers will think that I'm still a drug user. (Choose one)	1	Never
		2	Not often
		3	Somewhat often
		4	Often
		5	Very often
		8	Refuse to Answer
Q331.	Employers will think that I'm a bad employee. (Choose one)	1	Never
		2	Not often
		3	Somewhat often
		4	Often
		5	Very often
		8	Refuse to Answer

Q332. Employers will think that managing my methadone treatment schedule will be a problem. (Choose one)

1 Never
2 Not often
3 Somewhat often
4 Often
5 Very often
8 Refuse to Answer

Q333. Healthcare workers will think that I'm still a drug user. (Choose one)

1 Never
2 Not often
3 Somewhat often
4 Often
5 Very often
8 Refuse to Answer

Q334. Healthcare workers will give me poor care. (Choose one)

1 Never
2 Not often
3 Somewhat often
4 Often
5 Very often
8 Refuse to Answer

Q335. Healthcare workers will not prescribe me medication that I need. (Choose one)

1 Never
2 Not often
3 Somewhat often
4 Often
5 Very often
8 Refuse to Answer

INTERNALIZED STIGMA ITEMS (IS1-IS7)

Q336. Receiving methadone makes me feel like I'm a bad person. (Choose one)

1 Never
2 Not often
3 Somewhat often
4 Often
5 Very often
8 Refuse to Answer

Q337. I feel I'm not as good as others because I receive methadone. (Choose one)

1 Never
2 Not often
3 Somewhat often
4 Often
5 Very often
8 Refuse to Answer

Q338.	I feel ashamed of my methadone treatment. (Choose one)	1	Never
		2	Not often
		3	Somewhat often
		4	Often
		5	Very often
		8	Refuse to Answer
Q339.	I think less of myself because I receive methadone. (Choose one)	1	Never
		2	Not often
		3	Somewhat often
		4	Often
		5	Very often
		8	Refuse to Answer
Q340.	Receiving methadone makes me feel unclean. (Choose one)	1	Never
		2	Not often
		3	Somewhat often
		4	Often
		5	Very often
		8	Refuse to Answer
Q341.	Being on methadone treatment is disgusting to me. (Choose one)	1	Never
		2	Not often
		3	Somewhat often
		4	Often
		5	Very often
		8	Refuse to Answer

Q342. Being on methadone treatment makes me feel like I'm still a drug user. (Choose one)

1	Never
2	Not often
3	Somewhat often
4	Often
5	Very often
8	Refuse to Answer

NEVER on MMT Methadone Maintenance Treatment Stigma (18 items)

METHADONE MAINTENANCE TREATMENT STIGMA (18 items)-MMTS1-MMTS18. Note: MMTS17 is reversed coded.

If Q314 is not equal to 1 then skip to instruction before Q343

If not, then skip to instruction before Q361.

READ: Interviewer, read to the respondent: Methadone is a medication that can be taken to treat opioid dependence. Methadone is medication that treats heroin addiction and other opioids. It reduces withdrawal symptoms and drug cravings, but won't' make you feel high. These questions will ask about how you might be treated in the future if you were to take methadone. Please select one response option for each question. Do not spend too much time considering your answer. Your first impression is usually best.

Q343. How concerned are you that you will be denied housing if you take methadone? **(Choose one)** (Choose one)

1 Not at all

2 A little bit

3 Somewhat

4 Quite a bit

5 Extremely

8 Refuse to Answer

Q344. How concerned are you that you will be denied employment if you take methadone? **(Choose one)** (Choose one)

1 Not at all

2 A little bit

3 Somewhat

4 Quite a bit

5 Extremely

8 Refuse to Answer

Q345. How concerned are you that you will be denied government assistance or similar benefits if you take methadone? **(Choose one)** (Choose one)

1 Not at all

2 A little bit

3 Somewhat

4 Quite a bit

5 Extremely

8 Refuse to Answer

READ: READ THE FOLLOWING TO THE RESPONDENT AND THEN MOVE ON TO THE NEXT SCREEN AND READ THE RELATED STATEMENTS: How likely is it that people will treat you in the following ways in the future if you were to take methadone?

Q346. Family members will think that Im still a drug user. **(Choose one)** (Choose one)

1 Never

2 Not often

3 Somewhat often

4 Often

5 Very often

8 Refuse to Answer

Q347. Family members will not support my methadone treatment. **(Choose one)** (Choose one)

1 Never

2 Not often

3 Somewhat often

4 Often

5 Very often

8 Refuse to Answer

Q348. Family members will think that I cannot recover. **(Choose one)** (Choose one)

1 Never

2 Not often

3 Somewhat often

4 Often

5 Very often

8 Refuse to Answer

Q349. Healthcare workers will think that Im still a drug user. **(Choose one)** (Choose one)

1 Never

2 Not often

3 Somewhat often

4 Often

5 Very often

8 Refuse to Answer

Q350. Healthcare workers will give me poor care. **(Choose one)** (Choose one)

1 Never

2 Not often

3 Somewhat often

4 Often

5 Very often

8 Refuse to Answer

Q351. Healthcare workers will not prescribe me medication that I need. **(Choose one)** (Choose one)

1 Never

2 Not often

3 Somewhat often

4 Often

5 Very often

8 Refuse to Answer

Q352. Other people who inject drugs will think Im weak if I take methadone. **(Choose one)** (Choose one)

1 Never

2 Not often

3 Somewhat often

4 Often

5 Very often

8 Refuse to Answer

Q353. Other people who inject drugs will not support me if I take methadone. **(Choose one)** (Choose one)

1 Never

2 Not often

3 Somewhat often

4 Often

5 Very often

8 Refuse to Answer

Q354. Other people who inject drugs wont trust me if I take methadone. **(Choose one)** (Choose one)

1 Never

2 Not often

3 Somewhat often

4 Often

5 Very often

8 Refuse to Answer

READ: READ THE FOLLOWING TO THE RESPONDENT AND THEN MOVE ON TO THE NEXT SCREEN AND READ THE RELATED STATEMENTS: How do you feel about people who take methadone?

Q355. People who take methadone are replacing one drug for another. **(Choose one)** (Choose one)

1 Strongly disagree

2 Disagree

3 Neither disagree nor agree

4 Agree

5 Strongly agree

8 Refuse to Answer

Q356. People who take methadone cannot be trusted. **(Choose one)** (Choose one)

1 Strongly disagree

2 Disagree

3 Neither disagree nor agree

4 Agree

5 Strongly agree

8 Refuse to Answer

Q357. People who take methadone <u>are not in control of their own life</u>. **(Choose one)** (Choose one)

1 Strongly disagree

2 Disagree

3 Neither disagree nor agree

4 Agree

5 Strongly agree

8 Refuse to Answer

Q358. People who take methadone make me feel <u>uncomfortable</u>. **(Choose one)** (Choose one)

1 Strongly disagree

2 Disagree

3 Neither disagree nor agree

4 Agree

5 Strongly agree

8 Refuse to Answer

Q359. People who take methadone make me feel <u>hopeful.</u> **(Choose one)** (Choose one)

1 Strongly agree

2 Agree

3 Neither disagree nor agree

4 Agree

5 Strongly agree

8 Refuse to Answer

Q360. People who take methadone make me feel angry. **(Choose one)** (Choose one)

1	Strongly disagree
2	Disagree
3	Neither disagree nor agree
4	Agree
5	Strongly agree
8	Refuse to Answer

Buprenorphine/Suboxone Maintenance Treatment Stigma Mechanism Scale (BSMT-SMS)

If Q317 is equal to 1 and Q15 is equal to 1 then skip to instruction before Q361

If not, then skip to instruction before Q386.

READ: Interviewer, READ TO THE RESPONDENT:The following questions ask about receiving medications for opioid dependence like buprenorphine or Suboxone maintenance treatment. Please think about each question and circle your answer. The first group of questions asks you about how people will treat you in the future because of your buprenorphine or Suboxone maintenance treatment. The second group of questions asks you about how people have treated you in the past because of your buprenorphine or Suboxone maintenance treatment. The third group of questions asks about how you feel about your buprenorphine or Suboxone maintenance treatment.

READ: READ THE FOLLOWING TO THE ESPONDENT AND THEN MOVE ON TO THE NEXT SCREEN AND READ THE RELATED STATEMENTS: How often have people treated you this way in the past because you receive buprenorphine or Suboxone?

ENACTED STIGMA ITEMS (BSES1-BSES9)

Q361. Family members have thought that Im still a drug user. (Choose one)

1	Never
2	Not often
3	Somewhat often
4	Often
5	Very often
8	Refuse to Answer

Q362. Family members have not supported my buprenorphine/ Suboxone treatment. (Choose one)

1	Never
2	Not often
3	Somewhat often
4	Often
5	Very often
8	Refuse to Answer

Q363. Family members have thought that I cannot recover. (Choose one)

1	Never
2	Not often
3	Somewhat often
4	Often
5	Very often
8	Refuse to Answer

Q364. Employers have thought that Im still a drug user. (Choose one)

1	Never
2	Not often
3	Somewhat often
4	Often
5	Very often
8	Refuse to Answer

Q365. Employers have thought that Im a bad employee. (Choose one)

1 Never
2 Not often
3 Somewhat often
4 Often
5 Very often
8 Refuse to Answer

Q366. Employers have thought that managing my buprenorphine/ Suboxone dosage schedule will be a problem. (Choose one)

1 Never
2 Not often
3 Somewhat often
4 Often
5 Very often
8 Refuse to Answer

Q367. Healthcare workers have thought that Im still a drug user. (Choose one)

1 Never
2 Not often
3 Somewhat often
4 Often
5 Very often
8 Refuse to Answer

Q368. Healthcare workers have given me poor treatment. (Choose one)

1 Never

2 Not often

3 Somewhat often

4 Often

5 Very often

8 Refuse to Answer

Q369. Healthcare workers have not prescribed me medication that I needed. (Choose one)

1 Never

2 Not often

3 Somewhat often

4 Often

5 Very often

8 Refuse to Answer

READ: READ THE FOLLOWING TO THE RESPONDENT AND THEN MOVE ON TO THE NEXT SCREEN AND READ THE RELATED STATEMENTS: How likely is it that people will treat you in the following ways in the future because you receive buprenorphine or Suboxone?

ANTICIPATED STIGMA ITEMS (BSAS1-BSAS9)

Q370. Family members will think that Im still a drug user. (Choose one)

1 Never

2 Not often

3 Somewhat often

4 Often

5 Very often

8 Refuse to Answer

Q371. Family members will not support my buprenorphine/ Suboxone treatment. (Choose one)

1 Never
2 Not often
3 Somewhat often
4 Often
5 Very often
8 Refuse to Answer

Q372. Family members will think that I cannot recover. (Choose one)

1 Never
2 Not often
3 Somewhat often
4 Often
5 Very often
8 Refuse to Answer

Q373. Employers will think that Im still a drug user. (Choose one)

1 Never
2 Not often
3 Somewhat often
4 Often
5 Very often
8 Refuse to Answer

Q374. Employers will think that Im a bad employee. (Choose one)

1 Never
2 Not often
3 Somewhat often
4 Often
5 Very often
8 Refuse to Answer

Q375. Employers will think that managing my buprenorphine/ Suboxone treatment schedule will be a problem. (Choose one)

1 Never
2 Not often
3 Somewhat often
4 Often
5 Very often
8 Refuse to Answer

Q376. Healthcare workers will think that Im still a drug user. (Choose one)

1 Never
2 Not often
3 Somewhat often
4 Often
5 Very often
8 Refuse to Answer

Q377. Healthcare workers will give me poor care. (Choose one)

1 Never
2 Not often
3 Somewhat often
4 Often
5 Very often
8 Refuse to Answer

Q378. Healthcare workers will not prescribe me medication that I need. (Choose one)

1 Never
2 Not often
3 Somewhat often
4 Often
5 Very often
8 Refuse to Answer

INTERNALIZED STIGMA ITEMS (BSIS1-BSIS7)

Q379. Receiving buprenorphine/saboxone makes me feel like Im a bad person. (Choose one)

1 Never
2 Not often
3 Somewhat often
4 Often
5 Very often
8 Refuse to Answer

Q380. I feel Im not as good as others because I receive buprenorphine/saboxone. (Choose one)

1 Never
2 Not often
3 Somewhat often
4 Often
5 Very often
8 Refuse to Answer

Q381. I feel ashamed of my buprenorphine/saboxone treatment. (Choose one)

1 Never
2 Not often
3 Somewhat often
4 Often
5 Very often
8 Refuse to Answer

Q382. I think less of myself because I receive buprenorphine/saboxone. (Choose one)

1 Never
2 Not often
3 Somewhat often
4 Often
5 Very often
8 Refuse to Answer

Q383. Receiving buprenorphine/ saboxone makes me feel unclean. (Choose one)

1 Never
2 Not often
3 Somewhat often
4 Often
5 Very often
8 Refuse to Answer

Q384. Being on buprenorphine/ saboxone treatment is disgusting to me. (Choose one)

1 Never
2 Not often
3 Somewhat often
4 Often
5 Very often
8 Refuse to Answer

Q385. Being on buprenorphine/ saboxone treatment makes me feel like Im still a drug user. (Choose one)

1 Never
2 Not often
3 Somewhat often
4 Often
5 Very often
8 Refuse to Answer

NEVER on B/Smt BUPRENORPHINE/ SUBOXONE Maintenance Treatment Stigma (18 items)

If Q316 is not equal to 1 and Q15 is equal to 1 then skip to instruction before Q386

If not, then skip to Q404.

READ: Interviewer, READ TO THE RESPONDENT: Buprenorphine and Suboxone are medications that treat heroin addiction and other opioids. They reduce withdrawal symptoms and drug cravings, but won't' make you feel high. These questions will ask about how you might be treated in the future if you were to take buprenorphine or Suboxone.
Please select one response option for each question. Do not spend too much time considering your answer. Your first impression is usually best.

BUPRENORPHINE/ SUBOXONE MAINTENANCE TREATMENT STIGMA (18 items)-BMTS1-BMTS18. Note: BMTS17 is reversed coded.

Q386. How concerned are you that you will be denied housing if you take buprenorphine/ Suboxone? (Choose one)

1 Not at all
2 A little bit
3 Somewhat
4 Quite a bit
5 Extremely
8 Refuse to Answer

Q387. How concerned are you that you will be denied employment if you take buprenorphine/ Suboxone? (Choose one)

1 Not at all
2 A little bit
3 Somewhat
4 Quite a bit
5 Extremely
8 Refuse to Answer

Q388. **How concerned are you that you will be denied <u>government assistance or similar benefits</u> if you take buprenorphine/ Suboxone?** (Choose one)

1 Not at all

2 A little bit

3 Somewhat

4 Quite a bit

5 Extremely

8 Refuse to Answer

READ: READ THE FOLLOWING TO THE RESPONDENT AND THEN MOVE ON TO THE NEXT SCREEN AND READ THE RELATED STATEMENTS: How likely is it that people will treat you in the following ways in the <u>future</u> if you were to take buprenorphine/ Suboxone?

Q389. Family members will think that Im still a drug user. (Choose one)

1 Never

2 Not often

3 Somewhat often

4 Often

5 Very often

8 Refuse to Answer

Q390. Family members will not support my buprenorphine/ Suboxone treatment. (Choose one)

1 Never

2 Not often

3 Somewhat often

4 Often

5 Very often

8 Refuse to Answer

Q391. Family members will think that I cannot recover. (Choose one)

1 Never
2 Not often
3 Somewhat often
4 Often
5 Very often
8 Refuse to Answer

Q392. Healthcare workers will think that Im still a drug user. (Choose one)

1 Never
2 Not often
3 Somewhat often
4 Often
5 Very often
8 Refuse to Answer

Q393. Healthcare workers will give me poor care. (Choose one)

1 Never
2 Not often
3 Somewhat often
4 Often
5 Very often
8 Refuse to Answer

Q394. Healthcare workers will not prescribe me medication that I need. (Choose one)

1 Never
2 Not often
3 Somewhat often
4 Often
5 Very often
8 Refuse to Answer

Q395. Other people who inject drugs will think Im weak if I take buprenorphine/ Suboxone. (Choose one)

1 Never
2 Not often
3 Somewhat often
4 Often
5 Very often
8 Refuse to Answer

Q396. Other people who inject drugs will not support me if I take buprenorphine/ Suboxone. (Choose one)

1 Never
2 Not often
3 Somewhat often
4 Often
5 Very often
8 Refuse to Answer

Q397. Other people who inject drugs wont trust me if I take buprenorphine/ Suboxone. (Choose one)

1 Never
2 Not often
3 Somewhat often
4 Often
5 Very often
8 Refuse to Answer

READ: READ THE FOLLOWING TO THE RESPONDENT AND THEN MOVE ON TO THE NEXT SCREEN AND READ THE RELATED STATEMENTS: How do you feel about people who take buprenorphine/ Suboxone?

Q398. People who take buprenorphine/ Suboxone are replacing one drug for another. (Choose one)

1 Strongly disagree
2 Disagree
3 Neither disagree nor agree
4 Agree
5 Strongly agree
8 Refuse to Answer

Q399. People who take buprenorphine/ Suboxone cannot be trusted. (Choose one)

1 Strongly disagree
2 Disagree
3 Neither disagree nor agree
4 Agree
5 Strongly agree
8 Refuse to Answer

Q400. People who take buprenorphine/ Suboxone are not in control of their own life. (Choose one)

1 Strongly disagree
2 Disagree
3 Neither disagree nor agree
4 Agree
5 Strongly agree
8 Refuse to Answer

Q401. People who take buprenorphine/ Suboxone make me feel uncomfortable. (Choose one)

1 Strongly disagree
2 Disagree
3 Neither disagree nor agree
4 Agree
5 Strongly agree
8 Refuse to Answer

Q402. People who take buprenorphine/ Suboxone make me feel hopeful. (Choose one)

1 Strongly disagree
2 Disagree
3 Neither disagree nor agree
4 Agree
5 Strongly agree
8 Refuse to Answer

Q403. People who take buprenorphine/ Suboxone make me feel angry. (Choose one)

1	Strongly disagree
2	Disagree
3	Neither disagree nor agree
4	Agree
5	Strongly agree
8	Refuse to Answer

Q404. In the next section, you will be asked to list the names of people in your social network, along with questions about them such as your relationship to them, and the sexual and drug use practices of these people who are not in the study (i.e. they have not provided consent). This information is very important for improving disease prevention strategies to reduce HIV transmission in communities. However, there is a possibility that your sharing of information about members of your social network without their consent will become known to them and/or the community through speculation. We would like to assure you that we will never contact anyone you name during the survey under any circumstance, and we will never share this information with anyone outside of the people working on this study. The information you share with us is private and confidential, and we will make every effort to safeguard such information from unauthorized access or dissemination. If you feel uncomfortable about providing names, you may provide nicknames or initials instead, or opt out of individual questions. **Do we have your consent to proceed to these questions?**

1	Yes
0	No
8	Refuse to Answer

If Q404 is equal to 1 then skip to instruction before Q405

If not, then skip to Q1150.

READ: Interviewer, SAY TO THE RESPONDENT: For this survey we will be talking about people you hang out with frequently and that you consider friends. I'm going to ask you about people you know well. We are trying to learn about how people are connected to one another and how those connections relate to preventing HIV infection and other diseases. Having names and demographic information will help us see how people in this study are connected to one another, for example through mutual friends. Please keep in mind that everything you tell me is confidential and that we will protect your privacy and the privacy of the people you mention. We will never at any time use this information to contact the people you tell us about.

Specifically, I'm going to ask you about how many people you know who you have seen in the past 30 days who you talk to about things that are important to you. These people could be your partner (girlfriend/boyfriend, wife/husband), family, friends, people you use drugs with, or anyone else you consider yourself close with. For example, a person that reminds you to take medication, someone you ask to borrow money, someone you pool drugs or money with, someone you use drugs with, or someone you talk with about drug use or personal issues. Remember, we are only interested in those people you have seen in the past 30 days.

Q405. Please tell me how many friends or people who you associate with and talk to about things that are important to you, have you seen in the past 30 days?

— — —	people
998	Refuse to Answer

If Q405 is equal to 0 or Q405 is equal to 998, then skip to instruction before Q686.

READ: What are the names of these people? Please start with the one closest to you. Interviewer: In the screens that follow, you'll be prompted to enter the names.

If Q405 is greater than 0 then skip to Q406

If not, then skip to instruction before Q408.

Q406. What is the name of the person closest to you that you talked to about things that are important to you and you have seen in the past 30 days? **Interviewer: IF THE PARTICIPANT IS RELUCTANT TO GIVE FULL NAME READ THE FOLLOWING TO THEM: If you do not want to give us their full name you can use initials or just their First name or a nickname, so that you will know who we are talking about when I ask you follow up questions about this person. If the participant gives ONLY a nickname or just initials, enter it here and on the next screen.**

— —

If Q406 is equal to "Refuse to Answer", then skip to instruction before Q446.

Q407. Can you tell me [Response to Q406]'s nickname if they have one? Interviewer, please record the nickname.

— —

If Q405 is greater than 1 then skip to Q408

If not, then skip to instruction before Q410.

Q408. So, the name of the first person that you talked to about things that are important to you and you have seen in the past 30 days is [Response to Q406]. What is the name of the 2nd person? **Interviewer: If the participant is having a hard time naming anyone, read to them: "Please think back to the last time or two you talked with a family member, friend or partner about things that are important to you. Can you recall their names?" IF THE PARTICIPANT IS RELUCTANT TO GIVE THE PERSON'S FULL NAME, tell them that they can use initials or just the first name or a nickname.**

——————————————————————————————

If Q408 is equal to "Refuse to Answer", then skip to instruction before Q446.

Q409. Can you tell me [Response to Q408]'s nickname if they have one? Interviewer, please record the nickname.

——————————————————————————————

If Q405 is greater than 2 then skip to Q410

If not, then skip to instruction before Q412.

Q410. So far you named [Response to Q406] and [Response to Q408]. What is the name of the 3rd person? **Interviewer: If the participant is having a hard time naming anyone, read to them: "Please think back to the last time or two you talked with a family member, friend or partner about things that are important to you. Can you recall their names?" IF THE PARTICIPANT IS RELUCTANT TO GIVE THE PERSON'S FULL NAME, tell them that they can use initials or just the first name or a nickname.**

——————————————————————————————

If Q410 is equal to "Refuse to Answer", then skip to instruction before Q446.

Q411. Can you tell me [Response to Q410]'s nickname if they have one? Interviewer, please record the nickname.

——————————————————————————————

If Q405 is greater than 3 then skip to Q412

If not, then skip to instruction before Q414.

Q412. So far you named [Response to Q406], [Response to Q408] and [Response to Q410]. What is the name of the 4th person? **Interviewer: If the participant is having a hard time naming anyone, read to them: "Please think back to the last time or two you talked with a family member, friend or partner about things that are important to you. Can you recall their names?" IF THE PARTICIPANT IS RELUCTANT TO GIVE THE PERSON'S FULL NAME, tell them that they can use initials or just the first name or a nickname.**

____

If Q412 is equal to "Refuse to Answer", then skip to instruction before Q446.

Q413. Can you tell me [Response to Q412]'s nickname if they have one? Interviewer, please record the nickname.

____

If Q405 is greater than 4 then skip to Q414

If not, then skip to instruction before Q416.

Q414. So far you named [Response to Q406], [Response to Q408], [Response to Q410] and [Response to Q412]. What is the name of the 5th person? **Interviewer: If the participant is having a hard time naming anyone, read to them: "Please think back to the last time or two you talked with a family member, friend or partner about things that are important to you. Can you recall their names?" IF THE PARTICIPANT IS RELUCTANT TO GIVE THE PERSON'S FULL NAME, tell them that they can use initials or just the first name or a nickname.**

____

If Q414 is equal to "Refuse to Answer", then skip to instruction before Q446.

Q415. Can you tell me [Response to Q414]'s nickname if they have one? Interviewer, please record the nickname.

____

If Q405 is greater than 5 then skip to Q416

If not, then skip to instruction before Q418.

Q416. So far you named [Response to Q406], [Response to Q408], [Response to Q410], [Response to Q412], and [Response to Q414]. Can you tell me what the name of the 6th person is?

____

If Q416 is equal to "Refuse to Answer", then skip to instruction before Q446.

Q417. Can you tell me [Response to Q416]'s nickname if they have one? Interviewer, please record the nickname.

____

If Q405 is greater than 6 then skip to Q418

If not, then skip to instruction before Q420.

Q418. So far you named [Response to Q406], [Response to Q408], [Response to Q410], [Response to Q412], [Response to Q414], and [Response to Q416]. What is the name of the 7th person.

____

If Q418 is equal to "Refuse to Answer", then skip to instruction before Q446.

Q419. Can you tell me [Response to Q418]'s nickname if they have one? Interviewer, please record the nickname.

____

If Q405 is greater than 7 then skip to Q420

If not, then skip to instruction before Q422.

Q420. So far you named [Response to Q406], [Response to Q408], [Response to Q410], [Response to Q412], [Response to Q414], [Response to Q416] and [Response to Q418]. What is the name of the 8th person?

____

If Q420 is equal to "Refuse to Answer", then skip to instruction before Q446.

Q421. Can you tell me [Response to Q420]'s nickname if they have one? Interviewer, please record the nickname.

____

If Q405 is greater than 8 then skip to Q422

If not, then skip to instruction before Q424.

Q422. So far you named [Response to Q406], [Response to Q408], [Response to Q410], [Response to Q412], [Response to Q414], [Response to Q416], [Response to Q418] and [Response to Q420]. What is the name of the 9th person?

____

If Q422 is equal to "Refuse to Answer", then skip to instruction before Q446.

Q423. Can you tell me [Response to Q422]'s nickname if they have one? Interviewer, please record the nickname.

____

If Q405 is greater than 9 then skip to Q424

If not, then skip to instruction before Q426.

Q424. So far you named [Response to Q406], [Response to Q408], [Response to Q410], [Response to Q412], [Response to Q414], [Response to Q416], [Response to Q418], [Response to Q420], and [Response to Q422]. What is the name of the 10th person.

____

If Q424 is equal to "Refuse to Answer", then skip to instruction before Q446.

Q425. Can you tell me [Response to Q424]'s nickname if they have one? Interviewer, please record the nickname.

____

If Q405 is greater than 10 then skip to Q426

If not, then skip to instruction before Q428.

Q426. So far you named [Response to Q406], [Response to Q408], [Response to Q410], [Response to Q412], [Response to Q414], [Response to Q416], [Response to Q418], [Response to Q420], [Response to Q422], and [Response to Q424]. What is the name of the 11th person?

____

If Q426 is equal to "Refuse to Answer", then skip to instruction before Q446.

Q427. Can you tell me [Response to Q426]'s nickname if they have one? Interviewer, please record the nickname.

____

If Q405 is greater than 11 then skip to Q428

If not, then skip to instruction before Q430.

Q428. So far you named [Response to Q406], [Response to Q408], [Response to Q410], [Response to Q412], [Response to Q414] ,[Response to Q416], [Response to Q418], [Response to Q420], [Response to Q422], [Response to Q424] and [Response to Q426]. What is the name of the 12th person?

____

If Q428 is equal to "Refuse to Answer", then skip to instruction before Q446.

Q429. Can you tell me [Response to Q428]'s nickname if they have one? Interviewer, please record the nickname.

____

If Q405 is greater than 12 then skip to Q430

If not, then skip to instruction before Q432.

Q430. So far you named [Response to Q406], [Response to Q408], [Response to Q410], [Response to Q412], [Response to Q414] ,[Response to Q416], [Response to Q418], [Response to Q420], [Response to Q422], [Response to Q424], [Response to Q426], and [Response to Q428]. What is the name of the 13th person?

____

If Q430 is equal to "Refuse to Answer", then skip to instruction before Q446.

Q431. Can you tell me [Response to Q430]'s nickname if they have one? Interviewer, please record the nickname.

____

If Q405 is greater than 13 then skip to Q432

If not, then skip to instruction before Q434.

Q432. So far you named [Response to Q406], [Response to Q408], [Response to Q410], [Response to Q412], [Response to Q414] ,[Response to Q416], [Response to Q418], [Response to Q420], [Response to Q422], [Response to Q424], [Response to Q426], [Response to Q428] and [Response to Q430]. What is the name of the 14th person?

____

If Q432 is equal to "Refuse to Answer", then skip to instruction before Q446.

Q433. Can you tell me [Response to Q432]'s nickname if they have one? Interviewer, please record the nickname.

____

If Q405 is greater than 14 then skip to Q434

If not, then skip to instruction before Q436.

Q434. So far you named [Response to Q406], [Response to Q408], [Response to Q410], [Response to Q412], [Response to Q414], [Response to Q416], [Response to Q418], [Response to Q420], [Response to Q422], [Response to Q424], [Response to Q426], [Response to Q428], [Response to Q430] and [Response to Q432]. What is the name of the 15th person?

____

If Q434 is equal to "Refuse to Answer", then skip to instruction before Q446.

Q435. Can you tell me [Response to Q434]'s nickname if they have one? Interviewer, please record the nickname.

____

If Q405 is greater than 15 then skip to Q436

If not, then skip to instruction before Q438.

Q436. So far you named [Response to Q406], [Response to Q408], [Response to Q410], [Response to Q412], [Response to Q414], [Response to Q416], [Response to Q418], [Response to Q420], [Response to Q422], [Response to Q424], [Response to Q426], [Response to Q428], [Response to Q430], [Response to Q432] and [Response to Q434]. What is the name of the 16th person?

____

If Q436 is equal to "Refuse to Answer", then skip to instruction before Q446.

Q437. Can you tell me [Response to Q436]'s nickname if they have one? Interviewer, please record the nickname.

____

If Q405 is greater than 16 then skip to Q438

If not, then skip to instruction before Q440.

Q438. So far you named [Response to Q406], [Response to Q408], [Response to Q410], [Response to Q412], [Response to Q414], [Response to Q416], [Response to Q418], [Response to Q420], [Response to Q422], [Response to Q424], [Response to Q426], [Response to Q428], [Response to Q430], [Response to Q432], [Response to Q434] and [Response to Q436]. What is the name of the 17th person?

____

If Q438 is equal to "Refuse to Answer", then skip to instruction before Q446.

Q439. Can you tell me [Response to Q438]'s nickname if they have one? Interviewer, please record the nickname.

____

If Q405 is greater than 17 then skip to Q440

If not, then skip to instruction before Q442.

Q440. So far you named [Response to Q406], [Response to Q408], [Response to Q410], [Response to Q412], [Response to Q414], [Response to Q416], [Response to Q418], [Response to Q420], [Response to Q422], [Response to Q424], [Response to Q426], [Response to Q428], [Response to Q430], [Response to Q432], [Response to Q434], [Response to Q436] and [Response to Q438]. What is the name of the 18th person?

____

If Q440 is equal to "Refuse to Answer", then skip to instruction before Q446.

Q441. Can you tell me [Response to Q440]'s nickname if they have one? Interviewer, please record the nickname.

____

If Q405 is greater than 18 then skip to Q442

If not, then skip to instruction before Q444.

Q442. So far you named [Response to Q406], [Response to Q408], [Response to Q410], [Response to Q412], [Response to Q414], [Response to Q416], [Response to Q418], [Response to Q420], [Response to Q422], [Response to Q424], [Response to Q426], [Response to Q428], [Response to Q430], [Response to Q432], [Response to Q434], [Response to Q436], [Response to Q438], and [Response to Q440]. What is the name of the 19th person?

____

If Q442 is equal to "Refuse to Answer", then skip to instruction before Q446.

Q443. Can you tell me [Response to Q442]'s nickname if they have one? Interviewer, please record the nickname.

____

If Q405 is greater than 19 then skip to Q444

If not, then skip to instruction before Q446.

Q444. So far you named [Response to Q406], [Response to Q408], [Response to Q410], [Response to Q412], [Response to Q414], [Response to Q416], [Response to Q418], [Response to Q420], [Response to Q422], [Response to Q424], [Response to Q426], [Response to Q428], [Response to Q430], [Response to Q432], [Response to Q434], [Response to Q436], [Response to Q438], [Response to Q440], and [Response to Q442]. What is the name of the 20th person?

____

If Q444 is equal to "Refuse to Answer", then skip to instruction before Q446.

Q445. Can you tell me [Response to Q444]'s nickname if they have one? Interviewer, please record the nickname.

____

READ: Now I will ask questions about the first five people you have listed. These questions are about basic information. Keep in mind that everything you tell me is confidential and that we will protect your privacy and the privacy of the people that you mention.

If Q405 is greater than 0 and Q406 is not equal to "Refuse to Answer" then skip to Q446

If not, then skip to instruction before Q468.

Q446. What is [Response to Q406]'s age?

__ __	years old
97	Don't Know
98	Refuse to Answer

Q447. How long have you known [Response to Q406]?

__ __	YEARS
__ __	MONTHS
__ __	DAYS
97	Don't Know (Years)
98	Refuse to Answer (Years)

If Q447 is greater than Q446 then READ: "The participant said that they have known this person for longer than this person's age. You are redirected to make corrections." and skip to Q446.

Q448. What is your relationship to [Response to Q406]? (Choose one)

1	Romantic or sex partner
2	Friend
3	Neighboor or housemate
4	Co-worker or boss
5	Family - relative by blood or marriage
6	Drug or Alcohol Related
7	Other
8	Refuse to Answer

If Q448 is equal to 1 then skip to Q449

If not, then skip to instruction before Q450.

Q449. Is [Response to Q406]? (Choose one)

1 Spouse
2 Current romantic partner
3 Ex-romantic partner
4 Sex partner (non-romantic)
5 Other
8 Refuse to Answer

If Q449 is equal to 5 then skip to Q450

If not, then skip to instruction before Q451.

Q450. Please specify your relationship with [Response to Q406].

——————————————————————————————

If Q448 is equal to 2 then skip to Q451

If not, then skip to instruction before Q452.

Q451. Is [Response to Q406]? (Choose one)

1 Best Friend
2 Close Friend
3 Friend
4 Acquaintance
5 Other
8 Refuse to Answer

If Q451 is equal to 5 then skip to Q452

If not, then skip to instruction before Q453.

Q452. Please specify your relationship with [Response to Q406].

——————————————————————————————

If Q448 is equal to 3 then skip to Q453

If not, then skip to instruction before Q454.

Q453. Is [Response to Q406]? (Choose one)

1	Neighbor
2	Housemate/Roommate
3	Other
8	Refuse to Answer

If Q453 is equal to 3 then skip to Q454

If not, then skip to instruction before Q455.

Q454. Please specify your relationship with [Response to Q406].

____

If Q448 is equal to 4 then skip to Q455

If not, then skip to instruction before Q456.

Q455. Is [Response to Q406]? (Choose one)

1	Co-worker
2	Boss
3	Other
8	Refuse to Answer

If Q455 is equal to 3 then skip to Q456

If not, then skip to instruction before Q457.

Q456. Please specify your relationship with [Response to Q406].

____

If Q448 is equal to 5 then skip to Q457

If not, then skip to instruction before Q458.

Q457. Is [Response to Q406]? (Choose one)

1 Parent [Mother/Father/Step-parent]

2 Child

3 Sibling [Brother/sister/step-brother/sister]

4 Brother-in-law or Sister-in-law

5 Other Relative

8 Refuse to Answer

If Q457 is equal to 5 then skip to Q458

If not, then skip to instruction before Q459.

Q458. Please specify your relationship with [Response to Q406].

____

If Q448 is equal to 6 then skip to Q459

If not, then skip to instruction before Q460.

Q459. Is [Response to Q406]? (Choose one)

1 Bartender

2 Drug Dealer

3 Someone who injects others for a fee or for drugs

4 Drug Selling Client (someone you sell drugs to)

5 Drinking Buddy

6 Someone you use drugs with

7 Other

8 Refuse to Answer

If Q459 is equal to 7 then skip to Q460

If not, then skip to instruction before Q461.

Q460. Please specify your relationship with [Response to Q406].

____

If Q448 is equal to 7 then skip to Q461

If not, then skip to instruction before Q462.

Q461. Is [Response to Q406]? (Choose one)

1 Religious or Spiritual Advisor

2 Teacher or Other Educator

3 Medical Professional

4 Mental Health Professional

5 Other

8 Refuse to Answer

If Q461 is equal to 5 then skip to Q462

If not, then skip to instruction before Q463.

Q462. Please specify your relationship with [Response to Q406]?

____

If Q448 is not equal to 5 and Q448 is not equal to 8 then skip to Q463

If not, then skip to Q464.

Q463. Did you have vaginal or anal sex with [Response to Q406] in the previous 6 months?

1 Yes

0 No

8 Refuse to Answer

Q464. Is [Response to Q406] male, female, transgender, non-binary or other? (Choose one)

1 Male

2 Female

3 Transgender woman (i.e., male to female)

4 Transgender man (i.e., female to male)

5 Non-binary / genderqueer / gender fluid / agender

6 Another identity not listed

8 Refuse to Answer

Q465. Please tell me how often you communicate with [Response to Q406] (in person, phone, email, and on social media, like Facebook, count) (Choose one)

01 Every Day

02 Several times a week

03 Once a week

04 Once every two weeks

05 Once a month

06 A couple of times a year

07 Once a year

08 Less than once a year

98 Refuse to Answer

Q466. How close do you feel to [Response to Q406]? (Choose one)

1 Very close

2 Somewhat close

3 Not very close

4 Not close at all

8 Refuse to Answer

Q467. How long have you felt this close to [Response to Q406]?

__ __	YEARS
__ __	MONTHS
__ __	DAYS
97	Don't Know (Years)
98	Refuse to Answer (Years)

If Q467 is greater than Q447 and Q447 is not equal to 97 and Q447 is not equal to 98 then READ: "That response is greater than the time you said you have known &[NAME1]. Please check the response." and skip to Q467.

If Q405 is greater than 1 and Q408 is not equal to "Refuse to Answer" and Q408 is not equal to "skipped" then skip to Q468

If not, then skip to instruction before Q490.

Q468. What is [Response to Q408]'s age?

__ __	years old
97	Don't Know
98	Refuse to Answer

Q469. How long have you known [Response to Q408]?

__ __	YEARS
__ __	MONTHS
__ __	DAYS
97	Don't Know (Years)
98	Refuse to Answer (Years)

If Q469 is greater than Q468 then READ: "The participant said that they have known this person for longer than this person's age. You are redirected to make corrections." and skip to Q468.

Q470. What is your relationship to [Response to Q408]? (Choose one)

1 Romantic or sex partner

2 Friend

3 Neighboor or housemate

4 Co-worker or boss

5 Family - relative by blood or marriage

6 Drug or Alcohol Related

7 Other

8 Refuse to Answer

If Q470 is equal to 1 then skip to Q471

If not, then skip to instruction before Q472.

Q471. Is [Response to Q408]? (Choose one)

1 Spouse

2 Current romantic partner

3 Ex-romantic partner

4 Sex partner (non-romantic)

5 Other

8 Refuse to Answer

If Q471 is equal to 5 then skip to Q472

If not, then skip to instruction before Q473.

Q472. Please specify your relationship with [Response to Q408].

____

If Q470 is equal to 2 then skip to Q473

If not, then skip to instruction before Q474.

Q473. Is [Response to Q408]? (Choose one)

1 Best Friend
2 Close Friend
3 Friend
4 Acquaintance
5 Other
8 Refuse to Answer

If Q473 is equal to 5 then skip to Q474

If not, then skip to instruction before Q475.

Q474. Please specify your relationship with [Response to Q408].

____

If Q470 is equal to 3 then skip to Q475

If not, then skip to instruction before Q476.

Q475. Is [Response to Q408]? (Choose one)

1 Neighbor
2 Housemate/Roommate
3 Other
8 Refuse to Answer

If Q475 is equal to 3 then skip to Q476

If not, then skip to instruction before Q477.

Q476. Please specify your relationship with [Response to Q408].

____

If Q470 is equal to 4 then skip to Q477

If not, then skip to instruction before Q478.

Q477. Is [Response to Q408]? (Choose one)

1 Co-worker

2 Boss

3 Other

8 Refuse to Answer

If Q477 is equal to 3 then skip to Q478

If not, then skip to instruction before Q479.

Q478. Please specify your relationship with [Response to Q408].

____

If Q470 is equal to 5 then skip to Q479

If not, then skip to instruction before Q480.

Q479. Is [Response to Q408]? (Choose one)

1 Parent [Mother/Father/Step-parent]

2 Child

3 Sibling [Brother/sister/step-brother/sister]

4 Brother-in-law or Sister-in-law

5 Other Relative

8 Refuse to Answer

If Q479 is equal to 5 then skip to Q480

If not, then skip to instruction before Q481.

Q480. Please specify your relationship with [Response to Q408].

____

If Q470 is equal to 6 then skip to Q481

If not, then skip to instruction before Q482.

Q481. Is [Response to Q408]? (Choose one)

1 Bartender

2 Drug Dealer

3 Someone who injects others for a fee or for drugs

4 Drug Selling Client (someone you sell drugs to)

5 Drinking Buddy

6 Someone you use drugs with

7 Other

8 Refuse to Answer

If Q481 is equal to 7 then skip to Q482

If not, then skip to instruction before Q483.

Q482. Please specify your relationship with [Response to Q408].

____

If Q470 is equal to 7 then skip to Q483

If not, then skip to instruction before Q484.

Q483. Is [Response to Q408]? (Choose one)

1 Religious or Spiritual Advisor

2 Teacher or Other Educator

3 Medical Professional

4 Mental Health Professional

5 Other

8 Refuse to Answer

If Q483 is equal to 5 then skip to Q484

If not, then skip to instruction before Q485.

Q484. Please specify your relationship with [Response to Q408]?

____

If Q470 is not equal to 5 and Q470 is not equal to 8 then skip to Q485

If not, then skip to Q486.

Q485. Did you have vaginal or anal sex with [Response to Q408] in the previous 6 months?

1 Yes

0 No

8 Refuse to Answer

Q486. Is [Response to Q408] male, female, transgender, non-binary or other? (Choose one)

1 Male

2 Female

3 Transgender woman (i.e., male to female)

4 Transgender man (i.e., female to male)

5 Non-binary / genderqueer / gender fluid / agender

6 Another identity not listed

8 Refuse to Answer

Q487. Please tell me how often you communicate with [Response to Q408] (in person, phone, email, and on social media, like Facebook, count) (Choose one)

01	Every Day
02	Several times a week
03	Once a week
04	Once every two weeks
05	Once a month
06	A couple of times a year
07	Once a year
08	Less than once a year
98	Refuse to Answer

Q488. How close do you feel to [Response to Q408]? (Choose one)

1	Very close
2	Somewhat close
3	Not very close
4	Not close at all
8	Refuse to Answer

Q489. How long have you felt this close to [Response to Q408]?

__ __	YEARS
__ __	MONTHS
__ __	DAYS
97	Don't Know (Years)
98	Refuse to Answer (Years)

If Q489 is greater than Q469 and Q469 is not equal to 97 and Q469 is not equal to 98 then READ: "That response is greater than the times you said you know &[NAME2]. Please check the response." and skip to Q489.

If Q405 is greater than 2 and Q410 is not equal to "Refuse to Answer" and Q410 is not equal to "skipped" then skip to Q490

If not, then skip to instruction before Q512.

Q490. What is [Response to Q410]'s age?

__ __	years old
97	Don't Know
98	Refuse to Answer

Q491. How long have you known [Response to Q410]?

__ __	YEARS
__ __	MONTHS
__ __	DAYS
97	Don't Know (Years)
98	Refuse to Answer (Years)

If Q491 is greater than Q490 then READ: "The participant said that they have known this person for longer than this person's age. You are redirected to make corrections." and skip to Q490.

Q492. What is your relationship to [Response to Q410]? (Choose one)

1	Romantic or sex partner
2	Friend
3	Neighboor or housemate
4	Co-worker or boss
5	Family - relative by blood or marriage
6	Drug or Alcohol Related
7	Other
8	Refuse to Answer

If Q492 is equal to 1 then skip to Q493

If not, then skip to instruction before Q494.

Q493. Is [Response to Q410]? (Choose one)

1 Spouse
2 Current romantic partner
3 Ex-romantic partner
4 Sex partner (non-romantic)
5 Other
8 Refuse to Answer

If Q493 is equal to 5 then skip to Q494

If not, then skip to instruction before Q495.

Q494. Please specify your relationship with [Response to Q410].

____

If Q492 is equal to 2 then skip to Q495

If not, then skip to instruction before Q496.

Q495. Is [Response to Q410]? (Choose one)

1 Best Friend
2 Close Friend
3 Friend
4 Acquaintance
5 Other
8 Refuse to Answer

If Q495 is equal to 5 then skip to Q496

If not, then skip to instruction before Q497.

Q496. Please specify your relationship with [Response to Q410].

____

If Q492 is equal to 3 then skip to Q497

If not, then skip to instruction before Q498.

Q497. Is [Response to Q410]? (Choose one)

1	Neighbor
2	Housemate/Roommate
3	Other
8	Refuse to Answer

If Q497 is equal to 3 then skip to Q498

If not, then skip to instruction before Q499.

Q498. Please specify your relationship with [Response to Q410].

____

If Q492 is equal to 4 then skip to Q499

If not, then skip to instruction before Q500.

Q499. Is [Response to Q410]? (Choose one)

1	Co-worker
2	Boss
3	Other
8	Refuse to Answer

If Q499 is equal to 3 then skip to Q500

If not, then skip to instruction before Q501.

Q500. Please specify your relationship with [Response to Q410].

____

If Q492 is equal to 5 then skip to Q501

If not, then skip to instruction before Q502.

Q501. Is [Response to Q410]? (Choose one)

1 Parent [Mother/Father/Step-parent]

2 Child

3 Sibling [Brother/sister/step-brother/sister]

4 Brother-in-law or Sister-in-law

5 Other Relative

8 Refuse to Answer

If Q501 is equal to 5 then skip to Q502

If not, then skip to instruction before Q503.

Q502. Please specify your relationship with [Response to Q410].

____

If Q492 is equal to 6 then skip to Q503

If not, then skip to instruction before Q504.

Q503. Is [Response to Q410]? (Choose one)

1 Bartender

2 Drug Dealer

3 Someone who injects others for a fee or for drugs

4 Drug Selling Client (someone you sell drugs to)

5 Drinking Buddy

6 Someone you use drugs with

7 Other

8 Refuse to Answer

If Q503 is equal to 7 then skip to Q504

If not, then skip to instruction before Q505.

Q504. Please specify your relationship with [Response to Q410].

____

If Q492 is equal to 7 then skip to Q505

If not, then skip to instruction before Q506.

Q505. Is [Response to Q410]? (Choose one)

1 Religious or Spiritual Advisor

2 Teacher or Other Educator

3 Medical Professional

4 Mental Health Professional

5 Other

8 Refuse to Answer

If Q505 is equal to 5 then skip to Q506

If not, then skip to instruction before Q507.

Q506. Please specify your relationship with [Response to Q410]?

____

If Q492 is not equal to 5 and Q492 is not equal to 8 then skip to Q507

If not, then skip to Q508.

Q507. Did you have vaginal or anal sex with [Response to Q410] in the previous 6 months?

1 Yes

0 No

8 Refuse to Answer

Q508. Is [Response to Q410] male, female, transgender, non-binary or other? (Choose one)

1 Male

2 Female

3 Transgender woman (i.e., male to female)

4 Transgender man (i.e., female to male)

5 Non-binary / genderqueer / gender fluid / agender

6 Another identity not listed

8 Refuse to Answer

Q509. Please tell me how often you communicate with [Response to Q410] (in person, phone, email, and on social media, like Facebook, count) (Choose one)

01 Every Day

02 Several times a week

03 Once a week

04 Once every two weeks

05 Once a month

06 A couple of times a year

07 Once a year

08 Less than once a year

98 Refuse to Answer

Q510. How close do you feel to [Response to Q410]? (Choose one)

1 Very close

2 Somewhat close

3 Not very close

4 Not close at all

8 Refuse to Answer

Q511. How long have you felt this close to [Response to Q410]?

__ __	YEARS
__ __	MONTHS
__ __	DAYS
97	Don't Know (Years)
98	Refuse to Answer (Years)

If Q511 is greater than Q491 and Q491 is not equal to 97 and Q491 is not equal to 98 then READ: "That response is greater than the times you said you know &[NAME3]. Please check the response." and skip to Q511.

If Q405 is greater than 3 and Q412 is not equal to "Refuse to Answer" and Q412 is not equal to "skipped" then skip to Q512

If not, then skip to instruction before Q534.

Q512. What is [Response to Q412]'s age?

__ __	years old
97	Don't Know
98	Refuse to Answer

Q513. How long have you known [Response to Q412]?

__ __	YEARS
__ __	MONTHS
__ __	DAYS
97	Don't Know (Years)
98	Refuse to Answer (Years)

If Q513 is greater than Q512 then READ: "The participant said that they have known this person for longer than this person's age. You are redirected to make corrections." and skip to Q512.

Q514. What is your relationship to [Response to Q412]? (Choose one)

1 Romantic or sex partner

2 Friend

3 Neighboor or housemate

4 Co-worker or boss

5 Family - relative by blood or marriage

6 Drug or Alcohol Related

7 Other

8 Refuse to Answer

If Q514 is equal to 1 then skip to Q515

If not, then skip to instruction before Q516.

Q515. Is [Response to Q412]? (Choose one)

1 Spouse

2 Current romantic partner

3 Ex-romantic partner

4 Sex partner (non-romantic)

5 Other

8 Refuse to Answer

If Q515 is equal to 5 then skip to Q516

If not, then skip to instruction before Q517.

Q516. Please specify your relationship with [Response to Q412].

____

If Q514 is equal to 2 then skip to Q517

If not, then skip to instruction before Q518.

Q517. Is [Response to Q412]? (Choose one)

1 Best Friend
2 Close Friend
3 Friend
4 Acquaintance
5 Other
8 Refuse to Answer

If Q517 is equal to 5 then skip to Q518

If not, then skip to instruction before Q519.

Q518. Please specify your relationship with [Response to Q412].

____

If Q514 is equal to 3 then skip to Q519

If not, then skip to instruction before Q520.

Q519. Is [Response to Q412]? (Choose one)

1 Neighbor
2 Housemate/Roommate
3 Other
8 Refuse to Answer

If Q519 is equal to 3 then skip to Q520

If not, then skip to instruction before Q521.

Q520. Please specify your relationship with [Response to Q412].

____

If Q514 is equal to 4 then skip to Q521

If not, then skip to instruction before Q522.

Q521. Is [Response to Q412]? (Choose one)

1 Co-worker

2 Boss

3 Other

8 Refuse to Answer

If Q521 is equal to 3 then skip to Q522

If not, then skip to instruction before Q523.

Q522. Please specify your relationship with [Response to Q412].

——————————————————————————————

If Q514 is equal to 5 then skip to Q523

If not, then skip to instruction before Q524.

Q523. Is [Response to Q412]? (Choose one)

1 Parent [Mother/Father/Step-parent]

2 Child

3 Sibling [Brother/sister/step-brother/sister]

4 Brother-in-law or Sister-in-law

5 Other Relative

8 Refuse to Answer

If Q523 is equal to 5 then skip to Q524

If not, then skip to instruction before Q525.

Q524. Please specify your relationship with [Response to Q412].

——————————————————————————————

If Q514 is equal to 6 then skip to Q525

If not, then skip to instruction before Q526.

Q525. Is [Response to Q412]? (Choose one)

1 Bartender
2 Drug Dealer
3 Someone who injects others for a fee or for drugs
4 Drug Selling Client (someone you sell drugs to)
5 Drinking Buddy
6 Someone you use drugs with
7 Other
8 Refuse to Answer

If Q525 is equal to 7 then skip to Q526

If not, then skip to instruction before Q527.

Q526. Please specify your relationship with [Response to Q412].

____

If Q514 is equal to 7 then skip to Q527

If not, then skip to instruction before Q528.

Q527. Is [Response to Q412]? (Choose one)

1 Religious or Spiritual Advisor
2 Teacher or Other Educator
3 Medical Professional
4 Mental Health Professional
5 Other
8 Refuse to Answer

If Q527 is equal to 5 then skip to Q528

If not, then skip to instruction before Q529.

Q528. Please specify your relationship with [Response to Q412]?

____

If Q514 is not equal to 5 and Q514 is not equal to 8 then skip to Q529

If not, then skip to Q530.

Q529. Did you have vaginal or anal sex with [Response to Q412] in the previous 6 months?

1 Yes

0 No

8 Refuse to Answer

Q530. Is [Response to Q412] male, female, transgender, non-binary or other? (Choose one)

1 Male

2 Female

3 Transgender woman (i.e., male to female)

4 Transgender man (i.e., female to male)

5 Non-binary / genderqueer / gender fluid / agender

6 Another identity not listed

8 Refuse to Answer

Q531. Please tell me how often you communicate with [Response to Q412] (in person, phone, email, and on social media, like Facebook, count) (Choose one)

01	Every Day
02	Several times a week
03	Once a week
04	Once every two weeks
05	Once a month
06	A couple of times a year
07	Once a year
08	Less than once a year
98	Refuse to Answer

Q532. How close do you feel to [Response to Q412]? (Choose one)

1	Very close
2	Somewhat close
3	Not very close
4	Not close at all
8	Refuse to Answer

Q533. How long have you felt this close to [Response to Q412]?

__ __	YEARS
__ __	MONTHS
__ __	DAYS
97	Don't Know (Years)
98	Refuse to Answer (Years)

If Q533 is greater than Q513 and Q513 is not equal to 97 and Q513 is not equal to 98 then READ: "That response is greater than the times you said you know &[NAME4]. Please check the response." and skip to Q533.

If Q405 is greater than 4 and Q414 is not equal to "Refuse to Answer" and Q414 is not equal to "skipped" then skip to Q534

If not, then skip to instruction before Q556.

Q534. What is [Response to Q414]'s age?

__ __	years old
97	Don't Know
98	Refuse to Answer

Q535. How long have you known [Response to Q414]?

__ __	YEARS
__ __	MONTHS
__ __	DAYS
97	Don't Know (Years)
98	Refuse to Answer (Years)

If Q535 is greater than Q534 then READ: "The participant said that they have known this person for longer than this person's age. You are redirected to make corrections." and skip to Q534.

Q536. What is your relationship to [Response to Q414]? (Choose one)

1	Romantic or sex partner
2	Friend
3	Neighboor or housemate
4	Co-worker or boss
5	Family - relative by blood or marriage
6	Drug or Alcohol Related
7	Other
8	Refuse to Answer

If Q536 is equal to 1 then skip to Q537

If not, then skip to instruction before Q538.

Q537. Is [Response to Q414]? (Choose one)

1 Spouse

2 Current romantic partner

3 Ex-romantic partner

4 Sex partner (non-romantic)

5 Other

8 Refuse to Answer

If Q537 is equal to 5 then skip to Q538

If not, then skip to instruction before Q539.

Q538. Please specify your relationship with [Response to Q414].

____

If Q536 is equal to 2 then skip to Q539

If not, then skip to instruction before Q540.

Q539. Is [Response to Q414]? (Choose one)

1 Best Friend

2 Close Friend

3 Friend

4 Acquaintance

5 Other

8 Refuse to Answer

If Q539 is equal to 5 then skip to Q540

If not, then skip to instruction before Q541.

Q540. Please specify your relationship with [Response to Q414].

____

If Q536 is equal to 3 then skip to Q541

If not, then skip to instruction before Q542.

Q541. Is [Response to Q414]? (Choose one)

1	Neighbor
2	Housemate/Roommate
3	Other
8	Refuse to Answer

If Q541 is equal to 3 then skip to Q542

If not, then skip to instruction before Q543.

Q542. Please specify your relationship with [Response to Q414].

____

If Q536 is equal to 4 then skip to Q543

If not, then skip to instruction before Q544.

Q543. Is [Response to Q414]? (Choose one)

1	Co-worker
2	Boss
3	Other
8	Refuse to Answer

If Q543 is equal to 3 then skip to Q544

If not, then skip to instruction before Q545.

Q544. Please specify your relationship with [Response to Q414].

____

If Q536 is equal to 5 then skip to Q545

If not, then skip to instruction before Q546.

Q545. Is [Response to Q414]? (Choose one)

1 Parent [Mother/Father/Step-parent]

2 Child

3 Sibling [Brother/sister/step-brother/sister]

4 Brother-in-law or Sister-in-law

5 Other Relative

8 Refuse to Answer

If Q545 is equal to 5 then skip to Q546

If not, then skip to instruction before Q547.

Q546. Please specify your relationship with [Response to Q414].

____

If Q536 is equal to 6 then skip to Q547

If not, then skip to instruction before Q548.

Q547. Is [Response to Q414]? (Choose one)

1 Bartender

2 Drug Dealer

3 Someone who injects others for a fee or for drugs

4 Drug Selling Client (someone you sell drugs to)

5 Drinking Buddy

6 Someone you use drugs with

7 Other

8 Refuse to Answer

If Q547 is equal to 7 then skip to Q548

If not, then skip to instruction before Q549.

Q548. Please specify your relationship with [Response to Q414].

____

If Q536 is equal to 7 then skip to Q549

If not, then skip to instruction before Q550.

Q549. Is [Response to Q414]? (Choose one)

1 Religious or Spiritual Advisor

2 Teacher or Other Educator

3 Medical Professional

4 Mental Health Professional

5 Other

8 Refuse to Answer

If Q549 is equal to 5 then skip to Q550

If not, then skip to instruction before Q551.

Q550. Please specify your relationship with [Response to Q414]?

____

If Q536 is not equal to 5 and Q536 is not equal to 8 then skip to Q551

If not, then skip to Q552.

Q551. Did you have vaginal or anal sex with [Response to Q414] in the previous 6 months?

1 Yes

0 No

8 Refuse to Answer

Q552. Is [Response to Q414] male, female, transgender, non-binary or other? (Choose one)

1 Male

2 Female

3 Transgender woman (i.e., male to female)

4 Transgender man (i.e., female to male)

5 Non-binary / genderqueer / gender fluid / agender

6 Another identity not listed

8 Refuse to Answer

Q553. Please tell me how often you communicate with [Response to Q414] (in person, phone, email, and on social media, like Facebook, count) (Choose one)

01 Every Day

02 Several times a week

03 Once a week

04 Once every two weeks

05 Once a month

06 A couple of times a year

07 Once a year

08 Less than once a year

98 Refuse to Answer

Q554. How close do you feel to [Response to Q414]? (Choose one)

1 Very close

2 Somewhat close

3 Not very close

4 Not close at all

8 Refuse to Answer

Q555. How long have you felt this close to [Response to Q414]?

__ __	YEARS
__ __	MONTHS
__ __	DAYS
97	Don't Know (Years)
98	Refuse to Answer (Years)

If Q555 is greater than Q535 and Q535 is not equal to 97 and Q535 is not equal to 98 then READ: "That response is greater than the times you said you know &[NAME5]. Please check the response." and skip to Q555.

If Q405 is greater than 1 and Q406 is not equal to "Refuse to Answer" and Q406 is not equal to "skipped" and Q408 is not equal to "Refuse to Answer" and Q408 is not equal to "skipped" then skip to instruction before Q556

If not, then skip to instruction before Q557.

READ: In the next set of questions, I'm going to give you two of the names you listed earlier, and ask you to indicate how frequently these two people talk to each other. Once we get started, I think you'll see that this works pretty easily.

Q556. How frequently do [Response to Q406] and [Response to Q408] communicate with each other, that could be by talking, texting, or chatting? (Choose one)

01	Several times a day
02	Every day
03	Several times a week
04	Once a week
05	Once every two weeks
06	Once a month
07	A couple of times a year
08	Once a year
09	Never
97	Don't Know
98	Refuse to Answer

If Q405 is greater than 2 and Q406 is not equal to "Refuse to Answer" and Q406 is not equal to "skipped" and Q410 is not equal to "Refuse to Answer" and Q410 is not equal to "skipped" then skip to Q557

If not, then skip to instruction before Q558.

Q557. How frequently do [Response to Q406] and [Response to Q410] communicate with each other, that could be by talking, texting, or chatting? (Choose one)

01	Several times a day
02	Every day
03	Several times a week
04	Once a week
05	Once every two weeks
06	Once a month
07	A couple of times a year
08	Once a year
09	Never
97	Don't Know
98	Refuse to Answer

If Q405 is greater than 3 and Q406 is not equal to "Refuse to Answer" and Q406 is not equal to "skipped" and Q412 is not equal to "Refuse to Answer" and Q412 is not equal to "skipped" then skip to Q558

If not, then skip to instruction before Q559.

Q558. How frequently do [Response to Q406] and [Response to Q412] communicate with each other, that could be by talking, texting, or chatting? (Choose one)

01	Several times a day
02	Every day
03	Several times a week
04	Once a week
05	Once every two weeks
06	Once a month
07	A couple of times a year
08	Once a year
09	Never
97	Don't Know
98	Refuse to Answer

If Q405 is greater than 4 and Q406 is not equal to "Refuse to Answer" and Q406 is not equal to "skipped" and Q414 is not equal to "Refuse to Answer" and Q414 is not equal to "skipped" then skip to Q559

If not, then skip to instruction before Q560.

Q559. How frequently do [Response to Q406] and [Response to Q414] communicate with each other, that could be by talking, texting, or chatting? (Choose one)

01	Several times a day
02	Every day
03	Several times a week
04	Once a week
05	Once every two weeks
06	Once a month
07	A couple of times a year
08	Once a year
09	Never
97	Don't Know
98	Refuse to Answer

If Q405 is greater than 2 and Q408 is not equal to "Refuse to Answer" and Q408 is not equal to "skipped" and Q410 is not equal to "Refuse to Answer" and Q410 is not equal to "skipped" then skip to Q560

If not, then skip to instruction before Q561.

Q560. How frequently do [Response to Q408] and [Response to Q410] communicate with each other, that could be by talking, texting, or chatting? (Choose one)

01	Several times a day
02	Every day
03	Several times a week
04	Once a week
05	Once every two weeks
06	Once a month
07	A couple of times a year
08	Once a year
09	Never
97	Don't Know
98	Refuse to Answer

If Q405 is greater than 3 and Q408 is not equal to "Refuse to Answer" and Q408 is not equal to "skipped" and Q412 is not equal to "Refuse to Answer" and Q412 is not equal to "skipped" then skip to Q561

If not, then skip to instruction before Q562.

Q561. How frequently do [Response to Q408] and [Response to Q412] communicate with each other, that could be by talking, texting, or chatting? (Choose one)

01 Several times a day

02 Every day

03 Several times a week

04 Once a week

05 Once every two weeks

06 Once a month

07 A couple of times a year

08 Once a year

09 Never

97 Don't Know

98 Refuse to Answer

If Q405 is greater than 4 and Q408 is not equal to "Refuse to Answer" and Q408 is not equal to "skipped" and Q414 is not equal to "Refuse to Answer" and Q414 is not equal to "skipped" then skip to Q562

If not, then skip to instruction before Q563.

Q562. How frequently do [Response to Q408] and [Response to Q414] communicate with each other, that could be by talking, texting, or chatting? (Choose one)

01	Several times a day
02	Every day
03	Several times a week
04	Once a week
05	Once every two weeks
06	Once a month
07	A couple of times a year
08	Once a year
09	Never
97	Don't Know
98	Refuse to Answer

If Q405 is greater than 3 and Q410 is not equal to "Refuse to Answer" and Q410 is not equal to "skipped" and Q412 is not equal to "Refuse to Answer" and Q412 is not equal to "skipped" then skip to Q563

If not, then skip to instruction before Q564.

Q563. How frequently do [Response to Q410] and [Response to Q412] communicate with each other, that could be by talking, texting, or chatting? (Choose one)

01	Several times a day
02	Every day
03	Several times a week
04	Once a week
05	Once every two weeks
06	Once a month
07	A couple of times a year
08	Once a year
09	Never
97	Don't Know
98	Refuse to Answer

If Q405 is greater than 4 and Q410 is not equal to "Refuse to Answer" and Q410 is not equal to "skipped" and Q414 is not equal to "Refuse to Answer" and Q414 is not equal to "skipped" then skip to Q564

If not, then skip to instruction before Q565.

Q564. How frequently do [Response to Q410] and [Response to Q414] communicate with each other, that could be by talking, texting, or chatting? (Choose one)

01	Several times a day
02	Every day
03	Several times a week
04	Once a week
05	Once every two weeks
06	Once a month
07	A couple of times a year
08	Once a year
09	Never
97	Don't Know
98	Refuse to Answer

If Q405 is greater than 4 and Q412 is not equal to "Refuse to Answer" and Q412 is not equal to "skipped" and Q414 is not equal to "Refuse to Answer" and Q414 is not equal to "skipped" then skip to Q565

If not, then skip to instruction before Q566.

Q565. How frequently do [Response to Q412] and [Response to Q414] communicate with each other, that could be by talking, texting, or chatting? (Choose one)

01	Several times a day
02	Every day
03	Several times a week
04	Once a week
05	Once every two weeks
06	Once a month
07	A couple of times a year
08	Once a year
09	Never
97	Don't Know
98	Refuse to Answer

If Q405 is greater than 0 and Q406 is not equal to "Refuse to Answer" and Q406 is not equal to "skipped" then skip to Q566

If not, then skip to instruction before Q590.

Q566. How often does [Response to Q406] use drugs? (Choose one)

01	Every day
02	Several times a week
03	Once a week
04	Once every two weeks
05	Once a month
06	A couple of times a year
07	Once a year
08	Less than once a year
09	Never
10	Used to use drugs, but does not at the present time
97	Don't Know
98	Refuse to Answer

If Q566 is not less than 1 and Q566 is not greater than 8 then skip to Q567

If not, then skip to instruction before Q568.

Q567. Which of these does [Response to Q406] use? (Check all that apply) (Check all that apply)

__ Alcohol

__ Marijuana

__ Heroin by itself

__ Crack cocaine by itself

__ Cocaine powder by itself

__ Heroin and cocaine together (speedball)

__ Methamphetamine/crystal by itself

__ Methamphetamine/crystal and heroin together

__ China White by itself

__ China White and heroin together

__ China White and crystal/meth together

__ PCP

__ Hallucinogenic drugs like acid, LSD, peyote, mescaline, mushrooms

__ Inhalants (for example, glue, gasoline, paint thinner, agua celeste or other chemicals)

__ Tranquilizers without a prescription (Rivotril, Diazepam, Valium, Ativan or Restoril)

__ OxyContin without a prescription

__ Other prescription opioids without a prescription (Vicodin, Darvon, Percocet)

__ Barbiturates without a prescription (Amytal, Nembutal, and Seconal)

__ Another drug or combination that was not already mentioned

__ Don't Know

__ Refuse to Answer

If Q567S is equal to 1 then skip to Q568

If not, then skip to instruction before Q569.

Q568. Please specify what other drugs or combination of drugs [Response to Q406] uses.

____

If Q566 is not less than 1 and Q566 is not greater than 8 then skip to Q569

If not, then skip to instruction before Q570.

Q569. Does [Response to Q406] use drugs by injection, by noninjection, or sometimes one and sometimes the other? (Choose one)

1 Only uses drugs by injection

2 Only uses drugs by non-injection

3 Sometimes uses drugs by injection and sometimes by non-injection

7 Don't Know

8 Refuse to Answer

If Q569 is equal to 1 or Q569 is equal to 3 then skip to Q570

If not, then skip to instruction before Q571.

Q570. Have you ever used a needle, water, cooker or cotton that had already been used by [Response to Q406]?

1 Yes

0 No

8 Refuse to Answer

If Q566 is not equal to 9 and Q566 is not equal to 97 and Q566 is not equal to 98 then skip to Q571

If not, then skip to instruction before Q572.

Q571. Has [Response to Q406] ever offered to share or encouraged you to use drugs?

1	Yes
0	No
8	Refuse to Answer

If Q571 is equal to 1 then skip to Q572

If not, then skip to instruction before Q573.

Q572. Which of these has [Response to Q406] offered to share or encouraged you to use? (Check all that apply) (Check all that apply)

__ Alcohol

__ Marijuana

__ Heroin by itself

__ Crack cocaine by itself

__ Cocaine powder by itself

__ Heroin and cocaine together (speedball)

__ Methamphetamine/crystal by itself

__ Methamphetamine/crystal and heroin together

__ China White by itself

__ China White and heroin together

__ China White and crystal/meth together

__ PCP

__ Hallucinogenic drugs like acid, LSD, peyote, mescaline, mushrooms

__ Inhalants (for example, glue, gasoline, paint thinner, agua celeste or other chemicals)

__ Tranquilizers without a prescription (Rivotril, Diazepam, Valium, Ativan or Restoril)

__ OxyContin without a prescription

__ Other prescription opioids without a prescription (Vicodin, Darvon, Percocet)

__ Barbiturates without a prescription (Amytal, Nembutal, and Seconal)

__ Another drug or combination that was not already mentioned

__ Refuse to Answer

If Q572S is equal to 1 then skip to Q573

If not, then skip to Q574.

Q573. Please specify which of drugs has [Response to Q406] offered to share or encouraged you to use?

____

Q574. Has [Response to Q406] ever encouraged you to stop using drugs?

1 Yes

0 No

8 Refuse to Answer

If Q574 is equal to 1 then skip to Q575

If not, then skip to instruction before Q576.

Q575. Has [Response to Q406] ever encouraged you to specifically stop using drugs by injection?

1 Yes

0 No

7 Don't Know

8 Refuse to Answer

If Q566 is not equal to 9 and Q566 is not equal to 97 and Q566 is not equal to 98 then skip to Q576

If not, then skip to instruction before Q577.

Q576. How old was [Response to Q406] when they first started using drugs?

__ __ years old

97 Don't Know

98 Refuse to Answer

If Q566 is not equal to 9 and Q566 is not equal to 10 and Q566 is not equal to 97 and Q566 is not equal to 98 then skip to Q577

If not, then skip to Q578.

Q577. Consider [Response to Q406] usual daily drug use, does [Response to Q406] ever use double the amount or mix with other drugs?

1 Yes

0 No

7 Don't Know

8 Refuse to Answer

Q578. Has [Response to Q406] ever lived on the street, in a shelter, in a Single Room Occupancy hotel (SRO), temporarily with friends/relatives, in a car, or squatted?

1 Yes

0 No

7 Don't Know

8 Refuse to Answer

Q579. Where does [Response to Q406] currently live? (Choose one)

1 San Diego County

2 Tijuana

3 Other

7 Don't Know

8 Refuse to Answer

If Q579 is equal to 3 then skip to Q580

If not, then skip to instruction before Q581.

Q580. Please specify where [Response to Q406] lives.

____

If Q579 is equal to 1 and Q566 is not equal to 9 and Q566 is not equal to 97 and Q566 is not equal to 98 then skip to Q581

If not, then skip to instruction before Q582.

Q581. **In the past 6 months**, had [Response to Q406] traveled to Mexico to buy or obtain drugs?

1	Yes
0	No
7	Don't Know
8	Refuse to Answer

If Q581 is equal to 1 then skip to Q582

If not, then skip to instruction before Q583.

Q582. Which types of drugs did [Response to Q406] buy in Mexico? (Check all that apply) (Check all that apply)

__ Marijuana

__ Heroin

__ Crack cocaine by itself

__ Cocaine (powder) by itself

__ Heroin and cocaine together (speedball)

__ Methamphetamine/crystal by itself

__ Methamphetamine/crystal and heroin together

__ China White by itself

__ China White and heroin together

__ China White and crystal/meth together

__ PCP

__ Hallucinogenic drugs like acid, LSD, peyote, mescaline, mushrooms

__ Inhalants (for example, glue, gasoline, paint thinner, agua celeste or other chemicals)

__ Tranquilizers without a prescription (Rivotril, Diazepam, Valium, Ativan or Restoril)

__ OxyContin without a prescription

__ Other prescription opioids without a prescription (Vicodin, Darvon, Percocet)

__ Barbiturates without a prescription (Amytal, Nembutal, and Seconal)

__ Another drug or combination that was not already mentioned

__ Don't Know

__ Refuse to Answer

If Q582R is equal to 1 then skip to Q583

If not, then skip to instruction before Q584.

Q583. Please specify which types of drugs did [Response to Q406] buy in Mexico?

____

If Q579 is equal to 1 then skip to Q584

If not, then skip to instruction before Q585.

Q584. **In the past 6 months**, had [Response to Q406] traveled to Mexico to pay money or drugs for sex?

1	Yes
0	No
7	Don't Know
8	Refuse to Answer

If Q579 is equal to 2 and Q566 is not equal to 9 and Q566 is not equal to 97 and Q566 is not equal to 98 then skip to Q585

If not, then skip to instruction before Q586.

Q585. **In the past 6 months**, had [Response to Q406] traveled to the United states to buy or obtain drugs?

1	Yes
0	No
7	Don't Know
8	Refuse to Answer

If Q585 is equal to 1 then skip to Q586

If not, then skip to instruction before Q587.

Q586. Which types of drugs did [Response to Q406] buy in the US? (Check all that apply) (Check all that apply)

__ Marijuana

__ Heroin

__ Crack cocaine by itself

__ Cocaine (powder) by itself

__ Heroin and cocaine together (speedball)

__ Methamphetamine/crystal by itself

__ Methamphetamine/crystal and heroin together

__ China White by itself

__ China White and heroin together

__ China White and crystal/meth together

__ PCP

__ Hallucinogenic drugs like acid, LSD, peyote, mescaline, mushrooms

__ Inhalants (for example, glue, gasoline, paint thinner, agua celeste or other chemicals)

__ Tranquilizers without a prescription (Rivotril, Diazepam, Valium, Ativan or Restoril)

__ OxyContin without a prescription

__ Other prescription opioids without a prescription (Vicodin, Darvon, Percocet)

__ Barbiturates without a prescription (Amytal, Nembutal, and Seconal)

__ Another drug or combination that was not already mentioned

__ Don't Know

__ Refuse to Answer

If Q586R is equal to 1 then skip to Q587

If not, then skip to instruction before Q588.

Q587. Please specify which types of drugs did [Response to Q406] buy in the US?

____

If Q579 is equal to 2 then skip to Q588

If not, then skip to Q589.

Q588. **In the past 6 months**, has [Response to Q406] had sex for money or drugs with someone from the United States?

1 Yes

0 No

7 Don't Know

8 Refuse to Answer

Q589. Has [Response to Q406] ever been detained or arrested?

1 Yes

0 No

7 Don't Know

8 Refuse to Answer

If Q405 is greater than 1 and Q408 is not equal to "Refuse to Answer" and Q408 is not equal to "skipped" then skip to Q590

If not, then skip to instruction before Q614.

Q590. How often does [Response to Q408] use drugs? (Choose one)

01	Every day
02	Several times a week
03	Once a week
04	Once every two weeks
05	Once a month
06	A couple of times a year
07	Once a year
08	Less than once a year
09	Never
10	Used to use drugs, but does not at the present time
97	Don't Know
98	Refuse to Answer

If Q590 is not less than 1 and Q590 is not greater than 8 then skip to Q591

If not, then skip to instruction before Q592.

Q591. Which of these does [Response to Q408] use? (Check all that apply) (Check all that apply)

__ Alcohol

__ Marijuana

__ Heroin by itself

__ Crack cocaine by itself

__ Cocaine powder by itself

__ Heroin and cocaine together (speedball)

__ Methamphetamine/crystal by itself

__ Methamphetamine/crystal and heroin together

__ China White by itself

__ China White and heroin together

__ China White and crystal/meth together

__ PCP

__ Hallucinogenic drugs like acid, LSD, peyote, mescaline, mushrooms

__ Inhalants (for example, glue, gasoline, paint thinner, agua celeste or other chemicals)

__ Tranquilizers without a prescription (Rivotril, Diazepam, Valium, Ativan or Restoril)

__ OxyContin without a prescription

__ Other prescription opioids without a prescription (Vicodin, Darvon, Percocet)

__ Barbiturates without a prescription (Amytal, Nembutal, and Seconal)

__ Another drug or combination that was not already mentioned

__ Don't Know

__ Refuse to Answer

If Q591S is equal to 1 then skip to Q592

If not, then skip to instruction before Q593.

Q592. Please specify what other drugs or combination of drugs [Response to Q408] uses.

____

If Q590 is not less than 1 and Q590 is not greater than 8 then skip to Q593

If not, then skip to instruction before Q594.

Q593. Does [Response to Q408] use drugs by injection, by noninjection, or sometimes one and sometimes the other? (Choose one)

1 Only uses drugs by injection

2 Only uses drugs by non-injection

3 Sometimes uses drugs by injection and sometimes by non-injection

7 Don't Know

8 Refuse to Answer

If Q593 is equal to 1 or Q593 is equal to 3 then skip to Q594

If not, then skip to instruction before Q595.

Q594. Have you ever used a needle, water, cooker or cotton that had already been used by [Response to Q408]?

1 Yes

0 No

8 Refuse to Answer

If Q590 is not equal to 9 and Q590 is not equal to 97 and Q590 is not equal to 98 then skip to Q595

If not, then skip to instruction before Q596.

Q595. Has [Response to Q408] ever offered to share or encouraged you to use drugs?

1	Yes
0	No
8	Refuse to Answer

If Q595 is equal to 1 then skip to Q596

If not, then skip to instruction before Q597.

Q596. Which of these has [Response to Q408] offered to share or encouraged you to use? (Check all that apply) (Check all that apply)

__ Alcohol

__ Marijuana

__ Heroin by itself

__ Crack cocaine by itself

__ Cocaine powder by itself

__ Heroin and cocaine together (speedball)

__ Methamphetamine/crystal by itself

__ Methamphetamine/crystal and heroin together

__ China White by itself

__ China White and heroin together

__ China White and crystal/meth together

__ PCP

__ Hallucinogenic drugs like acid, LSD, peyote, mescaline, mushrooms

__ Inhalants (for example, glue, gasoline, paint thinner, agua celeste or other chemicals)

__ Tranquilizers without a prescription (Rivotril, Diazepam, Valium, Ativan or Restoril)

__ OxyContin without a prescription

__ Other prescription opioids without a prescription (Vicodin, Darvon, Percocet)

__ Barbiturates without a prescription (Amytal, Nembutal, and Seconal)

__ Another drug or combination that was not already mentioned

__ Refuse to Answer

If Q596S is equal to 1 then skip to Q597

If not, then skip to Q598.

Q597. Please specify which of drugs has [Response to Q408] offered to share or encouraged you to use?

____

Q598. Has [Response to Q408] ever encouraged you to stop using drugs?

1 Yes

0 No

8 Refuse to Answer

If Q598 is equal to 1 then skip to Q599

If not, then skip to instruction before Q600.

Q599. Has [Response to Q408] ever encouraged you to specifically stop using drugs by injection?

1 Yes

0 No

7 Don't Know

8 Refuse to Answer

If Q590 is not equal to 9 and Q590 is not equal to 97 and Q590 is not equal to 98 then skip to Q600

If not, then skip to instruction before Q601.

Q600. How old was [Response to Q408] when they first started using drugs?

__ __ years old

97 Don't Know

98 Refuse to Answer

If Q590 is not equal to 9 and Q590 is not equal to 10 and Q590 is not equal to 97 and Q590 is not equal to 98 then skip to Q601

If not, then skip to Q602.

Q601. Consider [Response to Q408] usual daily drug use, does [Response to Q408] ever use double the amount or mix with other drugs?

1 Yes

0 No

7 Don't Know

8 Refuse to Answer

Q602. Has [Response to Q408] ever lived on the street, in a shelter, in a Single Room Occupancy hotel (SRO), temporarily with friends/relatives, in a car, or squatted?

1 Yes

0 No

7 Don't Know

8 Refuse to Answer

Q603. Where does [Response to Q408] currently live? (Choose one)

1 San Diego County

2 Tijuana

3 Other

7 Don't Know

8 Refuse to Answer

If Q603 is equal to 3 then skip to Q604

If not, then skip to instruction before Q605.

Q604. Please specify where [Response to Q408] lives.

____

If Q603 is equal to 1 and Q590 is not equal to 9 and Q590 is not equal to 97 and Q590 is not equal to 98 then skip to Q605

If not, then skip to instruction before Q606.

Q605. **In the past 6 months**, had [Response to Q408] traveled to Mexico to buy or obtain drugs?

1	Yes
0	No
7	Don't Know
8	Refuse to Answer

If Q605 is equal to 1 then skip to Q606

If not, then skip to instruction before Q607.

Q606. Which types of drugs did [Response to Q408] buy in Mexico? (Check all that apply) (Check all that apply)

__ Marijuana

__ Heroin

__ Crack cocaine by itself

__ Cocaine (powder) by itself

__ Heroin and cocaine together (speedball)

__ Methamphetamine/crystal by itself

__ Methamphetamine/crystal and heroin together

__ China White by itself

__ China White and heroin together

__ China White and crystal/meth together

__ PCP

__ Hallucinogenic drugs like acid, LSD, peyote, mescaline, mushrooms

__ Inhalants (for example, glue, gasoline, paint thinner, agua celeste or other chemicals)

__ Tranquilizers without a prescription (Rivotril, Diazepam, Valium, Ativan or Restoril)

__ OxyContin without a prescription

__ Other prescription opioids without a prescription (Vicodin, Darvon, Percocet)

__ Barbiturates without a prescription (Amytal, Nembutal, and Seconal)

__ Another drug or combination that was not already mentioned

__ Don't Know

__ Refuse to Answer

If Q606R is equal to 1 then skip to Q607

If not, then skip to instruction before Q608.

Q607. Please specify which types of drugs did [Response to Q408] buy in Mexico?

____

If Q603 is equal to 1 then skip to Q608

If not, then skip to instruction before Q609.

Q608. **In the past 6 months**, had [Response to Q408] traveled to Mexico to pay money or drugs for sex?

1 Yes

0 No

7 Don't Know

8 Refuse to Answer

If Q603 is equal to 2 and Q590 is not equal to 9 and Q590 is not equal to 97 and Q590 is not equal to 98 then skip to Q609

If not, then skip to instruction before Q610.

Q609. **In the past 6 months**, had [Response to Q408] traveled to the United states to buy or obtain drugs?

1 Yes

0 No

7 Don't Know

8 Refuse to Answer

If Q609 is equal to 1 then skip to Q610

If not, then skip to instruction before Q611.

Q610. Which types of drugs did [Response to Q408] buy in the US? (Check all that apply) (Check all that apply)

__ Marijuana

__ Heroin

__ Crack cocaine by itself

__ Cocaine (powder) by itself

__ Heroin and cocaine together (speedball)

__ Methamphetamine/crystal by itself

__ Methamphetamine/crystal and heroin together

__ China White by itself

__ China White and heroin together

__ China White and crystal/meth together

__ PCP

__ Hallucinogenic drugs like acid, LSD, peyote, mescaline, mushrooms

__ Inhalants (for example, glue, gasoline, paint thinner, agua celeste or other chemicals)

__ Tranquilizers without a prescription (Rivotril, Diazepam, Valium, Ativan or Restoril)

__ OxyContin without a prescription

__ Other prescription opioids without a prescription (Vicodin, Darvon, Percocet)

__ Barbiturates without a prescription (Amytal, Nembutal, and Seconal)

__ Another drug or combination that was not already mentioned

__ Don't Know

__ Refuse to Answer

If Q610R is equal to 1 then skip to Q611

If not, then skip to instruction before Q612.

Q611. Please specify which types of drugs did [Response to Q408] buy in the US?

____

If Q603 is equal to 2 then skip to Q612

If not, then skip to Q613.

Q612. **In the past 6 months**, has [Response to Q408] had sex for money or drugs with someone from the United States?

1	Yes
0	No
7	Don't Know
8	Refuse to Answer

Q613. Has [Response to Q408] ever been detained or arrested?

1	Yes
0	No
7	Don't Know
8	Refuse to Answer

If Q405 is greater than 2 and Q410 is not equal to "Refuse to Answer" and Q410 is not equal to "skipped" then skip to Q614

If not, then skip to instruction before Q638.

Q614. How often does [Response to Q410] use drugs? (Choose one)

01	Every day
02	Several times a week
03	Once a week
04	Once every two weeks
05	Once a month
06	A couple of times a year
07	Once a year
08	Less than once a year
09	Never
10	Used to use drugs, but does not at the present time
97	Don't Know
98	Refuse to Answer

If Q614 is not less than 1 and Q614 is not greater than 8 then skip to Q615

If not, then skip to instruction before Q616.

Q615. Which of these does [Response to Q410] use? (Check all that apply) (Check all that apply)

__ Alcohol

__ Marijuana

__ Heroin by itself

__ Crack cocaine by itself

__ Cocaine powder by itself

__ Heroin and cocaine together (speedball)

__ Methamphetamine/crystal by itself

__ Methamphetamine/crystal and heroin together

__ China White by itself

__ China White and heroin together

__ China White and crystal/meth together

__ PCP

__ Hallucinogenic drugs like acid, LSD, peyote, mescaline, mushrooms

__ Inhalants (for example, glue, gasoline, paint thinner, agua celeste or other chemicals)

__ Tranquilizers without a prescription (Rivotril, Diazepam, Valium, Ativan or Restoril)

__ OxyContin without a prescription

__ Other prescription opioids without a prescription (Vicodin, Darvon, Percocet)

__ Barbiturates without a prescription (Amytal, Nembutal, and Seconal)

__ Another drug or combination that was not already mentioned

__ Don't Know

__ Refuse to Answer

If Q615S is equal to 1 then skip to Q616

If not, then skip to instruction before Q617.

Q616. Please specify what other drugs or combination of drugs [Response to Q410] uses.

____

If Q614 is not less than 1 and Q614 is not greater than 8 then skip to Q617

If not, then skip to instruction before Q618.

Q617. Does [Response to Q410] use drugs by injection, by noninjection, or sometimes one and sometimes the other? (Choose one)

1 Only uses drugs by injection

2 Only uses drugs by non-injection

3 Sometimes uses drugs by injection and sometimes by non-injection

7 Don't Know

8 Refuse to Answer

If Q617 is equal to 1 or Q617 is equal to 3 then skip to Q618

If not, then skip to instruction before Q619.

Q618. Have you ever used a needle, water, cooker or cotton that had already been used by [Response to Q410]?

1 Yes

0 No

8 Refuse to Answer

If Q614 is not equal to 9 and Q614 is not equal to 97 and Q614 is not equal to 98 then skip to Q619

If not, then skip to instruction before Q620.

Q619. Has [Response to Q410] ever offered to share or encouraged you to use drugs?

1	Yes
0	No
8	Refuse to Answer

If Q619 is equal to 1 then skip to Q620

If not, then skip to instruction before Q621.

Q620. Which of these has [Response to Q410] offered to share or encouraged you to use? (Check all that apply) (Check all that apply)

__ Alcohol

__ Marijuana

__ Heroin by itself

__ Crack cocaine by itself

__ Cocaine powder by itself

__ Heroin and cocaine together (speedball)

__ Methamphetamine/crystal by itself

__ Methamphetamine/crystal and heroin together

__ China White by itself

__ China White and heroin together

__ China White and crystal/meth together

__ PCP

__ Hallucinogenic drugs like acid, LSD, peyote, mescaline, mushrooms

__ Inhalants (for example, glue, gasoline, paint thinner, agua celeste or other chemicals)

__ Tranquilizers without a prescription (Rivotril, Diazepam, Valium, Ativan or Restoril)

__ OxyContin without a prescription

__ Other prescription opioids without a prescription (Vicodin, Darvon, Percocet)

__ Barbiturates without a prescription (Amytal, Nembutal, and Seconal)

__ Another drug or combination that was not already mentioned

__ Refuse to Answer

If Q620S is equal to 1 then skip to Q621

If not, then skip to Q622.

Q621. Please specify which of drugs has [Response to Q410] offered to share or encouraged you to use?

____

Q622. Has [Response to Q410] ever encouraged you to stop using drugs?

1 Yes

0 No

8 Refuse to Answer

If Q622 is equal to 1 then skip to Q623

If not, then skip to instruction before Q624.

Q623. Has [Response to Q410] ever encouraged you to specifically stop using drugs by injection?

1 Yes

0 No

7 Don't Know

8 Refuse to Answer

If Q614 is not equal to 9 and Q614 is not equal to 97 and Q614 is not equal to 98 then skip to Q624

If not, then skip to instruction before Q625.

Q624. How old was [Response to Q410] when they first started using drugs?

__ __ years old

97 Don't Know

98 Refuse to Answer

If Q614 is not equal to 9 and Q614 is not equal to 10 and Q614 is not equal to 97 and Q614 is not equal to 98 then skip to Q625

If not, then skip to Q626.

Q625. Consider [Response to Q410] usual daily drug use, does [Response to Q410] ever use double the amount or mix with other drugs?

1	Yes
0	No
7	Don't Know
8	Refuse to Answer

Q626. Has [Response to Q410] ever lived on the street, in a shelter, in a Single Room Occupancy hotel (SRO), temporarily with friends/relatives, in a car, or squatted?

1	Yes
0	No
7	Don't Know
8	Refuse to Answer

Q627. Where does [Response to Q410] currently live? (Choose one)

1	San Diego County
2	Tijuana
3	Other
7	Don't Know
8	Refuse to Answer

If Q627 is equal to 3 then skip to Q628

If not, then skip to instruction before Q629.

Q628. Please specify where [Response to Q410] lives.

____

If Q627 is equal to 1 and Q614 is not equal to 9 and Q614 is not equal to 97 and Q614 is not equal to 98 then skip to Q629

If not, then skip to instruction before Q630.

Q629. **In the past 6 months**, had [Response to Q410] traveled to Mexico to buy or obtain drugs?

1	Yes
0	No
7	Don't Know
8	Refuse to Answer

If Q629 is equal to 1 then skip to Q630

If not, then skip to instruction before Q631.

Q630. Which types of drugs did [Response to Q410] buy in Mexico? (Check all that apply) (Check all that apply)

__ Marijuana

__ Heroin

__ Crack cocaine by itself

__ Cocaine (powder) by itself

__ Heroin and cocaine together (speedball)

__ Methamphetamine/crystal by itself

__ Methamphetamine/crystal and heroin together

__ China White by itself

__ China White and heroin together

__ China White and crystal/meth together

__ PCP

__ Hallucinogenic drugs like acid, LSD, peyote, mescaline, mushrooms

__ Inhalants (for example, glue, gasoline, paint thinner, agua celeste or other chemicals)

__ Tranquilizers without a prescription (Rivotril, Diazepam, Valium, Ativan or Restoril)

__ OxyContin without a prescription

__ Other prescription opioids without a prescription (Vicodin, Darvon, Percocet)

__ Barbiturates without a prescription (Amytal, Nembutal, and Seconal)

__ Another drug or combination that was not already mentioned

__ Don't Know

__ Refuse to Answer

If Q630R is equal to 1 then skip to Q631

If not, then skip to instruction before Q632.

Q631. Please specify which types of drugs did [Response to Q410] buy in Mexico?

____

If Q627 is equal to 1 then skip to Q632

If not, then skip to instruction before Q633.

Q632. **In the past 6 months**, had [Response to Q410] traveled to Mexico to pay money or drugs for sex?

1 Yes

0 No

7 Don't Know

8 Refuse to Answer

If Q627 is equal to 2 and Q614 is not equal to 9 and Q614 is not equal to 97 and Q614 is not equal to 98 then skip to Q633

If not, then skip to instruction before Q634.

Q633. **In the past 6 months**, had [Response to Q410] traveled to the United states to buy or obtain drugs?

1 Yes

0 No

7 Don't Know

8 Refuse to Answer

If Q633 is equal to 1 then skip to Q634

If not, then skip to instruction before Q635.

Q634. Which types of drugs did [Response to Q410] buy in the US? (Check all that apply) (Check all that apply)

__ Marijuana

__ Heroin

__ Crack cocaine by itself

__ Cocaine (powder) by itself

__ Heroin and cocaine together (speedball)

__ Methamphetamine/crystal by itself

__ Methamphetamine/crystal and heroin together

__ China White by itself

__ China White and heroin together

__ China White and crystal/meth together

__ PCP

__ Hallucinogenic drugs like acid, LSD, peyote, mescaline, mushrooms

__ Inhalants (for example, glue, gasoline, paint thinner, agua celeste or other chemicals)

__ Tranquilizers without a prescription (Rivotril, Diazepam, Valium, Ativan or Restoril)

__ OxyContin without a prescription

__ Other prescription opioids without a prescription (Vicodin, Darvon, Percocet)

__ Barbiturates without a prescription (Amytal, Nembutal, and Seconal)

__ Another drug or combination that was not already mentioned

__ Don't Know

__ Refuse to Answer

If Q634R is equal to 1 then skip to Q635

If not, then skip to instruction before Q636.

Q635. Please specify which types of drugs did [Response to Q410] buy in the US?

____

If Q627 is equal to 2 then skip to Q636

If not, then skip to Q637.

Q636. **In the past 6 months**, has [Response to Q410] had sex for money or drugs with someone from the United States?

1 Yes

0 No

7 Don't Know

8 Refuse to Answer

Q637. Has [Response to Q410] ever been detained or arrested?

1 Yes

0 No

7 Don't Know

8 Refuse to Answer

If Q405 is greater than 3 and Q412 is not equal to "Refuse to Answer" and Q412 is not equal to "skipped" then skip to Q638

If not, then skip to instruction before Q662.

Q638. How often does [Response to Q412] use drugs? (Choose one)

01	Every day
02	Several times a week
03	Once a week
04	Once every two weeks
05	Once a month
06	A couple of times a year
07	Once a year
08	Less than once a year
09	Never
10	Used to use drugs, but does not at the present time
97	Don't Know
98	Refuse to Answer

If Q638 is not less than 1 and Q638 is not greater than 8 then skip to Q639

If not, then skip to instruction before Q640.

Q639. Which of these does [Response to Q412] use? (Check all that apply) (Check all that apply)

__ Alcohol

__ Marijuana

__ Heroin by itself

__ Crack cocaine by itself

__ Cocaine powder by itself

__ Heroin and cocaine together (speedball)

__ Methamphetamine/crystal by itself

__ Methamphetamine/crystal and heroin together

__ China White by itself

__ China White and heroin together

__ China White and crystal/meth together

__ PCP

__ Hallucinogenic drugs like acid, LSD, peyote, mescaline, mushrooms

__ Inhalants (for example, glue, gasoline, paint thinner, agua celeste or other chemicals)

__ Tranquilizers without a prescription (Rivotril, Diazepam, Valium, Ativan or Restoril)

__ OxyContin without a prescription

__ Other prescription opioids without a prescription (Vicodin, Darvon, Percocet)

__ Barbiturates without a prescription (Amytal, Nembutal, and Seconal)

__ Another drug or combination that was not already mentioned

__ Don't Know

__ Refuse to Answer

If Q639S is equal to 1 then skip to Q640

If not, then skip to instruction before Q641.

Q640. Please specify what other drugs or combination of drugs [Response to Q412] uses.

____

If Q638 is not less than 1 and Q638 is not greater than 8 then skip to Q641

If not, then skip to instruction before Q642.

Q641. Does [Response to Q412] use drugs by injection, by noninjection, or sometimes one and sometimes the other? (Choose one)

1 Only uses drugs by injection

2 Only uses drugs by non-injection

3 Sometimes uses drugs by injection and sometimes by non-injection

7 Don't Know

8 Refuse to Answer

If Q641 is equal to 1 or Q641 is equal to 3 then skip to Q642

If not, then skip to instruction before Q643.

Q642. Have you ever used a needle, water, cooker or cotton that had already been used by [Response to Q412]?

1 Yes

0 No

8 Refuse to Answer

If Q638 is not equal to 9 and Q638 is not equal to 97 and Q638 is not equal to 98 then skip to Q643

If not, then skip to instruction before Q644.

Q643. Has [Response to Q412] ever offered to share or encouraged you to use drugs?

1	Yes
0	No
8	Refuse to Answer

If Q643 is equal to 1 then skip to Q644

If not, then skip to instruction before Q645.

Q644. Which of these has [Response to Q412] offered to share or encouraged you to use? (Check all that apply) (Check all that apply)

__ Alcohol

__ Marijuana

__ Heroin by itself

__ Crack cocaine by itself

__ Cocaine powder by itself

__ Heroin and cocaine together (speedball)

__ Methamphetamine/crystal by itself

__ Methamphetamine/crystal and heroin together

__ China White by itself

__ China White and heroin together

__ China White and crystal/meth together

__ PCP

__ Hallucinogenic drugs like acid, LSD, peyote, mescaline, mushrooms

__ Inhalants (for example, glue, gasoline, paint thinner, agua celeste or other chemicals)

__ Tranquilizers without a prescription (Rivotril, Diazepam, Valium, Ativan or Restoril)

__ OxyContin without a prescription

__ Other prescription opioids without a prescription (Vicodin, Darvon, Percocet)

__ Barbiturates without a prescription (Amytal, Nembutal, and Seconal)

__ Another drug or combination that was not already mentioned

__ Refuse to Answer

If Q644S is equal to 1 then skip to Q645

If not, then skip to Q646.

Q645. Please specify which of drugs has [Response to Q412] offered to share or encouraged you to use?

____

Q646. Has [Response to Q412] ever encouraged you to stop using drugs?

1 Yes

0 No

8 Refuse to Answer

If Q646 is equal to 1 then skip to Q647

If not, then skip to instruction before Q648.

Q647. Has [Response to Q412] ever encouraged you to specifically stop using drugs by injection?

1 Yes

0 No

7 Don't Know

8 Refuse to Answer

If Q638 is not equal to 9 and Q638 is not equal to 97 and Q638 is not equal to 98 then skip to Q648

If not, then skip to instruction before Q649.

Q648. How old was [Response to Q412] when they first started using drugs?

__ __ years old

97 Don't Know

98 Refuse to Answer

If Q638 is not equal to 9 and Q638 is not equal to 10 and Q638 is not equal to 97 and Q638 is not equal to 98 then skip to Q649

If not, then skip to Q650.

Q649. Consider [Response to Q412] usual daily drug use, does [Response to Q412] ever use double the amount or mix with other drugs?

1 Yes

0 No

7 Don't Know

8 Refuse to Answer

Q650. Has [Response to Q412] ever lived on the street, in a shelter, in a Single Room Occupancy hotel (SRO), temporarily with friends/relatives, in a car, or squatted?

1 Yes

0 No

7 Don't Know

8 Refuse to Answer

Q651. Where does [Response to Q412] currently live? (Choose one)

1 San Diego County

2 Tijuana

3 Other

7 Don't Know

8 Refuse to Answer

If Q651 is equal to 3 then skip to Q652

If not, then skip to instruction before Q653.

Q652. Please specify where [Response to Q412] lives.

____

If Q651 is equal to 1 and Q638 is not equal to 9 and Q638 is not equal to 97 and Q638 is not equal to 98 then skip to Q653

If not, then skip to instruction before Q654.

Q653. **In the past 6 months**, had [Response to Q412] traveled to Mexico to buy or obtain drugs?

1	Yes
0	No
7	Don't Know
8	Refuse to Answer

If Q653 is equal to 1 then skip to Q654

If not, then skip to instruction before Q655.

Q654. Which types of drugs did [Response to Q412] buy in Mexico? (Check all that apply) (Check all that apply)

__ Marijuana

__ Heroin

__ Crack cocaine by itself

__ Cocaine (powder) by itself

__ Heroin and cocaine together (speedball)

__ Methamphetamine/crystal by itself

__ Methamphetamine/crystal and heroin together

__ China White by itself

__ China White and heroin together

__ China White and crystal/meth together

__ PCP

__ Hallucinogenic drugs like acid, LSD, peyote, mescaline, mushrooms

__ Inhalants (for example, glue, gasoline, paint thinner, agua celeste or other chemicals)

__ Tranquilizers without a prescription (Rivotril, Diazepam, Valium, Ativan or Restoril)

__ OxyContin without a prescription

__ Other prescription opioids without a prescription (Vicodin, Darvon, Percocet)

__ Barbiturates without a prescription (Amytal, Nembutal, and Seconal)

__ Another drug or combination that was not already mentioned

__ Don't Know

__ Refuse to Answer

If Q654R is equal to 1 then skip to Q655

If not, then skip to instruction before Q656.

Q655. Please specify which types of drugs did [Response to Q412] buy in Mexico?

____

If Q651 is equal to 1 then skip to Q656

If not, then skip to instruction before Q657.

Q656. **In the past 6 months**, had [Response to Q412] traveled to Mexico to pay money or drugs for sex?

1 Yes

0 No

7 Don't Know

8 Refuse to Answer

If Q651 is equal to 2 and Q638 is not equal to 9 and Q638 is not equal to 97 and Q638 is not equal to 98 then skip to Q657

If not, then skip to instruction before Q658.

Q657. **In the past 6 months**, had [Response to Q412] traveled to the United states to buy or obtain drugs?

1 Yes

0 No

7 Don't Know

8 Refuse to Answer

If Q657 is equal to 1 then skip to Q658

If not, then skip to instruction before Q659.

Q658. Which types of drugs did [Response to Q412] buy in the US? (Check all that apply) (Check all that apply)

__ Marijuana

__ Heroin

__ Crack cocaine by itself

__ Cocaine (powder) by itself

__ Heroin and cocaine together (speedball)

__ Methamphetamine/crystal by itself

__ Methamphetamine/crystal and heroin together

__ China White by itself

__ China White and heroin together

__ China White and crystal/meth together

__ PCP

__ Hallucinogenic drugs like acid, LSD, peyote, mescaline, mushrooms

__ Inhalants (for example, glue, gasoline, paint thinner, agua celeste or other chemicals)

__ Tranquilizers without a prescription (Rivotril, Diazepam, Valium, Ativan or Restoril)

__ OxyContin without a prescription

__ Other prescription opioids without a prescription (Vicodin, Darvon, Percocet)

__ Barbiturates without a prescription (Amytal, Nembutal, and Seconal)

__ Another drug or combination that was not already mentioned

__ Don't Know

__ Refuse to Answer

If Q658R is equal to 1 then skip to Q659

If not, then skip to instruction before Q660.

Q659. Please specify which types of drugs did [Response to Q412] buy in the US?

____

If Q651 is equal to 2 then skip to Q660

If not, then skip to Q661.

Q660. **In the past 6 months**, has [Response to Q412] had sex for money or drugs with someone from the United States?

1	Yes
0	No
7	Don't Know
8	Refuse to Answer

Q661. Has [Response to Q412] ever been detained or arrested?

1	Yes
0	No
7	Don't Know
8	Refuse to Answer

If Q405 is greater than 4 and Q414 is not equal to "Refuse to Answer" and Q414 is not equal to "skipped" then skip to Q662

If not, then skip to instruction before Q686.

Q662. How often does [Response to Q414] use drugs? (Choose one)

01	Every day
02	Several times a week
03	Once a week
04	Once every two weeks
05	Once a month
06	A couple of times a year
07	Once a year
08	Less than once a year
09	Never
10	Used to use drugs, but does not at the present time
97	Don't Know
98	Refuse to Answer

If Q662 is not less than 1 and Q662 is not greater than 8 then skip to Q663

If not, then skip to instruction before Q664.

Q663. Which of these does [Response to Q414] use? (Check all that apply) (Check all that apply)

__ Alcohol

__ Marijuana

__ Heroin by itself

__ Crack cocaine by itself

__ Cocaine powder by itself

__ Heroin and cocaine together (speedball)

__ Methamphetamine/crystal by itself

__ Methamphetamine/crystal and heroin together

__ China White by itself

__ China White and heroin together

__ China White and crystal/meth together

__ PCP

__ Hallucinogenic drugs like acid, LSD, peyote, mescaline, mushrooms

__ Inhalants (for example, glue, gasoline, paint thinner, agua celeste or other chemicals)

__ Tranquilizers without a prescription (Rivotril, Diazepam, Valium, Ativan or Restoril)

__ OxyContin without a prescription

__ Other prescription opioids without a prescription (Vicodin, Darvon, Percocet)

__ Barbiturates without a prescription (Amytal, Nembutal, and Seconal)

__ Another drug or combination that was not already mentioned

__ Don't Know

__ Refuse to Answer

If Q663S is equal to 1 then skip to Q664

If not, then skip to instruction before Q665.

Q664. Please specify what other drugs or combination of drugs [Response to Q414] uses.

____

If Q662 is not less than 1 and Q662 is not greater than 8 then skip to Q665

If not, then skip to instruction before Q666.

Q665. Does [Response to Q414] use drugs by injection, by noninjection, or sometimes one and sometimes the other? (Choose one)

1 Only uses drugs by injection

2 Only uses drugs by non-injection

3 Sometimes uses drugs by injection and sometimes by non-injection

7 Don't Know

8 Refuse to Answer

If Q665 is equal to 1 or Q665 is equal to 3 then skip to Q666

If not, then skip to instruction before Q667.

Q666. Have you ever used a needle, water, cooker or cotton that had already been used by [Response to Q414]?

1 Yes

0 No

8 Refuse to Answer

If Q662 is not equal to 9 and Q662 is not equal to 97 and Q662 is not equal to 98 then skip to Q667

If not, then skip to instruction before Q668.

Q667. Has [Response to Q414] ever offered to share or encouraged you to use drugs?

1	Yes
0	No
8	Refuse to Answer

If Q667 is equal to 1 then skip to Q668

If not, then skip to instruction before Q669.

Q668. Which of these has [Response to Q414] offered to share or encouraged you to use? (Check all that apply) (Check all that apply)

__ Alcohol

__ Marijuana

__ Heroin by itself

__ Crack cocaine by itself

__ Cocaine powder by itself

__ Heroin and cocaine together (speedball)

__ Methamphetamine/crystal by itself

__ Methamphetamine/crystal and heroin together

__ China White by itself

__ China White and heroin together

__ China White and crystal/meth together

__ PCP

__ Hallucinogenic drugs like acid, LSD, peyote, mescaline, mushrooms

__ Inhalants (for example, glue, gasoline, paint thinner, agua celeste or other chemicals)

__ Tranquilizers without a prescription (Rivotril, Diazepam, Valium, Ativan or Restoril)

__ OxyContin without a prescription

__ Other prescription opioids without a prescription (Vicodin, Darvon, Percocet)

__ Barbiturates without a prescription (Amytal, Nembutal, and Seconal)

__ Another drug or combination that was not already mentioned

__ Refuse to Answer

If Q668S is equal to 1 then skip to Q669

If not, then skip to Q670.

Q669. Please specify which of drugs has [Response to Q414] offered to share or encouraged you to use?

____

Q670. Has [Response to Q414] ever encouraged you to stop using drugs?

1 Yes

0 No

8 Refuse to Answer

If Q670 is equal to 1 then skip to Q671

If not, then skip to instruction before Q672.

Q671. Has [Response to Q414] ever encouraged you to specifically stop using drugs by injection?

1 Yes

0 No

7 Don't Know

8 Refuse to Answer

If Q662 is not equal to 9 and Q662 is not equal to 97 and Q662 is not equal to 98 then skip to Q672

If not, then skip to instruction before Q673.

Q672. How old was [Response to Q414] when they first started using drugs?

__ __ years old

97 Don't Know

98 Refuse to Answer

If Q662 is not equal to 9 and Q662 is not equal to 10 and Q662 is not equal to 97 and Q662 is not equal to 98 then skip to Q673

If not, then skip to Q674.

Q673. Consider [Response to Q414] usual daily drug use, does [Response to Q414] ever use double the amount or mix with other drugs?

1 Yes

0 No

7 Don't Know

8 Refuse to Answer

Q674. Has [Response to Q414] ever lived on the street, in a shelter, in a Single Room Occupancy hotel (SRO), temporarily with friends/relatives, in a car, or squatted?

1 Yes

0 No

7 Don't Know

8 Refuse to Answer

Q675. Where does [Response to Q414] currently live? (Choose one)

1 San Diego County

2 Tijuana

3 Other

7 Don't Know

8 Refuse to Answer

If Q675 is equal to 3 then skip to Q676

If not, then skip to instruction before Q677.

Q676. Please specify where [Response to Q414] lives.

____

If Q675 is equal to 1 and Q662 is not equal to 9 and Q662 is not equal to 97 and Q662 is not equal to 98 then skip to Q677

If not, then skip to instruction before Q678.

Q677. **In the past 6 months**, had [Response to Q414] traveled to Mexico to buy or obtain drugs?

1	Yes
0	No
7	Don't Know
8	Refuse to Answer

If Q677 is equal to 1 then skip to Q678

If not, then skip to instruction before Q679.

Q678. Which types of drugs did [Response to Q414] buy in Mexico? (Check all that apply) (Check all that apply)

__ Marijuana

__ Heroin

__ Crack cocaine by itself

__ Cocaine (powder) by itself

__ Heroin and cocaine together (speedball)

__ Methamphetamine/crystal by itself

__ Methamphetamine/crystal and heroin together

__ China White by itself

__ China White and heroin together

__ China White and crystal/meth together

__ PCP

__ Hallucinogenic drugs like acid, LSD, peyote, mescaline, mushrooms

__ Inhalants (for example, glue, gasoline, paint thinner, agua celeste or other chemicals)

__ Tranquilizers without a prescription (Rivotril, Diazepam, Valium, Ativan or Restoril)

__ OxyContin without a prescription

__ Other prescription opioids without a prescription (Vicodin, Darvon, Percocet)

__ Barbiturates without a prescription (Amytal, Nembutal, and Seconal)

__ Another drug or combination that was not already mentioned

__ Don't Know

__ Refuse to Answer

If Q678R is equal to 1 then skip to Q679

If not, then skip to instruction before Q680.

Q679. Please specify which types of drugs did [Response to Q414] buy in Mexico?

____

If Q675 is equal to 1 then skip to Q680

If not, then skip to instruction before Q681.

Q680. **In the past 6 months**, had [Response to Q414] traveled to Mexico to pay money or drugs for sex?

1 Yes

0 No

7 Don't Know

8 Refuse to Answer

If Q675 is equal to 2 and Q662 is not equal to 9 and Q662 is not equal to 97 and Q662 is not equal to 98 then skip to Q681

If not, then skip to instruction before Q682.

Q681. **In the past 6 months**, had [Response to Q414] traveled to the United states to buy or obtain drugs?

1 Yes

0 No

7 Don't Know

8 Refuse to Answer

If Q681 is equal to 1 then skip to Q682

If not, then skip to instruction before Q683.

Q682. Which types of drugs did [Response to Q414] buy in the US? (Check all that apply) (Check all that apply)

__ Marijuana

__ Heroin

__ Crack cocaine by itself

__ Cocaine (powder) by itself

__ Heroin and cocaine together (speedball)

__ Methamphetamine/crystal by itself

__ Methamphetamine/crystal and heroin together

__ China White by itself

__ China White and heroin together

__ China White and crystal/meth together

__ PCP

__ Hallucinogenic drugs like acid, LSD, peyote, mescaline, mushrooms

__ Inhalants (for example, glue, gasoline, paint thinner, agua celeste or other chemicals)

__ Tranquilizers without a prescription (Rivotril, Diazepam, Valium, Ativan or Restoril)

__ OxyContin without a prescription

__ Other prescription opioids without a prescription (Vicodin, Darvon, Percocet)

__ Barbiturates without a prescription (Amytal, Nembutal, and Seconal)

__ Another drug or combination that was not already mentioned

__ Don't Know

__ Refuse to Answer

If Q682R is equal to 1 then skip to Q683

If not, then skip to instruction before Q684.

Q683. Please specify which types of drugs did [Response to Q414] buy in the US?

____

If Q675 is equal to 2 then skip to Q684

If not, then skip to Q685.

Q684. **In the past 6 months**, has [Response to Q414] had sex for money or drugs with someone from the United States?

1	Yes
0	No
7	Don't Know
8	Refuse to Answer

Q685. Has [Response to Q414] ever been detained or arrested?

1	Yes
0	No
7	Don't Know
8	Refuse to Answer

CoV-2 SOCIAL NETWORK COMPONENT

READ: Now we are going to switch gears and talk about more recent times that you have spent time with other people. As before, we are trying to learn about how people are connected to one another and how those connections relate to preventing diseases, but now we will focus on CoV-2 infection. Having names and demographic information will help us see how people in this study are connected to one another, for example through mutual friends.

Please keep in mind that everything you tell me is confidential and that we will protect your privacy and the privacy of the people you mention. We will never at any time use this information to contact the people you tell us about.

READ: Please think about the following types of people you've had close contact with in the past 14 days: romantic or sex partners or spouse; other family-relatives by blood or marriage; close friends; roommates; neighbors; work related (co-workers, bosses, customers); classmates; drug or alcohol related; people who provide you a service; others (e.g., people you saw at community groups, religious services, sports or exercise classes, school, etc.)

We are interested in people you've had either: In person, non-physical contact with (within 6ft) that lasted over an hour OR Physical contact with.

We'll start by first listing their names (first, last, nickname). Feel free to look through your phone to jog your memory. This is important. We will never share the names you provide us. Only the researchers will have access to these names.

Q686. Let's start with the people from the category **Romantic or sex partners or spouse** that you had close contact with in the past 14 days (again by close contact, we mean either: non-physical contact with (within 6ft) that lasted over an hour **OR** Physical contact). How many of them have you had contact with? Next, we'll list their names (up to 5 names may be listed).

— —

98 Refuse to Answer

If Q686 is greater than 0 then skip to Q687

If not, then skip to instruction before Q691.

Q687. **Romantic or sex partner or spouse**: Person #1 First Name

— —

Q688. **Romantic or sex partner or spouse**: Person #1 Last Name

— —

Q689. **Romantic or sex partner or spouse**: Person #1 Nick Name

— —

Q690. **Romantic or sex partner or spouse**: Person #1 Age

__ __	years old
97	Don't Know
98	Refuse to Answer

If Q686 is greater than 1 then skip to Q691

If not, then skip to instruction before Q695.

Q691. **Romantic or sex partner or spouse**: Person #2 First Name

____

Q692. **Romantic or sex partner or spouse**: Person #2 Last Name

____

Q693. **Romantic or sex partner or spouse**: Person #2 Nick Name

____

Q694. **Romantic or sex partner or spouse**: Person #2 Age

__ __	years old
97	Don't Know
98	Refuse to Answer

If Q686 is greater than 2 then skip to Q695

If not, then skip to instruction before Q699.

Q695. **Romantic or sex partner or spouse**: Person #3 First Name

____

Q696. **Romantic or sex partner or spouse**: Person #3 Last Name

____

Q697. **Romantic or sex partner or spouse**: Person #3 Nick Name

____

Q698. **Romantic or sex partner or spouse**: Person #3 Age __ __ years old

97 Don't Know

98 Refuse to Answer

If Q686 is greater than 3 then skip to Q699

If not, then skip to instruction before Q703.

Q699. **Romantic or sex partner or spouse**: Person #4 First Name

____

Q700. **Romantic or sex partner or spouse**: Person #4 Last Name

____

Q701. **Romantic or sex partner or spouse**: Person #4 Nick Name

____

Q702. **Romantic or sex partner or spouse**: Person #4 Age __ __ years old

97 Don't Know

98 Refuse to Answer

If Q686 is greater than 4 then skip to Q703

If not, then skip to Q707.

Q703. **Romantic or sex partner or spouse**: Person #5 First Name

____

Q704. **Romantic or sex partner or spouse**: Person #5 Last Name

____

Q705. **Romantic or sex partner or spouse**: Person #5 Nick Name

____

Q706. **Romantic or sex partner or spouse**: Person #5 Age

__ __ years old

97 Don't Know

98 Refuse to Answer

Q707. **Now, please think of other family-relatives by blood or marriage** that you have had close contact with in the past 14 days. How many of them have you had contact with?

__ __

98 Refuse to Answer

If Q707 is greater than 0 then skip to Q708

If not, then skip to instruction before Q712.

Q708. Next, we'll list their names (up to 5 names may be listed).
Family relative by blood or marriage: Person #1 First Name

____

Q709. **Family Relative by Blood or Marriage**: Person #1 Last Name

____

Q710. **Family Relative by Blood or Marriage**: Person #1 Nick Name

____

Q711. **Family Relative by Blood or Marriage:** Person #1 Age

__ __ years old

97 Don't Know

98 Refuse to Answer

If Q707 is greater than 1 then skip to Q712

If not, then skip to instruction before Q716.

Q712. **Family relative by blood or marriage**: Person #2 First Name

____

Q713. **Family Relative by Blood or Marriage**: Person #2 Last Name

____

Q714. **Family Relative by Blood or Marriage**: Person #2 Nick Name

____

Q715. **Family Relative by Blood or Marriage:** Person #2 Age

__ __	years old
97	Don't Know
98	Refuse to Answer

If Q707 is greater than 2 then skip to Q716

If not, then skip to instruction before Q720.

Q716. **Family relative by blood or marriage**: Person #3 First Name

____

Q717. **Family Relative by Blood or Marriage**: Person #3 Last Name

____

Q718. **Family Relative by Blood or Marriage**: Person #3 Nick Name

____

Q719. **Family Relative by Blood or Marriage:** Person #3 Age

__ __	years old
97	Don't Know
98	Refuse to Answer

If Q707 is greater than 3 then skip to Q720

If not, then skip to instruction before Q724.

Q720. **Family relative by blood or marriage**: Person #4 First Name

____

Q721. **Family Relative by Blood or Marriage**: Person #4 Last Name

____

Q722. **Family Relative by Blood or Marriage**: Person #4 Nick Name

____

Q723. **Family Relative by Blood or Marriage:** Person #4 Age

__ __	years old
97	Don't Know
98	Refuse to Answer

If Q707 is greater than 4 then skip to Q724

If not, then skip to Q728.

Q724. **Family relative by blood or marriage**: Person #5 First Name

____

Q725. **Family Relative by Blood or Marriage**: Person #5 Last Name

____

Q726. **Family Relative by Blood or Marriage**: Person #5 Nick Name

____

Q727. **Family Relative by Blood or Marriage:** Person #5 Age

__ __	years old
97	Don't Know
98	Refuse to Answer

Q728. **Now, please think of close friends** that you have had close contact with in the past 14 days. How many of them have you had contact with?

__ __	
98	Refuse to Answer

If Q728 is greater than 0 then skip to Q729

If not, then skip to instruction before Q733.

Q729. Next, we'll list their names (up to 5 names may be listed).
Close Friend: Person #1 First Name

____

Q730. **Close friend**: Person #1 Last Name

____

Q731. **Close Friend**: Person #1 Nick Name

____

Q732. **Close Friend:** Person #1 Age

__ __	years old
97	Don't Know
98	Refuse to Answer

If Q728 is greater than 1 then skip to Q733

If not, then skip to instruction before Q737.

Q733. **Close Friend:** Person #2 First Name

____

Q734. **Close friend**: Person #2 Last Name

____

Q735. **Close Friend**: Person #2 Nick Name

____

Q736. **Close Friend:** Person #2 Age

__ __	years old
97	Don't Know
98	Refuse to Answer

If Q728 is greater than 2 then skip to Q737

If not, then skip to instruction before Q741.

Q737. **Close Friend:** Person #3 First Name

____

Q738. **Close friend**: Person #3 Last Name

____

Q739. **Close Friend**: Person #3 Nick Name

____

Q740. **Close Friend:** Person #3 Age

__ __	years old
97	Don't Know
98	Refuse to Answer

If Q728 is greater than 3 then skip to Q741

If not, then skip to instruction before Q745.

Q741. **Close Friend:** Person #4 First Name

____

Q742. **Close friend**: Person #4 Last Name

____

Q743. **Close Friend**: Person #4 Nick Name

____

Q744. **Close Friend:** Person #4 Age

__ __	years old
97	Don't Know
98	Refuse to Answer

If Q728 is greater than 4 then skip to Q745

If not, then skip to Q749.

Q745. **Close Friend:** Person #5 First Name

____

Q746. **Close friend**: Person #5 Last Name

____

Q747. **Close Friend**: Person #5 Nick Name

____

Q748. **Close Friend:** Person #5 Age

__ __	years old
97	Don't Know
98	Refuse to Answer

Q749. **Now, please think of roommates *(i.e. housemates, flat mates)*** that you have had close contact with in the past 14 days. How many of them have you had contact with?

__ __	
98	Refuse to Answer

If Q749 is greater than 0 then skip to Q750

If not, then skip to instruction before Q754.

Q750. Next, we'll list their names (up to 5 names may be listed).
Roommate: Person #1 First Name

____

Q751. **Roommate**: Person #1 Last Name

____

Q752. **Roommate**: Person #1 Nick Name

____

Q753. **Roommate:** Person #1 Age

__ __	years old
97	Don't Know
98	Refuse to Answer

If Q749 is greater than 1 then skip to Q754

If not, then skip to instruction before Q758.

Q754. **Roommate:** Person #2 First Name

____

Q755. **Roommate**: Person #2 Last Name

____

Q756. **Roommate**: Person #2 Nick Name

____

Q757. **Roommate:** Person #2 Age

__ __	years old
97	Don't Know
98	Refuse to Answer

If Q749 is greater than 2 then skip to Q758

If not, then skip to instruction before Q762.

Q758. **Roommate:** Person #3 First Name

____

Q759. **Roommate**: Person #3 Last Name

____

Q760. **Roommate**: Person #3 Nick Name

____

Q761. **Roommate:** Person #3 Age

__ __	years old
97	Don't Know
98	Refuse to Answer

If Q749 is greater than 3 then skip to Q762

If not, then skip to instruction before Q766.

Q762. **Roommate:** Person #4 First Name

____

Q763. **Roommate**: Person #4 Last Name

____

Q764. **Roommate**: Person #4 Nick Name

____

Q765. **Roommate:** Person #4 Age

__ __	years old
97	Don't Know
98	Refuse to Answer

If Q749 is greater than 4 then skip to Q766

If not, then skip to Q770.

Q766. **Roommate:** Person #5 First Name

____

Q767. **Roommate**: Person #5 Last Name

____

Q768. **Roommate**: Person #5 Nick Name

____

Q769. **Roommate:** Person #5 Age

__ __ years old

97 Don't Know

98 Refuse to Answer

Q770. **Now, please think of neighbors** that you have had close contact with in the past 14 days. How many of them have you had contact with?

__ __

98 Refuse to Answer

If Q770 is greater than 0 then skip to Q771

If not, then skip to instruction before Q775.

Q771. Next, we'll list their names (up to 5 names may be listed).
Neighbor: Person #1 First Name

____

Q772. **Neighbor:** Person #1 Last Name

____

Q773. **Neighbor**: Person #1 Nick Name

____

Q774. **Neighbor:** Person #1 Age

__ __ years old

97 Don't Know

98 Refuse to Answer

If Q770 is greater than 1 then skip to Q775

If not, then skip to instruction before Q779.

Q775. **Neighbor:** Person #2 First Name

____

Q776. **Neighbor:** Person #2 Last Name

____

Q777. **Neighbor**: Person #2 Nick Name

____

Q778. **Neighbor:** Person #2 Age

__ __	years old
97	Don't Know
98	Refuse to Answer

If Q770 is greater than 2 then skip to Q779

If not, then skip to instruction before Q783.

Q779. **Neighbor:** Person #3 First Name

____

Q780. **Neighbor:** Person #3 Last Name

____

Q781. **Neighbor**: Person #3 Nick Name

____

Q782. **Neighbor:** Person #3 Age

__ __	years old
97	Don't Know
98	Refuse to Answer

If Q770 is greater than 3 then skip to Q783

If not, then skip to instruction before Q787.

Q783. **Neighbor:** Person #4 First Name

____

Q784. **Neighbor:** Person #4 Last Name

____

Q785. **Neighbor**: Person #4 Nick Name

____

Q786. **Neighbor:** Person #4 Age

__ __	years old
97	Don't Know
98	Refuse to Answer

If Q770 is greater than 4 then skip to Q787

If not, then skip to Q791.

Q787. **Neighbor:** Person #5 First Name

____

Q788. **Neighbor:** Person #5 Last Name

____

Q789. **Neighbor**: Person #5 Nick Name

____

Q790. **Neighbor:** Person #5 Age

__ __	years old
97	Don't Know
98	Refuse to Answer

Q791. **Now, please think of work related people (co-workers, bosses, customers)** that you have had close contact with in the past 14 days. How many of them have you had contact with?

__ __	
98	Refuse to Answer

If Q791 is greater than 0 then skip to Q792

If not, then skip to instruction before Q796.

Q792. Next, we'll list their names (up to 5 names may be listed).
Work Related: Person #1 First Name

____

Q793. **Work Related:** Person #1 Last Name

____

Q794. **Work Related**: Person #1 Nick Name

____

Q795. **Work Related:** Person #1 Age

__ __	years old
97	Don't Know
98	Refuse to Answer

If Q791 is greater than 1 then skip to Q796

If not, then skip to instruction before Q800.

Q796. **Work Related:** Person #2 First Name

____

Q797. **Work Related:** Person #2 Last Name

____

Q798. **Work Related**: Person #2 Nick Name

____

Q799. **Work Related:** Person #2 Age

__ __	years old
97	Don't Know
98	Refuse to Answer

If Q791 is greater than 2 then skip to Q800

If not, then skip to instruction before Q804.

Q800. **Work Related:** Person #3 First Name

____

Q801. **Work Related:** Person #3 Last Name

____

Q802. **Work Related**: Person #3 Nick Name

____

Q803. **Work Related:** Person #3 Age

__ __	years old
97	Don't Know
98	Refuse to Answer

If Q791 is greater than 3 then skip to Q804

If not, then skip to instruction before Q808.

Q804. **Work Related:** Person #4 First Name

____

Q805. **Work Related:** Person #4 Last Name

____

Q806. **Work Related**: Person #4 Nick Name

____

Q807. **Work Related:** Person #4 Age

__ __	years old
97	Don't Know
98	Refuse to Answer

If Q791 is greater than 4 then skip to Q808

If not, then skip to Q812.

Q808. **Work Related:** Person #5 First Name

____

Q809. **Work Related:** Person #5 Last Name

____

Q810. **Work Related**: Person #5 Nick Name

____

Q811. **Work Related:** Person #5 Age

__ __	years old
97	Don't Know
98	Refuse to Answer

Q812. **Now, please think of classmates** that you have had close contact with in the past 14 days. How many of them have you had contact with?

__ __	
98	Refuse to Answer

If Q812 is greater than 0 then skip to Q813

If not, then skip to instruction before Q817.

Q813. Next, we'll list their names (up to 5 names may be listed).
Classmate: Person #1 First Name

____

Q814. **Classmate:** Person #1 Last Name

____

Q815. **Classmate**: Person #1 Nick Name

____

Q816. **Classmate:** Person #1 Age

__ __	years old
97	Don't Know
98	Refuse to Answer

If Q812 is greater than 1 then skip to Q817

If not, then skip to instruction before Q821.

Q817. **Classmate:** Person #2 First Name

____

Q818. **Classmate:** Person #2 Last Name

____

Q819. **Classmate**: Person #2 Nick Name

____

Q820. **Classmate:** Person #2 Age

__ __	years old
97	Don't Know
98	Refuse to Answer

If Q812 is greater than 2 then skip to Q821

If not, then skip to instruction before Q825.

Q821. **Classmate:** Person #3 First Name

____

Q822. **Classmate:** Person #3 Last Name

____

Q823. **Classmate**: Person #3 Nick Name

____

Q824. **Classmate:** Person #3 Age __ __ years old

97 Don't Know

98 Refuse to Answer

If Q812 is greater than 3 then skip to Q825

If not, then skip to instruction before Q829.

Q825. **Classmate:** Person #4 First Name

____

Q826. **Classmate:** Person #4 Last Name

____

Q827. **Classmate**: Person #4 Nick Name

____

Q828. **Classmate:** Person #4 Age __ __ years old

97 Don't Know

98 Refuse to Answer

If Q812 is greater than 4 then skip to Q829

If not, then skip to Q833.

Q829. **Classmate:** Person #5 First Name

____

Q830. **Classmate:** Person #5 Last Name

____

Q831. **Classmate**: Person #5 Nick Name

____

Q832. **Classmate:** Person #5 Age

__ __ years old

97 Don't Know

98 Refuse to Answer

Q833. **Now, please think of drugs or alcohol related persons** that you have had close contact with in the past 14 days. How many of them have you had contact with?

__ __

98 Refuse to Answer

If Q833 is greater than 0 then skip to Q834

If not, then skip to instruction before Q838.

Q834. Next, we'll list their names (up to 5 names may be listed).
Drugs or alcohol related: Person #1 First Name

__ __

Q835. **Drugs or alcohol related:** Person #1 Last Name

__ __

Q836. **Drugs or alcohol related**: Person #1 Nick Name

__ __

Q837. **Drugs or alcohol related:** Person #1 Age

__ __ years old

97 Don't Know

98 Refuse to Answer

If Q833 is greater than 1 then skip to Q838

If not, then skip to instruction before Q842.

Q838. **Drugs or alcohol related:** Person #2 First Name

__ __

Q839. **Drugs or alcohol related:** Person #2 Last Name

____

Q840. **Drugs or alcohol related**: Person #2 Nick Name

____

Q841. **Drugs or alcohol related:** Person #2 Age

__ __	years old
97	Don't Know
98	Refuse to Answer

If Q833 is greater than 2 then skip to Q842

If not, then skip to instruction before Q846.

Q842. **Drugs or alcohol related:** Person #3 First Name

____

Q843. **Drugs or alcohol related:** Person #3 Last Name

____

Q844. **Drugs or alcohol related**: Person #3 Nick Name

____

Q845. **Drugs or alcohol related:** Person #3 Age

__ __	years old
97	Don't Know
98	Refuse to Answer

If Q833 is greater than 3 then skip to Q846

If not, then skip to instruction before Q850.

Q846. **Drugs or alcohol related:** Person #4 First Name

____

Q847. **Drugs or alcohol related:** Person #4 Last Name

____

Q848. **Drugs or alcohol related**: Person #4 Nick Name

____

Q849. **Drugs or alcohol related:** Person #4 Age

__ __	years old
97	Don't Know
98	Refuse to Answer

If Q833 is greater than 4 then skip to Q850

If not, then skip to Q854.

Q850. **Drugs or alcohol related:** Person #5 First Name

____

Q851. **Drugs or alcohol related:** Person #5 Last Name

____

Q852. **Drugs or alcohol related**: Person #5 Nick Name

____

Q853. **Drugs or alcohol related:** Person #5 Age

__ __	years old
97	Don't Know
98	Refuse to Answer

Q854. **Now, please think of *people who provide you a service*** that you have had close contact with in the past 14 days. How many of them have you had contact with?

__ __	
98	Refuse to Answer

If Q854 is greater than 0 then skip to Q855

If not, then skip to instruction before Q859.

Q855. Next, we'll list their names (up to 5 names may be listed).
People who provide you a service: Person #1 First Name

____

Q856. **People who provide you a service:** Person #1 Last Name

____

Q857. **People who provide you a service**: Person #1 Nick Name

____

Q858. **People who provide you a service**: Person #1 Age

__ __	years old
97	Don't Know
98	Refuse to Answer

If Q854 is greater than 1 then skip to Q859

If not, then skip to instruction before Q863.

Q859. **People who provide you a service:** Person #2 First Name

____

Q860. **People who provide you a service:** Person #2 Last Name

____

Q861. **People who provide you a service**: Person #2 Nick Name

____

Q862. **People who provide you a service**: Person #2 Age

__ __	years old
97	Don't Know
98	Refuse to Answer

If Q854 is greater than 2 then skip to Q863

If not, then skip to instruction before Q867.

Q863. **People who provide you a service:** Person #3 First Name

____

Q864. **People who provide you a service:** Person #3 Last Name

____

Q865. **People who provide you a service**: Person #3 Nick Name

____

Q866. **People who provide you a service**: Person #3 Age

__ __	years old
97	Don't Know
98	Refuse to Answer

If Q854 is greater than 3 then skip to Q867

If not, then skip to instruction before Q871.

Q867. **People who provide you a service:** Person #4 First Name

____

Q868. **People who provide you a service:** Person #4 Last Name

____

Q869. **People who provide you a service**: Person #4 Nick Name

____

Q870. **People who provide you a service**: Person #4 Age

__ __	years old
97	Don't Know
98	Refuse to Answer

If Q854 is greater than 4 then skip to Q871

If not, then skip to Q875.

Q871. **People who provide you a service:** Person #5 First Name

____

Q872. **People who provide you a service:** Person #5 Last Name

____

Q873. **People who provide you a service**: Person #5 Nick Name

____

Q874. **People who provide you a service**: Person #5 Age

__ __	years old
97	Don't Know
98	Refuse to Answer

Q875. **Now, please think of *other people not mentioned previously (e.g., people you saw at community groups, religious services, sports or exercise classes, school, etc.)*** that you have had close contact with in the past 14 days. How many of them have you had contact with?

__ __	
98	Refuse to Answer

If Q875 is greater than 0 then skip to Q876

If not, then skip to instruction before Q880.

Q876. Next, we'll list their names (up to 5 names may be listed).
Other people: Person #1 First Name

____

Q877. **Other people:** Person #1 Last Name

____

Q878. **Other people**: Person #1 Nick Name

____

Q879. **Other people**: Person #1 Age

__ __	years old
97	Don't Know
98	Refuse to Answer

If Q875 is greater than 1 then skip to Q880

If not, then skip to instruction before Q884.

Q880. **Other people:** Person #2 First Name

____

Q881. **Other people:** Person #2 Last Name

____

Q882. **Other people**: Person #2 Nick Name

____

Q883. **Other people**: Person #2 Age

__ __	years old
97	Don't Know
98	Refuse to Answer

If Q875 is greater than 2 then skip to Q884

If not, then skip to instruction before Q888.

Q884. **Other people:** Person #3 First Name

____

Q885. **Other people:** Person #3 Last Name

____

Q886. **Other people**: Person #3 Nick Name

____

Q887. **Other people**: Person #3 Age __ __ years old

97 Don't Know

98 Refuse to Answer

If Q875 is greater than 3 then skip to Q888

If not, then skip to instruction before Q892.

Q888. **Other people:** Person #4 First Name

____

Q889. **Other people:** Person #4 Last Name

____

Q890. **Other people**: Person #4 Nick Name

____

Q891. **Other people**: Person #4 Age __ __ years old

97 Don't Know

98 Refuse to Answer

If Q875 is greater than 4 then skip to Q892

If not, then skip to instruction before Q896.

Q892. **Other people:** Person #5 First Name

____

Q893. **Other people:** Person #5 Last Name

____

Q894. **Other people**: Person #5 Nick Name

____

Q895. **Other people**: Person #5 Age

__ __	years old
97	Don't Know
98	Refuse to Answer

If Q686 + Q707 + Q728 + Q749 + Q770 + Q791 + Q812 + Q833 + Q854 + Q875 is greater than 0 then skip to instruction before Q896

If not, then skip to instruction before Q899.

READ: Of the people you listed, please think about which 5 people you've had the most contact with. This could be the longest number of hours of non-physical contact (within 6 ft) with a person and/or the most physical contact.

Q896. Next, we'll list their names.
Most Contact: Person #1 First Name

____

Q897. **Most Contact:** Person #1 Last Name

____

Q898. **Most Contact**: Person #1 Nick Name

____

If Q686 + Q707 + Q728 + Q749 + Q770 + Q791 + Q812 + Q833 + Q854 + Q875 is greater than 1 then skip to Q899

If not, then skip to instruction before Q902.

Q899. **Most Contact:** Person #2 First Name

____

Q900. **Most Contact:** Person #2 Last Name

____

Q901. **Most Contact**: Person #2 Nick Name

____

If Q686 + Q707 + Q728 + Q749 + Q770 + Q791 + Q812 + Q833 + Q854 + Q875 is greater than 2 then skip to Q902

If not, then skip to instruction before Q905.

Q902. **Most Contact:** Person #3 First Name

____

Q903. **Most Contact:** Person #3 Last Name

____

Q904. **Most Contact**: Person #3 Nick Name

____

If Q686 + Q707 + Q728 + Q749 + Q770 + Q791 + Q812 + Q833 + Q854 + Q875 is greater than 3 then skip to Q905

If not, then skip to instruction before Q908.

Q905. **Most Contact:** Person #4 First Name

____

Q906. **Most Contact:** Person #4 Last Name

____

Q907. **Most Contact**: Person #4 Nick Name

____

If Q686 + Q707 + Q728 + Q749 + Q770 + Q791 + Q812 + Q833 + Q854 + Q875 is greater than 4 then skip to Q908

If not, then skip to instruction before Q911.

Q908. **Most Contact:** Person #5 First Name

____

Q909. **Most Contact:** Person #5 Last Name

____

Q910. **Most Contact**: Person #5 Nick Name

____

MCL1 = If(Q897 is not equal to 97 and Q897 is not equal to 98, Q897,)

MCN1 = If(Q898 is not equal to 97 and Q898 is not equal to 98, Q898,)

MCL2 = If(Q900 is not equal to 97 and Q900 is not equal to 98, Q900,)

MCN2 = If(Q901 is not equal to 97 and Q901 is not equal to 98, Q901,)

MCL3 = If(Q903 is not equal to 97 and Q903 is not equal to 98, Q903,)

MCN3 = If(Q904 is not equal to 97 and Q904 is not equal to 98, Q904,)

MCL4 = If(Q906 is not equal to 97 and Q906 is not equal to 98, Q906,)

MCN4 = If(Q907 is not equal to 97 and Q907 is not equal to 98, Q907,)

MCL5 = If(Q909 is not equal to 97 and Q909 is not equal to 98, Q909,)

MCN5 = If(Q910 is not equal to 97 and Q910 is not equal to 98, Q910,)

READ: Now we are going to ask some follow-up questions about the people you said you've had the most close contact with in the past 14 days.

If Q686 + Q707 + Q728 + Q749 + Q770 + Q791 + Q812 + Q833 + Q854 + Q875 is greater than 0 and Q896 is not equal to "Refuse to Answer" and Q896 is not equal to "skipped" then skip to Q911

If not, then skip to instruction before Q954.

Q911. Is [Response to Q896] [MCL1] ([MCN1]) male, female, transgender, non-binary or another? (Choose one)

1 Male

2 Female

3 Trans female

4 Trans male

5 Non-binary / genderqueer / gender fluid / agender

6 Another gender identity not listed

8 Refuse to Answer

If Q911 is equal to 6 then skip to Q912

If not, then skip to Q913.

Q912. Please specify the gender identity of [Response to Q896].

____

Q913. Where does [Response to Q896] primarily live? (Choose one)

1 Mexico

2 United States

3 Other

7 Don't Know

8 Refuse to Answer

If Q913 is equal to 3 then skip to Q914

If not, then skip to Q915.

Q914. Please specify where [Response to Q896] primarily lives.

____

Q915. Did you meet [Response to Q896] for the first time in the previous 14 days?

1	Yes
0	No
8	Refuse to Answer

Q916. How many days have you seen [Response to Q896] in the previous 14 days?

__ __	Days
98	Refuse to Answer

Q917. On average, on the days that you saw [Response to Q896], how much time (e.g., hours, minutes) did you spend in close contact with him/her? Interviewer: the max amount of time per day is 24 hours.

__ __	HOURS
__ __	MINUTES
98	Refuse to Answer (Hours)

Q918. When was the last time you had close contact with [Response to Q896] in the past 14 days? Interviewer: Enter how many days ago the participant had close contact for the last time with the person in question. If the last time was today, enter 0.

__ __	DAYS
98	Refuse to Answer (Days)

If Q916 is greater than 1 then skip to Q919

If not, then skip to Q920.

Q919. During that last contact, on how many consecutive days did you see [Response to Q896]?

__ __	days
98	Refuse to Answer

MC1FDAY = If(Q918 is equal to 1, Q918, If(Q918 is not equal to 98 and Q918 is greater than 1, Q918 + Q919, "missing"))

Q920. When you saw [Response to Q896] that last time, what type of contact did you have with them? (Check all that apply) (Check all that apply)

__ Physical, non-sexual [i.e. handshake, hug, skin contact, etc.]

__ Physical, sexual

__ Non-physical [i.e. in-person conversation, meal/drinks, meeting]

__ Refuse to Answer

Q921. When you saw [Response to Q896] that last time, at which of the following places did the close contact occur? (Check all that apply) (Check all that apply)

__ Home/someone's house

__ Work

__ Restaurant or cafe

__ Leisure (i.e. a theater, bar, night club)

__ School/education

__ Exercise or fitness related (i.e., gym, fitness studio)

__ Personal service (i.e. haircut, massage)

__ Religious service (place of worshiip)

__ Community Center/Group

__ Park

__ Street

__ El Bordo/Canal

__ Shooting gallery

__ Other

__ Refuse to Answer

If Q921N is equal to 1 then skip to Q922

If not, then skip to instruction before Q923.

Q922. Please specify the place (i.e., name of the place and cross-streets or neighborhood/colonia) where the last close contact wiith [Response to Q896] occurred.

____

If Q921A is equal to 1 then skip to Q923

If not, then skip to instruction before Q924.

Q923. You said the the last close contact with [Response to Q896] occurred at your house/someone else's house. Where is this house located? Could you please provide the name of the cross-streets at the nearest intersection? Interviewer: Please type in the names of the cross-streets. If the participant cannot provide this information, ask them the name of the neighborhood/colonia and type in that info.

____

If Q921B is equal to 1 then skip to Q924

If not, then skip to instruction before Q925.

Q924. You said that the last close contact with [Response to Q896] occurred at work. Could you please provide the name of the work place and the address? nterviewer: If the work place does not have a name or address, record whatever info the participant can provide for the location (i.e., cross-streets, neighborhood/colonia).

____

If Q921C is equal to 1 then skip to Q925

If not, then skip to instruction before Q926.

Q925. You said that the last close contact with [Response to Q896] occurred at a restaurant/cafe. Could you please provide the name of this place and the cross-streets/neighborhood/colonia where this restaurant is located?

____

If Q921D is equal to 1 then skip to Q926

If not, then skip to instruction before Q927.

Q926. You said that the last close contact with [Response to Q896] occurred at a place of leisure (i.e. a theater, bar, night club). Could you please provide the name of the place and the cross-streets/neighborhood/colonia where this place is located?

— —

If Q921E is equal to 1 then skip to Q927

If not, then skip to instruction before Q928.

Q927. You said that the last close contact with [Response to Q896] occurred at a school/education place. Could you please provide the name of this place and the cross-streets/neighborhood/colonia where this place is located?

— —

If Q921F is equal to 1 then skip to Q928

If not, then skip to instruction before Q929.

Q928. You said that the last close contact with [Response to Q896] occurred at a gym/fitness place. Could you please provide the name of this place and the cross streets/neighborhood/colonia where this place is located?

— —

If Q921G is equal to 1 then skip to Q929

If not, then skip to instruction before Q930.

Q929. You said that the last close contact with [Response to Q896] occurred at a personal service (i.e. haircut, massage) place. Could you please provide the name of this place and the cross streets/neighborhood/colonia where this place is located?

— —

If Q921H is equal to 1 then skip to Q930

If not, then skip to instruction before Q931.

Q930. You said that the last close contact with [Response to Q896] occurred at a religious service place. Could you please provide the name of this place and the cross streets/neighborhood/colonia where this place is located?

— —
— — — — — — — — — —

If Q921I is equal to 1 then skip to Q931

If not, then skip to instruction before Q932.

Q931. You said that the last close contact with [Response to Q896] occurred at a community center/group place. Could you please provide the name of this place and the cross streets/neighborhood/colonia where this place is located?

— —
— — — — — — — — — —

If Q921J is equal to 1 then skip to Q932

If not, then skip to instruction before Q933.

Q932. You said that the last close contact with [Response to Q896] occurred at a park. Could you please provide the name of this place and the cross streets/neighborhood/colonia where this place is located?

— —
— — — — — — — — — —

If Q921K is equal to 1 then skip to Q933

If not, then skip to instruction before Q934.

Q933. You said that the last close contact with [Response to Q896] occurred on the street. Could you please provide the name of thie street and the neighborhood/colonia where this place is located?

— —
— — — — — — — — — —

If Q921M is equal to 1 then skip to Q934

If not, then skip to instruction before Q935.

Q934. You said that the last close contact with [Response to Q896] occurred at a shooting gallery. Which shotting gallery did it occur at? (Choose one)

01	Las Palmitas/Zona Norte
02	Los Pinos/ Zona Norte
03	Las Bombas/ Zona Norte
04	Los Aragón/ Zona Norte
05	La Veinte/ Bordo, Col. Veinte de Noviembre
06	El Azteca/ Cerro Colorado
07	Oxxo, Calle 5ta Zona Norte
08	Bordo/ Los Álamos, Mundo divertido
09	Cañón Guerrero/Maclovio Herrera Colonia Guerrero
10	Col. Guaycura (casa privada)
11	Col. Presidentes (Infonavit edificio)
12	Florido 1era Sección (casa privada)
13	Florido 1era Sección (Bomberos, conecta)
14	Col. Francisco Villa (edificio abandonado)
15	Cañón del Pato/ Colonia Villa
16	Col. Postal (casa privada)
17	Col. del Río (Parte baja, barranco)
18	Sánchez Taboada (Av. Sánchez Taboada y Parque Industrial)
19	La cima (casa abandonado)
20	Otay módulos
21	Other
98	Refuse to Answer

If Q934 is equal to 21 then skip to Q935

If not, then skip to instruction before Q936.

Q935. Please specify the shooting gallery (i.e., name of the place and cross-streets or neighborhood/colonia) where the last close contact wiith [Response to Q896] occurred.

____

If Q916 is greater than 1 then skip to Q936

If not, then skip to instruction before Q954.

Q936. Please think of a different time that you saw [Response to Q896] in the past 14 days, other than the contact that you just described. By 'time' we mean a contact where you saw [Response to Q896] in person and then separated. How many days ago did that take place?

__ __	DAYS
98	Refuse to Answer (Days)

If Q936 is less than MC1FDAY then READ: "This time is more recent than the first day of the last contact. Please make corrections." and skip to Q936.

If Q916 is greater than 1 then skip to Q937

If not, then skip to Q938.

Q937. During this contact, on how many consecutive days did you see [Response to Q896]?

__ __	days
98	Refuse to Answer

Q938. When you saw [Response to Q896] that time, what type of contact did you have with them? (Check all that apply) (Check all that apply)

__	Physical, non-sexual [i.e. handshake, hug, skin contact, etc.]
__	Physical, sexual
__	Non-physical [i.e. in-person conversation, meal/drinks, meeting]
__	Refuse to Answer

Q939. When you saw [Response to Q896] that time, at which of the following places did the close contact occur? (Check all that apply) (Check all that apply)

__ Home/someone's house

__ Work

__ Restaurant or cafe

__ Leisure (i.e. a theater, bar, night club)

__ School/education

__ Exercise or fitness related (i.e., gym, fitness studio)

__ Personal service (i.e. haircut, massage)

__ Religious service (place of worshiip)

__ Community Center/Group

__ Park

__ Street

__ El Bordo/Canal

__ Shooting gallery

__ Other

__ Refuse to Answer

If Q939N is equal to 1 then skip to Q940

If not, then skip to instruction before Q941.

Q940. Please specify the place (i.e., name of the place and cross-streets or neighborhood/colonia) where that close contact wiith [Response to Q896] occurred.

____

If Q939A is equal to 1 then skip to Q941

If not, then skip to instruction before Q942.

Q941. You said the the last close contact with [Response to Q896] occurred at your house/someone else's house. Where is this house located? Could you please provide the name of the cross-streets at the nearest intersection? Interviewer: Please type in the names of the cross-streets. If the participant cannot provide this information, ask them the name of the neighborhood/colonia and type in that info.

____

If Q939B is equal to 1 then skip to Q942

If not, then skip to instruction before Q943.

Q942. You said that the last close contact with [Response to Q896] occurred at work. Could you please provide the name of the work place and the address? Interviewer: If the work place does not have a name or address, record whatever info the participant can provide for the location (i.e., cross-streets, neighborhood/colonia).

____

If Q939C is equal to 1 then skip to Q943

If not, then skip to instruction before Q944.

Q943. You said that the last close contact with [Response to Q896] occurred at a restaurant/cafe. Could you please provide the name of this place and the cross-streets/neighborhood/colonia where this restaurant is located?

____

If Q939D is equal to 1 then skip to Q944

If not, then skip to instruction before Q945.

Q944. You said that the last close contact with [Response to Q896] occurred at a place of leisure (i.e. a theater, bar, night club). Could you please provide the name of the place and the cross-streets/neighborhood/colonia where this place is located?

____

If Q939E is equal to 1 then skip to Q945

If not, then skip to instruction before Q946.

Q945. You said that the last close contact with [Response to Q896] occurred at a school/education place. Could you please provide the name of this place and the cross-streets/neighborhood/colonia where this place is located?

— —

If Q939F is equal to 1 then skip to Q946

If not, then skip to instruction before Q947.

Q946. You said that the last close contact with [Response to Q896] occurred at a gym/fitness place. Could you please provide the name of this place and the cross streets/neighborhood/colonia where this place is located?

— —

If Q939G is equal to 1 then skip to Q947

If not, then skip to instruction before Q948.

Q947. You said that the last close contact with [Response to Q896] occurred at a personal service (i.e. haircut, massage) place. Could you please provide the name of this place and the cross streets/neighborhood/colonia where this place is located?

— —

If Q939H is equal to 1 then skip to Q948

If not, then skip to instruction before Q949.

Q948. You said that the last close contact with [Response to Q896] occurred at a religious service place. Could you please provide the name of this place and the cross streets/neighborhood/colonia where this place is located?

— —

If Q939I is equal to 1 then skip to Q949

If not, then skip to instruction before Q950.

Q949. You said that the last close contact with [Response to Q896] occurred at a community center/group place. Could you please provide the name of this place and the cross streets/neighborhood/colonia where this place is located?

— —

If Q939J is equal to 1 then skip to Q950

If not, then skip to instruction before Q951.

Q950. You said that the last close contact with [Response to Q896] occurred at a park. Could you please provide the name of this place and the cross streets/neighborhood/colonia where this place is located?

— —

If Q939K is equal to 1 then skip to Q951

If not, then skip to instruction before Q952.

Q951. You said that the last close contact with [Response to Q896] occurred on the street. Could you please provide the name of thie street and the neighborhood/colonia where this place is located?

— —

If Q939L is equal to 1 then skip to Q952

If not, then skip to instruction before Q953.

Q952. You said that the last close contact with [Response to Q896] occurred at a shooting gallery. Which shotting gallery did it occur at? (Choose one)

01	Las Palmitas/Zona Norte
02	Los Pinos/ Zona Norte
03	Las Bombas/ Zona Norte
04	Los Aragón/ Zona Norte
05	La Veinte/ Bordo, Col. Veinte de Noviembre
06	El Azteca/ Cerro Colorado
07	Oxxo, Calle 5ta Zona Norte
08	Bordo/ Los Álamos, Mundo divertido
09	Cañón Guerrero/Maclovio Herrera Colonia Guerrero
10	Col. Guaycura (casa privada)
11	Col. Presidentes (Infonavit edificio)
12	Florido 1era Sección (casa privada)
13	Florido 1era Sección (Bomberos, conecta)
14	Col. Francisco Villa (edificio abandonado)
15	Cañón del Pato/ Colonia Villa
16	Col. Postal (casa privada)
17	Col. del Río (Parte baja, barranco)
18	Sánchez Taboada (Av. Sánchez Taboada y Parque Industrial)
19	La cima (casa abandonado)
20	Otay módulos
21	Other
98	Refuse to Answer

If Q952 is equal to 21 then skip to Q953

If not, then skip to instruction before Q954.

Q953. Please specify the shooting gallery (i.e., name of the place and cross-streets or neighborhood/colonia) where the last close contact wiith [Response to Q896] occurred.

____

If Q686 + Q707 + Q728 + Q749 + Q770 + Q791 + Q812 + Q833 + Q854 + Q875 is greater than 1 and Q899 is not equal to "Refuse to Answer" and Q899 is not equal to "skipped" then skip to Q954

If not, then skip to instruction before Q997.

Q954. Is [Response to Q899] [MCL2] ([MCN2]) male, female, transgender, non-binary or another? (Choose one)

1	Male
2	Female
3	Trans female
4	Trans male
5	Non-binary / genderqueer / gender fluid / agender
6	Another gender identity not listed
8	Refuse to Answer

If Q954 is equal to 6 then skip to Q955

If not, then skip to Q956.

Q955. Please specify the gender identity of [Response to Q899].

____

Q956. Where does [Response to Q899] primarily live? (Choose one)

1	Mexico
2	United States
3	Other
7	Don't Know
8	Refuse to Answer

If Q956 is equal to 3 then skip to Q957

If not, then skip to Q958.

Q957. Please specify where [Response to Q899] primarily lives.

— —

Q958. Did you meet [Response to Q899] for the first time in the previous 14 days?

1	Yes
0	No
8	Refuse to Answer

Q959. How many days have you seen [Response to Q899] in the previous 14 days?

— —	Days
98	Refuse to Answer

Q960. On average, on the days that you saw [Response to Q899], how much time (e.g., hours, minutes) did you spend in close contact with him/her? Interviewer: the max amount of time per day is 24 hours.

— —	HOURS
— —	MINUTES
98	Refuse to Answer (Hours)

Q961. When was the last time you had close contact with [Response to Q899] in the past 14 days? Interviewer: Enter how many days ago the participant had close contact for the last time with the person in question. If the last time was today, enter 0.

— —	DAYS
98	Refuse to Answer (Days)

If Q959 is greater than 1 then skip to Q962

If not, then skip to Q963.

Q962. During that last contact, on how many consecutive days did you see [Response to Q899]?

__ __	days
98	Refuse to Answer

MC2FDAY = If(Q961 is equal to 1, Q961, If(Q961 is not equal to 98 and Q961 is greater than 1, Q961 + Q962, "missing"))

Q963. When you saw [Response to Q899] that last time, what type of contact did you have with them? (Check all that apply) (Check all that apply)

__	Physical, non-sexual [i.e. handshake, hug, skin contact, etc.]
__	Physical, sexual
__	Non-physical [i.e. in-person conversation, meal/drinks, meeting]
__	Refuse to Answer

Q964. When you saw [Response to Q899] that last time, at which of the following places did the close contact occur? (Check all that apply) (Check all that apply)

__ Home/someone's house

__ Work

__ Restaurant or cafe

__ Leisure (i.e. a theater, bar, night club)

__ School/education

__ Exercise or fitness related (i.e., gym, fitness studio)

__ Personal service (i.e. haircut, massage)

__ Religious service (place of worshiip)

__ Community Center/Group

__ Park

__ Street

__ El Bordo/Canal

__ Shooting gallery

__ Other

__ Refuse to Answer

If Q964N is equal to 1 then skip to Q965

If not, then skip to instruction before Q966.

Q965. Please specify the place (i.e., name of the place and cross-streets or neighborhood/colonia) where the last close contact wiith [Response to Q899] occurred.

____

If Q964A is equal to 1 then skip to Q966

If not, then skip to instruction before Q967.

Q966. You said the the last close contact with [Response to Q899] occurred at your house/someone else's house. Where is this house located? Could you please provide the name of the cross-streets at the nearest intersection? Interviewer: Please type in the names of the cross-streets. If the participant cannot provide this information, ask them the name of the neighborhood/colonia and type in that info.

— —

If Q964B is equal to 1 then skip to Q967

If not, then skip to instruction before Q968.

Q967. You said that the last close contact with [Response to Q899] occurred at work. Could you please provide the name of the work place and the address? nterviewer: If the work place does not have a name or address, record whatever info the participant can provide for the location (i.e., cross-streets, neighborhood/colonia).

— —

If Q964C is equal to 1 then skip to Q968

If not, then skip to instruction before Q969.

Q968. You said that the last close contact with [Response to Q899] occurred at a restaurant/cafe. Could you please provide the name of this place and the cross-streets/neighborhood/colonia where this restaurant is located?

— —

If Q964D is equal to 1 then skip to Q969

If not, then skip to instruction before Q970.

Q969. You said that the last close contact with [Response to Q899] occurred at a place of leisure (i.e. a theater, bar, night club). Could you please provide the name of the place and the cross-streets/neighborhood/colonia where this place is located?

— —

If Q964E is equal to 1 then skip to Q970

If not, then skip to instruction before Q971.

Q970. You said that the last close contact with [Response to Q896] occurred at a school/education place. Could you please provide the name of this place and the cross-streets/neighborhood/colonia where this place is located?

— —

If Q964F is equal to 1 then skip to Q971

If not, then skip to instruction before Q972.

Q971. You said that the last close contact with [Response to Q899] occurred at a gym/fitness place. Could you please provide the name of this place and the cross streets/neighborhood/colonia where this place is located?

— —

If Q964G is equal to 1 then skip to Q972

If not, then skip to instruction before Q973.

Q972. You said that the last close contact with [Response to Q899] occurred at a personal service (i.e. haircut, massage) place. Could you please provide the name of this place and the cross streets/neighborhood/colonia where this place is located?

— —

If Q964H is equal to 1 then skip to Q973

If not, then skip to instruction before Q974.

Q973. You said that the last close contact with [Response to Q899] occurred at a religious service place. Could you please provide the name of this place and the cross streets/neighborhood/colonia where this place is located?

— —

If Q964I is equal to 1 then skip to Q974

If not, then skip to instruction before Q975.

Q974. You said that the last close contact with [Response to Q899] occurred at a community center/group place. Could you please provide the name of this place and the cross streets/neighborhood/colonia where this place is located?

— —

If Q964J is equal to 1 then skip to Q975

If not, then skip to instruction before Q976.

Q975. You said that the last close contact with [Response to Q899] occurred at a park. Could you please provide the name of this place and the cross streets/neighborhood/colonia where this place is located?

— —

If Q964K is equal to 1 then skip to Q976

If not, then skip to instruction before Q977.

Q976. You said that the last close contact with [Response to Q899] occurred on the street. Could you please provide the name of thie street and the neighborhood/colonia where this place is located?

— —

If Q964M is equal to 1 then skip to Q977

If not, then skip to instruction before Q978.

Q977. You said that the last close contact with [Response to Q899] occurred at a shooting gallery. Which shotting gallery did it occur at? (Choose one)

01	Las Palmitas/Zona Norte
02	Los Pinos/ Zona Norte
03	Las Bombas/ Zona Norte
04	Los Aragón/ Zona Norte
05	La Veinte/ Bordo, Col. Veinte de Noviembre
06	El Azteca/ Cerro Colorado
07	Oxxo, Calle 5ta Zona Norte
08	Bordo/ Los Álamos, Mundo divertido
09	Cañón Guerrero/Maclovio Herrera Colonia Guerrero
10	Col. Guaycura (casa privada)
11	Col. Presidentes (Infonavit edificio)
12	Florido 1era Sección (casa privada)
13	Florido 1era Sección (Bomberos, conecta)
14	Col. Francisco Villa (edificio abandonado)
15	Cañón del Pato/ Colonia Villa
16	Col. Postal (casa privada)
17	Col. del Río (Parte baja, barranco)
18	Sánchez Taboada (Av. Sánchez Taboada y Parque Industrial)
19	La cima (casa abandonado)
20	Otay módulos
21	Other
98	Refuse to Answer

If Q977 is equal to 21 then skip to Q978

If not, then skip to instruction before Q979.

Q978. Please specify the shooting gallery (i.e., name of the place and cross-streets or neighborhood/colonia) where the last close contact wiith [Response to Q899] occurred.

__ __

If Q959 is greater than 1 then skip to Q979

If not, then skip to instruction before Q997.

Q979. Please think of a different time that you saw [Response to Q899] in the past 14 days, other than the contact that you just described. By 'time' we mean a contact where you saw [Response to Q899] in person and then separated. How many days ago did that take place?

__ __	DAYS
98	Refuse to Answer (Days)

If Q979 is less than MC2FDAY then READ: "This time is more recent than the first day of the last contact. Please make corrections." and skip to Q979.

If Q959 is greater than 1 then skip to Q980

If not, then skip to Q981.

Q980. During this contact, on how many consecutive days did you see [Response to Q899]?

__ __	days
98	Refuse to Answer

Q981. When you saw [Response to Q899] that time, what type of contact did you have with them? (Check all that apply) (Check all that apply)

__	Physical, non-sexual [i.e. handshake, hug, skin contact, etc.]
__	Physical, sexual
__	Non-physical [i.e. in-person conversation, meal/drinks, meeting]
__	Refuse to Answer

Q982. When you saw [Response to Q899] that time, at which of the following places did the close contact occur? (Check all that apply) (Check all that apply)

__ Home/someone's house

__ Work

__ Restaurant or cafe

__ Leisure (i.e. a theater, bar, night club)

__ School/education

__ Exercise or fitness related (i.e., gym, fitness studio)

__ Personal service (i.e. haircut, massage)

__ Religious service (place of worshiip)

__ Community Center/Group

__ Park

__ Street

__ El Bordo/Canal

__ Shooting gallery

__ Other

__ Refuse to Answer

If Q982N is equal to 1 then skip to Q983

If not, then skip to instruction before Q984.

Q983. Please specify the place (i.e., name of the place and cross-streets or neighborhood/colonia) where that close contact wiith [Response to Q899] occurred.

____

If Q982A is equal to 1 then skip to Q984

If not, then skip to instruction before Q985.

Q984. You said the the last close contact with [Response to Q899] occurred at your house/someone else's house. Where is this house located? Could you please provide the name of the cross-streets at the nearest intersection? Interviewer: Please type in the names of the cross-streets. If the participant cannot provide this information, ask them the name of the neighborhood/colonia and type in that info.

— —

If Q982B is equal to 1 then skip to Q985

If not, then skip to instruction before Q986.

Q985. You said that the last close contact with [Response to Q899] occurred at work. Could you please provide the name of the work place and the address? nterviewer: If the work place does not have a name or address, record whatever info the participant can provide for the location (i.e., cross-streets, neighborhood/colonia).

— —

If Q982C is equal to 1 then skip to Q986

If not, then skip to instruction before Q987.

Q986. You said that the last close contact with [Response to Q899] occurred at a restaurant/cafe. Could you please provide the name of this place and the cross-streets/neighborhood/colonia where this restaurant is located?

— —

If Q982D is equal to 1 then skip to Q987

If not, then skip to instruction before Q988.

Q987. You said that the last close contact with [Response to Q899] occurred at a place of leisure (i.e. a theater, bar, night club). Could you please provide the name of the place and the cross-streets/neighborhood/colonia where this place is located?

— —

If Q982E is equal to 1 then skip to Q988

If not, then skip to instruction before Q989.

Q988. You said that the last close contact with [Response to Q899] occurred at a school/education place. Could you please provide the name of this place and the cross-streets/neighborhood/colonia where this place is located?

— —

If Q982F is equal to 1 then skip to Q989

If not, then skip to instruction before Q990.

Q989. You said that the last close contact with [Response to Q899] occurred at a gym/fitness place. Could you please provide the name of this place and the cross streets/neighborhood/colonia where this place is located?

— —

If Q982G is equal to 1 then skip to Q990

If not, then skip to instruction before Q991.

Q990. You said that the last close contact with [Response to Q899] occurred at a personal service (i.e. haircut, massage) place. Could you please provide the name of this place and the cross streets/neighborhood/colonia where this place is located?

— —

If Q982H is equal to 1 then skip to Q991

If not, then skip to instruction before Q992.

Q991. You said that the last close contact with [Response to Q899] occurred at a religious service place. Could you please provide the name of this place and the cross streets/neighborhood/colonia where this place is located?

— —

If Q982I is equal to 1 then skip to Q992

If not, then skip to instruction before Q993.

Q992. You said that the last close contact with [Response to Q899] occurred at a community center/group place. Could you please provide the name of this place and the cross streets/neighborhood/colonia where this place is located?

— —

If Q982J is equal to 1 then skip to Q993

If not, then skip to instruction before Q994.

Q993. You said that the last close contact with [Response to Q899] occurred at a park. Could you please provide the name of this place and the cross streets/neighborhood/colonia where this place is located?

— —

If Q982K is equal to 1 then skip to Q994

If not, then skip to instruction before Q995.

Q994. You said that the last close contact with [Response to Q899] occurred on the street. Could you please provide the name of thie street and the neighborhood/colonia where this place is located?

— —

If Q982M is equal to 1 then skip to Q995

If not, then skip to instruction before Q996.

Q995. You said that the last close contact with [Response to Q899] occurred at a shooting gallery. Which shotting gallery did it occur at? (Choose one)

01	Las Palmitas/Zona Norte
02	Los Pinos/ Zona Norte
03	Las Bombas/ Zona Norte
04	Los Aragón/ Zona Norte
05	La Veinte/ Bordo, Col. Veinte de Noviembre
06	El Azteca/ Cerro Colorado
07	Oxxo, Calle 5ta Zona Norte
08	Bordo/ Los Álamos, Mundo divertido
09	Cañón Guerrero/Maclovio Herrera Colonia Guerrero
10	Col. Guaycura (casa privada)
11	Col. Presidentes (Infonavit edificio)
12	Florido 1era Sección (casa privada)
13	Florido 1era Sección (Bomberos, conecta)
14	Col. Francisco Villa (edificio abandonado)
15	Cañón del Pato/ Colonia Villa
16	Col. Postal (casa privada)
17	Col. del Río (Parte baja, barranco)
18	Sánchez Taboada (Av. Sánchez Taboada y Parque Industrial)
19	La cima (casa abandonado)
20	Otay módulos
21	Other
98	Refuse to Answer

If Q995 is equal to 21 then skip to Q996

If not, then skip to instruction before Q997.

Q996. Please specify the shooting gallery (i.e., name of the place and cross-streets or neighborhood/colonia) where the last close contact wiith [Response to Q899] occurred.

____

If Q686 + Q707 + Q728 + Q749 + Q770 + Q791 + Q812 + Q833 + Q854 + Q875 is greater than 2 and Q902 is not equal to "Refuse to Answer" and Q902 is not equal to "skipped" then skip to Q997

If not, then skip to instruction before Q1040.

Q997. Is [Response to Q902] [MCL3] ([MCN3]) male, female, transgender, non-binary or another? (Choose one)

1	Male
2	Female
3	Trans female
4	Trans male
5	Non-binary / genderqueer / gender fluid / agender
6	Another gender identity not listed
8	Refuse to Answer

If Q997 is equal to 6 then skip to Q998

If not, then skip to Q999.

Q998. Please specify the gender identity of [Response to Q902].

____

Q999. Where does [Response to Q902] primarily live? (Choose one)

1	Mexico
2	United States
3	Other
7	Don't Know
8	Refuse to Answer

If Q999 is equal to 3 then skip to Q1000

If not, then skip to Q1001.

Q1000. Please specify where [Response to Q902] primarily lives.

____

Q1001. Did you meet [Response to Q902] for the first time in the previous 14 days?

1	Yes
0	No
8	Refuse to Answer

Q1002. How many days have you seen [Response to Q902] in the previous 14 days?

__ __	Days
98	Refuse to Answer

Q1003. On average, on the days that you saw [Response to Q902], how much time (e.g., hours, minutes) did you spend in close contact with him/her? Interviewer: the max amount of time per day is 24 hours.

__ __	HOURS
__ __	MINUTES
98	Refuse to Answer (Hours)

Q1004. When was the last time you had close contact with [Response to Q902] in the past 14 days? Interviewer: Enter how many days ago the participant had close contact for the last time with the person in question. If the last time was today, enter 0.

__ __	DAYS
98	Refuse to Answer (Days)

If Q1002 is greater than 1 then skip to Q1005

If not, then skip to Q1006.

Q1005. During that last contact, on how many consecutive days did you see [Response to Q902]?

__ __	days
98	Refuse to Answer

MC3FDAY = If(Q1004 is equal to 1, Q1004, If(Q1004 is not equal to 98 and Q1004 is greater than 1, Q1004 + Q1005, "missing"))

Q1006. When you saw [Response to Q902] that last time, what type of contact did you have with them? (Check all that apply) (Check all that apply)

__	Physical, non-sexual [i.e. handshake, hug, skin contact, etc.]
__	Physical, sexual
__	Non-physical [i.e. in-person conversation, meal/drinks, meeting]
__	Refuse to Answer

Q1007. When you saw [Response to Q902] that last time, at which of the following places did the close contact occur? (Check all that apply) (Check all that apply)

__ Home/someone's house

__ Work

__ Restaurant or cafe

__ Leisure (i.e. a theater, bar, night club)

__ School/education

__ Exercise or fitness related (i.e., gym, fitness studio)

__ Personal service (i.e. haircut, massage)

__ Religious service (place of worshiip)

__ Community Center/Group

__ Park

__ Street

__ El Bordo/Canal

__ Shooting gallery

__ Other

__ Refuse to Answer

If Q1007N is equal to 1 then skip to Q1008

If not, then skip to instruction before Q1009.

Q1008. Please specify the place (i.e., name of the place and cross-streets or neighborhood/colonia) where the last close contact wiith [Response to Q902] occurred.

____

If Q1007A is equal to 1 then skip to Q1009

If not, then skip to instruction before Q1010.

Q1009. You said the the last close contact with [Response to Q902] occurred at your house/someone else's house. Where is this house located? Could you please provide the name of the cross-streets at the nearest intersection? Interviewer: Please type in the names of the cross-streets. If the participant cannot provide this information, ask them the name of the neighborhood/colonia and type in that info.

____

If Q1007B is equal to 1 then skip to Q1010

If not, then skip to instruction before Q1011.

Q1010. You said that the last close contact with [Response to Q902] occurred at work. Could you please provide the name of the work place and the address? nterviewer: If the work place does not have a name or address, record whatever info the participant can provide for the location (i.e., cross-streets, neighborhood/colonia).

____

If Q1007C is equal to 1 then skip to Q1011

If not, then skip to instruction before Q1012.

Q1011. You said that the last close contact with [Response to Q902] occurred at a restaurant/cafe. Could you please provide the name of this place and the cross-streets/neighborhood/colonia where this restaurant is located?

____

If Q1007D is equal to 1 then skip to Q1012

If not, then skip to instruction before Q1013.

Q1012. You said that the last close contact with [Response to Q902] occurred at a place of leisure (i.e. a theater, bar, night club). Could you please provide the name of the place and the cross-streets/neighborhood/colonia where this place is located?

____

If Q1007E is equal to 1 then skip to Q1013

If not, then skip to instruction before Q1014.

Q1013. You said that the last close contact with [Response to Q902] occurred at a school/education place. Could you please provide the name of this place and the cross-streets/neighborhood/colonia where this place is located?

— —
— — — — — — — — — — — —

If Q1007F is equal to 1 then skip to Q1014

If not, then skip to instruction before Q1015.

Q1014. You said that the last close contact with [Response to Q902] occurred at a gym/fitness place. Could you please provide the name of this place and the cross streets/neighborhood/colonia where this place is located?

— —
— — — — — — — — — — — —

If Q1007G is equal to 1 then skip to Q1015

If not, then skip to instruction before Q1016.

Q1015. You said that the last close contact with [Response to Q902] occurred at a personal service (i.e. haircut, massage) place. Could you please provide the name of this place and the cross streets/neighborhood/colonia where this place is located?

— —
— — — — — — — — — — — —

If Q1007H is equal to 1 then skip to Q1016

If not, then skip to instruction before Q1017.

Q1016. You said that the last close contact with [Response to Q902] occurred at a religious service place. Could you please provide the name of this place and the cross streets/neighborhood/colonia where this place is located?

— —
— — — — — — — — — — — —

If Q1007I is equal to 1 then skip to Q1017

If not, then skip to instruction before Q1018.

Q1017. You said that the last close contact with [Response to Q902] occurred at a community center/group place. Could you please provide the name of this place and the cross streets/neighborhood/colonia where this place is located?

— —

If Q1007J is equal to 1 then skip to Q1018

If not, then skip to instruction before Q1019.

Q1018. You said that the last close contact with [Response to Q902] occurred at a park. Could you please provide the name of this place and the cross streets/neighborhood/colonia where this place is located?

— —

If Q1007K is equal to 1 then skip to Q1019

If not, then skip to instruction before Q1020.

Q1019. You said that the last close contact with [Response to Q902] occurred on the street. Could you please provide the name of thie street and the neighborhood/colonia where this place is located?

— —

If Q1007M is equal to 1 then skip to Q1020

If not, then skip to instruction before Q1021.

Q1020. You said that the last close contact with [Response to Q902] occurred at a shooting gallery. Which shotting gallery did it occur at? (Choose one)

01	Las Palmitas/Zona Norte
02	Los Pinos/ Zona Norte
03	Las Bombas/ Zona Norte
04	Los Aragón/ Zona Norte
05	La Veinte/ Bordo, Col. Veinte de Noviembre
06	El Azteca/ Cerro Colorado
07	Oxxo, Calle 5ta Zona Norte
08	Bordo/ Los Álamos, Mundo divertido
09	Cañón Guerrero/Maclovio Herrera Colonia Guerrero
10	Col. Guaycura (casa privada)
11	Col. Presidentes (Infonavit edificio)
12	Florido 1era Sección (casa privada)
13	Florido 1era Sección (Bomberos, conecta)
14	Col. Francisco Villa (edificio abandonado)
15	Cañón del Pato/ Colonia Villa
16	Col. Postal (casa privada)
17	Col. del Río (Parte baja, barranco)
18	Sánchez Taboada (Av. Sánchez Taboada y Parque Industrial)
19	La cima (casa abandonado)
20	Otay módulos
21	Other
98	Refuse to Answer

If Q1020 is equal to 21 then skip to Q1021

If not, then skip to instruction before Q1022.

Q1021. Please specify the shooting gallery (i.e., name of the place and cross-streets or neighborhood/colonia) where the last close contact wiith [Response to Q902] occurred.

____

If Q1002 is greater than 1 then skip to Q1022

If not, then skip to instruction before Q1040.

Q1022. Please think of a different time that you saw [Response to Q902] in the past 14 days, other than the contact that you just described. By 'time' we mean a contact where you saw [Response to Q902] in person and then separated. How many days ago did that take place?

__ __ DAYS

98 Refuse to Answer (Days)

If Q1022 is less than MC3FDAY then READ: "This time is more recent than the first day of the last contact. Please make corrections." and skip to Q1022.

If Q1002 is greater than 1 then skip to Q1023

If not, then skip to Q1024.

Q1023. During this contact, on how many consecutive days did you see [Response to Q902]?

__ __ days

98 Refuse to Answer

Q1024. When you saw [Response to Q902] that time, what type of contact did you have with them? (Check all that apply) (Check all that apply)

__ Physical, non-sexual [i.e. handshake, hug, skin contact, etc.]

__ Physical, sexual

__ Non-physical [i.e. in-person conversation, meal/drinks, meeting]

__ Refuse to Answer

Q1025. When you saw [Response to Q902] that time, at which of the following places did the close contact occur? (Check all that apply) (Check all that apply)

__ Home/someone's house

__ Work

__ Restaurant or cafe

__ Leisure (i.e. a theater, bar, night club)

__ School/education

__ Exercise or fitness related (i.e., gym, fitness studio)

__ Personal service (i.e. haircut, massage)

__ Religious service (place of worshiip)

__ Community Center/Group

__ Park

__ Street

__ El Bordo/Canal

__ Shooting gallery

__ Other

__ Refuse to Answer

If Q1025N is equal to 1 then skip to Q1026

If not, then skip to instruction before Q1027.

Q1026. Please specify the place (i.e., name of the place and cross-streets or neighborhood/colonia) where that close contact wiith [Response to Q902] occurred.

____

If Q1025A is equal to 1 then skip to Q1027

If not, then skip to instruction before Q1028.

Q1027. You said the the last close contact with [Response to Q902] occurred at your house/someone else's house. Where is this house located? Could you please provide the name of the cross-streets at the nearest intersection? Interviewer: Please type in the names of the cross-streets. If the participant cannot provide this information, ask them the name of the neighborhood/colonia and type in that info.

— —

If Q1025B is equal to 1 then skip to Q1028

If not, then skip to instruction before Q1029.

Q1028. You said that the last close contact with [Response to Q902] occurred at work. Could you please provide the name of the work place and the address? nterviewer: If the work place does not have a name or address, record whatever info the participant can provide for the location (i.e., cross-streets, neighborhood/colonia).

— —

If Q1025C is equal to 1 then skip to Q1029

If not, then skip to instruction before Q1030.

Q1029. You said that the last close contact with [Response to Q902] occurred at a restaurant/cafe. Could you please provide the name of this place and the cross-streets/neighborhood/colonia where this restaurant is located?

— —

If Q1025D is equal to 1 then skip to Q1030

If not, then skip to instruction before Q1031.

Q1030. You said that the last close contact with [Response to Q902] occurred at a place of leisure (i.e. a theater, bar, night club). Could you please provide the name of the place and the cross-streets/neighborhood/colonia where this place is located?

— —

If Q1025E is equal to 1 then skip to Q1031

If not, then skip to instruction before Q1032.

Q1031. You said that the last close contact with [Response to Q902] occurred at a school/education place. Could you please provide the name of this place and the cross-streets/neighborhood/colonia where this place is located?

— —

If Q1025F is equal to 1 then skip to Q1032

If not, then skip to instruction before Q1033.

Q1032. You said that the last close contact with [Response to Q902] occurred at a gym/fitness place. Could you please provide the name of this place and the cross streets/neighborhood/colonia where this place is located?

— —

If Q1025G is equal to 1 then skip to Q1033

If not, then skip to instruction before Q1034.

Q1033. You said that the last close contact with [Response to Q902] occurred at a personal service (i.e. haircut, massage) place. Could you please provide the name of this place and the cross streets/neighborhood/colonia where this place is located?

— —

If Q1025H is equal to 1 then skip to Q1034

If not, then skip to instruction before Q1035.

Q1034. You said that the last close contact with [Response to Q902] occurred at a religious service place. Could you please provide the name of this place and the cross streets/neighborhood/colonia where this place is located?

— —

If Q1025I is equal to 1 then skip to Q1035

If not, then skip to instruction before Q1036.

Q1035. You said that the last close contact with [Response to Q902] occurred at a community center/group place. Could you please provide the name of this place and the cross streets/neighborhood/colonia where this place is located?

— —

If Q1025J is equal to 1 then skip to Q1036

If not, then skip to instruction before Q1037.

Q1036. You said that the last close contact with [Response to Q902] occurred at a park. Could you please provide the name of this place and the cross streets/neighborhood/colonia where this place is located?

— —

If Q1025K is equal to 1 then skip to Q1037

If not, then skip to instruction before Q1038.

Q1037. You said that the last close contact with [Response to Q902] occurred on the street. Could you please provide the name of thie street and the neighborhood/colonia where this place is located?

— —

If Q1025M is equal to 1 then skip to Q1038

If not, then skip to instruction before Q1039.

Q1038. You said that the last close contact with [Response to Q902] occurred at a shooting gallery. Which shotting gallery did it occur at? (Choose one)

01	Las Palmitas/Zona Norte
02	Los Pinos/ Zona Norte
03	Las Bombas/ Zona Norte
04	Los Aragón/ Zona Norte
05	La Veinte/ Bordo, Col. Veinte de Noviembre
06	El Azteca/ Cerro Colorado
07	Oxxo, Calle 5ta Zona Norte
08	Bordo/ Los Álamos, Mundo divertido
09	Cañón Guerrero/Maclovio Herrera Colonia Guerrero
10	Col. Guaycura (casa privada)
11	Col. Presidentes (Infonavit edificio)
12	Florido 1era Sección (casa privada)
13	Florido 1era Sección (Bomberos, conecta)
14	Col. Francisco Villa (edificio abandonado)
15	Cañón del Pato/ Colonia Villa
16	Col. Postal (casa privada)
17	Col. del Río (Parte baja, barranco)
18	Sánchez Taboada (Av. Sánchez Taboada y Parque Industrial)
19	La cima (casa abandonado)
20	Otay módulos
21	Other
98	Refuse to Answer

If Q1038 is equal to 21 then skip to Q1039

If not, then skip to instruction before Q1040.

Q1039. Please specify the shooting gallery (i.e., name of the place and cross-streets or neighborhood/colonia) where the last close contact wiith [Response to Q902] occurred.

____

If Q686 + Q707 + Q728 + Q749 + Q770 + Q791 + Q812 + Q833 + Q854 + Q875 is greater than 3 and Q905 is not equal to "Refuse to Answer" and Q905 is not equal to "skipped" then skip to Q1040

If not, then skip to instruction before Q1083.

Q1040. Is [Response to Q905] [Response to Q906] ([Response to Q907]) male, female, transgender, non-binary or another? (Choose one)

1	Male
2	Female
3	Trans female
4	Trans male
5	Non-binary / genderqueer / gender fluid / agender
6	Another gender identity not listed
8	Refuse to Answer

If Q1040 is equal to 6 then skip to Q1041

If not, then skip to Q1042.

Q1041. Please specify the gender identity of [Response to Q905].

____

Q1042. Where does [Response to Q905] primarily live? (Choose one)

1	Mexico
2	United States
3	Other
7	Don't Know
8	Refuse to Answer

If Q1042 is equal to 3 then skip to Q1043

If not, then skip to Q1044.

Q1043. Please specify where [Response to Q905] primarily lives.

____

Q1044. Did you meet [Response to Q905] for the first time in the previous 14 days?

1	Yes
0	No
8	Refuse to Answer

Q1045. How many days have you seen [Response to Q905] in the previous 14 days?

__ __	Days
98	Refuse to Answer

Q1046. On average, on the days that you saw [Response to Q905], how much time (e.g., hours, minutes) did you spend in close contact with him/her? Interviewer: the max amount of time per day is 24 hours.

__ __	HOURS
__ __	MINUTES
98	Refuse to Answer (Hours)

Q1047. When was the last time you had close contact with [Response to Q905] in the past 14 days? Interviewer: Enter how many days ago the participant had close contact for the last time with the person in question. If the last time was today, enter 0.

__ __	DAYS
98	Refuse to Answer (Days)

If Q1045 is greater than 1 then skip to Q1048

If not, then skip to Q1049.

Q1048. During that last contact, on how many consecutive days did you see [Response to Q905]?

__ __ days

98 Refuse to Answer

MC4FDAY = If(Q1047 is equal to 1, Q1047, If(Q1047 is not equal to 98 and Q1047 is greater than 1, Q1047 + Q1048, "missing"))

Q1049. When you saw [Response to Q905] that last time, what type of contact did you have with them? (Check all that apply) (Check all that apply)

__ Physical, non-sexual [i.e. handshake, hug, skin contact, etc.]

__ Physical, sexual

__ Non-physical [i.e. in-person conversation, meal/drinks, meeting]

__ Refuse to Answer

Q1050. When you saw [Response to Q905] that last time, at which of the following places did the close contact occur? (Check all that apply) (Check all that apply)

__ Home/someone's house

__ Work

__ Restaurant or cafe

__ Leisure (i.e. a theater, bar, night club)

__ School/education

__ Exercise or fitness related (i.e., gym, fitness studio)

__ Personal service (i.e. haircut, massage)

__ Religious service (place of worshiip)

__ Community Center/Group

__ Park

__ Street

__ El Bordo/Canal

__ Shooting gallery

__ Other

__ Refuse to Answer

If Q1050N is equal to 1 then skip to Q1051

If not, then skip to instruction before Q1052.

Q1051. Please specify the place (i.e., name of the place and cross-streets or neighborhood/colonia) where the last close contact wiith [Response to Q905] occurred.

____

If Q1050A is equal to 1 then skip to Q1052

If not, then skip to instruction before Q1053.

Q1052. You said the the last close contact with [Response to Q905] occurred at your house/someone else's house. Where is this house located? Could you please provide the name of the cross-streets at the nearest intersection? Interviewer: Please type in the names of the cross-streets. If the participant cannot provide this information, ask them the name of the neighborhood/colonia and type in that info.

— —

If Q1050B is equal to 1 then skip to Q1053

If not, then skip to instruction before Q1054.

Q1053. You said that the last close contact with [Response to Q905] occurred at work. Could you please provide the name of the work place and the address? Interviewer: If the work place does not have a name or address, record whatever info the participant can provide for the location (i.e., cross-streets, neighborhood/colonia).

— —

If Q1050C is equal to 1 then skip to Q1054

If not, then skip to instruction before Q1055.

Q1054. You said that the last close contact with [Response to Q905] occurred at a restaurant/cafe. Could you please provide the name of this place and the cross-streets/neighborhood/colonia where this restaurant is located?

— —

If Q1050D is equal to 1 then skip to Q1055

If not, then skip to instruction before Q1056.

Q1055. You said that the last close contact with [Response to Q905] occurred at a place of leisure (i.e. a theater, bar, night club). Could you please provide the name of the place and the cross-streets/neighborhood/colonia where this place is located?

— —

If Q1050E is equal to 1 then skip to Q1056

If not, then skip to instruction before Q1057.

Q1056. You said that the last close contact with [Response to Q905] occurred at a school/education place. Could you please provide the name of this place and the cross-streets/neighborhood/colonia where this place is located?

— —

If Q1050F is equal to 1 then skip to Q1057

If not, then skip to instruction before Q1058.

Q1057. You said that the last close contact with [Response to Q905] occurred at a gym/fitness place. Could you please provide the name of this place and the cross streets/neighborhood/colonia where this place is located?

— —

If Q1050G is equal to 1 then skip to Q1058

If not, then skip to instruction before Q1059.

Q1058. You said that the last close contact with [Response to Q905] occurred at a personal service (i.e. haircut, massage) place. Could you please provide the name of this place and the cross streets/neighborhood/colonia where this place is located?

— —

If Q1050H is equal to 1 then skip to Q1059

If not, then skip to instruction before Q1060.

Q1059. You said that the last close contact with [Response to Q905] occurred at a religious service place. Could you please provide the name of this place and the cross streets/neighborhood/colonia where this place is located?

— —

If Q1050I is equal to 1 then skip to Q1060

If not, then skip to instruction before Q1061.

Q1060. You said that the last close contact with [Response to Q905] occurred at a community center/group place. Could you please provide the name of this place and the cross streets/neighborhood/colonia where this place is located?

— —

If Q1050J is equal to 1 then skip to Q1061

If not, then skip to instruction before Q1062.

Q1061. You said that the last close contact with [Response to Q905] occurred at a park. Could you please provide the name of this place and the cross streets/neighborhood/colonia where this place is located?

— —

If Q1050K is equal to 1 then skip to Q1062

If not, then skip to instruction before Q1063.

Q1062. You said that the last close contact with [Response to Q905] occurred on the street. Could you please provide the name of thie street and the neighborhood/colonia where this place is located?

— —

If Q1050M is equal to 1 then skip to Q1063

If not, then skip to instruction before Q1064.

Q1063. You said that the last close contact with [Response to Q905] occurred at a shooting gallery. Which shotting gallery did it occur at? (Choose one)

01	Las Palmitas/Zona Norte
02	Los Pinos/ Zona Norte
03	Las Bombas/ Zona Norte
04	Los Aragón/ Zona Norte
05	La Veinte/ Bordo, Col. Veinte de Noviembre
06	El Azteca/ Cerro Colorado
07	Oxxo, Calle 5ta Zona Norte
08	Bordo/ Los Álamos, Mundo divertido
09	Cañón Guerrero/Maclovio Herrera Colonia Guerrero
10	Col. Guaycura (casa privada)
11	Col. Presidentes (Infonavit edificio)
12	Florido 1era Sección (casa privada)
13	Florido 1era Sección (Bomberos, conecta)
14	Col. Francisco Villa (edificio abandonado)
15	Cañón del Pato/ Colonia Villa
16	Col. Postal (casa privada)
17	Col. del Río (Parte baja, barranco)
18	Sánchez Taboada (Av. Sánchez Taboada y Parque Industrial)
19	La cima (casa abandonado)
20	Otay módulos
21	Other
98	Refuse to Answer

If Q1063 is equal to 21 then skip to Q1064

If not, then skip to instruction before Q1065.

Q1064. Please specify the shooting gallery (i.e., name of the place and cross-streets or neighborhood/colonia) where the last close contact wiith [Response to Q905] occurred.

____

If Q1045 is greater than 1 then skip to Q1065

If not, then skip to instruction before Q1083.

Q1065. Please think of a different time that you saw [Response to Q905] in the past 14 days, other than the contact that you just described. By 'time' we mean a contact where you saw [Response to Q905] in person and then separated. How many days ago did that take place?

__ __	DAYS
98	Refuse to Answer (Days)

If Q1065 is less than MC4FDAY then READ: "This time is more recent than the first day of the last contact. Please make corrections." and skip to Q1065.

If Q1045 is greater than 1 then skip to Q1066

If not, then skip to Q1067.

Q1066. During this contact, on how many consecutive days did you see [Response to Q905]?

__ __	days
98	Refuse to Answer

Q1067. When you saw [Response to Q905] that time, what type of contact did you have with them? (Check all that apply) (Check all that apply)

__	Physical, non-sexual [i.e. handshake, hug, skin contact, etc.]
__	Physical, sexual
__	Non-physical [i.e. in-person conversation, meal/drinks, meeting]
__	Refuse to Answer

Q1068. When you saw [Response to Q905] that time, at which of the following places did the close contact occur? (Check all that apply) (Check all that apply)

__ Home/someone's house

__ Work

__ Restaurant or cafe

__ Leisure (i.e. a theater, bar, night club)

__ School/education

__ Exercise or fitness related (i.e., gym, fitness studio)

__ Personal service (i.e. haircut, massage)

__ Religious service (place of worshiip)

__ Community Center/Group

__ Park

__ Street

__ El Bordo/Canal

__ Shooting gallery

__ Other

__ Refuse to Answer

If Q1068N is equal to 1 then skip to Q1069

If not, then skip to instruction before Q1070.

Q1069. Please specify the place (i.e., name of the place and cross-streets or neighborhood/colonia) where that close contact wiith [Response to Q905] occurred.

____

If Q1068A is equal to 1 then skip to Q1070

If not, then skip to instruction before Q1071.

Q1070. You said the the last close contact with [Response to Q905] occurred at your house/someone else's house. Where is this house located? Could you please provide the name of the cross-streets at the nearest intersection? Interviewer: Please type in the names of the cross-streets. If the participant cannot provide this information, ask them the name of the neighborhood/colonia and type in that info.

———

If Q1068B is equal to 1 then skip to Q1071

If not, then skip to instruction before Q1072.

Q1071. You said that the last close contact with [Response to Q905] occurred at work. Could you please provide the name of the work place and the address? nterviewer: If the work place does not have a name or address, record whatever info the participant can provide for the location (i.e., cross-streets, neighborhood/colonia).

———

If Q1068C is equal to 1 then skip to Q1072

If not, then skip to instruction before Q1073.

Q1072. You said that the last close contact with [Response to Q905] occurred at a restaurant/cafe. Could you please provide the name of this place and the cross-streets/neighborhood/colonia where this restaurant is located?

———

If Q1068D is equal to 1 then skip to Q1073

If not, then skip to instruction before Q1074.

Q1073. You said that the last close contact with [Response to Q905] occurred at a place of leisure (i.e. a theater, bar, night club). Could you please provide the name of the place and the cross-streets/neighborhood/colonia where this place is located?

———

If Q1068E is equal to 1 then skip to Q1074

If not, then skip to instruction before Q1075.

Q1074. You said that the last close contact with [Response to Q905] occurred at a school/education place. Could you please provide the name of this place and the cross-streets/neighborhood/colonia where this place is located?

— —

If Q1068F is equal to 1 then skip to Q1075

If not, then skip to instruction before Q1076.

Q1075. You said that the last close contact with [Response to Q905] occurred at a gym/fitness place. Could you please provide the name of this place and the cross streets/neighborhood/colonia where this place is located?

— —

If Q1068G is equal to 1 then skip to Q1076

If not, then skip to instruction before Q1077.

Q1076. You said that the last close contact with [Response to Q905] occurred at a personal service (i.e. haircut, massage) place. Could you please provide the name of this place and the cross streets/neighborhood/colonia where this place is located?

— —

If Q1068H is equal to 1 then skip to Q1077

If not, then skip to instruction before Q1078.

Q1077. You said that the last close contact with [Response to Q905] occurred at a religious service place. Could you please provide the name of this place and the cross streets/neighborhood/colonia where this place is located?

— —

If Q1068I is equal to 1 then skip to Q1078

If not, then skip to instruction before Q1079.

Q1078. You said that the last close contact with [Response to Q905] occurred at a community center/group place. Could you please provide the name of this place and the cross streets/neighborhood/colonia where this place is located?

— —

If Q1068J is equal to 1 then skip to Q1079

If not, then skip to instruction before Q1080.

Q1079. You said that the last close contact with [Response to Q905] occurred at a park. Could you please provide the name of this place and the cross streets/neighborhood/colonia where this place is located?

— —

If Q1068K is equal to 1 then skip to Q1080

If not, then skip to instruction before Q1081.

Q1080. You said that the last close contact with [Response to Q905] occurred on the street. Could you please provide the name of thie street and the neighborhood/colonia where this place is located?

— —

If Q1068M is equal to 1 then skip to Q1081

If not, then skip to instruction before Q1082.

Q1081. You said that the last close contact with [Response to Q905] occurred at a shooting gallery. Which shotting gallery did it occur at? (Choose one)

01	Las Palmitas/Zona Norte
02	Los Pinos/ Zona Norte
03	Las Bombas/ Zona Norte
04	Los Aragón/ Zona Norte
05	La Veinte/ Bordo, Col. Veinte de Noviembre
06	El Azteca/ Cerro Colorado
07	Oxxo, Calle 5ta Zona Norte
08	Bordo/ Los Álamos, Mundo divertido
09	Cañón Guerrero/Maclovio Herrera Colonia Guerrero
10	Col. Guaycura (casa privada)
11	Col. Presidentes (Infonavit edificio)
12	Florido 1era Sección (casa privada)
13	Florido 1era Sección (Bomberos, conecta)
14	Col. Francisco Villa (edificio abandonado)
15	Cañón del Pato/ Colonia Villa
16	Col. Postal (casa privada)
17	Col. del Río (Parte baja, barranco)
18	Sánchez Taboada (Av. Sánchez Taboada y Parque Industrial)
19	La cima (casa abandonado)
20	Otay módulos
21	Other
98	Refuse to Answer

If Q1081 is equal to 21 then skip to Q1082

If not, then skip to instruction before Q1083.

Q1082. Please specify the shooting gallery (i.e., name of the place and cross-streets or neighborhood/colonia) where the last close contact wiith [Response to Q905] occurred.

____

If Q686 + Q707 + Q728 + Q749 + Q770 + Q791 + Q812 + Q833 + Q854 + Q875 is greater than 4 and Q908 is not equal to "Refuse to Answer" and Q908 is not equal to "skipped" then skip to Q1083

If not, then skip to instruction before Q1126.

Q1083. Is [Response to Q908] [MCL5] ([MCN5]) male, female, transgender, non-binary or another? (Choose one)

1	Male
2	Female
3	Trans female
4	Trans male
5	Non-binary / genderqueer / gender fluid / agender
6	Another gender identity not listed
8	Refuse to Answer

If Q1083 is equal to 6 then skip to Q1084

If not, then skip to Q1085.

Q1084. Please specify the gender identity of [Response to Q908].

____

Q1085. Where does [Response to Q908] primarily live? (Choose one)

1	Mexico
2	United States
3	Other
7	Don't Know
8	Refuse to Answer

If Q1085 is equal to 3 then skip to Q1086

If not, then skip to Q1087.

Q1086. Please specify where [Response to Q908] primarily lives.

____

Q1087. Did you meet [Response to Q908] for the first time in the previous 14 days?

1 Yes

0 No

8 Refuse to Answer

Q1088. How many days have you seen [Response to Q908] in the previous 14 days?

__ __ Days

98 Refuse to Answer

Q1089. On average, on the days that you saw [Response to Q908], how much time (e.g., hours, minutes) did you spend in close contact with him/her? Interviewer: the max amount of time per day is 24 hours.

__ __ HOURS

__ __ MINUTES

98 Refuse to Answer (Hours)

Q1090. When was the last time you had close contact with [Response to Q908] in the past 14 days? Interviewer: Enter how many days ago the participant had close contact for the last time with the person in question. If the last time was today, enter 0.

__ __ DAYS

98 Refuse to Answer (Days)

If Q1088 is greater than 1 then skip to Q1091

If not, then skip to Q1092.

Q1091. During that last contact, on how many consecutive days did you see [Response to Q908]?

__ __	days
98	Refuse to Answer

MC5FDAY = If(Q1090 is equal to 1, Q1090, If(Q1090 is not equal to 98 and Q1090 is greater than 1, Q1090 + Q1091, "missing"))

Q1092. When you saw [Response to Q908] that last time, what type of contact did you have with them? (Check all that apply) (Check all that apply)

__	Physical, non-sexual [i.e. handshake, hug, skin contact, etc.]
__	Physical, sexual
__	Non-physical [i.e. in-person conversation, meal/drinks, meeting]
__	Refuse to Answer

Q1093. When you saw [Response to Q908] that last time, at which of the following places did the close contact occur? (Check all that apply) (Check all that apply)

__ Home/someone's house

__ Work

__ Restaurant or cafe

__ Leisure (i.e. a theater, bar, night club)

__ School/education

__ Exercise or fitness related (i.e., gym, fitness studio)

__ Personal service (i.e. haircut, massage)

__ Religious service (place of worshiip)

__ Community Center/Group

__ Park

__ Street

__ El Bordo/Canal

__ Shooting gallery

__ Other

__ Refuse to Answer

If Q1093N is equal to 1 then skip to Q1094

If not, then skip to instruction before Q1095.

Q1094. Please specify the place (i.e., name of the place and cross-streets or neighborhood/colonia) where the last close contact wiith [Response to Q908] occurred.

____

If Q1093A is equal to 1 then skip to Q1095

If not, then skip to instruction before Q1096.

Q1095. You said the the last close contact with [Response to Q908] occurred at your house/someone else's house. Where is this house located? Could you please provide the name of the cross-streets at the nearest intersection? Interviewer: Please type in the names of the cross-streets. If the participant cannot provide this information, ask them the name of the neighborhood/colonia and type in that info.

——

If Q1093B is equal to 1 then skip to Q1096

If not, then skip to instruction before Q1097.

Q1096. You said that the last close contact with [Response to Q908] occurred at work. Could you please provide the name of the work place and the address? nterviewer: If the work place does not have a name or address, record whatever info the participant can provide for the location (i.e., cross-streets, neighborhood/colonia).

——

If Q1093C is equal to 1 then skip to Q1097

If not, then skip to instruction before Q1098.

Q1097. You said that the last close contact with [Response to Q908] occurred at a restaurant/cafe. Could you please provide the name of this place and the cross-streets/neighborhood/colonia where this restaurant is located?

——

If Q1093D is equal to 1 then skip to Q1098

If not, then skip to instruction before Q1099.

Q1098. You said that the last close contact with [Response to Q908] occurred at a place of leisure (i.e. a theater, bar, night club). Could you please provide the name of the place and the cross-streets/neighborhood/colonia where this place is located?

——

If Q1093E is equal to 1 then skip to Q1099

If not, then skip to instruction before Q1100.

Q1099. You said that the last close contact with [Response to Q908] occurred at a school/education place. Could you please provide the name of this place and the cross-streets/neighborhood/colonia where this place is located?

— —

If Q1093F is equal to 1 then skip to Q1100

If not, then skip to instruction before Q1101.

Q1100. You said that the last close contact with [Response to Q908] occurred at a gym/fitness place. Could you please provide the name of this place and the cross streets/neighborhood/colonia where this place is located?

— —

If Q1093G is equal to 1 then skip to Q1101

If not, then skip to instruction before Q1102.

Q1101. You said that the last close contact with [Response to Q908] occurred at a personal service (i.e. haircut, massage) place. Could you please provide the name of this place and the cross streets/neighborhood/colonia where this place is located?

— —

If Q1093H is equal to 1 then skip to Q1102

If not, then skip to instruction before Q1103.

Q1102. You said that the last close contact with [Response to Q908] occurred at a religious service place. Could you please provide the name of this place and the cross streets/neighborhood/colonia where this place is located?

— —

If Q1093I is equal to 1 then skip to Q1103

If not, then skip to instruction before Q1104.

Q1103. You said that the last close contact with [Response to Q908] occurred at a community center/group place. Could you please provide the name of this place and the cross streets/neighborhood/colonia where this place is located?

— —

If Q1093J is equal to 1 then skip to Q1104

If not, then skip to instruction before Q1105.

Q1104. You said that the last close contact with [Response to Q908] occurred at a park. Could you please provide the name of this place and the cross streets/neighborhood/colonia where this place is located?

— —

If Q1093K is equal to 1 then skip to Q1105

If not, then skip to instruction before Q1106.

Q1105. You said that the last close contact with [Response to Q908] occurred on the street. Could you please provide the name of thie street and the neighborhood/colonia where this place is located?

— —

If Q1093M is equal to 1 then skip to Q1106

If not, then skip to instruction before Q1107.

Q1106. You said that the last close contact with [Response to Q908] occurred at a shooting gallery. Which shotting gallery did it occur at? (Choose one)

01	Las Palmitas/Zona Norte
02	Los Pinos/ Zona Norte
03	Las Bombas/ Zona Norte
04	Los Aragón/ Zona Norte
05	La Veinte/ Bordo, Col. Veinte de Noviembre
06	El Azteca/ Cerro Colorado
07	Oxxo, Calle 5ta Zona Norte
08	Bordo/ Los Álamos, Mundo divertido
09	Cañón Guerrero/Maclovio Herrera Colonia Guerrero
10	Col. Guaycura (casa privada)
11	Col. Presidentes (Infonavit edificio)
12	Florido 1era Sección (casa privada)
13	Florido 1era Sección (Bomberos, conecta)
14	Col. Francisco Villa (edificio abandonado)
15	Cañón del Pato/ Colonia Villa
16	Col. Postal (casa privada)
17	Col. del Río (Parte baja, barranco)
18	Sánchez Taboada (Av. Sánchez Taboada y Parque Industrial)
19	La cima (casa abandonado)
20	Otay módulos
21	Other
98	Refuse to Answer

If Q1106 is equal to 21 then skip to Q1107

If not, then skip to instruction before Q1108.

Q1107. Please specify the shooting gallery (i.e., name of the place and cross-streets or neighborhood/colonia) where the last close contact wiith [Response to Q908] occurred.

__ __

If Q1088 is greater than 1 then skip to Q1108

If not, then skip to instruction before Q1126.

Q1108. Please think of a different time that you saw [Response to Q908] in the past 14 days, other than the contact that you just described. By 'time' we mean a contact where you saw [Response to Q908] in person and then separated. How many days ago did that take place?

__ __	DAYS
98	Refuse to Answer (Days)

If Q1108 is less than MC5FDAY then READ: "This time is more recent than the first day of the last contact. Please make corrections." and skip to Q1108.

If Q1088 is greater than 1 then skip to Q1109

If not, then skip to Q1110.

Q1109. During this contact, on how many consecutive days did you see [Response to Q908]?

__ __	days
98	Refuse to Answer

Q1110. When you saw [Response to Q908] that time, what type of contact did you have with them? (Check all that apply) (Check all that apply)

__	Physical, non-sexual [i.e. handshake, hug, skin contact, etc.]
__	Physical, sexual
__	Non-physical [i.e. in-person conversation, meal/drinks, meeting]
__	Refuse to Answer

Q1111. When you saw [Response to Q908] that time, at which of the following places did the close contact occur? (Check all that apply) (Check all that apply)

__ Home/someone's house

__ Work

__ Restaurant or cafe

__ Leisure (i.e. a theater, bar, night club)

__ School/education

__ Exercise or fitness related (i.e., gym, fitness studio)

__ Personal service (i.e. haircut, massage)

__ Religious service (place of worshiip)

__ Community Center/Group

__ Park

__ Street

__ El Bordo/Canal

__ Shooting gallery

__ Other

__ Refuse to Answer

If Q1111N is equal to 1 then skip to Q1112

If not, then skip to instruction before Q1113.

Q1112. Please specify the place (i.e., name of the place and cross-streets or neighborhood/colonia) where that close contact wiith [Response to Q908] occurred.

____

If Q1111A is equal to 1 then skip to Q1113

If not, then skip to instruction before Q1114.

Q1113. You said the the last close contact with [Response to Q908] occurred at your house/someone else's house. Where is this house located? Could you please provide the name of the cross-streets at the nearest intersection? Interviewer: Please type in the names of the cross-streets. If the participant cannot provide this information, ask them the name of the neighborhood/colonia and type in that info.

____

If Q1111B is equal to 1 then skip to Q1114

If not, then skip to instruction before Q1115.

Q1114. You said that the last close contact with [Response to Q908] occurred at work. Could you please provide the name of the work place and the address? nterviewer: If the work place does not have a name or address, record whatever info the participant can provide for the location (i.e., cross-streets, neighborhood/colonia).

____

If Q1111C is equal to 1 then skip to Q1115

If not, then skip to instruction before Q1116.

Q1115. You said that the last close contact with [Response to Q908] occurred at a restaurant/cafe. Could you please provide the name of this place and the cross-streets/neighborhood/colonia where this restaurant is located?

____

If Q1111D is equal to 1 then skip to Q1116

If not, then skip to instruction before Q1117.

Q1116. You said that the last close contact with [Response to Q908] occurred at a place of leisure (i.e. a theater, bar, night club). Could you please provide the name of the place and the cross-streets/neighborhood/colonia where this place is located?

____

If Q1111E is equal to 1 then skip to Q1117

If not, then skip to instruction before Q1118.

Q1117. You said that the last close contact with [Response to Q908] occurred at a school/education place. Could you please provide the name of this place and the cross-streets/neighborhood/colonia where this place is located?

— —

If Q1111F is equal to 1 then skip to Q1118

If not, then skip to instruction before Q1119.

Q1118. You said that the last close contact with [Response to Q908] occurred at a gym/fitness place. Could you please provide the name of this place and the cross streets/neighborhood/colonia where this place is located?

— —

If Q1111G is equal to 1 then skip to Q1119

If not, then skip to instruction before Q1120.

Q1119. You said that the last close contact with [Response to Q908] occurred at a personal service (i.e. haircut, massage) place. Could you please provide the name of this place and the cross streets/neighborhood/colonia where this place is located?

— —

If Q1111H is equal to 1 then skip to Q1120

If not, then skip to instruction before Q1121.

Q1120. You said that the last close contact with [Response to Q908] occurred at a religious service place. Could you please provide the name of this place and the cross streets/neighborhood/colonia where this place is located?

— —

If Q1111I is equal to 1 then skip to Q1121

If not, then skip to instruction before Q1122.

Q1121. You said that the last close contact with [Response to Q908] occurred at a community center/group place. Could you please provide the name of this place and the cross streets/neighborhood/colonia where this place is located?

— —

If Q1111J is equal to 1 then skip to Q1122

If not, then skip to instruction before Q1123.

Q1122. You said that the last close contact with [Response to Q908] occurred at a park. Could you please provide the name of this place and the cross streets/neighborhood/colonia where this place is located?

— —

If Q1111K is equal to 1 then skip to Q1123

If not, then skip to instruction before Q1124.

Q1123. You said that the last close contact with [Response to Q908] occurred on the street. Could you please provide the name of thie street and the neighborhood/colonia where this place is located?

— —

If Q1111M is equal to 1 then skip to Q1124

If not, then skip to instruction before Q1125.

Q1124. You said that the last close contact with [Response to Q908] occurred at a shooting gallery. Which shotting gallery did it occur at? (Choose one)

01	Las Palmitas/Zona Norte
02	Los Pinos/ Zona Norte
03	Las Bombas/ Zona Norte
04	Los Aragón/ Zona Norte
05	La Veinte/ Bordo, Col. Veinte de Noviembre
06	El Azteca/ Cerro Colorado
07	Oxxo, Calle 5ta Zona Norte
08	Bordo/ Los Álamos, Mundo divertido
09	Cañón Guerrero/Maclovio Herrera Colonia Guerrero
10	Col. Guaycura (casa privada)
11	Col. Presidentes (Infonavit edificio)
12	Florido 1era Sección (casa privada)
13	Florido 1era Sección (Bomberos, conecta)
14	Col. Francisco Villa (edificio abandonado)
15	Cañón del Pato/ Colonia Villa
16	Col. Postal (casa privada)
17	Col. del Río (Parte baja, barranco)
18	Sánchez Taboada (Av. Sánchez Taboada y Parque Industrial)
19	La cima (casa abandonado)
20	Otay módulos
21	Other
98	Refuse to Answer

If Q1124 is equal to 21 then skip to Q1125

If not, then skip to instruction before Q1126.

Q1125. Please specify the shooting gallery (i.e., name of the place and cross-streets or neighborhood/colonia) where the last close contact wiith [Response to Q908] occurred.

____

READ: Now we will ask you about potential changes in contact behaviors related to SARSCoV-2.

Try to think back to the time before we were aware of the novel coronavirus epidemic.

Q1126. How many friends or people that you associated with, who you talked to about things that were important to you do you no longer see due to the pandemic?

__ __ __ people

998 Refuse to Answer

If Q1126 is greater than 0 and Q1126 is not equal to 998 then skip to Q1127

If not, then skip to instruction before Q1135.

Q1127. Of the people you no longer see, how many were: Sex Partners? __ __ __ people

998 Refuse to Answer

If Q1127 is not equal to 998 and Q1127 is greater than Q1126 then READ: "This number is bigger than the total number. Please make corrections." and skip to Q1127.

If Q1127 is not equal to 998 and Q1126 is greater than Q1127 then skip to Q1128

If not, then skip to instruction before Q1129.

Q1128. Of the people you no longer see, how many were: Friends? __ __ __ people

998 Refuse to Answer

If Q1128 is not equal to 998 and Q1127 is not equal to 998 and Q1128 is greater than Q1126 - Q1127 then READ: "This number is bigger than the total number. Please make corrections." and skip to Q1128.

If Q1127 is not equal to 998 and Q1128 is not equal to 998 and Q1126 is greater than Q1127 + Q1128 then skip to Q1129

If not, then skip to instruction before Q1130.

Q1129. Of the people you no longer see, how many were: Neighbor or Housemates?

__ __ __	people
998	Refuse to Answer

If Q1129 is not equal to 998 and Q1127 is not equal to 998 and Q1128 is not equal to 998 and Q1129 is greater than Q1126 - Q1127 - Q1128 then READ: "This number is bigger than the total number. Please make corrections." and skip to Q1129.

If Q1127 is not equal to 998 and Q1128 is not equal to 998 and Q1129 is not equal to 998 and Q1126 is greater than Q1127 + Q1128 + Q1129 then skip to Q1130

If not, then skip to instruction before Q1131.

Q1130. Of the people you no longer see, how many were: Co-workers or Bosses?

__ __ __	people
998	Refuse to Answer

If Q1130 is not equal to 998 and Q1127 is not equal to 998 and Q1128 is not equal to 998 and Q1129 is not equal to 998 and Q1130 is greater than Q1126 - Q1127 - Q1128 - Q1129 then READ: "This number is bigger than the total number. Please make corrections." and skip to Q1130.

If Q1127 is not equal to 998 and Q1128 is not equal to 998 and Q1129 is not equal to 998 and Q1130 is not equal to 998 and Q1126 is greater than Q1127 + Q1128 + Q1129 + Q1130 then skip to Q1131

If not, then skip to instruction before Q1132.

Q1131. Of the people you no longer see, how many were: Family-relatives by blood or marriage

__ __ __	people
998	Refuse to Answer

If Q1131 is not equal to 998 and Q1130 is not equal to 998 and Q1127 is not equal to 998 and Q1128 is not equal to 998 and Q1129 is not equal to 998 and Q1131 is greater than Q1126 - Q1127 - Q1128 - Q1129 - Q1130 then READ: "This number is bigger than the total number. Please make corrections." and skip to Q1131.

If Q1127 is not equal to 998 and Q1128 is not equal to 998 and Q1129 is not equal to 998 and Q1130 is not equal to 998 and Q1131 is not equal to 998 and Q1126 is greater than Q1127 + Q1128 + Q1129 + Q1130 + Q1131 then skip to Q1132

If not, then skip to instruction before Q1133.

Q1132. Of the people you no longer see, how many were: Drug or Alcohol Related

___ ___ ___ people

998 Refuse to Answer

If Q1132 is not equal to 998 and Q1131 is not equal to 998 and Q1130 is not equal to 998 and Q1127 is not equal to 998 and Q1128 is not equal to 998 and Q1129 is not equal to 998 and Q1132 is greater than Q1126 - Q1127 - Q1128 - Q1129 - Q1130 - Q1131 then READ: "This number is bigger than the total number. Please make corrections." and skip to Q1132.

If Q1127 is not equal to 998 and Q1128 is not equal to 998 and Q1129 is not equal to 998 and Q1130 is not equal to 998 and Q1131 is not equal to 998 and Q1132 is not equal to 998 and Q1126 is greater than Q1127 + Q1128 + Q1129 + Q1130 + Q1131 + Q1132 then skip to Q1133

If not, then skip to instruction before Q1134.

Q1133. Of the people you no longer see, how many were: Other ___ ___ ___ people

998 Refuse to Answer

If Q1133 is not equal to 998 and Q1132 is not equal to 998 and Q1131 is not equal to 998 and Q1130 is not equal to 998 and Q1127 is not equal to 998 and Q1128 is not equal to 998 and Q1129 is not equal to 998 and Q1133 is greater than Q1126 - Q1127 - Q1128 - Q1129 - Q1130 - Q1131 - Q1132 then READ: "This number is bigger than the total number. Please make corrections." and skip to Q1133.

If Q1133 is greater than 0 then skip to Q1134

If not, then skip to instruction before Q1135.

Q1134. Please specify.

____

READ: Now try to remember the two first weeks of February 2020 (or a typical 14 day period in 2020 before we were aware of the novel coronavirus epidemic).

Q1135. How many people would you normally have had close contact with over those 14 days? Remember, we are interested in people you've had: Non-physical (face-to-face) contact with (within 6ft) that **lasted over an hour** OR Physical contact with. Again, feel free to look through your phone or calendar to jog your memory.

___ ___ ___ people

998 Refuse to Answer

If Q1135 is greater than 0 and Q1135 is not equal to 998 then skip to Q1136

If not, then skip to Q1150.

Q1136. How many of these close contacts were: Living in the United States?

__ __ __	people
997	Don't Know
998	Refuse to Answer

If Q1136 is not equal to 997 and Q1136 is not equal to 998 and Q1136 is greater than Q1135 then READ: "This number is bigger than the total number. Please make corrections." and skip to Q1136.

If Q1135 is greater than Q1136 then skip to Q1137

If not, then skip to instruction before Q1138.

Q1137. How many of these close contacts were: Living in Mexico?

__ __ __	people
997	Don't Know
998	Refuse to Answer

If Q1136 is not equal to 997 and Q1136 is not equal to 998 and Q1137 is not equal to 997 and Q1137 is not equal to 998 and Q1137 is greater than Q1135 - Q1136 then READ: "This number is bigger than the total number. Please make corrections." and skip to Q1137.

Q1138. How many of these close contacts were: Male?

__ __ __	people
998	Refuse to Answer

If Q1138 is not equal to 998 and Q1138 is greater than Q1135 then READ: "This number is bigger than the total number. Please make corrections." and skip to Q1138.

If Q1135 is greater than Q1138 then skip to Q1139

If not, then skip to instruction before Q1140.

Q1139. How many of these close contacts were: Female?

__ __ __	people
997	Don't Know
998	Refuse to Answer

If Q1138 is not equal to 998 and Q1139 is not equal to 998 and Q1139 is greater than Q1135 - Q1138 then READ: "This number is bigger than the total number. Please make corrections." and skip to Q1139.

If Q1135 is greater than Q1138 + Q1139 then skip to Q1140

If not, then skip to instruction before Q1141.

Q1140. How many of these close contacts were: Trans Male? __ __ __ people

997 Don't Know

998 Refuse to Answer

If Q1140 is not equal to 998 and Q1140 is greater than Q1135 then READ: "This number is bigger than the total number. Please make corrections." and skip to Q1140.

If Q1135 is greater than Q1138 + Q1139 + Q1140 then skip to Q1141

If not, then skip to instruction before Q1142.

Q1141. How many of these close contacts were: Trans Female? __ __ __ people

997 Don't Know

998 Refuse to Answer

If Q1140 is not equal to 998 and Q1141 is not equal to 998 and Q1141 is greater than Q1135 - Q1140 then READ: "This number is bigger than the total number. Please make corrections." and skip to Q1141.

If Q1135 is greater than Q1138 + Q1139 + Q1140 + Q1141 then skip to Q1142

If not, then skip to instruction before Q1143.

Q1142. How many of these close contacts were: Non-binary/genderqueer/gender fluid/agender?

__ __ __ people

997 Don't Know

998 Refuse to Answer

If Q1142 is not equal to 998 and Q1140 is not equal to 998 and Q1141 is not equal to 998 and Q1142 is greater than Q1135 - Q1140 - Q1141 then READ: "This number is bigger than the total number. Please make corrections." and skip to Q1142.

If Q1135 is greater than Q1138 + Q1139 + Q1140 + Q1141 + Q1142 then skip to Q1143

If not, then skip to instruction before Q1144.

Q1143. How many of these close contacts were: Another identity not listed?

__ __ __	people
997	Don't Know
998	Refuse to Answer

If Q1143 is not equal to 998 and Q1142 is not equal to 998 and Q1140 is not equal to 998 and Q1141 is not equal to 998 and Q1143 is greater than Q1135 - Q1140 - Q1141 - Q1142 then READ: "This number is bigger than the total number. Please make corrections." and skip to Q1143.

If Q1143 is greater than 0 then skip to Q1144

If not, then skip to Q1145.

Q1144. Please specify.

__ __

Q1145. How many of these close contacts were: <18 years old?

__ __ __	people
997	Don't Know
998	Refuse to Answer

If Q1145 is not equal to 997 and Q1145 is not equal to 998 and Q1145 is greater than Q1135 then READ: "This number is bigger than the total number. Please make corrections." and skip to Q1145.

If Q1135 is greater than Q1145 then skip to Q1146

If not, then skip to instruction before Q1147.

Q1146. How many of these close contacts were: 18-35 years old?

__ __ __	people
997	Don't Know
998	Refuse to Answer

If Q1145 is not equal to 997 and Q1145 is not equal to 998 and Q1146 is not equal to 997 and Q1146 is not equal to 998 and Q1146 is greater than Q1135 - Q1145 then READ: "This number is bigger than the total number. Please make corrections." and skip to Q1146.

If Q1135 is greater than Q1145 + Q1146 then skip to Q1147

If not, then skip to instruction before Q1148.

Q1147. How many of these close contacts were: >35 years old? __ __ __ people

997 Don't Know

998 Refuse to Answer

If Q1145 is not equal to 997 and Q1145 is not equal to 998 and Q1146 is not equal to 997 and Q1146 is not equal to 998 and Q1147 is not equal to 997 and Q1147 is not equal to 998 and Q1147 is greater than Q1135 - Q1145 - Q1146 then READ: "This number is bigger than the total number. Please make corrections." and skip to Q1147.

Q1148. How many of these close contacts did you have vaginal or anal sexual contact with over those 14 days?

__ __ __ people

998 Refuse to Answer

If Q1148 is not equal to 998 and Q1148 is greater than Q1135 then READ: "This number is bigger than the total number. Please make corrections." and skip to Q1148.

Q1149. How many of these close contacts did you use drugs with over those 14 days?

__ __ __ people

998 Refuse to Answer

If Q1149 is not equal to 998 and Q1149 is greater than Q1135 then READ: "This number is bigger than the total number. Please make corrections." and skip to Q1149.

Q1150. **Interviewer:** How honest do you think this participant was in their responses? (Choose one)

1 Not honest at all

2 Somewhat dishonest

3 Somewhat honest

4 Very honest

If Q204 is equal to 4 and (Q195 is equal to 1 or Q197 is equal to 1 or Q207 is equal to 1 or Q209 is equal to 1 or Q211 is equal to 1 or Q225 is equal to 1 or Q227 is equal to 1 or Q231 is equal to 1) or Q229 is equal to 1 then skip to Q1151

If not, then skip to instruction before Q1152.

Q1151. **Interviewer: This is a reminder to provide the participant with a TB referral. Please confirm.** (Choose one)

1 Yes

If Q191 is equal to 1 or Q193 is equal to 1 or Q195 is equal to 1 or Q197 is equal to 1 or Q199 is equal to 1 or Q201 is equal to 1 or Q203 is equal to 1 or Q205 is equal to 1 or Q207 is equal to 1 or Q209 is equal to 1 or Q213 is equal to 1 or Q215 is equal to 1 or Q217 is equal to 1 or Q219 is equal to 1 or Q221 is equal to 1 or Q223 is equal to 1 or Q227 is equal to 1 then skip to Q1152

If not, then skip to end of questionnaire.

Q1152. **Interviewer: This is a reminder to provide the participant with a COVID-19 referral. Please confirm.** (Choose one)

1 Yes

READ: End of survey.

Proyecto La Frontera

Entrevista Suplemento

Descargo de responsabilidad: Esta entrevista ha sido adaptada para su programación en QDS y no ha sido adaptada para ser administrada con papel y lápiz

IP: Steffanie Strathdee
10/18/2021

LaFrontera.Supplemental.20210820 (Español)

READ: La Frontera
Entrevista suplementaria
Español

VERSION = 20210820

Q1. Por favor ingresar la fecha de entrevista __ __ / __ __ / __ __ __ __ dd / mm / aaaa

If Q1 is greater than TODAY and TODAY is not equal to "Falta" then READ: "Esa fecha ocurre en el futuro. Verificar respuesta." and skip to Q1.

Q2. Iniciales del Entrevistador. __

Q3. Ciudad de entrevista.
Entrevistador, ¡es crucial que ingrese correctamente el lugar de la entrevista!
(Elija Uno)

1 San Diego

2 Tijuana

Q4. Entrevistador, ¿cómo se realizará esta entrevista? (Elija Uno)

1 En persona, adentro

2 En persona, afuera

3 Por video llamada (por ejemplo: Zoom o Skype)

4 Por audio llamada (por ejemplo telefono, Zoom)

5 Otro

8 Se rehúsa a contestar

If Q4 is not equal to 5, then skip to Q6.

Q5. Por favor especifique de qué otra manera.

__ __

Q6. ID de Participante (e.g., LF001) __ __ __ __ __

ID1 = SubString(Q6, 0, 1)

ID2 = SubString(Q6, 1, 1)

ID3 = SubString(Q6, 2, 1)

ID4 = SubString(Q6, 3, 1)

ID5 = SubString(Q6, 4, 1)

IDSTUDY = Concatenated(ID1, ID2)

If IDSTUDY is not equal to LF then READ: "Verificar ID" and skip to Q6.

If ID3 is not equal to 0 and ID3 is not equal to 1 and ID3 is not equal to 2 and ID3 is not equal to 3 and ID3 is not equal to 4 and ID3 is not equal to 5 and ID3 is not equal to 6 and ID3 is not equal to 7 and ID3 is not equal to 8 and ID3 is not equal to 9 or ID4 is not equal to 0 and ID4 is not equal to 1 and ID4 is not equal to 2 and ID4 is not equal to 3 and ID4 is not equal to 4 and ID4 is not equal to 5 and ID4 is not equal to 6 and ID4 is not equal to 7 and ID4 is not equal to 8 and ID4 is not equal to 9 or ID5 is not equal to 0 and ID5 is not equal to 1 and ID5 is not equal to 2 and ID5 is not equal to 3 and ID5 is not equal to 4 and ID5 is not equal to 5 and ID5 is not equal to 6 and ID5 is not equal to 7 and ID5 is not equal to 8 and ID5 is not equal to 9 then READ: "Verificar ID" and skip to Q6.

Q7. Por favor ingresar ID de SCREENER del participante (ej. LF0001) **NOTA PARA Entrevistador: Por favor no confundir el número de screener con el ID de Participante.**

__ __ __ __ __ __

SCRID1 = SubString(Q7, 0, 1)

SCRID2 = SubString(Q7, 1, 1)

SCRID3 = SubString(Q7, 2, 1)

SCRID4 = SubString(Q7, 3, 1)

SCRID5 = SubString(Q7, 4, 1)

SCRID6 = SubString(Q7, 5, 1)

IDSCREEN = Concatenated(SCRID1, SCRID2)

If IDSCREEN is not equal to LF then READ: "Verificar SCRID." and skip to Q7.

If SCRID3 is not equal to 0 and SCRID3 is not equal to 1 and SCRID3 is not equal to 2 and SCRID3 is not equal to 3 and SCRID3 is not equal to 4 and SCRID3 is not equal to 5 and SCRID3 is not equal to 6 and SCRID3 is not equal to 7 and SCRID3 is not equal to 8 and SCRID3 is not equal to 9 or SCRID4 is not equal to 0 and SCRID4 is not equal to 1 and SCRID4 is not equal to 2 and SCRID4 is not equal to 3 and SCRID4 is not equal to 4 and SCRID4 is not equal to 5 and SCRID4 is not equal to 6 and SCRID4 is not equal to 7 and SCRID4 is not equal to 8 and SCRID4 is not equal to 9 or SCRID5 is not equal to 0 and SCRID5 is not equal to 1 and SCRID5 is not equal to 2 and SCRID5 is not equal to 3 and SCRID5 is not equal to 4 and SCRID5 is not equal to 5 and SCRID5 is not equal to 6 and SCRID5 is not equal to 7 and SCRID5 is not equal to 8 and SCRID5 is not equal to 9 or SCRID6 is not equal to 0 and SCRID6 is not equal to 1 and SCRID6 is not equal to 2 and SCRID6 is not equal to 3 and SCRID6 is not equal to 4 and SCRID6 is not equal to 5 and SCRID6 is not equal to 6 and SCRID6 is not equal to 7 and SCRID6 is not equal to 8 and SCRID6 is not equal to 9 then READ: "Verificar ID" and skip to Q6.

Q8. Lugar de entrevista. (Escoja uno) (Elija Uno)

1 Tijuana

2 San Diego

3 Otro

If Q8 is equal to 3 then skip to Q9

If not, then skip to instruction before Q11.

Q9. Por favor especifica lugar(ciudad) de entrevista

____

Q10. Por favor especifica lugar de entrevista en [Response to Q9]

____

If Q8 is equal to 1 then skip to Q11

If not, then skip to instruction before Q13.

Q11. Lugar de entrevista en Tijuana (escoja solo una) (Elija Uno)

1 Tijuana 1 Zona Norte - El Mapa

2 Tijuana 2 Coahuila

3 Tijuana 3

4 Rehab

5 Cárcel

6 Otro

If Q11 is equal to 6 then skip to Q12

If not, then skip to instruction before Q13.

Q12. Especifica lugar de entrevista en Tijuana.

____

If Q8 is equal to 2 then skip to Q13

If not, then skip to Q15.

Q13. Lugar de entrevista en San Diego. (escoja solo una) (Elija Uno)

1 San Diego 1

2 San Diego 2

3 San Diego 3

4 Rehab

5 Cárcel

6 Otro

If Q13 is equal to 6 then skip to Q14

If not, then skip to Q15.

Q14. Especifica lugar de entrevista en San Diego

__ __
__ __
__ __

Q15. ¿En qué país vives? (**escoja solo una**) (Elija Uno)

1 Estados Unidos

2 México

3 Otro

8 Se rehúsa a contestar

If Q15 is equal to 3 then skip to Q16

If not, then skip to instruction before Q17.

Q16. Especifica el país en dónde vives.

__ __
__ __
__ __

If Q15 is equal to 1 then skip to Q17

If not, then skip to instruction before Q19.

Q17. ¿En qué región de San Diego vives? (**escoja solo una**) (Elija Uno)

1 Ciudad de San Diego

2 Condado Este (East County)

3 Condado Norte de la costa (North County Costal)

4 Condado Norte del interior (North County Inland)

5 El Sur de la Bahia (South Bay)

6 Otra región de San Diego

7 No vivo en el condado de San Diego

8 Se rehúsa a contestar

If Q17 is not equal to 6, then skip to instruction before Q19.

Q18. Por favor especifica la región en donde vives.

____

If Q15 is equal to 2 then skip to Q19

If not, then skip to instruction before Q21.

Q19. ¿En qué ciudad vives? (**escoja solo una**) (Elija Uno)

1 Tijuana

2 Mexicali

3 Ensenada

4 Rosarito

5 Otro

8 Se rehúsa a contestar

If Q19 is not equal to 5, then skip to instruction before Q21.

Q20. Por favor especifica el nombre de la ciudad en la que vives.

— —
— —
— —
— —
— —
— —

If Q15 is equal to 1 then skip to instruction before Q21

If not, then skip to instruction before Q25.

If Q8 is equal to 2 then skip to Q21

If not, then skip to Q22.

Q21. ¿Alguna vez has estado en México?

1 Si

0 No

8 Se rehúsa a contestar

If Q21 is not equal to 1, then skip to instruction before Q25.

Q22.	¿Alguna vez en su vida ha usado drogas en México?	1	Si
		0	No
		8	Se rehúsa a contestar

If Q22 is not equal to 1, then skip to instruction before Q24.

Q23.	¿Alguna vez en su vida se inyecto drogas en México?	1	Si
		0	No
		8	Se rehúsa a contestar

If Q23 is not equal to 1, then skip to instruction before Q25.

Q24. ¿Cuándo fue la última vez que se inyectó drogas en México?

__ __ / __ __ / __ __ __ __	dd / mm / aaaa
2098	Se rehúsa a contestar (Año)

If Q24 is not equal to 2098 and Q24 is not equal to "Saltó" then skip to instruction before Q25

If not, then skip to instruction before Q25.

INJTMEX = TODAY - Q24

If Q8 is equal to 1 and Q15 is equal to 2 then skip to Q25

If not, then skip to instruction before Q29.

Q25.	¿Alguna vez en su vida ha estado en EUA?	1	Si
		0	No
		8	Se rehúsa a contestar

If Q25 is not equal to 1, then skip to instruction before Q29.

Q26.	¿Alguna vez en tu vida ha usado alguna droga en EUA?	1	Si
		0	No
		8	Se rehúsa a contestar

If Q26 is not equal to 1, then skip to instruction before Q28.

Q27.	¿Alguna vez en su vida se ha inyectado alguna droga en EUA?	1	Si
		0	No
		8	Se rehúsa a contestar

If Q27 is not equal to 1, then skip to instruction before Q29.

Q28. ¿Cuándo fue la última vez que se inyecto alguna droga en EUA?

__ __ / __ __ / __ __ __ __	dd / mm / aaaa
2098	Se rehúsa a contestar (Año)

If Q28 is not equal to 2098 and Q28 is not equal to "Saltó" then skip to instruction before Q29

If not, then skip to instruction before Q29.

INJTUS = TODAY - Q28

NDT_SD = If(Q8 is equal to 2 and (Q17 is equal to 1 or Q17 is equal to 2 or Q17 is equal to 3 or Q17 is equal to 4 or Q17 is equal to 5 or Q17 is equal to 6) and (Q21 is equal to 0 or Q22 is equal to 0), 1, 0)

NDT_TJ = If(Q8 is equal to 1 and Q19 is equal to 1 and (Q25 is equal to 0 or Q26 is equal to 0), 1, 0)

DT_SD = If(NDT_SD is equal to 0 and NDT_TJ is equal to 0, 1, 0)

READ: LEA: ENTREVISTADOR, POR FAVOR LEER EL SIGUIENTE TEXTO EN VOZ ALTA AL PARTICIPANTE: Esta entrevista de suplemento es parte de un estudio de investigación sobre VIH/VHC, SARSCoV-2 y sobredósis entre personas que usan drogas inyectables en San Diego y Tijuana. Las preguntas que estaremos haciendo complementan las pregunta que le hicimos durante su primera visita y están relacionadas a alguna información básica sobre usted, su salud de SARSCoV-2 y TB, sus amigos y personas que usted conoce y el uso de servicios de salud y otros servicios, y sus actitudes hacia algunos de estos problemas. Sé que algunas preguntas que haremos pueden ser un poco incomodas de contestar. Quiero recordarle que al igual que en su primera visita, esta entrevista es confidencial, y nosotros no le daremos información personal suya a nadie fuera de este estudio. Nada de lo que nos diga puede ser relacionado con usted como individuo. Es importante que sepa que su participación en este estudio es totalmente voluntaria. No necesita responder alguna pregunta si no quiere, y puede interrumpir la entrevista en cualquier momento. ¿Tiene alguna pregunta antes de comenzar?

Q29. ¿Cuál es su fecha de nacimiento?

__ __ / __ __ / __ __ __ __ dd / mm / aaaa

2098 Se rehúsa a contestar (Año)

AGE = Age(Q29, TODAY)

Q30. ¿Qué sexo aparece anotado en su acta de nacimiento? (**Escoja solo uno)** (Elija Uno)

1 Hombre

2 Mujer

3 Intersexual

8 Se rehúsa a contestar

READ: LEA: LEER A PARTICIPANTE: Ahora te voy a hacerle algunas preguntas sobre su uso y acceso a agua en su vida diaria.

Q31. En los últimos 6 meses, ¿Cuáles de estas fuentes de agua utilizó para tomar? **Entrevistador: seleccione todas las que apliquen.** (Marque Todas las que Correspondan)

__ Agua de la llave/potable privada (llave de casa)

__ Agua de la llave/potable privada (llave de vecino, familiar, amigo)

__ Agua de la llave pública (parque/gasolinera/afuera de tienda)

__ Agua de aspersores (parques públicos)

__ Pozo desprotegido

__ Pozo protegido

__ Fuente natural desprotegida

__ Fuente natural protegida

__ Agua de lluvia

__ Pipa de agua (camión)

__ Envasada (embotellada/en bolsita/de garrafón)

__ Agua superficial (río/lago/canal)

__ Tubos/hoyos dentro del canal del río Tijuana (lloraderos)

__ Sin recurso de agua

__ Otro

__ Se rehúsa a contestar

If Q31N is equal to 1 and Q31 is not equal to 98 and Q31 is greater than 1 then READ: "Solo puedes escoger la opcion "Sin recurso de agua" por si sola, verificar respuesta." and skip to Q31.

If Q31O is equal to 1 then skip to Q32

If not, then skip to Q33.

Q32. Dijiste que tuviste acceso a otras fuentes de agua en los últimos 6 meses. Por favor especifica.

— —
— —
— —

Q33. ¿Cuál es su fuente principal (la más común) de agua para <u>tomar</u>? **ENTREVISTADOR: escoja solo una** . (Elija Uno)

01 Agua entubada privada (llave de casa)

02 Agua entubada privada (llave de vecino, familiar, amigo)

03 Agua entubada pública (parque/gasolinera/afuera de tienda)

04 Agua de aspersores (parques públicos)

05 Pozo desprotegido

06 Pozo protegido

07 Fuente natural desprotegida

08 Fuente natural protegida

09 Agua de lluvia

10 Pipa de agua (camión)

11 Envasada (embotellada/en bolsita/de garrafón)

12 Agua superficial (río/lago/canal)

13 Tubos/hoyos dentro del canal del río Tijuana (lloraderos)

14 Sin recurso de agua

15 Otro

98 Se rehúsa a contestar

If Q33 is equal to 15 then skip to Q34

If not, then skip to Q35.

Q34. Por favor especifique cuál es su fuente principal (la más común) de agua para tomar.

__ __
__ __
__ __

Q35. Su fuente principal de agua para tomar ¿está siempre disponible para usted? (escoger una) (Elija Uno)

1 Si, siempre

2 El agua está disponible la mayor parte del tiempo

3 El agua está disponible a veces

4 No, el agua raramente está disponible

8 Se rehúsa a contestar

Q36. ¿Cuánto tiempo tardas en conseguir agua para tomar de su fuente principal de agua (caminar de ida, tomar el agua y regresar)? ***Entrevistador, si el participante indica que el agua siempre está disponible, ingresar 0.***

__ __ __ HOURS

__ __ __ MINUTES

998 Se rehúsa a contestar (Minutes)

Q37. Desde que inició la epidemia de COVID-19 en Marzo 2020, ¿cambió su disponibilidad de agua? (escoge una) (Elija Uno)

0 No, es igual

1 Si, empeoró

2 Si, mejoró

8 Se rehúsa a contestar

Q38. En la última semana, ¿cuál fue su principal bebida (más común) cuando tuvo sed? (escoge una) (Elija Uno)

01	Agua
02	Agua mineral
03	Leche
04	Café
05	Té
06	Jugo/bebidas azucaradas
07	Soda
08	Bebidas alcohólicas
09	Ninguno
10	Otro
98	Se rehúsa a contestar

If Q38 is equal to 10 then skip to Q39

If not, then skip to Q40.

Q39. Por favor especifique cuál es su bebida principal.

__ __
__ __
__ __

Q40. En la última semana, ¿con qué frecuencia tuvo sed y no tenía agua? (escoge una) (Elija Uno)

1	Varias veces al día
2	Diario
3	Varias veces en la semana
4	Una vez
5	Nunca
8	Se rehúsa a contestar

Q41. En la última semana, ¿cuántos vasos de agua tomó por día? (escoge una) (Elija Uno)

01	1 vaso=250 ml/8 oz
02	2 vasos=500 ml/16 oz
03	3 vasos =750 ml/24 oz
04	4 vasos =1 ltr/32 oz
05	5 vasos =1.25 ltr/40 oz
06	6 vasos =1.5 ltr/48 oz
07	7 vasos =1.75 ltr/52oz
08	8 vasos =2 ltr/64 oz
09	9 vasos =2.25 ltr/72 oz
10	Más de 9 vasos por día
98	Se rehúsa a contestar

If Q41 is equal to 10 then skip to Q42

If not, then skip to Q43.

Q42. Por favor especifique cuántos vasos de agua tomó por día.

— —

98 Se rehúsa a contestar

Q43. En la última semana, ¿cuánto dinero gastó en agua para tomar? ***Entrevistador, por favor ingresa la cantidad.***

— — — — —

99998 Se rehúsa a contestar

If Q43 is not equal to 99998 and Q43 is not equal to 0 then skip to Q44

If not, then skip to Q45.

Q44. Entrevistador, por favor ingresa el **tipo de moneda.** (Elija Uno)

1 Dólares

2 Pesos

8 Se rehúsa a contestar

Q45. En los últimos 6 meses, ¿cuáles fuentes de agua utilizó para lavarse las manos?
Entrevistador escoja todas las que apliquen. (Marque Todas las que Correspondan)

__ Agua de la llave/potable privada (llave de casa)

__ Agua de la llave/potable privada (llave de vecino, familiar, amigo)

__ Agua de la llave pública (parque/gasolinera/afuera de tienda)

__ Agua de aspersores (parques públicos)

__ Pozo desprotegido

__ Pozo protegido

__ Fuente natural desprotegida

__ Fuente natural protegida

__ Agua de lluvia

__ Pipa de agua (camión)

__ Envasada (embotellada/en bolsita/de garrafón)

__ Agua superficial (río/lago/canal)

__ Tubos/hoyos dentro del canal del río Tijuana (lloraderos)

__ Sin recurso de agua

__ Otro

__ Se rehúsa a contestar

If Q45N is equal to 1 and Q45 is not equal to 98 and Q45 is greater than 1 then READ: "Solo puedes escoger la opcion "Sin recurso de agua" por si sola, verificar respuesta." and skip to Q45.

If Q45O is equal to 1 then skip to Q46

If not, then skip to Q47.

Q46. Usted mencionó que usa otra fuente de agua para lavarse las manos. Por favor especifique.

— —
— —
— —

Q47. ¿Cuál es su fuente principal de agua para lavarse las manos (la más común)?
ENTREVISTADOR: elija solo una. (Elija Uno)

01 Agua entubada privada (llave de casa)

02 Agua entubada privada (llave de vecino, familiar, amigo)

03 Agua entubada pública (parque/gasolinera/afuera de tienda)

04 Agua de aspersores (parques públicos)

05 Pozo desprotegido

06 Pozo protegido

07 Fuente natural desprotegida

08 Fuente natural protegida

09 Agua de lluvia

10 Pipa de agua (camión)

11 Envasada (embotellada/en bolsita/de garrafón)

12 Agua superficial (río/lago/canal)

13 Tubos/hoyos dentro del canal del río Tijuana (lloraderos)

14 Sin recurso de agua

15 Otro

98 Se rehúsa a contestar

If Q47 is equal to 15 then skip to Q48

If not, then skip to Q49.

Q48. Por favor especifique su fuente principal de agua para lavarse las manos.

____

Q49. ¿Con qué frecuencia está disponible para usted esta fuente (principal) de agua para el lavarse las manos? (Elija Uno)

1 Siempre

2 Usualmente

3 A veces

4 Raramente

5 Nunca

8 Se rehúsa a contestar

Q50. ¿Con qué frecuencia tiene jabón disponible para usted? (escoja uno) (Elija Uno)

1 Siempre

2 Usualmente

3 A veces

4 Raramente

5 Nunca

8 Se rehúsa a contestar

Q51. ¿Con qué frecuencia puede lavarse las manos con agua y jabón en un día promedio? (escoja uno) (Elija Uno)

1	Siempre
2	Usualmente
3	A veces
4	Raramente
5	Nunca
8	Se rehúsa a contestar

Q52. Desde que inició la epidemia de COVID-19, ¿cambió su disponibilidad de jabón? (escoja uno) (Elija Uno)

0	No, es lo mismo
1	Si, empeoro
2	Si, mejoro
8	Se rehúsa a contestar

Q53. ¿Con qué frecuencia está disponible el gel antibacterial a base de alcohol para usted? (escoja uno) (Elija Uno)

1	Siempre
2	Usualmente
3	A veces
4	Raramente
5	Nunca
8	Se rehúsa a contestar

Q54. En la última semana, ¿qué tan seguido lavó sus manos (con agua y jabón) después de hacer del baño (defecar)? (escoja uno) (Elija Uno)

1 Siempre

2 Usualmente

3 A veces

4 Raramente

5 Nunca

8 Se rehúsa a contestar

Q55. En la última semana, ¿qué tan seguido lavó sus manos (con agua y jabón) antes de comer? (escoja uno) (Elija Uno)

1 Siempre

2 Usualmente

3 A veces

4 Raramente

5 Nunca

8 Se rehúsa a contestar

Q56. En la última semana, ¿qué tan seguido lavó sus manos (con agua y jabón) antes de preparar comida? (escoja uno)   (Elija Uno)

1	Siempre
2	Usualmente
3	A veces
4	Raramente
5	Nunca
8	Se rehúsa a contestar

Q57. ¿Cuál es su fuente principal de agua para bañarse (la más común)? **ENTREVISTADOR: elija solo una.** (Elija Uno)

01	Agua entubada privada (llave de casa)
02	Agua entubada privada (llave de vecino, familiar, amigo)
03	Agua entubada pública (parque/gasolinera/afuera de tienda)
04	Agua de aspersores (parques públicos)
05	Pozo desprotegido
06	Pozo protegido
07	Fuente natural desprotegida
08	Fuente natural protegida
09	Agua de lluvia
10	Pipa de agua (camión)
11	Envasada (embotellada/en bolsita/de garrafón)
12	Agua superficial (río/lago/canal)
13	Tubos/hoyos dentro del canal del río Tijuana (lloraderos)
14	Sin recurso de agua
15	Otro
98	Se rehúsa a contestar

If Q57 is equal to 15 then skip to Q58

If not, then skip to Q59.

Q58. Por favor especifique qué fuente principal de agua utiliza para bañarse.

__ __
__ __
__ __

Q59. En la última semana, ¿cuántas veces se bañó/lavó? __ __

98 Se rehúsa a contestar

Q60. ¿Cuál es la principal fuente de agua que utiliza para limpiar heridas/abscesos? ***Entrevistador: elija solo una.*** (Elija Uno)

01	Agua de la llave/potable privada (llave de casa)
02	Agua de la llave/potable privada (llave de vecino, familiar, amigo)
03	Agua de la llave pública (parque/gasolinera/afuera de tienda)
04	Agua de aspersores (parques públicos)
05	Pozo desprotegido
06	Pozo protegido
07	Fuente natural desprotegida
08	Fuente natural protegida
09	Agua de lluvia
10	Pipa de agua (camión)
11	Envasada (embotellada/en bolsita/de garrafón)
12	Agua superficial (río/lago/canal)
13	Tubos/hoyos dentro del canal del río Tijuana (lloraderos)
14	Sin recurso de agua
15	Otro
98	Se rehúsa a contestar

If Q60 is equal to 15 then skip to Q61

If not, then skip to Q62.

Q61. Mencionó que usa otra fuente de agua para limpiar heridas/abscesos. Por favor especifique.

— —
— —
— —

Q62. ¿Cuál es su principal fuente de agua para preparar drogas para inyectarse? ***Entrevistador: elija solo una.*** (Elija Uno)

01	Agua entubada privada (llave de casa)
02	Agua entubada privada (llave de vecino, familiar, amigo)
03	Agua entubada pública (parque/gasolinera/afuera de tienda)
04	Agua de aspersores (parques públicos)
05	Pozo desprotegido
06	Pozo protegido
07	Fuente natural desprotegida
08	Fuente natural protegida
09	Agua de lluvia
10	Pipa de agua (camión)
11	Envasada (embotellada/en bolsita/de garrafón)
12	Agua superficial (río/lago/canal)
13	Tubos/hoyos dentro del canal del río Tijuana (lloraderos)
14	Sin recurso de agua
15	Otro
98	Se rehúsa a contestar

If Q62 is equal to 15 then skip to Q63

If not, then skip to Q64.

Q63. Mencionó que usa otra fuente de agua para preparar drogas para inyectarte. Por favor especifique cuál.

— —
— —
— —

Q64. En la última semana, ¿cuánto dinero gastó en higiene personal (jabón, agua, baños, lavandería, higiene menstrual)? ***Entrevistador, por favor ingresa la cantidad.***

— — — — —

99998 Se rehúsa a contestar

If Q64 is not equal to 99998 and Q64 is not equal to 0 then skip to Q65

If not, then skip to instruction before Q66.

Q65. Entrevistador, por favor ingresa el **tipo de moneda.** (Elija Uno)

1 Dólares

2 Pesos

8 Se rehúsa a contestar

If Q30 is equal to 2 or Q30 is equal to 3 then skip to Q66

If not, then skip to instruction before Q67.

Q66. ¿Alguna vez ha tenido períodos menstruales?

1 Si

0 No

8 Se rehúsa a contestar

If Q66 is equal to 1 then skip to Q67

If not, then skip to instruction before Q69.

Q67. ¿Hace cuánto tiempo fue su último periodo menstrual? ***Entrevistador, ingrese el número en el apartado correspondiente.***

__ __ __	AñOS
__ __ __	MESES
__ __ __	DíAS
997	No Sabe (Meses)
998	Se rehúsa a contestar (Meses)

Q68. Durante su último período menstrual, ¿qué productos de higiene femenina usó? ***Entrevistador, escoja todas las que apliquen.*** (Marque Todas las que Correspondan)

__	Trapo/tela
__	Toalla sanitaria reutilizable
__	Toalla sanitaria desechable
__	Tampones
__	Copa menstrual
__	Papel de baño
__	Ropa interior sola
__	Nada
__	Otro (especificar)
__	N/A (No tengo menstruación)
__	Se rehúsa a contestar

If Q68I is equal to 1 then skip to Q69

If not, then skip to Q70.

Q69. Por favor especifique qué materiales de higiene usó durante su último período menstrual.

— —
— —
— —

Q70. En los últimos 6 meses, ¿cuáles de estos tipo de baños/sanitarios usó? ***Entrevistador, Escoja todas las que apliquen*** (Marque Todas las que Correspondan)

__ Escusado conectado al drenaje/utilizando cubeta

__ Letrina de pozo con loza

__ Letrina de pozo sin loza/pozo abierto

__ Baño de compostaje

__ Baño portátil (contenedor)

__ Cubeta

__ Inodoro/letrina colgante

__ Ninguno (defecación al aire libre)

__ Otro

__ Se rehúsa a contestar

If Q70I is equal to 1 then skip to Q71

If not, then skip to Q72.

Q71. Por favor especifique qué otro tipo de baño/sanitario usó en los últimos 6 meses.

— —
— —
— —

Q72. De estos tipos de baños que mencionó, ¿Cuáles es el que más comúnmente usa? (escoge uno) (Elija Uno)

01	Escusado conectado al drenaje/utilizando cubeta
02	Letrina de pozo con loza
03	Letrina de pozo sin loza/pozo abierto
04	Baño de compostaje
05	Contenedor (baño portátil)
06	Cubeta
07	Inodoro/letrina colgante
08	Ninguno (defecación al aire libre/en la calle o callejón
09	Otro
98	Se rehúsa a contestar

If Q72 is equal to 9 then skip to Q73

If not, then skip to Q74.

Q73. Por favor especifique qué tipo de baño/sanitario usa más comúnmente.

— —
— —
— —

Q74. ¿Este baño/sanitario (principal) lo comparte con personas de otras viviendas que usted conoce o con el público en general? (Elija Uno) (Elija Uno)

1	Compartido con más de una vivienda que conozco
2	Público en general
3	No lo comparte
8	Se rehúsa a contestar
9	No Corresponde

Q75. ¿Con qué frecuencia este baño/sanitario (principal) está disponible (abierto y sin llave)? (Elija Uno) (Elija Uno)

1	Siempre
2	Usualmente
3	A veces
4	Raramente
5	Nunca
8	Se rehúsa a contestar
9	No Corresponde

Q76. ¿Con qué frecuencia este baño/sanitario (principal) funciona? (Elija Uno) (Elija Uno)

1 Siempre

2 Usualmente

3 A veces

4 Raramente

5 Nunca

8 Se rehúsa a contestar

9 No Corresponde

Q77. ¿Con qué frecuencia este baño/sanitario (principal) es privado? (Elija Uno) (Elija Uno)

1 Siempre

2 Usualmente

3 A veces

4 Raramente

5 Nunca

8 Se rehúsa a contestar

9 No Corresponde

Q78. ¿Cuánto tiempo se tarda en llegar caminando al baño/sanitario (principal)? (Entrevistador: si el participante indica que no tiene que caminar al baño/sanitario principal, escriba 0)

__ __ __ HOURS

__ __ __ MINUTES

998 Se rehúsa a contestar (Minutes)

999 No Corresponde (Minutes)

Q79. ¿Alguna vez ha experimentado acoso o violencia usando el baño (escusado)?

1 Si

0 No

8 Se rehúsa a contestar

9 No Corresponde

If Q79 is equal to 1 then skip to Q80

If not, then skip to instruction before Q81.

Q80. ¿Qué tipos de acoso o violencia ha sufrido cuando usa el baño (escusado)? ***Entrevistador, selecciona todas las que apliquen.*** (Marque Todas las que Correspondan)

__ Ataque físico

__ Ataque sexual

__ Ataque verbal

__ Robo

__ Arresto

__ Otro tipo de acoso/violencia

__ Se rehúsa a contestar

__ No Corresponde

If Q80F is equal to 1 then skip to Q81

If not, then skip to Q82.

Q81. Por favor especifique qué otros tipos de acoso o violencia ha sufrido usando el baño (escusado).

____

Q82. En la última semana, ¿cuántas veces hizo del baño (defecó) al aire libre?

__ __ __

998 Se rehúsa a contestar

Q83. En la última semana, ¿cuánto dinero gastó usando el baño/sanitario? ***Entrevistador, ingrese cantidad.***

__ __ __ __ __

99998 Se rehúsa a contestar

If Q83 is not equal to 99998 and Q83 is not equal to 0 then skip to Q84

If not, then skip to instruction before Q85.

Q84. Entrevistador, por favor ingresa el **tipo de moneda**. (Elija Uno)

1 Dólares

2 Pesos

8 Se rehúsa a contestar

READ: LEA AL PARTICIPANTE: El COVID-19 es una enfermedad nueva, causada por un coronavirus nuevo que no se había visto antes en los seres humanos. Le voy a hacer algunas preguntas sobre signos o síntomas que Ud. haya podido tener en las últimas 2 semanas. También le preguntaremos sobre sus actividades diarias, trabajo y su adaptación a la situación actual.

READ: LEA AL PARTICIPANTE: Ahora te vamos a preguntar sobre cómo el COVID-19 y los cambios en tu comunidad han afectado tu vida y tu salud.

Q85. ¿Qué está haciendo/hizo durante la pandemia de COVID-19 para protegerse a sí mismo y a otros? **(Entrevistador: Lea en voz alta el listado y marque todas las que apliquen).** (Marque Todas las que Correspondan)

__ Prac

__ Aisla

__ Cuid

__ Trab

__ No t

__ Segu

__ Cam

__ May

__ Cubr

__ Evita

__ No h

__ Se r

If Q85L is equal to 1 and Q85 is greater than 1 then READ: ""Ningún cambio en mi vida o mi comportamiento" no se puede seleccionar en conjunto con otras opciones. Lo regresaremos para que realice las correcciones necesarias." and skip to Q85.

Q86. ¿Qué tanto afecta/afectó la pandemia de COVID-19 a su vida cotidiana? (Escoja una) (Elija Uno)

1	De ningún modo
2	Un poco
3	Bastante
4	Muchísimo
5	Extremadamente
8	Se rehúsa a contestar

Q87. En las últimas 2 semanas, debido a la pandemia de COVID-19, yo (me) he: **(Entrevistador lea en voz alta y seleccione todos los que correspondan. Si ninguno aplica, ir a la siguiente pantalla)** (Marque Todas las que Correspondan)

__ Abastecido de alcohol

__ Abastecido de drogas ilícitas

__ Abastecido de suministros para la reducción de daños (agujas limpias, equipo de inyección)

__ Evitado compartir cigarrillos, porros o cigarrillos electrónicos / vaporizadores (más de lo habitual)

__ Evitado compartir pipas de crack, pipas de metanfetamina o bongas (más de lo habitual)

__ Evitado compartir bebidas con otros (más de lo habitual)

__ Evitado albergues

__ Evitados sitios de distribución de jeringas

__ Evitado recoger medicamentos para el trastorno por uso de opioides (MTUO)

__ Evitado recoger otros medicamentos

__ Evitado atención médica (por ejemplo, citas médicas, hospitales, clínicas de salud)

__ Dejar de fumar

__ Accedido a tratamientos para el trastorno por consumo de alcohol

__ Otro

__ Se rehúsa a contestar

If Q87N is equal to 1 then skip to Q88

If not, then skip to Q89.

Q88. Por favor especifique qué más ha hecho en las últimas 2 semanas, debido a la pandemia de COVID-19.

__ __

Q89. ¿Ha sido impactado tu acceso a comida en las últimas 2 semanas? (Escoge todas las que apliquen) (Marque Todas las que Correspondan)

__ No,

__ Si, n

__ Si, h

__ Si, n

__ Si, n

__ Se r

If Q89A is equal to 1 and Q89 is greater than 1 then READ: "Seleccionaste la opcion "No" y tambien la opcion "Si." Por favor regresar y revisar la respuesta." and skip to Q89.

If Q89F is equal to 1 then skip to Q90

If not, then skip to instruction before Q91.

Q90. Por favor, especifique de qué otra forma ha sido afectado su acceso a los alimentos.

__ __
__ __
__ __

SARSCoV-2 COMPONENT: Inseguridad Alimentaria de la Encuesta de Inseguridad Alimentaria para Adultos del USDA

READ: LEA AL PARTICIPANTE: Ahora le voy a leer algunas declaraciones que las personas han hecho sobre situaciones alimentarias. Para estas declaraciones, por favor dígame si fue cierto a menudo, a veces, o nunca fue cierto (para Ud./las personas con quien vive), eso es desde que la pandemia de COVID-19 comenzó en Marzo 2020.

Q91. La primera declaración es: Me preocupaba que (mi/nuestra) comida se pudiera acabar antes de que tuviera dinero para comprar más, ¿Esto fue cierto a menudo, a veces, o nunca fue cierto para usted desde que comenzó la pandemia de COVID en Marzo de 2020? (Escoja una) (Elija Uno)

1 Fue sierto a menudo

2 A veces fue sierto

3 Nunca fue sierto

8 Se rehúsa a contestar

Q92. La comida que compré no duró lo suficiente, y no tenía dinero para comprar más. (Elija Uno)

1 Fue sierto a menudo

2 A veces fue sierto

3 Nunca fue sierto

8 Se rehúsa a contestar

Q93. No tenía recursos suficientes para comer comida variada y nutritiva. (Elija Uno)

1 Fue sierto a menudo

2 A veces fue sierto

3 Nunca fue sierto

8 Se rehúsa a contestar

Q94. Desde que comenzó la pandemia de COVID en Marzo de 2020, ¿Usted redujo alguna vez la cantidad de sus comidas o tuvo que dejar de comer porque le faltaba dinero para alimentos?

1 Si

0 No

8 Se rehúsa a contestar

If Q94 is equal to 1 then skip to Q95

If not, then skip to Q96.

Q95. ¿Con qué frecuencia sucedió esto? (Escoja una) (Elija Uno)

1 Casi todos los meses

2 Algunos meses pero no todos

3 Solamente en 1 ó 2 meses

8 Se rehúsa a contestar

Q96. Desde que comenzó la pandemia de COVID en Marzo de 2020, ¿comió Ud. alguna vez menos de lo que pensaba que debía comer porque le faltaba dinero para alimentos?

1 Si

0 No

8 Se rehúsa a contestar

Q97. Desde que comenzó la pandemia de COVID en Marzo de 2020, ¿Tuvo Ud. hambre alguna vez pero no comió porque le faltaba dinero para alimentos?

1 Si

0 No

8 Se rehúsa a contestar

Q98. Desde que comenzó la pandemia de COVID en Marzo de 2020, ¿Perdió Ud. peso porque no comió los alimentos suficientes por falta de dinero para comida?

1 Si

0 No

8 Se rehúsa a contestar

Q99. Desde que comenzó la pandemia de COVID en Marzo de 2020, ¿alguna vez no comió Ud. en todo el día porque le faltaba dinero para comida?

1 Si

0 No

8 Se rehúsa a contestar

If Q99 is equal to 1 then skip to Q100

If not, then skip to Q101.

Q100. ¿Con qué frecuencia sucedió esto? (escoja uno) (Elija Uno)

1 Casi todos los meses

2 Algunos meses pero no todos

3 Solamente en 1 ó 2 meses

8 Se rehúsa a contestar

Q101. ¿Sigue alguna dieta especial (por ejemplo: baja en colesterol, vegana, vegetariana, paleo, para bajar de peso, orgánica, etc.)?

1 Si

0 No

8 Se rehúsa a contestar

If Q101 is equal to 1 then skip to Q102

If not, then skip to Q103.

Q102. Por favor especifique qué dieta.

____

Q103. ¿Tiene Ud. alguna de las siguientes condiciones crónicas
Entrevistador: Si ninguna de las opciones corresponden, no indique nada y siga con la siguiente pantalla. (Marque Todas las que Correspondan)

__ Alergias de temporada

__ Asma u otros problemas pulmonares

__ Problemas cardíacos

__ Problemas renales

__ Desórdenes inmunológicos

__ Diabetes o niveles elevados de azúcar en sangre

__ Cáncer

__ Artritis

__ Dolores de cabeza fuertes o frecuentes

__ Epilepsia o ataques

__ Problemas de intestinos o estomacales serios

__ Problemas de acné o de piel serios

__ Hipertensión

__ Enfermedad Crónica obstructiva Pulmonar (EPOC)

__ Congestión Cardíaca

__ Fibrilacion Atrial o Flutter Atrial

__ Enfermedad Cardíaca/Infarto de Miocardio

__ Derrame cerebral

__ Cualquier enfermedad autoinmune

__ VIH

__ Trombósis de vena profunda

__ Embolismo pulmonar

__ Se rehúsa a contestar

Q104. ¿Ha tomado antibióticos en los últimos 6 meses?

1 Si

0 No

8 Se rehúsa a contestar

If Q104 is equal to 1 then skip to Q105

If not, then skip to Q106.

Q105. ¿Por favor especifique para qué problema de salud está tomando antibióticos?

____

Q106. ¿Ha estado expuesto a alguien que probablemente tenga Coronavirus/COVID-19? (Marque Todas las que Correspondan)

__ Si, alguien que tuvo una prueba positiva

__ Si, alguien con un diagnóstico de médico pero sin prueba

__ Si, alguien con posibles síntomas, pero sin diagnóstico de doctor

__ No

__ Se rehúsa a contestar

If Q106D is equal to 1 and Q106A + Q106B + Q106C is greater than 0 then READ: "Solo puedes escoger la opcion "No" por si sola, verificar respuesta." and skip to Q106.

Q107. ¿De dónde obtiene Ud. información sobre COVID19/el coronavirus? **(Entrevistador: indique todas las que correspondan, si no se indica ninguna, siga con la siguiente pantalla)** (Marque Todas las que Correspondan)

__ Amistades

__ Doctores/Profesionales de la salud

__ Radio/TV Conservadora (Fox, Red Una América, El Informe Drudge)

__ Radio/TV Liberal (CNN, MSNBC, Huffington Post, NPR)

__ Medios sociales (Facebook, YouTube, Twiter)

__ Se rehúsa a contestar

Q108. ¿Cuál de estas fuentes de información sobre COVID19 e más importante para Ud? (Elija Uno)

1 Amistades

2 Doctores/Profesionales de la salud

3 Radio/TV Conservadora (Fox, Red Una América, El Informe Drudge)

4 Radio/TV Liberal (CNN, MSNBC, Huffington Post, NPR)

5 Medios sociales (Facebook, YouTube, Twiter)

8 Se rehúsa a contestar

READ: Ahora le voy a leer algunas oraciones sobre COVID-19. Por favor quiero que me diga si a ud. le parece que la oración es verdadera, falsa o si no está seguro.

Q109. La industria farmacéutica fue quien creó el virus COVID-19. (Elija Uno)

1 Verdadero

2 Falso

3 No estoy seguro

8 Se rehúsa a contestar

Q110. El coronavirus fué creado por el gobierno Chino como un arma biológica. (Elija Uno)

1 Verdadero

2 Falso

3 No estoy seguro

8 Se rehúsa a contestar

Q111. Algunas personas en el gobierno de EUA están exagerando el riesgo que representa el COVID19. (Elija Uno)

1 Verdadero

2 Falso

3 No estoy seguro

8 Se rehúsa a contestar

Q112. Las vacunas que se le dan a los niños para enfermedades como la varicela y paperas causan autismo. (Elija Uno)

1 Verdadero

2 Falso

3 No estoy seguro

8 Se rehúsa a contestar

Q113. El virus que causa el COVID19 se puede transmitir fácilmente de una persona a otra. (Elija Uno)

1 Verdadero

2 Falso

3 No estoy seguro

8 Se rehúsa a contestar

Q114. Muchos miles de personas han fallecido por COVID19. (Elija Uno)

1 Verdadero

2 Falso

3 No estoy seguro

8 Se rehúsa a contestar

Q115. Se encuentra disponible una vacuna para prevenir el COVID19. (Elija Uno)

1 Verdadero

2 Falso

3 No estoy seguro

8 Se rehúsa a contestar

Q116. La mayoría de las personas ya tienen immunidad contra el COVID19. (Elija Uno)

1 Verdadero

2 Falso

3 No estoy seguro

8 Se rehúsa a contestar

Q117. Uno se puede dar cuenta si alguien tiene COVID19 con solo mirarlo. (Elija Uno)

1 Verdadero

2 Falso

3 No estoy seguro

8 Se rehúsa a contestar

Q118. Existen tratamientos efectivos para COVID19 que pueden curar a la mayoría de las personas. (Elija Uno)

1 Verdadero

2 Falso

3 No estoy seguro

8 Se rehúsa a contestar

Q119. El tener COVID19 es tan peligroso como el tener la gripa. (Elija Uno)

1 Verdadero

2 Falso

3 No estoy seguro

8 Se rehúsa a contestar

Q120. Las vacunas para el COVID que le ofrecen a personas como yo no son tan seguras como las otras vacunas para el COVID. (Elija Uno)

1 Verdadero

2 Falso

3 No estoy seguro

8 Se rehúsa a contestar

Q121. Las vacunas para el COVID tienen un dispositivo de rastreo. (Elija Uno)

1 Verdadero

2 Falso

3 No estoy seguro

8 Se rehúsa a contestar

Q122. Las vacunas para el COVID son seguras para las mujeres embarazadas. (Elija Uno)

1 Verdadero

2 Falso

3 No estoy seguro

8 Se rehúsa a contestar

Q123. Algunas vacunas para el COVID pueden cambiar mi ADN. (Elija Uno)

1 Verdadero

2 Falso

3 No estoy seguro

8 Se rehúsa a contestar

Q124. Si le ofrecieran una prueba rápida por hisopo nasal para el COVID hoy día y que le daría sus resultados en 20 minutos, ¿querría Ud. que se la hicieran? (Elija Uno)

0 No

1 Si

2 No estoy seguro

7 No Sabe

8 Se rehúsa a contestar

If Q124 is equal to 1 and Q15 is equal to 1 then skip to Q125

If not, then skip to Q126.

Q125. ¿Estaría Ud. dispuesto a hacerse la prueba si el gobierno de San Diego le exigiera que se reporte con su nombre el resultado de su prueba?

1	Si
0	No
7	No Sabe
8	Se rehúsa a contestar
9	No Corresponde

Q126. ¿Cree Ud. que ya le dió COVID-19? (Elija Uno)

1	Si
2	No
3	No estoy seguro
8	Se rehúsa a contestar

Q127. En una escala del 1 al 10, qué tan preocupada está de infectarse con COVID-19 (o de re-infectarse)? (Nota: 1 es no estar para nada preocupadad, y 10 es estar demasiado preocupada.) **Entrevistador, por favor haga la pregunta aún cuando el participante le diga que ya tuvieron COVID o si ya se pusieron la vacuna.**

01	Low
02	
03	
04	
05	
06	
07	
08	
09	
10	High
98	Se rehúsa a contestar

If Q126 is equal to 1 then skip to Q128

If not, then skip to Q129.

Q128. ¿Cuánto hace que Ud. cree que le dió COVID-19? __ __ MESES

__ __ WEEKS

__ __ DíAS

98 Se rehúsa a contestar (Meses)

Q129. ¿Se ha hecho la prueba para COVID-19 (aparte de este estudio)? (Elija Uno)

1 Sí, me hice una prueba

2 No, no me han realizado la prueba

3 Traté de hacerme la prueba pero no pude

4 No estoy seguro/a si me han hecho prueba para COVID-19

8 Se rehúsa a contestar

If Q129 is equal to 1 then skip to Q130

If not, then skip to Q158.

Q130. ¿Cuántas veces se ha hecho la prueba para COVID-19 fuera de nuestro estudio?

__ __

98 Se rehúsa a contestar

Q131. Desde su última visita, ¿ en qué país/es se hizo la prueba para COVID-19t? **(Entrevistador, marque todas las que correspondan)** (Marque Todas las que Correspondan)

__ EUA

__ México

__ Otro país

__ Se rehúsa a contestar

Q132. Cuando se hizo la prueba para COVID-19 fuera de nuestro estudio, ¿qué tipo de prueba fué? **(Marque todas las que correspondan)** (Marque Todas las que Correspondan)

__ Prueba de hisopado nasal

__ Prueba de sangre (de anticuerpos)

__ Saliva

__ Otro tipo de prueba para COVID-19

__ Se rehúsa a contestar

If Q132D is equal to 1 then skip to Q133

If not, then skip to Q134.

Q133. Por favor especifique qué otro tipo de prueba para COVID-19 se hizo.

__ __

Q134. Dónde se hizo la prueba de COVID-19, fuera de nuestro estudio? **(Entrevistador, lea en voz alta las opciones y marque todas las que correspondan)** (Marque Todas las que Correspondan)

__ Consultorio de un doctor

__ Clínica/Centro de Salud de la Comunidad

__ Programa de Intercambio de Jeringas

__ Proyecto de Investigación (distinto de nuestro proyecto)

__ Kit de prueba rapida en el hogar autoadministrada

__ Otro

__ Se rehúsa a contestar

If Q134F is equal to 1 then skip to Q135

If not, then skip to Q136.

Q135. Por favor especifique dónde se hizo la prueba para COVID-19 desde su última visita.

__ __

Q136. ¿Cuándo fue la última vez que se hizo la prueba de COVID-19 fuera de nuestro estudio?

__ __ / __ __ / __ __ __ __	dd / mm / aaaa
2097	No Sabe (Año)
2098	Se rehúsa a contestar (Año)

Q137. ¿Le dieron un resultado positivo para al COVID-19 fuera de nuestro estudio? (Escoja una) (Elija Uno)

1	Sí, obtuve resultado positivo
2	No, obtuve resultado negativo
3	Me realizaron la prueba, pero no obtuve el resultado
4	Desconoce/no entendió el resultado
8	Se rehúsa a contestar

If Q137 is equal to 1 then skip to Q138

If not, then skip to instruction before Q139.

Q138. ¿Cuándo fue la última vez que se hizo la prueba de COVID-19 fuera de nuestro estudio?

__ __ / __ __ / __ __ __ __	dd / mm / aaaa
2097	No Sabe (Año)
2098	Se rehúsa a contestar (Año)

If Q126 is equal to 1 or Q137 is equal to 1 then skip to Q139

If not, then skip to Q158.

Q139. Cuando Ud. tuvo o Ud. creyó que tuvo COVID, ¿cuál de los siguientes síntomas tuvo Ud.? (Indique todos los que correspondan) (Marque Todas las que Correspondan)

__ Fiebre de > 100.4F (38C)

__ Fiebre subjetiva (se sintió afiebrado, sin confirmar)

__ Fiebre moderada o escalofrios

__ Sudores nocturnos (que mojas las sábanas como 3 o más veces por semana)

__ Estremecimientos reiterados con escalofríos

__ Nariz escurridiza

__ Tos

__ Dolor de garganta

__ Carraspera

__ Falta de respiración

__ Nausea o vómito

__ Dolor de cabeza

__ Dolor abdominal

__ Diarrea (tres o más evacuaciones sueltas o más sueltas de lo normal en un período de 24 horas)

__ Perdida repentina del olfato

__ Perdida repentina del sabor

__ Dolor de articulaciones

__ Fatiga/cansancio extremo

__ Se rehúsa a contestar

If Q139A is equal to 1 then skip to Q140

If not, then skip to instruction before Q141.

Q140. ¿Por cuánto tiempo ha tenido Fiebre >100.4F (38C)? (Elija Uno)

1	Menos de 8 días
2	8-13 días
3	14-20 días
4	Más de 20 días
7	No Sabe
8	Se rehúsa a contestar

If Q139B is equal to 1 then skip to Q141

If not, then skip to instruction before Q142.

Q141. ¿Por cuánto tiempo ha tenido Fiebre subjetiva? (Elija Uno)

1	Menos de 8 días
2	8-13 días
3	14-20 días
4	Más de 20 días
7	No Sabe
8	Se rehúsa a contestar

If Q139C is equal to 1 then skip to Q142

If not, then skip to instruction before Q143.

Q142. ¿Por cuánto tiempo ha tenido Fiebre moderada o escalofríos? (Elija Uno)

1 Menos de 8 días

2 8-13 días

3 14-20 días

4 Más de 20 días

7 No Sabe

8 Se rehúsa a contestar

If Q139D is equal to 1 then skip to Q143

If not, then skip to instruction before Q144.

Q143. ¿Por cuánto tiempo ha tenido Sudoración nocturna? (Elija Uno)

1 Menos de 8 días

2 8-13 días

3 14-20 días

4 Más de 20 días

7 No Sabe

8 Se rehúsa a contestar

If Q139E is equal to 1 then skip to Q144

If not, then skip to instruction before Q145.

Q144. ¿Por cuánto tiempo ha tenido Estremecimientos reiterados con escalofríos? (Elija Uno)

1 Menos de 8 días

2 8-13 días

3 14-20 días

4 Más de 20 días

7 No Sabe

8 Se rehúsa a contestar

If Q139F is equal to 1 then skip to Q145

If not, then skip to instruction before Q146.

Q145. ¿Por cuánto tiempo ha tenido Nariz escurridiza? (Elija Uno)

1 Menos de 8 días

2 8-13 días

3 14-20 días

4 Más de 20 días

7 No Sabe

8 Se rehúsa a contestar

If Q139G is equal to 1 then skip to Q146

If not, then skip to instruction before Q147.

Q146. ¿Por cuánto tiempo ha tenido Tos? (Elija Uno)

1 Menos de 8 días

2 8-13 días

3 14-20 días

4 Más de 20 días

7 No Sabe

8 Se rehúsa a contestar

If Q139H is equal to 1 then skip to Q147

If not, then skip to instruction before Q148.

Q147. ¿Por cuánto tiempo ha tenido Dolor de garganta? (Elija Uno)

1	Menos de 8 días
2	8-13 días
3	14-20 días
4	Más de 20 días
7	No Sabe
8	Se rehúsa a contestar

If Q139I is equal to 1 then skip to Q148

If not, then skip to instruction before Q149.

Q148. ¿Por cuánto tiempo ha tenido Carraspera? (Elija Uno)

1	Menos de 8 días
2	8-13 días
3	14-20 días
4	Más de 20 días
7	No Sabe
8	Se rehúsa a contestar

If Q139J is equal to 1 then skip to Q149

If not, then skip to instruction before Q150.

Q149. ¿Por cuánto tiempo ha tenido Falta de respiración? (Elija Uno)

1 Menos de 8 días

2 8-13 días

3 14-20 días

4 Más de 20 días

7 No Sabe

8 Se rehúsa a contestar

If Q139K is equal to 1 then skip to Q150

If not, then skip to instruction before Q151.

Q150. ¿Por cuánto tiempo ha tenido Nausea o vómito? (Elija Uno)

1 Menos de 8 días

2 8-13 días

3 14-20 días

4 Más de 20 días

7 No Sabe

8 Se rehúsa a contestar

If Q139L is equal to 1 then skip to Q151

If not, then skip to instruction before Q152.

Q151. ¿Por cuánto tiempo ha tenido Dolor de cabeza? (Elija Uno)

1 Menos de 8 días

2 8-13 días

3 14-20 días

4 Más de 20 días

7 No Sabe

8 Se rehúsa a contestar

If Q139M is equal to 1 then skip to Q152

If not, then skip to instruction before Q153.

Q152. ¿Por cuánto tiempo ha tenido Dolor abdominal? (Elija Uno)

1 Menos de 8 días

2 8-13 días

3 14-20 días

4 Más de 20 días

7 No Sabe

8 Se rehúsa a contestar

If Q139N is equal to 1 then skip to Q153

If not, then skip to instruction before Q154.

Q153. ¿Por cuánto tiempo ha tenido Diarrea? (Elija Uno)

1 Menos de 8 días

2 8-13 días

3 14-20 días

4 Más de 20 días

7 No Sabe

8 Se rehúsa a contestar

If Q139O is equal to 1 then skip to Q154

If not, then skip to instruction before Q155.

Q154. ¿Por cuánto tiempo ha tenido Perdida repentina del olfato? (Elija Uno)

1 Menos de 8 días

2 8-13 días

3 14-20 días

4 Más de 20 días

7 No Sabe

8 Se rehúsa a contestar

If Q139P is equal to 1 then skip to Q155

If not, then skip to instruction before Q156.

Q155. ¿Por cuánto tiempo ha tenido Perdida repentina del sabor? (Elija Uno)

1	Menos de 8 días
2	8-13 días
3	14-20 días
4	Más de 20 días
7	No Sabe
8	Se rehúsa a contestar

If Q139Q is equal to 1 then skip to Q156

If not, then skip to instruction before Q157.

Q156. ¿Por cuánto tiempo ha tenido Dolor de articulaciones? (Elija Uno)

1	Menos de 8 días
2	8-13 días
3	14-20 días
4	Más de 20 días
7	No Sabe
8	Se rehúsa a contestar

If Q139R is equal to 1 then skip to Q157

If not, then skip to Q158.

Q157. ¿Por cuánto tiempo ha tenido Fatiga/cansancio extremo? (Elija Uno)

1 Menos de 8 días

2 8-13 días

3 14-20 días

4 Más de 20 días

7 No Sabe

8 Se rehúsa a contestar

Q158. ¿Lo han hospitalizado por COVID-19? (Elija Uno)

0 No

1 Si, he sido hospitalizado

8 Se rehúsa a contestar

If Q158 is equal to 1 then skip to Q159

If not, then skip to Q160.

Q159. ¿Cuándo fue hospitalizado por COVID-19 la última vez?

__ __ / __ __ / __ __ __ __ dd / mm / aaaa

2097 No Sabe (Año)

Q160. ¿Cuántas personas conoce Ud. que han fallecido de COVID-19?

__ __ __

998 Se rehúsa a contestar

Q161. ¿Cuántas personas conoce que se han puesto la vacuna para el COVID-19?

__ __ __ __

9998 Se rehúsa a contestar

Q162. ¿Alguna vez en su vida ha recibido una vacuna para la gripa?

1 Si

0 No

7 No Sabe

8 Se rehúsa a contestar

Q163. ¿Le han ofrecido una vacuna contra el COVID19? (Elija Uno)

1 Si

2 No

3 No estoy seguro

8 Se rehúsa a contestar

Q164. ¿Ya le han puesto unaa vacuna contra el COVID19? (Elija Uno)

1 Si

2 No

3 No estoy seguro-a

8 Se rehúsa a contestar

If Q164 is equal to 1 then skip to Q165

If not, then skip to instruction before Q172.

Q165. ¿En qué país recibió la vacuna de COVID-19? (Elija Uno)

1 EUA

2 México

3 Otro país

8 Se rehúsa a contestar

If Q165 is equal to 3 then skip to Q166

If not, then skip to Q167.

Q166. Por favor, especifique en qué otro país recibió la vacuna para el COVID-19.

____

Q167. ¿Cuál vacuna contra el COVID19 le pusieron? (Elija Uno)

01	Moderna
02	Pfizer
03	Johnson y Johnson
04	Astra Zeneca
05	CanSino
06	Sinovac
07	Sputnik
08	Otra
97	No Sabe
98	Se rehúsa a contestar

If Q167 is equal to 8 then skip to Q168

If not, then skip to Q169.

Q168. Por favor, especifique la vacuna para el COVID-19.

__ __

Q169. ¿Cuántas dósis de la vacuna le pusieron ya? (Elija Uno)

1	Una
2	Dos
3	Me olvidé cuántas
8	Se rehúsa a contestar

Q170. ¿Cuándo recibió la primera inyección de la vacuna COVID-19?

__ __ / __ __ / __ __ __ __	dd / mm / aaaa
2097	No Sabe (Año)
2098	Se rehúsa a contestar (Año)

If Q169 is equal to 2 then skip to Q171

If not, then skip to instruction before Q172.

Q171. ¿Cuándo recibió la segunda inyección de la vacuna COVID-19?

__ __ / __ __ / __ __ __ __	dd / mm / aaaa
2097	No Sabe (Año)
2098	Se rehúsa a contestar (Año)

If Q164 is not equal to 1 then skip to Q172

If not, then skip to Q186.

Q172. ¿Ha tratado de ponerse la vacuna para el COVID pero no lo ha logrado?

1	Si
0	No
8	Se rehúsa a contestar

If Q172 is equal to 1 then skip to Q173

If not, then skip to instruction before Q174.

Q173. ¿Qué tipo de problemas ha tenido al tratar de ponerse la vacuna para el COVID?
(Entrevistador, lea la lista en voz alta e indiquen todas las que correspondan)
(Marque Todas las que Correspondan)

__ No sé a dónde ir

__ Demasiado lejos/Problemas de transporte

__ No tenía una cita

__ No tengo acceso a un teléfono o computadora para hacer la cita

__ No me gusta el lugar o las personas en el lugar donde estann ofreciendo la vacuna

__ La línea estab muy larga

__ El personal me maltrató/me trató mal

__ No reunía los requisitos (p. ej., edad, condiciones pre-existentes

__ No tengo seguranza/dinero

__ No tenía identificación, ID

__ Solamente ofrecían CanSino, Sinovac o Sputnik

__ Otra

__ Se rehúsa a contestar

If Q173L is equal to 1 then skip to Q174

If not, then skip to Q175.

Q174. Especifique qué otro tipo de problemas tuvo al tratar de conseguir ponerse la vacuna para el COVID?

____

Q175. Si le ofrecieran una vacuna gratis para prevenir/evitar que se infecte con COVID 19 o que se vuelva a infectar, ¿estaría de acuerdo en recibirla/ponérsela? (Elija Uno)

1	Si
2	No
3	No estoy seguro
8	Se rehúsa a contestar

If Q175 is equal to 2 or Q175 is equal to 3 then skip to Q176

If not, then skip to Q186.

Q176. ¿Pro qué no querría recibir/ponerse una vacuna para evitar/prevenir que se infecte con COVID-19 (o que se vuelva a infectar)? (Marque Todas las que Correspondan)

__ No me gustan las vacunas/jeringas

__ Tengo alergias a las vacunas

__ No me preocupa que me vaya a enfermar mucho con el COVID-19

__ Pienso que las vacunas no funcionan muy bien

__ No confío que las vacunas sean seguras

__ No creo que la pandemia del COVID-19 sea tan mal como dice la gente

__ No quiero tener que pagar por la vacuna

__ No sé lo suficiente sobre cómo funciona la vacuna contra el COVID-19

__ Me preocupa que me vaya a infectar con COVID-19 por la vacuna

__ No me preocupa mi salud

__ De todas maneras me voy a infectar con COVID-19/mi destino

__ Me preocupa que la prueba de COVID me salga positiva por la vacuna

__ Toma demasiado tiempo/Está muy lejos

__ Me preocupan los efectos secundarios de la vacuna/que me haga sentir enfermo

__ Ya me dió COVID y no creo que me vuelva a dar

__ Tengo un sistema de defensas muy fuerte/ Creo que soy inmune

__ Estoy tomando vitaminas y remedios naturales para protegerme de infectarme con COVID-19

__ Me preocupa que me vaya a infectar porque tengo condiciones de salud pre-existentes

__ Otra

__ Se rehúsa a contestar

If Q176S is equal to 1 then skip to Q177

If not, then skip to Q178.

Q177. Por favor especifique por qué Ud. no querría recibir/ponerse la vacuna para evitar/prevenir infectarse con COVID-19.

— —
— —
— —

Q178. ¿A cuál de los siguientes lugares estaría Ud. dispuesto a asistir para obtener/recibir gratuitamente la vacuna para el COVID-19? **(Marque todas las que correspondan)** (Marque Todas las que Correspondan)

__ Consultorio de un doctor

__ Clínica/Centro de Salud de la Comunidad

__ Farmacia

__ Cárcel/Prisión/CERESO (si estuviera privado de la libertad

__ Unidad móvil del Programa de Intercambio de Jeringas

__ Otra unidad móvil

__ Albergue

__ Programa de comidas

__ Centro de servicios

__ Iglesia/Templo

__ Feria de Salud

__ Otro

__ Estaría dispuesto a ir a cualquier lugar

__ Se rehúsa a contestar

If Q178M is equal to 1 and Q178 is greater than 1 then READ: "No se puede elegir la opción "Estoy dispuesto a ir a cualquier lugar" junto con cualquiera de las otras opciones. Por favor verifique con el participante y actualice su respuesta." and skip to Q178.

If Q178L is equal to 1 then skip to Q179

If not, then skip to Q180.

Q179. Por favor especifique a qué otro lugar/es estaría dispuesto a ir para recibir/ponerse la vacuna gratuita para el COVID-19?

__ __
__ __
__ __

Q180. ¿Cuánto tiempo estaría Ud. dispuesto a dedicar de su tiempo para obtener/ponerse la vacuna gratuita para el COVID-19? (Elija Uno)

0 Nada de mi tiempo (ni un minuto)

1 Menos de 1 hora

2 De 1 a 3 horas

3 Cualquier cantidad de tiempo

8 Se rehúsa a contestar

Q181. ¿Cuál de lo siguiente estaría dispuesto a hacer para obtener/ponerse gratuitamente la vacuna para el COVID-19? (Marque Todas las que Correspondan)

__ Si fuera una dósis en lugar de 2

__ Si pudiera inhalar/tragar la vacuna

__ Si la pudiera conseguir en un lugar cerca de donde vivo/me la llevo

__ Si la pudiera conseguir en algún lugar donde ya voy

__ Si mis amistades ya se la hubieran puesto

__ Si alguien en quien confío me dijera que es segura

__ Otra

__ Nada me podría convencer de ponermela

__ Se rehúsa a contestar

If Q181H is equal to 1 and Q181 is greater than 1 then READ: "No se puede usar la opción "Nada podría convencerme de ponerme la vacuna" junto con cualquiera de las otras opciones. por favor revise con el participante y actualice la respuesta." and skip to Q181.

Q182. ¿Estaría Ud. más o menos dispuesto a ponerse la vacuna del COVID si Ud. recibiera $10 dólares? (Elija Uno)

1 Más probablemente

2 Menos probablemente

3 No haría diferencia

8 Se rehúsa a contestar

Q183. ¿Estaría Ud. más o menos dispuesto a ponerse la vacuna del COVID si Ud. recibiera $25 dólares? (Elija Uno)

1 Más probablemente

2 Menos probablemente

3 No haría diferencia

8 Se rehúsa a contestar

Q184. ¿Estaría Ud. más o menos dispuesto a ponerse la vacuna del COVID si Ud. recibiera $50 dólares? (Elija Uno)

1 Más probablemente

2 Menos probablemente

3 No haría diferencia

8 Se rehúsa a contestar

Q185. ¿Estaría Ud. más o menos dispuesto a ponerse la vacuna del COVID si Ud. recibiera $100 dólares? (Elija Uno)

1 Más probablemente

2 Menos probablemente

3 No haría diferencia

8 Se rehúsa a contestar

Q186. En caso que le salga positiva la prueba para el COVID-19, ¿se prodrá aislar?

1	Si
0	No
7	No Sabe
8	Se rehúsa a contestar

Q187. En caso que Ud estuviera expuesto a alguien con COVID-19, ¿se podría poner Ud. en cuarentena (es decir, separase Ud mismo de las demás personas por 10 días)?

1	Si
0	No
7	No Sabe
8	Se rehúsa a contestar

READ: Hasta qué punto está de acuerdo con las dos siguientes oraciones:

Q188. Yo sé dónde puedo conseguir prueba para el COVID-19 en mi comunidad. (Elija Uno)

0	Totalmente en desacuerdo
1	En desacuerdo
2	Ni en desacuerdo ni de acuerdo
3	De acuerdo
4	Totalmente de acuerdo
8	Se rehúsa a contestar

Q189. Es fácil conseguir hacerse la prueba para el COVID-19. (Elija Uno)

0 Totalmente en desacuerdo

1 En desacuerdo

2 Ni en desacuerdo ni de acuerdo

3 De acuerdo

4 Totalmente de acuerdo

8 Se rehúsa a contestar

Q190. Si me dan un resultado negativo a la prueba, quiere decir...**(Entrevistador lea las opciones al participante en voz alta e indique todas las que correspondan)** (Marque Todas las que Correspondan)

__ No me tengo que preocupar de infectarme con COVID-19

__ No tengo COVID-19 ahora

__ Puedo estar con otros sin preocuparme de contagiarlos con COVID-19

__ Puedo estar con otros sin preocuparme que me vayan a infectar con COVID-19

__ Se rehúsa a contestar

READ: LEA: Ahora tengo algunas preguntas sobre tu salud. Recuerda que toda la información que tu me proporciones es privada y no se compartirá fuera del estudio. Empezaremos con una serie de signos y síntomas, te preguntaré si actualmente lo/los tienes, de ser así, por cuánto tiempo lo/s has tenido.

Q191. Actualmente, ¿tienes Fiebre de > 100.4F (38C)?

1 Si

0 No

7 No Sabe

8 Se rehúsa a contestar

If Q191 is equal to 1 then skip to Q192

If not, then skip to Q193.

Q192. ¿Cuánto tiempo la has tenido? (escoja uno) (Elija Uno)

1 Menos de 8 días

2 8-13 días

3 14-20 días

4 Más de 20 días

7 No Sabe

8 Se rehúsa a contestar

Q193. Actualmente, ¿tienes fiebre subjetiva (se sintió afiebrado, sin confirmar)?

1 Si

0 No

7 No Sabe

8 Se rehúsa a contestar

If Q193 is equal to 1 then skip to Q194

If not, then skip to Q195.

Q194. ¿Cuánto tiempo la has tenido? (escoja uno) (Elija Uno)

1 Menos de 8 días

2 8-13 días

3 14-20 días

4 Más de 20 días

7 No Sabe

8 Se rehúsa a contestar

Q195. Actualmente, ¿tienes fiebre moderada o escalofrios?

1 Si

0 No

7 No Sabe

8 Se rehúsa a contestar

If Q195 is equal to 1 then skip to Q196

If not, then skip to Q197.

Q196. ¿Cuánto tiempo lo has tenido? (escoja uno) (Elija Uno)

1	Menos de 8 días
2	8-13 días
3	14-20 días
4	Más de 20 días
7	No Sabe
8	Se rehúsa a contestar

Q197. ¿Tienes**: sudores nocturnos (que mojas las sábanas como 3 o más veces por semana?**

1	Si
0	No
7	No Sabe
8	Se rehúsa a contestar

If Q197 is equal to 1 then skip to Q198

If not, then skip to Q199.

Q198. ¿Cuánto tiempo lo has tenido? (escoja uno) (Elija Uno)

1	Menos de 8 días
2	8-13 días
3	14-20 días
4	Más de 20 días
7	No Sabe
8	Se rehúsa a contestar

Q199. ¿Tienes**: Temblores repetitivos con escalofríos?**	1	Si
	0	No
	7	No Sabe
	8	Se rehúsa a contestar

If Q199 is equal to 1 then skip to Q200

If not, then skip to Q201.

Q200. ¿Cuánto tiempo lo has tenido? (escoja uno) (Elija Uno)	1	Menos de 8 días
	2	8-13 días
	3	14-20 días
	4	Más de 20 días
	7	No Sabe
	8	Se rehúsa a contestar
Q201. ¿Tienes**: escurrimiento nasal?**	1	Si
	0	No
	7	No Sabe
	8	Se rehúsa a contestar

If Q201 is equal to 1 then skip to Q202

If not, then skip to Q203.

Q202. ¿Cuánto tiempo lo has tenido? (escoja uno) (Elija Uno)	1	Menos de 8 días
	2	8-13 días
	3	14-20 días
	4	Más de 20 días
	7	No Sabe
	8	Se rehúsa a contestar

Q203. ¿Tienes**: tos?**	1	Si
	0	No
	7	No Sabe
	8	Se rehúsa a contestar

If Q203 is equal to 1 then skip to Q204

If not, then skip to Q205.

Q204. ¿Cuánto tiempo lo has tenido? (escoja uno) (Elija Uno)	1	Menos de 8 días
	2	8-13 días
	3	14-20 días
	4	Más de 20 días
	7	No Sabe
	8	Se rehúsa a contestar
Q205. ¿Tienes**: dolor de garganta?**	1	Si
	0	No
	7	No Sabe
	8	Se rehúsa a contestar

If Q205 is equal to 1 then skip to Q206

If not, then skip to Q207.

Q206. ¿Cuánto tiempo lo has tenido? (escoja uno) (Elija Uno)	1	Menos de 8 días
	2	8-13 días
	3	14-20 días
	4	Más de 20 días
	7	No Sabe
	8	Se rehúsa a contestar

Q207. ¿Tienes**: voz ronca?**	1	Si
	0	No
	7	No Sabe
	8	Se rehúsa a contestar

If Q207 is equal to 1 then skip to Q208

If not, then skip to Q209.

Q208. ¿Cuánto tiempo lo has tenido? (escoja uno) (Elija Uno)	1	Menos de 8 días
	2	8-13 días
	3	14-20 días
	4	Más de 20 días
	7	No Sabe
	8	Se rehúsa a contestar
Q209. ¿Tienes**: dificultad para respirar?**	1	Si
	0	No
	7	No Sabe
	8	Se rehúsa a contestar

If Q209 is equal to 1 then skip to Q210

If not, then skip to Q211.

Q210. ¿Cuánto tiempo lo has tenido? (escoja uno) (Elija Uno)	1	Menos de 8 días
	2	8-13 días
	3	14-20 días
	4	Más de 20 días
	7	No Sabe
	8	Se rehúsa a contestar

Q211. ¿Tienes**: los ganglios linfáticos agrandados**?	1	Si
	0	No
	7	No Sabe
	8	Se rehúsa a contestar

If Q211 is equal to 1 then skip to Q212

If not, then skip to Q213.

Q212. ¿Cuánto tiempo lo has tenido? (escoja uno) (Elija Uno)	1	Menos de 8 días
	2	8-13 días
	3	14-20 días
	4	Más de 20 días
	7	No Sabe
	8	Se rehúsa a contestar
Q213. ¿Tienes**: Náuseas o vómitos?**	1	Si
	0	No
	7	No Sabe
	8	Se rehúsa a contestar

If Q213 is equal to 1 then skip to Q214

If not, then skip to Q215.

Q214. ¿Cuánto tiempo lo has tenido? (escoja uno) (Elija Uno)	1	Menos de 8 días
	2	8-13 días
	3	14-20 días
	4	Más de 20 días
	7	No Sabe
	8	Se rehúsa a contestar

Q215. ¿Tienes**: dolor de cabeza?**

1	Si
0	No
7	No Sabe
8	Se rehúsa a contestar

If Q215 is equal to 1 then skip to Q216

If not, then skip to Q217.

Q216. ¿Cuánto tiempo lo has tenido? (escoja uno) (Elija Uno)

1	Menos de 8 días
2	8-13 días
3	14-20 días
4	Más de 20 días
7	No Sabe
8	Se rehúsa a contestar

Q217. ¿Tienes**: dolor abdominal?**

1	Si
0	No
7	No Sabe
8	Se rehúsa a contestar

If Q217 is equal to 1 then skip to Q218

If not, then skip to Q219.

Q218. ¿Cuánto tiempo lo has tenido? (escoja uno) (Elija Uno)

1	Menos de 8 días
2	8-13 días
3	14-20 días
4	Más de 20 días
7	No Sabe
8	Se rehúsa a contestar

Q219. ¿Tienes**: Diarrea (3 o más evacuaciones sueltas/más flojas de lo normal en un período de 24 horas)**?

	1	Si
	0	No
	7	No Sabe
	8	Se rehúsa a contestar

If Q219 is equal to 1 then skip to Q220

If not, then skip to Q221.

Q220. ¿Cuánto tiempo lo has tenido? (escoja uno) (Elija Uno)	1	Menos de 8 días
	2	8-13 días
	3	14-20 días
	4	Más de 20 días
	7	No Sabe
	8	Se rehúsa a contestar
Q221. ¿Tienes**: pérdida del olfato repentina?**	1	Si
	0	No
	7	No Sabe
	8	Se rehúsa a contestar

If Q221 is equal to 1 then skip to Q222

If not, then skip to Q223.

Q222. ¿Cuánto tiempo lo has tenido? (escoja uno) (Elija Uno)	1	Menos de 8 días
	2	8-13 días
	3	14-20 días
	4	Más de 20 días
	7	No Sabe
	8	Se rehúsa a contestar
Q223. ¿Tienes**: pérdida del gusto repentino?**	1	Si
	0	No
	7	No Sabe
	8	Se rehúsa a contestar

If Q223 is equal to 1 then skip to Q224

If not, then skip to Q225.

Q224. ¿Cuánto tiempo lo has tenido? (escoja uno) (Elija Uno)	1	Menos de 8 días
	2	8-13 días
	3	14-20 días
	4	Más de 20 días
	7	No Sabe
	8	Se rehúsa a contestar
Q225. ¿Tienes**: dolor de articulaciones?**	1	Si
	0	No
	7	No Sabe
	8	Se rehúsa a contestar

If Q225 is equal to 1 then skip to Q226

If not, then skip to Q227.

Q226. ¿Cuánto tiempo lo has tenido? (escoja uno) (Elija Uno)	1	Menos de 8 días
	2	8-13 días
	3	14-20 días
	4	Más de 20 días
	7	No Sabe
	8	Se rehúsa a contestar
Q227. ¿Tienes**: fatiga/cansancio extremo?**	1	Si
	0	No
	7	No Sabe
	8	Se rehúsa a contestar

If Q227 is equal to 1 then skip to Q228

If not, then skip to Q229.

Q228. ¿Cuánto tiempo lo has tenido? (escoja uno) (Elija Uno)	1	Menos de 8 días
	2	8-13 días
	3	14-20 días
	4	Más de 20 días
	7	No Sabe
	8	Se rehúsa a contestar
Q229. ¿Tienes**: hemóptisis** (**tos y flema con sangre**)	1	Si
	0	No
	7	No Sabe
	8	Se rehúsa a contestar

If Q229 is equal to 1 then skip to Q230

If not, then skip to Q231.

Q230. ¿Cuánto tiempo lo has tenido? (escoja uno) (Elija Uno)

1	Menos de 8 días
2	8-13 días
3	14-20 días
4	Más de 20 días
7	No Sabe
8	Se rehúsa a contestar

Q231. ¿Tienes**: dolor o presión en el pecho?**

1	Si
0	No
7	No Sabe
8	Se rehúsa a contestar

If Q231 is equal to 1 then skip to Q232

If not, then skip to Q233.

Q232. ¿Cuánto tiempo lo has tenido? (escoja uno) (Elija Uno)

1	Menos de 8 días
2	8-13 días
3	14-20 días
4	Más de 20 días
7	No Sabe
8	Se rehúsa a contestar

Q233. ¿Tienes**: pérdida de peso sin querer?**

1	Si
0	No
7	No Sabe
8	Se rehúsa a contestar

If Q233 is equal to 1 then skip to Q234

If not, then skip to instruction before Q235.

Q234. ¿Cuánto tiempo lo has tenido? (escoja uno) (Elija Uno)

1	Menos de 8 días
2	8-13 días
3	14-20 días
4	Más de 20 días
7	No Sabe
8	Se rehúsa a contestar

PREGUNTAS SOBRE EL ESTIGMA INTERSECCIONAL DE COVID

READ: LEA AL PARTICIPANTE: Las siguientes preguntas hablarán sobre si tu has sido tratado de una manera diferente durante la pandemia de COVID-19, cuando el gobierno le pidió a las personas que se resguardaran, se quedaran en casa, y que limitaran el número de personas con las que pasaban el tiempo en público.
Por COVID-19, nos referimos al coronavirus que también se le llama corona, el Rona, y Señorita Rona.
Las primeras preguntas serán sobre qué tan preocupado estabas que te hubieran tratado diferente, y las últimas preguntas serán sobre las diferentes formas en que a usted realmente lo trataron de manera diferente.

Q235. Cuando la pandemia de COVID-19 comenzó en Marzo 2020, ¿estabas preocupado de que pudieras ser acosado/a verbalmente o físicamente por otras personas (incluyendo la policía) porque había menos personas en público?

1	Si
0	No
8	Se rehúsa a contestar

If Q235 is equal to 1 then skip to instruction before Q236

If not, then skip to Q242.

READ: LEA AL PARTICIPANTE: En una escala del 1-5, con 1 siendo para nada y 5 siendo mucho, cuanto le preocupó que fuera amenazado o acosado por lo siguiente:

Q236. Cuánto te preocupó que fueras amenazado o acosado por: **Tu género** (Escoja uno) (Elija Uno)

1 Para nada

2 Un poco

3 Algo

4 Bastante

5 Mucho

8 Se rehúsa a contestar

Q237. Cuánto te preocupó que fueras amenazado o acosado por: **El color de tu piel** (Escoja uno) (Elija Uno)

1 Para nada

2 Un poco

3 Algo

4 Bastante

5 Mucho

8 Se rehúsa a contestar

Q238. Cuánto te preocupó que fueras amenazado o acosado por: **tatuajes visibles u otras marcas físicas** (Escoja uno) (Elija Uno)

1 Para nada

2 Un poco

3 Algo

4 Bastante

5 Mucho

8 Se rehúsa a contestar

Q239. Cuánto te preocupó que fueras amenazado o acosado por: **la ropa que usas** (Escoja uno) (Elija Uno)

1	Para nada
2	Un poco
3	Algo
4	Bastante
5	Mucho
8	Se rehúsa a contestar

Q240. Cuánto te preocupó que fueras amenazado o acosado por: **Tener trakes u otros signos de uso activo de drogas** (Escoja uno) (Elija Uno)

1	Para nada
2	Un poco
3	Algo
4	Bastante
5	Mucho
8	Se rehúsa a contestar

Q241. Cuánto te preocupó que fueras amenazado o acosado por: **Tu orientación sexual** (Escoja uno) (Elija Uno)

1	Para nada
2	Un poco
3	Algo
4	Bastante
5	Mucho
8	Se rehúsa a contestar

Q242. Cuando la pandemia de COVID-19 empezó en Marzo 2020 ¿te preocupaba que pudieras ser acosado verbalmente o físicamente por otros (incluyendo la policía) si usabas cubre bocas?

1 Si

0 No

8 Se rehúsa a contestar

If Q242 is equal to 1 then skip to instruction before Q243

If not, then skip to Q249.

READ: LEA AL PARTICIPANTE: En una escala del 1-5, con 1 siendo para nada y 5 siendo mucho, cuánto te preocupó que te amenazaran o acosaran por usar cubre bocas por lo siguiente:

Q243. Cuánto te preocupó que fueras amenazado o acosado por *usar cubre bocas* por: **su género** (escoja uno) (Elija Uno)

1 Para nada

2 Un poco

3 Algo

4 Bastante

5 Mucho

8 Se rehúsa a contestar

Q244. Cuánto te preocupó que fueras amenazado o acosado por *usar cubre bocas* por: **el color de tu piel** (escoja uno) (Elija Uno)

1 Para nada

2 Un poco

3 Algo

4 Bastante

5 Mucho

8 Se rehúsa a contestar

Q245. Cuánto te preocupó que fueras amenazado o acosado por *usar cubre bocas* por: **tatuajes visibles u otras marcas físicas** (escoja uno) (Elija Uno)

1 Para nada

2 Un poco

3 Algo

4 Bastante

5 Mucho

8 Se rehúsa a contestar

Q246. Cuánto te preocupó que fueras amenazado o acosado por *usar cubre bocas* por: **la ropa que usas** (escoja uno) (Elija Uno)

1 Para nada

2 Un poco

3 Algo

4 Bastante

5 Mucho

8 Se rehúsa a contestar

Q247. Cuánto te preocupó que fueras amenazado o acosado por *usar cubre bocas* por: **tener trakes u otros signos de uso activo de drogas** (escoja uno) (Elija Uno)

1 Para nada

2 Un poco

3 Algo

4 Bastante

5 Mucho

8 Se rehúsa a contestar

Q248. Cuánto te preocupó que fueras amenazado o acosado por *usar cubre bocas* por: **su orientación sexual** (escoja uno) (Elija Uno)

1 Para nada

2 Un poco

3 Algo

4 Bastante

5 Mucho

8 Se rehúsa a contestar

Q249. Durante la pandemia COVID-19 ¿sufriste más acoso verbal que antes (que te llamaran nombres o insultos)?

1 Si

0 No

8 Se rehúsa a contestar

If Q249 is equal to 1 then skip to instruction before Q250

If not, then skip to Q256.

READ: LEA AL PARTICIPANTE: En una escala de 1-5, con 1 siendo para nada y 5 siendo mucho, cuánto ……. este trato por lo siguiente:

Q250. Sufriste más abuso verbal que antes por: **Tu género** (escoja uno) (Elija Uno)

1 Para nada

2 Un poco

3 Algo

4 Bastante

5 Mucho

8 Se rehúsa a contestar

Q251. Sufriste más abuso verbal que antes por: **el color de tu piel** (escoja uno) (Elija Uno)

1 Para nada

2 Un poco

3 Algo

4 Bastante

5 Mucho

8 Se rehúsa a contestar

Q252. Sufriste más abuso verbal que antes por: **tatuajes visibles u otras marcas fisicas** (escoja uno) (Elija Uno)

1 Para nada

2 Un poco

3 Algo

4 Bastante

5 Mucho

8 Se rehúsa a contestar

Q253. Sufriste más abuso verbal que antes por: **La ropa que usas** (escoja uno) (Elija Uno)

1 Para nada

2 Un poco

3 Algo

4 Bastante

5 Mucho

8 Se rehúsa a contestar

Q254. Sufriste más abuso verbal que antes por: **tener trakes u otros signos de uso activo de drogas** (escoja uno) (Elija Uno)

1 Para nada

2 Un poco

3 Algo

4 Bastante

5 Mucho

8 Se rehúsa a contestar

Q255. Sufriste más abuso verbal que antes por: **tu orientación sexual** (escoja uno) (Elija Uno)

1	Para nada
2	Un poco
3	Algo
4	Bastante
5	Mucho
8	Se rehúsa a contestar

Q256. ¿Durante la pandemia de COVID-19 fuiste amenazado o dañado físicamente más que antes?

1	Si
0	No
8	Se rehúsa a contestar

If Q256 is equal to 1 then skip to instruction before Q257

If not, then skip to instruction before Q263.

READ: LEER AL PARTICIPANTE: En una escala del 1-5, con 1 siendo para nada y 5 siendo mucho, ¿cuánto fuiste por lo siguiente?

Q257. Amenazado o dañado físicamente más que antes por: **tu género** (escoja uno) (Elija Uno)

1	Para nada
2	Un poco
3	Algo
4	Bastante
5	Mucho
8	Se rehúsa a contestar

Q258. Amenazado o dañado físicamente más que antes por: **el color de tu piel** (escoja uno) (Elija Uno)

1 Para nada

2 Un poco

3 Algo

4 Bastante

5 Mucho

8 Se rehúsa a contestar

Q259. Amenazado o dañado físicamente más que antes por: **tatuajes visibles u otras marcas físicas** (escoja uno) (Elija Uno)

1 Para nada

2 Un poco

3 Algo

4 Bastante

5 Mucho

8 Se rehúsa a contestar

Q260. Amenazado o dañado físicamente más que antes por: **la ropa que usas** (escoja uno) (Elija Uno)

1 Para nada

2 Un poco

3 Algo

4 Bastante

5 Mucho

8 Se rehúsa a contestar

Q261. Amenazado o dañado físicamente más que antes por: **tener trakes u otros signos de uso activo de drogas** (escoja uno) (Elija Uno)

1	Para nada
2	Un poco
3	Algo
4	Bastante
5	Mucho
8	Se rehúsa a contestar

Q262. Amenazado o dañado físicamente más que antes por: **orientación sexual** (escoja uno) (Elija Uno)

1	Para nada
2	Un poco
3	Algo
4	Bastante
5	Mucho
8	Se rehúsa a contestar

READ: LEER AL PARTICIPANTE: Las siguientes preguntas se tratan sobre su estado de ánimo.

ITEMS DE LA ESCALA DE ANSIEDAD GAD-7 (COVBO1-COVBO7)

Q263. En las últimas 2 semanas, ¿con qué frecuencia le han molestado los siguientes problemas: sentirse nervioso, ansioso o al límite? (Escoja una) (Elija Uno)

1 Para nada

2 Varios días

3 Más de la mitad de los días

4 Casi todos los días

7 No Sabe

8 Se rehúsa a contestar

Q264. En las últimas 2 semanas, ¿con qué frecuencia le ha molestado el: no poder detener o controlar las preocupaciones? (Escoja una) (Elija Uno)

1 Para nada

2 Varios días

3 Más de la mitad de los días

4 Casi todos los días

7 No Sabe

8 Se rehúsa a contestar

Q265. En las últimas 2 semanas, ¿con qué frecuencia le ha molestado el: preocuparse demasiado por diferentes cosas? (Escoja una) (Elija Uno)

1 Para nada

2 Varios días

3 Más de la mitad de los días

4 Casi todos los días

7 No Sabe

8 Se rehúsa a contestar

Q266. En las últimas 2 semanas, ¿con qué frecuencia le ha molestado el: tener problemas para relajarse? (Escoja una) (Elija Uno)

1 Para nada

2 Varios días

3 Más de la mitad de los días

4 Casi todos los días

7 No Sabe

8 Se rehúsa a contestar

Q267. En las últimas 2 semanas, ¿con qué frecuencia le ha molestado: estar tan intranquilo que es difícil quedarse quieto? (Escoja una) (Elija Uno)

1 Para nada

2 Varios días

3 Más de la mitad de los días

4 Casi todos los días

7 No Sabe

8 Se rehúsa a contestar

Q268. En las últimas 2 semanas, ¿con qué frecuencia le ha molestado: Ser fácilmente irritado o encontrarse irritable? (Escoja una) (Elija Uno)

1 Para nada

2 Varios días

3 Más de la mitad de los días

4 Casi todos los días

7 No Sabe

8 Se rehúsa a contestar

Q269. En las últimas 2 semanas, ¿con qué frecuencia le ha molestado: sentir miedo como si algo horrible fuera a suceder? (Escoja una) (Elija Uno)

1 Para nada

2 Varios días

3 Más de la mitad de los días

4 Casi todos los días

7 No Sabe

8 Se rehúsa a contestar

READ: LEA: LEA: Por favor indique hasta qué punto está de acuerdo con cada una de las siguientes declaraciones.

ITEMS DE LA ESCALA BREVE SOBRE RESILIENCIA (COVCOP1-COVCOP6)

Q270. Tiendo a recuperarme rápidamente después de tiempos difíciles. (Escoja una) (Elija Uno)

1 Muy en desacuerdo

2 En desacuerdo

3 Neutral

4 De acuerdo

5 Muy de acuerdo

8 Se rehúsa a contestar

Q271. Tengo dificultad pasando por eventos estresantes. (Escoja una) (Elija Uno)

1 Muy en desacuerdo

2 En desacuerdo

3 Neutral

4 De acuerdo

5 Muy de acuerdo

8 Se rehúsa a contestar

Q272. No me toma mucho tiempo el recuperarme de un evento estresante. (Escoja una) (Elija Uno)

1 Muy en desacuerdo

2 En desacuerdo

3 Neutral

4 De acuerdo

5 Muy de acuerdo

8 Se rehúsa a contestar

Q273. Regresarme cuesta recuperarme de inmediato de un evento malo. (Escoja una) (Elija Uno)

1 Muy en desacuerdo

2 En desacuerdo

3 Neutral

4 De acuerdo

5 Muy de acuerdo

8 Se rehúsa a contestar

Q274. Normalmente me recupero de tiempos difíciles sin mucho problemas. (Escoja una) (Elija Uno)

1 Muy en desacuerdo

2 En desacuerdo

3 Neutral

4 De acuerdo

5 Muy de acuerdo

8 Se rehúsa a contestar

Q275. Tiendo a tomar mucho tiempo para superar los contratiempos en mi vida. (Escoja una) (Elija Uno)

1 Muy en desacuerdo

2 En desacuerdo

3 Neutral

4 De acuerdo

5 Muy de acuerdo

8 Se rehúsa a contestar

Q276. ¿Cuándo fue su visita más reciente a un médico, enfermera u otro proveedor de salud? Incluyendo telemedicina y visitas remotas. (Escoja una) (Elija Uno)

1 En las últimas dos semanas

2 Entre 2 semanas y 1 mes

3 Más de 1 mes pero dentro de los 6 meses

4 Hace 6-12 meses

5 Hace más de 12 meses.

8 Se rehúsa a contestar

Q277. En los últimos 7 días, ¿se le ha acabado algún medicamento recetado? (Escoja una) (Elija Uno)

1 Sí, n

2 Sí, n

3 Sí, t

4 No

8 Se r

Tuberculosis, Percepciones, Pruebas, y Tratamiento

READ: LEA: Las siguientes preguntas son acerca de su entendimiento y sentimientos acerca de la Tuberculosis (TB). Por favor responda "Si" o "No" a cada pregunta. Si usted no sabe la respuesta, está bien; solo dígame que no lo sabe.

Q278. ¿Puede la gente tener TB latente (dormida) en su cuerpo y no tener la enfermedad activa?

1 Si

0 No

7 No Sabe

8 Se rehúsa a contestar

Q279. ¿El compartir los trastes, botellas, o cepillos de dientes con alguien que tenga TB aumenta la posibilidad de contagiarse con TB?

1 Si

0 No

7 No Sabe

8 Se rehúsa a contestar

Q280. ¿Se puede contagiar la TB de persona a persona por medio del aire?

1 Si

0 No

7 No Sabe

8 Se rehúsa a contestar

Q281. ¿La TB se puede curar tomando medicinas?

1 Si

0 No

7 No Sabe

8 Se rehúsa a contestar

Q282. ¿Tiene la cicatriz de la vacuna BCG? (la cicatriz BCG se hace en el bíceps, a diferencia de la prueba cutánea de TB que se hace en el antebrazo).

1 Si

0 No

7 No Sabe

8 Se rehúsa a contestar

Q283. ¿Alguna vez se hizo una prueba de la piel para la TB?

1 Si

0 No

7 No Sabe

8 Se rehúsa a contestar

If Q283 is equal to 1 then skip to Q284

If not, then skip to Q285.

Q284. ¿Cuál fue el resultado de la prueba? (Elija Uno)

0 Negativo

1 Positivo

2 No determinado

7 No Sabe

8 Se rehúsa a contestar

Q285. ¿Alguna vez le han hecho una radiografía del pecho porque el personal médico pensó que usted podría tener TB?

1 Si

0 No

7 No Sabe

8 Se rehúsa a contestar

If Q285 is equal to 1 then skip to Q286

If not, then skip to Q287.

Q286. ¿Cuando fue la última vez que le hicieron una radiografía del pecho para la TB? (Elija Uno) (Elija Uno)

1 Durante el último mes

2 Hace 2-6 meses

3 Hace 7-12 meses

4 Hace Más de un Año

5 No Sabe

6 Se rehúsa a contestar

Q287. ¿Alguna vez le ha dicho un doctor o un trabajador de salud que usted tiene la TB <u>activa</u>? Esto no incluye tener una prueba de piel positiva pero sin síntomas.

1 Si

0 No

7 No Sabe

8 Se rehúsa a contestar

If Q287 is equal to 1 then skip to Q288

If not, then skip to Q293.

Q288. ¿En qué CIUDAD le dijeron que tenía TB <u>activa?</u>

____

Q289. ¿En qué ESTADO le dijeron que tenía TB <u>activa?</u>

____

Q290. ¿En que PAIS le dijeron que tenía TB activa? (Elija Uno)

0 México

1 EUA

2 Otro

7 No Sabe

8 Se rehúsa a contestar

If Q290 is not equal to 2, then skip to Q292.

Q291. Especifique qué otro país:

—

Q292. ¿Cuándo le dijeron que su prueba de TB activa salió positiva? (Indique el Mes y Año)

— — / — — — — mm / aaaa

2097 No Sabe (Año)

2098 Se rehúsa a contestar (Año)

Q293. ¿Alguna vez ha tomado medicamentos para la enfermedad de la TB o TB latente?

1 Si

0 No

7 No Sabe

8 Se rehúsa a contestar

If Q293 is equal to 1 then skip to Q294

If not, then skip to Q298.

Q294. ¿Por cuántos meses tomó medicamentos para la TB? — —

97 No Sabe

98 Se rehúsa a contestar

Q295. ¿Dejaste de tomar tu medicamento antes de lo que te recetó el doctor?

1 Si

0 No

7 No Sabe

8 Se rehúsa a contestar

If Q295 is equal to 1 then skip to Q296

If not, then skip to instruction before Q297.

Q296. Si usted dejó de tomar sus medicamentos antes de lo que le indicó su doctor, ¿cuál fue la razón por la que dejó de tomarlos? **(Entrevistador: Lea la lista, marque todas las respuestas que apliquen)** (Marque Todas las que Correspondan)

__ Los medicamentos me hacían daño

__ No creía que necesitara tomarlos

__ No podía pagar más medicamentos

__ Mi doctor me dijo que los dejara de tomar

__ Me deportaron a México

__ Otro

__ Se rehúsa a contestar

If Q296F is equal to 1 then skip to Q297

If not, then skip to Q298.

Q297. Especifique cuál otra razón por la que interrumpió el medicamento antes de tiempo:

__ __

Q298. ¿Ha conocido a alguien que tuviera TB?

1	Si
0	No
7	No Sabe
8	Se rehúsa a contestar

If Q298 is equal to 1 then skip to Q299

If not, then skip to instruction before Q301.

Q299. ¿Cuántas personas ha conocido que tenían TB? __ __ __ __

9997	No Sabe
9998	Se rehúsa a contestar

Q300. ¿Cuál era su relación con esta persona o personas? **(Entrevistador: Lea la lista, marque todas las respuestas que apliquen)** (Marque Todas las que Correspondan)

__ Miembro Adulto de la familia que vive con usted, incluyendo conyugue

__ Miembro menor de la familia que vive con usted

__ Persona que vive con usted que no es familiar

__ Miembro de la familia que NO vive con usted

__ Pareja sexual que no vive con usted

__ Amistad cercana

__ Alguien que trabaja con usted

__ Conocido (alguien que conociste una o dos veces)

__ Otro usuario de drogas

__ Otro

__ No Sabe

__ Se rehúsa a contestar

If Q300J is equal to 1 then skip to Q301

If not, then skip to Q302.

Q301. Especifique la relación a esa persona:

____

Q302. En los últimos **6 meses**, ha tomado leche bronca o queso sin pasteurizar? Estos son usualmente productos que no han sido procesados en una fábrica como **queso fresco.**

1 Si

0 No

7 No Sabe

8 Se rehúsa a contestar

Q303. ¿Alguna vez ha vivido o trabajado en una lechería, rancho, o un rastro, u otro lugar donde se crían o faenan/carnean/sacrifican vacas?

1 Si

0 No

7 No Sabe

8 Se rehúsa a contestar

If Q191 is equal to 1 or Q193 is equal to 1 or Q195 is equal to 1 or Q197 is equal to 1 or Q199 is equal to 1 or Q201 is equal to 1 or Q203 is equal to 1 or Q205 is equal to 1 or Q207 is equal to 1 or Q209 is equal to 1 or Q213 is equal to 1 or Q215 is equal to 1 or Q217 is equal to 1 or Q219 is equal to 1 or Q221 is equal to 1 or Q223 is equal to 1 or Q227 is equal to 1 then READ: "Interviewer, at the end of the interview you will be prompted to give the participant a Covid-19 referral.".

If Q204 is equal to 4 and (Q195 is equal to 1 or Q197 is equal to 1 or Q207 is equal to 1 or Q209 is equal to 1 or Q211 is equal to 1 or Q225 is equal to 1 or Q227 is equal to 1 or Q231 is equal to 1) or Q229 is equal to 1 then READ: "Al final de la entrevista, brinde al participante una referencia para la Clínica de tratamiento de TB.".

Sección de Autoexámen de VIH

READ: LEA AL PARTICIPANTE: Las siguientes preguntas son sobre autoexámenes de VIH. Un autoexámen de VIH se refiere a una prueba rápida de VIH que puedes administrarte a ti mismo en casa o en cualquier ubicación conveniente al recolectar tu propia saliva o sangre al pinchar su dedo con una pequeña aguja. Si sales positivo al VIH en un autoexamen de VIH, aun tendras que hacerte una prueba standard de VIH en una clínica u organización comunitaria que realice la prueba de VIH para confirmar tus resultados. Los autoexámenes de VIH están actualmente disponibles en otros países, incluyendo Estados Unidos. Para planear para el uso futuro de autoexámenes de VIH en México, nos gustaría saber qué piensas tu sobre los autoexámenes de VIH.

Q304. ¿Alguna vez te has hecho un autoexámen de VIH? (escoja uno) (Elija Uno)

0 No

1 Si

2 No aplica (es VIH Positivo)

8 Se rehúsa a contestar

If Q304 is equal to 2, then skip to instruction before Q314.

Q305. ¿Estarías dispuesto a usar un autoexámen de VIH? (escoja uno) (Elija Uno)

1 Definitivamente no

2 Probablemente no

3 No estoy seguro, no se

4 Probablemente

5 Definitivamente

8 Se rehúsa a contestar

Q306. ¿Estarías dispuesto a darles un autoexámen de VIH a tus parejas sexuales para que ellos se hagan la prueba de VIH? (escoja uno) (Elija Uno)

1 Definitivamente no

2 Probablemente no

3 No estoy seguro, no se

4 Probablemente

5 Definitivamente

8 Se rehúsa a contestar

Q307. ¿Estarías dispuesto a darle autoexámenes de VIH a tus compañeros usuarios de drogas para que ellos se hagan la prueba de VIH? (escoja uno) (Elija Uno)

1 Definitivamente no

2 Probablemente no

3 No estoy seguro, no se

4 Probablemente

5 Definitivamente

8 Se rehúsa a contestar

If Q305 is equal to 1 or Q305 is equal to 2 or Q305 is equal to 3 then skip to instruction before Q308

If not, then skip to instruction before Q311.

READ: LEA: LEA AL PARTICIPANTE: Ahora te voy a leer una lista de razones por las que algunas personas no quieren usar autoexámenes de VIH. Por favor indica qué tan de acuerdo o en desacuerdo estás con cada declaración.

Q308. No me gustaría usar un autoexámen de VIH porque **me preocuparía que los autoexámenes de VIH son menos acertados que las pruebas standard de VIH** (escoja uno) (Elija Uno)

1 Totalmente en desacuerdo

2 En desacuerdo

3 Ni en acuerdo o desacuerdo

4 De acuerdo

5 Totalmente de acuerdo

8 Se rehúsa a contestar

Q309. No me gustaría usar un autoexámen de VIH porque **me preocuparía usar mal el** autoexámen **de VIH** (escoja uno) (Elija Uno)

1 Totalmente en desacuerdo

2 En desacuerdo

3 Ni en acuerdo o desacuerdo

4 De acuerdo

5 Totalmente de acuerdo

8 Se rehúsa a contestar

Q310. No me gustaría usar un autoexámen de VIH porque **me preocuparía malinterpretar los resultados** (escoja uno) (Elija Uno)

1 Totalmente en desacuerdo

2 En desacuerdo

3 Ni en acuerdo o desacuerdo

4 De acuerdo

5 Totalmente de acuerdo

8 Se rehúsa a contestar

If Q305 is equal to 3 or Q305 is equal to 4 or Q305 is equal to 5 then skip to instruction before Q311

If not, then skip to instruction before Q314.

READ: LEER: LEER AL PARTICIPANTE: Ahora te voy a leer una lista de razones por las que algunas personas se motivan a querer usar autoexámenes de VIH. Por favor indica que tan de acuerdo o en desacuerdo estas con cada declaración.

Q311. Me motivaría a usar autoexámenes de VIH **porque podría hacerme pruebas de VIH más seguido.** (Escoja uno) (Elija Uno)

1 Totalmente en desacuerdo

2 En desacuerdo

3 Ni en acuerdo o desacuerdo

4 De acuerdo

5 Totalmente de acuerdo

8 Se rehúsa a contestar

Q312. Me motivaría a usar autoexámenes de VIH **porque sería más conveniente que ir a una clínica u organización comunitaria para una prueba de VIH standard.** (Escoja uno) (Elija Uno)

1 Totalmente en desacuerdo

2 En desacuerdo

3 Ni en acuerdo o desacuerdo

4 De acuerdo

5 Totalmente de acuerdo

8 Se rehúsa a contestar

Q313. Me motivaría a usar autoexámenes de VIH **porque me daría más privacidad y me ayudaría a asegurar la confidencialidad de mis resultados de prueba de VIH.** (Escoja uno) (Elija Uno)

1	Totalmente en desacuerdo
2	En desacuerdo
3	Ni en acuerdo o desacuerdo
4	De acuerdo
5	Totalmente de acuerdo
8	Se rehúsa a contestar

Sección de Estigma de Metadona, Buprenorfina y Suboxona

READ: LEA: LEA AL PARTICIPANTE: Las siguientes preguntas serán sobre acceso a tratamiento de rehabilitación de drogas. Hablamos sobre esto en su visita inicial un poco. ¿Puede recordarme de sus experiencias con programas de rehabilitación de drogas?

Q314. ¿Alguna vez en su vida se ha inscrito en un programa de metadona? **Entrevistador: Si el participante dice: "No sé", LEA: La Metadona es una terapia oral de sustitución, lo que significa que el medicamento se le administra por vía oral (tomada) para ayudar a lidiar con su adicción.)**

1 Si

0 No

8 Se rehúsa a contestar

If Q314 is equal to 1 then skip to Q315

If not, then skip to Q316.

Q315. ¿Actualmente se encuentra inscrito en un programa de metadona?

1 Si

0 No

8 Se rehúsa a contestar

Q316. ¿Alguna vez en su vida ha estado inscrito en un programa de Buprenorfina-Suboxona? **(Entrevistador: Si el participante dice: "No sé", LEA: La buprenorfina/suboxona es una terapia oral de sustitución similar a la metadona, lo que significa que el medicamento se le administra por vía oral (tomada) para ayudar a lidiar con su adicción.)**

1 Si

0 No

8 Se rehúsa a contestar

If Q316 is equal to 1 then skip to Q317

If not, then skip to instruction before Q318.

Q317. ¿Actualmente se encuentra inscrito en un programa de Buprenorfina/Suboxona?

1 Si

0 No

8 Se rehúsa a contestar

Escala del Mecanismo de Estigma del Tratamiento de Mantenimiento con Metadona (MMT-SMS)

If Q315 is equal to 1 and Q15 is equal to 1 then skip to instruction before Q318

If not, then skip to instruction before Q343.

READ: LEA: LEA AL PARTICIPANTE: Las siguientes preguntas son sobre recibir medicamento para la dependencia a opioides como la metadona, tratamiento de mantenimiento. Por favor piensa sobre cada pregunta e indica tu respuesta. El primer grupo de preguntas son sobre cómo te tratarían las personas en un futuro por recibir metadona. El segundo grupo de preguntas es sobre cómo te han tratado las personas en el pasado por haber recibido metadona. El tercer grupo es sobre cómo te sientes tu por estar recibiendo metadona.

READ: LEA LO SIGUIENTE AL PARTICIPANTE Y LUEGO PASE A LA SIGUIENTE PANTALLA Y LEA LAS AFIRMACIONES RELACIONADAS: ¿Qué tan seguido la gente te ha tratado así en el pasado porque recibías metadona?

ITEMS DE LA ESCALA DE ESTIGMA ESTABLECIDO (ES1-ES9)

Q318. Miembros de mi familia han pensado que sigo siendo un usuario de drogas. (Elija Uno)

1	Nunca
2	No muy seguido
3	Algo seguido
4	Seguido
5	Muy seguido
8	Se rehúsa a contestar

Q319. Miembros de mi familia no han apoyado mi tratamiento de metadona. (Escoja una) (Elija Uno)

1 Nunca

2 No muy seguido

3 Algo seguido

4 Seguido

5 Muy seguido

8 Se rehúsa a contestar

Q320. Miembros de mi familia han pensado que no puedo mejorar. (Escoja una) (Elija Uno)

1 Nunca

2 No muy seguido

3 Algo seguido

4 Seguido

5 Muy seguido

8 Se rehúsa a contestar

Q321. Los empleadores han pesado que sigo siendo un usuario de drogas. (Escoja una) (Elija Uno)

1 Nunca

2 No muy seguido

3 Algo seguido

4 Seguido

5 Muy seguido

8 Se rehúsa a contestar

Q322. Los empleadores han pensado que soy un mal trabajador. (Escoja una) (Elija Uno)

1 Nunca

2 No muy seguido

3 Algo seguido

4 Seguido

5 Muy seguido

8 Se rehúsa a contestar

Q323. Los empleadores han pensado que el manejar mi horario de dósis de metadona sería un problema. (Escoja una) (Elija Uno)

1 Nunca

2 No muy seguido

3 Algo seguido

4 Seguido

5 Muy seguido

8 Se rehúsa a contestar

Q324. Los trabajadores de salud han pensado que sigo siendo un usuario de drogas. (Escoja una) (Elija Uno)

1 Nunca

2 No muy seguido

3 Algo seguido

4 Seguido

5 Muy seguido

8 Se rehúsa a contestar

Q325. Los trabajadores de salud me han dado un mal trato. (Escoja una) (Elija Uno)

1	Nunca
2	No muy seguido
3	Algo seguido
4	Seguido
5	Muy seguido
8	Se rehúsa a contestar

Q326. Los trabajadores de salud no me han recetado medicamento que yo necesitaba. (Escoja una) (Elija Uno)

1	Nunca
2	No muy seguido
3	Algo seguido
4	Seguido
5	Muy seguido
8	Se rehúsa a contestar

READ: LEER LO SIGUIENTE AL PARTICIPANTE Y LUEGO PASAR A LA SIGUIENTE PANTALLA Y LEER LAS AFIRMACIONES RELACIONADAS: ¿Qué tan probable es que las personas te trataran de alguna de las siguientes maneras en el futuro porque recibes metadona?

ITEMS DE LA ESCALA DE ESTIGMA ANTICIPADO (AS1-AS9)

Q327. Miembros de mi familia pensarán que sigo siendo un usuario de drogas. (Escoja una) (Elija Uno)

1 Nunca

2 No muy seguido

3 Algo seguido

4 Seguido

5 Muy seguido

8 Se rehúsa a contestar

Q328. Miembros de mi familia no me apoyarán con mi tratamiento de metadona. (Escoja una) (Elija Uno)

1 Nunca

2 No muy seguido

3 Algo seguido

4 Seguido

5 Muy seguido

8 Se rehúsa a contestar

Q329. Miembros de mi familia pensarán que no puedo mejorar. (Escoja una) (Elija Uno)

1 Nunca

2 No muy seguido

3 Algo seguido

4 Seguido

5 Muy seguido

8 Se rehúsa a contestar

Q330. Los empleadores pensarán que sigo siendo un usuario de drogas. (Escoja una) (Elija Uno)

1 Nunca

2 No muy seguido

3 Algo seguido

4 Seguido

5 Muy seguido

8 Se rehúsa a contestar

Q331. Los empleadores pensarán que soy un mal trabajador. (Escoja una) (Elija Uno)

1 Nunca

2 No muy seguido

3 Algo seguido

4 Seguido

5 Muy seguido

8 Se rehúsa a contestar

Q332. Los empleadores pensarán que el manejar mi horario de dosis de metadona será un problema. (Escoja una) (Elija Uno)

1 Nunca

2 No muy seguido

3 Algo seguido

4 Seguido

5 Muy seguido

8 Se rehúsa a contestar

Q333. Los trabajadores de salud pensarán que sigo siendo un usuario de drogas. (Escoja una) (Elija Uno)

1 Nunca

2 No muy seguido

3 Algo seguido

4 Seguido

5 Muy seguido

8 Se rehúsa a contestar

Q334. Los trabajadores de salud me darán un mal trato. (Escoja una) (Elija Uno)

1 Nunca

2 No muy seguido

3 Algo seguido

4 Seguido

5 Muy seguido

8 Se rehúsa a contestar

Q335. Los trabajadores de salud no me van a recetar medicamento que yo necesito. (Escoja una) (Elija Uno)

1 Nunca

2 No muy seguido

3 Algo seguido

4 Seguido

5 Muy seguido

8 Se rehúsa a contestar

ITEMS DE ESTIGMA INTERNALIZADO (IS1-IS7)

Q336. El recibir metadona me hace sentir que soy una mala persona. (Escoja una) (Elija Uno)

1 Nunca

2 No muy seguido

3 Algo seguido

4 Seguido

5 Muy seguido

8 Se rehúsa a contestar

Q337. Siento que no soy tan bueno/a como otras personas porque recibo metadona. (Escoja una) (Elija Uno)

1 Nunca

2 No muy seguido

3 Algo seguido

4 Seguido

5 Muy seguido

8 Se rehúsa a contestar

Q338. Me siento avergonzado/a de mi tratamiento de metadona. (Escoja una) (Elija Uno)

1 Nunca

2 No muy seguido

3 Algo seguido

4 Seguido

5 Muy seguido

8 Se rehúsa a contestar

Q339. No me respeto a mí mismo/a porque recibo metadona. (Escoja una) (Elija Uno)

1 Nunca

2 No muy seguido

3 Algo seguido

4 Seguido

5 Muy seguido

8 Se rehúsa a contestar

Q340. El recibir metadona me hace sentir sucio/a. (Escoja una) (Elija Uno)

1 Nunca

2 No muy seguido

3 Algo seguido

4 Seguido

5 Muy seguido

8 Se rehúsa a contestar

Q341. Estar en tratamiento de metadona es algo desagradable/asqueroso para mí. (Escoja una) (Elija Uno)

1 Nunca

2 No muy seguido

3 Algo seguido

4 Seguido

5 Muy seguido

8 Se rehúsa a contestar

Q342. Estar en tratamiento de metadona me hace sentir que sigo siendo un usuario de drogas. (Escoja una) (Elija Uno)

1 Nunca

2 No muy seguido

3 Algo seguido

4 Seguido

5 Muy seguido

8 Se rehúsa a contestar

ESCALA DE ESTIGMA HACIA TRATAMIENTO DE MANTENIMIENTO CON METADONA (18 items)-MMTS1-MMTS18. Nota: MMTS17 estácodificado al revés.

If Q314 is not equal to 1 then skip to instruction before Q343

If not, then skip to instruction before Q361.

READ: Entrevistador, lea al participante: La metadona es un medicamento que se puede tomar para tratar la dependencia a opioides. La metadona es un medicamento que trata la adicción a la heroína y otros opioides; reduce los síntomas de abstinencia y antojo de drogas, pero no te hará sentirte drogado. Estas preguntas serán sobre cómo te podrían tratar en un <u>futuro</u> si empezaras a tomar metadona. Por favor selecciona una respuesta para cada pregunta. No tardes mucho tiempo considerando tu respuesta. Tu primera impresión es usualmente la mejor.

Q343. ¿Qué tan preocupado/a estás que se te negara alojamiento si tomas metadona? **(escoja uno)** (Elija Uno)

1 Para nada

2 Un poco

3 Algo

4 Bastante

5 Extremadamente

8 Se rehúsa a contestar

Q344. ¿Qué tan preocupado/a estás que se te negara trabajo si tomas metadona? **(escoja uno)** (Elija Uno)

1 Para nada

2 Un poco

3 Algo

4 Bastante

5 Extremadamente

8 Se rehúsa a contestar

Q345. ¿Qué tan preocupado/a estás que se te niegue asistencia de gobierno o beneficios similares si tomas metadona? **(escoja uno)** (Elija Uno)

1 Para nada

2 Un poco

3 Algo

4 Bastante

5 Extremadamente

8 Se rehúsa a contestar

READ: LEA: LEA LO SIGUIENTE AL PARTICIPANTE Y LUEGO PASE A LA SIGUIENTE PANTALLA Y LEA LAS AFIRMACIONES RELACIONADAS: ¿Qué tan probable crees que es que las personas te vayan a tratar así en el futuro si estuvieras tomando metadona?

Q346. Mis familiares pensarán que sigo siendo un usuario de drogas. **(escoja uno)** (Elija Uno)

1 Nunca

2 No seguido

3 Algunas veces

4 Seguido

5 Muy seguido

8 Se rehúsa a contestar

Q347. Mis familiares no apoyarán mi tratamiento de metadona. **(escoja uno)** (Elija Uno)

1 Nunca

2 No seguido

3 Algunas veces

4 Seguido

5 Muy seguido

8 Se rehúsa a contestar

Q348. Mis familiares pensarán que no puedo mejorar. **(escoja uno)** (Elija Uno)

1 Nunca

2 No seguido

3 Algunas veces

4 Seguido

5 Muy seguido

8 Se rehúsa a contestar

Q349. Los trabajadores de salud pensarán que sigo siendo un usuario de drogas. **(escoja uno)** (Elija Uno)

1 Nunca

2 No seguido

3 Algunas veces

4 Seguido

5 Muy seguido

8 Se rehúsa a contestar

Q350. Los trabajadores de salud me darán un mal trato. **(escoja uno)** (Elija Uno)

1 Nunca

2 No seguido

3 Algunas veces

4 Seguido

5 Muy seguido

8 Se rehúsa a contestar

Q351. Los trabajadores de salud no me recetarán el medicamento que necesito. **(escoja uno)** (Elija Uno)

1 Nunca

2 No seguido

3 Algunas veces

4 Seguido

5 Muy seguido

8 Se rehúsa a contestar

Q352. Otras personas que se inyectan drogas pensarán que soy débil si tomo metadona. **(escoja uno)** (Elija Uno)

1 Nunca

2 No seguido

3 Algunas veces

4 Seguido

5 Muy seguido

8 Se rehúsa a contestar

Q353. Otras personas que se inyectan drogas no me apoyarán si tomo metadona.**(escoja uno)** (Elija Uno)

1 Nunca

2 No seguido

3 Algunas veces

4 Seguido

5 Muy seguido

8 Se rehúsa a contestar

Q354. Otras personas que se inyectan drogas no me tendrán confianza si tomo metadona. **(escoja uno)** (Elija Uno)

1 Nunca

2 No seguido

3 Algunas veces

4 Seguido

5 Muy seguido

8 Se rehúsa a contestar

READ: LEA: LEA: LEA LO SIGUIENTE AL PARTICIPANTE Y LUEGO PASE A LA SIGUIENTE PANTALLA Y LEA LAS AFIRMACIONES RELACIONADAS: ¿Cómo te sientes sobre las personas que toman metadona?

Q355. Las personas que toman metadona están reEmplazando una droga por otra. **(Escoja una)** (Elija Uno)

1 Totalmente en desacuerdo

2 En desacuerdo

3 Ni de acuerdo ni en desacuerdo

4 De acuerdo

5 Totalmente de acuerdo

8 Se rehúsa a contestar

Q356. Las personas que toman metadona no son de fiar/confiables. **(Escoja una)** (Elija Uno)

1 Totalmente en desacuerdo

2 En desacuerdo

3 Ni de acuerdo ni en desacuerdo

4 De acuerdo

5 Totalmente de acuerdo

8 Se rehúsa a contestar

Q357. Las personas que toman metadona no están en control de su propia vida. **(Escoja una)** (Elija Uno)

1 Totalmente en desacuerdo

2 En desacuerdo

3 Ni de acuerdo ni en desacuerdo

4 De acuerdo

5 Totalmente de acuerdo

8 Se rehúsa a contestar

Q358. Las personas que toman metadona me hacen sentir incómodo/a. **(Escoja una)** (Elija Uno)

1 Totalmente en desacuerdo

2 En desacuerdo

3 Ni de acuerdo ni en desacuerdo

4 De acuerdo

5 Totalmente de acuerdo

8 Se rehúsa a contestar

Q359. Las personas que toman metadona me hacen sentir con esperanza. **(Escoja una)** (Elija Uno)

1 Totalmente en desacuerdo

2 En desacuerdo

3 Ni de acuerdo ni en desacuerdo

4 De acuerdo

5 Totalmente de acuerdo

8 Se rehúsa a contestar

Q360. Las personas que toman metadona me hacen sentir enojado/a. **(Escoja una)** (Elija Uno)

1 Totalmente en desacuerdo

2 En desacuerdo

3 Ni de acuerdo ni en desacuerdo

4 De acuerdo

5 Totalmente de acuerdo

8 Se rehúsa a contestar

Escala de Mecanismo de Estigma por Tratamiento de Mantenimiento con Buprenorfina/Suboxona (BSMT-SMS)

If Q317 is equal to 1 and Q15 is equal to 1 then skip to instruction before Q361

If not, then skip to instruction before Q386.

READ: LEA: Entrevistador LEA AL PARTICIPANTE: Las siguientes preguntas son sobre el recibir medicamentos para la dependencia a opioides como Buprenorfina o suboxona, tratamiento de mantenimiento. Por favor piensa sobre cada pregunta e indica tu respuesta. El primer grupo de preguntas son sobre cómo te tratarían las personas en el futuro por tu tratamiento de buprenorfina o suboxona. El segundo grupo de preguntas es sobre cómo te han tratado en el pasado por tu tratamiento de buprenorfina o suboxona. El tercer grupo de preguntas es sobre cómo te sientes tu sobre tu tratamiento de buprenorfina o suboxona.

READ: LEA LO SIGUIENTE AL PARTICIPANTE Y LUEGO PASE A LA SIGUIENTE PANTALLA Y LEA LAS AFIRMACIONES RELACIONADAS: ¿Qué tan seguido te han tratado las personas así en el pasado porque recibías buprenorfina/suboxona?

ITEMS DE ESTIGMA ESTABLECIDO (BSES1-BSES9)

Q361. Miembros de mi familia han pensado que aún sigo siendo un usuario de drogas. **(Escoja una)** (Elija Uno)

1 Nunca

2 No seguido

3 Algunas veces

4 Seguido

5 Muy seguido

8 Se rehúsa a contestar

Q362. Miembros de mi familia no han apoyado mi tratamiento de buprenorfina/suboxona. **(Escoja una)** (Elija Uno)

1	Nunca
2	No seguido
3	Algunas veces
4	Seguido
5	Muy seguido
8	Se rehúsa a contestar

Q363. Miembros de mi familia han pensado que no puedo mejorar. **(Escoja una)** (Elija Uno)

1	Nunca
2	No seguido
3	Algunas veces
4	Seguido
5	Muy seguido
8	Se rehúsa a contestar

Q364. Los empleadores han pensado que sigo siendo un usuario de drogas. **(Escoja una)** (Elija Uno)

1	Nunca
2	No seguido
3	Algunas veces
4	Seguido
5	Muy seguido
8	Se rehúsa a contestar

Q365. Los empleadores han pensado que soy un mal trabajador. **(Escoja una)** (Elija Uno)

1 Nunca

2 No seguido

3 Algunas veces

4 Seguido

5 Muy seguido

8 Se rehúsa a contestar

Q366. Los empleadores han pensado que el manejar mi horario de dosis de buprenorfina/suboxona era un problema. **(Escoja una)** (Elija Uno)

1 Nunca

2 No seguido

3 Algunas veces

4 Seguido

5 Muy seguido

8 Se rehúsa a contestar

Q367. Los trabajadores de salud han pensado que sigo siendo un usuario de drogas. **(Escoja una)** (Elija Uno)

1 Nunca

2 No seguido

3 Algunas veces

4 Seguido

5 Muy seguido

8 Se rehúsa a contestar

Q368. Los trabajadores de salud me han tratado mal. **(Escoja una)** (Elija Uno)

1 Nunca

2 No seguido

3 Algunas veces

4 Seguido

5 Muy seguido

8 Se rehúsa a contestar

Q369. Los trabajadores de salud no me han recetado medicamento que yo necesitaba. **(Escoja una)** (Elija Uno)

1 Nunca

2 No seguido

3 Algunas veces

4 Seguido

5 Muy seguido

8 Se rehúsa a contestar

READ: LEA LO SIGUIENTE AL PARTICIPANTE Y LUEGO PASE A LA PANTALLA SIGUIENTE Y LEA LAS AFIRMACIONES RELACIONADAS: ¿Qué tan probable es que las personas te vayan a tratar así en el futuro porque estás usando buprenorfina/suboxona?

ITEMS DE ESTIGMA ANTICIPADO (BSAS1-BSAS9)

Q370. Miembros de mi familia van a pensar que sigo siendo un usuario de drogas. **(Escoja una)** (Elija Uno)

1 Nunca

2 No seguido

3 Algunas veces

4 Seguido

5 Muy seguido

8 Se rehúsa a contestar

Q371. Miembros de mi familia no me apoyarán con mi tratamiento de buprenorfina/suboxona. **(Escoja una)** (Elija Uno)

1 Nunca

2 No seguido

3 Algunas veces

4 Seguido

5 Muy seguido

8 Se rehúsa a contestar

Q372. Miembros de mi familia pensarán que no puedo mejorar. **(Escoja una)** (Elija Uno)

1 Nunca

2 No seguido

3 Algunas veces

4 Seguido

5 Muy seguido

8 Se rehúsa a contestar

Q373. Los empleadores pensarán que sigo siendo un usuario de drogas. **(Escoja una)** (Elija Uno)

1 Nunca

2 No seguido

3 Algunas veces

4 Seguido

5 Muy seguido

8 Se rehúsa a contestar

Q374. Los empleadores pensarán que soy un mal trabajador. **(Escoja una)** (Elija Uno)

1 Nunca

2 No seguido

3 Algunas veces

4 Seguido

5 Muy seguido

8 Se rehúsa a contestar

Q375. Los empleadores pensarán que el manejar mi horario de dósis de buprenorfina/suboxona será un problema. **(Escoja una)** (Elija Uno)

1 Nunca

2 No seguido

3 Algunas veces

4 Seguido

5 Muy seguido

8 Se rehúsa a contestar

Q376. Los trabajadores de salud pensarán que sigo siendo un usuario de drogas. **(Escoja una)** (Elija Uno)

1 Nunca

2 No seguido

3 Algunas veces

4 Seguido

5 Muy seguido

8 Se rehúsa a contestar

Q377. Los trabajadores de salud me darán un mal trato. **(Escoja una)** (Elija Uno)

1 Nunca

2 No seguido

3 Algunas veces

4 Seguido

5 Muy seguido

8 Se rehúsa a contestar

Q378. Los trabajadores de salud no me van a recetar el medicamento que yo necesito. **(Escoja una)** (Elija Uno)

1 Nunca

2 No seguido

3 Algunas veces

4 Seguido

5 Muy seguido

8 Se rehúsa a contestar

ITEMS DE ESTIGMA INTERNALIZADO (BSIS1-BSIS7)

Q379. El recibir buprenorfina/suboxona me hace sentir que soy una mala persona. **(Escoja una)** (Elija Uno)

1 Nunca

2 No seguido

3 Algunas veces

4 Seguido

5 Muy seguido

8 Se rehúsa a contestar

Q380. Siento que no soy tan bueno/a como otras personas porque recibo metadona/buprenorfina/suboxona. **(Escoja una)** (Elija Uno)

1 Nunca

2 No seguido

3 Algunas veces

4 Seguido

5 Muy seguido

8 Se rehúsa a contestar

Q381. Me siento avergonzado/a de mi tratamiento de buprenorfina/suboxona. **(Escoja una)** (Elija Uno)

1 Nunca

2 No seguido

3 Algunas veces

4 Seguido

5 Muy seguido

8 Se rehúsa a contestar

Q382. Me respeto menos a mí mismo/a porque recibo buprenorfina/suboxona. **(Escoja una)** (Elija Uno)

1 Nunca

2 No seguido

3 Algunas veces

4 Seguido

5 Muy seguido

8 Se rehúsa a contestar

Q383. El recibir buprenorfina/suboxona me hace sentir sucio/a. **(Escoja una)** (Elija Uno)

1 Nunca

2 No seguido

3 Algunas veces

4 Seguido

5 Muy seguido

8 Se rehúsa a contestar

Q384. El usar tratamiento de buprenorfina/suboxona es algo desagradable/asqueroso para mí. **(Escoja una)** (Elija Uno)

1 Nunca

2 No seguido

3 Algunas veces

4 Seguido

5 Muy seguido

8 Se rehúsa a contestar

Q385. El usar tratamiento de buprenorfina/suboxona me hace sentir que sigo siendo un usuario de drogas. **(Escoja una)** (Elija Uno)

1 Nunca

2 No seguido

3 Algunas veces

4 Seguido

5 Muy seguido

8 Se rehúsa a contestar

If Q316 is not equal to 1 and Q15 is equal to 1 then skip to instruction before Q386

If not, then skip to Q404.

READ: LEA: LEA AL PARTICIPANTE: LA Buprenorfina y suboxona son medicamentos que tratan la adicción a la heroína y otros opioides. Reducen los síntomas de abstinencia y el antojo de droga pero no te harán sentir dopado. Estas preguntas serán sobre cómo podrías llegar a ser tratado/a en el futuro si tomas buprenorfina o suboxona.
Por favor selecciona una respuesta para cada pregunta. No te demores mucho pensando tu repuesta. Tu primera impresión usualmente es la mejor.

ESTIGMA POR TRATAMIENTO DE MANTENIMIENTO CON BUPRENORFINA/ SUBOXONA (18 items)-BMTS1-BMTS18. Nota: BMTS17 está codificada al revés.

Q386. ¿Qué tan preocupado estás que se te niegue alojamiento si tomas buprenorfina/suboxona? **(Escoja una)** (Elija Uno)

1 Nunca

2 No seguido

3 Algunas veces

4 Seguido

5 Muy seguido

8 Se rehúsa a contestar

Q387. ¿Qué tan preocupado estás que se te niegue trabajo si tomas buprenorfina/suboxona? **(Escoja una)** (Elija Uno)

1 Nunca

2 No seguido

3 Algunas veces

4 Seguido

5 Muy seguido

8 Se rehúsa a contestar

Q388. ¿Qué tan preocupado estás que se te niegue asistencia de gobierno o beneficios similares si tomas buprenorfina/suboxona? **(Escoja una)** (Elija Uno)

1 Nunca

2 No seguido

3 Algunas veces

4 Seguido

5 Muy seguido

8 Se rehúsa a contestar

READ: LEA LO SIGUIENTE AL PARTICIPANTE Y LUEGO PASE A LA PANTALLA SIGUIENTE Y LEA LAS AFIRMACIONES RELACIONADAS: ¿Qué tan probable es que las personas te traten así en el futuro si fueras a tomar buprenorfina/Suboxona?

Q389. Miembros de mi familia pensarán que sigo siendo usuario de drogas. **(Escoja una)** (Elija Uno)

1 Nunca

2 No seguido

3 Algunas veces

4 Seguido

5 Muy seguido

8 Se rehúsa a contestar

Q390. Miembros de mi familia no me apoyarán con mi tratamiento de buprenorfina/Suboxona. **(Escoja una)** (Elija Uno)

1 Nunca

2 No seguido

3 Algunas veces

4 Seguido

5 Muy seguido

8 Se rehúsa a contestar

Q391. Miembros de mi familia pensarán que no me puedo recuperar. **(Escoja una)** (Elija Uno)

1 Nunca

2 No seguido

3 Algunas veces

4 Seguido

5 Muy seguido

8 Se rehúsa a contestar

Q392. Los trabajadores de salud pensarán que sigo siendo un usuario de drogas. **(Escoja una)** (Elija Uno)

1 Nunca

2 No seguido

3 Algunas veces

4 Seguido

5 Muy seguido

8 Se rehúsa a contestar

Q393. Los trabajadores de salud me darán un mal trato. **(Escoja una)** (Elija Uno)

1 Nunca

2 No seguido

3 Algunas veces

4 Seguido

5 Muy seguido

8 Se rehúsa a contestar

Q394. Los trabajadores de salud no me recetarán el medicamento que necesito. **(Escoja una)** (Elija Uno)

1 Nunca

2 No seguido

3 Algunas veces

4 Seguido

5 Muy seguido

8 Se rehúsa a contestar

Q395. Otras personas que se inyectan drogas pensarán que soy débil si tomo buprenofina/Suboxona. **(Escoja una)** (Elija Uno)

1 Nunca

2 No seguido

3 Algunas veces

4 Seguido

5 Muy seguido

8 Se rehúsa a contestar

Q396. Otras personas que se inyectan drogas no me apoyarán si tomo buprenofina/Suboxona. **(Escoja una)** (Elija Uno)

1 Nunca

2 No seguido

3 Algunas veces

4 Seguido

5 Muy seguido

8 Se rehúsa a contestar

Q397. Otras personas que se inyectan drogas no me tendrán confianza si tomo buprenofina/Suboxona. **(Escoja una)** (Elija Uno)

1 Nunca

2 No seguido

3 Algunas veces

4 Seguido

5 Muy seguido

8 Se rehúsa a contestar

READ: LEA LO SIGUIENTE AL PARTICIPANTE Y LUEGO PASE A LA PANTALLA SIGUIENTE Y LEA LAS AFIRMACIONES RELACIONADAS: ¿Cómo te sientes sobre las personas que toman buprenorfina/saboxona?

Q398. Las personas que toman buprenorfina/suboxona están reemplazando una droga con otra. **(Escoja una)** (Elija Uno)

1 Totalmente en desacuerdo

2 En desacuerdo

3 Ni de acuerdo ni en desacuerdo

4 De acuerdo

5 Totalmente de acuerdo

8 Se rehúsa a contestar

Q399. Las personas que toman buprenorfina/suboxona no son de fiar/confiables**. (Escoja una)** (Elija Uno)

1 Totalmente en desacuerdo

2 En desacuerdo

3 Ni de acuerdo ni en desacuerdo

4 De acuerdo

5 Totalmente de acuerdo

8 Se rehúsa a contestar

Q400. Las personas que toman buprenorfina/suboxona no tienen control sobre sus propias vidas. **(Escoja una)** (Elija Uno)

1 Totalmente en desacuerdo

2 En desacuerdo

3 Ni de acuerdo ni en desacuerdo

4 De acuerdo

5 Totalmente de acuerdo

8 Se rehúsa a contestar

Q401. Las personas que toman buprenorfina/suboxona me hacen sentir incómodo/a. **(Escoja una)** (Elija Uno)

1 Totalmente en desacuerdo

2 En desacuerdo

3 Ni de acuerdo ni en desacuerdo

4 De acuerdo

5 Totalmente de acuerdo

8 Se rehúsa a contestar

Q402. Las personas que toman buprenorfina/suboxoná me dan esperanza. **(Escoja una)** (Elija Uno)

1 Totalmente en desacuerdo

2 En desacuerdo

3 Ni de acuerdo ni en desacuerdo

4 De acuerdo

5 Totalmente de acuerdo

8 Se rehúsa a contestar

Q403. Las personas que toman buprenorfina/suboxoná me hacen sentir enojado/a. **(Escoja una)** (Elija Uno)

1 Totalmente en desacuerdo

2 En desacuerdo

3 Ni de acuerdo ni en desacuerdo

4 De acuerdo

5 Totalmente de acuerdo

8 Se rehúsa a contestar

Q404. En la siguiente sección, se le preguntaran los nombres de las personas en su círculo social, también habrá preguntas acerca de su relación con su círculo y su uso de drogas y practicas variadas de personas que no están en el estudio (no han dado su consentimiento escrito). Esta información es muy importante para mejorar las estrategias de prevención de salud, específicamente la reducción de la transmisión de VIH en su comunidad. Sin embargo, existe la posibilidad de que al compartir información respecto a su red social sin consentimiento de aquellas personas que usted menciona en esta entrevista, sepan de su participación a través de especulación. Me gustaría asegurarle que en ningún momento vamos a contactar a nadie que usted mencione en su entrevista y que la información que nos dé es privada y confidencial. Siempre vamos a hacer el mayor esfuerzo para evitar la diseminación y el acceso no autorizado a la información que usted nos provea. Si se siente incómodo o incomoda de proveer nombres, puede darnos apodos o iniciales de las personas en su círculo social, o bien también tiene derecho a negarse a contestar cualquiera de las preguntas. **¿Tengo su consentimiento para proceder con estas preguntas?**

1 Si

0 No

8 Se rehúsa a contestar

If Q404 is equal to 1 then skip to instruction before Q405

If not, then skip to Q1150.

READ: LEA: DIGA LO SIGUIENTE: Para esta entrevista vamos a platicar sobre personas con las que te juntas frecuentemente y que consideras amigos/as. Voy a preguntarte sobre personas que tú conoces bien. Nosotros intentamos aprender sobre cómo las personas que están conectadas entre sí y cómo esas conexiones se relacionan con el prevenir la infección de VIH y otras enfermedades. El contar con nombres e información demográfica nos ayudará a ver cómo las personas en este estudio están conectadas unas con otras, es decir, a través de amigos en común. Por favor recuerda que todo lo que me digas será confidencial y que nosotros protegeremos tu privacidad y la privacidad de las personas que menciones. Nosotros nunca en ningún momento usaremos esta información para contactar a las personas de las que tú nos hables.
Específicamente, voy a preguntarte sobre cuántas personas conoces que hayas visto en los últimos 30 días, con quien hablas sobre cosas que son importantes para ti. Estas personas pueden ser tu pareja (novia/novio, esposa/esposo), familia, amigos/as, personas con las que usas drogas, o cualquier otra persona con quien tú te consideras cercano/a. Por ejemplo, una persona que te recuerde tomar tu medicamento, alguien a quien le puedes pedir dinero prestado, alguien con quien juntas drogas o dinero, alguien con quien usas drogas, o alguien con quien puedes hablar sobre el uso de drogas o problemas personales. Recuerda, solo estamos interesados en esas personas que has visto en los últimos 30 días.

Q405. Por favor dime ¿cuántos amigos o personas con las que tú te asocias y con quién hablas sobre cosas que son importantes para ti has visto en los últimos 30 días?

— — —

998 Se rehúsa a contestar

If Q405 is equal to 0 or Q405 is equal to 998, then skip to instruction before Q686.

READ: LEA: ¿Cuáles son los nombres de estas personas? Por favor comienza con los más cercanos a ti.
Entrevistador: Por favor escriba los nombres de las personas en una hoja de papel, primero, y luego el programa QDS le dará la instrucción de ingresar cada nombre.
SI EL PARTICIPANTE PARECE NO QUERER DAR EL NOMBRE COMPLETO DIGA: Si no quieres darnos su nombre completo puedes usar sus iniciales o solo su primer nombre o un apodo, para que tú puedas saber de quién hablas cuando te haga las preguntas de seguimiento sobre esta persona.
SI EL PARTICIPANTE ENUMERA MENOS DE 5 O SI ESTA TENIENDO PROBLEMAS NOMBRANDO A ALGUIEN, PREGUNTA, [¿A quién más deberías agregar?]
Por favor, piensa en la última vez o la anterior en las que tu hablaste con un amigo o pareja sobre cosas que son importantes para ti. ¿No has mencionado a alguno de ellos?
TRANSLATION

If Q405 is greater than 0 then skip to Q406

If not, then skip to instruction before Q408.

Q406. ¿Cuál es el nombre de la persona más cercana a Ud. con la que ha hablado sobre cosas importantes para Ud. y a quien ha visto en los últimos 30 días? **SI EL PARTICIPANTE MUESTRA RETICENCIA PARA DAR EL NOMBRE COMPLETO DE LA PERSONA, dígale lo siguiente:Si Ud. no quiere darnos su nombre completo, Ud puede darnos sus iniciales, o solo el primer nombre o su apodo para que así Ud pueda reconocer de quién estamos hablando cuando le hagamos mas preguntas sobre ésta persona. Si el participante le dá solo el apodo o las iniciales, anotelas aquí y en la siguiente pantalla.**

____

If Q406 is equal to "Se rehúsa a contestar", then skip to instruction before Q446.

Q407. ¿Me puedes decir el apodo de [Response to Q406], si es que tienen un apodo? Entrevistador, por favor anotar el apodo.

____

If Q405 is greater than 1 then skip to Q408

If not, then skip to instruction before Q410.

Q408. Entonces, el nombre de la priemra persona con la que hablas sobre cosas que son importantes para ti y a quien has visto en los últimos 30 días es [Response to Q406]. ¿Cuál es el nombre de la 2da persona con la que hablas y has visto en los últimos 30 días? **Entrevistador: Si el participante tiene dificultad para nombrar a otra persona, por favor dígale lo siguiente: "Por favor piense en las últimas veces que tuvo que hablar con un familiar, amigo/a o pareja sobre cosas que eran importantes para Ud. ¿Puede recordar sus nombres?" SI EL PARTICIPANTE MUESTRA RETICENCIA PARA DAR EL NOMBRE COMPLETO DE LA PERSONA, dígale que le puede dar las iniciales del nombre de la persona o su apodo.**

____

If Q408 is equal to "Se rehúsa a contestar", then skip to instruction before Q446.

Q409. ¿Me puedes decir el apodo de [Response to Q408], si es que tiene un apodo? Entrevistador, por favor anotar el apodo.

____

If Q405 is greater than 2 then skip to Q410

If not, then skip to instruction before Q412.

Q410. Hasta ahora has nombrado a [Response to Q406] y a [Response to Q408]. ¿Cuál es el nombre de la 3ra persona? **Entrevistador: Si el participante tiene dificultad para nombrar a otra persona, por favor dígale lo siguiente: "Por favor piense en las últimas veces que tuvo que hablar con un familiar, amigo/a o pareja sobre cosas que eran importantes para Ud. ¿Puede recordar sus nombres?" SI EL PARTICIPANTE MUESTRA RETICENCIA PARA DAR EL NOMBRE COMPLETO DE LA PERSONA, dígale que le puede dar las iniciales del nombre de la persona o su apodo.**

____

If Q410 is equal to "Se rehúsa a contestar", then skip to instruction before Q446.

Q411. ¿Me puedes decir el apodo de [Response to Q410], si es que tiene un apodo? Entrevistador, por favor anotar el apodo.

____

If Q405 is greater than 3 then skip to Q412

If not, then skip to instruction before Q414.

Q412. Hasta ahora has nombrado a [Response to Q406], [Response to Q408] y a [Response to Q410]. ¿Cuál es el nombre de la 4ta persona? **Entrevistador: Si el participante tiene dificultad para nombrar a otra persona, por favor dígale lo siguiente: "Por favor piense en las últimas veces que tuvo que hablar con un familiar, amigo/a o pareja sobre cosas que eran importantes para Ud. ¿Puede recordar sus nombres?" SI EL PARTICIPANTE MUESTRA RETICENCIA PARA DAR EL NOMBRE COMPLETO DE LA PERSONA, dígale que le puede dar las iniciales del nombre de la persona o su apodo.**

____

If Q412 is equal to "Se rehúsa a contestar", then skip to instruction before Q446.

Q413. ¿Me puedes decir el apodo de [Response to Q412], si es que tiene un apodo? Entrevistador, por favor anotar el apodo.

____

If Q405 is greater than 4 then skip to Q414

If not, then skip to instruction before Q416.

Q414. Hasta ahora has nombrado a [Response to Q406], [Response to Q408], [Response to Q410] y a [Response to Q412]. ¿Cuál es el nombre de la 5ta persona? **Entrevistador: Si el participante tiene dificultad para nombrar a otra persona, por favor dígale lo siguiente: "Por favor piense en las últimas veces que tuvo que hablar con un familiar, amigo/a o pareja sobre cosas que eran importantes para Ud. ¿Puede recordar sus nombres?" SI EL PARTICIPANTE MUESTRA RETICENCIA PARA DAR EL NOMBRE COMPLETO DE LA PERSONA, dígale que le puede dar las iniciales del nombre de la persona o su apodo.**

____

If Q414 is equal to "Se rehúsa a contestar", then skip to instruction before Q446.

Q415. ¿Me puedes decir el apodo de [Response to Q414], si es que tiene un apodo? Entrevistador, por favor anotar el apodo.

____

If Q405 is greater than 5 then skip to Q416

If not, then skip to instruction before Q418.

Q416. Hasta ahora has nombrado a [Response to Q406], [Response to Q408], [Response to Q410], [Response to Q412] y a [Response to Q414]. ¿Cuál es el nombre de la 6ta persona?

____

If Q416 is equal to "Se rehúsa a contestar", then skip to instruction before Q446.

Q417. ¿Me puedes decir el apodo de [Response to Q416], si es que tiene un apodo? Entrevistador, por favor anotar el apodo.

____

If Q405 is greater than 6 then skip to Q418

If not, then skip to instruction before Q420.

Q418. Hasta ahora has nombrado a [Response to Q406], [Response to Q408], [Response to Q410], [Response to Q412], [Response to Q414] y a [Response to Q416]. ¿Cuál es el nombre de la 7ma persona?

____

If Q418 is equal to "Se rehúsa a contestar", then skip to instruction before Q446.

Q419. ¿Me puedes decir el apodo de [Response to Q418], si es que tiene un apodo? Entrevistador, por favor anotar el apodo.

____

If Q405 is greater than 7 then skip to Q420

If not, then skip to instruction before Q422.

Q420. Hasta ahora has nombrado a [Response to Q406], [Response to Q408], [Response to Q410], [Response to Q412], [Response to Q414], [Response to Q416] y a [Response to Q418]. ¿Cuál es el nombre de la 8va persona?

____

If Q420 is equal to "Se rehúsa a contestar", then skip to instruction before Q446.

Q421. ¿Me puedes decir el apodo de [Response to Q420], si es que tiene un apodo? Entrevistador, por favor anotar el apodo.

____

If Q405 is greater than 8 then skip to Q422

If not, then skip to instruction before Q424.

Q422. Hasta ahora has nombrado a [Response to Q406], [Response to Q408], [Response to Q410], [Response to Q412], [Response to Q414], [Response to Q416], [Response to Q418] y a [Response to Q420]. ¿Cuál es el nombre de la 9na persona?

____

If Q422 is equal to "Se rehúsa a contestar", then skip to instruction before Q446.

Q423. ¿Me puedes decir el apodo de [Response to Q422], si tienen uno? Entrevistador, por favor ingresar apodo.

____

If Q405 is greater than 9 then skip to Q424

If not, then skip to instruction before Q426.

Q424. Hasta ahora has nombrado a [Response to Q406], [Response to Q408], [Response to Q410], [Response to Q412], [Response to Q414], [Response to Q416], [Response to Q418], [Response to Q420] y a [Response to Q422]. ¿Cuál es el nombre de la 10a persona?

____

If Q424 is equal to "Se rehúsa a contestar", then skip to instruction before Q446.

Q425. ¿Me puedes decir el apodo de [Response to Q424], si es que tiene un apodo? Entrevistador, por favor anotar el apodo.

____

If Q405 is greater than 10 then skip to Q426

If not, then skip to instruction before Q428.

Q426. Hasta ahora has nombrado a [Response to Q406], [Response to Q408], [Response to Q410], [Response to Q412], [Response to Q414], [Response to Q416], [Response to Q418], [Response to Q420], [Response to Q422] y a [Response to Q424]. ¿Cuál es el nombre de la 11a persona?

____

If Q426 is equal to "Se rehúsa a contestar", then skip to instruction before Q446.

Q427. ¿Me puedes decir el apodo de [Response to Q426], si es que tiene un apodo? Entrevistador, por favor anotar el apodo.

____

If Q405 is greater than 11 then skip to Q428

If not, then skip to instruction before Q430.

Q428. Hasta ahora has nombrado a [Response to Q406], [Response to Q408], [Response to Q410], [Response to Q412], [Response to Q414], [Response to Q416], [Response to Q418], [Response to Q420], [Response to Q422], [Response to Q424] y a [Response to Q426]. ¿Cuál es el nombre de la 12a persona?

____

If Q428 is equal to "Se rehúsa a contestar", then skip to instruction before Q446.

Q429. ¿Me puedes decir el apodo de [Response to Q428], si es que tiene un apodo? Entrevistador, por favor anotar el apodo.

____

If Q405 is greater than 12 then skip to Q430

If not, then skip to instruction before Q432.

Q430. Hasta ahora has nombrado a [Response to Q406], [Response to Q408], [Response to Q410], [Response to Q412], [Response to Q414], [Response to Q416], [Response to Q418], [Response to Q420], [Response to Q422], [Response to Q424], [Response to Q426] y a [Response to Q428]. ¿Cuál es el nombre de la 13a persona?

____

If Q430 is equal to "Se rehúsa a contestar", then skip to instruction before Q446.

Q431. ¿Me puedes decir el apodo de [Response to Q430], si es que tiene un apodo? Entrevistador, por favor anotar el apodo.

____

If Q405 is greater than 13 then skip to Q432

If not, then skip to instruction before Q434.

Q432. Hasta ahora has nombrado a [Response to Q406], [Response to Q408], [Response to Q410], [Response to Q412], [Response to Q414], [Response to Q416], [Response to Q418], [Response to Q420], [Response to Q422], [Response to Q424], [Response to Q426], [Response to Q428] y a [Response to Q430]. ¿Cuál es el nombre de la 14a persona?

____

If Q432 is equal to "Se rehúsa a contestar", then skip to instruction before Q446.

Q433. ¿Me puedes decir el apodo de [Response to Q432], si es que tiene un apodo? Entrevistador, por favor anotar el apodo.

____

If Q405 is greater than 14 then skip to Q434

If not, then skip to instruction before Q436.

Q434. Hasta ahora has nombrado a [Response to Q406], [Response to Q408], [Response to Q410], [Response to Q412], [Response to Q414], [Response to Q416], [Response to Q418], [Response to Q420], [Response to Q422], [Response to Q424], [Response to Q426], [Response to Q428], [Response to Q430] y a [Response to Q432]. ¿Cuál es el nombre de la 15a persona?

____

If Q434 is equal to "Se rehúsa a contestar", then skip to instruction before Q446.

Q435. ¿Me puedes decir el apodo de [Response to Q434], si es que tiene un apodo? Entrevistador, por favor anotar el apodo.

____

If Q405 is greater than 15 then skip to Q436

If not, then skip to instruction before Q438.

Q436. Hasta ahora has nombrado a [Response to Q406], [Response to Q408], [Response to Q410], [Response to Q412], [Response to Q414], [Response to Q416], [Response to Q418], [Response to Q420], [Response to Q422], [Response to Q424], [Response to Q426], [Response to Q428], [Response to Q430], [Response to Q432], y a [Response to Q434]. ¿Cuál es el nombre de la 16a persona?
Nombre de la 16a persona. TRANSLATION

____

If Q436 is equal to "Se rehúsa a contestar", then skip to instruction before Q446.

Q437. ¿Me puedes decir el apodo de [Response to Q436], si es que tiene un apodo? Entrevistador, por favor anotar el apodo.

____

If Q405 is greater than 16 then skip to Q438

If not, then skip to instruction before Q440.

Q438. Hasta ahora has nombrado a [Response to Q406], [Response to Q408], [Response to Q410], [Response to Q412], [Response to Q414], [Response to Q416], [Response to Q418], [Response to Q420], [Response to Q422], [Response to Q424], [Response to Q426], [Response to Q428], [Response to Q430], [Response to Q432], [Response to Q434] and [Response to Q436]. ¿Cuál es el nombre de la 17a persona?

____

If Q438 is equal to "Se rehúsa a contestar", then skip to instruction before Q446.

Q439. ¿Me puedes decir el apodo de [Response to Q438], si es que tiene un apodo? Entrevistador, por favor anotar el apodo.

____

If Q405 is greater than 17 then skip to Q440

If not, then skip to instruction before Q442.

Q440. Hasta ahora has nombrado a [Response to Q406], [Response to Q408], [Response to Q410], [Response to Q412], [Response to Q414], [Response to Q416], [Response to Q418], [Response to Q420], [Response to Q422], [Response to Q424], [Response to Q426], [Response to Q428], [Response to Q430], [Response to Q432], [Response to Q434], [Response to Q436] y a [Response to Q438]. ¿Cuál es el nombre de la 18a persona?

____

If Q440 is equal to "Se rehúsa a contestar", then skip to instruction before Q446.

Q441. ¿Me puedes decir el apodo de [Response to Q440], si es que tiene un apodo? Entrevistador, por favor anotar el apodo.

____

If Q405 is greater than 18 then skip to Q442

If not, then skip to instruction before Q444.

Q442. Hasta ahora has nombrado a [Response to Q406], [Response to Q408], [Response to Q410], [Response to Q412], [Response to Q414], [Response to Q416], [Response to Q418], [Response to Q420], [Response to Q422], [Response to Q424], [Response to Q426], [Response to Q428], [Response to Q430], [Response to Q432], [Response to Q434], [Response to Q436], [Response to Q438], y a [Response to Q440]. ¿Cuál es el nombre de la 19a persona?

____

If Q442 is equal to "Se rehúsa a contestar", then skip to instruction before Q446.

Q443. ¿Me puedes decir el apodo de [Response to Q442], si es que tiene un apodo? Entrevistador, por favor anotar el apodo.

____

If Q405 is greater than 19 then skip to Q444

If not, then skip to instruction before Q446.

Q444. Hasta ahora has nombrado a [Response to Q406], [Response to Q408], [Response to Q410], [Response to Q412], [Response to Q414], [Response to Q416], [Response to Q418], [Response to Q420], [Response to Q422], [Response to Q424], [Response to Q426], [Response to Q428], [Response to Q430], [Response to Q432], [Response to Q434], [Response to Q436], [Response to Q438], [Response to Q440], y a [Response to Q442]. ¿Cuál es el nombre de la 20a persona?

____

If Q444 is equal to "Se rehúsa a contestar", then skip to instruction before Q446.

Q445. ¿Me puedes decir el apodo de [Response to Q444], si es que tiene un apodo? Entrevistador, por favor anotar el apodo.

____

READ: LEA: Ahora te haré una serie de preguntas sobre las primeras cinco personas que mencionaste. Estas preguntas son sobre información básica. Recuerda que todo lo que me digas es confidencial y que nosotros protegeremos tu privacidad y la privacidad de las personas a las que menciones.

If Q405 is greater than 0 and Q406 is not equal to "Se rehúsa a contestar" then skip to Q446

If not, then skip to instruction before Q468.

Q446. ¿Cuál es la edad de [Response to Q406]? __ __

97 No Sabe

98 Se rehúsa a contestar

Q447. ¿Cuánto hace que conoces a [Response to Q406]? __ __ AñOS

__ __ MESES

__ __ DíAS

97 No Sabe (Años)

98 Se rehúsa a contestar (Años)

If Q447 is greater than Q446 then READ: "El participante dice que ha conocido a esta persona por un tiempo más largo que la edad de la persona. Lo estamos redireccionando para que se hagan las correcciones necesarias." and skip to Q446.

Q448. ¿Cuál es tu relación con [Response to Q406]? (Elija Uno)

1 Romántica o pareja sexual

2 Amigo/a

3 Vecino/a o compañero de casa

4 Compañero/a de trabajo o jefe/a

5 Familia - familiares de sangre o por matrimonio

6 Relacionado con drogas o alcohol

7 OTRO [especifica]

8 Se rehúsa a contestar

If Q448 is equal to 1 then skip to Q449

If not, then skip to instruction before Q450.

Q449. Es [Response to Q406] tu: (Elija Uno)

1 Esposo/a

2 Pareja romántica actual

3 Expareja romántica

4 Pareja sexual (no romántica)

5 OTRO [especifica]

8 Se rehúsa a contestar

If Q449 is equal to 5 then skip to Q450

If not, then skip to instruction before Q451.

Q450. Por favor especifica tu relación con [Response to Q406].

____

If Q448 is equal to 2 then skip to Q451

If not, then skip to instruction before Q452.

Q451. Es [Response to Q406] tu: (Elija Uno)

1 Mejor amigo/a
2 Amigo/a cercano/a
3 Amigo/a
4 Conocido/a
5 OTRO [especifica]
8 Se rehúsa a contestar

If Q451 is equal to 5 then skip to Q452

If not, then skip to instruction before Q453.

Q452. Por favor especifica tu relación con [Response to Q406]

____

If Q448 is equal to 3 then skip to Q453

If not, then skip to instruction before Q454.

Q453. Es [Response to Q406] tu: (Elija Uno)

1 Vecino/a
2 Compañero de cuarto o casa
3 OTRO [especifica]
8 Se rehúsa a contestar

If Q453 is equal to 3 then skip to Q454

If not, then skip to instruction before Q455.

Q454. Por favor especifica tu relación con [Response to Q406]

____

If Q448 is equal to 4 then skip to Q455

If not, then skip to instruction before Q456.

Q455. Es [Response to Q406] tu: (Elija Uno)

1 Compañero/a de trabajo

2 Jefe/a

3 OTRO [especifica]

8 Se rehúsa a contestar

If Q455 is equal to 3 then skip to Q456

If not, then skip to instruction before Q457.

Q456. Por favor especifica tu relación con [Response to Q406].

____

If Q448 is equal to 5 then skip to Q457

If not, then skip to instruction before Q458.

Q457. Es [Response to Q406] tu: (Elija Uno)

1 Padre/Madre/Padrastro/Madrastra

2 Hijo/a

3 Hermano/a o Medio-Hermano/a

4 Cuñado o cuñada

5 OTRO FAMILIAR [especifica]

8 Se rehúsa a contestar

If Q457 is equal to 5 then skip to Q458

If not, then skip to instruction before Q459.

Q458. Por favor especifica tu relación con [Response to Q406].

____

If Q448 is equal to 6 then skip to Q459

If not, then skip to instruction before Q460.

Q459. Es [Response to Q406] tu: (Elija Uno)

1 Camarero/a

2 Distribuidor de drogas

3 Alguien quien inyecta a otros por una tarifa o por drogas

4 Cliente de venta de drogas (alguien a quien le vendes droga)

5 Compañero/a para beber

6 Alguien con quien tu usas drogas

7 Otro

8 Se rehúsa a contestar

If Q459 is equal to 7 then skip to Q460

If not, then skip to instruction before Q461.

Q460. Por favor especifica tu relación con [Response to Q406].

____

If Q448 is equal to 7 then skip to Q461

If not, then skip to instruction before Q462.

Q461. Es [Response to Q406] tu: (Elija Uno)

1 Consejero religioso o espiritual

2 Maestro u otro tipo de educador

3 Profesional Medico

4 Profesional en salud mental

5 OTRO [especifica]

8 Se rehúsa a contestar

If Q461 is equal to 5 then skip to Q462

If not, then skip to instruction before Q463.

Q462. Por favor especifica tu relación con [Response to Q406].

____

If Q448 is not equal to 5 and Q448 is not equal to 8 then skip to Q463

If not, then skip to Q464.

Q463. ¿Tuviste sexo vaginal o anal con [Response to Q406] en los 6 meses anteriores?

1 Si

0 No

8 Se rehúsa a contestar

Q464. ¿Es [Respuesta a Q330] hombre, mujer o transgénero, no binario u otro? (Elija Uno)

1 Hombre

2 Mujer

3 Mujer transgénero (hombre a mujer)

4 Hombre transgénero (mujer a hombre)

5 No binario/género fludo/ sin género

6 Otra identidad no incluída

8 Se rehúsa a contestar

Q465. Por favor díme qué tan seguido te comunicas con [Respuesta a Q330] (en persona, por teléfono, correo electrónico y/o por redes sociales, como Facebook) (Elija Uno)

01	Todos los días
02	Varias veces a la semana
03	Una vez a la semana
04	Una vez cada 2 semanas
05	Una vez al mes
06	Varias veces al año
07	Una vez al año
08	Menos de una vez al año
98	Se rehúsa a contestar

Q466. ¿Qué tan cercano/a te sientes a [Response to Q406]? (Elija Uno)

1	Muy cercano/a
2	Algo cercano/a
3	No tan cercano
4	No cercano
8	Se rehúsa a contestar

Q467. ¿Cuánto hace que te sientes así de cercano/a con [Respuesta a Q330]?

__ __	AñOS
__ __	MESES
__ __	DíAS
97	No Sabe (Años)
98	Se rehúsa a contestar (Años)

If Q467 is greater than Q447 and Q447 is not equal to 97 and Q447 is not equal to 98 then READ: "La respuesta es mayor a las veces que tú nos dijiste que conoces a &[NAME1]. Por favor checa la respuesta." and skip to Q467.

If Q405 is greater than 1 and Q408 is not equal to "Se rehúsa a contestar" and Q408 is not equal to "Saltó" then skip to Q468

If not, then skip to instruction before Q490.

Q468. ¿Cuál es la edad de [Response to Q408]? __ __

97 No Sabe

98 Se rehúsa a contestar

Q469. ¿Cuánto hace que conoces a [Respuesta a Q332]?

__ __ AñOS

__ __ MESES

__ __ DíAS

97 No Sabe (Años)

98 Se rehúsa a contestar (Años)

If Q469 is greater than Q468 then READ: "El participante dice que ha conocido a esta persona por un tiempo más largo que la edad de la persona. Lo estamos redireccionando para que se hagan las correcciones necesarias." and skip to Q468.

Q470. ¿Cuál es tu relación con [Response to Q408]? (Elija Uno)

1 Romántica o pareja sexual

2 Amigo/a

3 Vecino/a o compañero de casa

4 Compañero/a de trabajo o jefe/a

5 Familia - familiares de sangre o por matrimonio

6 Relacionado con drogas o alcohol

7 OTRO [especifica]

8 Se rehúsa a contestar

If Q470 is equal to 1 then skip to Q471

If not, then skip to instruction before Q472.

Q471. Es [Response to Q408] tu: (Elija Uno)

1 Esposo/a

2 Pareja romántica actual

3 Expareja romántica

4 Pareja sexual (no romántica)

5 OTRO [especifica]

8 Se rehúsa a contestar

If Q471 is equal to 5 then skip to Q472

If not, then skip to instruction before Q473.

Q472. Por favor especifica tu relación con [Response to Q408].

____

If Q470 is equal to 2 then skip to Q473

If not, then skip to instruction before Q474.

Q473. Es [Response to Q408] tu: (Elija Uno)

1 Mejor amigo/a

2 Amigo/a cercano/a

3 Amigo/a

4 Conocido/a

5 OTRO [especifica]

8 Se rehúsa a contestar

If Q473 is equal to 5 then skip to Q474

If not, then skip to instruction before Q475.

Q474. Por favor especifica tu relación con [Response to Q408].

____

If Q470 is equal to 3 then skip to Q475

If not, then skip to instruction before Q476.

Q475. Es [Response to Q408] tu: (Elija Uno)

1 Vecino/a

2 Compañero de cuarto o casa

3 OTRO [especifica]

8 Se rehúsa a contestar

If Q475 is equal to 3 then skip to Q476

If not, then skip to instruction before Q477.

Q476. Por favor especifica tu relación con [Response to Q408].

____

If Q470 is equal to 4 then skip to Q477

If not, then skip to instruction before Q478.

Q477. Es [Response to Q408] tu: (Elija Uno)

1 Compañero/a de trabajo

2 Jefe/a

3 OTRO [especifica]

8 Se rehúsa a contestar

If Q477 is equal to 3 then skip to Q478

If not, then skip to instruction before Q479.

Q478. Por favor especifica tu relación con [Response to Q408].

____

If Q470 is equal to 5 then skip to Q479

If not, then skip to instruction before Q480.

Q479. Es [Response to Q408] tu: (Elija Uno)

1 Padre/Madre/Padrastro/Madrastra

2 Hijo/a

3 Hermano/a o Medio-Hermano/a

4 Cuñado o cuñada

5 OTRO FAMILIAR [especifica]

8 Se rehúsa a contestar

If Q479 is equal to 5 then skip to Q480

If not, then skip to instruction before Q481.

Q480. Por favor especifica tu relación con [Response to Q408].

____

If Q470 is equal to 6 then skip to Q481

If not, then skip to instruction before Q482.

Q481. Es [Response to Q408] tu: (Elija Uno)

1 Camarero/a

2 Distribuidor de drogas

3 Alguien quien inyecta a otros por una tarifa o por drogas

4 Cliente de venta de drogas (alguien a quien le vendes droga)

5 Compañero/a para beber

6 Alguien con quien tú usas drogas

7 OTRO [especifica]

8 Se rehúsa a contestar

If Q481 is equal to 7 then skip to Q482

If not, then skip to instruction before Q483.

Q482. Por favor especifica tu relación con [Response to Q408].

____

If Q470 is equal to 7 then skip to Q483

If not, then skip to instruction before Q484.

Q483. Es [Response to Q408] tu: (Elija Uno)

1	Consejero religioso o espiritual
2	Maestro u otro tipo de educador
3	Profesional Medico
4	Profesional en salud mental
5	OTRO [especifica]
8	Se rehúsa a contestar

If Q483 is equal to 5 then skip to Q484

If not, then skip to instruction before Q485.

Q484. Por favor especifica tu relación con [Response to Q408].

____

If Q470 is not equal to 5 and Q470 is not equal to 8 then skip to Q485

If not, then skip to Q486.

Q485. ¿Tuviste sexo vaginal o anal con [Response to Q408] en los 6 meses anteriores?

1	Si
0	No
8	Se rehúsa a contestar

Q486. ¿Es [Respuesta a Q332] hombre, mujer o transgénero? (Elija Uno)

1	Hombre
2	Mujer
3	Mujer transgénero
4	Hombre transgénero
5	No binario/género fluido/ agénero
6	Otro no en la lista
8	Se rehúsa a contestar

Q487. Por favor dime que tan seguido te comunicas con [Response to Q408] (por teléfono, correo electrónico y/o por redes sociales, como Facebook) (Elija Uno)

01	Todos los días
02	Varias veces a la semana
03	Una vez a la semana
04	Una vez cada 2 semanas
05	Una vez al mes
06	Varias veces al año
07	Una vez al año
08	Menos de una vez al año
98	Se rehúsa a contestar

Q488. ¿Qué tan cercano/a te sientes a [Response to Q408]? (Elija Uno)

1	Muy cercano/a
2	Algo cercano/a
3	No tan cercano
4	No cercano
8	Se rehúsa a contestar

Q489. ¿Cuánto llevas de sentirte así de cercano/a con [Response to Q408]?

__ __	AñOS
__ __	MESES
__ __	DíAS
97	No Sabe (Años)
98	Se rehúsa a contestar (Años)

If Q489 is greater than Q469 and Q469 is not equal to 97 and Q469 is not equal to 98 then READ: "La respuesta es mayor a las veces que tú nos dijiste que conoces a &[NAME2]. Por favor revisa la respuesta." and skip to Q489.

If Q405 is greater than 2 and Q410 is not equal to "Se rehúsa a contestar" and Q410 is not equal to "Saltó" then skip to Q490

If not, then skip to instruction before Q512.

Q490. ¿Cuál es la edad de [Response to Q410]? __ __

97	No Sabe
98	Se rehúsa a contestar

Q491. ¿Cuánto hace que conoces [Response to Q410]?

__ __	AñOS
__ __	MESES
__ __	DíAS
97	No Sabe (Años)
98	Se rehúsa a contestar (Años)

If Q491 is greater than Q490 then READ: "El participante dice que ha conocido a esta persona por un tiempo más largo que la edad de la persona. Lo estamos redireccionando para que se hagan las correcciones necesarias." and skip to Q490.

Q492. ¿Cuál es tu relación con [Response to Q410]? (Elija Uno)

1	Romántica o pareja sexual
2	Amigo/a
3	Vecino/a o compañero de casa
4	Compañero/a de trabajo o jefe/a
5	Familia - familiares de sangre o por matrimonio
6	Relacionado con drogas o alcohol
7	OTRO [especifica]
8	Se rehúsa a contestar

If Q492 is equal to 1 then skip to Q493

If not, then skip to instruction before Q494.

Q493. Es [Response to Q410] tu: (Elija Uno)

1	Esposo/a
2	Pareja romántica actual
3	Expareja romántica
4	Pareja sexual (no romántica)
5	OTRO [especifica]
8	Se rehúsa a contestar

If Q493 is equal to 5 then skip to Q494

If not, then skip to instruction before Q495.

Q494. Por favor especifica tu relación con [Response to Q410].

____

If Q492 is equal to 2 then skip to Q495

If not, then skip to instruction before Q496.

Q495. Es [Response to Q410] tu: (Elija Uno)

1 Mejor amigo/a

2 Amigo/a cercano/a

3 Amigo/a

4 Conocido/a

5 OTRO [especifica]

8 Se rehúsa a contestar

If Q495 is equal to 5 then skip to Q496

If not, then skip to instruction before Q497.

Q496. Por favor especifica tu relación con [Response to Q410].

____

If Q492 is equal to 3 then skip to Q497

If not, then skip to instruction before Q498.

Q497. Es [Response to Q410] tu: (Elija Uno)

1 Vecino/a

2 Compañero de cuarto o casa

3 OTRO [especifica]

8 Se rehúsa a contestar

If Q497 is equal to 3 then skip to Q498

If not, then skip to instruction before Q499.

Q498. Por favor especifica tu relación con [Response to Q410].

____

If Q492 is equal to 4 then skip to Q499

If not, then skip to instruction before Q500.

Q499. Es [Response to Q410] tu: (Elija Uno)

1 Compañero/a de trabajo

2 Jefe/a

3 OTRO [especifica]

8 Se rehúsa a contestar

If Q499 is equal to 3 then skip to Q500

If not, then skip to instruction before Q501.

Q500. Por favor especifica tu relación con [Response to Q410].

____

If Q492 is equal to 5 then skip to Q501

If not, then skip to instruction before Q502.

Q501. Es [Response to Q410] tu: (Elija Uno)

1 Padre/Madre/Padrastro/Madrastra

2 Hijo/a

3 Hermano/a o Medio-Hermano/a

4 Cuñado o cuñada

5 OTRO FAMILIAR [especifica]

8 Se rehúsa a contestar

If Q501 is equal to 5 then skip to Q502

If not, then skip to instruction before Q503.

Q502. Por favor especifica tu relación con [Response to Q410].

____

If Q492 is equal to 6 then skip to Q503

If not, then skip to instruction before Q504.

Q503. Es [Response to Q410] tu: (Elija Uno)

1 Camarero/a

2 Distribuidor de drogas

3 Alguien quien inyecta a otros por una tarifa o por drogas

4 Cliente de venta de drogas (alguien a quien le vendes droga)

5 Compañero/a para beber

6 Alguien con quien tú usas drogas

7 OTRO [especifica]

8 Se rehúsa a contestar

If Q503 is equal to 7 then skip to Q504

If not, then skip to instruction before Q505.

Q504. Por favor especifica tu relación con [Response to Q410].

____

If Q492 is equal to 7 then skip to Q505

If not, then skip to instruction before Q506.

Q505. Es [Response to Q410] tu: (Elija Uno)

1 Consejero religioso o espiritual

2 Maestro u otro tipo de educador

3 Profesional Medico

4 Profesional en salud mental

5 OTRO [especifica]

8 Se rehúsa a contestar

If Q505 is equal to 5 then skip to Q506

If not, then skip to instruction before Q507.

Q506. Por favor especifica tu relación con [Response to Q410].

____

If Q492 is not equal to 5 and Q492 is not equal to 8 then skip to Q507

If not, then skip to Q508.

Q507. ¿Tuviste sexo vaginal o anal con [Response to Q410] en los 6 meses anteriores?

1 Si

0 No

8 Se rehúsa a contestar

Q508. Es ¿[Response to Q410] hombre, mujer o transgénero? (Elija Uno)

1 Hombre

2 Mujer

3 Mujer transgénero

4 Hombre transgénero

5 No binario/género fluido/ agénero

6 Otro no en la lista

8 Se rehúsa a contestar

Q509. Por favor dime qué tan seguido te comunicas con [Response to Q410] (por teléfono, correo electrónico y/o por redes sociales, como Facebook) (Elija Uno)

01 Todos los días

02 Varias veces a la semana

03 Una vez a la semana

04 Una vez cada 2 semanas

05 Una vez al mes

06 Varias veces al año

07 Una vez al año

08 Menos de una vez al año

98 Se rehúsa a contestar

Q510. ¿Qué tan cercano/a te sientes a [Response to Q410]? (Elija Uno)

1	Muy cercano/a
2	Algo cercano/a
3	No tan cercano
4	No cercano
8	Se rehúsa a contestar

Q511. ¿Cuánto llevas de sentirte así de cercano/a con [Response to Q410]?

__ __	AñOS
__ __	MESES
__ __	DíAS
97	No Sabe (Años)
98	Se rehúsa a contestar (Años)

If Q511 is greater than Q491 and Q491 is not equal to 97 and Q491 is not equal to 98 then READ: "La respuesta es mayor a las veces que tú nos dijiste que conoces a &[NAME3]. Por favor revisa la respuesta." and skip to Q511.

If Q405 is greater than 3 and Q412 is not equal to "Se rehúsa a contestar" and Q412 is not equal to "Saltó" then skip to Q512

If not, then skip to instruction before Q534.

Q512. ¿Cuál es la edad de [Response to Q412]? __ __

97	No Sabe
98	Se rehúsa a contestar

Q513. ¿Cuánto hace que conoces a [Response to Q412]?

__ __	AñOS
__ __	MESES
__ __	DíAS
97	No Sabe (Años)
98	Se rehúsa a contestar (Años)

If Q513 is greater than Q512 then READ: "El participante dice que ha conocido a esta persona por un tiempo más largo que la edad de la persona. Lo estamos redireccionando para que se hagan las correcciones necesarias." and skip to Q512.

Q514. ¿Cuál es tu relación con [Response to Q412]? (Elija Uno)

1	Romántica o pareja sexual
2	Amigo/a
3	Vecino/a o compañero de casa
4	Compañero/a de trabajo o jefe/a
5	Familia - familiares de sangre o por matrimonio
6	Relacionado con drogas o alcohol
7	OTRO [especifica]
8	Se rehúsa a contestar

If Q514 is equal to 1 then skip to Q515

If not, then skip to instruction before Q516.

Q515. Es [Response to Q412] tu: (Elija Uno)

1	Esposo/a
2	Pareja romántica actual
3	Expareja romántica
4	Pareja sexual (no romántica)
5	OTRO [especifica]
8	Se rehúsa a contestar

If Q515 is equal to 5 then skip to Q516

If not, then skip to instruction before Q517.

Q516. Por favor especifica tu relación con [Response to Q412].

____

If Q514 is equal to 2 then skip to Q517

If not, then skip to instruction before Q518.

Q517. Es [Response to Q412] tu: (Elija Uno)

1 Mejor amigo/a

2 Amigo/a cercano/a

3 Amigo/a

4 Conocido/a

5 OTRO [especifica]

8 Se rehúsa a contestar

If Q517 is equal to 5 then skip to Q518

If not, then skip to instruction before Q519.

Q518. Por favor especifica tu relación con [Response to Q412].

____

If Q514 is equal to 3 then skip to Q519

If not, then skip to instruction before Q520.

Q519. Es [Response to Q412] tu: (Elija Uno)

1 Vecino/a

2 Compañero de cuarto o casa

3 OTRO [especifica]

8 Se rehúsa a contestar

If Q519 is equal to 3 then skip to Q520

If not, then skip to instruction before Q521.

Q520. Por favor especifica tu relación con [Response to Q412].

____

If Q514 is equal to 4 then skip to Q521

If not, then skip to instruction before Q522.

Q521. Es [Response to Q412] tu: (Elija Uno)

1 Compañero/a de trabajo

2 Jefe/a

3 OTRO [especifica]

8 Se rehúsa a contestar

If Q521 is equal to 3 then skip to Q522

If not, then skip to instruction before Q523.

Q522. Por favor especifica tu relación con [Response to Q412].

____

If Q514 is equal to 5 then skip to Q523

If not, then skip to instruction before Q524.

Q523. Es [Response to Q412] tu: (Elija Uno)

1 Padre/Madre/Padrastro/Madrastra

2 Hijo/a

3 Hermano/a o Medio-Hermano/a

4 Cuñado o cuñada

5 OTRO FAMILIAR [especifica]

8 Se rehúsa a contestar

If Q523 is equal to 5 then skip to Q524

If not, then skip to instruction before Q525.

Q524. Por favor especifica tu relación con [Response to Q412].

____

If Q514 is equal to 6 then skip to Q525

If not, then skip to instruction before Q526.

Q525. Es [Response to Q412] tu: (Elija Uno)

1 Camarero/a

2 Distribuidor de drogas

3 Alguien quien inyecta a otros por una tarifa o por drogas

4 Cliente de venta de drogas (alguien a quien le vendes droga)

5 Compañero/a para beber

6 Alguien con quien tú usas drogas

7 OTRO [especifica]

8 Se rehúsa a contestar

If Q525 is equal to 7 then skip to Q526

If not, then skip to instruction before Q527.

Q526. Por favor especifica tu relación con [Response to Q412].

____

If Q514 is equal to 7 then skip to Q527

If not, then skip to instruction before Q528.

Q527. Es [Response to Q412] tu: (Elija Uno)

1 Consejero religioso o espiritual

2 Maestro u otro tipo de educador

3 Profesional Medico

4 Profesional en salud mental

5 OTRO [especifica]

8 Se rehúsa a contestar

If Q527 is equal to 5 then skip to Q528

If not, then skip to instruction before Q529.

Q528. Por favor especifica tu relación con [Response to Q412].

____

If Q514 is not equal to 5 and Q514 is not equal to 8 then skip to Q529

If not, then skip to Q530.

Q529. ¿Tuviste sexo vaginal o anal con [Response to Q412] en los 6 meses anteriores?

1 Si

0 No

8 Se rehúsa a contestar

Q530. Es ¿[Response to Q410] hombre, mujer o transgénero? (Elija Uno)

1 Hombre

2 Mujer

3 Mujer transgénero

4 Hombre transgénero

5 No binario/género fluido/ agénero

6 Otro no en la lista

8 Se rehúsa a contestar

Q531. Por favor dime qué tan seguido te comunicas con [Response to Q412] (por teléfono, correo electrónico y/o por redes sociales, como Facebook) (Elija Uno)

01 Todos los días

02 Varias veces a la semana

03 Una vez a la semana

04 Una vez cada 2 semanas

05 Una vez al mes

06 Varias veces al año

07 Una vez al año

08 Menos de una vez al año

98 Se rehúsa a contestar

Q532. ¿Qué tan cercano/a te sientes a [Response to Q412]? (Elija Uno)

1	Muy cercano/a
2	Algo cercano/a
3	No tan cercano
4	No cercano
8	Se rehúsa a contestar

Q533. ¿Cuánto llevas de sentirte así de cercano/a con [Response to Q412]?

__ __	AñOS
__ __	MESES
__ __	DíAS
97	No Sabe (Años)
98	Se rehúsa a contestar (Años)

If Q533 is greater than Q513 and Q513 is not equal to 97 and Q513 is not equal to 98 then READ: "La respuesta es mayor a las veces que tú nos dijiste que conoces a &[NAME4]. Por favor revisa la respuesta." and skip to Q533.

If Q405 is greater than 4 and Q414 is not equal to "Se rehúsa a contestar" and Q414 is not equal to "Saltó" then skip to Q534

If not, then skip to instruction before Q556.

Q534. ¿Cuál es la edad de [Response to Q414]? __ __

97	No Sabe
98	Se rehúsa a contestar

Q535. ¿Cuánto hace que conoces a [Response to Q414]?

__ __	AñOS
__ __	MESES
__ __	DíAS
97	No Sabe (Años)
98	Se rehúsa a contestar (Años)

If Q535 is greater than Q534 then READ: "El participante dice que ha conocido a esta persona por un tiempo más largo que la edad de la persona. Lo estamos redireccionando para que se hagan las correcciones necesarias." and skip to Q534.

Q536. ¿Cuál es tu relación con [Response to Q414]? (Elija Uno)

1	Romántica o pareja sexual
2	Amigo/a
3	Vecino/a o compañero de casa
4	Compañero/a de trabajo o jefe/a
5	Familia - familiares de sangre o por matrimonio
6	Relacionado con drogas o alcohol
7	OTRO [especifica]
8	Se rehúsa a contestar

If Q536 is equal to 1 then skip to Q537

If not, then skip to instruction before Q538.

Q537. Es [Response to Q414] tu: (Elija Uno)

1	Esposo/a
2	Pareja romántica actual
3	Expareja romántica
4	Pareja sexual (no romántica)
5	OTRO [especifica]
8	Se rehúsa a contestar

If Q537 is equal to 5 then skip to Q538

If not, then skip to instruction before Q539.

Q538. Por favor especifica tu relación con [Response to Q414].

____

If Q536 is equal to 2 then skip to Q539

If not, then skip to instruction before Q540.

Q539. Es [Response to Q414] tu: (Elija Uno)

1 Mejor amigo/a

2 Amigo/a cercano/a

3 Amigo/a

4 Conocido/a

5 OTRO [especifica]

8 Se rehúsa a contestar

If Q539 is equal to 5 then skip to Q540

If not, then skip to instruction before Q541.

Q540. Por favor especifica tu relación con [Response to Q414].

____

If Q536 is equal to 3 then skip to Q541

If not, then skip to instruction before Q542.

Q541. Es [Response to Q414] tu: (Elija Uno)

1 Vecino/a

2 Compañero de cuarto o casa

3 OTRO [especifica]

8 Se rehúsa a contestar

If Q541 is equal to 3 then skip to Q542

If not, then skip to instruction before Q543.

Q542. Por favor especifica tu relación con [Response to Q414].

____

If Q536 is equal to 4 then skip to Q543

If not, then skip to instruction before Q544.

Q543. Es [Response to Q414] tu: (Elija Uno)

1	Compañero/a de trabajo
2	Jefe/a
3	OTRO [especifica]
8	Se rehúsa a contestar

If Q543 is equal to 3 then skip to Q544

If not, then skip to instruction before Q545.

Q544. Por favor especifica tu relación con [Response to Q414].

____

If Q536 is equal to 5 then skip to Q545

If not, then skip to instruction before Q546.

Q545. Es [Response to Q414] tu: (Elija Uno)

1	Padre/Madre/Padrastro/Madrastra
2	Hijo/a
3	Hermano/a o Medio-Hermano/a
4	Cuñado o cuñada
5	OTRO FAMILIAR [especifica]
8	Se rehúsa a contestar

If Q545 is equal to 5 then skip to Q546

If not, then skip to instruction before Q547.

Q546. Por favor especifica tu relación con [Response to Q414].

____

If Q536 is equal to 6 then skip to Q547

If not, then skip to instruction before Q548.

Q547. Es [Response to Q414] tu: (Elija Uno)

1 Camarero/a

2 Distribuidor de drogas

3 Alguien quien inyecta a otros por una tarifa o por drogas

4 Cliente de venta de drogas (alguien a quien le vendes droga)

5 Compañero/a para beber

6 Alguien con quien tú usas drogas

7 OTRO [especifica]

8 Se rehúsa a contestar

If Q547 is equal to 7 then skip to Q548

If not, then skip to instruction before Q549.

Q548. Por favor especifica tu relación con [Response to Q414].

____

If Q536 is equal to 7 then skip to Q549

If not, then skip to instruction before Q550.

Q549. Es [Response to Q414] tu: (Elija Uno)

1 Consejero religioso o espiritual

2 Maestro u otro tipo de educador

3 Profesional Medico

4 Profesional en salud mental

5 OTRO [especifica]

8 Se rehúsa a contestar

If Q549 is equal to 5 then skip to Q550

If not, then skip to instruction before Q551.

Q550. Por favor especifica tu relación con [Response to Q414].

____

If Q536 is not equal to 5 and Q536 is not equal to 8 then skip to Q551

If not, then skip to Q552.

Q551. ¿Tuviste sexo vaginal o anal con [Response to Q414] en los 6 meses anteriores?

1 Si

0 No

8 Se rehúsa a contestar

Q552. Es ¿[Response to Q414] hombre, mujer o transgénero? (Elija Uno)

1 Hombre

2 Mujer

3 Mujer transgénero

4 Hombre transgénero

5 No binario/género fluido/ agénero

6 Otro no en la lista

8 Se rehúsa a contestar

Q553. Por favor dime qué tan seguido te comunicas con [Response to Q414] (por teléfono, correo electrónico y/o por redes sociales, como Facebook) (Elija Uno)

01 Todos los días

02 Varias veces a la semana

03 Una vez a la semana

04 Una vez cada 2 semanas

05 Una vez al mes

06 Varias veces al año

07 Una vez al año

08 Menos de una vez al año

98 Se rehúsa a contestar

Q554. ¿Qué tan cercano/a te sientes a [Response to Q414]? (Elija Uno)

1	Muy cercano/a
2	Algo cercano/a
3	No tan cercano
4	No cercano
8	Se rehúsa a contestar

Q555. ¿Cuánto llevas de sentirte así de cercano/a con [Response to Q414]?

__ __	AñOS
__ __	MESES
__ __	DíAS
97	No Sabe (Años)
98	Se rehúsa a contestar (Años)

If Q555 is greater than Q535 and Q535 is not equal to 97 and Q535 is not equal to 98 then READ: "La respuesta es mayor a las veces que tú nos dijiste que conoces a &[NAME5]. Por favor revisa la respuesta." and skip to Q555.

If Q405 is greater than 1 and Q406 is not equal to "Se rehúsa a contestar" and Q406 is not equal to "Saltó" and Q408 is not equal to "Se rehúsa a contestar" and Q408 is not equal to "Saltó" then skip to instruction before Q556

If not, then skip to instruction before Q557.

READ: LEA: En el siguiente grupo de preguntas, te voy a dar dos nombres de los que nombraste anteriormente, y te voy a pedir que indiques qué tan frecuentemente estas dos personas se hablan entre si usando las categorías en esta tarjeta. Una vez que hayamos empezado, yo creo que podrás ver que esto funciona fácilmente.

Q556. ¿Qué tan seguido [Response to Q406] y [Response to Q408] se comunican entre ellos, ya sea platicando en persona, por mensaje de texto, o por chat? (Elija Uno)

01	Varias veces al día
02	Todos los días
03	Varias veces a la semana
04	Una vez a la semana
05	Una vez cada dos semanas
06	Una vez al Mes
07	Algunas veces al año
08	Una vez al año
09	Nunca
97	No Sabe
98	Se rehúsa a contestar

If Q405 is greater than 2 and Q406 is not equal to "Se rehúsa a contestar" and Q406 is not equal to "Saltó" and Q410 is not equal to "Se rehúsa a contestar" and Q410 is not equal to "Saltó" then skip to Q557

If not, then skip to instruction before Q558.

Q557. ¿Qué tan seguido [Response to Q406] y [Response to Q410] se comunican entre ellos, ya sea platicando en persona, por mensaje de texto, o por chat? (Elija Uno)

01	Varias veces al día
02	Todos los días
03	Varias veces a la semana
04	Una vez a la semana
05	Una vez cada dos semanas
06	Una vez al Mes
07	Algunas veces al año
08	Una vez al año
09	Nunca
97	No Sabe
98	Se rehúsa a contestar

If Q405 is greater than 3 and Q406 is not equal to "Se rehúsa a contestar" and Q406 is not equal to "Saltó" and Q412 is not equal to "Se rehúsa a contestar" and Q412 is not equal to "Saltó" then skip to Q558

If not, then skip to instruction before Q559.

Q558. ¿Qué tan seguido [Response to Q406] y [Response to Q412] se comunican entre ellos, ya sea platicando en persona, por mensaje de texto, o por chat? (Elija Uno)

01	Varias veces al día
02	Todos los días
03	Varias veces a la semana
04	Una vez a la semana
05	Una vez cada dos semanas
06	Una vez al Mes
07	Algunas veces al año
08	Una vez al año
09	Nunca
97	No Sabe
98	Se rehúsa a contestar

If Q405 is greater than 4 and Q406 is not equal to "Se rehúsa a contestar" and Q406 is not equal to "Saltó" and Q414 is not equal to "Se rehúsa a contestar" and Q414 is not equal to "Saltó" then skip to Q559

If not, then skip to instruction before Q560.

Q559. ¿Qué tan seguido [Response to Q406] y [Response to Q414] se comunican entre ellos, ya sea platicando en persona, por mensaje de texto, o por chat? (Elija Uno)

01	Varias veces al día
02	Todos los días
03	Varias veces a la semana
04	Una vez a la semana
05	Una vez cada dos semanas
06	Una vez al Mes
07	Algunas veces al año
08	Una vez al año
09	Nunca
97	No Sabe
98	Se rehúsa a contestar

If Q405 is greater than 2 and Q408 is not equal to "Se rehúsa a contestar" and Q408 is not equal to "Saltó" and Q410 is not equal to "Se rehúsa a contestar" and Q410 is not equal to "Saltó" then skip to Q560

If not, then skip to instruction before Q561.

Q560. ¿Qué tan seguido [Response to Q408] y [Response to Q410] se comunican entre ellos, ya sea platicando en persona, por mensaje de texto, o por chat? (Elija Uno)

01	Varias veces al día
02	Todos los días
03	Varias veces a la semana
04	Una vez a la semana
05	Una vez cada dos semanas
06	Una vez al Mes
07	Algunas veces al año
08	Una vez al año
09	Nunca
97	No Sabe
98	Se rehúsa a contestar

If Q405 is greater than 3 and Q408 is not equal to "Se rehúsa a contestar" and Q408 is not equal to "Saltó" and Q412 is not equal to "Se rehúsa a contestar" and Q412 is not equal to "Saltó" then skip to Q561

If not, then skip to instruction before Q562.

Q561. ¿Qué tan seguido [Response to Q408] y [Response to Q412] se comunican entre ellos, ya sea platicando en persona, por mensaje de texto, o por chat? (Elija Uno)

01	Varias veces al día
02	Todos los días
03	Varias veces a la semana
04	Una vez a la semana
05	Una vez cada dos semanas
06	Una vez al Mes
07	Algunas veces al año
08	Una vez al año
09	Nunca
97	No Sabe
98	Se rehúsa a contestar

If Q405 is greater than 4 and Q408 is not equal to "Se rehúsa a contestar" and Q408 is not equal to "Saltó" and Q414 is not equal to "Se rehúsa a contestar" and Q414 is not equal to "Saltó" then skip to Q562

If not, then skip to instruction before Q563.

Q562. ¿Qué tan seguido [Response to Q408] y [Response to Q414] se comunican entre ellos, ya sea platicando en persona, por mensaje de texto, o por chat? (Elija Uno)

01	Varias veces al día
02	Todos los días
03	Varias veces a la semana
04	Una vez a la semana
05	Una vez cada dos semanas
06	Una vez al Mes
07	Algunas veces al año
08	Una vez al año
09	Nunca
97	No Sabe
98	Se rehúsa a contestar

If Q405 is greater than 3 and Q410 is not equal to "Se rehúsa a contestar" and Q410 is not equal to "Saltó" and Q412 is not equal to "Se rehúsa a contestar" and Q412 is not equal to "Saltó" then skip to Q563

If not, then skip to instruction before Q564.

Q563. ¿Qué tan seguido [Response to Q410] y [Response to Q412] se comunican entre ellos, ya sea platicando en persona, por mensaje de texto, o por chat? (Elija Uno)

01	Varias veces al día
02	Todos los días
03	Varias veces a la semana
04	Una vez a la semana
05	Una vez cada dos semanas
06	Una vez al Mes
07	Algunas veces al año
08	Una vez al año
09	Nunca
97	No Sabe
98	Se rehúsa a contestar

If Q405 is greater than 4 and Q410 is not equal to "Se rehúsa a contestar" and Q410 is not equal to "Saltó" and Q414 is not equal to "Se rehúsa a contestar" and Q414 is not equal to "Saltó" then skip to Q564

If not, then skip to instruction before Q565.

Q564. ¿Qué tan seguido [Response to Q410] y [Response to Q414] se comunican entre ellos, ya sea platicando en persona, por mensaje de texto, o por chat? (Elija Uno)

01	Varias veces al día
02	Todos los días
03	Varias veces a la semana
04	Una vez a la semana
05	Una vez cada dos semanas
06	Una vez al Mes
07	Algunas veces al año
08	Una vez al año
09	Nunca
97	No Sabe
98	Se rehúsa a contestar

If Q405 is greater than 4 and Q412 is not equal to "Se rehúsa a contestar" and Q412 is not equal to "Saltó" and Q414 is not equal to "Se rehúsa a contestar" and Q414 is not equal to "Saltó" then skip to Q565

If not, then skip to instruction before Q566.

Q565. ¿Qué tan seguido [Response to Q412] y [Response to Q414] se comunican entre ellos, ya sea platicando en persona, por mensaje de texto, o por chat? (Elija Uno)

01	Varias veces al día
02	Todos los días
03	Varias veces a la semana
04	Una vez a la semana
05	Una vez cada dos semanas
06	Una vez al Mes
07	Algunas veces al año
08	Una vez al año
09	Nunca
97	No Sabe
98	Se rehúsa a contestar

If Q405 is greater than 0 and Q406 is not equal to "Se rehúsa a contestar" and Q406 is not equal to "Saltó" then skip to Q566

If not, then skip to instruction before Q590.

Q566. ¿Qué tan seguido usa drogas [Response to Q406]? (Elija Uno)

01	Todos los días
02	Varias veces a la semana
03	Una vez a la semana
04	Una vez cada dos semanas
05	Una vez al mes
06	Algunas veces al año
07	Una vez al año
08	Menos de una vez al año
09	Nunca
10	Usaba drogas anteriormente pero no en el presente
97	No Sabe
98	Se rehúsa a contestar

If Q566 is not less than 1 and Q566 is not greater than 8 then skip to Q567

If not, then skip to instruction before Q568.

Q567. ¿Cuál de las siguientes drogas usa [Response to Q406]? (Marque Todas las que Correspondan)

__ Alcohol

__ Mariguana

__ Heroína sola

__ Cocaína crack sola

__ Cocaína en polvo sola

__ Heroína y cocaína juntas (speedball)

__ Metanfetaminas/cristal solo

__ Metanfetaminas/cristal y heroína juntas

__ China White sola

__ China White y heroína juntas

__ China White y metanfetaminas/cristal juntas

__ PCP

__ Alucinógenos como acido, LSD, peyote, mescalina, hongos

__ Inhalantes (por ejemplo, pegamento, gasolina, diluyente de pintura, agua celeste u otros químicos)

__ Tranquilizantes sin prescripción (Rivotril, Diazepam, Valium, Ativan o Restoril)

__ Oxycontin sin prescripción

__ Otros opioides de prescripción sin prescripción (Vicodin, Darvon, Percocet)

__ Barbitúricos sin prescripción (Amytal, Nembutal y Seconal)

__ Otro tipo de droga o combinación que no ha sido mencionada

__ No Sabe

__ Se rehúsa a contestar

If Q567S is equal to 1 then skip to Q568

If not, then skip to instruction before Q569.

Q568. Otra droga o combinación de drogas [especifica]

____

If Q566 is not less than 1 and Q566 is not greater than 8 then skip to Q569

If not, then skip to instruction before Q570.

Q569. Usa ¿[Response to Q406] drogas inyectadas, o no inyectadas, o a veces las usa de una manera y a veces de la otra? (Elija Uno)

1	Solo usa drogas inyectadas
2	Solo usa drogas no inyectadas
3	A veces usa drogas inyectables
7	No Sabe
8	Se rehúsa a contestar

If Q569 is equal to 1 or Q569 is equal to 3 then skip to Q570

If not, then skip to instruction before Q571.

Q570. ¿Alguna vez has usado la jeringa, agua, cacharro o algodón que ya había sido usada por [Response to Q406]?

1	Si
0	No
8	Se rehúsa a contestar

If Q566 is not equal to 9 and Q566 is not equal to 97 and Q566 is not equal to 98 then skip to Q571

If not, then skip to instruction before Q572.

Q571. ¿Alguna vez te ha ofrecido o animado a usar drogas [Response to Q406]?

1	Si
0	No
8	Se rehúsa a contestar

If Q571 is equal to 1 then skip to Q572

If not, then skip to instruction before Q573.

Q572. ¿Cuál de las siguientes drogas te ha ofrecido o animado a usar [Response to Q406]? (Marque Todas las que Correspondan)

__ Alcohol

__ Mariguana

__ Heroína sola

__ Cocaína crack sola

__ Cocaína en polvo sola

__ Heroína y cocaína juntas (speedball)

__ Metanfetaminas/cristal solo

__ Metanfetaminas/cristal y heroína juntas

__ China White sola

__ China White y heroína juntas

__ China White y metanfetaminas/cristal juntas

__ PCP

__ Alucinógenos como acido, LSD, peyote, mescalina, hongos

__ Inhalantes (por ejemplo, pegamento, gasolina, diluyente de pintura, agua celeste u otros químicos)

__ Tranquilizantes sin prescripción (Rivotril, Diazepam, Valium, Ativan o Restoril)

__ Oxycontin sin prescripción

__ Otros opioides de prescripción sin prescripción (Vicodin, Darvon, Percocet)

__ Barbitúricos sin prescripción (Amytal, Nembutal y Seconal)

__ Otro tipo de droga o combinación que no ha sido mencionada

__ Se rehúsa a contestar

If Q572S is equal to 1 then skip to Q573

If not, then skip to Q574.

Q573. Otra droga o combinación de drogas [especifica]

____

Q574. ¿Alguna vez [Response to Q406] te ha animado a dejar de usar drogas?

1 Si

0 No

8 Se rehúsa a contestar

If Q574 is equal to 1 then skip to Q575

If not, then skip to instruction before Q576.

Q575. ¿Alguna vez [Response to Q406] te ha animado a dejar de usar drogas inyectables específicamente?

1 Si

0 No

7 No Sabe

8 Se rehúsa a contestar

If Q566 is not equal to 9 and Q566 is not equal to 97 and Q566 is not equal to 98 then skip to Q576

If not, then skip to instruction before Q577.

Q576. ¿Qué edad tenía [Response to Q406] cuando empezó a usar drogas por primera vez?

__ __

97 No Sabe

98 Se rehúsa a contestar

If Q566 is not equal to 9 and Q566 is not equal to 10 and Q566 is not equal to 97 and Q566 is not equal to 98 then skip to Q577

If not, then skip to Q578.

Q577. Considera el uso diario de drogas de [Response to Q406], ¿Alguna vez en su vida ha usado [Response to Q406] el doble de la dosis de droga o la ha mezclado con otra droga?

1 Si

0 No

7 No Sabe

8 Se rehúsa a contestar

Q578. ¿Alguna vez en su vida [Response to Q406] ha vivido en la calle, en un albergue, en un hotel, temporalmente con amigos/familiares, en un carro, o en una casa deshabitada?

1 Si

0 No

7 No Sabe

8 Se rehúsa a contestar

Q579. ¿Dónde vive [Response to Q406] actualmente? (Elija Uno)

1 Condado de San Diego

2 Tijuana

3 Otro (especifica)

7 No Sabe

8 Se rehúsa a contestar

If Q579 is equal to 3 then skip to Q580

If not, then skip to instruction before Q581.

Q580. Por favor especifica donde vive [Response to Q406].

———————————————————————————————

If Q579 is equal to 1 and Q566 is not equal to 9 and Q566 is not equal to 97 and Q566 is not equal to 98 then skip to Q581

If not, then skip to instruction before Q582.

Q581. **En los últimos 6 meses**, ¿ha viajado [Response to Q406] a México para comprar o conseguir drogas?

1	Si
0	No
7	No Sabe
8	Se rehúsa a contestar

If Q581 is equal to 1 then skip to Q582

If not, then skip to instruction before Q583.

Q582. ¿Cuál de los siguientes tipos de drogas compró [Response to Q406] en México? (Marque Todas las que Correspondan)

__ Mariguana

__ Heroina

__ Cocaína crack sola

__ Cocaína en polvo sola

__ Heroína y cocaína juntas (speedball)

__ Metanfetaminas/cristal solo

__ Metanfetaminas/cristal y heroína juntas

__ China White sola

__ China White y heroína juntas

__ China White y metanfetaminas/cristal juntas

__ PCP

__ Alucinógenos como acido, LSD, peyote, mescalina, hongos

__ Inhalantes (por ejemplo, pegamento, gasolina, diluyente de pintura, agua celeste u otros químicos)

__ Tranquilizantes sin prescripción (Rivotril, Diazepam, Valium, Ativan o Restoril)

__ Oxycontin sin prescripción

__ Otros opioides de prescripción sin prescripción (Vicodin, Darvon, Percocet)

__ Barbituricos sin prescripción (Amytal, Nembutal y Seconal)

__ Otro tipo de droga o combinación que no ha sido mencionada

__ No Sabe

__ Se rehúsa a contestar

If Q582R is equal to 1 then skip to Q583

If not, then skip to instruction before Q584.

Q583. Por favor especifica qué tipo de drogas compró [Response to Q406] en México.

____

If Q579 is equal to 1 then skip to Q584

If not, then skip to instruction before Q585.

Q584. **En los últimos 6 meses**, ¿ha viajado [Response to Q406] a México para pagar por sexo con dinero o drogas?

1 Si

0 No

7 No Sabe

8 Se rehúsa a contestar

If Q579 is equal to 2 and Q566 is not equal to 9 and Q566 is not equal to 97 and Q566 is not equal to 98 then skip to Q585

If not, then skip to instruction before Q586.

Q585. **En los últimos 6 meses**, ¿ha viajado [Response to Q406] a Estados Unidos para comprar o conseguir drogas?

1 Si

0 No

7 No Sabe

8 Se rehúsa a contestar

If Q585 is equal to 1 then skip to Q586

If not, then skip to instruction before Q587.

Q586. ¿Cuál de los siguientes tipos de drogas compró [Response to Q406] en Estados Unidos? (Marque Todas las que Correspondan)

__ Mariguana

__ Heroína

__ Cocaína crack sola

__ Cocaína en polvo sola

__ Heroína y cocaína juntas (speedball)

__ Metanfetaminas/cristal solo

__ Metanfetaminas/cristal y heroína juntas

__ China White sola

__ China White y heroína juntas

__ China White y metanfetaminas/cristal juntas

__ PCP

__ Alucinógenos como acido, LSD, peyote, mescalina, hongos

__ Inhalantes (por ejemplo, pegamento, gasolina, diluyente de pintura, agua celeste u otros químicos)

__ Tranquilizantes sin prescripción (Rivotril, Diazepam, Valium, Ativan o Restoril)

__ Oxycontin sin prescripción

__ Otros opioides de prescripción sin prescripción (Vicodin, Darvon, Percocet)

__ Barbituricos sin prescripción (Amytal, Nembutal y Seconal)

__ Otro tipo de droga o combinación que no ha sido mencionada

__ No Sabe

__ Se rehúsa a contestar

If Q586R is equal to 1 then skip to Q587

If not, then skip to instruction before Q588.

Q587. Por favor especifica qué tipo de drogas compró [Response to Q406] en Estados Unidos.

____

If Q579 is equal to 2 then skip to Q588

If not, then skip to Q589.

Q588. **En los últimos 6 meses**, ¿[Response to Q406] ha tenido sexo por dinero o drogas con alguien de los Estados Unidos?

1 Si

0 No

7 No Sabe

8 Se rehúsa a contestar

Q589. ¿Alguna vez [Response to Q406] ha sido detenido o arrestado?

1 Si

0 No

7 No Sabe

8 Se rehúsa a contestar

If Q405 is greater than 1 and Q408 is not equal to "Se rehúsa a contestar" and Q408 is not equal to "Saltó" then skip to Q590

If not, then skip to instruction before Q614.

Q590. ¿Qué tan seguido usa drogas [Response to Q408]? (Elija Uno)

01	Todos los días
02	Varias veces a la semana
03	Una vez a la semana
04	Una vez cada dos semanas
05	Una vez al mes
06	Algunas veces al año
07	Una vez al año
08	Menos de una vez al año
09	Nunca
10	Usaba drogas anteriormente pero no en el presente
97	No Sabe
98	Se rehúsa a contestar

If Q590 is not less than 1 and Q590 is not greater than 8 then skip to Q591

If not, then skip to instruction before Q592.

Q591. ¿Cuál de las siguientes drogas usa [Response to Q408]? (Marque Todas las que Correspondan)

__ Alcohol

__ Mariguana

__ Heroína sola

__ Cocaína crack sola

__ Cocaína en polvo sola

__ Heroína y cocaína juntas (speedball)

__ Metanfetaminas/cristal solo

__ Metanfetaminas/cristal y heroína juntas

__ China White sola

__ China White y heroína juntas

__ China White y metanfetaminas/cristal juntas

__ PCP

__ Alucinógenos como acido, LSD, peyote, mescalina, hongos

__ Inhalantes (por ejemplo, pegamento, gasolina, diluyente de pintura, agua celeste u otros químicos)

__ Tranquilizantes sin prescripción (Rivotril, Diazepam, Valium, Ativan o Restoril)

__ Oxycontin sin prescripción

__ Otros opioides de prescripción sin prescripción (Vicodin, Darvon, Percocet)

__ Barbituricos sin prescripción (Amytal, Nembutal y Seconal)

__ Otro tipo de droga o combinación que no ha sido mencionada

__ No Sabe

__ Se rehúsa a contestar

If Q591S is equal to 1 then skip to Q592

If not, then skip to instruction before Q593.

Q592. Otra droga o combinación de drogas [especifica]

____

If Q590 is not less than 1 and Q590 is not greater than 8 then skip to Q593

If not, then skip to instruction before Q594.

Q593. Usa ¿[Response to Q408] drogas inyectadas, o no inyectadas, o a veces las usa de una manera y a veces de la otra? (Elija Uno)

1 Solo usa drogas inyectadas

2 Solo usa drogas no inyectadas

3 A veces usa drogas inyectables

7 No Sabe

8 Se rehúsa a contestar

If Q593 is equal to 1 or Q593 is equal to 3 then skip to Q594

If not, then skip to instruction before Q595.

Q594. ¿Alguna vez has usado la jeringa, agua, cacharro o algodón que ya había sido usada por [Response to Q408]?

1 Si

0 No

8 Se rehúsa a contestar

If Q590 is not equal to 9 and Q590 is not equal to 97 and Q590 is not equal to 98 then skip to Q595

If not, then skip to instruction before Q596.

Q595. ¿Alguna vez te ha ofrecido o animado a usar drogas [Response to Q408]?

1	Si
0	No
8	Se rehúsa a contestar

If Q595 is equal to 1 then skip to Q596

If not, then skip to instruction before Q597.

Q596. ¿Cuál de las siguientes drogas te ha ofrecido o animado a usar [Response to Q408]? (Marque Todas las que Correspondan)

__ Alcohol

__ Mariguana

__ Heroína sola

__ Cocaína crack sola

__ Cocaína en polvo sola

__ Heroína y cocaína juntas (speedball)

__ Metanfetaminas/cristal solo

__ Metanfetaminas/cristal y heroína juntas

__ China White sola

__ China White y heroína juntas

__ China White y metanfetaminas/cristal juntas

__ PCP

__ Alucinógenos como acido, LSD, peyote, mescalina, hongos

__ Inhalantes (por ejemplo, pegamento, gasolina, diluyente de pintura, agua celeste u otros químicos)

__ Tranquilizantes sin prescripción (Rivotril, Diazepam, Valium, Ativan o Restoril)

__ Oxycontin sin prescripción

__ Otros opioides de prescripción sin prescripción (Vicodin, Darvon, Percocet)

__ Barbituricos sin prescripción (Amytal, Nembutal y Seconal)

__ Otro tipo de droga o combinación que no ha sido mencionada

__ Se rehúsa a contestar

If Q596S is equal to 1 then skip to Q597

If not, then skip to Q598.

Q597. Otra droga o combinación de drogas [especifica]

____

Q598. ¿Alguna vez [Response to Q408] te ha animado a dejar de usar drogas?

1 Si

0 No

8 Se rehúsa a contestar

If Q598 is equal to 1 then skip to Q599

If not, then skip to instruction before Q600.

Q599. ¿Alguna vez [Response to Q408] te ha animado a dejar de usar drogas inyectables específicamente?

1 Si

0 No

7 No Sabe

8 Se rehúsa a contestar

If Q590 is not equal to 9 and Q590 is not equal to 97 and Q590 is not equal to 98 then skip to Q600

If not, then skip to instruction before Q601.

Q600. ¿Qué edad tenía [Response to Q408] cuando empezó a usar drogas por primera vez?

__ __

97 No Sabe

98 Se rehúsa a contestar

If Q590 is not equal to 9 and Q590 is not equal to 10 and Q590 is not equal to 97 and Q590 is not equal to 98 then skip to Q601

If not, then skip to Q602.

Q601. Considera el uso diario de drogas de [Response to Q408], ¿Alguna vez en su vida ha usado [Response to Q408] el doble de la dosis de droga o la ha mezclado con otra droga?

1 Si

0 No

7 No Sabe

8 Se rehúsa a contestar

Q602. ¿Alguna vez [Response to Q408] ha vivido en la calle, en un albergue, en un hotel, temporalmente con amigos/familiares, en un carro, o en una casa deshabitada?

1 Si

0 No

7 No Sabe

8 Se rehúsa a contestar

Q603. ¿Dónde vive [Response to Q408] actualmente? (Elija Uno)

1 Condado de San Diego

2 Tijuana

3 Otro (especifica)

7 No Sabe

8 Se rehúsa a contestar

If Q603 is equal to 3 then skip to Q604

If not, then skip to instruction before Q605.

Q604. Por favor especifica donde vive [Response to Q408].

____

If Q603 is equal to 1 and Q590 is not equal to 9 and Q590 is not equal to 97 and Q590 is not equal to 98 then skip to Q605

If not, then skip to instruction before Q606.

Q605. **En los últimos 6 meses**, ¿ha viajado [Response to Q408] a México para comprar u conseguir drogas?

1 Si

0 No

7 No Sabe

8 Se rehúsa a contestar

If Q605 is equal to 1 then skip to Q606

If not, then skip to instruction before Q607.

Q606. ¿Cuál de los siguientes tipos de drogas compró [Response to Q408] en México? (Marque Todas las que Correspondan)

__ Mariguana

__ Heroína

__ Cocaína crack sola

__ Cocaína en polvo sola

__ Heroína y cocaína juntas (speedball)

__ Metanfetaminas/cristal solo

__ Metanfetaminas/cristal y heroína juntas

__ China White sola

__ China White y heroína juntas

__ China White y metanfetaminas/cristal juntas

__ PCP

__ Alucinógenos como acido, LSD, peyote, mescalina, hongos

__ Inhalantes (por ejemplo, pegamento, gasolina, diluyente de pintura, agua celeste u otros químicos)

__ Tranquilizantes sin prescripción (Rivotril, Diazepam, Valium, Ativan o Restoril)

__ Oxycontin sin prescripción

__ Otros opioides de prescripción sin prescripción (Vicodin, Darvon, Percocet)

__ Barbituricos sin prescripción (Amytal, Nembutal y Seconal)

__ Otro tipo de droga o combinación que no ha sido mencionada

__ No Sabe

__ Se rehúsa a contestar

If Q606R is equal to 1 then skip to Q607

If not, then skip to instruction before Q608.

Q607. Por favor especifica qué tipo de drogas compró [Response to Q408] en México.

____

If Q603 is equal to 1 then skip to Q608

If not, then skip to instruction before Q609.

Q608. **En los últimos 6 meses**, ¿ha viajado [Response to Q408] a México para pagar por sexo con dinero o drogas?

1 Si

0 No

7 No Sabe

8 Se rehúsa a contestar

If Q603 is equal to 2 and Q590 is not equal to 9 and Q590 is not equal to 97 and Q590 is not equal to 98 then skip to Q609

If not, then skip to instruction before Q610.

Q609. **En los últimos 6 meses**, ¿ha viajado [Response to Q408] a Estados Unidos para comprar u conseguir drogas?

1 Si

0 No

7 No Sabe

8 Se rehúsa a contestar

If Q609 is equal to 1 then skip to Q610

If not, then skip to instruction before Q611.

Q610. ¿Cuál de los siguientes tipos de drogas compró [Response to Q408] en Estados Unidos? (Marque Todas las que Correspondan)

__ Mariguana

__ Heroína

__ Cocaína crack sola

__ Cocaína en polvo sola

__ Heroína y cocaína juntas (speedball)

__ Metanfetaminas/cristal solo

__ Metanfetaminas/cristal y heroína juntas

__ China White sola

__ China White y heroína juntas

__ China White y metanfetaminas/cristal juntas

__ PCP

__ Alucinógenos como acido, LSD, peyote, mescalina, hongos

__ Inhalantes (por ejemplo, pegamento, gasolina, diluyente de pintura, agua celeste u otros químicos)

__ Tranquilizantes sin prescripción (Rivotril, Diazepam, Valium, Ativan o Restoril)

__ Oxycontin sin prescripción

__ Otros opioides de prescripción sin prescripción (Vicodin, Darvon, Percocet)

__ Barbituricos sin prescripción (Amytal, Nembutal y Seconal)

__ Otro tipo de droga o combinación que no ha sido mencionada

__ No Sabe

__ Se rehúsa a contestar

If Q610R is equal to 1 then skip to Q611

If not, then skip to instruction before Q612.

Q611. Por favor especifica qué tipo de drogas compró [Response to Q408] en Estados Unidos.

____

If Q603 is equal to 2 then skip to Q612

If not, then skip to Q613.

Q612. **En los últimos 6 meses**, ¿[Response to Q408] ha tenido sexo por dinero o drogas con alguien de los Estados Unidos?

1 Si

0 No

7 No Sabe

8 Se rehúsa a contestar

Q613. ¿Alguna vez [Response to Q408] ha sido detenido o arrestado?

1 Si

0 No

7 No Sabe

8 Se rehúsa a contestar

If Q405 is greater than 2 and Q410 is not equal to "Se rehúsa a contestar" and Q410 is not equal to "Saltó" then skip to Q614

If not, then skip to instruction before Q638.

Q614. ¿Qué tan seguido usa drogas [Response to Q410]? (Elija Uno)

01	Todos los días
02	Varias veces a la semana
03	Una vez a la semana
04	Una vez cada dos semanas
05	Una vez al mes
06	Algunas veces al año
07	Una vez al año
08	Menos de una vez al año
09	Nunca
10	Usaba drogas anteriormente pero no en el presente
97	No Sabe
98	Se rehúsa a contestar

If Q614 is not less than 1 and Q614 is not greater than 8 then skip to Q615

If not, then skip to instruction before Q616.

Q615. ¿Cuál de las siguientes drogas usa [Response to Q410]? (Marque Todas las que Correspondan)

__ Alcohol

__ Mariguana

__ Heroína sola

__ Cocaína crack sola

__ Cocaína en polvo sola

__ Heroína y cocaína juntas (speedball)

__ Metanfetaminas/cristal solo

__ Metanfetaminas/cristal y heroína juntas

__ China White sola

__ China White y heroína juntas

__ China White y metanfetaminas/cristal juntas

__ PCP

__ Alucinógenos como acido, LSD, peyote, mescalina, hongos

__ Inhalantes (por ejemplo, pegamento, gasolina, diluyente de pintura, agua celeste u otros químicos)

__ Tranquilizantes sin prescripción (Rivotril, Diazepam, Valium, Ativan o Restoril)

__ Oxycontin sin prescripción

__ Otros opioides de prescripción sin prescripción (Vicodin, Darvon, Percocet)

__ Barbituricos sin prescripción (Amytal, Nembutal y Seconal)

__ Otro tipo de droga o combinación que no ha sido mencionada

__ No Sabe

__ Se rehúsa a contestar

If Q615S is equal to 1 then skip to Q616

If not, then skip to instruction before Q617.

Q616. Otra droga o combinación de drogas [especifica]

____

If Q614 is not less than 1 and Q614 is not greater than 8 then skip to Q617

If not, then skip to instruction before Q618.

Q617. Usa ¿[Response to Q410] drogas inyectadas, o no inyectadas, o a veces las usa de una manera y a veces de la otra? (Elija Uno)

1	Solo usa drogas inyectadas
2	Solo usa drogas no inyectadas
3	A veces usa drogas inyectables
7	No Sabe
8	Se rehúsa a contestar

If Q617 is equal to 1 or Q617 is equal to 3 then skip to Q618

If not, then skip to instruction before Q619.

Q618. ¿Alguna vez has usado la jeringa, agua, cacharro o algodón que ya había sido usada por [Response to Q410]?

1	Si
0	No
8	Se rehúsa a contestar

If Q614 is not equal to 9 and Q614 is not equal to 97 and Q614 is not equal to 98 then skip to Q619

If not, then skip to instruction before Q620.

Q619. ¿Alguna vez te ha ofrecido o animado a usar drogas [Response to Q410]?

1 Si

0 No

8 Se rehúsa a contestar

If Q619 is equal to 1 then skip to Q620

If not, then skip to instruction before Q621.

Q620. ¿Cuál de las siguientes drogas te ha ofrecido o animado a usar [Response to Q410]? (Marque Todas las que Correspondan)

__ Alcohol

__ Mariguana

__ Heroína sola

__ Cocaína crack sola

__ Cocaína en polvo sola

__ Heroína y cocaína juntas (speedball)

__ Metanfetaminas/cristal solo

__ Metanfetaminas/cristal y heroína juntas

__ China White sola

__ China White y heroína juntas

__ China White y metanfetaminas/cristal juntas

__ PCP

__ Alucinógenos como acido, LSD, peyote, mescalina, hongos

__ Inhalantes (por ejemplo, pegamento, gasolina, diluyente de pintura, agua celeste u otros químicos)

__ Tranquilizantes sin prescripción (Rivotril, Diazepam, Valium, Ativan o Restoril)

__ Oxycontin sin prescripción

__ Otros opioides de prescripción sin prescripción (Vicodin, Darvon, Percocet)

__ Barbituricos sin prescripción (Amytal, Nembutal y Seconal)

__ Otro tipo de droga o combinación que no ha sido mencionada

__ Se rehúsa a contestar

If Q620S is equal to 1 then skip to Q621

If not, then skip to Q622.

Q621. Otra droga o combinación de drogas [especifica]

____

Q622. ¿Alguna vez [Response to Q410] te ha animado a dejar de usar drogas?

1	Si
0	No
8	Se rehúsa a contestar

If Q622 is equal to 1 then skip to Q623

If not, then skip to instruction before Q624.

Q623. ¿Alguna vez [Response to Q410] te ha animado a dejar de usar drogas inyectables específicamente?

1	Si
0	No
7	No Sabe
8	Se rehúsa a contestar

If Q614 is not equal to 9 and Q614 is not equal to 97 and Q614 is not equal to 98 then skip to Q624

If not, then skip to instruction before Q625.

Q624. ¿Qué edad tenía [Response to Q410] cuando empezó a usar drogas por primera vez?

__ __

97	No Sabe
98	Se rehúsa a contestar

If Q614 is not equal to 9 and Q614 is not equal to 10 and Q614 is not equal to 97 and Q614 is not equal to 98 then skip to Q625

If not, then skip to Q626.

Q625. Considera el uso diario de drogas de [Response to Q410], ¿Alguna vez en su vida ha usado [Response to Q410] el doble de la dosis de droga o la ha mezclado con otra droga?

1 Si

0 No

7 No Sabe

8 Se rehúsa a contestar

Q626. ¿Alguna vez [Response to Q410] ha vivido en la calle, en un albergue, en un hotel, temporalmente con amigos/familiares, en un carro, o en una casa deshabitada?

1 Si

0 No

7 No Sabe

8 Se rehúsa a contestar

Q627. ¿Dónde vive [Response to Q410] actualmente? (Elija Uno)

1 Condado de San Diego

2 Tijuana

3 Otro (especifica)

7 No Sabe

8 Se rehúsa a contestar

If Q627 is equal to 3 then skip to Q628

If not, then skip to instruction before Q629.

Q628. Por favor especifica dónde vive [Response to Q410].

____

If Q627 is equal to 1 and Q614 is not equal to 9 and Q614 is not equal to 97 and Q614 is not equal to 98 then skip to Q629

If not, then skip to instruction before Q630.

Q629. **En los últimos 6 meses**, ¿ha viajado [Response to Q410] a México para comprar u conseguir drogas?

1	Si
0	No
7	No Sabe
8	Se rehúsa a contestar

If Q629 is equal to 1 then skip to Q630

If not, then skip to instruction before Q631.

Q630. ¿Cuál de los siguientes tipos de drogas compró [Response to Q410] en México? (Marque Todas las que Correspondan)

__ Mariguana

__ Heroina

__ Cocaína crack sola

__ Cocaína en polvo sola

__ Heroína y cocaína juntas (speedball)

__ Metanfetaminas/cristal solo

__ Metanfetaminas/cristal y heroína juntas

__ China White sola

__ China White y heroína juntas

__ China White y metanfetaminas/cristal juntas

__ PCP

__ Alucinógenos como acido, LSD, peyote, mescalina, hongos

__ Inhalantes (por ejemplo, pegamento, gasolina, diluyente de pintura, agua celeste u otros químicos)

__ Tranquilizantes sin prescripción (Rivotril, Diazepam, Valium, Ativan o Restoril)

__ Oxycontin sin prescripción

__ Otros opioides de prescripción sin prescripción (Vicodin, Darvon, Percocet)

__ Barbituricos sin prescripción (Amytal, Nembutal y Seconal)

__ Otro tipo de droga o combinación que no ha sido mencionada

__ No Sabe

__ Se rehúsa a contestar

If Q630R is equal to 1 then skip to Q631

If not, then skip to instruction before Q632.

Q631. Por favor especifica qué tipo de drogas compró [Response to Q410] en México.

____

If Q627 is equal to 1 then skip to Q632

If not, then skip to instruction before Q633.

Q632. **En los últimos 6 meses**, ¿ha viajado [Response to Q410] a México para pagar con dinero o drogas por sexo?

1 Si

0 No

7 No Sabe

8 Se rehúsa a contestar

If Q627 is equal to 2 and Q614 is not equal to 9 and Q614 is not equal to 97 and Q614 is not equal to 98 then skip to Q633

If not, then skip to instruction before Q634.

Q633. **En los últimos 6 meses**, ¿ha viajado [Response to Q410] a Estados Unidos para comprar u conseguir drogas?

1 Si

0 No

7 No Sabe

8 Se rehúsa a contestar

If Q633 is equal to 1 then skip to Q634

If not, then skip to instruction before Q635.

Q634. ¿Cuál de los siguientes tipos de drogas compró [Response to Q410] en Estados Unidos? (Marque Todas las que Correspondan)

__ Mariguana

__ Heroína

__ Cocaína crack sola

__ Cocaína en polvo sola

__ Heroína y cocaína juntas (speedball)

__ Metanfetaminas/cristal solo

__ Metanfetaminas/cristal y heroína juntas

__ China White sola

__ China White y heroína juntas

__ China White y metanfetaminas/cristal juntas

__ PCP

__ Alucinógenos como acido, LSD, peyote, mescalina, hongos

__ Inhalantes (por ejemplo, pegamento, gasolina, diluyente de pintura, agua celeste u otros químicos)

__ Tranquilizantes sin prescripción (Rivotril, Diazepam, Valium, Ativan o Restoril)

__ Oxycontin sin prescripción

__ Otros opioides de prescripción sin prescripción (Vicodin, Darvon, Percocet)

__ Barbituricos sin prescripción (Amytal, Nembutal y Seconal)

__ Otro tipo de droga o combinación que no ha sido mencionada

__ No Sabe

__ Se rehúsa a contestar

If Q634R is equal to 1 then skip to Q635

If not, then skip to instruction before Q636.

Q635. Por favor especifica qué tipo de drogas compró [Response to Q410] en Estados Unidos.

____

If Q627 is equal to 2 then skip to Q636

If not, then skip to Q637.

Q636. **En los últimos 6 meses**, ¿[Response to Q410] ha tenido sexo por dinero o drogas con alguien de los Estados Unidos?

1	Si
0	No
7	No Sabe
8	Se rehúsa a contestar

Q637. ¿Alguna vez [Response to Q410] ha sido detenido o arrestado?

1	Si
0	No
7	No Sabe
8	Se rehúsa a contestar

If Q405 is greater than 3 and Q412 is not equal to "Se rehúsa a contestar" and Q412 is not equal to "Saltó" then skip to Q638

If not, then skip to instruction before Q662.

Q638. ¿Qué tan seguido usa drogas [Response to Q412]? (Elija Uno)

01	Todos los días
02	Varias veces a la semana
03	Una vez a la semana
04	Una vez cada dos semanas
05	Una vez al mes
06	Algunas veces al año
07	Una vez al año
08	Menos de una vez al año
09	Nunca
10	Usaba drogas anteriormente pero no en el presente
97	No Sabe
98	Se rehúsa a contestar

If Q638 is not less than 1 and Q638 is not greater than 8 then skip to Q639

If not, then skip to instruction before Q640.

Q639. ¿Cuál de las siguientes drogas usa [Response to Q412]? (Marque Todas las que Correspondan)

__ Alcohol

__ Mariguana

__ Heroína sola

__ Cocaína crack sola

__ Cocaína en polvo sola

__ Heroína y cocaína juntas (speedball)

__ Metanfetaminas/cristal solo

__ Metanfetaminas/cristal y heroína juntas

__ China White sola

__ China White y heroína juntas

__ China White y metanfetaminas/cristal juntas

__ PCP

__ Alucinógenos como acido, LSD, peyote, mescalina, hongos

__ Inhalantes (por ejemplo, pegamento, gasolina, diluyente de pintura, agua celeste u otros químicos)

__ Tranquilizantes sin prescripción (Rivotril, Diazepam, Valium, Ativan o Restoril)

__ Oxycontin sin prescripción

__ Otros opioides de prescripción sin prescripción (Vicodin, Darvon, Percocet)

__ Barbituricos sin prescripción (Amytal, Nembutal y Seconal)

__ Otro tipo de droga o combinación que no ha sido mencionada

__ No Sabe

__ Se rehúsa a contestar

If Q639S is equal to 1 then skip to Q640

If not, then skip to instruction before Q641.

Q640. Otra droga o combinación de drogas [especifica]

____

If Q638 is not less than 1 and Q638 is not greater than 8 then skip to Q641

If not, then skip to instruction before Q642.

Q641. Usa ¿[Response to Q412] drogas inyectadas, o no inyectadas, o a veces las usa de una manera y a veces de la otra? (Elija Uno)

1	Solo usa drogas inyectadas
2	Solo usa drogas no inyectadas
3	A veces usa drogas inyectables
7	No Sabe
8	Se rehúsa a contestar

If Q641 is equal to 1 or Q641 is equal to 3 then skip to Q642

If not, then skip to instruction before Q643.

Q642. ¿Alguna vez has usado la jeringa, agua, cacharro o algodón que ya había sido usada por [Response to Q412]?

1	Si
0	No
8	Se rehúsa a contestar

If Q638 is not equal to 9 and Q638 is not equal to 97 and Q638 is not equal to 98 then skip to Q643

If not, then skip to instruction before Q644.

Q643. ¿Alguna vez te ha ofrecido o animado a usar drogas [Response to Q412]?

1	Si
0	No
8	Se rehúsa a contestar

If Q643 is equal to 1 then skip to Q644

If not, then skip to instruction before Q645.

Q644. ¿Cuál de las siguientes drogas te ha ofrecido o animado a usar [Response to Q412]? (Marque Todas las que Correspondan)

__ Alcohol

__ Mariguana

__ Heroina sola

__ Cocaína crack sola

__ Cocaína en polvo sola

__ Heroína y cocaína juntas (speedball)

__ Metanfetaminas/cristal solo

__ Metanfetaminas/cristal y heroína juntas

__ China White sola

__ China White y heroína juntas

__ China White y metanfetaminas/cristal juntas

__ PCP

__ Alucinógenos como acido, LSD, peyote, mescalina, hongos

__ Inhalantes (por ejemplo, pegamento, gasolina, diluyente de pintura, agua celeste u otros químicos)

__ Tranquilizantes sin prescripción (Rivotril, Diazepam, Valium, Ativan o Restoril)

__ Oxycontin sin prescripción

__ Otros opioides de prescripción sin prescripción (Vicodin, Darvon, Percocet)

__ Barbituricos sin prescripción (Amytal, Nembutal y Seconal)

__ Otro tipo de droga o combinación que no ha sido mencionada

__ Se rehúsa a contestar

If Q644S is equal to 1 then skip to Q645

If not, then skip to Q646.

Q645. Otra droga o combinación de drogas [especifica].

____

Q646. ¿Alguna vez [Response to Q412] te ha animado a dejar de usar drogas?

1 Si

0 No

8 Se rehúsa a contestar

If Q646 is equal to 1 then skip to Q647

If not, then skip to instruction before Q648.

Q647. ¿Alguna vez [Response to Q412] te ha animado a dejar de usar drogas inyectables específicamente?

1 Si

0 No

7 No Sabe

8 Se rehúsa a contestar

If Q638 is not equal to 9 and Q638 is not equal to 97 and Q638 is not equal to 98 then skip to Q648

If not, then skip to instruction before Q649.

Q648. ¿Qué edad tenía [Response to Q412] cuando empezó a usar drogas por primera vez?

__ __

97 No Sabe

98 Se rehúsa a contestar

If Q638 is not equal to 9 and Q638 is not equal to 10 and Q638 is not equal to 97 and Q638 is not equal to 98 then skip to Q649

If not, then skip to Q650.

Q649. Considera el uso diario de drogas de [Response to Q412], ¿Alguna vez en su vida ha usado [Response to Q412] el doble de la dosis de droga o la ha mezclado con otra droga?

1 Si

0 No

7 No Sabe

8 Se rehúsa a contestar

Q650. ¿Alguna vez [Response to Q412] ha vivido en la calle, en un albergue, en un hotel, temporalmente con amigos/familiares, en un carro, o en una casa deshabitada?

1 Si

0 No

7 No Sabe

8 Se rehúsa a contestar

Q651. ¿Dónde vive [Response to Q412] actualmente? (Elija Uno)

1 Condado de San Diego

2 Tijuana

3 Otro (especifica)

7 No Sabe

8 Se rehúsa a contestar

If Q651 is equal to 3 then skip to Q652

If not, then skip to instruction before Q653.

Q652. Por favor especifica donde vive [Response to Q412].

____

If Q651 is equal to 1 and Q638 is not equal to 9 and Q638 is not equal to 97 and Q638 is not equal to 98 then skip to Q653

If not, then skip to instruction before Q654.

Q653. **En los últimos 6 meses**, ¿ha viajado [Response to Q412] a México para comprar u conseguir drogas?

1	Si
0	No
7	No Sabe
8	Se rehúsa a contestar

If Q653 is equal to 1 then skip to Q654

If not, then skip to instruction before Q655.

Q654. ¿Cuál de los siguientes tipos de drogas compró [Response to Q412] en México? (Marque Todas las que Correspondan)

__ Mariguana

__ Heroina

__ Cocaína crack sola

__ Cocaína en polvo sola

__ Heroína y cocaína juntas (speedball)

__ Metanfetaminas/cristal solo

__ Metanfetaminas/cristal y heroína juntas

__ China White sola

__ China White y heroína juntas

__ China White y metanfetaminas/cristal juntas

__ PCP

__ Alucinógenos como acido, LSD, peyote, mescalina, hongos

__ Inhalantes (por ejemplo, pegamento, gasolina, diluyente de pintura, agua celeste u otros químicos)

__ Tranquilizantes sin prescripción (Rivotril, Diazepam, Valium, Ativan o Restoril)

__ Oxycontin sin prescripción

__ Otros opioides de prescripción sin prescripción (Vicodin, Darvon, Percocet)

__ Barbituricos sin prescripción (Amytal, Nembutal y Seconal)

__ Otro tipo de droga o combinación que no ha sido mencionada

__ No Sabe

__ Se rehúsa a contestar

If Q654R is equal to 1 then skip to Q655

If not, then skip to instruction before Q656.

Q655. Por favor especifica qué tipo de drogas compro [Response to Q412] en México.

____

If Q651 is equal to 1 then skip to Q656

If not, then skip to instruction before Q657.

Q656. **En los últimos 6 meses**, ¿ha viajado [Response to Q412] a México para pagar por sexo con dinero o drogas?

1 Si

0 No

7 No Sabe

8 Se rehúsa a contestar

If Q651 is equal to 2 and Q638 is not equal to 9 and Q638 is not equal to 97 and Q638 is not equal to 98 then skip to Q657

If not, then skip to instruction before Q658.

Q657. **En los últimos 6 meses**, ¿ha viajado [Response to Q412] a Estados Unidos para comprar o conseguir drogas?

1 Si

0 No

7 No Sabe

8 Se rehúsa a contestar

If Q657 is equal to 1 then skip to Q658

If not, then skip to instruction before Q659.

Q658. ¿Cuál de los siguientes tipos de drogas compró [Response to Q412] en Estados Unidos? (Marque Todas las que Correspondan)

__ Mariguana

__ Heroína

__ Cocaína crack sola

__ Cocaína en polvo sola

__ Heroína y cocaína juntas (speedball)

__ Metanfetaminas/cristal solo

__ Metanfetaminas/cristal y heroína juntas

__ China White sola

__ China White y heroína juntas

__ China White y metanfetaminas/cristal juntas

__ PCP

__ Alucinógenos como acido, LSD, peyote, mescalina, hongos

__ Inhalantes (por ejemplo, pegamento, gasolina, diluyente de pintura, agua celeste u otros químicos)

__ Tranquilizantes sin prescripción (Rivotril, Diazepam, Valium, Ativan o Restoril)

__ Oxycontin sin prescripción

__ Otros opioides de prescripción sin prescripción (Vicodin, Darvon, Percocet)

__ Barbituricos sin prescripción (Amytal, Nembutal y Seconal)

__ Otro tipo de droga o combinación que no ha sido mencionada

__ No Sabe

__ Se rehúsa a contestar

If Q658R is equal to 1 then skip to Q659

If not, then skip to instruction before Q660.

Q659. Por favor especifica qué tipo de drogas compro [Response to Q412] en Estados Unidos.

____

If Q651 is equal to 2 then skip to Q660

If not, then skip to Q661.

Q660. **En los últimos 6 meses**, ¿[Response to Q412] ha tenido sexo por dinero o drogas con alguien de los Estados Unidos?

1 Si

0 No

7 No Sabe

8 Se rehúsa a contestar

Q661. ¿Alguna vez [Response to Q412] ha sido detenido o arrestado?

1 Si

0 No

7 No Sabe

8 Se rehúsa a contestar

If Q405 is greater than 4 and Q414 is not equal to "Se rehúsa a contestar" and Q414 is not equal to "Saltó" then skip to Q662

If not, then skip to instruction before Q686.

Q662. ¿Qué tan seguido usa drogas [Response to Q414]? (Elija Uno)

01	Todos los días
02	Varias veces a la semana
03	Una vez a la semana
04	Una vez cada dos semanas
05	Una vez al mes
06	Algunas veces al año
07	Una vez al año
08	Menos de una vez al año
09	Nunca
10	Usaba drogas anteriormente pero no en el presente
97	No Sabe
98	Se rehúsa a contestar

If Q662 is not less than 1 and Q662 is not greater than 8 then skip to Q663

If not, then skip to instruction before Q664.

Q663. ¿Cuál de las siguientes drogas usa [Response to Q414]? (Marque Todas las que Correspondan)

__ Alcohol

__ Mariguana

__ Heroína sola

__ Cocaína crack sola

__ Cocaína en polvo sola

__ Heroína y cocaína juntas (speedball)

__ Metanfetaminas/cristal solo

__ Metanfetaminas/cristal y heroína juntas

__ China White sola

__ China White y heroína juntas

__ China White y metanfetaminas/cristal juntas

__ PCP

__ Alucinógenos como acido, LSD, peyote, mescalina, hongos

__ Inhalantes (por ejemplo, pegamento, gasolina, diluyente de pintura, agua celeste u otros químicos)

__ Tranquilizantes sin prescripción (Rivotril, Diazepam, Valium, Ativan o Restoril)

__ Oxycontin sin prescripción

__ Otros opioides de prescripción sin prescripción (Vicodin, Darvon, Percocet)

__ Barbituricos sin prescripción (Amytal, Nembutal y Seconal)

__ Otro tipo de droga o combinación que no ha sido mencionada

__ No Sabe

__ Se rehúsa a contestar

If Q663S is equal to 1 then skip to Q664

If not, then skip to instruction before Q665.

Q664. Otra droga o combinación de drogas [especifica]

____

If Q662 is not less than 1 and Q662 is not greater than 8 then skip to Q665

If not, then skip to instruction before Q666.

Q665. ¿Usa [Response to Q414] drogas inyectadas, o no inyectadas, o a veces las usa de una manera y a veces de la otra? (Elija Uno)

1 Solo usa drogas inyectadas

2 Solo usa drogas no inyectadas

3 A veces usa drogas inyectables

7 No Sabe

8 Se rehúsa a contestar

If Q665 is equal to 1 or Q665 is equal to 3 then skip to Q666

If not, then skip to instruction before Q667.

Q666. ¿Alguna vez has usado la jeringa, agua, cacharro o algodón que ya había sido usada por [Response to Q414]?

1 Si

0 No

8 Se rehúsa a contestar

If Q662 is not equal to 9 and Q662 is not equal to 97 and Q662 is not equal to 98 then skip to Q667

If not, then skip to instruction before Q668.

Q667. ¿Alguna vez te ha ofrecido o animado a usar drogas [Response to Q414]?

1 Si

0 No

8 Se rehúsa a contestar

If Q667 is equal to 1 then skip to Q668

If not, then skip to instruction before Q669.

Q668. ¿Cuál de las siguientes drogas te ha ofrecido o animado a usar [Response to Q414]? (Marque Todas las que Correspondan)

__ Alcohol

__ Mariguana

__ Heroina sola

__ Cocaína crack sola

__ Cocaína en polvo sola

__ Heroína y cocaína juntas (speedball)

__ Metanfetaminas/cristal solo

__ Metanfetaminas/cristal y heroína juntas

__ China White sola

__ China White y heroína juntas

__ China White y metanfetaminas/cristal juntas

__ PCP

__ Alucinógenos como acido, LSD, peyote, mescalina, hongos

__ Inhalantes (por ejemplo, pegamento, gasolina, diluyente de pintura, agua celeste u otros químicos)

__ Tranquilizantes sin prescripción (Rivotril, Diazepam, Valium, Ativan o Restoril)

__ Oxycontin sin prescripción

__ Otros opioides de prescripción sin prescripción (Vicodin, Darvon, Percocet)

__ Barbituricos sin prescripción (Amytal, Nembutal y Seconal)

__ Otro tipo de droga o combinación que no ha sido mencionada

__ Se rehúsa a contestar

If Q668S is equal to 1 then skip to Q669

If not, then skip to Q670.

Q669. Otra droga o combinación de drogas [especifica]

____

Q670. ¿Alguna vez [Response to Q414] te ha animado a dejar de usar drogas?

1	Si
0	No
8	Se rehúsa a contestar

If Q670 is equal to 1 then skip to Q671

If not, then skip to instruction before Q672.

Q671. ¿Alguna vez [Response to Q414] te ha animado a dejar de usar drogas inyectables específicamente?

1	Si
0	No
7	No Sabe
8	Se rehúsa a contestar

If Q662 is not equal to 9 and Q662 is not equal to 97 and Q662 is not equal to 98 then skip to Q672

If not, then skip to instruction before Q673.

Q672. ¿Qué edad tenía [Response to Q414] cuando empezó a usar drogas por primera vez?

__ __	
97	No Sabe
98	Se rehúsa a contestar

If Q662 is not equal to 9 and Q662 is not equal to 10 and Q662 is not equal to 97 and Q662 is not equal to 98 then skip to Q673

If not, then skip to Q674.

Q673. Considera el uso diario de drogas de [Response to Q414], ¿Alguna vez en su vida ha usado [Response to Q414] el doble de la dosis de droga o la ha mezclado con otra droga?

1	Si
0	No
7	No Sabe
8	Se rehúsa a contestar

Q674. ¿Alguna vez [Response to Q414] ha vivido en la calle, en un albergue, en un hotel, temporalmente con amigos/familiares, en un carro, o en una casa deshabitada?

1	Si
0	No
7	No Sabe
8	Se rehúsa a contestar

Q675. ¿Dónde vive [Response to Q414] actualmente? (Elija Uno)

1	Condado de San Diego
2	Tijuana
3	Otro (specifica)
7	No Sabe
8	Se rehúsa a contestar

If Q675 is equal to 3 then skip to Q676

If not, then skip to instruction before Q677.

Q676. Por favor especifica donde vive [Response to Q414].

____

If Q675 is equal to 1 and Q662 is not equal to 9 and Q662 is not equal to 97 and Q662 is not equal to 98 then skip to Q677

If not, then skip to instruction before Q678.

Q677. **En los últimos 6 meses**, ¿ha viajado [Response to Q414] a México para comprar u obtener drogas?

1	Si
0	No
7	No Sabe
8	Se rehúsa a contestar

If Q677 is equal to 1 then skip to Q678

If not, then skip to instruction before Q679.

Q678. ¿Cuál de los siguientes tipos de drogas compró [Response to Q414] en México? (Marque Todas las que Correspondan)

__ Mariguana

__ Heroína

__ Cocaína crack sola

__ Cocaína en polvo sola

__ Heroína y cocaína juntas (speedball)

__ Metanfetaminas/cristal solo

__ Metanfetaminas/cristal y heroína juntas

__ China White sola

__ China White y heroína juntas

__ China White y metanfetaminas/cristal juntas

__ PCP

__ Alucinógenos como acido, LSD, peyote, mescalina, hongos

__ Inhalantes (por ejemplo, pegamento, gasolina, diluyente de pintura, agua celeste u otros químicos)

__ Tranquilizantes sin prescripción (Rivotril, Diazepam, Valium, Ativan o Restoril)

__ Oxycontin sin prescripción

__ Otros opioides de prescripción sin prescripción (Vicodin, Darvon, Percocet)

__ Barbituricos sin prescripción (Amytal, Nembutal y Seconal)

__ Otro tipo de droga o combinación que no ha sido mencionada

__ No Sabe

__ Se rehúsa a contestar

If Q678R is equal to 1 then skip to Q679

If not, then skip to instruction before Q680.

Q679. Por favor especifica qué tipo de drogas compró [Response to Q414] en México.

____

If Q675 is equal to 1 then skip to Q680

If not, then skip to instruction before Q681.

Q680. **En los últimos 6 meses**, ¿ha viajado [Response to Q414] a México para pagar con dinero o drogas por sexo?

1 Si

0 No

7 No Sabe

8 Se rehúsa a contestar

If Q675 is equal to 2 and Q662 is not equal to 9 and Q662 is not equal to 97 and Q662 is not equal to 98 then skip to Q681

If not, then skip to instruction before Q682.

Q681. **En los últimos 6 meses**, ¿ha viajado [Response to Q414] a Estados Unidos para comprar o conseguir drogas?

1 Si

0 No

7 No Sabe

8 Se rehúsa a contestar

If Q681 is equal to 1 then skip to Q682

If not, then skip to instruction before Q683.

Q682. ¿Cuál de los siguientes tipos de drogas compró [Response to Q414] en Estados Unidos? (Marque Todas las que Correspondan)

__ Mariguana

__ Heroína

__ Cocaína crack sola

__ Cocaína en polvo sola

__ Heroína y cocaína juntas (speedball)

__ Metanfetaminas/cristal solo

__ Metanfetaminas/cristal y heroína juntas

__ China White sola

__ China White y heroína juntas

__ China White y metanfetaminas/cristal juntas

__ PCP

__ Alucinógenos como acido, LSD, peyote, mescalina, hongos

__ Inhalantes (por ejemplo, pegamento, gasolina, diluyente de pintura, agua celeste u otros químicos)

__ Tranquilizantes sin prescripción (Rivotril, Diazepam, Valium, Ativan o Restoril)

__ Oxycontin sin prescripción

__ Otros opioides de prescripción sin prescripción (Vicodin, Darvon, Percocet)

__ Barbituricos sin prescripción (Amytal, Nembutal y Seconal)

__ Otro tipo de droga o combinación que no ha sido mencionada

__ No Sabe

__ Se rehúsa a contestar

If Q682R is equal to 1 then skip to Q683

If not, then skip to instruction before Q684.

Q683. Por favor especifica qué tipo de drogas compró [Response to Q414] en Estados Unidos.

____

If Q675 is equal to 2 then skip to Q684

If not, then skip to Q685.

Q684. **En los últimos 6 meses**, ¿[Response to Q414] ha tenido sexo por dinero o drogas con alguien de los Estados Unidos?

1 Si

0 No

7 No Sabe

8 Se rehúsa a contestar

Q685. ¿Alguna vez [Response to Q414] ha sido detenido o arrestado?

1 Si

0 No

7 No Sabe

8 Se rehúsa a contestar

COMPONENTE DE REDES SOCIALES DE SARSCoV-2

READ: LEA: Ahora vamos a cambiar de tema y vamos a hablar más sobre tiempos más recientes en los que has pasado tiempo con otras personas. Como dijimos antes, estamos intentando aprender sobre cómo las personas se conectan unas con l otras y cómo esas conexiones se relacionan para prevenir enfermedades, pero ahora nos vamos a enfocar en la infección SARSCoV-2. El tener nombres e información demográfica nos ayudará a ver cómo las personas en este estudio están conectadas unas a otras, por ejemplo, a través de amigos en común.
Por favor recuerda que todo lo que me digas es confidencial y que nosotros protegeremos tu privacidad y la de las personas que tú menciones. Nosotros nunca en ningún momento usaremos esta información para contactar a las personas que nos has mencionado.

READ: LEA: Por favor piensa sobre los siguientes tipos de personas con las que tú has tenido contacto cercano dentro de los últimos 14 días. Nosotros estamos interesados en las personas con las que has tenido:
Contacto no físico (con 2 metros de distancia) que duró más de una hora
O
Contacto físico
Por favor incluye sus nombres (primer nombre, apellido, apodo) en la parte de abajo. Puedes usar tu teléfono para ayudarte a recordar. Esto es importante. Nosotros nunca vamos a compartir los nombres que tú nos darás. Sólo los/as investigadores/as tendrán acceso a estos nombres.

Q686. Vamos a comenzar con personas en la categoría de **Pareja romántica o sexual o esposo/a** con quien/quienes tú has tenido contacto en los últimos 14 días (nuevamente por contacto cercano, nos referimos a: no contacto físico (dentro de 6 pies de distancia) que haya durado más de 1 hora O contacto físico. ¿Con cuantas personas has tenido contacto? A continuación, vamos a anotar sus nombres (se pueden nombrar hasta 5).

__ __

98 Se rehúsa a contestar

If Q686 is greater than 0 then skip to Q687

If not, then skip to instruction before Q691.

Q687. **Pareja romántica o sexual o esposo/a:** Persona #1 Primer Nombre

____

Q688. **Pareja romántica o sexual o esposo/a:** Persona #1 Apellido

____

Q689. **Pareja romántica o sexual o esposo/a:** Persona #1 Apodo

__ __

Q690. **Pareja romántica o sexual o esposo/a:** Persona #1 Edad__ __

97 No Sabe

98 Se rehúsa a contestar

If Q686 is greater than 1 then skip to Q691

If not, then skip to instruction before Q695.

Q691. **Pareja romántica o sexual o esposo/a:** Persona #2 Primer Nombre

__ __

Q692. **Pareja romántica o sexual o esposo/a:** Persona #2 Apellido

__ __

Q693. **Pareja romántica o sexual o esposo/a:** Persona #2 Apodo

__ __

Q694. **Pareja romántica o sexual o esposo/a:** Persona #2 Edad__ __

97 No Sabe

98 Se rehúsa a contestar

If Q686 is greater than 2 then skip to Q695

If not, then skip to instruction before Q699.

Q695. **Pareja romántica o sexual o esposo/a:** Persona #3 Primer Nombre

__ __

Q696. **Pareja romántica o sexual o esposo/a:** Persona #3 Apellido

__ __

Q697. **Pareja romántica o sexual o esposo/a:** Persona #3 Apodo

__ __

Q698. **Pareja romántica o sexual o esposo/a:** Persona #3 Edad__ __

97 No Sabe

98 Se rehúsa a contestar

If Q686 is greater than 3 then skip to Q699

If not, then skip to instruction before Q703.

Q699. **Pareja romántica o sexual o esposo/a:** Persona #4 Primer Nombre

__ __

Q700. **Pareja romántica o sexual o esposo/a:** Persona #4 Apellido

__ __

Q701. **Pareja romántica o sexual o esposo/a:** Persona #4 Apodo

__ __

Q702. **Pareja romántica o sexual o esposo/a:** Persona #4 Edad__ __

97 No Sabe

98 Se rehúsa a contestar

If Q686 is greater than 4 then skip to Q703

If not, then skip to Q707.

Q703. **Pareja romántica o sexual o esposo/a:** Persona #5 Primer Nombre

__ __

Q704. **Pareja romántica o sexual o esposo/a:** Persona #5 Apellido

__ __

Q705. **Pareja romántica o sexual o esposo/a:** Persona #5 Apodo

__ __

Q706. **Pareja romántica o sexual o esposo/a:** Persona #5 Edad__ __

97 No Sabe

98 Se rehúsa a contestar

Q707. Ahora, por favor piensa en **otros familiares relacionados por vínculo sanguíneo o matrimonio** con quien tú has tenido contacto cercano dentro de los últimos 14 días. ¿Con cuántos de ellos tuviste contacto?

__ __

98 Se rehúsa a contestar

If Q707 is greater than 0 then skip to Q708

If not, then skip to instruction before Q712.

Q708. **Otros familiares relacionados por sangre o matrimonio:** Persona #1 Primer Nombre

__ __

Q709. **Otros familiares relacionados por sangre o matrimonio:** Persona #1 Apellido

__ __

Q710. **Otros familiares relacionados por sangre o matrimonio:** Persona #1 Apodo

__ __

Q711. **Otros familiares relacionados por sangre o matrimonio:** Persona #1 Edad

__ __

97 No Sabe

98 Se rehúsa a contestar

If Q707 is greater than 1 then skip to Q712

If not, then skip to instruction before Q716.

Q712. **Otros familiares relacionados por sangre o matrimonio:** Persona #2 Primer Nombre

____

Q713. **Otros familiares relacionados por sangre o matrimonio:** Persona #2 Apellido

____

Q714. **Otros familiares relacionados por sangre o matrimonio:** Persona #2 Apodo

____

Q715. **Otros familiares relacionados por sangre o matrimonio:** Persona #2 Edad

__ __

97 No Sabe

98 Se rehúsa a contestar

If Q707 is greater than 2 then skip to Q716

If not, then skip to instruction before Q720.

Q716. **Otros familiares relacionados por sangre o matrimonio:** Persona #3 Primer Nombre

____

Q717. **Otros familiares relacionados por sangre o matrimonio:** Persona #3 Apellido

____

Q718. **Otros familiares relacionados por sangre o matrimonio:** Persona #3 Apodo

____

Q719. **Otros familiares relacionados por sangre o matrimonio:** Persona #3 Edad

__ __

97 No Sabe

98 Se rehúsa a contestar

If Q707 is greater than 3 then skip to Q720

If not, then skip to instruction before Q724.

Q720. **Otros familiares relacionados por sangre o matrimonio:** Persona #4 Primer Nombre

____

Q721. **Otros familiares relacionados por sangre o matrimonio:** Persona #4 Apellido

____

Q722. **Otros familiares relacionados por sangre o matrimonio:** Persona #4 Apodo

____

Q723. **Otros familiares relacionados por sangre o matrimonio:** Persona #4 Edad

__ __

97 No Sabe

98 Se rehúsa a contestar

If Q707 is greater than 4 then skip to Q724

If not, then skip to Q728.

Q724. **Otros familiares relacionados por sangre o matrimonio:** Persona #5 Primer Nombre

____

Q725. **Otros familiares relacionados por sangre o matrimonio:** Persona #5 Apellido

____

Q726. **Otros familiares relacionados por sangre o matrimonio:** Persona #5 Apodo

____

Q727. **Otros familiares relacionados por sangre o matrimonio:** Persona #5 Edad

__ __

97 No Sabe

98 Se rehúsa a contestar

Q728. Ahora, por favor piensa en **Amigos Cercanos** con quien tú has tenido contacto cercano dentro de los últimos 14 días. ¿Con cuántos de ellos tuviste contacto?

__ __

98 Se rehúsa a contestar

If Q728 is greater than 0 then skip to Q729

If not, then skip to instruction before Q733.

Q729. **Amigos Cercanos:** Persona #1 Primer Nombre

__ __

Q730. **Amigos Cercanos:** Persona #1 Apellido

__ __

Q731. **Amigos Cercanos:** Persona #1 Apodo

__ __

Q732. **Amigos Cercanos:** Persona #1 Edad __ __

97 No Sabe

98 Se rehúsa a contestar

If Q728 is greater than 1 then skip to Q733

If not, then skip to instruction before Q737.

Q733. **Amigos Cercanos:** Persona #2 Primer Nombre

__ __

Q734. **Amigos Cercanos:** Persona #2 Apellido

__ __

Q735. **Amigos Cercanos:** Persona #2 Apodo

__ __

Q736. **Amigos Cercanos:** Persona #2 Edad __ __

97 No Sabe

98 Se rehúsa a contestar

If Q728 is greater than 2 then skip to Q737

If not, then skip to instruction before Q741.

Q737. **Amigos Cercanos:** Persona #3 Primer Nombre

__ __

Q738. **Amigos Cercanos:** Persona #3 Apellido

__ __

Q739. **Amigos Cercanos:** Persona #3 Apodo

__ __

Q740. **Amigos Cercanos:** Persona #3 Edad __ __

97 No Sabe

98 Se rehúsa a contestar

If Q728 is greater than 3 then skip to Q741

If not, then skip to instruction before Q745.

Q741. **Amigos Cercanos:** Persona #4 Primer Nombre

__ __

Q742. **Amigos Cercanos:** Persona #4 Apellido

__ __

Q743. **Amigos Cercanos:** Persona #4 Apodo

__ __

Q744. **Amigos Cercanos:** Persona #4 Edad __ __

97 No Sabe

98 Se rehúsa a contestar

If Q728 is greater than 4 then skip to Q745

If not, then skip to Q749.

Q745. **Amigos Cercanos:** Persona #5 Primer Nombre

__ __

Q746. **Amigos Cercanos:** Persona #5 Apellido

__ __

Q747. **Amigos Cercanos:** Persona #5 Apodo

__ __

Q748. **Amigos Cercanos:** Persona #5 Edad __ __

97 No Sabe

98 Se rehúsa a contestar

Q749. Ahora, por favor piensa en **Compañeros de cuarto o rumis (e.g. compañeros de casa, de cuarto)** con quien tú has tenido contacto cercano dentro de los últimos 14 días. ¿Con cuántos de ellos tuviste contacto?

__ __

98 Se rehúsa a contestar

If Q749 is greater than 0 then skip to Q750

If not, then skip to instruction before Q754.

Q750. **Compañeros de cuarto (e.g. compañeros de casa, de cuarto):** Persona #1 Primer Nombre

__ __

Q751. **Compañeros de cuarto (e.g. compañeros de casa, de cuarto):** Persona #1 Apellido

____

Q752. **Compañeros de cuarto (e.g. compañeros de casa, de cuarto):** Persona #1 Apodo

____

Q753. **Compañeros de cuarto (e.g. compañeros de casa, de cuarto):** Persona #1 Edad

__ __

97 No Sabe

98 Se rehúsa a contestar

If Q749 is greater than 1 then skip to Q754

If not, then skip to instruction before Q758.

Q754. **Compañeros de cuarto (e.g. compañeros de casa, de cuarto):** Persona #2 Primer Nombre

____

Q755. **Compañeros de cuarto (e.g. compañeros de casa, de cuarto):** Persona #2 Apellido

____

Q756. **Compañeros de cuarto (e.g. compañeros de casa, de cuarto):** Persona #2 Apodo

____

Q757. **Compañeros de cuarto (e.g. compañeros de casa, de cuarto):** Persona #2 Edad

__ __

97 No Sabe

98 Se rehúsa a contestar

If Q749 is greater than 2 then skip to Q758

If not, then skip to instruction before Q762.

Q758. **Compañeros de cuarto (e.g. compañeros de casa, de cuarto):** Persona #3 Primer Nombre

____

Q759. **Compañeros de cuarto (e.g. compañeros de casa, de cuarto):** Persona #3 Apellido

____

Q760. **Compañeros de cuarto (e.g. compañeros de casa, de cuarto):** Persona #3 Apodo

____

Q761. **Compañeros de cuarto (e.g. compañeros de casa, de cuarto):** Persona #3 Edad

__ __

97 No Sabe

98 Se rehúsa a contestar

If Q749 is greater than 3 then skip to Q762

If not, then skip to instruction before Q766.

Q762. **Compañeros de cuarto (e.g. compañeros de casa, de cuarto):** Persona #4 Primer Nombre

____

Q763. **Compañeros de cuarto (e.g. compañeros de casa, de cuarto):** Persona #4 Apellido

____

Q764. **Compañeros de cuarto (e.g. compañeros de casa, de cuarto):** Persona #4 Apodo

____

Q765. **Compañeros de cuarto (e.g. compañeros de casa, de cuarto):** Persona #4 Edad

__ __

97 No Sabe

98 Se rehúsa a contestar

If Q749 is greater than 4 then skip to Q766

If not, then skip to Q770.

Q766. **Compañeros de cuarto (e.g. compañeros de casa, de cuarto):** Persona #5 Primer Nombre

____

Q767. **Compañeros de cuarto (e.g. compañeros de casa, de cuarto):** Persona #5 Apellido

____

Q768. **Compañeros de cuarto (e.g. compañeros de casa, de cuarto):** Persona #5 Apodo

____

Q769. **Compañeros de cuarto (e.g. compañeros de casa, de cuarto):** Persona #5 Edad

__ __

97 No Sabe

98 Se rehúsa a contestar

Q770. Ahora, por favor piensa en **Vecinos** con quien tú has tenido contacto cercano dentro de los últimos 14 días. ¿Con cuántos de ellos tuviste contacto?

__ __

98 Se rehúsa a contestar

If Q770 is greater than 0 then skip to Q771

If not, then skip to instruction before Q775.

Q771. **Vecinos:** Persona #1 Primer Nombre

____

Q772. **Vecinos:** Persona #1 Apellido

____

Q773. **Vecinos:** Persona #1 Apodo

____

Q774. **Vecinos:** Persona #1 Edad __ __

97 No Sabe

98 Se rehúsa a contestar

If Q770 is greater than 1 then skip to Q775

If not, then skip to instruction before Q779.

Q775. **Vecinos:** Persona #2 Primer Nombre

____

Q776. **Vecinos:** Persona #2 Apellido

____

Q777. **Vecinos:** Persona #2 Apodo

____

Q778. **Vecinos:** Persona #2 Edad __ __

97 No Sabe

98 Se rehúsa a contestar

If Q770 is greater than 2 then skip to Q779

If not, then skip to instruction before Q783.

Q779. **Vecinos:** Persona #3 Primer Nombre

____

Q780. **Vecinos:** Persona #3 Apellido

____

Q781. **Vecinos:** Persona #3 Apodo

__ __

Q782. **Vecinos:** Persona #3 Edad __ __

97 No Sabe

98 Se rehúsa a contestar

If Q770 is greater than 3 then skip to Q783

If not, then skip to instruction before Q787.

Q783. **Vecinos:** Persona #4 Primer Nombre

__ __

Q784. **Vecinos:** Persona #4 Apellido

__ __

Q785. **Vecinos:** Persona #4 Apodo

__ __

Q786. **Vecinos:** Persona #4 Edad __ __

97 No Sabe

98 Se rehúsa a contestar

If Q770 is greater than 4 then skip to Q787

If not, then skip to Q791.

Q787. **Vecinos:** Persona #5 Primer Nombre

__ __

Q788. **Vecinos:** Persona #5 Apellido

__ __

Q789. **Vecinos:** Persona #5 Apodo

__ __

Q790. **Vecinos:** Persona #5 Edad __ __

97 No Sabe

98 Se rehúsa a contestar

Q791. Ahora, por favor piensa en los **relacionados con el trabajo (compañeros de trabajo, jefes, clientes):** con quien tú has tenido contacto cercano dentro de los últimos 14 días. ¿Con cuántos de ellos tuviste contacto?

__ __

98 Se rehúsa a contestar

If Q791 is greater than 0 then skip to Q792

If not, then skip to instruction before Q796.

Q792. **Relacionado con trabajo (compañeros de trabajo, jefes, clientes):** Persona #1 Primer Nombre

__ __

Q793. **Relacionado con trabajo (compañeros de trabajo, jefes, clientes):** Persona #1 Apellido

__ __

Q794. **Relacionado con trabajo (compañeros de trabajo, jefes, clientes):** Persona #1 Apodo

__ __

Q795. **Relacionado con trabajo (compañeros de trabajo, jefes, clientes):** Persona #1 Edad

__ __

97 No Sabe

98 Se rehúsa a contestar

If Q791 is greater than 1 then skip to Q796

If not, then skip to instruction before Q800.

Q796. **Relacionado con trabajo (compañeros de trabajo, jefes, clientes):** Persona #2 Primer Nombre

__ __

Q797. **Relacionado con trabajo (compañeros de trabajo, jefes, clientes):** Persona #2 Apellido

__ __

Q798. **Relacionado con trabajo (compañeros de trabajo, jefes, clientes):** Persona #2 Apodo

__ __

Q799. **Relacionado con trabajo (compañeros de trabajo, jefes, clientes):** Persona #2 Edad

__ __

97 No Sabe

98 Se rehúsa a contestar

If Q791 is greater than 2 then skip to Q800

If not, then skip to instruction before Q804.

Q800. **Relacionado con trabajo (compañeros de trabajo, jefes, clientes):** Persona #3 Primer Nombre

__ __

Q801. **Relacionado con trabajo (compañeros de trabajo, jefes, clientes):** Persona #3 Apellido

__ __

Q802. **Relacionado con trabajo (compañeros de trabajo, jefes, clientes):** Persona #3 Apodo

__ __

Q803. **Relacionado con trabajo (compañeros de trabajo, jefes, clientes):** Persona #3 Edad

__ __

97 No Sabe

98 Se rehúsa a contestar

If Q791 is greater than 3 then skip to Q804

If not, then skip to instruction before Q808.

Q804. **Relacionado con trabajo (compañeros de trabajo, jefes, clientes):** Persona #4 Primer Nombre

__ __

Q805. **Relacionado con trabajo (compañeros de trabajo, jefes, clientes):** Persona #4 Apellido

__ __

Q806. **Relacionado con trabajo (compañeros de trabajo, jefes, clientes):** Persona #4 Apodo

__ __

Q807. **Relacionado con trabajo (compañeros de trabajo, jefes, clientes):** Persona #4 Edad

__ __

97 No Sabe

98 Se rehúsa a contestar

If Q791 is greater than 4 then skip to Q808

If not, then skip to Q812.

Q808. **Relacionado con trabajo (compañeros de trabajo, jefes, clientes):** Persona #5 Primer Nombre

__ __

Q809. **Relacionado con trabajo (compañeros de trabajo, jefes, clientes):** Persona #5 Apellido

__ __

Q810. **Relacionado con trabajo (compañeros de trabajo, jefes, clientes):** Persona #5 Apodo

__ __

Q811. **Relacionado con trabajo (compañeros de trabajo, jefes, clientes):** Persona #5 Edad

__ __

97 No Sabe

98 Se rehúsa a contestar

Q812. Ahora, por favor piensa en **Compañeros de escuela:** con quien tú has tenido contacto cercano dentro de los últimos 14 días. ¿Con cuántos de ellos tuviste contacto?

__ __

98 Se rehúsa a contestar

If Q812 is greater than 0 then skip to Q813

If not, then skip to instruction before Q817.

Q813. **Compañeros de escuela:** Persona #1 Primer Nombre

__ __

Q814. **Compañeros de escuela:** Persona #1 Apellido

__ __

Q815. **Compañeros de escuela:** Persona #1 Apodo

__ __

Q816. **Compañeros de escuela:** Persona #1 Edad __ __

97 No Sabe

98 Se rehúsa a contestar

If Q812 is greater than 1 then skip to Q817

If not, then skip to instruction before Q821.

Q817. **Compañeros de escuela:** Persona #2 Primer Nombre

__ __

Q818. **Compañeros de escuela:** Persona #2 Apellido

__ __

Q819. **Compañeros de escuela:** Persona #2 Apodo

__ __

Q820. **Compañeros de escuela:** Persona #2 Edad __ __

97 No Sabe

98 Se rehúsa a contestar

If Q812 is greater than 2 then skip to Q821

If not, then skip to instruction before Q825.

Q821. **Compañeros de escuela:** Persona #3 Primer Nombre

__ __

Q822. **Compañeros de escuela:** Persona #3 Apellido

__ __

Q823. **Compañeros de escuela:** Persona #3 Apodo

__ __

Q824. **Compañeros de escuela:** Persona #3 Edad __ __

97 No Sabe

98 Se rehúsa a contestar

If Q812 is greater than 3 then skip to Q825

If not, then skip to instruction before Q829.

Q825. **Compañeros de escuela:** Persona #4 Primer Nombre

__ __

Q826. **Compañeros de escuela:** Persona #4 Apellido

__ __

Q827. **Compañeros de escuela:** Persona #4 Apodo

__ __

Q828. **Compañeros de escuela:** Persona #4 Edad __ __

97 No Sabe

98 Se rehúsa a contestar

If Q812 is greater than 4 then skip to Q829

If not, then skip to Q833.

Q829. **Compañeros de escuela:** Persona #5 Primer Nombre

__ __

Q830. **Compañeros de escuela:** Persona #5 Apellido

__ __

Q831. **Compañeros de escuela:** Persona #5 Apodo

__ __

Q832. **Compañeros de escuela:** Persona #5 Edad __ __

97 No Sabe

98 Se rehúsa a contestar

Q833. Ahora, por favor piensa en los **relacionados con las drogas o el alcohol** con quien tú has tenido contacto cercano dentro de los últimos 14 días. ¿Con cuántos de ellos tuviste contacto?

__ __

98 Se rehúsa a contestar

If Q833 is greater than 0 then skip to Q834

If not, then skip to instruction before Q838.

Q834. **Relacionado con drogas o alcohol:** Persona #1 Primer Nombre

__ __

Q835. **Relacionado con drogas o alcohol:** Persona #1 Apellido

__ __

Q836. **Relacionado con drogas o alcohol:** Persona #1 Apodo

__ __

Q837. **Relacionado con drogas o alcohol:** Persona #1 Edad __ __

97 No Sabe

98 Se rehúsa a contestar

If Q833 is greater than 1 then skip to Q838

If not, then skip to instruction before Q842.

Q838. **Relacionado con drogas o alcohol:** Persona #2 Primer Nombre

__ __

Q839. **Relacionado con drogas o alcohol:** Persona #2 Apellido

__ __

Q840. **Relacionado con drogas o alcohol:** Persona #2 Apodo

__ __

Q841. **Relacionado con drogas o alcohol:** Persona #2 Edad __ __

97 No Sabe

98 Se rehúsa a contestar

If Q833 is greater than 2 then skip to Q842

If not, then skip to instruction before Q846.

Q842. **Relacionado con drogas o alcohol:** Persona #3 Primer Nombre

____

Q843. **Relacionado con drogas o alcohol:** Persona #3 Apellido

____

Q844. **Relacionado con drogas o alcohol:** Persona #3 Apodo

____

Q845. **Relacionado con drogas o alcohol:** Persona #3 Edad __ __

97 No Sabe

98 Se rehúsa a contestar

If Q833 is greater than 3 then skip to Q846

If not, then skip to instruction before Q850.

Q846. **Relacionado con drogas o alcohol:** Persona #4 Primer Nombre

____

Q847. **Relacionado con drogas o alcohol:** Persona #4 Apellido

____

Q848. **Relacionado con drogas o alcohol:** Persona #4 Apodo

____

Q849. **Relacionado con drogas o alcohol:** Persona #4 Edad __ __

97 No Sabe

98 Se rehúsa a contestar

If Q833 is greater than 4 then skip to Q850

If not, then skip to Q854.

Q850. **Relacionado con drogas o alcohol:** Persona #5 Primer Nombre

__ __

Q851. **Relacionado con drogas o alcohol:** Persona #5 Apellido

__ __

Q852. **Relacionado con drogas o alcohol:** Persona #5 Apodo

__ __

Q853. **Relacionado con drogas o alcohol:** Persona #5 Edad __ __

97 No Sabe

98 Se rehúsa a contestar

Q854. Ahora, por favor piensa en **Personas que brindan un servicio** con quien tú has tenido contacto cercano dentro de los últimos 14 días. ¿Con cuántos de ellos tuviste contacto?

__ __

98 Se rehúsa a contestar

If Q854 is greater than 0 then skip to Q855

If not, then skip to instruction before Q859.

Q855. **Personas que proveen un servicio:** Persona #1 Primer Nombre

__ __

Q856. **Personas que proveen un servicio:** Persona #1 Apellido

__ __

Q857. **Personas que proveen un servicio:** Persona #1 Apodo

__ __

Q858. **Personas que proveen un servicio:** Persona #1 Edad __ __

97 No Sabe

98 Se rehúsa a contestar

If Q854 is greater than 1 then skip to Q859

If not, then skip to instruction before Q863.

Q859. **Personas que proveen un servicio:** Persona #2 Primer Nombre

__ __

Q860. **Personas que proveen un servicio:** Persona #2 Apellido

__ __

Q861. **Personas que proveen un servicio:** Persona #2 Apodo

__ __

Q862. **Personas que proveen un servicio:** Persona #2 Edad __ __

97 No Sabe

98 Se rehúsa a contestar

If Q854 is greater than 2 then skip to Q863

If not, then skip to instruction before Q867.

Q863. **Personas que proveen un servicio:** Persona #3 Primer Nombre

__ __

Q864. **Personas que proveen un servicio:** Persona #3 Apellido

__ __

Q865. **Personas que proveen un servicio:** Persona #3 Apodo

__ __

Q866. **Personas que proveen un servicio:** Persona #3 Edad __ __

97 No Sabe

98 Se rehúsa a contestar

If Q854 is greater than 3 then skip to Q867

If not, then skip to instruction before Q871.

Q867. **Personas que proveen un servicio:** Persona #4 Primer Nombre

__ __

Q868. **Personas que proveen un servicio:** Persona #4 Apellido

__ __

Q869. **Personas que proveen un servicio:** Persona #4 Apodo

__ __

Q870. **Personas que proveen un servicio:** Persona #4 Edad__ __

97 No Sabe

98 Se rehúsa a contestar

If Q854 is greater than 4 then skip to Q871

If not, then skip to Q875.

Q871. **Personas que proveen un servicio:** Persona #5 Primer Nombre

__ __

Q872. **Personas que proveen un servicio:** Persona #5 Apellido

__ __

Q873. **Personas que proveen un servicio:** Persona #5 Apodo

__ __

Q874. Edad

__ __

97 No Sabe

98 Se rehúsa a contestar

Q875. Ahora, por favor piensa en **Otros (e.g. personas que viste en grupos comunitarios, servicios religiosos, deportes o clases de ejercicio, escuela, etc.)** con quien tú has tenido contacto cercano dentro de los últimos 14 días. ¿Con cuántos de ellos tuviste contacto?

__ __

98 Se rehúsa a contestar

If Q875 is greater than 0 then skip to Q876

If not, then skip to instruction before Q880.

Q876. **Otros (e.g. personas que viste en grupos comunitarios, servicios religiosos, deportes o clases de ejercicio, escuela, etc.):** Persona #1 Primer Nombre

__ __

Q877. **Otros (e.g. personas que viste en grupos comunitarios, servicios religiosos, deportes o clases de ejercicio, escuela, etc.):** Persona #1 Apellido

__ __

Q878. **Otros (e.g. personas que viste en grupos comunitarios, servicios religiosos, deportes o clases de ejercicio, escuela, etc.):** Persona #1 Apodo

__ __

Q879. **Otros (e.g. personas que viste en grupos comunitarios, servicios religiosos, deportes o clases de ejercicio, escuela, etc.):** Persona #1 Edad

__ __

97 No Sabe

98 Se rehúsa a contestar

If Q875 is greater than 1 then skip to Q880

If not, then skip to instruction before Q884.

Q880. **Otros (e.g. personas que viste en grupos comunitarios, servicios religiosos, deportes o clases de ejercicio, escuela, etc.):** Persona #2 Primer Nombre

__ __

Q881. **Otros (e.g. personas que viste en grupos comunitarios, servicios religiosos, deportes o clases de ejercicio, escuela, etc.):** Persona #2 Apellido

__ __

Q882. **Otros (e.g. personas que viste en grupos comunitarios, servicios religiosos, deportes o clases de ejercicio, escuela, etc.):** Persona #2 Apodo

__ __

Q883. **Otros (e.g. personas que viste en grupos comunitarios, servicios religiosos, deportes o clases de ejercicio, escuela, etc.):** Persona #2 Edad

__ __

97 No Sabe

98 Se rehúsa a contestar

If Q875 is greater than 2 then skip to Q884

If not, then skip to instruction before Q888.

Q884. **Otros (e.g. personas que viste en grupos comunitarios, servicios religiosos, deportes o clases de ejercicio, escuela, etc.):** Persona #3 Pimer Nombre

__ __

Q885. **Otros (e.g. personas que viste en grupos comunitarios, servicios religiosos, deportes o clases de ejercicio, escuela, etc.):** Persona #3 Apellido

____

Q886. **Otros (e.g. personas que viste en grupos comunitarios, servicios religiosos, deportes o clases de ejercicio, escuela, etc.):** Persona #3 Apodo

____

Q887. **Otros (e.g. personas que viste en grupos comunitarios, servicios religiosos, deportes o clases de ejercicio, escuela, etc.):** Persona #3 Edad

__ __

97 No Sabe

98 Se rehúsa a contestar

If Q875 is greater than 3 then skip to Q888

If not, then skip to instruction before Q892.

Q888. **Otros (e.g. personas que viste en grupos comunitarios, servicios religiosos, deportes o clases de ejercicio, escuela, etc.):** Persona #4 Primer Nombre

____

Q889. **Otros (e.g. personas que viste en grupos comunitarios, servicios religiosos, deportes o clases de ejercicio, escuela, etc.):** Persona #4 Apellido

____

Q890. **Otros (e.g. personas que viste en grupos comunitarios, servicios religiosos, deportes o clases de ejercicio, escuela, etc.):** Persona #4 Apodo

____

Q891. **Otros (e.g. personas que viste en grupos comunitarios, servicios religiosos, deportes o clases de ejercicio, escuela, etc.):** Persona #4 Edad

__ __

97 No Sabe

98 Se rehúsa a contestar

If Q875 is greater than 4 then skip to Q892

If not, then skip to instruction before Q896.

Q892. **Otros (e.g. personas que viste en grupos comunitarios, servicios religiosos, deportes o clases de ejercicio, escuela, etc.):** Persona #5 Primer Nombre

____

Q893. **Otros (e.g. personas que viste en grupos comunitarios, servicios religiosos, deportes o clases de ejercicio, escuela, etc.):** Persona #5 Apellido

____

Q894. **Otros (e.g. personas que viste en grupos comunitarios, servicios religiosos, deportes o clases de ejercicio, escuela, etc.):** Persona #5 Apodo

____

Q895. **Otros (e.g. personas que viste en grupos comunitarios, servicios religiosos, deportes o clases de ejercicio, escuela, etc.):** Persona #5 Edad

__ __

97 No Sabe

98 Se rehúsa a contestar

If Q686 + Q707 + Q728 + Q749 + Q770 + Q791 + Q812 + Q833 + Q854 + Q875 is greater than 0 then skip to instruction before Q896

If not, then skip to instruction before Q899.

READ: LEA: De las personas que indicaste, por favor piensa con cuáles 5 personas has tenido el mayor contacto. Esto puede ser el mayor número de horas sin contacto físico (a 2 metros de distancia) con una persona y/o el mayor contacto físico. Por favor escribe sus nombres a continuación.

Q896. **A continuación, vamos a anotar sus nombres.**
Mayor contacto: Persona #1 Primer Nombre

____

Q897. **Mayor contacto:** Persona #1 Apellido

____

Q898. **Mayor contacto:** Persona #1 Apodo

____

If Q686 + Q707 + Q728 + Q749 + Q770 + Q791 + Q812 + Q833 + Q854 + Q875 is greater than 1 then skip to Q899

If not, then skip to instruction before Q902.

Q899. **Mayor contacto:** Persona #2 Primer Nombre

____

Q900. **Mayor contacto:** Persona #2 Apellido

____

Q901. **Mayor contacto:** Persona #2 Apodo

____

If Q686 + Q707 + Q728 + Q749 + Q770 + Q791 + Q812 + Q833 + Q854 + Q875 is greater than 2 then skip to Q902

If not, then skip to instruction before Q905.

Q902. **Mayor contacto:** Persona #3 Primer Nombre

____

Q903. **Mayor contacto:** Persona #3 Apellido

____

Q904. **Mayor contacto:** Persona #3 Apodo

____

If Q686 + Q707 + Q728 + Q749 + Q770 + Q791 + Q812 + Q833 + Q854 + Q875 is greater than 3 then skip to Q905

If not, then skip to instruction before Q908.

Q905. **Mayor contacto:** Persona #4 Primer Nombre

_ _

Q906. **Mayor contacto:** Persona #4 Apellido

_ _

Q907. **Mayor contacto:** Persona #4 Apodo

_ _

If Q686 + Q707 + Q728 + Q749 + Q770 + Q791 + Q812 + Q833 + Q854 + Q875 is greater than 4 then skip to Q908

If not, then skip to instruction before Q911.

Q908. **Mayor contacto:** Persona #5 Primer Nombre

_ _

Q909. **Mayor contacto:** Persona #5 Apellido

_ _

Q910. **Mayor contacto:** Persona #5 Apodo

_ _

MCL1 = If(Q897 is not equal to 97 and Q897 is not equal to 98, Q897,)

MCN1 = If(Q898 is not equal to 97 and Q898 is not equal to 98, Q898,)

MCL2 = If(Q900 is not equal to 97 and Q900 is not equal to 98, Q900,)

MCN2 = If(Q901 is not equal to 97 and Q901 is not equal to 98, Q901,)

MCL3 = If(Q903 is not equal to 97 and Q903 is not equal to 98, Q903,)

MCN3 = If(Q904 is not equal to 97 and Q904 is not equal to 98, Q904,)

MCL4 = If(Q906 is not equal to 97 and Q906 is not equal to 98, Q906,)

MCN4 = If(Q907 is not equal to 97 and Q907 is not equal to 98, Q907,)

MCL5 = If(Q909 is not equal to 97 and Q909 is not equal to 98, Q909,)

MCN5 = If(Q910 is not equal to 97 and Q910 is not equal to 98, Q910,)

READ: Ahora te vamos a hacer algunas preguntas de seguimiento sobre las 5 personas con las que has tenido más contacto en los últimos 14 días.

If Q686 + Q707 + Q728 + Q749 + Q770 + Q791 + Q812 + Q833 + Q854 + Q875 is greater than 0 and Q896 is not equal to "Se rehúsa a contestar" and Q896 is not equal to "Saltó" then skip to Q911

If not, then skip to instruction before Q954.

Q911. **¿Es [Response to Q896] [MCL1] ([MCN1]) hombre, mujer, transgénero, no-binario u otro?**
(Elija Uno)

1	Hombre
2	Mujer
3	Mujer Trans
4	Hombre Trans
5	No-binario/ genero no-binario / género fluido/ agénero
6	Otra identidad no en la lista
8	Se rehúsa a contestar

If Q911 is equal to 6 then skip to Q912

If not, then skip to Q913.

Q912. Por favor especifica la identidad de género de [Response to Q896].

____

Q913. ¿Dónde vive [Response to Q896] principalmente? (Elija Uno)

1 Mexico

2 Estados Unidos

3 Otro

7 No Sabe

8 Se rehúsa a contestar

If Q913 is equal to 3 then skip to Q914

If not, then skip to Q915.

Q914. Por favor especifica donde vive principalmente [Response to Q896].

____

Q915. **¿Conociste a [Response to Q896] por primera vez en los últimos 14 días?**

1 Si

0 No

8 Se rehúsa a contestar

Q916. **¿Cuántas veces has tenido contacto cercano (dentro de 2 metros) con [Response to Q896] en los últimos 14 días?**

__ __

98 Se rehúsa a contestar

Q917. En promedio, durante los días que viste a [Response to Q896], ¿Cuánto tiempo (e.g. horas, minuto) duraste en contacto cercano con él/ella? Entrevistador: el tiempo máximo por día es 24 horas.

__ __ HOURS

__ __ MINUTES

98 Se rehúsa a contestar (Hours)

Q918. ¿Cuándo fue la última vez que tuviste contacto cercano con [Response to Q896] en los últimos 14 días? Entrevistador: Anotar cuántos días hace que el/la participante tuvo contacto cercano por última vez con la persona en cuestión. Si la última vez fue hoy, ingresa 0.

__ __ DíAS

98 Se rehúsa a contestar (Días)

If Q916 is greater than 1 then skip to Q919

If not, then skip to Q920.

Q919. Durante el último contacto, ¿Por cuantos días consecutivos viste a [Response to Q896]?

__ __

98 Se rehúsa a contestar

MC1FDAY = If(Q918 is equal to 1, Q918, If(Q918 is not equal to 98 and Q918 is greater than 1, Q918 + Q919, "Falta"))

Q920. La última vez que viste a [Response to Q896], ¿Qué tipo de contacto tuviste con esta persona? (elija todas las que apliquen) (Marque Todas las que Correspondan)

__ Contacto físico, no sexual [e.g. saludo de mano, abrazo, contacto de piel, etc.]

__ Contacto físico, sexual

__ No físico [e.g. conversación en persona, comer/beber, junta]

__ Se rehúsa a contestar

Q921. Cuando viste a [Response to Q896] por última vez, ¿en cuál de los siguientes lugares fué? (por favor escoge todas las que apliquen) (Marque Todas las que Correspondan)

__ En casa/casa de alguien

__ Trabajo

__ Restaurante o café

__ Tiempo Libre (i.e. cine, bar, club nocturno)

__ Escuela/educación

__ Ejercicio o relacionado al estado físico

__ Servicio personal (i.e. corte de cabello, masaje)

__ Servicio religioso

__ Grupo comunitario

__ Parque

__ Calle

__ El Bordo/Canal

__ Picadero

__ Otro

__ Se rehúsa a contestar

If Q921N is equal to 1 then skip to Q922

If not, then skip to instruction before Q923.

Q922. Por favor especifica el lugar (e.g. el nombre del lugar y las calles que cruzan o la colonia) donde tuviste contacto cercano con [Response to Q896] por última vez.

____

If Q921A is equal to 1 then skip to Q923

If not, then skip to instruction before Q924.

Q923. Tu nos dijiste que el último contacto cercano con [Response to Q896] fué en tu casa/la casa de alguien más. ¿Dónde se ubica esta casa? ¿Podrías darnos los nombres de las calles que cruzan la intersección más cercana? Entrevistador: Por favor anotar los nombres de las calles que lo cruzan. Si el participante no puede darnos esta información, pregúntele el nombre de la colonia y anote esa información.

__ __

If Q921B is equal to 1 then skip to Q924

If not, then skip to instruction before Q925.

Q924. Tú nos dijiste que el último contacto cercano con [Response to Q896] fué en tu trabajo. ¿Nos podrías dar el nombre y la dirección de tu lugar de trabajo? Entrevistador: Si el lugar de trabajo no tiene nombre o dirección, anote cualquier información que se dé para la ubicación (e.g. nombre de calles, intersección más cercana, colonia)

__ __

If Q921C is equal to 1 then skip to Q925

If not, then skip to instruction before Q926.

Q925. Tú nos dijiste que la última vez que estuviste en contacto cercano con [Response to Q896] fué en un restaurante/café. ¿Podrías darnos el nombre de este lugar y las calles que cruzan/colonia donde se ubica este restaurante?

__ __

If Q921D is equal to 1 then skip to Q926

If not, then skip to instruction before Q927.

Q926. Tú nos dijiste que la última vez que estuviste en contacto cercano con [Response to Q896] ocurrió en un lugar de tiempo libre (e.g. teatro, bar, club nocturno). ¿Podrías darnos el nombre del lugar y las calles que lo cruzan/colonia donde se ubica este lugar?

__ __

If Q921E is equal to 1 then skip to Q927

If not, then skip to instruction before Q928.

Q927. Tú nos dijiste que la última vez que estuviste en contacto cercano con [Response to Q896] fué en tu escuela o lugar de estudio. ¿Podrías darnos el nombre del lugar y las calles que cruzan/colonia donde se ubica este lugar?

— —
— — — — — — — — — — — — — —

If Q921F is equal to 1 then skip to Q928

If not, then skip to instruction before Q929.

Q928. Tú nos dijiste que la última vez que estuviste en contacto cercano con [Response to Q896] fué en un gimnasio/centro fitness. ¿Podrías darnos el nombre del lugar y las calles que cruzan/colonia donde se ubica este lugar?

— —
— — — — — — — — — — — — — —

If Q921G is equal to 1 then skip to Q929

If not, then skip to instruction before Q930.

Q929. Tú nos dijiste que la última vez que estuviste en contacto cercano con [Response to Q896] fué en un lugar donde dan servicios personales (e.g. corte de pelo, masaje). ¿Podrías darnos el nombre del lugar y las calles que cruzan/colonia donde se ubica este lugar?

— —
— — — — — — — — — — — — — —

If Q921H is equal to 1 then skip to Q930

If not, then skip to instruction before Q931.

Q930. Tú nos dijiste que la última vez que estuviste en contacto cercano con [Response to Q896] fué en un lugar de servicio religioso. ¿Podrías darnos el nombre del lugar y las calles que cruzan/colonia donde se ubica este lugar?

— —
— — — — — — — — — — — — — —

If Q921I is equal to 1 then skip to Q931

If not, then skip to instruction before Q932.

Q931. Tú nos dijiste que la última vez que estuviste en contacto cercano con [Response to Q896] fué en un centro comunitario/de reunión grupal. ¿Podrías darnos el nombre del lugar y las calles que cruzan/colonia donde se ubica este lugar?

— —

If Q921J is equal to 1 then skip to Q932

If not, then skip to instruction before Q933.

Q932. Tú nos dijiste que la última vez que estuviste en contacto cercano con [Response to Q896] fué en un parque. ¿Podrías darnos el nombre del lugar y las calles que cruzan/colonia donde se ubica este lugar?

— —

If Q921K is equal to 1 then skip to Q933

If not, then skip to instruction before Q934.

Q933. Tú nos dijiste que la última vez que estuviste en contacto cercano con [Response to Q896] fué en la calle. ¿Podrías darnos el nombre del lugar y las calles que cruzan/colonia donde se ubica este lugar?

— —

If Q921M is equal to 1 then skip to Q934

If not, then skip to instruction before Q935.

Q934. Tú nos dijiste que la última vez que estuviste en contacto cercano con [Response to Q896] fué en un picadero. ¿En qué picadero fué? (Elija Uno)

01	Las Palmitas/Zona Norte
02	Los Pinos/ Zona Norte
03	Las Bombas/ Zona Norte
04	Los Aragón/ Zona Norte
05	La Veinte/ Bordo, Col. Veinte de Noviembre
06	El Azteca/ Cerro Colorado
07	Oxxo, Calle 5ta Zona Norte
08	Bordo/ Los Álamos, Mundo divertido
09	Cañón Guerrero/Maclovio Herrera Colonia Guerrero
10	Col. Guaycura (casa privada)
11	Col. Presidentes (Infonavit edificio)
12	Florido 1era Sección (casa privada)
13	Florido 1era Sección (Bomberos, conecta)
14	Col. Francisco Villa (edificio abandonado)
15	Cañón del Pato/ Colonia Villa
16	Col. Postal (casa privada)
17	Col. del Río (Parte baja, barranco)
18	Sánchez Taboada (Av. Sánchez Taboada y Parque Industrial)
19	La cima (casa abandonado)
20	Otay módulos
21	Otro
98	Se rehúsa a contestar

If Q934 is equal to 21 then skip to Q935

If not, then skip to instruction before Q936.

Q935. Por favor especifica en cuál picadero (e.g. nombre del lugar y calles que cruzan o colonia) fué el último contacto cercano con [Response to Q896].

____

If Q916 is greater than 1 then skip to Q936

If not, then skip to instruction before Q954.

Q936. Por favor piensa en alguna otra vez que tuviste contacto cercano con [Response to Q896] en los últimos 14 días, aparte del contacto cercano que nos acaba de describir con la persona en cuestión. Cuando decimos alguna otra vez, queremos decir un contacto en el tu viste a [Response to Q896] en persona y luego se separaron, ¿Cuántos dias hace que se vieron?

__ __ DíAS

98 Se rehúsa a contestar (Días)

If Q936 is less than MC1FDAY then READ: "Este tiempo es más reciente que el tiempo que Ud. indicó que tuvo el último contacto. Por favor realice las correcciones necesarias. TRANSLATION" and skip to Q936.

If Q916 is greater than 1 then skip to Q937

If not, then skip to Q938.

Q937. Durante este contacto, ¿Cuántos días consecutivos viste a [Response to Q896]?

__ __

98 Se rehúsa a contestar

Q938. Cuándo viste a [Response to Q896] en esa ocasión, ¿qué tipo de contacto tuviste con él/ella? (Elije todas las que apliquen) (Marque Todas las que Correspondan)

__ Contacto físico, no sexual [e.g. saludo de mano, abrazo, contacto de piel, etc.]

__ Contacto físico, sexual

__ No físico [e.g. conversación en persona, comer/beber, junta]

__ Se rehúsa a contestar

Q939. Cuándo viste a [Response to Q896] en esa ocasión, ¿en cuál de los siguientes lugares fue que tuvieron contacto cercano? (elije todas las que apliquen) (Marque Todas las que Correspondan)

__ En casa/casa de alguien

__ Trabajo

__ Restaurante o Café

__ Tiempo Libre (i.e. cine, bar, club nocturno)

__ Escuela/educación

__ Ejercicio o relacionado al estado físico

__ Servicio personal (i.e. corte de cabello, masaje)

__ Servicio religioso

__ Grupo comunitario

__ Parque

__ Calle

__ El Bordo/Canal

__ Picadero

__ Otro

__ Se rehúsa a contestar

If Q939N is equal to 1 then skip to Q940

If not, then skip to instruction before Q941.

Q940. Por favor especifica el lugar (e.g. nombre del lugar y las calles que cruzan o la colonia) donde fué ese contacto cercano con [Response to Q896].

__ __

If Q939A is equal to 1 then skip to Q941

If not, then skip to instruction before Q942.

Q941. Tú nos dijiste que el último contacto cercano con [Response to Q896] fué en tu casa/casa de alguien más. ¿Dónde se ubica esta casa? ¿Podrías darnos los nombres de las calles que cruzan la intersección más cercana? Entrevistador: Por favor anotar los nombres de las calles que lo cruzan. Si el participante no puede darnos esta información, pregúntele el nombre de la colonia y anote esa información.

— —

If Q939B is equal to 1 then skip to Q942

If not, then skip to instruction before Q943.

Q942. Tú nos dijiste que el último contacto cercano con [Response to Q896] fué en tu trabajo. ¿Nos podrías dar el nombre y la dirección de tu lugar de trabajo? Entrevistador: Si el lugar de trabajo no tiene nombre o dirección, anotar cualquier información que se dé para la ubicación (e.g. nombre de calles, intersección más cercana, colonia)

— —

If Q939C is equal to 1 then skip to Q943

If not, then skip to instruction before Q944.

Q943. Tú nos dijiste que la última vez que estuviste en contacto cercano con [Response to Q896] fué en un restaurante/café. ¿Podrías darnos el nombre de este lugar y las calles que cruzan/colonia donde se ubica este restaurante?

— —

If Q939D is equal to 1 then skip to Q944

If not, then skip to instruction before Q945.

Q944. Tú nos dijiste que la última vez que estuviste en contacto cercano con [Response to Q896] fué en un lugar de tiempo libre (e.g. teatro, bar, club nocturno). ¿Podrías darnos el nombre del lugar y las calles que cruzan/colonia donde se ubica este lugar?

— —

If Q939E is equal to 1 then skip to Q945

If not, then skip to instruction before Q946.

Q945. Tú nos dijiste que la última vez que estuviste en contacto cercano con [Response to Q896] fué en tu escuela o lugar de estudio. ¿Podrías darnos el nombre del lugar y las calles que cruzan/colonia donde se ubica este lugar?

— —
— — — — — — — — — — — — — — —

If Q939F is equal to 1 then skip to Q946

If not, then skip to instruction before Q947.

Q946. Tú nos dijiste que la última vez que estuviste en contacto cercano con [Response to Q896] fué en un gimnasio/centro fitness. ¿Podrías darnos el nombre del lugar y las calles que cruzan/colonia donde se ubica este lugar?

— —
— — — — — — — — — — — — — — —

If Q939G is equal to 1 then skip to Q947

If not, then skip to instruction before Q948.

Q947. Tú nos dijiste que la última vez que estuviste en contacto cercano con [Response to Q896] fué en un lugar donde dan servicios personales (e.g. corte de pelo, masaje). ¿Podrías darnos el nombre del lugar y las calles que cruzan/colonia donde se ubica este lugar?

— —
— — — — — — — — — — — — — — —

If Q939H is equal to 1 then skip to Q948

If not, then skip to instruction before Q949.

Q948. Tú nos dijiste que la última vez que estuviste en contacto cercano con [Response to Q896] fué en un lugar de servicio religioso. ¿Podrías darnos el nombre del lugar y las calles que cruzan/colonia donde se ubica este lugar?

— —
— — — — — — — — — — — — — — —

If Q939I is equal to 1 then skip to Q949

If not, then skip to instruction before Q950.

Q949. Tú nos dijiste que la última vez que estuviste en contacto cercano con [Response to Q896] fué en un centro comunitario/de reunión grupal. ¿Podrías darnos el nombre del lugar y las calles que cruzan/colonia donde se ubica este lugar?

— —
— — — — — — — — — — — — — —

If Q939J is equal to 1 then skip to Q950

If not, then skip to instruction before Q951.

Q950. Tú nos dijiste que la última vez que estuviste en contacto cercano con [Response to Q896] fué en un parque. ¿Podrías darnos el nombre del lugar y las calles que cruzan/colonia donde se ubica este lugar?

— —
— — — — — — — — — — — — — —

If Q939K is equal to 1 then skip to Q951

If not, then skip to instruction before Q952.

Q951. Tú nos dijiste que la última vez que estuviste en contacto cercano con [Response to Q896] fué en la calle ¿Podrías darnos el nombre del lugar y las calles que cruzan/colonia donde se ubica este lugar?

— —
— — — — — — — — — — — — — —

If Q939L is equal to 1 then skip to Q952

If not, then skip to instruction before Q953.

Q952. Tú nos dijiste que la última vez que estuviste en contacto cercano con [Response to Q896] fué en un picadero. ¿En qué picadero fué? (Elija Uno)

01	Las Palmitas/Zona Norte
02	Los Pinos/ Zona Norte
03	Las Bombas/ Zona Norte
04	Los Aragón/ Zona Norte
05	Los Aragón/ Zona Norte
06	El Azteca/ Cerro Colorado
07	Oxxo, Calle 5ta Zona Norte
08	Bordo/ Los Álamos, Mundo divertido
09	Cañón Guerrero/Maclovio Herrera Colonia Guerrero
10	Col. Guaycura (casa privada)
11	Col. Presidentes (Infonavit edificio)
12	Florido 1era Sección (casa privada)
13	Florido 1era Sección (Bomberos, conecta)
14	Col. Francisco Villa (edificio abandonado)
15	Cañón del Pato/ Colonia Villa
16	Col. Postal (casa privada)
17	Col. del Río (Parte baja, barranco)
18	Sánchez Taboada (Av. Sánchez Taboada y Parque Industrial)
19	La cima (casa abandonado)
20	Otay módulos
21	Otro
98	Se rehúsa a contestar

If Q952 is equal to 21 then skip to Q953

If not, then skip to instruction before Q954.

Q953. Por favor especifica en qué picadero fué (e.g. nombre del lugar y calles que cruzan o colonia) el último contacto cercano con [Response to Q896].

____

If Q686 + Q707 + Q728 + Q749 + Q770 + Q791 + Q812 + Q833 + Q854 + Q875 is greater than 1 and Q899 is not equal to "Se rehúsa a contestar" and Q899 is not equal to "Saltó" then skip to Q954

If not, then skip to instruction before Q997.

Q954. ¿Es [Response to Q899] [MCL2] ([MCN2]) hombre, mujer, transgénero, no-binario u otro? (Elija Uno)

1 Hombre

2 Mujer

3 Mujer Trans

4 Hombre Trans

5 No-binario/ genero no-binario / género fluido/ agénero

6 Otra identidad no en la lista

8 Se rehúsa a contestar

If Q954 is equal to 6 then skip to Q955

If not, then skip to Q956.

Q955. Por favor especifica la identidad de género de [Response to Q899].

____

Q956. ¿Dónde vive [Response to Q899] principalmente? (Elija Uno)

1 Mexico

2 Estados Unidos

3 Otro

7 No Sabe

8 Se rehúsa a contestar

If Q956 is equal to 3 then skip to Q957

If not, then skip to Q958.

Q957. Por favor especifica dónde vive principalmente [Response to Q899].

— —

Q958. ¿Conociste a [Response to Q899] por primera vez en los últimos 14 días?

1 Si

0 No

8 Se rehúsa a contestar

Q959. ¿Cuántas veces has tenido contacto cercano (dentro de 2 metros) con [Response to Q899] en los últimos 14 días?

— —

98 Se rehúsa a contestar

Q960. En promedio, durante los días que viste a [Response to Q899], ¿Cuánto tiempo (e.g. horas, minuto) duraste en contacto cercano con él/ella? Entrevistador: el tiempo máximo por día es 24 horas.

— — HOURS

— — MINUTES

98 Se rehúsa a contestar (Hours)

Q961. ¿Cuándo fue la última vez que tuviste contacto cercano con [Response to Q899] en los últimos 14 días? Entrevistador: Anotar cuántos días hace que el/la participante tuvo contacto cercano por última vez con la persona en cuestión. Si la última vez fue hoy, ingresa 0.

__ __ DíAS

98 Se rehúsa a contestar (Días)

If Q959 is greater than 1 then skip to Q962

If not, then skip to Q963.

Q962. Durante el último contacto, ¿Por cuántos días consecutivos viste a [Response to Q899]?

__ __

98 Se rehúsa a contestar

MC2FDAY = If(Q961 is equal to 1, Q961, If(Q961 is not equal to 98 and Q961 is greater than 1, Q961 + Q962, "Falta"))

Q963. La última vez que viste a [Response to Q899], ¿Qué tipo de contacto tuviste con esta persona? (elija todas las que apliquen) (Marque Todas las que Correspondan)

__ Contacto físico, no sexual [e.g. saludo de mano, abrazo, contacto de piel, etc.]

__ Contacto físico, sexual

__ No físico [e.g. conversación en persona, comer/beber, junta]

__ Se rehúsa a contestar

Q964. Cuando viste a [Response to Q899] durante el contacto, ¿en cuál de los siguientes lugares fué? (por favor escoge todas las que apliquen) (Marque Todas las que Correspondan)

__ En casa/casa de alguien

__ Trabajo

__ Restaurante o café

__ Tiempo Libre (i.e. cine, bar, club nocturno)

__ Escuela/educación

__ Ejercicio o relacionado al estado físico

__ Servicio personal (i.e. corte de cabello, masaje)

__ Servicio religioso

__ Grupo comunitario

__ Parque

__ Calle

__ El Bordo/Canal

__ Picadero

__ Otro

__ Se rehúsa a contestar

If Q964N is equal to 1 then skip to Q965

If not, then skip to instruction before Q966.

Q965. Por favor especifica el lugar (e.g. el nombre del lugar y las calles que cruzan o la colonia) donde tuviste contacto cercano con [Response to Q899] por última vez.

__ __

If Q964A is equal to 1 then skip to Q966

If not, then skip to instruction before Q967.

Q966. Tú nos dijiste que el último contacto cercano con [Response to Q899] fué en tu casa/casa de alguien más. ¿Dónde se ubica esta casa? ¿Podrías darnos los nombres de las calles que cruzan la intersección más cercana? Entrevistador: Por favor anotar los nombres de las calles que cruzan. Si el participante no puede darnos esta información, pregúntele el nombre de la colonia y anote esa información.

— —

If Q964B is equal to 1 then skip to Q967

If not, then skip to instruction before Q968.

Q967. Tú nos dijiste que el último contacto cercano con [Response to Q899] fué en tu trabajo. ¿Nos podrías dar el nombre y la dirección de tu lugar de trabajo? Entrevistador: Si el lugar de trabajo no tiene nombre o dirección, ingresar cualquier información que se dé para la ubicación (e.g. nombre de calles, intersección más cercana, colonia)

— —

If Q964C is equal to 1 then skip to Q968

If not, then skip to instruction before Q969.

Q968. Tú nos dijiste que la última vez que estuviste en contacto cercano con [Response to Q899] fuéó en un restaurante/café. ¿Podríasdarnos el nombre de este lugar y las calles que cruzan/colonia donde se ubica este restaurante?

— —

If Q964D is equal to 1 then skip to Q969

If not, then skip to instruction before Q970.

Q969. Tú nos dijiste que la última vez que estuviste en contacto cercano con [Response to Q899] fué en un lugar de tiempo libre (e.g. teatro, bar, club nocturno). ¿Podrías darnos el nombre del lugar y las calles que cruzan/colonia donde se ubica este lugar?

— —

If Q964E is equal to 1 then skip to Q970

If not, then skip to instruction before Q971.

Q970. Tú nos dijiste que la última vez que estuviste en contacto cercano con [Response to Q899] fué en tu escuela o lugar de estudio. ¿Podrías darnos el nombre del lugar y las calles que cruzan/colonia donde se ubica este lugar?

__ __

If Q964F is equal to 1 then skip to Q971

If not, then skip to instruction before Q972.

Q971. Tú nos dijiste que la última vez que estuviste en contacto cercano con [Response to Q899] fué en un gimnasio/centro fitness. ¿Podrías darnos el nombre del lugar y las calles que cruzan/colonia donde se ubica este lugar?

__ __

If Q964G is equal to 1 then skip to Q972

If not, then skip to instruction before Q973.

Q972. Tú nos dijiste que la última vez que estuviste en contacto cercano con [Response to Q899] fué en un lugar donde dan servicios personales (e.g. corte de pelo, masaje). ¿Podrías darnos el nombre del lugar y las calles que cruzan/colonia donde se ubica este lugar?

__ __

If Q964H is equal to 1 then skip to Q973

If not, then skip to instruction before Q974.

Q973. Tú nos dijiste que la última vez que estuviste en contacto cercano con [Response to Q899] fué en un lugar de servicio religioso. ¿Podrías darnos el nombre del lugar y las calles que cruzan/colonia donde se ubica este lugar?

__ __

If Q964I is equal to 1 then skip to Q974

If not, then skip to instruction before Q975.

Q974. Tú nos dijiste que la última vez que estuviste en contacto cercano con [Response to Q899] fué en un centro comunitario/de reunión grupal. ¿Podrías darnos el nombre del lugar y las calles que cruzan/colonia donde se ubica este lugar?

— —

If Q964J is equal to 1 then skip to Q975

If not, then skip to instruction before Q976.

Q975. Tú nos dijiste que la última vez que estuviste en contacto cercano con [Response to Q899] fué en un parque. ¿Podrías darnos el nombre del lugar y las calles que cruzan/colonia donde se ubica este lugar?

— —

If Q964K is equal to 1 then skip to Q976

If not, then skip to instruction before Q977.

Q976. Tú nos dijiste que la última vez que estuviste en contacto cercano con [Response to Q899] fué en la calle. ¿Podrías darnos el nombre del lugar y las calles que cruzan/colonia donde se ubica este lugar?

— —

If Q964M is equal to 1 then skip to Q977

If not, then skip to instruction before Q978.

Q977. Tú nos dijiste que la última vez que estuviste en contacto cercano con [Response to Q899] fué en un picadero. ¿En qué picadero fué? (Elija Uno)

01	Las Palmitas/Zona Norte
02	Los Pinos/ Zona Norte
03	Las Bombas/ Zona Norte
04	Los Aragón/ Zona Norte
05	La Veinte/ Bordo, Col. Veinte de Noviembre
06	El Azteca/ Cerro Colorado
07	Oxxo, Calle 5ta Zona Norte
08	Bordo/ Los Álamos, Mundo divertido
09	Cañón Guerrero/Maclovio Herrera Colonia Guerrero
10	Col. Guaycura (casa privada)
11	Col. Presidentes (Infonavit edificio)
12	Florido 1era Sección (casa privada)
13	Florido 1era Sección (Bomberos, conecta)
14	Col. Francisco Villa (edificio abandonado)
15	Cañón del Pato/ Colonia Villa
16	Col. Postal (casa privada)
17	Col. del Río (Parte baja, barranco)
18	Sánchez Taboada (Av. Sánchez Taboada y Parque Industrial)
19	La cima (casa abandonado)
20	Otay módulos
21	Otros
98	Se rehúsa a contestar

If Q977 is equal to 21 then skip to Q978

If not, then skip to instruction before Q979.

Q978. Por favor especifica en cuál picadero fué (e.g. nombre del lugar y calles que cruzan o colonia) el último contacto cercano con [Response to Q899].

____

If Q959 is greater than 1 then skip to Q979

If not, then skip to instruction before Q997.

Q979. Por favor piensa en alguna otra vez que tuviste contacto cercano con [Response to Q899] en los últimos 14 días, aparte del contacto cercano que nos acaba de describir con la persona en cuestión. Cuando decimos alguna otra vez, queremos decir un contacto en el tu viste a [Response to Q899] en persona y luego se separaron, ¿Cuántos dias hace que se vieron?

__ __ DíAS

98 Se rehúsa a contestar (Días)

If Q979 is less than MC2FDAY then READ: "Este tiempo es más reciente que el tiempo que Ud. indicó que tuvo el último contacto. Por favor realice las correcciones necesarias. TRANSLATION" and skip to Q979.

If Q959 is greater than 1 then skip to Q980

If not, then skip to Q981.

Q980. Durante este contacto, ¿Cuántos días consecutivos viste a [Response to Q899]?

__ __

98 Se rehúsa a contestar

Q981. Cuándo viste a [Response to Q896] en esa ocasión, ¿qué tipo de contacto tuviste con él/ella? (Elije todas las que apliquen) (Marque Todas las que Correspondan)

__ Contacto físico, no sexual [e.g. saludo de mano, abrazo, contacto de piel, etc.]

__ Contacto físico, sexual

__ No físico [e.g. conversación en persona, comer/beber, junta]

__ Se rehúsa a contestar

Q982. Cuándo viste a [Response to Q899] en esa ocasión, ¿en cuál de los siguientes lugares fue que tuvieron contacto cercano? (elije todas las que apliquen) (Marque Todas las que Correspondan)

__ En casa/casa de alguien

__ Trabajo

__ Restaurante o café

__ Tiempo Libre (i.e. cine, bar, club nocturno)

__ Escuela/educación

__ Ejercicio o relacionado al estado físico

__ Servicio personal (i.e. corte de cabello, masaje)

__ Servicio religioso

__ Grupo comunitario

__ Parque

__ Calle

__ El Bordo/Canal

__ Picadero

__ Otro

__ Se rehúsa a contestar

If Q982N is equal to 1 then skip to Q983

If not, then skip to instruction before Q984.

Q983. Por favor especifica el lugar (e.g. el nombre del lugar y las calles que cruzan o la colonia) donde tuviste contacto cercano con [Response to Q899] por última vez.

____

If Q982A is equal to 1 then skip to Q984

If not, then skip to instruction before Q985.

Q984. Tú nos dijiste que el último contacto cercano con [Response to Q899] fué en tu casa/casa de alguien más. ¿Dónde se ubica esta casa? ¿Podrías darnos los nombres de las calles que cruzan la intersección más cercana? Entrevistador: Por favor anote los nombres de las calles que lo cruzan. Si el participante no puede darnos esta información, pregúntele el nombre de la colonia y anote esa información.

____

If Q982B is equal to 1 then skip to Q985

If not, then skip to instruction before Q986.

Q985. Tú nos dijiste que el último contacto cercano con [Response to Q899] fué en tu trabajo. ¿Nos podrías dar el nombre y la dirección de tu lugar de trabajo? Entrevistador: Si el lugar de trabajo no tiene nombre o dirección, ingresar cualquier información que se dé para la ubicación (e.g. nombre de calles, intersección más cercana, colonia)

____

If Q982C is equal to 1 then skip to Q986

If not, then skip to instruction before Q987.

Q986. Tú nos dijiste que la última vez que estuviste en contacto cercano con [Response to Q899] fué en un restaurante/café. ¿Podrías darnos el nombre de este lugar y las calles que cruzan/colonia donde se ubica este restaurante?

____

If Q982D is equal to 1 then skip to Q987

If not, then skip to instruction before Q988.

Q987. Tú nos dijiste que la última vez que estuviste en contacto cercano con [Response to Q899] fué en un lugar de tiempo libre (e.g. teatro, bar, club nocturno). ¿Podrías darnos el nombre del lugar y las calles que cruzan/colonia donde se ubica este lugar?

____

If Q982E is equal to 1 then skip to Q988

If not, then skip to instruction before Q989.

Q988. Tú nos dijiste que la última vez que estuviste en contacto cercano con [Response to Q899] fué en tu escuela o lugar de estudio. ¿Podrías darnos el nombre del lugar y las calles que cruzan/colonia donde se ubica este lugar?

__ __

If Q982F is equal to 1 then skip to Q989

If not, then skip to instruction before Q990.

Q989. Tú nos dijiste que la última vez que estuviste en contacto cercano con [Response to Q899] fué en gimnasio/centro fitness. ¿Podrías darnos el nombre del lugar y las calles que cruzan/colonia donde se ubica este lugar?

__ __

If Q982G is equal to 1 then skip to Q990

If not, then skip to instruction before Q991.

Q990. Tú nos dijiste que la última vez que estuviste en contacto cercano con [Response to Q899] fué en un lugar donde dan servicios personales (e.g. corte de pelo, masaje). ¿Podrías darnos el nombre del lugar y las calles que cruzan/colonia donde se ubica este lugar?

__ __

If Q982H is equal to 1 then skip to Q991

If not, then skip to instruction before Q992.

Q991. Tú nos dijiste que la última vez que estuviste en contacto cercano con [Response to Q899] fué en un lugar de servicio religioso. ¿Podrías darnos el nombre del lugar y las calles que cruzan/colonia donde se ubica este lugar?

__ __

If Q982I is equal to 1 then skip to Q992

If not, then skip to instruction before Q993.

Q992. Tú nos dijiste que la última vez que estuviste en contacto cercano con [Response to Q899] fué en un centro comunitario/de reunión grupal. ¿Podrías darnos el nombre del lugar y las calles que cruzan/colonia donde se ubica este lugar?

— —
— — — — — — — — — — — — — —

If Q982J is equal to 1 then skip to Q993

If not, then skip to instruction before Q994.

Q993. Tú nos dijiste que la última vez que estuviste en contacto cercano con [Response to Q899] fué en un parque. ¿Podrías darnos el nombre del lugar y las calles que cruzan/colonia donde se ubica este lugar?

— —
— — — — — — — — — — — — — —

If Q982K is equal to 1 then skip to Q994

If not, then skip to instruction before Q995.

Q994. Tú nos dijiste que la última vez que estuviste en contacto cercano con [Response to Q899] fué en la calle. ¿Podrías darnos el nombre del lugar y las calles que cruzan/colonia donde se ubica este lugar?

— —
— — — — — — — — — — — — — —

If Q982M is equal to 1 then skip to Q995

If not, then skip to instruction before Q996.

Q995. Tú nos dijiste que la última vez que estuviste en contacto cercano con [Response to Q899] fué en un picadero. ¿En qué picadero fué? (Elija Uno)

01	Las Palmitas/Zona Norte
02	Los Pinos/ Zona Norte
03	Las Bombas/ Zona Norte
04	Los Aragón/ Zona Norte
05	La Veinte/ Bordo, Col. Veinte de Noviembre
06	El Azteca/ Cerro Colorado
07	Oxxo, Calle 5ta Zona Norte
08	Bordo/ Los Álamos, Mundo divertido
09	Cañón Guerrero/Maclovio Herrera Colonia Guerrero
10	Col. Guaycura (casa privada)
11	Col. Presidentes (Infonavit edificio)
12	Florido 1era Sección (casa privada)
13	Florido 1era Sección (Bomberos, conecta)
14	Col. Francisco Villa (edificio abandonado)
15	Cañón del Pato/ Colonia Villa
16	Col. Postal (casa privada)
17	Col. del Río (Parte baja, barranco)
18	Sánchez Taboada (Av. Sánchez Taboada y Parque Industrial)
19	La cima (casa abandonado)
20	Otay módulos
21	Otro
98	Se rehúsa a contestar

If Q995 is equal to 21 then skip to Q996

If not, then skip to instruction before Q997.

Q996. Por favor especifica en qué picadero (e.g. nombre del lugar y calles que cruzan o colonia) fué el último contacto cercano con [Response to Q899].

____

If Q686 + Q707 + Q728 + Q749 + Q770 + Q791 + Q812 + Q833 + Q854 + Q875 is greater than 2 and Q902 is not equal to "Se rehúsa a contestar" and Q902 is not equal to "Saltó" then skip to Q997

If not, then skip to instruction before Q1040.

Q997. ¿Es [Response to Q902] [MCL3] ([MCN3]) hombre, mujer, transgénero, no-binario u otro? (Elija Uno)

1	Hombre
2	Mujer
3	Mujer Trans
4	Hombre Trans
5	No-binario/ genero no-binario / género fluido/ agénero
6	Otra identidad no en la lista
8	Se rehúsa a contestar

If Q997 is equal to 6 then skip to Q998

If not, then skip to Q999.

Q998. Por favor especifica la identidad de género de [Response to Q902].

____

Q999. ¿Dónde vive [Response to Q902] principalmente? (Elija Uno)

1 Mexico

2 Estados Unidos

3 Otro

7 No Sabe

8 Se rehúsa a contestar

If Q999 is equal to 3 then skip to Q1000

If not, then skip to Q1001.

Q1000. Por favor especifica donde vive principalmente [Response to Q902].

____

Q1001. ¿Conociste a [Response to Q902] por primera vez en los últimos 14 días?

1 Si

0 No

8 Se rehúsa a contestar

Q1002. ¿Cuántas veces has tenido contacto cercano (dentro de 2 metros) con [Response to Q902] en los últimos 14 días?

__ __

98 Se rehúsa a contestar

Q1003. En promedio, durante los días que viste a [Response to Q902], ¿Cuánto tiempo (e.g. horas, minuto) duraste en contacto cercano con él/ella? Entrevistador: el tiempo máximo por día es 24 horas.

__ __ HOURS

__ __ MINUTES

98 Se rehúsa a contestar (Hours)

Q1004. ¿Cuándo fue la última vez que tuviste contacto cercano con [Response to Q902] en los últimos 14 días? Entrevistador: Anote cuántos días hace que el/la participante tuvo contacto cercano por última vez con la persona en cuestión. Si la última vez fue hoy, ingresa 0.

__ __ DíAS

98 Se rehúsa a contestar (Días)

If Q1002 is greater than 1 then skip to Q1005

If not, then skip to Q1006.

Q1005. Durante el último contacto, ¿Por cuántos días consecutivos viste a [Response to Q902]?

__ __

98 Se rehúsa a contestar

MC3FDAY = If(Q1004 is equal to 1, Q1004, If(Q1004 is not equal to 98 and Q1004 is greater than 1, Q1004 + Q1005, "Falta"))

Q1006. Cuando viste a [Response to Q902], ¿Qué tipo de contacto tuviste con esta persona (por favor escoge todas las que apliquen)? (Marque Todas las que Correspondan)

__ Contacto físico, no sexual [e.g. saludo de mano, abrazo, contacto de piel, etc.]

__ Contacto físico, sexual

__ No físico [e.g. conversación en persona, comer/beber, junta]

__ Se rehúsa a contestar

Q1007. Cuando viste a [Response to Q902] durante el contacto, ¿en cuál de los siguientes lugares fué? (por favor escoge todas las que apliquen) (Marque Todas las que Correspondan)

__ En casa/casa de alguien

__ Trabajo

__ Restaurante o café

__ Tiempo Libre (i.e. cine, bar, club nocturno)

__ Escuela/educación

__ Ejercicio o relacionado al estado físico

__ Servicio personal (i.e. corte de cabello, masaje)

__ Servicio religioso

__ Grupo comunitario

__ Parque

__ Calle

__ El Bordo/Canal

__ Picadero

__ Otro

__ Se rehúsa a contestar

If Q1007N is equal to 1 then skip to Q1008

If not, then skip to instruction before Q1009.

Q1008. Por favor especifica el lugar (e.g. el nombre del lugar y las calles que cruzan o la colonia) donde tuviste contacto cercano con [Response to Q902] por última vez.

____

If Q1007A is equal to 1 then skip to Q1009

If not, then skip to instruction before Q1010.

Q1009. Tú nos dijiste que el último contacto cercano con [Response to Q902] fué en tu casa/casa de alguien más. ¿Dónde se ubica esta casa? ¿Podrías darnos los nombres de las calles que cruzan la intersección más cercana? Entrevistador: Por favor anote los nombres de las calles que cruzan. Si el participante no puede darnos esta información, pregúntele el nombre de la colonia y anote esa información.

— —

If Q1007B is equal to 1 then skip to Q1010

If not, then skip to instruction before Q1011.

Q1010. Tú nos dijiste que el último contacto cercano con [Response to Q902] fué en tu trabajo. ¿Nos podrías dar el nombre y la dirección de tu lugar de trabajo? Entrevistador: Si el lugar de trabajo no tiene nombre o dirección, anota cualquier información que se dé para la ubicación (e.g. nombre de calles, intersección más cercana, colonia)

— —

If Q1007C is equal to 1 then skip to Q1011

If not, then skip to instruction before Q1012.

Q1011.

Tú nos dijiste que la última vez que estuviste en contacto cercano con [Response to Q902] fué en un restaurante/café. ¿Podrías darnos el nombre de este lugar y las calles que cruzan/colonia donde se ubica este restaurante?

— —

If Q1007D is equal to 1 then skip to Q1012

If not, then skip to instruction before Q1013.

Q1012. Tú nos dijiste que la última vez que estuviste en contacto cercano con [Response to Q902] fué en un lugar de tiempo libre (e.g. teatro, bar, club nocturno). ¿Podrías darnos el nombre del lugar y las calles que cruzan/colonia donde se ubica este lugar?

— —

If Q1007E is equal to 1 then skip to Q1013

If not, then skip to instruction before Q1014.

Q1013. Tú nos dijiste que la última vez que estuviste en contacto cercano con [Response to Q902] fué en tu escuela o lugar de estudio. ¿Podrías darnos el nombre del lugar y las calles que cruzan/colonia donde se ubica este lugar?

— —

If Q1007F is equal to 1 then skip to Q1014

If not, then skip to instruction before Q1015.

Q1014. Tú nos dijiste que la última vez que estuviste en contacto cercano con [Response to Q902] fué en un gimnasio/centro fitness. ¿Podrías darnos el nombre del lugar y las calles que cruzan/colonia donde se ubica este lugar?

— —

If Q1007G is equal to 1 then skip to Q1015

If not, then skip to instruction before Q1016.

Q1015. Tú nos dijiste que la última vez que estuviste en contacto cercano con [Response to Q902] fué en un lugar donde dan servicios personales (e.g. corte de pelo, masaje). ¿Podrías darnos el nombre del lugar y las calles que cruzan/colonia donde se ubica este lugar?

— —

If Q1007H is equal to 1 then skip to Q1016

If not, then skip to instruction before Q1017.

Q1016. Tú nos dijiste que la última vez que estuviste en contacto cercano con [Response to Q902] fué en un lugar de servicio religioso. ¿Podrías darnos el nombre del lugar y las calles que cruzan/colonia donde se ubica este lugar?

— —

If Q1007I is equal to 1 then skip to Q1017

If not, then skip to instruction before Q1018.

Q1017. Tú nos dijiste que la última vez que estuviste en contacto cercano con [Response to Q902] fué en un centro comunitario/de reunión grupal. ¿Podrías darnos el nombre del lugar y las calles que cruzan/colonia donde se ubica este lugar?

__ __

If Q1007J is equal to 1 then skip to Q1018

If not, then skip to instruction before Q1019.

Q1018. Tú nos dijiste que la última vez que estuviste en contacto cercano con [Response to Q902] fué en un parque. ¿Podrías darnos el nombre del lugar y las calles que cruzan/colonia donde se ubica este lugar?

__ __

If Q1007K is equal to 1 then skip to Q1019

If not, then skip to instruction before Q1020.

Q1019. Tú nos dijiste que la última vez que estuviste en contacto cercano con [Response to Q902] fué en la calle. ¿Podrías darnos el nombre del lugar y las calles que cruzan/colonia donde se ubica este lugar?

__ __

If Q1007M is equal to 1 then skip to Q1020

If not, then skip to instruction before Q1021.

Q1020. Tú nos dijiste que la última vez que estuviste en contacto cercano con [Response to Q902] fué en un picadero. ¿En qué picadero fué? (Elija Uno)

01	Las Palmitas/Zona Norte
02	Los Pinos/ Zona Norte
03	Las Bombas/ Zona Norte
04	Los Aragón/ Zona Norte
05	La Veinte/ Bordo, Col. Veinte de Noviembre
06	El Azteca/ Cerro Colorado
07	Oxxo, Calle 5ta Zona Norte
08	Bordo/ Los Álamos, Mundo divertido
09	Cañón Guerrero/Maclovio Herrera Colonia Guerrero
10	Col. Guaycura (casa privada)
11	Col. Presidentes (Infonavit edificio)
12	Florido 1era Sección (casa privada)
13	Florido 1era Sección (Bomberos, conecta)
14	Col. Francisco Villa (edificio abandonado)
15	Cañón del Pato/ Colonia Villa
16	Col. Postal (casa privada)
17	Col. del Río (Parte baja, barranco)
18	Sánchez Taboada (Av. Sánchez Taboada y Parque Industrial)
19	La cima (casa abandonado)
20	Otay módulos
21	Otro
98	Se rehúsa a contestar

If Q1020 is equal to 21 then skip to Q1021

If not, then skip to instruction before Q1022.

Q1021. Por favor especifica en qué picadero (e.g. nombre del lugar y calles que cruzan o colonia) fué el último contacto cercano con [Response to Q902].

____

If Q1002 is greater than 1 then skip to Q1022

If not, then skip to instruction before Q1040.

Q1022. Por favor piensa en alguna otra vez que tuviste contacto cercano con [Response to Q902] en los últimos 14 días, aparte del contacto cercano que nos acaba de describir con la persona en cuestión. Cuando decimos alguna otra vez, queremos decir un contacto en el tu viste a [Response to Q902] en persona y luego se separaron, ¿Cuántos dias hace que se vieron?

__ __ DíAS

98 Se rehúsa a contestar (Días)

If Q1022 is less than MC3FDAY then READ: "Este tiempo es más reciente que el tiempo que Ud. indicó que tuvo el último contacto. Por favor realice las correcciones necesarias. TRANSLATION" and skip to Q1022.

If Q1002 is greater than 1 then skip to Q1023

If not, then skip to Q1024.

Q1023. Durante este contacto, ¿Cuántos días consecutivos viste a [Response to Q902]?

__ __

98 Se rehúsa a contestar

Q1024. Cuándo viste a [Response to Q902] en esa ocasión, ¿qué tipo de contacto tuviste con él/ella? (Elije todas las que apliquen) (Marque Todas las que Correspondan)

__ Contacto físico, no sexual [e.g. saludo de mano, abrazo, contacto de piel, etc.]

__ Contacto físico, sexual

__ No físico [e.g. conversación en persona, comer/beber, junta]

__ Se rehúsa a contestar

Q1025. Cuándo viste a [Response to Q902] en esa ocasión, ¿en cuál de los siguientes lugares fue que tuvieron contacto cercano? (elije todas las que apliquen) (Marque Todas las que Correspondan)

__ En casa/casa de alguien

__ Trabajo

__ Restaurante o Café

__ Tiempo Libre (i.e. cine, bar, club nocturno)

__ Escuela/educación

__ Ejercicio o relacionado al estado físico

__ Servicio personal (i.e. corte de cabello, masaje)

__ Servicio religioso

__ Grupo comunitario

__ Parque

__ Calle

__ El Bordo/Canal

__ Picadero

__ Otro

__ Se rehúsa a contestar

If Q1025N is equal to 1 then skip to Q1026

If not, then skip to instruction before Q1027.

Q1026. Por favor especifica el lugar (e.g. nombre del lugar y las calles que cruzan o la colonia) donde fué ese contacto cercano con [Response to Q902].

__ __

If Q1025A is equal to 1 then skip to Q1027

If not, then skip to instruction before Q1028.

Q1027. Tú nos dijiste que el último contacto cercano con [Response to Q902] fué en tu casa/casa de alguien más. ¿Dónde se ubica esta casa? ¿Podrías darnos los nombres de las calles que cruzan la intersección más cercana? Entrevistador: Por favor anote los nombres de las calles que lo cruzan. Si el participante no puede darnos esta información, preguntarles el nombre de la colonia y anote esa información.

— —

If Q1025B is equal to 1 then skip to Q1028

If not, then skip to instruction before Q1029.

Q1028. Tú nos dijiste que el último contacto cercano con [Response to Q902] fué en tu trabajo. ¿Nos podrías dar el nombre y la dirección de tu lugar de trabajo? Entrevistador: Si el lugar de trabajo no tiene nombre o dirección,anote cualquier información que se dé para la ubicación (e.g. nombre de calles, intersección más cercana, colonia)

— —

If Q1025C is equal to 1 then skip to Q1029

If not, then skip to instruction before Q1030.

Q1029. Tú nos dijiste que la última vez que estuviste en contacto cercano con [Response to Q902] fué en un restaurante/café. ¿Podrías darnos el nombre de este lugar y las calles que cruzan/colonia donde se ubica este restaurante?

— —

If Q1025D is equal to 1 then skip to Q1030

If not, then skip to instruction before Q1031.

Q1030. Tú nos dijiste que la última vez que estuviste en contacto cercano con [Response to Q902] fué en un lugar de tiempo libre (e.g. teatro, bar, club nocturno). ¿Podrías darnos el nombre del lugar y las calles que cruzan/colonia donde se ubica este lugar?

— —

If Q1025E is equal to 1 then skip to Q1031

If not, then skip to instruction before Q1032.

Q1031. Tú nos dijiste que la última vez que estuviste en contacto cercano con [Response to Q902] fué en tu escuela o lugar de estudio. ¿Podrías darnos el nombre del lugar y las calles que cruzan/colonia donde se ubica este lugar?

— —

If Q1025F is equal to 1 then skip to Q1032

If not, then skip to instruction before Q1033.

Q1032. Tú nos dijiste que la última vez que estuviste en contacto cercano con [Response to Q902] fué en un gimnasio/centro fitness. ¿Podrías darnos el nombre del lugar y las calles que cruzan/colonia donde se ubica este lugar?

— —

If Q1025G is equal to 1 then skip to Q1033

If not, then skip to instruction before Q1034.

Q1033. Tú nos dijiste que la última vez que estuviste en contacto cercano con [Response to Q902] fué en un lugar donde dan servicios personales (e.g. corte de pelo, masaje). ¿Podrías darnos el nombre del lugar y las calles que cruzan/colonia donde se ubica este lugar?

— —

If Q1025H is equal to 1 then skip to Q1034

If not, then skip to instruction before Q1035.

Q1034. Tú nos dijiste que la última vez que estuviste en contacto cercano con [Response to Q902] fué en un lugar de servicio religioso. ¿Podrías darnos el nombre del lugar y las calles que cruzan/colonia donde se ubica este lugar?

— —

If Q1025I is equal to 1 then skip to Q1035

If not, then skip to instruction before Q1036.

Q1035. Tú nos dijiste que la última vez que estuviste en contacto cercano con [Response to Q902] fué en un centro comunitario/de reunión grupal. ¿Podrías darnos el nombre del lugar y las calles que cruzan/colonia donde se ubica este lugar?

— —

If Q1025J is equal to 1 then skip to Q1036

If not, then skip to instruction before Q1037.

Q1036. Tú nos dijiste que la última vez que estuviste en contacto cercano con [Response to Q902] fué en un parque. ¿Podrías darnos el nombre del lugar y las calles que cruzan/colonia donde se ubica este lugar?

— —

If Q1025K is equal to 1 then skip to Q1037

If not, then skip to instruction before Q1038.

Q1037. Tú nos dijiste que la última vez que estuviste en contacto cercano con [Response to Q902] fué en la calle. ¿Podrías darnos el nombre del lugar y las calles que cruzan/colonia donde se ubica este lugar?

— —

If Q1025M is equal to 1 then skip to Q1038

If not, then skip to instruction before Q1039.

Q1038. Tú nos dijiste que la última vez que estuviste en contacto cercano con [Response to Q902] fué en un picadero. ¿En qué picadero fué? (Elija Uno)

01	Las Palmitas/Zona Norte
02	Los Pinos/ Zona Norte
03	Las Bombas/ Zona Norte
04	Los Aragón/ Zona Norte
05	La Veinte/ Bordo, Col. Veinte de Noviembre
06	El Azteca/ Cerro Colorado
07	Oxxo, Calle 5ta Zona Norte
08	Bordo/ Los Álamos, Mundo divertido
09	Cañón Guerrero/Maclovio Herrera Colonia Guerrero
10	Col. Guaycura (casa privada)
11	Col. Presidentes (Infonavit edificio)
12	Florido 1era Sección (casa privada)
13	Florido 1era Sección (Bomberos, conecta)
14	Col. Francisco Villa (edificio abandonado)
15	Cañón del Pato/ Colonia Villa
16	Col. Postal (casa privada)
17	Col. del Río (Parte baja, barranco)
18	Sánchez Taboada (Av. Sánchez Taboada y Parque Industrial)
19	La cima (casa abandonado)
20	Otay módulos
21	Otro
98	Se rehúsa a contestar

If Q1038 is equal to 21 then skip to Q1039

If not, then skip to instruction before Q1040.

Q1039. Por favor especifica en qué picadero (e.g. nombre del lugar y calles que cruzan o colonia) fué el último contacto cercano con [Response to Q902].

____

If Q686 + Q707 + Q728 + Q749 + Q770 + Q791 + Q812 + Q833 + Q854 + Q875 is greater than 3 and Q905 is not equal to "Se rehúsa a contestar" and Q905 is not equal to "Saltó" then skip to Q1040

If not, then skip to instruction before Q1083.

Q1040. ¿Es [Response to Q905] [Response to Q906] ([Response to Q907]) hombre, mujer, transgénero, no-binario u otro? (Elija Uno)

1 Hombre

2 Mujer

3 Mujer Trans

4 Hombre Trans

5 No-binario/ genero no-binario / género fluido/ agénero

6 Otra identidad no en la lista

8 Se rehúsa a contestar

If Q1040 is equal to 6 then skip to Q1041

If not, then skip to Q1042.

Q1041. Por favor especifica la identidad de género de [Response to Q905].

____

Q1042. ¿Dónde vive [Response to Q905] principalmente? (Elija Uno)

1	Mexico
2	Estado Unidos
3	Otro
7	No Sabe
8	Se rehúsa a contestar

If Q1042 is equal to 3 then skip to Q1043

If not, then skip to Q1044.

Q1043. Por favor especifica donde vive principalmente [Response to Q905].

__ __

Q1044. ¿Conociste a [Response to Q905] por primera vez en los últimos 14 días?

1	Si
0	No
8	Se rehúsa a contestar

Q1045. ¿Cuántas veces has tenido contacto cercano (dentro de 2 metros) con [Response to Q905] en los últimos 14 días?

__ __	
98	Se rehúsa a contestar

Q1046. En promedio, durante los días que viste a [Response to Q905], ¿Cuánto tiempo (e.g. horas, minuto) duraste en contacto cercano con él/ella? Entrevistador: el tiempo máximo por día es 24 horas.

__ __	HOURS
__ __	MINUTES
98	Se rehúsa a contestar (Hours)

Q1047. ¿Cuándo fue la última vez que tuviste contacto cercano con [Response to Q905] en los últimos 14 días? Entrevistador: Anote cuántos días hace que el/la participante tuvo contacto cercano por última vez con la persona en cuestión. Si la última vez fue hoy, ingresa 0.

__ __ DíAS

98 Se rehúsa a contestar (Días)

If Q1045 is greater than 1 then skip to Q1048

If not, then skip to Q1049.

Q1048. Durante el último contacto, ¿Por cuantos días consecutivos viste a [Response to Q905]?

__ __

98 Se rehúsa a contestar

MC4FDAY = If(Q1047 is equal to 1, Q1047, If(Q1047 is not equal to 98 and Q1047 is greater than 1, Q1047 + Q1048, "Falta"))

Q1049. La última vez que viste a [Response to Q905], ¿Qué tipo de contacto tuviste con esta persona? (elija todas las que apliquen) (Marque Todas las que Correspondan)

__ Contacto físico, no sexual [e.g. saludo de mano, abrazo, contacto de piel, etc.]

__ Contacto físico, sexual

__ No físico [e.g. conversación en persona, comer/beber, junta]

__ Se rehúsa a contestar

Q1050. Cuando viste a [Response to Q905] durante el contacto, ¿en cual de los siguientes lugares ocurrió? (por favor escoge todas las que apliquen) (Marque Todas las que Correspondan)

__ En casa/casa de alguien

__ Trabajo

__ Restaurante o Café

__ Tiempo Libre (i.e. cine, bar, club nocturno)

__ Escuela/educación

__ Ejercicio o relacionado al estado físico

__ Servicio personal (i.e. corte de cabello, masaje)

__ Servicio religioso

__ Grupo comunitario

__ Parque

__ Calle

__ El Bordo/Canal

__ Picadero

__ Otro

__ Se rehúsa a contestar

If Q1050N is equal to 1 then skip to Q1051

If not, then skip to instruction before Q1052.

Q1051. Por favor especifica el lugar (e.g. el nombre del lugar y las calles que cruzan o la colonia) donde tuviste contacto cercano con [Response to Q905] por última vez.

____

If Q1050A is equal to 1 then skip to Q1052

If not, then skip to instruction before Q1053.

Q1052. Tú nos dijiste que el último contacto cercano con [Response to Q905] fué en tu casa/la casa de alguien más. ¿Dónde se ubica esta casa? ¿Podrías darnos los nombres de las calles que cruzan la intersección más cercana? Entrevistador: Por favor anote los nombres de las calles que lo cruzan. Si el participante no puede darnos esta información, pregúntele el nombre de la colonia y anote esa información.

— —

If Q1050B is equal to 1 then skip to Q1053

If not, then skip to instruction before Q1054.

Q1053. Tú nos dijiste que el último contacto cercano con [Response to Q905] fué en tu trabajo. ¿Nos podrías dar el nombre y la dirección de tu lugar de trabajo? Entrevistador: Si el lugar de trabajo no tiene nombre o dirección, anote cualquier información que se dé para la ubicación (e.g. nombre de calles, intersección más cercana, colonia)

— —

If Q1050C is equal to 1 then skip to Q1054

If not, then skip to instruction before Q1055.

Q1054. Tú nos dijiste que la última vez que estuviste en contacto cercano con [Response to Q905] fué en un restaurante/café. ¿Podrías darnos el nombre de este lugar y las calles que cruzan/colonia donde se ubica este restaurante?

— —

If Q1050D is equal to 1 then skip to Q1055

If not, then skip to instruction before Q1056.

Q1055. Tú nos dijiste que la última vez que estuviste en contacto cercano con [Response to Q905] fué en un lugar de tiempo libre (e.g. teatro, bar, club nocturno). ¿Podrías darnos el nombre del lugar y las calles que cruzan/colonia donde se ubica este lugar?

— —

If Q1050E is equal to 1 then skip to Q1056

If not, then skip to instruction before Q1057.

Q1056. Tú nos dijiste que la última vez que estuviste en contacto cercano con [Response to Q905] fué en tu escuela o lugar de estudio. ¿Podrías darnos el nombre del lugar y las calles que cruzan/colonia donde se ubica este lugar?

— —

If Q1050F is equal to 1 then skip to Q1057

If not, then skip to instruction before Q1058.

Q1057. Tú nos dijiste que la última vez que estuviste en contacto cercano con [Response to Q905] fué en un gimnasio/centro fitness. ¿Podrías darnos el nombre del lugar y las calles que cruzan/colonia donde se ubica este lugar?

— —

If Q1050G is equal to 1 then skip to Q1058

If not, then skip to instruction before Q1059.

Q1058. Tú nos dijiste que la última vez que estuviste en contacto cercano con [Response to Q905] fué en un lugar donde dan servicios personales (e.g. corte de pelo, masaje). ¿Podrías darnos el nombre del lugar y las calles que cruzan/colonia donde se ubica este lugar?

— —

If Q1050H is equal to 1 then skip to Q1059

If not, then skip to instruction before Q1060.

Q1059. Tú nos dijiste que la última vez que estuviste en contacto cercano con [Response to Q905] fué en un lugar de servicio religioso. ¿Podrías darnos el nombre del lugar y las calles que cruzan/colonia donde se ubica este lugar?

— —

If Q1050I is equal to 1 then skip to Q1060

If not, then skip to instruction before Q1061.

Q1060. Tú nos dijiste que la última vez que estuviste en contacto cercano con [Response to Q905] fué en un centro comunitario/de reunión grupal. ¿Podrías darnos el nombre del lugar y las calles que cruzan/colonia donde se ubica este lugar?

__ __
__ __ __ __ __ __ __ __ __ __ __ __ __ __

If Q1050J is equal to 1 then skip to Q1061

If not, then skip to instruction before Q1062.

Q1061. Tú nos dijiste que la última vez que estuviste en contacto cercano con [Response to Q905] fué en un parque. ¿Podrías darnos el nombre del lugar y las calles que cruzan/colonia donde se ubica este lugar?

__ __
__ __ __ __ __ __ __ __ __ __ __ __ __ __

If Q1050K is equal to 1 then skip to Q1062

If not, then skip to instruction before Q1063.

Q1062. Tú nos dijiste que la última vez que estuviste en contacto cercano con [Response to Q905] fué en la calle. ¿Podrías darnos el nombre del lugar y las calles que cruzan/colonia donde se ubica este lugar?

__ __
__ __ __ __ __ __ __ __ __ __ __ __ __ __

If Q1050M is equal to 1 then skip to Q1063

If not, then skip to instruction before Q1064.

Q1063. Tú nos dijiste que la última vez que estuviste en contacto cercano con [Response to Q905] fué en un picadero. ¿En qué picadero fué? (Elija Uno)

01	Las Palmitas/Zona Norte
02	Los Pinos/ Zona Norte
03	Las Bombas/ Zona Norte
04	Los Aragón/ Zona Norte
05	La Veinte/ Bordo, Col. Veinte de Noviembre
06	El Azteca/ Cerro Colorado
07	Oxxo, Calle 5ta Zona Norte
08	Bordo/ Los Álamos, Mundo divertido
09	Cañón Guerrero/Maclovio Herrera Colonia Guerrero
10	Col. Guaycura (casa privada)
11	Col. Presidentes (Infonavit edificio)
12	Florido 1era Sección (casa privada)
13	Florido 1era Sección (Bomberos, conecta)
14	Col. Francisco Villa (edificio abandonado)
15	Cañón del Pato/ Colonia Villa
16	Col. Postal (casa privada)
17	Col. del Río (Parte baja, barranco)
18	Sánchez Taboada (Av. Sánchez Taboada y Parque Industrial)
19	La cima (casa abandonado)
20	Otay módulos
21	Other
98	Se rehúsa a contestar

If Q1063 is equal to 21 then skip to Q1064

If not, then skip to instruction before Q1065.

Q1064. Por favor especifica en qué picadero (e.g. nombre del lugar y calles que cruzan o colonia) fué el último contacto cercano con [Response to Q905].

____

If Q1045 is greater than 1 then skip to Q1065

If not, then skip to instruction before Q1083.

Q1065. Por favor piensa en alguna otra vez que tuviste contacto cercano con [Response to Q905] en los últimos 14 días, aparte del contacto cercano que nos acaba de describir con la persona en cuestión. Cuando decimos alguna otra vez, queremos decir un contacto en el tu viste a [Response to Q905] en persona y luego se separaron, ¿Cuántos dias hace que se vieron?

__ __ DíAS

98 Se rehúsa a contestar (Días)

If Q1065 is less than MC4FDAY then READ: "Este tiempo es más reciente que el tiempo que Ud. indicó que tuvo el último contacto. Por favor realice las correcciones necesarias. TRANSLATION" and skip to Q1065.

If Q1045 is greater than 1 then skip to Q1066

If not, then skip to Q1067.

Q1066. Durante este contacto, ¿Cuántos días consecutivos viste a [Response to Q905]?

__ __

98 Se rehúsa a contestar

Q1067. Cuándo viste a [Response to Q905] en esa ocasión, ¿qué tipo de contacto tuviste con él/ella? (Elije todas las que apliquen) (Marque Todas las que Correspondan)

__ Contacto físico, no sexual [e.g. saludo de mano, abrazo, contacto de piel, etc.]

__ Contacto físico, sexual

__ No físico [e.g. conversación en persona, comer/beber, junta]

__ Se rehúsa a contestar

Q1068. Cuándo viste a [Response to Q905] en esa ocasión, ¿en cuál de los siguientes lugares fue que tuvieron contacto cercano? (elije todas las que apliquen) (Marque Todas las que Correspondan)

__ En casa/casa de alguien

__ Trabajo

__ Restaurante o Café

__ Tiempo Libre (i.e. cine, bar, club nocturno)

__ Escuela/educación

__ Ejercicio o relacionado al estado físico

__ Servicio personal (i.e. corte de cabello, masaje)

__ Servicio religioso

__ Grupo comunitario

__ Parque

__ Calle

__ El Bordo/Canal

__ Picadero

__ Otro

__ Se rehúsa a contestar

If Q1068N is equal to 1 then skip to Q1069

If not, then skip to instruction before Q1070.

Q1069. Por favor especifica el lugar (e.g. nombre del lugar y las calles que cruzan o la colonia) donde fué ese contacto cercano con [Response to Q905].

____

If Q1068A is equal to 1 then skip to Q1070

If not, then skip to instruction before Q1071.

Q1070. Tú nos dijiste que el último contacto cercano con [Response to Q905] fué en tu casa/la casa de alguien más. ¿Dónde se ubica esta casa? ¿Podrías darnos los nombres de las calles que cruzan la intersección más cercana? Entrevistador: Por favor anote los nombres de las calles que cruzan. Si el participante no puede darnos esta información, pregúntele el nombre de la colonia y anote esa información.

__ __

If Q1068B is equal to 1 then skip to Q1071

If not, then skip to instruction before Q1072.

Q1071. Tú nos dijiste que el último contacto cercano con [Response to Q905] fué en tu trabajo. ¿Nos podrías dar el nombre y la dirección de tu lugar de trabajo? Entrevistador: Si el lugar de trabajo no tiene nombre o dirección, anote cualquier información que se dé para la ubicación (e.g. nombre de calles, intersección más cercana, colonia)

__ __

If Q1068C is equal to 1 then skip to Q1072

If not, then skip to instruction before Q1073.

Q1072. Tú nos dijiste que la última vez que estuviste en contacto cercano con [Response to Q905] fué en un restaurante/café. ¿Podrías darnos el nombre de este lugar y las calles que cruzan/colonia donde se ubica este restaurante?

__ __

If Q1068D is equal to 1 then skip to Q1073

If not, then skip to instruction before Q1074.

Q1073. Tú nos dijiste que la última vez que estuviste en contacto cercano con [Response to Q905] fué en un lugar de tiempo libre (e.g. teatro, bar, club nocturno). ¿Podrías darnos el nombre del lugar y las calles que cruzan/colonia donde se ubica este lugar?

__ __

If Q1068E is equal to 1 then skip to Q1074

If not, then skip to instruction before Q1075.

Q1074. Tú nos dijiste que la última vez que estuviste en contacto cercano con [Response to Q905] fué en tu escuela o lugar de estudio. ¿Podrías darnos el nombre del lugar y las calles que cruzan/colonia donde se ubica este lugar?

— —

If Q1068F is equal to 1 then skip to Q1075

If not, then skip to instruction before Q1076.

Q1075. Tú nos dijiste que la última vez que estuviste en contacto cercano con [Response to Q905] fué en un gimnasio/centro fitness. ¿Podrías darnos el nombre del lugar y las calles que cruzan/colonia donde se ubica este lugar?

— —

If Q1068G is equal to 1 then skip to Q1076

If not, then skip to instruction before Q1077.

Q1076. Tú nos dijiste que la última vez que estuviste en contacto cercano con [Response to Q905] fué en un lugar donde dan servicios personales (e.g. corte de pelo, masaje). ¿Podrías darnos el nombre del lugar y las calles que cruzan/colonia donde se ubica este lugar?

— —

If Q1068H is equal to 1 then skip to Q1077

If not, then skip to instruction before Q1078.

Q1077. Tú nos dijiste que la última vez que estuviste en contacto cercano con [Response to Q905] fué en un lugar de servicio religioso. ¿Podrías darnos el nombre del lugar y las calles que cruzan/colonia donde se ubica este lugar?

— —

If Q1068I is equal to 1 then skip to Q1078

If not, then skip to instruction before Q1079.

Q1078. Tú nos dijiste que la última vez que estuviste en contacto cercano con [Response to Q905] ocurrió en un centro comunitario/de reunión grupal. ¿Podrías darnos el nombre del lugar y las calles que cruzan/colonia donde se ubica este lugar?

— —

If Q1068J is equal to 1 then skip to Q1079

If not, then skip to instruction before Q1080.

Q1079. Tú nos dijiste que la última vez que estuviste en contacto cercano con [Response to Q905] fué en un parque. ¿Podrías darnos el nombre del lugar y las calles que cruzan/colonia donde se ubica este lugar?

— —

If Q1068K is equal to 1 then skip to Q1080

If not, then skip to instruction before Q1081.

Q1080. Tú nos dijiste que la última vez que estuviste en contacto cercano con [Response to Q905] fué en la calle. ¿Podrías darnos el nombre del lugar y las calles que cruzan/colonia donde se ubica este lugar?

— —

If Q1068M is equal to 1 then skip to Q1081

If not, then skip to instruction before Q1082.

Q1081. Tú nos dijiste que la última vez que estuviste en contacto cercano con [Response to Q905] fué en un picadero. ¿En qué picadero fué? (Elija Uno)

01	Las Palmitas/Zona Norte
02	Los Pinos/ Zona Norte
03	Las Bombas/ Zona Norte
04	Los Aragón/ Zona Norte
05	La Veinte/ Bordo, Col. Veinte de Noviembre
06	El Azteca/ Cerro Colorado
07	Oxxo, Calle 5ta Zona Norte
08	Bordo/ Los Álamos, Mundo divertido
09	Cañón Guerrero/Maclovio Herrera Colonia Guerrero
10	Col. Guaycura (casa privada)
11	Col. Presidentes (Infonavit edificio)
12	Florido 1era Sección (casa privada)
13	Florido 1era Sección (Bomberos, conecta)
14	Col. Francisco Villa (edificio abandonado)
15	Cañón del Pato/ Colonia Villa
16	Col. Postal (casa privada)
17	Col. del Río (Parte baja, barranco)
18	Sánchez Taboada (Av. Sánchez Taboada y Parque Industrial)
19	La cima (casa abandonado)
20	Otay módulos
21	Otro
98	Se rehúsa a contestar

If Q1081 is equal to 21 then skip to Q1082

If not, then skip to instruction before Q1083.

Q1082. Por favor especifica en qué picadero (e.g. nombre del lugar y calles que cruzan o colonia) fué el último contacto cercano con [Response to Q905].

____

If Q686 + Q707 + Q728 + Q749 + Q770 + Q791 + Q812 + Q833 + Q854 + Q875 is greater than 4 and Q908 is not equal to "Se rehúsa a contestar" and Q908 is not equal to "Saltó" then skip to Q1083

If not, then skip to instruction before Q1126.

Q1083. ¿Es [Response to Q908] [MCL5] ([MCN5]) hombre, mujer, transgénero, no-binario u otro? (Elija Uno)

1	Hombre
2	Mujer
3	Mujer Trans
4	Hombre Trans
5	No-binario/ genero no-binario / género fluido/ agénero
6	Otra identidad no en la lista
8	Se rehúsa a contestar

If Q1083 is equal to 6 then skip to Q1084

If not, then skip to Q1085.

Q1084. Por favor especifica la identidad de género de [Response to Q908].

____

Q1085. ¿Dónde vive [Response to Q908] principalmente? (Elija Uno)

1 Mexico

2 Estados Unidos

3 Otro

7 No Sabe

8 Se rehúsa a contestar

If Q1085 is equal to 3 then skip to Q1086

If not, then skip to Q1087.

Q1086. Por favor especifica donde vive principalmente [Response to Q908].

____

Q1087. ¿Conociste a [Response to Q908] por primera vez en los últimos 14 días?

1 Si

0 No

8 Se rehúsa a contestar

Q1088. ¿Cuántas veces has tenido contacto cercano (dentro de 2 metros) con [Response to Q908] en los últimos 14 días?

__ __

98 Se rehúsa a contestar

Q1089. En promedio, durante los días que viste a [Response to Q908], ¿Cuánto tiempo (e.g. horas, minuto) duraste en contacto cercano con él/ella? Entrevistador: el tiempo máximo por día es 24 horas.

__ __ HOURS

__ __ MINUTES

98 Se rehúsa a contestar (Hours)

Q1090. ¿Cuándo fue la última vez que tuviste contacto cercano con [Response to Q908] en los últimos 14 días? Entrevistador: Anote cuántos días hace que el/la participante tuvo contacto cercano por última vez con la persona en cuestión. Si la última vez fue hoy, ingresa 0.

__ __ DíAS

98 Se rehúsa a contestar (Días)

If Q1088 is greater than 1 then skip to Q1091

If not, then skip to Q1092.

Q1091. Durante el último contacto, ¿Por cuantos días consecutivos viste a [Response to Q908]?

__ __

98 Se rehúsa a contestar

MC5FDAY = If(Q1090 is equal to 1, Q1090, If(Q1090 is not equal to 98 and Q1090 is greater than 1, Q1090 + Q1091, "Falta"))

Q1092. La última vez que viste a [Response to Q908], ¿Qué tipo de contacto tuviste con esta persona? (elija todas las que apliquen) (Marque Todas las que Correspondan)

__ Contacto físico, no sexual [e.g. saludo de mano, abrazo, contacto de piel, etc.]

__ Contacto físico, sexual

__ No físico [e.g. conversación en persona, comer/beber, junta]

__ Se rehúsa a contestar

Q1093. Cuando viste a [Response to Q908] durante el contacto, ¿en cual de los siguientes lugares ocurrió? (por favor escoge todas las que apliquen) (Marque Todas las que Correspondan)

__ En casa/casa de alguien

__ Trabajo

__ Restaurante o Café

__ Tiempo Libre (i.e. cine, bar, club nocturno)

__ Escuela/educación

__ Ejercicio o relacionado al estado físico

__ Servicio personal (i.e. corte de cabello, masaje)

__ Servicio religioso

__ Grupo comunitario

__ Parque

__ Calle

__ El Bordo/Canal

__ Picadero

__ Otro

__ Se rehúsa a contestar

If Q1093N is equal to 1 then skip to Q1094

If not, then skip to instruction before Q1095.

Q1094. Por favor especifica el lugar (e.g. el nombre del lugar y las calles que cruzan o la colonia) donde tuviste contacto cercano con [Response to Q908] por última vez.

____

If Q1093A is equal to 1 then skip to Q1095

If not, then skip to instruction before Q1096.

Q1095. Tú nos dijiste que el último contacto cercano con [Response to Q908] fué en tu casa/la casa de alguien más. ¿Dónde se ubica esta casa? ¿Podrías darnos los nombres de las calles que cruzan la intersección más cercana? Entrevistador: Por favor anote los nombres de las calles que cruzan. Si el participante no puede darnos esta información, pregúntele el nombre de la colonia y anote esa información.

____

If Q1093B is equal to 1 then skip to Q1096

If not, then skip to instruction before Q1097.

Q1096. Tú nos dijiste que el último contacto cercano con [Response to Q908] ocurrió en tu trabajo. ¿Nos podrías dar el nombre y la dirección de tu lugar de trabajo? Entrevistador: Si el lugar de trabajo no tiene nombre o dirección, anote cualquier información que se dé para la ubicación (e.g. nombre de calles, intersección más cercana, colonia)

____

If Q1093C is equal to 1 then skip to Q1097

If not, then skip to instruction before Q1098.

Q1097. Tú nos dijiste que la última vez que estuviste en contacto cercano con [Response to Q908] fué en un restaurante/café. ¿Podrías darnos el nombre de este lugar y las calles que cruzan/colonia donde se ubica este restaurante?

____

If Q1093D is equal to 1 then skip to Q1098

If not, then skip to instruction before Q1099.

Q1098. Tú nos dijiste que la última vez que estuviste en contacto cercano con [Response to Q908] fué en un lugar de tiempo libre (e.g. teatro, bar, club nocturno). ¿Podrías darnos el nombre del lugar y las calles que cruzan/colonia donde se ubica este lugar?

____

If Q1093E is equal to 1 then skip to Q1099

If not, then skip to instruction before Q1100.

Q1099. Tú nos dijiste que la última vez que estuviste en contacto cercano con [Response to Q908] fué en tu escuela o lugar de estudio. ¿Podrías darnos el nombre del lugar y las calles que cruzan/colonia donde se ubica este lugar?

____

If Q1093F is equal to 1 then skip to Q1100

If not, then skip to instruction before Q1101.

Q1100. Tú nos dijiste que la última vez que estuviste en contacto cercano con [Response to Q908] fué en un gimnasio/centro fitness. ¿Podrías darnos el nombre del lugar y las calles que cruzan/colonia donde se ubica este lugar?

____

If Q1093G is equal to 1 then skip to Q1101

If not, then skip to instruction before Q1102.

Q1101. Tú nos dijiste que la última vez que estuviste en contacto cercano con [Response to Q908] fué en un lugar donde dan servicios personales (e.g. corte de pelo, masaje). ¿Podrías darnos el nombre del lugar y las calles que cruzan/colonia donde se ubica este lugar?

____

If Q1093H is equal to 1 then skip to Q1102

If not, then skip to instruction before Q1103.

Q1102. Tú nos dijiste que la última vez que estuviste en contacto cercano con [Response to Q908] fué en un lugar de servicio religioso. ¿Podrías darnos el nombre del lugar y las calles que cruzan/colonia donde se ubica este lugar?

____

If Q1093I is equal to 1 then skip to Q1103

If not, then skip to instruction before Q1104.

Q1103. Tú nos dijiste que la última vez que estuviste en contacto cercano con [Response to Q908] fué en un centro comunitario/de reunión grupal. ¿Podrías darnos el nombre del lugar y las calles que cruzan/colonia donde se ubica este lugar?

— —
— — — — — — — — — — — — — —

If Q1093J is equal to 1 then skip to Q1104

If not, then skip to instruction before Q1105.

Q1104. Tú nos dijiste que la última vez que estuviste en contacto cercano con [Response to Q908] fué en un parque. ¿Podrías darnos el nombre del lugar y las calles que cruzan/colonia donde se ubica este lugar?

— —
— — — — — — — — — — — — — —

If Q1093K is equal to 1 then skip to Q1105

If not, then skip to instruction before Q1106.

Q1105. Tú nos dijiste que la última vez que estuviste en contacto cercano con [Response to Q908] fué en la calle. ¿Podrías darnos el nombre del lugar y las calles que cruzan/colonia donde se ubica este lugar?

— —
— — — — — — — — — — — — — —

If Q1093M is equal to 1 then skip to Q1106

If not, then skip to instruction before Q1107.

Q1106. Tú nos dijiste que la última vez que estuviste en contacto cercano con [Response to Q908] fué en un picadero. ¿En qué picadero fué? (Elija Uno)

01	Las Palmitas/Zona Norte
02	Los Pinos/ Zona Norte
03	Las Bombas/ Zona Norte
04	Los Aragón/ Zona Norte
05	La Veinte/ Bordo, Col. Veinte de Noviembre
06	El Azteca/ Cerro Colorado
07	Oxxo, Calle 5ta Zona Norte
08	Bordo/ Los Álamos, Mundo divertido
09	Cañón Guerrero/Maclovio Herrera Colonia Guerrero
10	Col. Guaycura (casa privada)
11	Col. Presidentes (Infonavit edificio)
12	Florido 1era Sección (casa privada)
13	Florido 1era Sección (Bomberos, conecta)
14	Col. Francisco Villa (edificio abandonado)
15	Cañón del Pato/ Colonia Villa
16	Col. Postal (casa privada)
17	Col. del Río (Parte baja, barranco)
18	Sánchez Taboada (Av. Sánchez Taboada y Parque Industrial)
19	La cima (casa abandonado)
20	Otay módulos
21	Otro
98	Se rehúsa a contestar

If Q1106 is equal to 21 then skip to Q1107

If not, then skip to instruction before Q1108.

Q1107. Por favor especifica en qué picadero (e.g. nombre del lugar y calles que cruzan o colonia) fué el último contacto cercano con [Response to Q908].

____

If Q1088 is greater than 1 then skip to Q1108

If not, then skip to instruction before Q1126.

Q1108. Por favor piensa en alguna otra vez que tuviste contacto cercano con [Response to Q908] en los últimos 14 días, aparte del contacto cercano que nos acaba de describir con la persona en cuestión. Cuando decimos alguna otra vez, queremos decir un contacto en el tu viste a [Response to Q908] en persona y luego se separaron, ¿Cuántos dias hace que se vieron?

__ __ DíAS

98 Se rehúsa a contestar (Días)

If Q1108 is less than MC5FDAY then READ: "Este tiempo es más reciente que el tiempo que Ud. indicó que tuvo el último contacto. Por favor realice las correcciones necesarias. TRANSLATION" and skip to Q1108.

If Q1088 is greater than 1 then skip to Q1109

If not, then skip to Q1110.

Q1109. Durante este contacto, ¿Cuántos días consecutivos viste a [Response to Q908]?

__ __

98 Se rehúsa a contestar

Q1110. Cuándo viste a [Response to Q908] en esa ocasión, ¿qué tipo de contacto tuviste con él/ella? (Elije todas las que apliquen) (Marque Todas las que Correspondan)

__ Contacto físico, no sexual [e.g. saludo de mano, abrazo, contacto de piel, etc.]

__ Contacto físico, sexual

__ No físico [e.g. conversación en persona, comer/beber, junta]

__ Se rehúsa a contestar

Q1111. Cuándo viste a [Response to Q908] en esa ocasión, ¿en cuál de los siguientes lugares fue que tuvieron contacto cercano? (elije todas las que apliquen) (Marque Todas las que Correspondan)

__ En casa/casa de alguien

__ Trabajo

__ Restaurante o Café

__ Tiempo Libre (i.e. cine, bar, club nocturno)

__ Escuela/educación

__ Ejercicio o relacionado al estado físico

__ Servicio personal (i.e. corte de cabello, masaje)

__ Servicio religioso

__ Grupo comunitario

__ Parque

__ Calle

__ El Bordo/Canal

__ Picadero

__ Otro

__ Se rehúsa a contestar

If Q1111N is equal to 1 then skip to Q1112

If not, then skip to instruction before Q1113.

Q1112. Por favor especifica el lugar (e.g. nombre del lugar y las calles que cruzan o la colonia) donde fué ese contacto cercano con [Response to Q908].

__ __

If Q1111A is equal to 1 then skip to Q1113

If not, then skip to instruction before Q1114.

Q1113. Tú nos dijiste que el último contacto cercano con [Response to Q908] ocurrió en tu casa/casa de alguien más. ¿Dónde se ubica esta casa? ¿Podrías proveer los nombres de las calles que cruzan la intersección más cercana? Entrevistador: Por favor anote los nombres de las calles que cruzan. Si el participante no puede darnos esta información, pregúntele el nombre de la colonia e ingresar esa información.

— —

If Q1111B is equal to 1 then skip to Q1114

If not, then skip to instruction before Q1115.

Q1114. Tú nos dijiste que el último contacto cercano con [Response to Q908] ocurrió en tu trabajo. ¿Nos podrías dar el nombre y la dirección de tu lugar de trabajo? Entrevistador: Si el lugar de trabajo no tiene nombre o dirección, anote cualquier información que se dé para la ubicación (e.g. nombre de calles, intersección más cercana, colonia)

— —

If Q1111C is equal to 1 then skip to Q1115

If not, then skip to instruction before Q1116.

Q1115. Tú nos dijiste que la última vez que estuviste en contacto cercano con [Response to Q908] fué en un restaurante/café. ¿Podrías darnos el nombre de este lugar y las calles que cruzan/colonia donde se ubica este restaurante?

— —

If Q1111D is equal to 1 then skip to Q1116

If not, then skip to instruction before Q1117.

Q1116. Tú nos dijiste que la última vez que estuviste en contacto cercano con [Response to Q908] fué en un lugar de tiempo libre (e.g. teatro, bar, club nocturno). ¿Podrías darnos el nombre del lugar y las calles que cruzan/colonia donde se ubica este lugar?

— —

If Q1111E is equal to 1 then skip to Q1117

If not, then skip to instruction before Q1118.

Q1117. Tú nos dijiste que la última vez que estuviste en contacto cercano con [Response to Q908] fué en tu escuela o lugar de estudio. ¿Podrías darnos el nombre del lugar y las calles que cruzan/colonia donde se ubica este lugar?

__ __

If Q1111F is equal to 1 then skip to Q1118

If not, then skip to instruction before Q1119.

Q1118. Tú nos dijiste que la última vez que estuviste en contacto cercano con [Response to Q908] ocurrió en un gimnasio/centro fitness. ¿Podrías darnos el nombre del lugar y las calles que cruzan/colonia donde se ubica este lugar?

__ __

If Q1111G is equal to 1 then skip to Q1119

If not, then skip to instruction before Q1120.

Q1119. Tú nos dijiste que la última vez que estuviste en contacto cercano con [Response to Q908] fué en un lugar donde proveen servicios personales (e.g. corte de pelo, masaje). ¿Podrías darnos el nombre del lugar y las calles que cruzan/colonia donde se ubica este lugar?

__ __

If Q1111H is equal to 1 then skip to Q1120

If not, then skip to instruction before Q1121.

Q1120. Tú nos dijiste que la última vez que estuviste en contacto cercano con [Response to Q908] fué en un lugar de servicio religioso. ¿Podrías darnos el nombre del lugar y las calles que cruzan/colonia donde se ubica este lugar?

__ __

If Q1111I is equal to 1 then skip to Q1121

If not, then skip to instruction before Q1122.

Q1121. Tú nos dijiste que la última vez que estuviste en contacto cercano con [Response to Q908] fué en un centro comunitario/de reunión grupal. ¿Podrías darnos el nombre del lugar y las calles que cruzan/colonia donde se ubica este lugar?

— —

If Q1111J is equal to 1 then skip to Q1122

If not, then skip to instruction before Q1123.

Q1122. Tú nos dijiste que la última vez que estuviste en contacto cercano con [Response to Q908] fué en un parque. ¿Podrías darnos el nombre del lugar y las calles que cruzan/colonia donde se ubica este lugar?

— —

If Q1111K is equal to 1 then skip to Q1123

If not, then skip to instruction before Q1124.

Q1123. Tú nos dijiste que la última vez que estuviste en contacto cercano con [Response to Q908] fué en la calle. ¿Podrías darnos el nombre del lugar y las calles que cruzan/colonia donde se ubica este lugar?

— —

If Q1111M is equal to 1 then skip to Q1124

If not, then skip to instruction before Q1125.

Q1124. Tú nos dijiste que la última vez que estuviste en contacto cercano con [Response to Q908] fué en un picadero. ¿En qué picadero fué? (Elija Uno)

01	Las Palmitas/Zona Norte
02	Los Pinos/ Zona Norte
03	Las Bombas/ Zona Norte
04	Los Aragón/ Zona Norte
05	La Veinte/ Bordo, Col. Veinte de Noviembre
06	El Azteca/ Cerro Colorado
07	Oxxo, Calle 5ta Zona Norte
08	Bordo/ Los Álamos, Mundo divertido
09	Cañón Guerrero/Maclovio Herrera Colonia Guerrero
10	Col. Guaycura (casa privada)
11	Col. Presidentes (Infonavit edificio)
12	Florido 1era Sección (casa privada)
13	Florido 1era Sección (Bomberos, conecta)
14	Col. Francisco Villa (edificio abandonado)
15	Cañón del Pato/ Colonia Villa
16	Col. Postal (casa privada)
17	Col. del Río (Parte baja, barranco)
18	Sánchez Taboada (Av. Sánchez Taboada y Parque Industrial)
19	La cima (casa abandonado)
20	Otay módulos
21	Otro
98	Se rehúsa a contestar

If Q1124 is equal to 21 then skip to Q1125

If not, then skip to instruction before Q1126.

Q1125. Por favor especifica en qué picadero (e.g. nombre del lugar y calles que cruzan o colonia) fué el último contacto cercano con [Response to Q908].

____

READ: Ahora te preguntaremos sobre cambios potenciales en los comportamientos de contacto relacionados al SARSCoV-2.
Intenta pensar un poco más allá al tiempo antes de que estuviéramos conscientes de la nueva epidemia de coronavirus.

Q1126. ¿Cuántos amigos o personas con las que tú te asociaste, con quien tú hablaste sobre cosas que eran importantes para ti ya no puedes ver por la pandemia?

__ __ __

998 Se rehúsa a contestar

If Q1126 is greater than 0 and Q1126 is not equal to 998 then skip to Q1127

If not, then skip to instruction before Q1135.

Q1127. **De las personas que ya no puedes ver, cuántas de ellas eran:** Parejas Sexuales

__ __ __

998 Se rehúsa a contestar

If Q1127 is not equal to 998 and Q1127 is greater than Q1126 then READ: "Este número es más grande que el número total. Por favor realice las correcciones necesarias." and skip to Q1127.

If Q1127 is not equal to 998 and Q1126 is greater than Q1127 then skip to Q1128

If not, then skip to instruction before Q1129.

Q1128. **De las personas que ya no puedes ver, cuántas de ellas eran:** amigos

__ __ __

998 Se rehúsa a contestar

If Q1128 is not equal to 998 and Q1127 is not equal to 998 and Q1128 is greater than Q1126 - Q1127 then READ: "Este número es más grande que el número total. Por favor realice las correcciones necesarias." and skip to Q1128.

If Q1127 is not equal to 998 and Q1128 is not equal to 998 and Q1126 is greater than Q1127 + Q1128 then skip to Q1129

If not, then skip to instruction before Q1130.

Q1129. **De las personas que ya no puedes ver, cuántas de ellas eran:** Vecinos o compañeros de casa

— — —

998 Se rehúsa a contestar

If Q1129 is not equal to 998 and Q1127 is not equal to 998 and Q1128 is not equal to 998 and Q1129 is greater than Q1126 - Q1127 - Q1128 then READ: "Este número es más grande que el número total. Por favor realice las correcciones necesarias." and skip to Q1129.

If Q1127 is not equal to 998 and Q1128 is not equal to 998 and Q1129 is not equal to 998 and Q1126 is greater than Q1127 + Q1128 + Q1129 then skip to Q1130

If not, then skip to instruction before Q1131.

Q1130. **De las personas que ya no puedes ver, cuántas de ellas eran:** Compañeros de trabajo o jefes

— — —

998 Se rehúsa a contestar

If Q1130 is not equal to 998 and Q1127 is not equal to 998 and Q1128 is not equal to 998 and Q1129 is not equal to 998 and Q1130 is greater than Q1126 - Q1127 - Q1128 - Q1129 then READ: "Este número es más grande que el número total. Por favor realice las correcciones necesarias." and skip to Q1130.

If Q1127 is not equal to 998 and Q1128 is not equal to 998 and Q1129 is not equal to 998 and Q1130 is not equal to 998 and Q1126 is greater than Q1127 + Q1128 + Q1129 + Q1130 then skip to Q1131

If not, then skip to instruction before Q1132.

Q1131. **De las personas que ya no puedes ver, cuántas de ellas eran:** Familia- familiares por sangre o matrimonio

— — —

998 Se rehúsa a contestar

If Q1131 is not equal to 998 and Q1130 is not equal to 998 and Q1127 is not equal to 998 and Q1128 is not equal to 998 and Q1129 is not equal to 998 and Q1131 is greater than Q1126 - Q1127 - Q1128 - Q1129 - Q1130 then READ: "Este número es más grande que el número total. Por favor realice las correcciones necesarias." and skip to Q1131.

If Q1127 is not equal to 998 and Q1128 is not equal to 998 and Q1129 is not equal to 998 and Q1130 is not equal to 998 and Q1131 is not equal to 998 and Q1126 is greater than Q1127 + Q1128 + Q1129 + Q1130 + Q1131 then skip to Q1132

If not, then skip to instruction before Q1133.

Q1132. **De las personas que ya no puedes ver, cuántas de ellas eran:** Relacionadas a las drogas o alcohol

— — —

998 Se rehúsa a contestar

If Q1132 is not equal to 998 and Q1131 is not equal to 998 and Q1130 is not equal to 998 and Q1127 is not equal to 998 and Q1128 is not equal to 998 and Q1129 is not equal to 998 and Q1132 is greater than Q1126 - Q1127 - Q1128 - Q1129 - Q1130 - Q1131 then READ: "Este número es más grande que el número total. Por favor realice las correcciones necesarias." and skip to Q1132.

If Q1127 is not equal to 998 and Q1128 is not equal to 998 and Q1129 is not equal to 998 and Q1130 is not equal to 998 and Q1131 is not equal to 998 and Q1132 is not equal to 998 and Q1126 is greater than Q1127 + Q1128 + Q1129 + Q1130 + Q1131 + Q1132 then skip to Q1133

If not, then skip to instruction before Q1134.

Q1133. **De las personas que ya no puedes ver, cuántas de ellas eran:** Otra relación (especifica)

— — —

998 Se rehúsa a contestar

If Q1133 is not equal to 998 and Q1132 is not equal to 998 and Q1131 is not equal to 998 and Q1130 is not equal to 998 and Q1127 is not equal to 998 and Q1128 is not equal to 998 and Q1129 is not equal to 998 and Q1133 is greater than Q1126 - Q1127 - Q1128 - Q1129 - Q1130 - Q1131 - Q1132 then READ: "Este número es más grande que el número total. Por favor realice las correcciones necesarias." and skip to Q1133.

If Q1133 is greater than 0 then skip to Q1134

If not, then skip to instruction before Q1135.

Q1134. Por favor, especifica qué otra relación.

____

READ: Ahora, intenta recordar las primeras dos semanas de Febrero 2020 (o un periodo típico de 14 días en 2020 antes de que estuviéramos conscientes de la nueva pandemia de coronavirus).

Q1135. **¿Normalmente con cuántas personas tenías contacto cercano en el trascurso de esos 14 días?** Recuerda, estamos interesados en las personas con las que tu has tenido:
Contacto no físico (con 2 metros de distancia) que duró **más de una hora**
O
Contacto físico
Por favor agrega el número de contactos en cada categoría. De nuevo, puedes fijarte tranquilamente en tu teléfono o calendario para recordar mejor

___

998 Se rehúsa a contestar

If Q1135 is greater than 0 and Q1135 is not equal to 998 then skip to Q1136

If not, then skip to Q1150.

Q1136. **¿Cuántos de estos contactos cercanos estuvieron…**Viviendo en Estados Unidos**?**

___

997 No Sabe

998 Se rehúsa a contestar

If Q1136 is not equal to 997 and Q1136 is not equal to 998 and Q1136 is greater than Q1135 then READ: "Este número es más grande que el número total. Por favor realice las correcciones necesarias." and skip to Q1136.

If Q1135 is greater than Q1136 then skip to Q1137

If not, then skip to instruction before Q1138.

Q1137. **¿Cuántos de estos contactos cercanos estuvieron…**Viviendo en México**?**

— — —

997 No Sabe

998 Se rehúsa a contestar

If Q1136 is not equal to 997 and Q1136 is not equal to 998 and Q1137 is not equal to 997 and Q1137 is not equal to 998 and Q1137 is greater than Q1135 - Q1136 then READ: "Este número es más grande que el número total. Por favor realice las correcciones necesarias." and skip to Q1137.

Q1138. **¿Cuántos de estos contactos cercanos fueron…** Hombres?

— — —

998 Se rehúsa a contestar

If Q1138 is not equal to 998 and Q1138 is greater than Q1135 then READ: "Este número es más grande que el número total. Por favor realice las correcciones necesarias." and skip to Q1138.

If Q1135 is greater than Q1138 then skip to Q1139

If not, then skip to instruction before Q1140.

Q1139. **¿Cuántos de estos contactos cercanos fueron…** Mujer**?**

— — —

997 No Sabe

998 Se rehúsa a contestar

If Q1138 is not equal to 998 and Q1139 is not equal to 998 and Q1139 is greater than Q1135 - Q1138 then READ: "Este número es más grande que el número total. Por favor realice las correcciones necesarias." and skip to Q1139.

If Q1135 is greater than Q1138 + Q1139 then skip to Q1140

If not, then skip to instruction before Q1141.

Q1140. **¿Cuántos de estos contactos cercanos fueron…** Hombre Transgénero**?**

— — —

997	No Sabe
998	Se rehúsa a contestar

If Q1140 is not equal to 998 and Q1140 is greater than Q1135 then READ: "Este número es más grande que el número total. Por favor realice las correcciones necesarias." and skip to Q1140.

If Q1135 is greater than Q1138 + Q1139 + Q1140 then skip to Q1141

If not, then skip to instruction before Q1142.

Q1141. **¿Cuántos de estos contactos cercanos fueron…** Mujer Transgénero**?**

— — —

997	No Sabe
998	Se rehúsa a contestar

If Q1140 is not equal to 998 and Q1141 is not equal to 998 and Q1141 is greater than Q1135 - Q1140 then READ: "Este número es más grande que el número total. Por favor realice las correcciones necesarias." and skip to Q1141.

If Q1135 is greater than Q1138 + Q1139 + Q1140 + Q1141 then skip to Q1142

If not, then skip to instruction before Q1143.

Q1142. **¿Cuántos de estos contactos cercanos fueron…** No-binario/ género no-binario / género fluido/ agénero**?**

— — —

997	No Sabe
998	Se rehúsa a contestar

If Q1142 is not equal to 998 and Q1140 is not equal to 998 and Q1141 is not equal to 998 and Q1142 is greater than Q1135 - Q1140 - Q1141 then READ: "Este número es más grande que el número total. Por favor realice las correcciones necesarias." and skip to Q1142.

If Q1135 is greater than Q1138 + Q1139 + Q1140 + Q1141 + Q1142 then skip to Q1143

If not, then skip to instruction before Q1144.

Q1143. **¿Cuántos de estos contactos cercanos fueron...** Otra identidad no incluida en la lista?

__ __ __

997 No Sabe

998 Se rehúsa a contestar

If Q1143 is not equal to 998 and Q1142 is not equal to 998 and Q1140 is not equal to 998 and Q1141 is not equal to 998 and Q1143 is greater than Q1135 - Q1140 - Q1141 - Q1142 then READ: "Este número es más grande que el número total. Por favor realice las correcciones necesarias." and skip to Q1143.

If Q1143 is greater than 0 then skip to Q1144

If not, then skip to Q1145.

Q1144. Por favor, especifica.

__ __

Q1145. **¿Cuántos de estos contactos cercanos eran...**<18 años de edad**?**

__ __ __

997 No Sabe

998 Se rehúsa a contestar

If Q1145 is not equal to 997 and Q1145 is not equal to 998 and Q1145 is greater than Q1135 then READ: "Este número es más grande que el número total. Por favor realice las correcciones necesarias." and skip to Q1145.

If Q1135 is greater than Q1145 then skip to Q1146

If not, then skip to instruction before Q1147.

Q1146. **¿Cuántos de estos contactos cercanos tenían...**18-35 años**?**

— — —

997 No Sabe

998 Se rehúsa a contestar

If Q1145 is not equal to 997 and Q1145 is not equal to 998 and Q1146 is not equal to 997 and Q1146 is not equal to 998 and Q1146 is greater than Q1135 - Q1145 then READ: "Este número es más grande que el número total. Por favor realice las correcciones necesarias." and skip to Q1146.

If Q1135 is greater than Q1145 + Q1146 then skip to Q1147

If not, then skip to instruction before Q1148.

Q1147. **¿Cuántos de estos contactos cercanos tenían...**>35 años**?**

— — —

997 No Sabe

998 Se rehúsa a contestar

If Q1145 is not equal to 997 and Q1145 is not equal to 998 and Q1146 is not equal to 997 and Q1146 is not equal to 998 and Q1147 is not equal to 997 and Q1147 is not equal to 998 and Q1147 is greater than Q1135 - Q1145 - Q1146 then READ: "Este número es más grande que el número total. Por favor realice las correcciones necesarias." and skip to Q1147.

Q1148. **¿Con cuántos de estos contactos cercanos tuviste sexo vagina o anal en el transcurso de esos 14 días?**

— — —

998 Se rehúsa a contestar

If Q1148 is not equal to 998 and Q1148 is greater than Q1135 then READ: "Este número es más grande que el número total. Por favor realice las correcciones necesarias." and skip to Q1148.

Q1149. **¿Con cuántos de estos contactos cercanos usaste drogas en el transcurso de esos 14 días?**

__ __ __

998 Se rehúsa a contestar

If Q1149 is not equal to 998 and Q1149 is greater than Q1135 then READ: "Este número es más grande que el número total. Por favor realice las correcciones necesarias." and skip to Q1149.

Q1150. **Entrevistador:** Qué tan honestas creen Ud. que sea el/la participante en sus respuestas? (Elija Uno)

1 Para nada honesto

2 Un poco deshonesto

3 Un poco honesto

4 Muy honesto

If Q204 is equal to 4 and (Q195 is equal to 1 or Q197 is equal to 1 or Q207 is equal to 1 or Q209 is equal to 1 or Q211 is equal to 1 or Q225 is equal to 1 or Q227 is equal to 1 or Q231 is equal to 1) or Q229 is equal to 1 then skip to Q1151

If not, then skip to instruction before Q1152.

Q1151. ENCUESTADOR: Acabas de recibir la instrucción de hacer una recomendación médica para Clínica de TB. Por favor indica que **SI** para recibir la instrucción al final de la entrevista. (Elija Uno)

1 Si

If Q191 is equal to 1 or Q193 is equal to 1 or Q195 is equal to 1 or Q197 is equal to 1 or Q199 is equal to 1 or Q201 is equal to 1 or Q203 is equal to 1 or Q205 is equal to 1 or Q207 is equal to 1 or Q209 is equal to 1 or Q213 is equal to 1 or Q215 is equal to 1 or Q217 is equal to 1 or Q219 is equal to 1 or Q221 is equal to 1 or Q223 is equal to 1 or Q227 is equal to 1 then skip to Q1152

If not, then skip to end of questionnaire.

Q1152. ENCUESTADOR: Acabas de recibir la instrucción de hacer una recomendación médica para TB. Por favor indica que **SI** para recibir la instrucción al final de la entrevista. (Elija Uno)

1 Si

READ: Fin de entrevista.
